# Root transcriptome reveals efficient cell signaling and energy conservation key to aluminum toxicity tolerance in acidic soil adapted rice genotype

**DOI:** 10.1038/s41598-020-61305-7

**Published:** 2020-03-12

**Authors:** Wricha Tyagi, Julia S. Yumnam, Devyani Sen, Mayank Rai

**Affiliations:** School of Crop Improvement, College of Post-Graduate Studies, Central Agricultural University (Imphal), Umroi Road, Umiam, Meghalaya 793103 India

**Keywords:** Transcriptomics, Plant stress responses

## Abstract

Aluminium (Al) toxicity is the single most important contributing factor constraining crop productivity in acidic soils. Hydroponics based screening of three rice genotypes, a tolerant (ARR09, AR), a susceptible (IR 1552, IR) and an acid soil adapted landrace (Theruvii, TH) revealed that AR accumulates less Al and shows minimum decrease in shoot and root biomass under Al toxicity conditions when compared with IR. Transcriptome data generated on roots (grown in presence or absence of Al) led to identification of ~1500 transcripts per genotype with percentage annotation ranging from 21.94% (AR) to 29.94% (TH). A total of 511, 804 and 912 DEGs were identified in genotypes AR, IR and TH, respectively. IR showed upregulation of transcripts involved in exergonic processes. AR appears to conserve energy by downregulating key genes of glycolysis pathway and maintaining transcript levels of key exergonic step enzymes under Al stress. The tolerance in AR appears to be as a result of novel mechanism as none of the reported Al toxicity genes or QTLs overlap with significant DEGs. Components of signal transduction and regulatory machinery like transcripts encoding zinc finger protein, calcieurin binding protein and cell wall associated transcripts are among the highly upregulated DEGs in AR, suggesting increased and better signal transduction in response to Al stress in tolerant rice. Sequencing of *NRAT*1 and glycine-rich protein A3 revealed distinct haplotype for *indica* type AR. The newly identified components of Al tolerance will help in designing molecular breeding tools to enhance rice productivity in acidic soils.

## Introduction

Soil acidity affects 40% of the global arable land^1^. Forty nine million hectares of the total land area of India is acidic in nature^2^, a majority of which is in the North-Eastern Region, with an estimated 65% of area having pH below 5.5^3^.

Aluminium (Al) is the most abundant metal and non-essential to plants. Enhanced soluble soil Al at low pH is the most important limiting factor in 67% of the acidic soils^4^. Under acidic condition, Al becomes more soluble. When pH drops, the soil bound Al is released in soluble forms such as Al(OH)_2_^+^ and Al(H_2_O)_6_^3+^, (commonly known as Al^3+^) and becomes phytotoxic to the plants. When present in micromolar levels, Al hinders root elongation affecting uptake of water and nutrients^5^. Al toxicity causes fixation of phosphorous in soils and on root surfaces. As a result of which the crop productivity, especially in the upland conditions (where Al toxicity predominantly exists), gets severely affected.

Differential tolerance to Al toxicity attributed to variation in structure and function of roots. Short exposure to low Al concentration may only inhibit cell elongation. However, continuous exposure to high Al concentration inhibits both cell elongation and division and causes abnormal root morphology^4,5^. The mechanism of inhibition of root growth and aluminium tolerance is still not well understood.

The term Al resistance is used for plants which retain sizeable yields in soils having Al toxicity^6^. Among the small grained cereal crops, rice is the most Al tolerant with japonica varieties being more tolerant than *indica* varieties^7^. The Al toxicity tolerance in rice is a multigenic trait having complex genetics as supported by identification of 32 non-overlapping quantitative trait loci (QTLs) from different bi-parental studies^8–12^, and identification of 48 novel loci for Al resistance by genome-wide association mapping^13^. Association study of Chinese rice landraces using genome-wide simple sequence repeats (SSRs) has suggested several genomic regions associated with Al toxicity tolerance^14^. Certain genes/loci of vital importance for better understanding have been suggested for Al toxicity tolerance^15^.

Several genes (*OsSTAR1* (Sensitive to Al Rhizotoxicity 1)*, OsSTAR2* (Sensitive to Al Rhizotoxicity 2)), transcription factors (*OsART1* (Aluminium rhizotoxicity 1)) and transporters (*ALMT, OsNRAT1 (Nramp (natural resistance-associated macrophage protein) aluminum transporter 1*), *OsFRDL4* (*FERRIC REDUTASE DEFECTIVE LIKE* 1)*, OsALS1* (*Al-Sensitive 1*)) associated with Al toxicity resistance have been identified till date^6,16^. Aluminium activated Malate Transporter (ALMT) was the first aluminium toxicity tolerance gene to be identified in wheat^7,17^. ALMT protein is involved in efflux of malate from roots^6^. A MATE (Multidrug And Toxic compound Extrusion) ortholog of SbMATE (*Sorghum bicolor* MATE) in rice is *FRDL4*^18^, which transports citrate transporter and can form complexes with Al. An aluminium transporter localized in the PM, *NRAT1*, is responsible for transporting Al across PM^19^. A vacuolar ABC (ATP Binding Cassette) transporter, ALS1, sequesters Al into vacuoles^20^, and in this sequestration process NRAT1 works together with ALS1^6^. The Al responsive genes, *STAR1* and *STAR2* were isolated by mutant screen and these together work as an efflux ABC transporter to transport UDP-glucose into cell wall^21,22^. This modification of cell wall by altering carbohydrate composition results in reduce Al binding and thereby, accumulation. A zinc finger transcription factor, *ART1*, was characterized as an ortholog of *Arabidopsis* transcription factor, *AtSTOP1*^23–25^. Os*ART1* regulates at least 30 down-stream genes including *STAR1, STAR2*, *NRAT1*, *FRDL4* and *ALS* mostly via a specific *cis*-element^26^. Apart from the aforementioned genes, two rice genes located on PM, an Mg uptake transporter (*OsMGT1*; *Magnesium Transporter 1*) and a small peptide (*OsCDT3*; *Cadmium Tolerance 3*), stop entry of Al inside roots by binding to it^27,28^.

The above mentioned studies indicate that there may be multiple genes and molecular mechanisms affecting Al toxicity tolerance in rice. However, a comprehensive understanding of nature and role of such genes in rice is lacking. It has also been suggested that high level of rice Al resistance is due to synergy of mechanisms involving multiple genes^6^.

The current study was undertaken to gain a better understanding of Al toxicity tolerance in *Indica* rice genotypes adapted to upland acidic soils of North Eastern India, where aluminum toxicity is a major problem. The aim of this study was to generate seedling stage root transcriptome (RNAseq) data on three different rice genotypes, a tolerant, a susceptible and an adapted landrace, to identify genes differentially regulated under Al toxic conditions. The selection of genotypes ARR09 (AR), Theruvii (TH) and IR1552 (IR) was based on (a) differential response in upland acidic field conditions (pH < 4.2) and (b) genetic diversity^29^. While AR is an upland genotype from North eastern state of Arunachal Pradesh, TH is an acidic soil adapted landrace from Meghalaya and IR is an international check for Al sensitivity. During field evaluation for performance under upland acidic field conditions, AR showed good performance (infact used as tolerant local check) while IR showed very poor performance (unpublished data). The genotype, TH, is a landrace with low yield. The analysis of transcriptome has led to identification of Al responsive genes and pathways associated with Al tolerance, which in addition to better insight, would lead to development of novel markers and molecular breeding tools for enhancing rice productivity in Al toxic acidic soils.

## Results

### Selection of rice genotypes for transcriptome study

**Figure 1:**
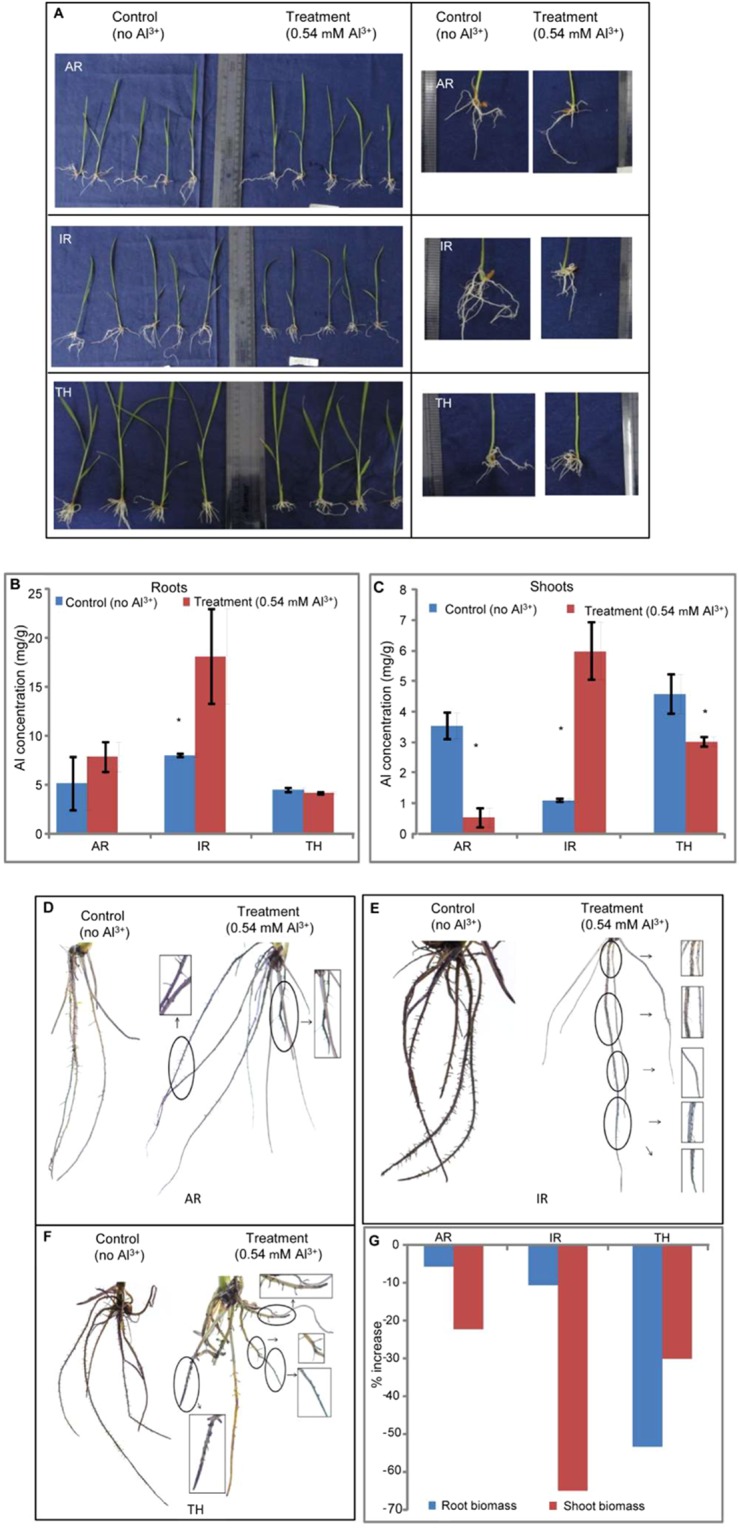
Morphological variation for 10 days old three rice genotypes grown in modified Magnavaca nutrient in presence or absence of Al^3+^ Variation in the root growth pattern (**A**), aluminium content in root (**b**) and shoot (**C**), 2% haematoxylin staining (**D–F**) of 10 days old rice genotypes grown under control (0 Al^3+^) and treatment (0.54 mM of Al³^+^) for five days (**G**) root and shoot biomass of treated plants expressed as respective percentage of plants grown under control condition. The encircled portions (**D–F**) represent the root portion where Al^3+^ gets accumulated Values are means (n = 3), and bars indicate ± standard errors. The significant values are indicated by *Names of the genotypes are given on the X-axis.

Hydroponics experiment conducted for aluminium toxicity tolerance (0.54 mM) proved that 5 days treatment is able to distinguish between tolerant (AR), susceptible genotype (IR) and acidic soil adapted landrace (TH) based on root thickness, lateral roots (Fig. 1A) and differential haematoxylin staining (Fig. 1C–E). Our previous experiments using background SSR markers had suggested that these three rice genotypes are genetically diverse^29^. The comparison of Al^3+^ accumulation, as indicated by aluminum estimation suggests that susceptible genotype IR accumulates significant amounts of Al in its roots whereas AR and TH do not. Hematoxylin staining (Fig. 1A), with RRG (relative root growth) data, showed that root inhibition and Al^3+^ accumulation in roots are related. RRG value was least in TH followed by IR and AR (Table 1).

**Table 1:** Physiological detail in rice genotypes showing contrasting response to aluminium toxicity.

Sl. No.	Genotype Code	Name	Aluminium content in roots (5-day treated seedlings) mg/g	Response to Al toxicity
1	AR	ARR 09	16.33	Tolerant
2	TH	Theruvii	20.82	Adapted landrace
3	IR	IR 1552	26.98	Susceptible

### Data generation and assembly of transcriptome

The transcriptome data generated on the three genotypes was checked for quality (QC) (Supplemental File S1) and the QC passed reads ranged from 0.41 million reads (AR_T) (Table 2) to 2.86 million reads (AR_C) (Table 2). These QC passed transcripts were then assembled. For assembly without reference reads>200 bp length were considered. The MIRA assembler provided a better integrated assembly of transcriptome, as compared to TRINITY (Tables 3 and 4). Also, the number of transcripts that were >200 bp in length was ~1500 per genotype for MIRA. A merged transcriptome assembly was done for each of the genotype (eg. ARC_ART denotes both samples of genotype AR merged to form the assembly) and this was used for further annotation of transcripts.

**Table 2:** Brief summary of QC passed data.

Sample Name	Number of quality reads (reads in million)
AR control	2.86
AR treatment	0.41
IR control	2.81
IR treatment	1.59
TH control	0.86
TH treatment	1.55

**Table 3:** Transcriptome assembly details.

Sample Assembler	0–199 bp	200–499 bp	500–2000 bp	Total
AR control_AR treatment_MIRA	88,865	1527	26	90418
AR control_AR treatment_TRINITY	8,821	253	24	9098
IR control_IR treatment_MIRA	10,7279	1189	27	108495
IR control_IR treatment_TRINITY	10,472	280	26	10778
TH control_TH treatment_MIRA	55,223	582	17	55822
THcontrol_TH treatment_TRINITY	2,996	140	13	3149

**Table 4:** Annotation summary.

Sample	Total Number of Input Transcripts	# Annotated Transcripts	# Unannotated Transcripts	% of Annotation
ARC_ART	25120	5512	19608	21.94
IRC_IRT	24725	5821	18904	23.54
THC_THT	8506	2547	5959	29.94

### Mapping statistics and annotation of transcripts

**Figure 2:**
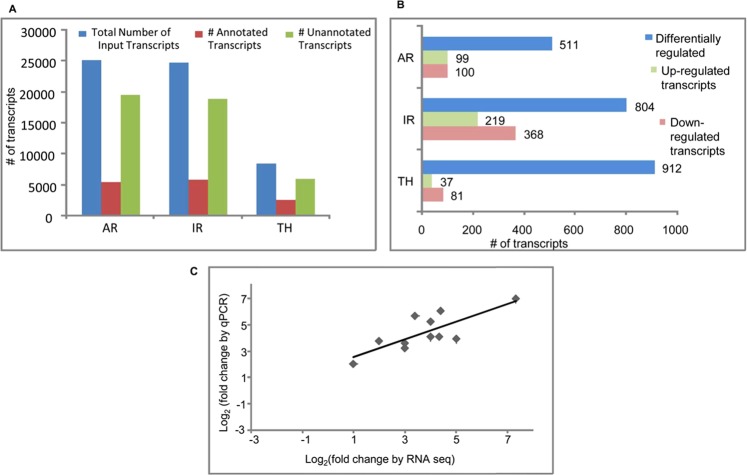
Differential gene expression under Al stress condition. (**A**) The total, unannotated and annotated transcripts identified for the three rice genotypes AR, IR and TH is shown in the bar graph. (**B**) Bar diagram showing the number of up- and down- regulated genes in response to Al toxicity in the roots of AR, IR and TH. The total number of genes differentially expressed under each condition is given on the top of each bar (**C**). The correlation of gene expression results obtained from qPCR analysis and RNA-seq for fifteen selected genes.

The QC passed transcripts were considered as expressed. A total of 8,506, 24,725 and 25,120 transcripts passed the cut-off for TH, IR and AR, respectively (Fig. 2A). The percentage annotation ranged from 21.94% (AR), 23.54% (IR) to 29.94% (TH) (Fig. 2A, Supplementary Tables S1–S3) suggesting transcriptionally active regions which are uncharacterized till date. The transcripts that passed QC were checked for differential expression in presence or absence of Al^3+^. A cut off p value (<0.05 and a minimum read count of 10) was used to identify differentially expressed genes (DEGs). Significant DEGs (>2 log_2_ fold expression) were also identified for each of the three genotypes. As shown in Fig. 2B, 511 DEGs were identified in AR out of which 99 and 100 transcripts were significantly upregulated and downregulated, respectively. Similarly, 804 DEGs were identified in IR, out of which 219 were upregulated and 368 were downregulated. In the case of TH, out of a total of 912 DEGs, only 37 transcripts showed significant upregulation in presence of Al^3+^ (Fig. 2B).

### Validation of selected up-regulated DEGs by Q-PCR

A set of 15 genes were randomly selected and validated by qRT–PCR analysis in three biological replicates for confirming the results obtained from DEG analysis. The analysis revealed similar expression pattern for all the selected genes in qPCR analysis as observed from RNA-seq data. The statistical analysis also showed significant association (r^2^ = 0.78) between the results of qPCR and RNA-seq data analyses (Fig. 2C).

### Analysis of stress-responsive genes reveals genes specific for Al toxicity conditions

**Figure 3:**
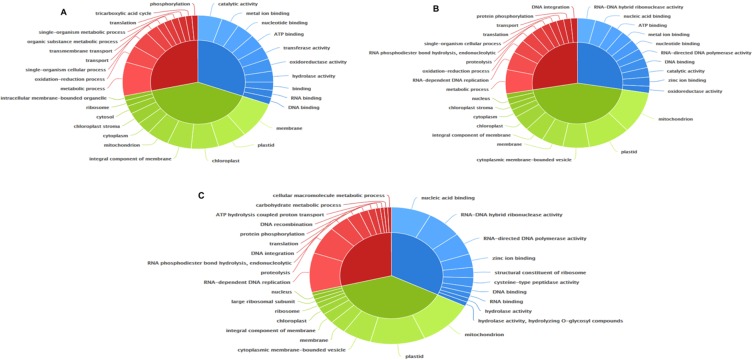
Top ten GO terms in each of the three categories, molecular function, cellular component and biological process for the three rice genotypes AR, IR and TH. The top GO terms for molecular function (blue), cellular component (green) and biological process (red) categorised for genotypes AR (**A**), IR (**B**) and TH (**C**).

Using rice Nipponbare genome as reference, transcripts were assigned gene ontology (GO) terms in three different categories: biological process, molecular function and cellular component. Using top 10 GO terms in each of the three categories, the figure generated for the tolerant and susceptible genotypes revealed a clear difference. In the case of biological activity, while the tolerant genotype AR showed increase in GO terms associated with metabolic processes, oxidation and reduction and cellular processes (Fig. 3A), IR showed increase in DNA replication along with metabolic processes and oxidation and reduction processes (Fig. 3B). The genotype TH under Al toxicity conditions shows increase in GO terms associated with DNA replication, proteolysis and endonucleolytic activity (Fig. 3C). For molecular function GOSlim terms, the largest number of transcripts for AR belonged to catalytic activity, metal ion and nucleotide binding activity. In the case of IR, transcripts with ribonuclease activity, nucleic acid and ATP binding activity were in greater number. The largest number of transcripts belonged to nucleic acid binding, ribonuclease and polymerase activity for genotype TH. Among the cellular components, transcripts related to membrane, plastid, chloroplast and integral component of membrane were largest in AR whereas transcripts related to mitochondria, plastid and membrane bound vesicle were largest in IR. Transcripts related to mitochondria, plastid and membrane bound vesicle were largest in TH.

**Figure 4:**
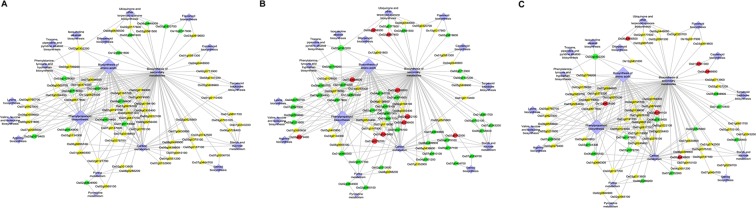
GO enrichment in response to Al stress across three rice genotypes. Significantly, enriched GO categories in the up-regulated genes in tolerant genotype AR (**a**) and susceptible genotype IR (**B**) and TH (**C**) are shown. The genes were analyzed using BiNGO and the biological process terms showing significant enrichment are shown. Node size is proportional to the number of genes in each category and shades represent the significance level (yellow—no significant difference; red −>log_2_ fold upregulated; green − <log_2_ fold downregulated; scale, P = 005 to P < 00000005).

The functionally annotated genes were mapped for the three genotypes to check whether any particular pathway related genes were differentially regulated in tolerant and susceptible genotypes (Fig. 4). It was observed that the susceptible genotype (IR) showed upregulation of enzymes like phosphoglycerate kinase involved in exergonic process (02g0169300). In the case of IR, an ABC transporter (01g0732500) was also upregulated. The acidic soil adapted TH showed an increase in transcript encoding citrate synthase (02g0194100). The genotype AR on other hand showed no differential expression for the transcripts for the two enzymes and downregulation for enolase (06g0136600); phosphofructokinase (05g0524400) and fructose bisphosphate (01g0118000) (Fig. 4A). In TH, transcripts for methionine synthase (012g0624000) and zeaxanthin epoxidase (04g0448900) were also upregulated. Arginine biosynthetic gene (03g0279400) and lignin biosynthetic genes (06g0656500 and 02g0697400) were significantly upregulated in IR (Fig. 4B) but downregulated in AR.

**Figure 5:**
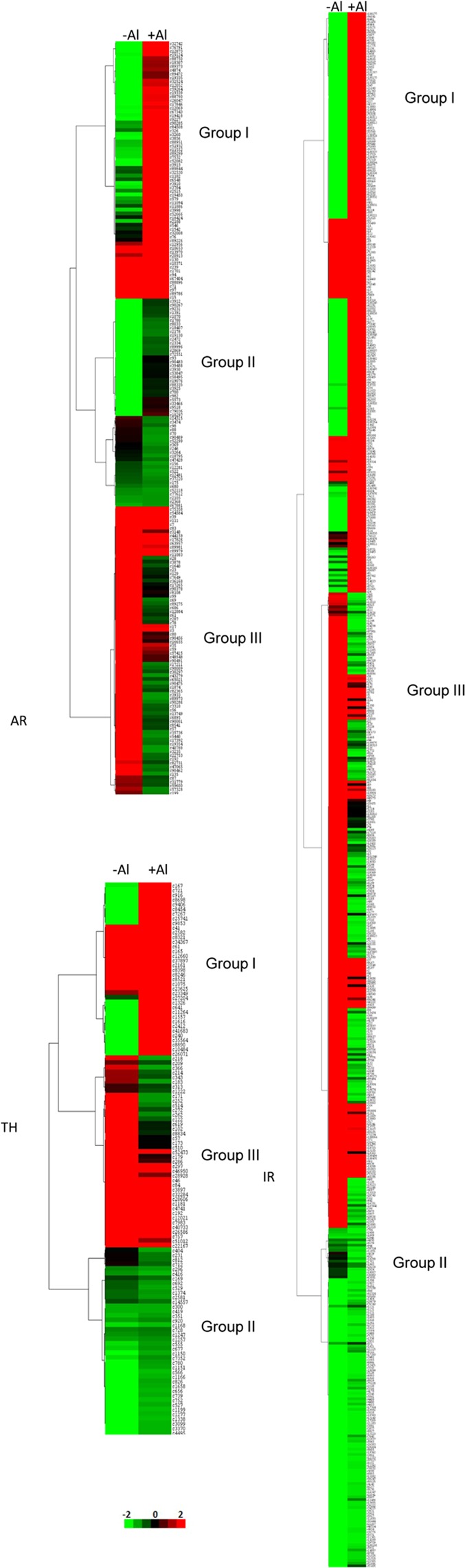
Heat map representation and K-means clustering of expression profiles of genes differentially expressed under Al stress conditions in AR (**A**), IR (**B**) and TH (**C**). The ion torrent RNA-seq data were re-analyzed, and the FPKM values were log_2_ transformed and heat map generated using MeV v4.11 software. Clustering was performed on log_2_ fold change for each gene under Al stress conditions when compared with control condition across the three genotypes. Genes exhibiting a similar pattern of expression under Al stress conditions cluster together. Red and green colours denote control (no Al^3+^) and treatment (0.54 mM Al^3+^), respectively. Bar at the bottom represents log_2_ fold values.

We also performed K-means clustering analysis for the three genotypes in response to Al stress condition and generated major clusters. Among the clusters generated, three groups of genes with distinct gene expression patterns (Fig. 5) were identified. Group I genes were up-regulated under Al stress. Group II genes were down-regulated under normal conditions. Group III genes were down-regulated under Al stress.

### Al toxicity response in rice genotypes with respect to known genes/QTLs

**Figure 6:**
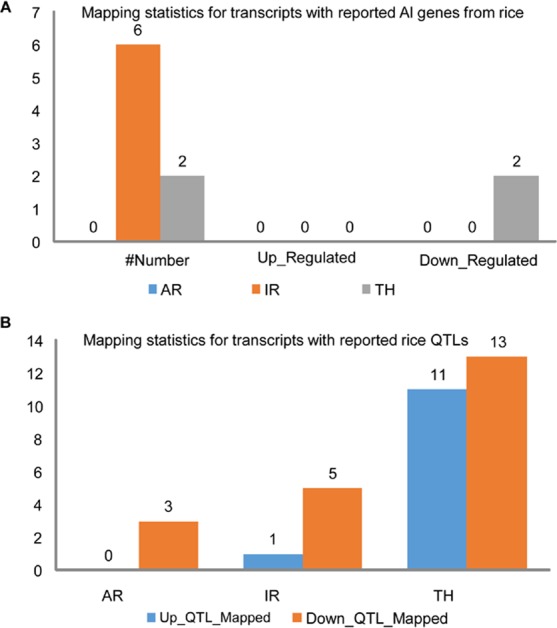
Mapping statistics of DEGs against already reported (**A**) genes and (**B**) QTLs for Al toxicity tolerance in rice. Total number of reported genes mapped- Number; Upregulated DEGs- Up_Regulated, Downregulated DEGs; Down_Regulated; Upregulated DEGs mapped to a reported rice QTL- Up_QTL_mapped; Downregulated DEGs mapped to reported rice QTL- Down_QTL_mapped.

A set of 54 already reported rice genes (Supplementary Table S4) playing different molecular and physiological roles in Al toxicity were cross-referred in the significantly expressed dataset produced for the three rice genotypes in this study. Surprisingly, DEGs from the tolerant genotype AR show no overlap (Fig. 6A). In fact, across genotypes, none of the upregulated DEGs show any overlap with reported genes for Al toxicity. In TH, two significantly downregulated transcripts show an overlap with an unknown protein and an expansin (Fig. 6A, Supplementary Table S5). A set of nine QTLs lying on chromosomes 1, 2, 6, 9 and 12 of rice have been previously identified using hydroponics based screening^13^ (Supplementary Table S6). The DEGs from tolerant genotype AR do not overlap with these reported QTLs. However, TH DEGs overlap with QTL Alt_TRG1.1_, Alt_PRG1.1_, Alt_PRG6.1_, Alt_LRG9.1_, Alt_TRG1.1_ and Alt_TRG12.1_. IR DEGs overlap with QTL Alt_TRG1.1_, Alt_PRG1.1_ and Alt_PRG6.1_. Among the 7 genomic loci/segments chosen, none of them harbored even one up regulated gene. Down regulated genes were found in chromosomes (chr #) 1, 4, 5 and 7. Chromosome 1 harbored the maximum number of down regulated genes (13 genes). ART1 is a transcription factor which is constitutively expressed and its expression is not affected by Al. It regulates a set of 31 genes in rice. We checked the status of these 31 genes in DEGs of the three genotypes. It was found that the DEGs found in the three genotypes are not regulated by ART1 (data not shown).

Significantly expressed DEGs were also mapped to check overlap with reported rice QTLs. In genotype AR, three downregulated DEGs map to 2 QTLs for plant hopper resistance (chr # 2) and ultra violet light resistance (chr # 1) (Fig. 6B, Supplementary Table S5). Genotype IR showed overlap with QTLs for plant hopper resistance (chr # 2), ultra violet light resistance (chr # 1 and 4) and spikelet number (chr # 2 and 9).Genotype TH showed maximum overlap (a total of 16 QTLs) including QTLs for spikelet density (chr # 3), spikelet number (chr # 2 and 9), spikelet sterility (chr # 5), tiller number (chr # 3 and 4), seed dormancy (chr # 1, 2, 3 and 8), plant height (chr # 1 and 4), leaf length (chr # 5 and 10) and root number (chr # 3 and 5).

**Figure 7:**
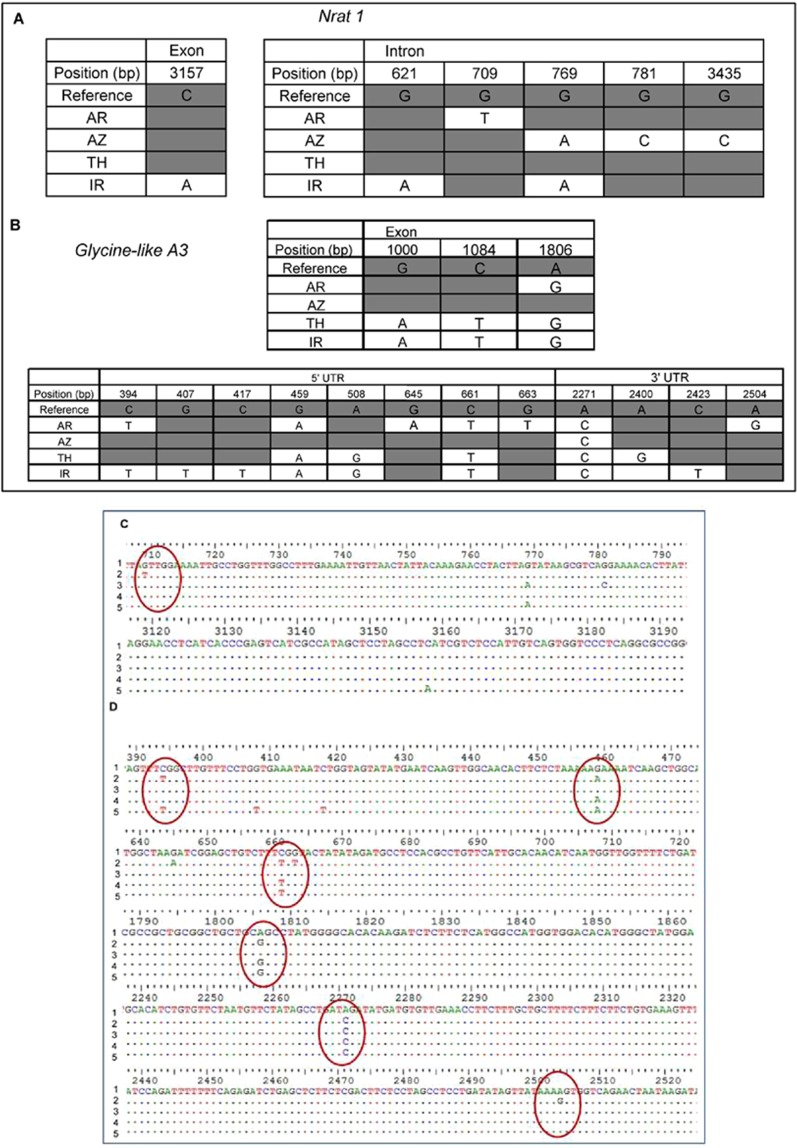
Single nucleotide polymorphism (SNP) and sequence alignment across *NRAT 1* (**A,C**) and *glycine-rich A3* (**B,D**) genes for five rice genotypes. Dark grey and white colours indicate reference and novel allele, respectively. Sequence alignment of *NRAT 1* (**C**) and *glycine-rich A3* for five rice genotypes where numbers 1, 2, 3, 4 and 5 indicate reference genotype, AR, AZ, TH and IR, respectively. The encircle portions indicate SNPs unique to AR.

Sequencing 3061 bp across *Nrat1* revealed a total of six nucleotide variations (Fig. 7A). Five nucleotide substitutions were observed in the intronic region. IR contains one SNP in the exonic region and two SNP in the intronic region. TH was found to carry the complete reference haplotype. Sequencing data showed that the exonic region of both azucena (AZ) and AR possess reference type allele but nucleotide variation in the intronic region was observed. AR has a SNP at position 729 in the genomic region while AZ has three nucleotide variations at positions 769, 781 and 3435. AZ had one SNP in common with IR at position 621 in the genomic region. One putative functional polymorphism specific to the Al sensitive genotype (IR) at position 3157 in the genomic region was identified. Change in the nucleotide level did not lead to any changes at the protein level in any of the genotypes. Though the SNP lie in the conserved region of the protein, it does not affect the conserved domain (Fig. 7c). All the tolerant and susceptible genotypes translate same protein for *Nrat1*. Four transversions and two transitions were observed for this gene. The nucleotide diversity was calculated for each genotype for genomic and coding sequence (CDS) regions (Supplemental Table S7). A genomic region of 2590 bp region covering 470 bp of the 5′ untranslated region (UTR), the whole CDS region and 574 bp of the 3′ UTR region was sequenced across 4 genotypes for *glycine-rich A3* (Fig. 7B). A total of fifteen nucleotide variations were observed. Eight nucleotide substitutions were observed in the 5′ UTR region while three nucleotide substitutions were observed in the exonic region and four in the 3′ UTR. AR contains one nucleotide substitution in the exonic region which is common between TH and IR. It also contains five nucleotide substitutions in the 5′ UTR and two in the 3′ UTR region. AZ possess a complete reference type haplotype except for one nucleotide variation observed in the 3′ UTR region at position 2271 bp (Fig. 7D). This nucleotide substitution in the 3′ UTR region was found to be common in all the four genotypes. Both IR and TH contain 3 common nucleotide substitutions in the exonic region as well as in the 5′ UTR region of the gene. At the protein level, three synonymous substitutions were observed. Nucleotide substitution unique to AR was observed at position 2504 bp in the 3′ UTR region. Similarly, SNP in the 3′ UTR region at position 2423 bp was observed only in IR. A total of twelve transitions and three transversions were observed in the genomic region. The nucleotide diversity was calculated for the genomic region and the CDS region for each genotype (Supplemental Table S7).

### Expression pattern of differentially regulated top 40 genes in three genotypes

A set of 40 transcripts highly expressed (log_2_ fold) in tolerant genotype AR were cross examined in the other two genotypes for expression levels (Table 5). Most of the DEGs were differentially regulated between the tolerant and susceptible genotypes. The highly expressed DEGs can be classified into; (a) signal transduction/regulatory components like transcripts encoding zinc finger protein (12g0581900) auxin induced protein (02g0445600), calcineurin binding protein (03g0685700); micro RNA gene (miR424a gene (AY730701.1), genes involved in chromatin modelling (02g02290) and RNA binding regulatory protein (12g01010); (b) cell wall associated transcripts like EP_003637074 and EXC01915 and cell wall hydrolase (AP014959). Hydrolase was significantly downregulated in AR (as compared with IR). (C) Genes for enzymes or accessory factors like peroxidase (11g0112200), isoflavone reductase (10g01044), peptidase (06g0625400), *etc*. are expressed in contrasting manner between AR, IR and TH. Transcripts encoding accessory factors for enzymes like LYR motif containing proteins (01g0342800) and UV repressible protein (02g0240100) are down regulated in AR but upregulated in TH and IR; (d) transcripts for components of translation machinery like 40S ribosomal protein S-15-like (03g0798600), 60S ribosomal protein L28-1 (05g0541900) and 18S rRNA sequence and (e) transcripts of unknown function (EPS70027, EXB92316, XP_007154367, 10g0102900, XP_006854095, 10g0154566, AP014960, 03g0160900, AK064232.1) were also differentially expressed.

**Table 5:** Top DEG summary details for genotype AR.

Sl. No.	Rice ID	log_2_ Fold Change [Treated/Control]			Putative function	Transcript_ID (AR genotype)	chr #
		AR	IR	TH			
1	Os03g0798600	7.32	17.44 (only T)	1.13	40S ribosomal protein S15-like	c3998	chr 3
2	Chloroplast gene	5.10	X	2.83 (only C)	ribulose bisphosphate carboxylase large chain	c579	
3	Os12g0581900	5.06	3.93	x	zinc finger protein 7	c52666	chr 12
4	Os02g0445600	4.86	X	x	auxin-induced protein 15 A	c88750	chr 2
5	Os03g0685700	4.76	2.49	2.91	calcineurin binding protein 1	c2515	chr 3
6	Chloroplast gene	4.65	−4.64	−0.76	cell wall-associated partial	c71	chl
7	Mitochondria gene	4.60	−1.34	−0.09	unknown	c19339	mito
8	No ID	4.54	X	0.1	unknown	c94	
9	CP012614.1	4.12	−0.8	0	cell wall-associated partial	c3912	
10	CP018158.1	4.02			unknown	c88896	
11	XR_003238916	3.95	−2.2	0.36	ncRNA	c67404	chr10
12	Os10g0102900	3.83	6.6	x	TMV resistance protein	c3260	chr 10
13	Os03g0685750	3.57	2.49	2.91	calcineurin binding protein 1	c89373	chr 3
14	Os11g0112200	3.40	X	x	cationic peroxidase 1	c89226	chr 11
15	No ID	3.22	30.73 (only T)	13.44 (only T)	cell wall-associated partial	c1701	
16	LOC102718242	3.21	X	x	Uncharacterized protein *Oryza bachyantha*	c7532	chr 2
17	AP014957	3.12	X	x	hypothetical protein	c130	
18	Os01g57968	−7.34	2.34	−0.67	expressed pro	c36168	chr 1
19	Os12g0100100	−5.37	3.42	1.04	RNA recognition motif containing protein; senescence related protein	c1640	chr 12
20	Os06g0136100	−5.26	−2.62	−4.34	unchr pro	c69	chr 6
21	Os01g0868000	−5.21	−9.54	1.5	ethylene-responsive transcription factor ABR1	c89973	chr 1
22	Os01g0342800	−5.02	2.23	1.92	LYR motif containing protein	c129	chr 1
23	Os02g0240100	−4.93	X	x	peroxidase 70	c23	chr 2
24	Os10g0154566	−4.76	X	x	unchr pro	c3235	chr 10
25	Os12g10710	−4.61	X	x	NB-ARC domain containing protein	c17211	chr 12
26	Os07g0673550	−4.39	2.43	1.58	putative UV-B repressible protein	c686	chr 7
27	AY730701.1	−4.12	X	x	miR424a gene	c35736	chr 9
28	XR_003238822.1	−3.93	X	x	18S rRNA sequence	c135	
29	Os02g02290	−3.75	2.52	−1.11	SNF2 family N-terminal domain containing protein	c61	chr 2
30	Os10g01044	−3.59	3.31	0.84	isoflavone reductase	c17392	chr 10
31	AP014960.1	−3.55	X	x	hypothetical protein	c1874	Chr 4
32	Os05g0541900	−3.51	X	0.46	60S ribosomal protein L28-1	c90489	chr 5
33	Os06g0625400	−3.50	−0.19	x	stromal processing peptidase	c17	chr 6
34	AP014959.1	−3.42	6.68	0.65	cell wall-associated hydrolase	c8108	chr 3
35	Os03g0160900	−3.28	−0.8	0	unchr pro	c88	chr 3
36	Os05g23180	−3.16	4.69	−0.22	transposon protein	c70	chr 5
37	CP012617.1	−3.03	X	x	unchr pro	c369	chr 9
38	Os03g0196500	−3.03	X	x	F-box SNE (OsFBX77)	c18795	chr 3
39	Os03g0278900	−3.03	X	x	ATP synthase chain 9-like protein	c156	chr 3
40	AK064232.1	−3.02	4.25	x	unchr pro	c7	chr 3

## Discussion

The major constrains to obtaining potential yields are various abiotic and biotic stresses encountered by plants. An estimated 39% and 52% of land suited to rice production in South and Southeast Asia, respectively is affected by severe soil problems^30^. These various soil problems are due to toxicity or deficiency of macro- and/or micro-nutrients. Therefore, a better understanding of molecular basis of uptake, transport, homeostasis and assimilation of these nutrients followed by application holds potential for gaining increases in crop productivity. In the case of acid soils, Al toxicity is the primary limitation to plant growth^31^. Avoidance and/or tolerance to Al is by exclusion and detoxification, respectively^31^. Till date various molecular and physiological strategies to combat Al toxicity from different higher plants have been reported like exclusion of Al^3+^ using malate transporter^4^, reactive oxygen species^32^, ART1^4^, modification of DNA^33^, etc. It is also known that most genes induced by Al are involved in various stress responses including phosphorus deficiency^34^. Rice is the most Al-tolerant of the cereal crop^35^ species. Inhibition of root growth, deposition of callose at the root apex along with cellular damage is the major Al toxicity symptom observed in plants^4,36^. Degree of toxicity varies depending on the genotype, Al concentration and duration of exposure^37^. Role of multiple mechanisms with underlying numerous genes has been suggested for high level of Al resistance in rice^6^. Therefore, it is important to understand Al toxicity response mechanism in diverse rice genotypes. Acidic soils adapted rice genotypes are therefore, one such genetically diverse pool, which can be targeted for better understanding of this complex mechanism. In our study, root transcriptome of diverse rice genotypes was targeted for better molecular understanding. The root growth inhibition in plants in Al^3+^ toxicity may not offer enough information about the level of tolerance^38^. It has also been reported that root biomass can be taken as an indirect trait for selection of tolerant genotypes. Similar visual toxic symptoms of Al^3+^ treated roots as observed by us have been reported earlier^39^. It has been proposed that cell wall of root cells is the site of both Al^3+^ toxicity and Al^3+^ exclusion. Most of the Al^3+^ absorbed by roots is localized to the apoplast^37^. Al^3+^ probably binds to the pectin matrix, replacing Ca^2+^ and makes the cell wall rigid, thereby affecting cell elongation^37^.

The data generated in the current study has revealed that none of DEGs identified from tolerant upland rice genotype overlap with the previous reported QTLs for Al toxicity tolerance^13^. However, TH DEGs overlap with QTLs Alt_TRG1.1_, Alt_PRG1.1_, Alt_PRG6.1_, Alt_LRG9.1_, Alt_TRG1.1_ and Alt_TRG12.1_. IR DEGs overlap with QTLs Alt_TRG1.1_, Alt_PRG1.1_ and Alt_PRG6.1_ with chromosome 1 showing maximum number of down-regulated genes in this susceptible genotype.

In the case of Al tolerance, using a set of 36,901 SNPs on a diverse set of 383 genotypes along with two immortal populations, three regions overlapping with genes *ART 1*, *STAR 2* and *NRAT 1* have been identified^13^. Further work on *NRAT 1*^35^ using 24 rice genotypes led to identification of alleles associated with Al tolerance. AR carries a distinct haplotype for *NRAT 1* (as well as glycine-rich A3), but the functional relevance, if any needs to be further investigated. ART1 is a transcription factor which is constitutively expressed and its expression is not affected by aluminum. It regulates a set of 31 genes in rice. We checked the status of these 31 genes in DEGs of the three genotypes. It was found that the DEGs found in the three genotypes are not regulated by ART1. It has been shown for an Al-tolerant cultivar Koshihikari, that other mechanisms apart from ART1-regulated mechanism do exist^40^. Root proteome studies on Al tolerant rice has revealed modulating the available energy may be one way of combating Al toxicity^41^. In high Al accumulating species like buckwheat, it has been shown that most of the ART1-regulated gene homologs were not differentially regulated in response to high Al levels^42^. ART2, which is a homolog of ART1 has also been reported to regulate Al toxicity tolerance albeit with a different set of genes^43^. These genes were also not differentially regulated in AR genotype. An ABC transporter was significantly upregulated in IR. Previously, ABC transporters located on tonoplast have been implicated in Al detoxification^20^. It would be of interest to check the role of this ABC transporter in Al toxicity response.

One of the transcripts that were highly upregulated in response to Al in AR encodes S15- like ribosomal protein. Members of ribosomal S15 proteins in *Arabidopsis* viz. *RPS15aA*, *-D* and *-F* are expressed abundantly under temperature and metal stress^44^. It has been suggested that stress may result in altering composition of ribosomes and biased translation^45^. Another transcript that was differentially expressed codes for large subunit of ribulose bisphosphate. It is reported that stresses like salt^46^, drought, ozone, high temperature and cadmium^47^ lead to accumulation of large chain of ribulose bisphosphate in rice and *Arabidopsis* cells, respectively. This is a major leaf protein involved in photosynthesis and photorespiration.

**Figure 8:**
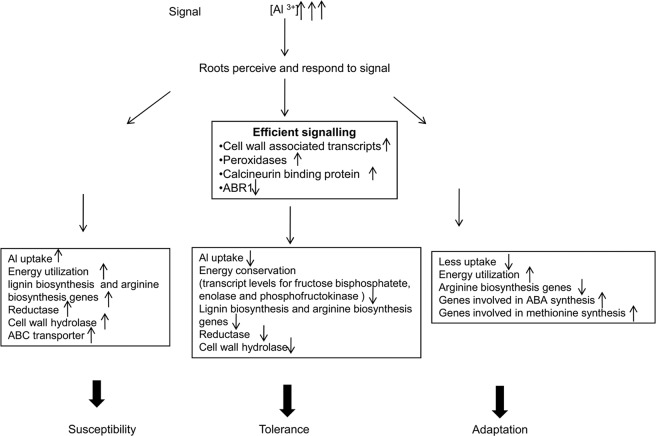
Schematic representation of the probable mechanism of susceptibility, tolerance and adaptation based on Al root transcriptome data for genotypes IR, AR and TH, respectively ↑–upregulation and ↓– downregulation.

Increased and better signal transduction in response to Al stress can be one of the strategies of tolerance (Fig. 8). The various components of signal transduction and regulatory machinery like zinc finger protein, calcieurin binding protein, cell wall associated transcripts are among the highly upregulated DEGs in tolerant genotype AR. It has been reported that cell wall associated kinases play a vital role in signaling mechanisms in response to heavy metal toxicity^48^. It is to be seen whether the cell wall associated proteins coded by transcripts upregulated in AR have kinase activity. AR DEGs reflect a better and efficient response to Al stress (as indicated by the GO terms). Also, based on pathway analysis of functionally annotated genes, it appears that AR conserves energy by downregulating key genes (fructose bisphosphate, enolase and phosphofructokinase) of glycolysis pathway and maintaining transcript levels of two key exergonic step metabolizing genes (citrate synthase and phosglycerate kinase) under Al stress (Fig. 4A). Thickening of rice roots due to callose deposition is an energy dependent process wherein sugar is diverted for deposition along cell walls^6,36,37^. Previously, it has been reported that metal stress leads to significant alterations in proteins involved in energy metabolism^41,49^. The number of downregulated transcripts is much higher in Al toxicity conditions. Another transcript which was significantly different between tolerant AR and susceptible IR encodes LYR motif containing protein. These motifs are characteristic of Fe-S proteins, which in turn are involved in electron transport, photosynthesis and DNA repair. Previously, it has been shown that rice genotypes carrying SUB1A gene survive flooding stress by suppressing shoot elongation and conserving energy^50^. This is achieved by higher level induction of genes, Slender Rice-1 (SLR1) and SLR Like-1 (SLRL1), involved in ethylene signalling pathway^50^.

Our work provides insight into a novel mechanism of Al toxicity tolerance in *indica* type rice, AR. The comparison of Al^3+^ accumulation, suggests that susceptible genotype IR accumulates significant amounts of Al in its roots whereas AR and TH do not. Transcriptome data generated on roots identified a total of 511, 804 and 912 DEGs in genotypes AR, IR and TH, respectively. The tolerance in AR appears to be as a result of novel mechanism as none of the reported Al toxicity genes or QTLs overlap with significant DEGs. Transcriptome reveals aluminum toxicity tolerant rice adapts to high aluminum through expression of genes involved in cell signaling and energy conservation whereas susceptible genotype responds though energy intensive processes. Sequencing of *NRAT1* and glycine-rich protein A3 revealed distinct haplotype for *indica* type AR. The newly identified components of Al tolerance will be helpful in development of molecular breeding strategies for development of Al toxicity tolerant rice varieties.

## Materials and method

### Plant material and Al toxicity treatment

Three rice genotypes (ARR 09 (AR); IR1552 (IR), and theruvii (TH)) were selected for generating NGS data for seedling stage Al toxicity tolerance. Seeds (30–40) of the selected genotypes were germinated on petri plates containing moist filter paper and kept in the green house. The 5-day old seedlings of each genotype were transferred to full strength Magnavaca’s solution containing 0.54 mM AlCl_3_ to create Al toxic hydroponic condition^51^. A separate set of seedlings was grown as control in Magvaca’s solution without AlCl_3_. After 5 days of treatment, phenotypic traits like fresh root weight and shoot weight were measured. The samples were divided in half: one half was cryopreserved for RNA extraction and transcriptome analysis. The other half was dried; dry weight measured and estimation of total Al carried out using Atomic Absorption Spectrometer (Perkin Elmer Analyst 200). Three independent replicates were used per genotype.

### Hematoxylin staining

The roots of 10-day old seedlings grown in presence or absence of Al, were used for hematoxylin staining [0.2% hematoxylin (Himedia) and 0.02% potassium iodide, w/v] following standard procedure^52^. Free hand cross sections for the root were made and studied under the microscope (Leica DM 750) at 10X and 40X.

### RNA extraction and generation of raw reads

All the six samples were sent to the commercial service provider, Xcelris Genomics Ltd, India for sequencing. The extraction of total RNA, RNA quality and quantity analysis, library generation and identification of quality reads was performed following previously reported methodology^53^. The mapping of the reads was done with spliced read mapper TopHat version 1.4.1 using Nipponbare (MSU release 6.1) genome as reference. The collection of mapped paired end reads and read counts were done using Picard 1.63 and HTSeq version 0.5.3, respectively^53^ (Supplemental Fig. S1).

### Quality check, data assembly and transcriptome generation

The assembly of QC passed sequence reads was performed to identify putative transcripts that were detected in each pair of rice genotypes (control and treatment) using two MIRA and TRINITY assemblers independently. The transcripts were divided into various sizes to find heterogeneity and degree of fragmentation. Typically, transcripts >100 bp in length were considered for downstream analysis (for ion torrent data).

### Differential gene expression and annotation of transcriptome

The differential analysis was done using DESeq v 2.0^54^ and annotation using Blast2go^55^ for all the six samples. Nonredundant protein database of National Center for Biotechnology Information (NCBI) was used for BLASTx^56^ (E value ≤0.00001; annotation cutoff = 55; GO weight = 5). The transcriptome for the three genotypes was analyzed for a) DEGs and b) transcripts which were specific to the Al concentration (treatment (0.54 mM Al^3+^)).

### Confirmation of random DEGs by qRT-PCR

To validate the results from the transcriptome experiment, 15 randomly selected DEGs were analyzed using qRT-PCR. Total RNA was extracted from roots using Spectrum plant total RNA kit (SIGMA) according to the manufacturer’s protocol. The first strand cDNA synthesis was carried out and rice ubiquitin gene^54^ was used as reference gene for normalization as previously described^57^. Three replicates were performed.

### Gene ontology, functional annotation and identification of pathways enriched in Al toxicity

The statistically significant genes were identified and classified using standard methods as previously described^53^. For functional annotation of the genes, all the pathway related genes sequence involved in rice were downloaded and blast of all pathway genes (as database) and transcripts (as query) from all samples was done. Mapped LOC ID (Pathway involved gene id) was converted into RAP ID and each RAP ID (Pathway involved gene id) was mapped to our transcript expression data and linked with pathway name. This data was used as input in programme cytoscape for image generation (≥2 (upregulate, red colour, ≤ −2 downregulated, green colour).

### Mapping of known/reported loci/QTLs

Sequences for all genomic positions were extracted and BLAST on up- and down-regulated transcript from all samples (as query) against genomic sequence (as database) was performed.

### Sequencing across reported genes for Al toxicity tolerance

Sequencing across the two reported genes, *NRAT 1* and glycine-rich protein A3^13^ was done following standard methods^3^. All the four genotypes (including Azucena (AZ), an already reported tolerant *aus* genotype was included for sequencing)^13^ produced single amplicon with the gene-specific primers using high fidelity Taq polymerase (Sigma-Aldrich, USA) when analyzed by agarose gel electrophoresis. The PCR products were sent for sequencing using forward and reverse primers to Amnion Biosciences Pvt. Ltd, India. Sequences obtained were aligned using BioEdit 7.2.5. *Nipponbare* was used as a reference genome for sequence alignment, putative SNPs identified based on sequence homology and nucleotide diversity (π) calculated.

## Supplementary information

Supplementary Information.

## Data Availability

The RNAseq data reported are available in NCBI sequence read archive under the accession number SUB986310 (PRJNA287493). The data sets supporting the results of this article are included within the article.
