# Supplementary material for: Root transcriptome reveals efficient cell signaling and energy conservation key to aluminum toxicity tolerance in acidic soil adapted rice genotype
Source: Sci Rep. 2020 Mar 12;10:4580. doi: 10.1038/s41598-020-61305-7 (PMC7067865; doi:10.1038/s41598-020-61305-7)
Supplement: Supplementary file 1 — Supplementary Information. [file 41598_2020_61305_MOESM1_ESM.pdf]

Root transcriptome reveals efficient cell signaling and energy conservation key to aluminum toxicity tolerance in acidic soil adapted rice genotype

Wricha Tyagi[1], Julia S. Yumnam[1], Devyani Sen[1], Mayank Rai*[1]

[1] School of Crop Improvement, College of Post-Graduate Studies, Central Agricultural University (Imphal), Umroi Road, Umiam, Meghalaya, India-793103

* **Corresponding author**: Mayank Rai

Supplemental Information

Supplementary Fig. S1 Details of bioinformatics pipeline and commands used for analyses

Supplemenary Table S1 Annotation report for AR

Supplemenary Table S2 Annotation report for IR

Supplemenary Table S3 Annotation report for TH

Supplemenary Table S4 List of *a priori* rice genes

Supplemenary Table S5 Details of mapped QTLs

Supplemenary Table S6 List of Al toxicity QTLs

Supplementary Table S7 Nucleotide diversity (π) estimates for genes *NRAT 1* and *glycine-like protein A3*

Supplemenary Dataset S1 Expression analyses report for AR

Supplemenary Dataset S2 Expression analyses report for IR

Supplemenary Dataset S3 Expression analyses report for TH

Supplementary Fig. S1 Details of bioinformatics pipeline and commands used for analyses

RNAseqDenovo Pipeline (Ion Torrent Data)

Raw Data

↓

QC (Prinseq)

↓

Assembly (Tool: mira)

↓

Assembly validation (Tool: bowtie2)

↓

Assembly filter (Transcript length >=100)

↓

CD-Hit clustering (cd-hit-est)

↓

Blast with NRDB and Gene ontology Analysis (Tool: blast2gO)

↓

Abudancecalculation (trinityrnaseq-align_and_estimate_abundance.pl)

↓

Deseq Differential calculation

Commands:

- **Prinseq (QC):**

```
perl /home/user/NGS/Programs/prinseq-lite-0.20.4/prinseq-lite.pl \
-fastqinput_file \
-out_format 3 \
-graph_data \
-log \
-min_len 15 \
-min_qual_mean 20
```

- **Mira Assembler:**

```
command
project = my_assembly_11_12
job = est,denovo,accurate
parameters = -GE:not=24

# The second part defines the sequencing data MIRA should load and assemble
# The data is logically divided into "readgroups": this reflects the
# ... that read sequences ...

readgroup = iontorrent_assembly
data = Sample_input
technology = iontor
```

- **Assembly validation**

```
bowtie2-build -f 11_AC_AT_Good_assembly_out.unpadded.fasta 11_AC_AT_Good_assembly
```

```
bowtie2 -p 7 -x 11_AC_AT_Good_assembly–UInput.fastq -S 11_12_good_assembly_map.sam
```

- **CD_hit clustering**

```
cd-hit-est \
-d 0 \
-c 0.8 \
-A 0.7 \
-l 100 \
-iInput_file\
-o Out_file \
-T 7\
-M 0
```

- **AbudanceCalulation**

```
perl /home/bionivid/Programs/trinityrnaseq-2.0.6/util/align_and_estimate_abundance.pl --transcripts 15_16_AC_AT_good_out.unpadded.fasta \
--seqTypefq \
--single 15_16_AC_AT_good.fastq \
--est_method RSEM \
--aln_method bowtie \
--prep_reference \
--coordsort_bam \
--thread_count 4 \
--output_dir 15_16_AC_AT_Good
```

Supplementary Table S1

Transcript_ID	Sequence length	NCBI nrdb Hit Acc	E-Value	GO	Similarity	Bit Score	Alignment length
ARC_ART_c1	872	XP_003637074	0.0	-	60	40.817	35
ARC_ART_c2	1263	AGV54820	0.0	GO:0009536	69	105.145	93
ARC_ART_c3	532	KJB76688	0.0	-	59	70.4774	84
ARC_ART_c6	438	EPS74345	0.0	-	58	71.633	93
ARC_ART_c7	465	EPS74494	0.0	GO:0005840	81	103.605	70
ARC_ART_c8	1179	CDY45505	0.0	-	64	53.1434	53
ARC_ART_c10	1025	EPS74505	0.0	GO:0009507	60	137.502	171
ARC_ART_c11	493	ERN16843	0.0	GO:0009536	63	64.6994	68
ARC_ART_c12	821	XP_003637074	0.0	GO:0008152	51	71.633	134
ARC_ART_c14	329	AFK35083	0.0	-	64	51.9878	57
ARC_ART_c15	307	AFK35083	0.0	GO:0005739	81	54.6842	38
ARC_ART_c17	407	CAN62678	0.0	GO:0009536	62	113.62	104
ARC_ART_c18	703	AGZ19352	0.0	GO:0009536	75	63.1586	52
ARC_ART_c19	475	AGV54820	0.0	GO:0008152	54	30.0314	37
ARC_ART_c22	725	AGV54820	0.0	GO:0008152	68	133.65	147
ARC_ART_c23	264	EMT21312	0.5	-	53	34.2686	56
ARC_ART_c24	537	XP_009388289	0.0	GO:0006810	78	52.373	37
ARC_ART_c25	374	XP_003588337	0.0	-	69	54.299	46
ARC_ART_c27	505	CDY45505	0.0	-	57	60.8474	80
ARC_ART_c29	291	XP_003599574	0.0	-	61	37.3502	52
ARC_ART_c31	493	EPS74494	0.0	GO:0009507	75	75.0998	70
ARC_ART_c32	518	XP_003588326	0.0	-	78	56.225	42
ARC_ART_c34	634	BAJ11784	0.0	-	91	118.242	62
ARC_ART_c35	267	EPS70027	0.0	-	47	45.0542	67
ARC_ART_c38	299	AGC78943	0.0	-	77	49.2914	36
ARC_ART_c39	822	XP_003616487	0.0	GO:0009536	53	59.3066	98
ARC_ART_c42	587	XP_003637074	0.0	GO:0008152	47	67.0106	171
ARC_ART_c43	668	EPS74505	0.0	GO:0009507	59	108.612	131
ARC_ART_c44	330	YP_001312258	0.0	-	64	63.5438	68
ARC_ART_c45	318	CDY63595	0.0	-	51	47.7506	77

ARC_ART_c46	280	EYU24190	0.0	-	65	79.7221	78
ARC_ART_c50	229	XP_011099030	0.5	-	56	33.8834	51
ARC_ART_c52	161	KFK40798	0.3	-	58	34.2686	51
ARC_ART_c54	472	ACU24411	0.0	-	73	51.6026	41
ARC_ART_c59	324	KGN55456	0.1	-	54	35.4242	46
ARC_ART_c61	309	XP_003610227	0.1	-	60	37.7354	51
ARC_ART_c65	468	AGC78943	0.0	-	78	105.531	74
ARC_ART_c66	961	EXC34899	0.0	GO:0008152	51	87.8113	152
ARC_ART_c69	190	KEH22088	0.0	-	75	46.2098	58
ARC_ART_c70	441	CDY63598	0.0	-	70	83.1889	64
ARC_ART_c71	299	XP_003637074	0.0	-	55	45.8246	72
ARC_ART_c72	212	EXC01914	0.0	GO:0009536	75	53.1434	37
ARC_ART_c73	472	YP_001152214	0.0	-	53	48.9062	79
ARC_ART_c74	466	BAJ11784	0.0	-	52	57.3806	93
ARC_ART_c79	243	KEH17025	0.0	-	51	42.743	70
ARC_ART_c80	253	CDY63598	0.0	-	72	66.6254	48
ARC_ART_c83	417	AGC78890	0.0	-	65	85.5001	93
ARC_ART_c86	214	AFK35083	0.0	-	68	48.9062	47
ARC_ART_c87	255	AAU90319	0.0	GO:0009536	73	82.0333	60
ARC_ART_c88	176	EPS70023	0.0	-	51	35.039	41
ARC_ART_c92	416	EPS74494	0.0	GO:0009536	68	88.9669	76
ARC_ART_c94	200	EXB92316	0.0	-	70	48.9062	37
ARC_ART_c96	313	YP_001152214	0.0	-	58	40.0466	43
ARC_ART_c104	313	KIZ06744	1.0	-	49	33.8834	55
ARC_ART_c105	450	XP_003616487	0.0	GO:0008152	86	70.0922	37
ARC_ART_c108	274	KJB44141	0.0	-	68	45.4394	38
ARC_ART_c109	188	XP_007154367	0.2	-	62	32.7278	35
ARC_ART_c111	1072	ERN10593	0.0	-	60	57.7658	64
ARC_ART_c113	398	CDY19671	0.0	-	54	63.929	81
ARC_ART_c115	182	BAJ11784	0.1	-	67	36.1946	34
ARC_ART_c116	531	CDY19671	0.0	GO:0009507	76	79.7221	52
ARC_ART_c123	263	CDY19671	0.0	-	52	48.9062	69
ARC_ART_c124	418	ABH09321	0.0	-	52	59.3066	107
ARC_ART_c126	181	EPS74531	0.0	-	67	39.6614	37

ARC_ART_c127	363	AGV54820	0.1	-	58	31.5722	41
ARC_ART_c128	242	XP_008447683	0.1	-	56	35.8094	39
ARC_ART_c129	383	EPS74511	0.0	-	73	86.2705	69
ARC_ART_c130	413	XP_003599574	0.1	-	52	35.8094	61
ARC_ART_c135	479	XP_001419182	0.0	-	66	58.151	59
ARC_ART_c136	281	XP_006364867	0.0	-	56	39.2762	44
ARC_ART_c137	851	ACU24256	0.0	-	76	43.1282	34
ARC_ART_c140	175	KFM26658	0.0	GO:0055114	79	70.0922	53
ARC_ART_c141	281	ACJ83969	0.1	-	62	35.039	43
ARC_ART_c142	161	XP_006363639	0.0	-	67	40.817	43
ARC_ART_c143	277	ABH09321	0.0	-	61	47.3654	49
ARC_ART_c145	211	XP_009350805	0.0	-	71	43.1282	38
ARC_ART_c149	617	KGN53990	0.0	-	57	32.7278	35
ARC_ART_c150	171	EMS49165	0.0	-	71	43.8986	38
ARC_ART_c153	323	NP_817264	0.0	GO:0009536	72	47.7506	37
ARC_ART_c156	375	NP_001169136	0.0	GO:0004497	56	48.521	64
ARC_ART_c158	277	EXC10888	0.0	-	72	43.8986	36
ARC_ART_c159	217	XP_011080634	0.9	-	64	33.113	37
ARC_ART_c168	156	NP_569677	0.8	-	55	30.8018	45
ARC_ART_c170	114	KEH16991	0.0	-	66	41.2022	39
ARC_ART_c171	373	XP_010239583	0.0	GO:0016021	96	227.254	123
ARC_ART_c172	366	XP_003638717	0.0	-	70	46.2098	34
ARC_ART_c174	209	XP_003627732	0.0	GO:0070330	78	99.3673	69
ARC_ART_c177	304	XP_002981727	0.2	-	61	35.8094	49
ARC_ART_c178	109	XP_006399187	0.2	-	61	33.8834	34
ARC_ART_c179	228	XP_001786759	0.0	-	66	56.9954	53
ARC_ART_c180	150	XP_002536832	0.0	GO:0055114	93	82.0333	49
ARC_ART_c187	172	KDP40381	0.8	-	57	33.113	49
ARC_ART_c188	170	XP_009350810	0.0	GO:0044763	83	85.8853	56
ARC_ART_c192	450	ABH09321	0.0	-	56	61.2326	110
ARC_ART_c193	148	XP_002540107	0.0	-	68	53.5286	47
ARC_ART_c195	110	EEC78702	0.0	GO:0044699	72	46.595	36

ARC_ART_c203	151	AAT46463	0.0	GO:0003333	88	35.4242	34
ARC_ART_c204	159	XP_009351044	0.0	-	67	53.9138	52
ARC_ART_c210	125	XP_005651524	0.0	-	60	43.5134	40
ARC_ART_c211	205	XP_002503512	0.0	-	53	37.3502	62
ARC_ART_c214	185	XP_006481707	0.1	-	57	35.8094	40
ARC_ART_c215	281	YP_009057850	0.0	GO:0006412	73	49.6766	42
ARC_ART_c216	125	XP_002950342	0.0	-	60	39.6614	38
ARC_ART_c222	211	AGZ19352	0.0	GO:0009536	74	47.7506	39
ARC_ART_c224	358	BAJ92142	0.5	-	41	35.039	65
ARC_ART_c229	196	XP_010465922	0.2	-	46	35.039	52
ARC_ART_c230	255	AGC78943	0.0	-	72	43.1282	37
ARC_ART_c235	267	ABO20848	0.0	-	47	47.3654	92
ARC_ART_c245	246	XP_008664051	1.0	-	43	32.7278	46
ARC_ART_c256	234	XP_001689652	0.9	-	53	32.7278	56
ARC_ART_c259	143	ABF81459	0.8	-	53	33.113	39
ARC_ART_c262	363	XP_002489102	0.0	-	57	50.0618	57
ARC_ART_c263	283	XP_002948757	0.0	-	60	37.3502	40
ARC_ART_c265	206	BAB33421	0.0	-	66	42.3578	39
ARC_ART_c266	161	XP_001697320	0.0	GO:0005786	85	68.1662	47
ARC_ART_c269	153	EAY94034	0.0	GO:0046872	85	75.8702	40
ARC_ART_c278	229	ERN10593	0.0	-	62	40.0466	35
ARC_ART_c285	260	XP_001701638	0.0	GO:0044763	67	81.2629	83
ARC_ART_c287	381	CDY45505	0.2	-	62	36.5798	40
ARC_ART_c290	206	XP_005648729	0.0	GO:0000166	88	119.398	68
ARC_ART_c295	345	KGN47269	0.0	-	63	45.0542	36
ARC_ART_c297	127	KJB31094	0.0	-	64	34.6538	34
ARC_ART_c298	232	XP_008455391	0.6	-	55	33.4982	45
ARC_ART_c299	241	ABH09321	0.5	-	60	33.8834	38
ARC_ART_c300	141	XP_011015255	0.0	-	70	47.7506	44

ARC_ART_c305	268	XP_009589852	0.0	-	75	60.4622	44
ARC_ART_c309	266	CCO66143	0.6	-	55	33.8834	45
ARC_ART_c318	110	XP_002973663	0.5	-	70	32.7278	34
ARC_ART_c319	147	XP_002540075	0.0	-	67	37.3502	37
ARC_ART_c324	306	BAD46202	0.1	-	59	35.8094	66
ARC_ART_c325	153	EMT00481	0.0	GO:0001510	82	56.6102	41
ARC_ART_c329	113	XP_002536079	0.0	-	66	41.2022	36
ARC_ART_c346	272	KEH15469	0.1	-	53	36.5798	88
ARC_ART_c356	188	XP_001787011	0.0	-	56	40.4318	60
ARC_ART_c358	343	XP_003610227	0.0	-	76	44.669	34
ARC_ART_c360	294	XP_008650700	0.7	-	50	34.2686	52
ARC_ART_c364	174	EPS70027	0.0	-	57	41.5874	57
ARC_ART_c367	271	XP_007023952	0.6	-	54	33.113	35
ARC_ART_c368	138	XP_009420080	0.0	-	60	38.5058	40
ARC_ART_c369	374	XP_003056974	0.1	-	55	35.039	40
ARC_ART_c370	300	XP_002488947	0.0	-	74	81.6481	63
ARC_ART_c374	228	YP_009019359	0.0	GO:0005840	77	66.2402	49
ARC_ART_c376	164	XP_002540328	0.0	-	60	46.595	51
ARC_ART_c378	252	XP_002538804	0.0	-	59	46.595	47
ARC_ART_c382	155	XP_002539938	0.0	GO:0098655	75	52.7582	48
ARC_ART_c392	209	XP_009350056	0.0	GO:0007127	84	75.0998	52
ARC_ART_c394	183	EAY93131	0.0	GO:0009536	90	80.8777	53
ARC_ART_c395	139	XP_011016264	0.0	GO:0016020	84	67.3958	45
ARC_ART_c396	165	YP_001312258	0.0	-	67	39.6614	40
ARC_ART_c401	253	KEH17348	0.0	-	69	43.5134	36
ARC_ART_c403	277	XP_003622234	0.0	-	92	84.7297	42

ARC_ART_c404	253	XP_002538550	0.0	-	94	120.939	69
ARC_ART_c410	194	BAD01716	0.0	-	55	37.3502	54
ARC_ART_c413	135	XP_002536203	0.0	-	81	51.6026	37
ARC_ART_c420	208	XP_003627732	0.0	-	100	90.1225	42
ARC_ART_c423	144	XP_009760221	0.0	GO:0016020	76	57.7658	50
ARC_ART_c424	107	XP_003057272	0.0	GO:0004871	74	44.2838	35
ARC_ART_c428	514	XP_003627732	0.0	-	66	41.9726	42
ARC_ART_c430	208	EXC01915	0.0	-	58	44.669	50
ARC_ART_c431	149	CAN73362	0.7	-	50	32.7278	40
ARC_ART_c436	110	XP_003520794	1.0	-	65	31.9574	35
ARC_ART_c438	124	XP_010438362	0.4	-	63	33.4982	38
ARC_ART_c440	178	XP_009350816	0.0	-	67	51.6026	43
ARC_ART_c441	350	XP_009762773	0.4	-	53	34.2686	41
ARC_ART_c444	175	XP_003637074	0.3	-	63	34.2686	36
ARC_ART_c452	201	XP_002534724	0.0	GO:0016874	67	47.7506	43
ARC_ART_c455	112	XP_011040809	0.7	-	55	32.7278	36
ARC_ART_c458	153	XP_002538398	0.1	-	60	36.1946	41
ARC_ART_c467	204	XP_006448110	0.2	-	60	35.8094	50
ARC_ART_c469	200	XP_002534945	0.0	GO:0010581	91	77.411	45
ARC_ART_c470	171	XP_005648749	0.1	-	63	34.6538	38
ARC_ART_c474	169	XP_008794883	0.0	GO:0033587	76	48.1358	34
ARC_ART_c478	150	KDD76762	0.0	-	69	37.7354	36
ARC_ART_c480	272	XP_001695777	0.1	-	64	36.5798	37
ARC_ART_c482	580	YP_001152215	0.0	-	65	50.447	41
ARC_ART_c485	362	EEC81324	0.0	-	58	77.0258	84
ARC_ART_c487	166	KEH15665	0.0	GO:0005488	77	52.7582	48
ARC_ART_c490	454	EPS74533	0.0	-	63	54.6842	49
ARC_ART_c492	475	AGC78943	0.0	-	69	53.9138	46
ARC_ART_c493	186	EYU24190	0.0	-	63	40.0466	41
ARC_ART_c497	248	KEH39580	0.8	-	51	33.4982	43

ARC_ART_c503	121	XP_002539099	0.0	GO:0005737	80	52.373	35
ARC_ART_c505	128	XP_002539818	0.0	-	73	41.5874	34
ARC_ART_c506	174	EDQ48307	0.0	GO:0044238	63	62.003	55
ARC_ART_c510	173	XP_002539337	0.0	-	68	53.9138	51
ARC_ART_c511	142	BAK02089	0.2	-	70	33.8834	40
ARC_ART_c519	146	XP_010314844	0.0	-	52	39.2762	48
ARC_ART_c520	154	EEC76122	0.0	GO:0044763	72	60.8474	50
ARC_ART_c522	189	EPS74505	0.0	GO:0005739	85	76.2554	47
ARC_ART_c523	165	XP_011070787	0.0	GO:0044238	58	42.743	51
ARC_ART_c525	375	ACU24411	0.2	-	51	34.2686	60
ARC_ART_c528	356	KJB44141	0.0	GO:0009507	79	62.3882	43
ARC_ART_c529	150	EAZ04247	0.6	-	55	33.113	34
ARC_ART_c531	152	XP_010914633	0.0	GO:0070011	70	51.6026	50
ARC_ART_c533	177	EAZ40562	0.1	-	59	36.1946	44
ARC_ART_c546	379	XP_008348155	0.0	-	54	39.2762	51
ARC_ART_c553	283	EEC76878	0.0	GO:0015986	84	64.3142	39
ARC_ART_c556	230	XP_003079855	0.3	-	52	34.6538	48
ARC_ART_c568	149	XP_011016696	0.0	GO:0035434	93	85.1149	47
ARC_ART_c571	207	EEE67871	0.0	GO:0055085	98	82.0333	55
ARC_ART_c574	136	XP_001787001	0.1	-	58	35.039	34
ARC_ART_c575	132	XP_001755239	0.3	-	62	33.8834	37
ARC_ART_c579	237	EXC35991	0.0	-	65	50.8322	44
ARC_ART_c580	336	XP_003614387	0.0	-	64	95.1301	98
ARC_ART_c581	127	BAN92365	0.3	-	52	33.8834	40
ARC_ART_c583	307	ABF94688	0.3	-	44	33.8834	105
ARC_ART_c589	315	KIY97037	0.0	-	49	38.5058	51
ARC_ART_c595	268	XP_001701046	0.0	-	65	42.743	35
ARC_ART_c600	167	XP_002949021	0.0	GO:0008236	60	51.9878	55
ARC_ART_c603	141	EXB42927	0.0	-	61	40.4318	36

ARC_ART_c604	160	XP_009393956	0.5	-	59	33.4982	49
ARC_ART_c605	328	KDP20462	0.0	-	58	42.743	43
ARC_ART_c607	178	EEC76877	0.0	GO:0046961	94	108.612	59
ARC_ART_c609	238	XP_010512881	0.5	-	48	33.8834	41
ARC_ART_c619	140	XP_002539886	0.0	-	67	51.2174	46
ARC_ART_c621	286	XP_010247505	0.7	-	47	33.8834	69
ARC_ART_c622	160	XP_008244970	0.3	-	50	32.3426	46
ARC_ART_c641	263	XP_002535044	0.0	GO:0004489	79	128.642	87
ARC_ART_c645	172	CEF96798	0.0	GO:0098655	76	51.2174	34
ARC_ART_c652	126	EEC75945	0.0	-	58	41.5874	41
ARC_ART_c658	159	XP_002960336	0.0	-	68	56.9954	54
ARC_ART_c666	255	KDP20462	0.0	-	66	70.0922	63
ARC_ART_c668	226	XP_003539573	1.0	-	48	33.113	70
ARC_ART_c672	120	CEF98618	0.5	-	53	33.113	39
ARC_ART_c675	122	XP_002539686	0.0	GO:0004673	78	48.1358	41
ARC_ART_c680	414	AGV54820	0.0	-	60	63.929	68
ARC_ART_c684	205	EPS70027	0.0	GO:0005739	74	61.2326	47
ARC_ART_c685	162	XP_005644356	0.0	-	56	41.9726	50
ARC_ART_c686	519	XP_007161040	0.0	-	70	56.225	44
ARC_ART_c690	242	ERN12145	0.7	-	58	32.3426	36
ARC_ART_c693	164	P53385	0.0	GO:0019557	82	63.929	41
ARC_ART_c695	292	CDX86307	0.2	-	54	35.8094	53
ARC_ART_c696	192	EEC68416	0.0	GO:0010048	72	60.8474	47
ARC_ART_c697	155	BAE71214	0.0	GO:0006527	85	65.0846	35
ARC_ART_c699	173	KFM23486	0.1	-	48	35.8094	39
ARC_ART_c701	153	XP_002959991	0.0	-	62	41.5874	43
ARC_ART_c703	138	XP_009364509	0.0	-	57	39.2762	38

ARC_ART_c704	253	KFM22554	0.0	-	53	38.891	56
ARC_ART_c706	231	CDM82334	0.0	GO:0009507	82	67.3958	39
ARC_ART_c707	292	XP_002445044	0.9	-	38	33.4982	78
ARC_ART_c716	225	XP_011016759	0.0	-	76	68.5514	60
ARC_ART_c718	208	ABR17255	0.5	-	57	32.3426	38
ARC_ART_c721	151	XP_010926696	0.5	-	52	33.4982	50
ARC_ART_c723	154	BAK00181	0.1	-	53	34.6538	52
ARC_ART_c726	147	XP_002535793	0.0	GO:0015031	80	69.3218	46
ARC_ART_c727	125	EEC76917	0.0	GO:0003677	90	69.707	41
ARC_ART_c730	140	EEC78702	0.0	GO:0016020	91	85.8853	46
ARC_ART_c731	223	XP_002465525	0.8	-	47	33.4982	57
ARC_ART_c738	159	XP_002538238	0.0	-	64	40.0466	34
ARC_ART_c741	193	XP_001701050	0.0	-	61	33.113	34
ARC_ART_c742	115	CAG30723	0.0	-	64	37.7354	34
ARC_ART_c743	171	XP_002538039	0.0	-	75	79.337	57
ARC_ART_c744	109	AHH02784	0.1	-	61	34.6538	34
ARC_ART_c747	395	AAV44205	0.0	-	59	67.0106	92
ARC_ART_c749	162	XP_002538951	0.0	-	73	68.5514	53
ARC_ART_c751	110	AEQ20864	0.0	GO:0043167	77	51.9878	35
ARC_ART_c758	110	XP_003061391	0.0	-	61	40.4318	39
ARC_ART_c761	111	EPS72369	0.0	-	63	37.3502	36
ARC_ART_c765	168	XP_005845301	0.0	-	60	50.8322	51
ARC_ART_c767	278	XP_010059781	0.6	-	47	34.2686	80
ARC_ART_c771	177	XP_002536999	0.0	GO:0050794	75	54.299	48
ARC_ART_c772	122	XP_002540134	0.0	-	69	43.5134	36

ARC_ART_c774	294	EEC76122	0.0	GO:0004149	84	97.0561	64
ARC_ART_c779	155	XP_002317132	0.8	-	63	32.7278	41
ARC_ART_c781	168	XP_003057890	0.0	GO:0009941	80	80.8777	52
ARC_ART_c783	119	XP_002539243	0.0	-	74	43.5134	35
ARC_ART_c788	195	EPS65861	0.7	-	55	33.113	56
ARC_ART_c792	161	XP_006594354	0.3	-	53	34.2686	45
ARC_ART_c796	278	XP_002534963	0.0	-	55	63.1586	87
ARC_ART_c797	251	XP_002536196	0.0	GO:0016787	69	51.2174	39
ARC_ART_c801	124	AIQ78384	0.0	GO:0004519	100	84.7297	41
ARC_ART_c807	150	XP_010505216	0.8	-	52	32.3426	38
ARC_ART_c812	109	XP_002950741	0.0	GO:0030170	88	60.8474	35
ARC_ART_c815	155	XP_005843958	0.0	-	56	37.3502	41
ARC_ART_c817	212	XP_009333814	0.1	-	51	35.8094	64
ARC_ART_c822	120	XP_002875865	0.3	-	54	33.8834	35
ARC_ART_c824	304	CAN80172	0.1	-	64	37.3502	39
ARC_ART_c836	170	EDQ48430	0.0	-	59	39.6614	49
ARC_ART_c842	147	XP_002979820	0.0	GO:0009657	62	48.521	45
ARC_ART_c843	154	CDX97840	0.0	-	55	41.2022	45
ARC_ART_c844	177	EPS74572	0.0	GO:0019752	71	62.7734	52
ARC_ART_c847	156	XP_002535428	0.0	GO:1902358	82	80.4925	52
ARC_ART_c849	158	XP_002537558	0.0	-	66	43.8986	48
ARC_ART_c851	186	XP_002538377	0.0	-	67	42.743	40
ARC_ART_c855	111	XP_010911770	0.0	-	88	53.5286	35
ARC_ART_c858	150	XP_011016603	0.0	GO:0004591	85	79.337	48

ARC_ART_c859	173	CDY23564	0.0	-	53	43.1282	54
ARC_ART_c861	280	ACN37093	0.0	-	49	35.4242	57
ARC_ART_c865	523	XP_002488976	0.0	-	59	48.9062	49
ARC_ART_c866	173	XP_002539781	0.0	-	65	40.4318	47
ARC_ART_c872	135	EEC79194	0.0	GO:0019752	88	73.559	44
ARC_ART_c876	176	EMS67607	0.6	-	65	33.4982	43
ARC_ART_c878	119	EEC67124	0.0	GO:0004325	84	61.2326	38
ARC_ART_c880	204	XP_008438902	0.0	GO:0003824	61	48.1358	57
ARC_ART_c883	133	XP_004500017	0.2	-	53	34.2686	39
ARC_ART_c887	178	XP_010242300	0.0	-	56	36.965	57
ARC_ART_c888	245	XP_003063226	0.0	GO:0004450	79	113.235	82
ARC_ART_c898	366	XP_007132740	0.0	-	60	48.1358	56
ARC_ART_c899	176	XP_002539780	0.0	-	61	35.4242	34
ARC_ART_c900	208	XP_009602703	0.4	-	56	34.2686	44
ARC_ART_c902	170	BAD94986	0.0	GO:0005739	83	80.4925	48
ARC_ART_c905	165	EDQ48403	0.0	-	72	52.7582	44
ARC_ART_c909	171	XP_002536517	0.0	GO:0050660	88	87.4261	54
ARC_ART_c912	183	KJB11774	0.0	-	81	57.3806	37
ARC_ART_c914	222	XP_002985145	0.1	-	58	36.1946	43
ARC_ART_c920	146	XP_003062922	0.6	-	66	32.3426	42
ARC_ART_c921	172	CAN68294	0.2	-	58	35.039	46
ARC_ART_c922	118	BAJ98151	0.0	-	68	40.0466	35
ARC_ART_c924	172	CAN60489	0.1	-	52	36.1946	44
ARC_ART_c925	173	XP_010911770	0.4	-	55	33.4982	40
ARC_ART_c929	137	XP_009350805	0.0	GO:1901363	76	63.5438	43
ARC_ART_c933	131	EMT06979	0.5	-	58	33.113	43
ARC_ART_c939	279	BAJ99415	0.0	GO:0006629	77	116.701	89
ARC_ART_c943	131	XP_002537871	0.0	-	74	41.9726	35
ARC_ART_c945	410	XP_007132740	0.0	-	93	127.102	66

ARC_ART_c948	244	XP_002538660	0.0	-	63	43.1282	41
ARC_ART_c949	127	XP_002969152	1.0	-	60	32.3426	38
ARC_ART_c951	261	XP_002535817	0.3	-	51	34.6538	74
ARC_ART_c962	104	XP_005849906	0.8	-	57	31.5722	35
ARC_ART_c964	111	KDP20270	0.3	-	63	33.4982	36
ARC_ART_c965	181	XP_002967717	0.0	GO:0005829	86	63.929	38
ARC_ART_c967	147	XP_002536081	0.0	GO:1902192	67	52.7582	40
ARC_ART_c971	120	XP_002537900	0.0	-	68	38.5058	38
ARC_ART_c975	118	AGG09511	0.0	GO:0003899	97	80.8777	39
ARC_ART_c978	196	XP_002538279	0.0	GO:0004177	89	109.768	64
ARC_ART_c981	130	KJB77245	0.6	-	55	31.5722	34
ARC_ART_c984	307	EEC80858	0.0	-	59	38.1206	44
ARC_ART_c985	151	EDQ48493	0.0	GO:0005488	72	64.6994	47
ARC_ART_c986	239	XP_002535323	0.0	GO:0051707	83	97.8265	77
ARC_ART_c988	177	XP_005850846	0.0	GO:0003887	55	43.1282	59
ARC_ART_c994	112	XP_002536731	0.0	-	62	40.4318	35
ARC_ART_c997	201	XP_001417314	0.0	GO:0044765	76	60.8474	42
ARC_ART_c1002	155	XP_005851307	0.0	GO:0015992	73	54.6842	46
ARC_ART_c1003	154	XP_003625472	0.0	GO:0035556	71	50.8322	42
ARC_ART_c1009	150	XP_002536367	0.0	GO:0020037	95	96.2857	48
ARC_ART_c1021	135	XP_007158282	0.6	-	60	33.113	38
ARC_ART_c1024	111	XP_006395129	0.1	-	72	35.039	36
ARC_ART_c1025	116	EEC69372	0.0	-	71	43.5134	38
ARC_ART_c1030	204	XP_005847344	0.0	-	66	63.5438	66
ARC_ART_c1033	185	CCO14729	0.0	-	56	38.1206	57
ARC_ART_c1036	194	XP_002535784	0.0	-	82	55.4546	39
ARC_ART_c1037	129	XP_002973884	0.2	-	66	34.2686	39
ARC_ART_c1038	157	XP_002535367	0.0	-	62	48.521	51

ARC_ART_c1041	314	YP_009105239	0.0	GO:0009536	56	46.2098	58
ARC_ART_c1046	173	XP_002984744	0.0	GO:0071704	61	42.743	57
ARC_ART_c1048	105	ACF86293	0.6	-	54	30.4166	35
ARC_ART_c1050	156	XP_002539959	0.0	GO:0050896	72	43.1282	43
ARC_ART_c1055	183	EMS59958	0.4	-	60	33.8834	55
ARC_ART_c1056	148	CDO97220	0.3	-	52	33.8834	46
ARC_ART_c1059	166	XP_001786575	0.5	-	48	33.113	49
ARC_ART_c1062	159	XP_004290822	0.0	GO:0019752	75	70.4774	52
ARC_ART_c1073	228	XP_002536366	0.0	-	90	102.449	55
ARC_ART_c1075	172	XP_001702436	0.3	-	53	34.2686	43
ARC_ART_c1081	160	EXB93351	0.0	GO:0004672	78	54.299	37
ARC_ART_c1096	225	XP_005643986	0.0	GO:0044763	75	47.7506	41
ARC_ART_c1102	156	XP_004309016	1.0	-	59	32.3426	42
ARC_ART_c1106	116	XP_006854853	0.0	-	60	36.965	35
ARC_ART_c1108	149	YP_001152206	0.0	-	60	46.595	53
ARC_ART_c1116	351	AEH27113	0.2	-	52	36.1946	44
ARC_ART_c1121	146	XP_003630582	0.0	GO:0008233	85	78.9518	48
ARC_ART_c1133	201	EAZ02189	0.0	-	60	43.8986	40
ARC_ART_c1135	199	KIY95278	0.0	GO:0009536	69	69.3218	63
ARC_ART_c1136	130	AFG71278	0.0	GO:0009536	83	56.225	43
ARC_ART_c1143	148	XP_008238954	0.0	GO:0009536	62	48.9062	45
ARC_ART_c1144	181	XP_009617105	0.7	-	57	33.113	49
ARC_ART_c1145	156	EDQ48453	0.0	-	65	37.7354	40
ARC_ART_c1146	117	XP_002537378	0.0	GO:0004871	76	53.5286	38
ARC_ART_c1149	153	XP_002960012	0.4	-	67	33.113	40
ARC_ART_c1150	263	XP_002536834	0.0	-	57	70.0922	87
ARC_ART_c1156	156	CCO14612	0.0	-	62	41.9726	45

ARC_ART_c1157	130	XP_003064451	0.0	-	62	41.5874	40
ARC_ART_c1162	186	XP_001786560	0.0	GO:0006289	79	72.4034	54
ARC_ART_c1169	174	NP_001054167	0.0	-	54	40.0466	50
ARC_ART_c1172	307	XP_011461855	0.3	-	53	35.4242	49
ARC_ART_c1180	151	BAI23037	0.0	GO:0044763	69	45.4394	43
ARC_ART_c1181	138	XP_003079479	0.0	-	68	36.5798	41
ARC_ART_c1182	216	EPS74531	0.0	-	57	36.1946	42
ARC_ART_c1185	166	XP_002539372	0.0	-	62	38.1206	53
ARC_ART_c1189	138	XP_001695969	0.3	-	53	33.8834	39
ARC_ART_c1192	163	XP_011469859	0.6	-	57	33.4982	57
ARC_ART_c1196	241	XP_002534890	0.0	GO:0006813	78	94.3597	73
ARC_ART_c1204	191	XP_002534959	0.0	GO:0008233	83	87.8113	65
ARC_ART_c1205	108	ACQ66327	0.0	-	65	38.1206	35
ARC_ART_c1206	151	XP_002537108	0.0	-	95	83.1889	42
ARC_ART_c1208	251	XP_007154367	0.0	-	79	49.6766	34
ARC_ART_c1212	222	XP_001780041	0.9	-	67	33.113	34
ARC_ART_c1216	257	KDO43708	0.1	-	50	36.5798	78
ARC_ART_c1219	173	XP_002986152	0.4	-	56	33.4982	51
ARC_ART_c1220	222	XP_002976667	0.3	-	50	34.6538	40
ARC_ART_c1226	172	XP_006402032	0.4	-	58	33.4982	41
ARC_ART_c1227	149	XP_002960004	0.0	-	56	39.2762	48
ARC_ART_c1229	131	XP_006386843	0.0	GO:0044699	74	52.7582	43
ARC_ART_c1230	211	XP_008359575	0.2	-	49	35.4242	63
ARC_ART_c1238	160	XP_002538779	0.0	GO:0050896	76	71.633	50

ARC_ART_c1244	130	XP_002504533	0.2	-	65	33.8834	38
ARC_ART_c1250	170	ERM98791	0.7	-	45	32.3426	53
ARC_ART_c1253	123	XP_002538146	0.0	GO:0016020	87	63.5438	39
ARC_ART_c1257	112	XP_002538477	0.0	GO:0008152	94	66.6254	36
ARC_ART_c1260	130	ACO50727	0.3	-	61	33.8834	36
ARC_ART_c1262	262	KGN44163	0.9	-	52	33.4982	70
ARC_ART_c1263	137	EEE67873	0.1	-	60	35.4242	45
ARC_ART_c1266	149	XP_001769997	0.0	GO:0046686	76	62.3882	47
ARC_ART_c1269	129	B23703	0.8	-	62	32.3426	37
ARC_ART_c1275	245	XP_011014247	0.0	GO:0019605	68	92.4337	80
ARC_ART_c1276	127	XP_002539972	0.0	-	74	39.2762	35
ARC_ART_c1277	280	XP_002535671	0.0	GO:0004333	92	95.1301	68
ARC_ART_c1281	138	XP_006393729	0.0	-	66	41.5874	39
ARC_ART_c1283	180	EEC76122	0.5	-	55	33.8834	54
ARC_ART_c1285	142	XP_002536189	0.0	-	61	35.4242	39
ARC_ART_c1288	191	XP_005644522	0.0	GO:0016020	63	47.3654	58
ARC_ART_c1293	314	EEE68528	0.0	GO:0055085	100	142.895	70
ARC_ART_c1299	166	XP_002539970	0.0	-	59	43.5134	57
ARC_ART_c1300	189	BAH57230	0.4	-	50	33.8834	42
ARC_ART_c1302	139	XP_009351102	0.8	-	51	32.7278	45
ARC_ART_c1304	166	XP_002539747	0.0	-	59	40.0466	54
ARC_ART_c1307	143	KDP21536	0.0	-	58	37.7354	41
ARC_ART_c1308	312	EMS59292	0.0	GO:0009941	56	46.2098	83
ARC_ART_c1310	153	EYU24102	0.0	-	64	36.965	34
ARC_ART_c1311	137	XP_008244560	0.9	-	57	31.9574	38
ARC_ART_c1314	203	EAY94034	0.0	GO:0043167	77	89.3521	58
ARC_ART_c1315	166	XP_002540328	0.0	-	66	42.3578	42

ARC_ART_c1316	139	XP_006288111	0.0	GO:0004124	84	82.8037	46
ARC_ART_c1317	276	XP_009758936	0.0	GO:0051287	69	76.6406	81
ARC_ART_c1324	145	XP_007227062	0.0	-	68	40.817	44
ARC_ART_c1326	134	XP_005643141	0.0	GO:0097159	70	45.0542	44
ARC_ART_c1327	130	KIY99335	0.0	GO:0016020	80	57.7658	40
ARC_ART_c1331	191	XP_008796927	0.0	GO:0017111	92	99.7525	63
ARC_ART_c1333	164	XP_005651109	0.0	GO:0009941	84	83.1889	50
ARC_ART_c1339	218	CDY18930	0.1	-	61	35.8094	34
ARC_ART_c1345	190	XP_010911538	0.0	-	91	84.3445	48
ARC_ART_c1349	132	XP_002535920	0.0	GO:0016798	70	43.1282	40
ARC_ART_c1352	215	XP_011080512	0.3	-	50	34.2686	40
ARC_ART_c1355	193	XP_001786704	0.0	GO:0015450	80	52.7582	41
ARC_ART_c1358	220	KEH15618	0.2	-	69	35.4242	43
ARC_ART_c1361	225	NP_001060556	0.0	GO:0008270	61	44.669	57
ARC_ART_c1364	268	EXC01912	0.0	-	73	41.2022	38
ARC_ART_c1365	112	XP_010492179	0.2	-	72	34.2686	36
ARC_ART_c1367	162	ACI87783	0.0	-	71	38.891	35
ARC_ART_c1368	149	ERN04671	0.0	GO:0016491	69	45.0542	42
ARC_ART_c1370	146	XP_001768590	0.0	-	77	49.6766	36
ARC_ART_c1371	139	EEE59053	0.0	-	64	36.1946	42
ARC_ART_c1373	285	YP_001152214	0.0	-	48	36.5798	76
ARC_ART_c1374	174	XP_008347297	0.9	-	46	32.7278	50
ARC_ART_c1379	173	XP_005852111	1.0	-	50	32.7278	55
ARC_ART_c1380	146	NP_038385	0.0	GO:0003899	82	51.2174	35
ARC_ART_c1387	164	XP_002535596	0.9	-	47	31.9574	53
ARC_ART_c1390	111	XP_002538125	0.0	GO:0004519	77	48.9062	35
ARC_ART_c1395	201	XP_002535112	0.0	-	68	63.1586	66
ARC_ART_c1396	200	EEC76953	0.0	-	60	38.1206	48

ARC_ART_c1401	148	XP_002535669	0.0	-	69	51.6026	49
ARC_ART_c1405	196	XP_005646550	0.0	-	67	68.9366	61
ARC_ART_c1408	109	EPS72132	0.0	-	70	35.039	34
ARC_ART_c1414	234	KEH15469	0.3	-	47	35.039	76
ARC_ART_c1425	209	XP_011078446	0.1	-	41	35.8094	63
ARC_ART_c1427	148	EAY75387	0.3	-	54	34.2686	44
ARC_ART_c1430	143	EEC73650	0.0	-	68	39.2762	35
ARC_ART_c1432	607	XP_005850993	0.0	GO:0016779	85	299.671	198
ARC_ART_c1433	112	XP_002976501	0.0	-	63	36.5798	38
ARC_ART_c1435	138	EEE52323	0.0	-	64	48.9062	45
ARC_ART_c1436	136	XP_002872330	0.2	-	58	34.2686	39
ARC_ART_c1439	184	XP_001415650	0.0	-	60	50.0618	60
ARC_ART_c1440	123	NP_001119208	0.9	-	41	31.5722	36
ARC_ART_c1447	126	XP_009387948	0.0	GO:0051287	76	50.0618	34
ARC_ART_c1450	110	XP_002535893	0.1	-	68	35.4242	35
ARC_ART_c1451	135	XP_001416687	0.0	GO:0030170	86	71.2478	43
ARC_ART_c1455	134	XP_001762156	0.0	GO:0003824	67	45.8246	37
ARC_ART_c1456	123	XP_005645589	0.0	-	66	39.2762	39
ARC_ART_c1458	119	XP_006357639	0.1	-	64	35.4242	39
ARC_ART_c1464	158	XP_002538377	0.0	-	70	50.447	44
ARC_ART_c1466	204	AAR89868	0.0	GO:0006261	77	72.4034	58
ARC_ART_c1469	120	XP_001693400	0.0	-	69	36.1946	36
ARC_ART_c1495	129	NP_001154660	0.8	-	50	32.7278	42
ARC_ART_c1498	107	EEC69469	0.0	GO:0046835	100	74.3294	34
ARC_ART_c1500	200	XP_009782486	1.0	-	54	33.113	53
ARC_ART_c1505	212	XP_003589934	0.5	-	58	32.3426	41
ARC_ART_c1506	154	EAZ02189	0.0	-	62	44.2838	40

ARC_ART_c1510	172	XP_001752808	0.0	-	59	37.3502	42
ARC_ART_c1512	131	XP_001761573	0.0	GO:0016740	73	50.0618	38
ARC_ART_c1514	190	CDY07402	0.9	-	54	32.7278	42
ARC_ART_c1519	143	XP_004493857	0.6	-	58	31.9574	43
ARC_ART_c1522	112	AFK37932	0.0	GO:0016627	64	41.2022	34
ARC_ART_c1537	143	XP_009351044	0.0	GO:0043565	78	64.6994	47
ARC_ART_c1551	261	XP_001780597	0.0	GO:0044237	59	64.6994	93
ARC_ART_c1552	152	XP_003570127	0.6	-	58	32.3426	43
ARC_ART_c1554	154	XP_007163768	0.1	-	53	35.8094	41
ARC_ART_c1557	178	XP_002534650	0.1	-	50	36.5798	51
ARC_ART_c1563	144	XP_002537066	0.0	-	80	48.1358	35
ARC_ART_c1568	202	KJB76205	0.7	-	54	33.113	51
ARC_ART_c1569	204	CAN62566	0.5	-	60	33.8834	40
ARC_ART_c1572	134	XP_002540080	0.0	GO:0009058	85	63.5438	40
ARC_ART_c1574	131	ABK24522	0.0	GO:0043229	72	52.7582	43
ARC_ART_c1575	162	XP_011015935	0.0	-	76	47.7506	38
ARC_ART_c1576	262	XP_002985008	0.0	-	59	61.2326	83
ARC_ART_c1595	149	XP_002946158	0.3	-	53	33.4982	47
ARC_ART_c1596	198	XP_002536531	0.3	-	63	34.6538	41
ARC_ART_c1598	111	CDX92904	0.1	-	67	34.6538	34
ARC_ART_c1600	129	EXC22241	0.0	GO:0016787	100	81.6481	38
ARC_ART_c1601	186	XP_009351044	0.0	-	70	68.1662	60
ARC_ART_c1604	125	AAT68205	0.0	GO:0004149	80	55.0694	40
ARC_ART_c1607	177	XP_010911647	0.0	GO:0010039	63	57.3806	55
ARC_ART_c1614	146	XP_002536964	0.0	-	71	36.1946	42

ARC_ART_c1619	156	XP_002514528	0.9	-	53	32.3426	41
ARC_ART_c1627	203	XP_002536536	0.0	-	57	53.1434	71
ARC_ART_c1628	251	XP_002955800	0.0	-	69	80.8777	82
ARC_ART_c1629	208	XP_002972019	0.0	GO:0016884	83	88.9669	62
ARC_ART_c1635	119	XP_002540460	0.2	-	58	33.8834	36
ARC_ART_c1640	401	XP_011013113	0.0	-	78	74.7146	50
ARC_ART_c1641	143	XP_002538442	0.0	GO:0046872	58	53.1434	41
ARC_ART_c1645	145	XP_005846025	0.0	GO:0003824	75	63.1586	45
ARC_ART_c1651	216	XP_003637954	1.0	-	46	32.7278	58
ARC_ART_c1657	197	XP_001786560	0.0	GO:0006289	86	77.0258	46
ARC_ART_c1660	317	XP_001419031	0.0	GO:0034660	63	95.5153	97
ARC_ART_c1662	363	EXC01914	0.0	-	60	55.8398	56
ARC_ART_c1666	249	XP_002959312	0.0	GO:0016747	70	95.1301	82
ARC_ART_c1669	160	XP_001690094	0.0	GO:0006596	65	50.447	46
ARC_ART_c1670	375	XP_009146915	0.7	-	38	35.039	83
ARC_ART_c1679	159	XP_011623344	0.0	GO:0009536	61	45.0542	42
ARC_ART_c1681	266	AAF16526	0.0	GO:0007010	89	146.747	86
ARC_ART_c1688	123	XP_002535972	0.0	-	70	41.5874	40
ARC_ART_c1689	122	AFK44444	0.0	-	67	48.1358	37
ARC_ART_c1695	115	YP_009020823	0.1	-	70	35.039	34
ARC_ART_c1698	218	XP_003530746	0.8	-	49	33.4982	59
ARC_ART_c1701	327	EXC01915	0.0	-	58	46.2098	51
ARC_ART_c1708	160	XP_002535338	0.0	-	76	59.6918	51
ARC_ART_c1709	131	XP_002534653	0.1	-	58	33.8834	43
ARC_ART_c1712	271	XP_002536752	0.0	GO:0004364	92	93.9745	55
ARC_ART_c1714	211	XP_003637074	0.0	GO:0009536	74	67.781	47
ARC_ART_c1717	214	XP_002535648	0.0	-	67	38.891	40
ARC_ART_c1724	325	XP_001419056	0.0	-	55	68.9366	88

ARC_ART_c1739	125	XP_002980166	0.1	-	48	35.039	35
ARC_ART_c1741	160	XP_002536196	0.0	GO:0016021	77	65.855	53
ARC_ART_c1743	150	EXC03805	0.0	GO:0042802	70	50.447	40
ARC_ART_c1748	190	XP_002540066	0.0	GO:0006164	77	59.6918	62
ARC_ART_c1753	125	EDQ48547	0.0	GO:0016021	82	45.8246	39
ARC_ART_c1762	157	XP_002947541	0.0	-	63	46.595	52
ARC_ART_c1764	224	CDO99597	0.1	-	57	35.8094	47
ARC_ART_c1767	108	ADV19276	0.0	-	77	41.2022	35
ARC_ART_c1776	176	XP_002540328	0.6	-	50	33.113	53
ARC_ART_c1779	111	ADK98242	0.0	GO:0016787	72	47.7506	36
ARC_ART_c1781	164	KFM27113	0.0	GO:0044763	72	55.8398	44
ARC_ART_c1788	242	XP_008246538	0.9	-	57	33.4982	38
ARC_ART_c1794	181	XP_001786560	0.0	GO:0006289	90	101.293	60
ARC_ART_c1801	177	XP_002507787	0.0	GO:0008152	66	60.8474	56
ARC_ART_c1811	102	KEH15702	0.8	-	67	32.3426	34
ARC_ART_c1819	167	XP_002989988	0.0	-	57	36.5798	49
ARC_ART_c1821	138	XP_002538276	0.0	-	69	44.2838	46
ARC_ART_c1822	176	XP_002526025	0.0	GO:0044699	63	51.9878	47
ARC_ART_c1823	176	CCO14435	0.0	GO:0044249	63	43.5134	38
ARC_ART_c1828	136	P31683	0.0	GO:0006096	86	68.9366	45
ARC_ART_c1829	151	XP_003081165	0.1	-	65	36.1946	44
ARC_ART_c1830	243	XP_002945866	0.0	-	56	48.521	65
ARC_ART_c1839	277	XP_002539100	0.0	GO:0016740	61	54.6842	75
ARC_ART_c1842	280	KEH15234	0.0	GO:0034641	69	80.1073	73
ARC_ART_c1847	125	XP_009759241	0.0	GO:0000166	75	43.8986	36

ARC_ART_c1853	114	XP_002530286	0.0	-	62	38.1206	37
ARC_ART_c1858	135	XP_002536193	0.0	GO:0015416	88	70.4774	43
ARC_ART_c1861	129	XP_002950210	0.0	-	65	37.7354	35
ARC_ART_c1865	178	XP_007044407	0.8	-	57	33.113	40
ARC_ART_c1866	229	XP_009350805	0.0	GO:0006399	71	89.3521	69
ARC_ART_c1868	168	XP_001780072	0.0	GO:0004672	65	47.3654	52
ARC_ART_c1871	122	XP_002535911	0.0	-	70	41.9726	37
ARC_ART_c1874	273	KJB49896	0.9	-	48	32.7278	62
ARC_ART_c1879	155	CDY00690	0.0	GO:0006261	71	59.3066	53
ARC_ART_c1881	191	XP_001781129	0.0	-	55	40.817	43
ARC_ART_c1885	120	EEC68664	0.0	GO:0043168	76	51.9878	39
ARC_ART_c1889	378	EPS74505	0.0	GO:0009507	89	91.2781	56
ARC_ART_c1895	196	XP_002537681	0.7	-	59	33.113	64
ARC_ART_c1900	118	XP_005848590	0.3	-	68	33.4982	38
ARC_ART_c1903	193	XP_009350057	0.0	-	77	61.2326	44
ARC_ART_c1905	147	EEC74306	0.2	-	65	34.6538	40
ARC_ART_c1910	108	ABR26094	0.2	-	56	31.9574	39
ARC_ART_c1911	166	XP_007020246	0.7	-	52	33.113	38
ARC_ART_c1914	183	EEC80863	0.0	-	70	58.5362	60
ARC_ART_c1916	186	XP_005646123	0.0	GO:0005488	69	65.4698	59
ARC_ART_c1919	121	XP_002953597	0.0	-	75	45.0542	37
ARC_ART_c1924	120	XP_002946575	0.7	-	59	32.7278	37
ARC_ART_c1925	118	XP_002535661	0.0	-	71	42.3578	38
ARC_ART_c1926	234	XP_002535080	0.0	GO:0007000	55	47.3654	65
ARC_ART_c1940	110	XP_010437044	0.0	GO:0044249	67	45.0542	34
ARC_ART_c1950	188	XP_002535920	0.0	GO:0090599	74	66.2402	62
ARC_ART_c1951	266	EEC76953	0.0	GO:0009058	75	55.8398	41
ARC_ART_c1954	126	KDD73829	0.0	GO:0048364	85	63.1586	41

ARC_ART_c1964	249	XP_002538398	0.0	GO:0009987	64	54.299	70
ARC_ART_c1973	194	KEH15415	0.0	GO:1901363	69	75.8702	63
ARC_ART_c1974	149	KIY95436	0.0	GO:0016491	81	46.2098	38
ARC_ART_c1979	167	XP_002540194	0.0	GO:0016740	70	50.447	40
ARC_ART_c1981	220	XP_011016741	0.0	-	81	66.6254	44
ARC_ART_c1985	120	ABX45865	0.0	GO:0046872	74	53.9138	39
ARC_ART_c1987	121	KDD74350	0.0	GO:0006457	81	58.9214	38
ARC_ART_c1992	113	XP_011015412	0.0	-	64	37.3502	34
ARC_ART_c1993	118	KFM27553	0.0	-	64	36.1946	34
ARC_ART_c1996	191	KJB49896	0.0	-	56	37.7354	44
ARC_ART_c2000	175	CEF98227	0.1	-	61	36.1946	44
ARC_ART_c2004	104	XP_002536843	0.0	-	79	57.3806	34
ARC_ART_c2005	125	XP_001422468	0.0	GO:0044710	68	45.0542	41
ARC_ART_c2007	278	XP_011014207	0.2	-	52	35.4242	74
ARC_ART_c2008	131	XP_002518106	0.2	-	58	34.6538	36
ARC_ART_c2015	232	XP_007132740	0.0	-	64	45.4394	37
ARC_ART_c2020	108	XP_002534861	0.0	GO:0030170	91	64.3142	34
ARC_ART_c2025	170	XP_002536262	0.0	GO:0006820	78	54.6842	38
ARC_ART_c2061	122	XP_001786560	0.0	GO:0006289	79	57.7658	39
ARC_ART_c2066	185	CCO20352	0.0	GO:0006164	73	43.1282	34
ARC_ART_c2072	130	EEC66960	0.0	GO:0006810	87	66.6254	41
ARC_ART_c2076	246	KDP20462	0.0	-	69	59.3066	49
ARC_ART_c2079	136	XP_002539615	0.0	-	62	40.4318	35
ARC_ART_c2090	152	CAN80172	0.9	-	50	30.4166	38
ARC_ART_c2093	110	XP_002538548	0.0	-	66	42.3578	36
ARC_ART_c2102	152	XP_006409984	0.2	-	65	34.6538	49
ARC_ART_c2105	220	XP_002466743	1.0	-	55	33.113	38
ARC_ART_c2107	124	KFM25862	0.0	GO:0003989	87	69.707	40

ARC_ART_c2108	118	XP_007014014	0.0	GO:0032259	82	59.3066	39
ARC_ART_c2112	147	XP_002538398	0.0	-	55	36.5798	47
ARC_ART_c2116	185	XP_002537276	0.0	-	70	55.8398	44
ARC_ART_c2125	137	EMT19079	0.0	-	52	34.2686	36
ARC_ART_c2137	115	ABC24948	0.0	GO:0005737	78	56.6102	38
ARC_ART_c2143	161	AHY28821	0.0	GO:0004478	79	70.0922	53
ARC_ART_c2144	127	XP_002539285	0.2	-	61	33.8834	36
ARC_ART_c2146	150	XP_002535045	0.0	-	93	92.4337	46
ARC_ART_c2147	250	YP_358637	0.0	-	65	39.6614	43
ARC_ART_c2149	233	AGB85039	0.1	-	59	34.6538	37
ARC_ART_c2151	154	EPS62231	0.0	GO:0005524	76	65.0846	51
ARC_ART_c2154	238	XP_002538709	0.0	-	73	93.2041	76
ARC_ART_c2156	127	XP_010505628	0.0	-	65	36.1946	44
ARC_ART_c2157	146	CDY24452	0.1	-	61	35.8094	39
ARC_ART_c2163	190	XP_009119279	0.7	-	57	33.113	52
ARC_ART_c2168	174	XP_002540131	0.0	GO:0005488	83	79.7221	56
ARC_ART_c2171	189	KEH16996	0.1	-	54	36.965	51
ARC_ART_c2175	178	XP_008233928	0.7	-	46	33.113	45
ARC_ART_c2176	132	XP_002534193	0.0	GO:0016021	79	53.1434	39
ARC_ART_c2180	148	XP_009383645	0.5	-	53	33.4982	41
ARC_ART_c2182	157	YP_006280957	0.0	-	68	47.7506	50
ARC_ART_c2187	119	XP_002539702	0.0	GO:0016491	66	48.1358	39
ARC_ART_c2197	223	EAZ10012	0.0	-	58	55.0694	70
ARC_ART_c2199	119	EEC77198	0.0	GO:0006259	72	47.7506	37
ARC_ART_c2204	282	XP_010909278	0.8	-	54	33.4982	37
ARC_ART_c2216	175	XP_005850227	0.4	-	63	33.113	36
ARC_ART_c2223	209	XP_008366477	0.0	GO:0004834	84	61.6178	39
ARC_ART_c2224	170	XP_002535691	0.0	-	74	38.5058	43

ARC_ART_c2227	141	XP_002537786	0.0	-	63	45.4394	47
ARC_ART_c2233	123	XP_001786560	0.0	GO:0044763	64	44.669	37
ARC_ART_c2244	145	XP_001700222	0.0	GO:0008750	91	77.7962	48
ARC_ART_c2246	131	XP_009385421	0.1	-	70	35.039	34
ARC_ART_c2248	161	KDP38398	0.8	-	57	32.3426	52
ARC_ART_c2250	236	XP_006650531	0.4	-	63	34.2686	38
ARC_ART_c2252	226	EEE54984	0.0	GO:0005737	98	153.295	74
ARC_ART_c2254	224	XP_001755595	0.0	-	56	49.2914	71
ARC_ART_c2258	146	XP_008441879	0.0	-	60	44.2838	46
ARC_ART_c2259	126	XP_005645707	0.0	GO:0008152	73	49.2914	41
ARC_ART_c2261	195	XP_008643987	0.3	-	61	34.2686	36
ARC_ART_c2262	128	NP_001149732	0.0	-	64	40.4318	42
ARC_ART_c2264	125	EMT00231	0.0	GO:0006355	92	67.781	39
ARC_ART_c2267	124	XP_005847301	0.0	-	58	42.743	41
ARC_ART_c2276	151	EEC76122	0.0	-	62	32.7278	37
ARC_ART_c2279	150	XP_002540033	0.0	-	75	76.6406	49
ARC_ART_c2291	159	CCH47171	0.0	GO:0006098	70	55.8398	47
ARC_ART_c2298	134	EEE54984	0.0	GO:0004819	82	64.3142	40
ARC_ART_c2302	163	XP_002535215	0.0	GO:0016021	88	65.855	43
ARC_ART_c2313	137	XP_003626378	0.7	-	51	32.7278	43
ARC_ART_c2315	133	XP_002535671	0.0	GO:0004333	92	77.411	39
ARC_ART_c2327	112	XP_009351044	0.0	-	65	36.965	35
ARC_ART_c2329	171	XP_002537029	0.0	-	60	43.5134	45

ARC_ART_c2332	188	XP_001771154	0.0	GO:0006090	70	58.151	50
ARC_ART_c2337	138	KJB09764	0.0	-	59	36.965	47
ARC_ART_c2342	176	EEE67873	0.0	-	67	49.2914	43
ARC_ART_c2349	337	KEH22228	0.1	-	56	36.5798	57
ARC_ART_c2350	110	AFI43489	0.0	GO:0006097	80	56.225	35
ARC_ART_c2356	191	AAD25611	0.0	GO:0009507	73	55.4546	57
ARC_ART_c2358	198	BAK07132	0.8	-	61	33.113	34
ARC_ART_c2360	175	YP_004581348	0.0	GO:0003899	77	65.855	49
ARC_ART_c2363	233	XP_009350065	0.0	-	80	76.6406	62
ARC_ART_c2365	149	XP_002539813	0.0	GO:0071704	75	57.7658	49
ARC_ART_c2366	116	XP_002535989	0.0	-	67	43.5134	37
ARC_ART_c2368	182	XP_010239583	0.0	-	57	40.0466	45
ARC_ART_c2375	435	XP_011016264	0.0	GO:0008272	80	174.096	141
ARC_ART_c2381	221	XP_010447546	0.4	-	52	33.8834	53
ARC_ART_c2382	109	AFK47956	0.0	GO:0009561	80	48.1358	36
ARC_ART_c2386	166	XP_002537860	0.0	GO:1901363	69	58.5362	55
ARC_ART_c2391	105	BAK07351	0.6	-	61	32.7278	34
ARC_ART_c2393	184	CDX81537	0.0	GO:0005886	81	56.9954	37
ARC_ART_c2398	193	XP_006295601	0.0	GO:0016021	81	75.0998	48
ARC_ART_c2400	217	KDD77116	0.0	-	51	50.8322	70
ARC_ART_c2404	179	XP_003614389	0.0	-	77	40.4318	35
ARC_ART_c2408	150	XP_002537786	0.0	-	61	42.743	44
ARC_ART_c2411	187	XP_001786938	0.0	GO:0016740	58	43.1282	53
ARC_ART_c2420	189	XP_011013113	0.0	-	75	48.521	36
ARC_ART_c2423	167	XP_005649685	0.0	GO:0016853	70	60.8474	54

ARC_ART_c2424	103	AAC25986	0.0	GO:0004834	82	48.1358	34
ARC_ART_c2426	237	XP_009350814	0.0	-	75	64.6994	57
ARC_ART_c2427	333	XP_002967566	1.0	-	48	33.8834	56
ARC_ART_c2430	123	XP_002536183	0.0	-	68	49.6766	38
ARC_ART_c2433	292	CDY63595	0.0	-	63	53.9138	63
ARC_ART_c2434	108	BAJ99459	0.0	GO:0050660	88	65.4698	35
ARC_ART_c2436	256	XP_010911593	0.0	-	62	41.5874	58
ARC_ART_c2438	107	XP_001694351	0.2	-	48	33.8834	35
ARC_ART_c2445	109	EEC68416	0.0	GO:0000166	68	44.2838	35
ARC_ART_c2451	117	BAH23787	0.0	GO:0016740	70	55.4546	44
ARC_ART_c2460	136	CDO96986	0.1	-	54	33.4982	44
ARC_ART_c2462	153	XP_002539346	0.1	-	63	35.039	49
ARC_ART_c2472	176	KGN55456	0.0	-	59	37.7354	37
ARC_ART_c2476	117	KDD73397	0.0	GO:0016620	76	52.373	38
ARC_ART_c2477	118	EEC69372	0.1	-	70	35.4242	34
ARC_ART_c2480	180	XP_003061384	0.0	GO:0004124	79	93.2041	59
ARC_ART_c2486	130	EAY97777	0.0	GO:0009058	80	51.2174	41
ARC_ART_c2500	129	CDY55675	0.2	-	60	33.8834	38
ARC_ART_c2515	223	EPS74505	0.0	-	82	54.6842	34
ARC_ART_c2527	126	EAY93456	0.0	GO:0004364	78	52.7582	41
ARC_ART_c2532	127	XP_001786710	0.0	-	69	38.5058	39
ARC_ART_c2536	177	XP_002948757	0.3	-	48	32.3426	45
ARC_ART_c2541	209	XP_002539813	0.0	GO:0016301	78	45.0542	37
ARC_ART_c2544	131	EAZ43720	0.0	GO:0006810	97	73.1738	39
ARC_ART_c2550	226	ABR16580	0.0	-	63	58.151	77
ARC_ART_c2555	171	XP_010911304	0.0	-	70	49.2914	51

ARC_ART_c2556	121	XP_002537971	0.3	-	57	33.113	40
ARC_ART_c2558	127	XP_002540478	0.0	GO:0003824	67	43.1282	34
ARC_ART_c2559	127	XP_002536517	0.0	GO:0050660	82	53.5286	35
ARC_ART_c2562	113	XP_004294943	0.0	-	67	39.6614	37
ARC_ART_c2571	105	XP_002535087	0.0	-	65	39.6614	35
ARC_ART_c2575	166	CAA74040	0.0	GO:0031047	88	51.9878	34
ARC_ART_c2577	106	AIA96001	0.0	-	75	43.5134	36
ARC_ART_c2581	111	BAJ98858	0.0	GO:0008152	71	44.669	35
ARC_ART_c2594	206	XP_005651441	0.0	GO:0097053	81	87.4261	59
ARC_ART_c2595	133	XP_002539124	0.0	-	82	47.7506	35
ARC_ART_c2596	126	XP_002534841	0.0	GO:0044763	74	53.5286	39
ARC_ART_c2597	169	NP_001184962	0.7	-	48	33.113	43
ARC_ART_c2598	221	XP_010035022	0.6	-	54	33.8834	53
ARC_ART_c2606	210	CAN68613	1.0	-	48	33.113	49
ARC_ART_c2611	179	EMT06200	0.5	-	60	33.4982	38
ARC_ART_c2618	148	EEE52320	0.0	GO:0055085	95	87.4261	43
ARC_ART_c2625	214	AAK49070	0.0	-	62	34.6538	35
ARC_ART_c2626	126	XP_004497139	0.6	-	68	31.9574	35
ARC_ART_c2629	156	XP_002536028	0.0	-	64	50.447	48
ARC_ART_c2630	274	XP_002535920	0.0	GO:0005975	89	139.043	79
ARC_ART_c2637	292	XP_011016741	0.0	-	100	116.316	56
ARC_ART_c2644	132	XP_006859134	0.0	-	68	40.0466	35
ARC_ART_c2648	150	XP_011016735	0.0	GO:0071704	74	55.4546	50
ARC_ART_c2654	147	XP_009361417	0.0	-	60	38.891	45
ARC_ART_c2667	118	AAO39037	0.0	-	70	44.2838	34
ARC_ART_c2672	139	XP_006591064	0.3	-	60	33.8834	43

ARC_ART_c2685	125	XP_002537329	0.0	-	84	65.855	38
ARC_ART_c2690	130	XP_002535213	0.1	-	75	35.039	36
ARC_ART_c2693	269	EEC77998	0.0	-	76	62.7734	42
ARC_ART_c2696	243	XP_002948755	0.0	-	67	37.3502	34
ARC_ART_c2698	155	KIY95278	0.0	-	58	39.6614	48
ARC_ART_c2701	113	XP_006357757	0.0	-	63	39.6614	36
ARC_ART_c2705	131	XP_002540597	0.1	-	61	33.4982	36
ARC_ART_c2706	120	XP_002535303	0.0	-	67	45.8246	37
ARC_ART_c2709	173	XP_008344910	0.3	-	45	34.6538	51
ARC_ART_c2711	252	XP_010486385	0.0	-	55	37.7354	52
ARC_ART_c2712	164	XP_001697756	0.0	GO:0080041	80	81.2629	52
ARC_ART_c2716	315	NP_050914	0.0	-	66	48.1358	56
ARC_ART_c2718	272	EPS74511	0.0	-	74	82.8037	59
ARC_ART_c2723	234	XP_003637074	0.0	-	61	40.817	44
ARC_ART_c2726	230	CCO17886	0.0	-	66	39.6614	39
ARC_ART_c2732	364	EXC01914	0.0	-	55	41.2022	59
ARC_ART_c2736	191	XP_001758543	0.0	-	66	44.669	39
ARC_ART_c2740	124	XP_011015935	0.0	-	70	46.595	37
ARC_ART_c2743	137	XP_007214188	0.0	-	59	38.5058	44
ARC_ART_c2747	229	CDP00645	0.1	-	53	35.8094	41
ARC_ART_c2750	132	EPS62049	0.7	-	64	32.7278	37
ARC_ART_c2751	201	XP_002949227	0.4	-	57	34.2686	40
ARC_ART_c2752	207	EPS74505	0.0	GO:0009507	79	65.0846	44
ARC_ART_c2757	215	XP_010508251	0.5	-	52	33.4982	55
ARC_ART_c2760	152	XP_010434296	0.1	-	62	34.6538	37

ARC_ART_c2763	111	XP_002537173	0.1	-	63	33.8834	36
ARC_ART_c2786	155	XP_002528144	0.1	-	52	35.8094	48
ARC_ART_c2793	119	XP_002537690	0.1	-	72	35.039	37
ARC_ART_c2810	132	EEC68421	0.0	GO:0016020	82	48.521	34
ARC_ART_c2812	166	XP_006844214	0.8	-	56	32.7278	37
ARC_ART_c2825	226	XP_008385716	0.0	GO:0008705	83	109.383	67
ARC_ART_c2827	436	XP_002538597	0.0	GO:0044763	53	78.5666	115
ARC_ART_c2839	202	DAA48678	0.2	-	50	33.8834	67
ARC_ART_c2842	133	CEF98367	0.0	-	65	38.5058	41
ARC_ART_c2845	162	XP_002447627	0.2	-	54	34.6538	42
ARC_ART_c2852	132	AAW30411	0.0	-	60	40.817	43
ARC_ART_c2857	113	CDP17952	0.0	GO:0006096	86	61.2326	36
ARC_ART_c2865	162	NP_001145246	0.4	-	48	32.7278	43
ARC_ART_c2866	138	KDO68907	0.9	-	55	32.7278	36
ARC_ART_c2867	194	KFM28619	0.0	GO:0030529	76	58.9214	46
ARC_ART_c2874	119	XP_002960330	0.1	-	73	35.039	34
ARC_ART_c2877	114	XP_002537854	0.0	-	75	43.5134	37
ARC_ART_c2883	311	EPS62117	0.0	-	60	37.7354	35
ARC_ART_c2885	226	XP_002320062	0.8	-	47	33.4982	72
ARC_ART_c2892	130	KJB09782	0.8	-	57	32.7278	42
ARC_ART_c2894	192	XP_001703326	0.0	-	68	56.6102	44
ARC_ART_c2897	249	XP_008460752	0.0	-	58	42.743	46
ARC_ART_c2900	169	XP_004972519	0.9	-	54	32.7278	44
ARC_ART_c2905	206	XP_002535042	0.0	GO:0016787	73	68.9366	46
ARC_ART_c2917	226	EPS60517	0.0	-	55	38.5058	43
ARC_ART_c2922	155	XP_001786754	0.1	-	62	35.039	43

ARC_ART_c2925	202	EYU17933	0.6	-	46	33.4982	60
ARC_ART_c2926	150	CBI18549	0.0	GO:0055072	70	51.9878	48
ARC_ART_c2933	138	XP_002536250	0.0	-	93	83.9593	45
ARC_ART_c2935	111	XP_005648475	0.1	-	62	35.8094	37
ARC_ART_c2938	137	XP_002539275	0.0	-	69	39.2762	36
ARC_ART_c2946	105	XP_002954758	0.4	-	58	33.113	34
ARC_ART_c2949	187	CBI36528	0.0	-	70	54.6842	44
ARC_ART_c2951	171	XP_006656912	0.9	-	64	33.113	34
ARC_ART_c2954	145	XP_009377366	0.0	-	67	36.1946	34
ARC_ART_c2957	170	XP_002537860	0.0	GO:0050794	74	59.3066	54
ARC_ART_c2963	228	KFM28355	0.0	-	60	38.891	56
ARC_ART_c2966	117	XP_001776046	0.6	-	63	32.7278	38
ARC_ART_c2975	127	XP_003079729	0.0	GO:0044237	71	47.3654	39
ARC_ART_c2976	183	XP_003588326	0.0	-	55	38.5058	54
ARC_ART_c2981	155	XP_002501044	0.0	-	60	43.1282	38
ARC_ART_c2988	180	XP_002534940	0.0	GO:0016810	74	66.2402	59
ARC_ART_c2989	113	XP_002537707	0.0	-	68	38.891	35
ARC_ART_c3003	117	CCO15754	0.7	-	54	32.3426	42
ARC_ART_c3009	295	XP_002501910	0.0	-	67	97.8265	92
ARC_ART_c3010	148	EAY84569	0.0	GO:0033539	89	85.8853	49
ARC_ART_c3017	222	XP_002536609	0.0	GO:0003824	63	66.2402	69
ARC_ART_c3019	109	BAB10615	0.4	-	65	33.4982	35
ARC_ART_c3022	218	XP_002974989	0.0	GO:0051536	75	77.411	60
ARC_ART_c3036	111	XP_005850521	0.0	GO:0016020	77	51.6026	40
ARC_ART_c3043	126	XP_009350076	0.0	GO:0034660	97	81.2629	42
ARC_ART_c3045	139	XP_002539344	0.1	-	64	35.8094	39
ARC_ART_c3048	145	XP_002535748	0.0	GO:0008483	77	69.3218	48
ARC_ART_c3054	226	XP_002535455	0.0	GO:0046872	79	42.743	48
ARC_ART_c3055	143	XP_002540587	0.3	-	75	33.113	41

ARC_ART_c3057	230	XP_010911665	0.0	GO:0044249	73	69.707	52
ARC_ART_c3062	181	XP_010934194	0.6	-	58	33.4982	50
ARC_ART_c3075	313	XP_005651253	0.0	GO:0005829	71	109.383	103
ARC_ART_c3076	135	KIZ02124	0.0	GO:0008152	72	55.0694	44
ARC_ART_c3090	211	XP_002540595	0.6	-	50	33.113	65
ARC_ART_c3100	182	ABA86578	0.0	GO:0006457	88	77.0258	45
ARC_ART_c3101	109	XP_010497619	0.4	-	66	32.3426	36
ARC_ART_c3103	142	XP_002537905	0.0	-	73	63.5438	46
ARC_ART_c3105	174	XP_002535370	0.0	-	50	36.1946	58
ARC_ART_c3106	139	XP_008374443	0.5	-	60	33.113	38
ARC_ART_c3107	144	XP_002533651	0.3	-	54	33.8834	42
ARC_ART_c3140	123	ERN07643	0.0	-	100	82.0333	39
ARC_ART_c3146	166	EPS62405	0.0	GO:0055114	87	93.5893	55
ARC_ART_c3149	126	XP_009419981	0.0	GO:0044260	73	54.6842	45
ARC_ART_c3153	123	EEC77111	0.0	-	80	36.5798	40
ARC_ART_c3155	106	XP_002538390	0.0	GO:0003824	94	72.0182	35
ARC_ART_c3156	162	XP_010238899	0.6	-	60	33.4982	43
ARC_ART_c3166	182	XP_002539002	0.0	-	54	48.9062	53
ARC_ART_c3178	281	EDQ48391	0.0	GO:0005829	84	150.214	92
ARC_ART_c3196	161	XP_002502109	0.0	-	65	39.2762	40
ARC_ART_c3199	163	XP_001786574	0.0	GO:0003824	75	66.2402	52
ARC_ART_c3201	138	KIY98401	0.0	GO:0006164	82	57.7658	39
ARC_ART_c3202	148	XP_002539120	0.0	-	66	42.743	39
ARC_ART_c3203	126	CDY16436	0.0	-	69	36.965	36
ARC_ART_c3210	157	AAF16525	0.0	GO:0001510	87	82.0333	48
ARC_ART_c3214	176	XP_011015251	0.0	-	65	46.2098	46

ARC_ART_c3225	120	XP_006373231	0.2	-	62	33.8834	35
ARC_ART_c3230	246	XP_009338215	0.6	-	47	34.2686	46
ARC_ART_c3234	128	XP_007051061	0.0	GO:0006541	81	49.2914	37
ARC_ART_c3235	170	EMS60018	0.1	-	54	35.4242	48
ARC_ART_c3259	142	XP_010039804	0.1	-	56	35.4242	44
ARC_ART_c3260	207	CAN59721	0.0	-	55	38.1206	49
ARC_ART_c3261	124	XP_002540139	0.0	-	65	41.2022	40
ARC_ART_c3263	223	AGZ19352	0.0	-	70	51.9878	48
ARC_ART_c3264	206	KEH38046	0.9	-	54	31.9574	50
ARC_ART_c3265	154	XP_001786668	0.0	-	77	46.9802	35
ARC_ART_c3267	114	CEF98003	0.0	-	80	39.6614	35
ARC_ART_c3277	163	XP_003078364	0.0	GO:0005488	60	47.3654	51
ARC_ART_c3282	144	XP_011016735	0.0	GO:0044763	81	62.003	44
ARC_ART_c3283	152	KJB47085	0.0	GO:0044249	78	63.5438	50
ARC_ART_c3286	228	XP_004953073	0.4	-	51	34.2686	35
ARC_ART_c3292	163	XP_011079635	0.2	-	64	33.4982	34
ARC_ART_c3295	160	BAJ94909	0.0	-	71	56.6102	38
ARC_ART_c3306	117	NP_194843	0.4	-	54	33.4982	35
ARC_ART_c3322	144	XP_002315097	0.0	GO:0019252	80	45.8246	35
ARC_ART_c3326	219	EXB96395	0.0	-	51	40.0466	62
ARC_ART_c3331	197	XP_009610464	0.0	-	61	46.2098	65
ARC_ART_c3338	140	CBI36950	0.7	-	55	33.113	43
ARC_ART_c3339	118	XP_001786692	0.0	GO:0006099	79	53.9138	39
ARC_ART_c3357	247	CCO17520	0.2	-	47	35.4242	59
ARC_ART_c3360	128	NP_683783	0.0	GO:0016491	78	46.595	38

ARC_ART_c3365	136	XP_002539100	0.0	GO:2001289	84	67.0106	44
ARC_ART_c3367	108	EEC76774	0.0	-	71	38.1206	35
ARC_ART_c3369	308	XP_002539014	0.0	GO:0008410	84	104.76	75
ARC_ART_c3372	177	XP_002535010	0.0	-	67	36.965	37
ARC_ART_c3376	118	AIU50686	0.0	-	63	41.5874	36
ARC_ART_c3379	103	KIY94060	0.0	-	66	38.891	36
ARC_ART_c3383	137	AID67469	0.0	GO:0005840	84	71.2478	45
ARC_ART_c3387	164	XP_009369477	0.1	-	61	34.6538	36
ARC_ART_c3398	152	XP_009386466	0.4	-	63	33.8834	38
ARC_ART_c3399	270	KFK42289	0.0	GO:0055114	82	118.627	73
ARC_ART_c3404	260	XP_001689951	0.0	GO:0042128	75	79.337	58
ARC_ART_c3409	121	XP_009388207	0.0	-	54	36.1946	37
ARC_ART_c3410	233	EPS70026	0.0	-	75	45.0542	37
ARC_ART_c3412	167	XP_009351046	0.0	GO:0003676	83	85.1149	55
ARC_ART_c3419	213	KIZ02834	0.0	GO:0016620	69	54.299	49
ARC_ART_c3423	162	XP_003057294	0.0	GO:0009051	77	55.8398	45
ARC_ART_c3427	111	KDD76334	0.0	GO:0003824	71	46.2098	35
ARC_ART_c3430	148	XP_002536270	0.0	-	56	38.1206	48
ARC_ART_c3465	220	O22553	0.0	-	53	60.077	71
ARC_ART_c3471	101	XP_001420812	0.0	-	63	37.7354	36
ARC_ART_c3490	194	XP_006647172	0.0	GO:0071704	66	54.6842	63
ARC_ART_c3495	202	XP_005850926	0.0	GO:0016787	57	46.2098	69
ARC_ART_c3499	112	EDQ48391	0.0	GO:0046872	91	63.929	37
ARC_ART_c3515	160	XP_005644625	0.0	GO:0016772	68	53.1434	48
ARC_ART_c3516	142	ACJ83969	0.4	-	62	31.5722	37
ARC_ART_c3527	143	CBY94069	0.0	GO:0044763	61	41.2022	34
ARC_ART_c3532	142	CDY20229	0.0	-	63	48.521	46
ARC_ART_c3541	201	XP_007159041	0.0	-	64	39.2762	42
ARC_ART_c3555	153	XP_002539320	0.0	GO:0006633	84	82.0333	50

ARC_ART_c3557	136	CCO15537	0.0	GO:0005975	72	65.855	44
ARC_ART_c3562	130	XP_002971538	0.2	-	55	33.4982	38
ARC_ART_c3573	186	XP_010420529	0.8	-	54	33.113	42
ARC_ART_c3579	120	XP_002502651	0.5	-	55	32.7278	34
ARC_ART_c3584	115	BAK01187	0.1	-	62	35.4242	35
ARC_ART_c3587	147	XP_002536402	0.0	GO:0007165	68	50.8322	48
ARC_ART_c3590	128	XP_001694415	0.0	GO:0005488	62	45.4394	40
ARC_ART_c3593	109	XP_003543750	0.1	-	66	35.039	36
ARC_ART_c3594	202	XP_002539047	0.0	GO:0008152	81	67.781	48
ARC_ART_c3598	116	XP_002540227	0.0	GO:0009166	97	75.8702	38
ARC_ART_c3613	120	KFM24979	0.0	GO:0000166	75	49.6766	40
ARC_ART_c3617	213	CDM82334	0.0	-	59	58.9214	64
ARC_ART_c3620	246	XP_002507787	0.0	GO:0003824	50	55.8398	80
ARC_ART_c3625	120	CEF97651	0.0	GO:0004017	82	46.9802	34
ARC_ART_c3626	283	YP_001152205	0.0	GO:0044444	70	60.8474	48
ARC_ART_c3627	129	XP_001417060	0.4	-	57	33.4982	42
ARC_ART_c3631	125	XP_007022789	0.0	GO:0006541	83	63.1586	36
ARC_ART_c3632	152	XP_001690386	1.0	-	59	32.7278	44
ARC_ART_c3635	105	XP_002464917	0.0	-	64	38.1206	34
ARC_ART_c3638	150	KDO38964	0.5	-	70	33.4982	34
ARC_ART_c3640	196	EDQ48527	0.0	-	63	45.4394	41
ARC_ART_c3647	112	XP_005843161	0.0	GO:0044237	77	45.0542	36
ARC_ART_c3662	143	XP_001700288	0.2	-	63	34.2686	41
ARC_ART_c3674	149	XP_001752830	0.0	GO:0044763	66	53.9138	48
ARC_ART_c3682	232	XP_009350073	0.0	-	64	67.3958	76
ARC_ART_c3697	224	XP_002538106	0.0	GO:0044763	75	88.9669	68
ARC_ART_c3711	123	XP_002537571	0.0	-	66	39.6614	36

ARC_ART_c3720	113	XP_002539330	0.0	-	89	64.6994	37
ARC_ART_c3732	155	EEE52320	0.0	GO:0019752	73	52.7582	38
ARC_ART_c3747	171	XP_002535741	0.0	GO:0050896	70	57.3806	57
ARC_ART_c3754	195	BAK01006	0.3	-	57	34.2686	35
ARC_ART_c3759	123	KDP33603	0.0	-	56	40.4318	39
ARC_ART_c3760	125	XP_002535467	0.0	-	64	48.1358	39
ARC_ART_c3763	115	XP_002536194	0.1	-	60	35.039	35
ARC_ART_c3765	179	KIZ03597	0.7	-	48	33.113	52
ARC_ART_c3768	246	XP_002535748	0.0	GO:0016740	61	68.5514	75
ARC_ART_c3775	141	XP_003058426	0.1	-	68	35.039	41
ARC_ART_c3787	134	XP_008797307	0.0	-	54	36.965	48
ARC_ART_c3795	179	EAY73221	0.0	-	82	41.5874	34
ARC_ART_c3797	267	XP_002455453	0.0	GO:0051287	85	77.7962	47
ARC_ART_c3818	150	EEC70905	0.0	GO:0003824	60	46.595	46
ARC_ART_c3819	159	XP_011020246	1.0	-	41	32.7278	46
ARC_ART_c3829	130	XP_002536193	0.0	GO:0015416	92	80.8777	42
ARC_ART_c3842	165	XP_006289875	0.8	-	53	32.7278	39
ARC_ART_c3844	233	EDQ48139	0.0	-	61	42.3578	47
ARC_ART_c3850	187	XP_001703480	0.0	-	66	43.5134	51
ARC_ART_c3855	154	XP_007133271	0.3	-	52	34.2686	44
ARC_ART_c3857	128	EEC76122	0.0	GO:0050660	87	63.929	39
ARC_ART_c3864	232	EEC81323	0.0	-	61	53.9138	71
ARC_ART_c3867	140	NP_001176741	0.2	-	50	33.113	36
ARC_ART_c3876	211	EEE50471	0.0	GO:0005840	98	93.9745	70
ARC_ART_c3886	145	CDY18677	0.0	GO:0008270	91	79.7221	48
ARC_ART_c3900	114	ACU18628	0.0	-	58	41.2022	34

ARC_ART_c3912	130	EPS70023	0.0	-	64	45.4394	39
ARC_ART_c3922	164	KFM27779	0.0	GO:0044464	68	48.1358	47
ARC_ART_c3944	141	XP_001786938	0.0	-	65	40.0466	40
ARC_ART_c3945	132	YP_001152206	0.0	-	62	38.1206	40
ARC_ART_c3950	198	XP_003599577	0.0	-	53	51.2174	63
ARC_ART_c3951	254	XP_002535710	0.0	-	65	39.2762	41
ARC_ART_c3955	167	YP_001312258	0.0	-	60	43.8986	55
ARC_ART_c3960	245	EPS74492	0.0	-	50	37.3502	40
ARC_ART_c3965	118	XP_005848116	0.0	-	69	39.2762	39
ARC_ART_c3991	162	XP_002504041	0.0	-	65	39.6614	35
ARC_ART_c3997	151	XP_005651257	0.0	GO:0009941	73	51.9878	42
ARC_ART_c3998	202	KJB76688	0.0	-	52	38.891	46
ARC_ART_c4012	140	KFM27986	0.0	-	53	38.891	47
ARC_ART_c4020	121	KEH15442	0.0	GO:0016021	87	58.9214	39
ARC_ART_c4032	170	XP_002958772	0.0	-	57	38.1206	54
ARC_ART_c4034	188	XP_011016758	0.0	-	66	58.9214	50
ARC_ART_c4035	134	XP_002535279	0.0	-	55	42.3578	36
ARC_ART_c4036	120	ACO50733	0.0	GO:0016021	81	54.299	37
ARC_ART_c4037	147	EEC78702	0.0	GO:0016787	70	51.2174	48
ARC_ART_c4038	125	EDQ48391	0.0	GO:0043229	90	73.559	41
ARC_ART_c4040	188	CCO14338	0.0	GO:0004427	83	58.151	36
ARC_ART_c4042	140	KEH15203	0.2	-	56	34.6538	44
ARC_ART_c4045	144	KDD75324	0.0	-	68	45.8246	41
ARC_ART_c4052	228	XP_002538450	0.1	-	52	35.8094	53
ARC_ART_c4054	137	EEC81324	0.0	GO:0044699	71	45.8246	38
ARC_ART_c4055	217	XP_002538077	0.0	-	62	42.3578	48
ARC_ART_c4062	259	XP_004144747	0.3	-	45	35.039	51
ARC_ART_c4065	255	XP_002536366	0.0	-	74	85.1149	66

ARC_ART_c4066	145	XP_011016369	0.0	GO:0046872	79	62.003	44
ARC_ART_c4068	103	XP_007039834	0.0	GO:0000166	73	53.1434	34
ARC_ART_c4071	111	XP_005850869	0.0	GO:0019752	77	49.2914	35
ARC_ART_c4081	204	XP_011090171	0.5	-	51	33.4982	52
ARC_ART_c4082	157	XP_002535455	0.0	GO:0046872	87	71.2478	49
ARC_ART_c4088	164	XP_010232200	0.3	-	57	34.2686	52
ARC_ART_c4091	128	XP_002535966	0.0	GO:0016020	85	58.151	40
ARC_ART_c4096	143	XP_007146919	0.7	-	53	32.7278	45
ARC_ART_c4104	188	XP_006487272	0.9	-	48	33.113	68
ARC_ART_c4112	203	EEE67871	0.0	-	52	35.039	44
ARC_ART_c4115	180	XP_009386082	0.0	GO:0004832	80	62.3882	41
ARC_ART_c4126	160	XP_002537497	0.0	-	86	89.7373	52
ARC_ART_c4129	117	CEF97097	0.0	-	57	37.7354	45
ARC_ART_c4142	132	KDP21008	0.0	GO:0016740	64	50.447	34
ARC_ART_c4146	178	EEC68227	0.0	GO:0008270	94	116.316	59
ARC_ART_c4148	124	XP_003055617	0.8	-	65	32.7278	35
ARC_ART_c4155	121	XP_010500620	0.0	GO:0005777	95	72.7886	40
ARC_ART_c4159	149	XP_008392351	0.7	-	53	32.7278	45
ARC_ART_c4163	240	XP_008349783	0.0	GO:0004725	75	60.8474	41
ARC_ART_c4165	104	XP_005647214	0.0	-	70	37.7354	34
ARC_ART_c4167	136	ERN17387	0.7	-	50	31.5722	40
ARC_ART_c4173	161	EEE52320	0.0	-	78	41.2022	41

ARC_ART_c4179	117	XP_008775174	0.3	-	65	33.8834	38
ARC_ART_c4181	103	XP_002538609	0.0	GO:0003824	70	46.595	34
ARC_ART_c4192	129	XP_008352655	0.0	GO:0046872	68	45.8246	38
ARC_ART_c4193	192	XP_011032902	0.7	-	58	33.113	34
ARC_ART_c4201	160	XP_004495585	0.8	-	55	32.7278	40
ARC_ART_c4205	119	XP_004253462	0.0	GO:0008270	79	58.9214	39
ARC_ART_c4210	158	XP_010922444	0.7	-	64	33.113	42
ARC_ART_c4227	175	XP_002984874	0.9	-	55	32.7278	36
ARC_ART_c4230	195	XP_003055243	0.0	GO:0016740	68	68.5514	64
ARC_ART_c4232	152	CAJ86404	0.5	-	54	33.4982	35
ARC_ART_c4240	197	XP_003588326	0.0	-	70	50.0618	40
ARC_ART_c4252	158	EMS47856	0.4	-	58	33.4982	41
ARC_ART_c4253	166	XP_011016759	0.0	-	66	39.6614	50
ARC_ART_c4260	161	XP_003614396	0.0	-	71	47.7506	38
ARC_ART_c4269	147	BAF79974	0.5	-	53	33.4982	41
ARC_ART_c4270	329	XP_011016126	0.0	GO:0005525	88	88.9669	51
ARC_ART_c4272	117	XP_004143369	0.0	GO:0043168	68	52.373	38
ARC_ART_c4273	111	XP_002535893	0.0	-	72	37.3502	37
ARC_ART_c4275	125	XP_002535367	0.1	-	61	35.8094	39
ARC_ART_c4289	127	XP_008354665	0.0	-	62	36.5798	37
ARC_ART_c4292	190	EEE52320	0.0	GO:0072330	100	123.25	63
ARC_ART_c4293	169	XP_007211772	0.0	-	57	37.3502	47
ARC_ART_c4309	142	XP_002536295	0.0	-	62	41.2022	37
ARC_ART_c4312	126	XP_002534913	0.0	GO:0009236	87	73.9442	41

ARC_ART_c4315	140	XP_005646891	0.0	GO:0044249	63	43.1282	47
ARC_ART_c4318	110	XP_002535209	0.5	-	61	32.7278	36
ARC_ART_c4324	121	EEC69551	0.2	-	55	34.2686	34
ARC_ART_c4325	123	XP_001786506	0.0	-	56	36.1946	39
ARC_ART_c4334	110	EMS63913	0.0	-	65	34.2686	35
ARC_ART_c4337	163	XP_002520895	0.0	GO:0019752	65	60.077	52
ARC_ART_c4340	149	XP_011096675	0.7	-	57	32.7278	38
ARC_ART_c4354	247	XP_002537813	0.0	-	48	39.6614	80
ARC_ART_c4361	147	XP_004253340	0.0	-	63	41.5874	38
ARC_ART_c4364	111	XP_002536189	0.0	GO:0051287	83	59.6918	36
ARC_ART_c4365	161	XP_005645798	0.0	GO:0008652	63	50.447	52
ARC_ART_c4374	198	XP_002537821	0.0	-	78	75.485	65
ARC_ART_c4388	376	YP_009104960	0.0	GO:0003746	79	166.007	120
ARC_ART_c4389	124	XP_002536583	0.0	-	69	45.8246	39
ARC_ART_c4395	138	CEF99477	0.0	GO:0005960	67	53.9138	46
ARC_ART_c4396	163	AFK36151	0.0	-	56	36.1946	50
ARC_ART_c4418	125	EEC77966	0.0	-	73	41.2022	38
ARC_ART_c4424	132	XP_002536830	0.2	-	62	34.6538	35
ARC_ART_c4427	136	XP_003057676	0.0	-	64	37.3502	37
ARC_ART_c4439	165	XP_005850825	0.0	GO:0046872	76	77.7962	55
ARC_ART_c4441	122	XP_008787396	0.1	-	48	35.4242	43
ARC_ART_c4442	191	KEH16995	0.0	-	61	56.9954	60
ARC_ART_c4448	132	XP_011016759	0.0	-	71	54.6842	39
ARC_ART_c4455	147	AFW62221	0.3	-	44	32.7278	52
ARC_ART_c4458	114	XP_002537914	0.0	-	83	49.6766	36
ARC_ART_c4467	145	KFM28844	0.0	GO:0046872	93	93.2041	48
ARC_ART_c4481	107	XP_002538804	0.0	-	71	40.817	35
ARC_ART_c4482	114	XP_002537898	0.0	GO:0008152	78	44.669	37

ARC_ART_c4485	165	XP_008800618	0.0	-	65	36.5798	41
ARC_ART_c4488	122	XP_003627732	0.0	-	80	50.447	40
ARC_ART_c4495	229	KFM25320	0.0	-	61	41.9726	49
ARC_ART_c4496	154	AFG58832	0.0	-	63	41.2022	47
ARC_ART_c4501	217	XP_002535681	0.0	GO:0005975	66	69.707	71
ARC_ART_c4502	228	ERN16355	0.3	-	50	34.2686	62
ARC_ART_c4521	249	XP_003588337	0.0	-	58	53.9138	55
ARC_ART_c4527	165	XP_002534902	0.0	-	69	58.151	52
ARC_ART_c4533	129	XP_005848245	0.0	GO:0005975	82	60.4622	40
ARC_ART_c4543	142	KDP26073	0.1	-	61	35.8094	44
ARC_ART_c4551	172	XP_001753431	0.0	-	69	51.2174	56
ARC_ART_c4557	123	XP_008462105	0.0	GO:0006435	79	63.1586	39
ARC_ART_c4562	250	CDY67756	0.0	GO:0005975	75	56.9954	41
ARC_ART_c4571	140	KEH15190	0.1	-	67	34.2686	37
ARC_ART_c4572	152	XP_002507775	0.0	-	63	41.2022	41
ARC_ART_c4582	145	EEE60756	0.0	-	53	42.3578	47
ARC_ART_c4584	116	XP_001765953	0.0	-	73	36.1946	38
ARC_ART_c4595	123	XP_011082719	0.0	GO:0005774	88	55.4546	36
ARC_ART_c4598	126	DAA62915	0.1	-	49	34.6538	57
ARC_ART_c4604	133	KEH15234	0.0	GO:0006096	95	72.4034	40
ARC_ART_c4608	150	XP_008438944	0.9	-	60	32.7278	41
ARC_ART_c4610	147	KDD74771	0.0	GO:0006779	76	50.8322	34
ARC_ART_c4611	142	XP_002536310	0.2	-	64	33.4982	34

ARC_ART_c4612	108	XP_009420476	0.0	-	67	39.2762	37
ARC_ART_c4613	107	XP_008802677	0.0	-	68	45.0542	35
ARC_ART_c4614	116	XP_002536424	0.0	-	67	43.1282	37
ARC_ART_c4617	124	XP_005650660	0.1	-	54	35.039	37
ARC_ART_c4618	181	CCO17293	0.0	-	58	38.5058	48
ARC_ART_c4627	192	XP_009107608	0.5	-	50	33.8834	55
ARC_ART_c4629	224	XP_002870879	0.5	-	54	31.5722	44
ARC_ART_c4636	136	XP_001786892	0.0	-	63	40.817	44
ARC_ART_c4646	113	XP_002539604	0.3	-	78	31.9574	37
ARC_ART_c4651	126	XP_007025224	0.9	-	64	30.4166	39
ARC_ART_c4655	113	XP_002956664	0.1	-	60	35.039	35
ARC_ART_c4659	157	EYU40811	0.5	-	56	33.4982	41
ARC_ART_c4662	128	XP_002987395	0.0	GO:0009295	92	70.8626	42
ARC_ART_c4666	173	AAF16526	0.0	GO:0044763	75	68.9366	49
ARC_ART_c4687	130	EMS53534	1.0	-	52	32.3426	38
ARC_ART_c4688	115	KJB69893	0.6	-	64	32.7278	39
ARC_ART_c4690	222	ERN14206	0.4	-	57	32.3426	40
ARC_ART_c4697	108	EMT00280	0.0	GO:0004019	88	60.4622	36
ARC_ART_c4700	185	XP_003610227	0.0	-	59	42.743	42
ARC_ART_c4702	155	NP_001236697	0.6	-	58	31.187	39
ARC_ART_c4713	145	XP_002509410	0.2	-	76	35.039	34
ARC_ART_c4730	139	XP_002535112	0.0	GO:0006810	71	58.5362	45
ARC_ART_c4735	271	XP_010467345	0.0	GO:0005829	65	86.2705	80
ARC_ART_c4737	168	BAK02939	0.0	-	66	39.6614	36
ARC_ART_c4740	127	EDQ48640	0.0	GO:0000166	72	43.5134	37
ARC_ART_c4743	257	YP_001312258	0.9	-	53	31.5722	39
ARC_ART_c4747	113	XP_002536543	0.0	GO:0004872	85	56.6102	35

ARC_ART_c4759	163	XP_011079838	0.8	-	65	32.7278	38
ARC_ART_c4762	183	EPS63983	0.0	GO:0000154	59	47.7506	61
ARC_ART_c4775	198	XP_003532633	0.2	-	59	34.2686	42
ARC_ART_c4779	113	XP_001786407	0.2	-	67	33.8834	34
ARC_ART_c4788	114	BAH23787	0.1	-	59	35.4242	37
ARC_ART_c4795	184	XP_002960000	0.0	GO:0009058	92	93.9745	52
ARC_ART_c4801	185	XP_002536967	0.0	GO:0000155	90	73.1738	61
ARC_ART_c4814	331	KFK30161	0.0	-	45	38.891	74
ARC_ART_c4815	268	XP_011468909	0.2	-	46	35.8094	47
ARC_ART_c4820	136	XP_002537624	0.0	GO:0009423	91	63.5438	34
ARC_ART_c4828	237	XP_011459240	0.8	-	50	33.4982	46
ARC_ART_c4845	127	XP_002503749	0.0	GO:0016021	75	46.2098	41
ARC_ART_c4850	147	XP_002534817	0.0	-	81	72.7886	48
ARC_ART_c4856	130	XP_002534985	0.0	-	61	54.6842	42
ARC_ART_c4857	136	XP_010028657	0.0	-	54	36.965	42
ARC_ART_c4868	120	XP_002957101	0.1	-	63	35.4242	38
ARC_ART_c4871	186	AGP25716	0.0	GO:0016620	70	70.8626	61
ARC_ART_c4879	242	CCO18841	0.0	GO:0016874	68	96.6709	80
ARC_ART_c4889	153	XP_009794639	0.0	-	70	36.965	34
ARC_ART_c4892	142	XP_011015413	0.0	-	94	73.559	38
ARC_ART_c4897	107	AES59867	0.1	-	62	35.4242	35
ARC_ART_c4905	110	XP_002539044	0.0	-	70	36.1946	34
ARC_ART_c4918	164	KFM27674	0.0	GO:0055085	72	60.4622	48
ARC_ART_c4928	221	XP_002501661	0.0	GO:0019752	50	50.447	80
ARC_ART_c4941	175	XP_004952422	0.3	-	52	34.2686	40
ARC_ART_c4950	207	CDX76650	0.9	-	44	32.3426	59
ARC_ART_c4953	229	XP_007150773	0.0	-	63	38.891	41

ARC_ART_c4960	144	EEE68528	0.0	GO:0055085	89	71.633	39
ARC_ART_c4963	233	KDD73662	0.0	GO:0004658	88	132.88	77
ARC_ART_c4967	196	XP_002536010	0.0	-	65	44.2838	40
ARC_ART_c4968	190	XP_008792966	0.0	GO:0005516	83	52.373	37
ARC_ART_c4973	258	EPS71500	0.2	-	50	35.4242	61
ARC_ART_c4978	133	XP_008807202	0.6	-	52	33.113	50
ARC_ART_c4985	157	XP_001699496	0.0	-	56	38.891	50
ARC_ART_c4988	150	XP_001786594	0.0	GO:0016810	72	43.1282	36
ARC_ART_c4996	135	EEC68421	0.0	GO:0048608	100	93.5893	44
ARC_ART_c5002	202	XP_009393916	0.2	-	52	35.4242	53
ARC_ART_c5010	171	XP_001770207	0.0	GO:0009295	77	82.8037	57
ARC_ART_c5017	172	XP_009149660	0.0	GO:0009570	63	60.4622	57
ARC_ART_c5019	185	XP_009350805	0.0	-	72	80.4925	55
ARC_ART_c5021	157	EEC68416	0.0	GO:0006810	61	51.9878	52
ARC_ART_c5026	112	XP_010270357	0.1	-	72	35.039	36
ARC_ART_c5027	215	AAG51335	0.4	-	50	34.2686	58
ARC_ART_c5029	209	XP_001695504	0.4	-	48	33.8834	58
ARC_ART_c5030	240	XP_002535792	0.0	GO:0003824	66	52.7582	48
ARC_ART_c5041	136	XP_002536314	0.0	-	57	40.817	45
ARC_ART_c5049	202	KDD72606	0.0	GO:0006810	60	69.3218	66
ARC_ART_c5061	220	EAY93455	0.0	-	60	38.891	58
ARC_ART_c5063	210	ABH09321	0.0	-	55	36.5798	45
ARC_ART_c5070	146	XP_002534902	0.0	-	85	71.2478	42
ARC_ART_c5075	111	KIZ04736	0.0	-	85	59.3066	35
ARC_ART_c5091	182	XP_002534980	0.0	-	55	37.7354	58

ARC_ART_c5099	115	XP_002539357	0.0	GO:0004067	94	68.5514	37
ARC_ART_c5108	154	XP_009350068	0.0	GO:0003723	80	67.781	50
ARC_ART_c5115	117	XP_002536034	0.0	-	91	61.2326	35
ARC_ART_c5118	125	XP_006356858	0.9	-	67	32.3426	40
ARC_ART_c5120	337	ABR26094	0.0	-	55	40.4318	76
ARC_ART_c5125	197	XP_001767396	0.0	GO:0003824	62	44.669	54
ARC_ART_c5127	124	XP_006413903	0.0	GO:0047652	67	45.4394	40
ARC_ART_c5133	133	KJB26907	0.0	GO:0008233	90	65.4698	42
ARC_ART_c5137	310	XP_003084425	0.0	-	56	41.9726	65
ARC_ART_c5139	166	EEC77997	0.0	GO:0055114	94	73.559	37
ARC_ART_c5145	105	XP_009350055	0.0	-	100	78.1814	34
ARC_ART_c5146	146	XP_002539587	0.0	-	68	38.5058	45
ARC_ART_c5147	121	XP_002536053	0.0	-	55	39.2762	40
ARC_ART_c5149	137	XP_006382153	0.8	-	65	32.7278	38
ARC_ART_c5159	130	XP_009350075	0.0	GO:0044249	95	79.337	43
ARC_ART_c5174	144	XP_005644205	0.0	GO:0044267	62	49.6766	48
ARC_ART_c5175	126	XP_009382921	0.1	-	70	35.039	40
ARC_ART_c5177	127	EEC76404	0.0	GO:0016020	100	88.9669	42
ARC_ART_c5186	109	XP_005646219	0.0	GO:0005829	77	53.5286	35
ARC_ART_c5190	218	XP_002536159	0.0	-	65	42.3578	47
ARC_ART_c5197	141	EYU41355	0.4	-	60	33.113	38
ARC_ART_c5199	263	KCW90033	0.0	GO:0006261	76	77.7962	72
ARC_ART_c5214	161	XP_001786560	0.0	GO:0006289	88	68.1662	45
ARC_ART_c5216	147	XP_011016368	0.0	-	64	39.6614	45
ARC_ART_c5219	121	XP_002535893	0.0	-	69	45.0542	36

ARC_ART_c5230	251	XP_002538597	0.0	GO:0044763	56	53.9138	64
ARC_ART_c5236	135	P53385	0.0	GO:0019557	84	76.2554	45
ARC_ART_c5245	183	XP_002537329	0.0	-	86	97.4413	60
ARC_ART_c5248	154	XP_010462967	0.4	-	50	33.4982	46
ARC_ART_c5251	109	XP_005846913	0.0	-	73	40.4318	34
ARC_ART_c5261	161	XP_002538477	0.0	GO:0008152	81	77.7962	49
ARC_ART_c5271	211	ACO50740	0.0	GO:0055114	75	69.707	62
ARC_ART_c5272	131	XP_003064277	0.0	GO:0044763	68	47.3654	45
ARC_ART_c5273	205	XP_002439635	0.3	-	48	32.3426	54
ARC_ART_c5277	186	XP_002538475	0.0	GO:0055085	65	62.3882	58
ARC_ART_c5280	161	XP_002307204	0.0	GO:0030170	85	56.9954	35
ARC_ART_c5287	276	XP_009350076	0.0	GO:0046914	85	107.842	60
ARC_ART_c5288	207	XP_009366572	0.0	-	66	73.9442	57
ARC_ART_c5295	126	XP_002960003	0.0	GO:0005739	71	53.1434	42
ARC_ART_c5301	164	XP_002535367	0.0	-	63	53.9138	52
ARC_ART_c5305	104	XP_002537034	0.0	-	88	65.4698	34
ARC_ART_c5306	127	XP_002948465	0.0	-	66	39.6614	42
ARC_ART_c5325	174	AGB85039	0.0	GO:0016020	86	78.9518	50
ARC_ART_c5326	118	XP_002960004	0.2	-	64	34.2686	34
ARC_ART_c5329	135	XP_007222113	0.9	-	63	32.3426	38
ARC_ART_c5331	173	KEH15587	0.0	GO:0005975	65	58.5362	49
ARC_ART_c5332	137	XP_002535563	0.0	GO:0050580	81	65.0846	44
ARC_ART_c5333	127	EEE67873	0.0	-	67	42.743	40
ARC_ART_c5337	195	XP_002959981	0.0	GO:0016740	69	66.2402	65
ARC_ART_c5340	150	XP_010319181	0.0	GO:0044763	76	43.5134	39
ARC_ART_c5341	111	XP_001778144	0.0	-	68	40.0466	35
ARC_ART_c5343	110	EAY93131	0.0	-	69	48.1358	36
ARC_ART_c5356	138	EEC83292	0.0	GO:0016021	86	69.3218	45

ARC_ART_c5357	144	XP_011075876	0.0	-	65	49.2914	44
ARC_ART_c5359	108	XP_006664909	0.2	-	60	34.2686	35
ARC_ART_c5361	122	AGR67375	0.0	GO:0048027	87	68.1662	40
ARC_ART_c5364	116	XP_002537471	0.0	-	61	38.5058	36
ARC_ART_c5367	324	XP_007132547	0.0	-	62	80.8777	90
ARC_ART_c5375	165	XP_002535103	0.0	GO:0008236	74	45.0542	39
ARC_ART_c5384	153	EEC77111	0.2	-	68	35.4242	41
ARC_ART_c5388	145	CCO15215	0.0	GO:0016833	74	43.8986	39
ARC_ART_c5394	283	XP_002534961	0.0	-	56	39.6614	87
ARC_ART_c5398	236	XP_007144259	0.9	-	53	33.113	39
ARC_ART_c5402	168	ACU14280	0.3	-	65	33.4982	38
ARC_ART_c5405	150	XP_002500036	0.0	GO:0017111	79	48.9062	44
ARC_ART_c5411	152	XP_001753431	0.0	-	58	52.7582	50
ARC_ART_c5412	138	BAI81979	0.0	GO:0006952	78	56.6102	41
ARC_ART_c5421	134	XP_010315071	0.0	-	66	46.9802	42
ARC_ART_c5426	151	XP_004289772	0.2	-	57	34.6538	45
ARC_ART_c5438	133	NP_683774	0.0	GO:0003899	92	74.7146	40
ARC_ART_c5439	111	XP_002537026	0.0	GO:0004871	75	49.6766	36
ARC_ART_c5447	132	XP_005847017	0.0	GO:0003935	80	58.9214	41
ARC_ART_c5452	121	XP_001701470	0.0	GO:0005525	78	50.8322	38
ARC_ART_c5453	118	XP_002536193	0.0	-	61	37.3502	39
ARC_ART_c5454	288	XP_007099554	0.3	-	52	35.4242	72
ARC_ART_c5457	196	XP_001780312	0.1	-	64	35.8094	39
ARC_ART_c5458	127	XP_002269951	0.8	-	56	32.3426	37
ARC_ART_c5477	177	CCO15102	0.0	GO:0005874	86	58.5362	58
ARC_ART_c5485	127	EEC80862	0.0	GO:0006184	94	70.0922	37
ARC_ART_c5486	120	XP_001781947	1.0	-	47	31.9574	34
ARC_ART_c5504	173	CBI28473	0.0	-	55	37.7354	40
ARC_ART_c5506	165	EEC79608	0.3	-	53	33.8834	39

ARC_ART_c5508	113	EEC80776	0.0	GO:0055085	83	54.299	37
ARC_ART_c5529	117	ACN40061	0.5	-	63	33.113	36
ARC_ART_c5533	103	KIZ05750	0.0	-	70	40.0466	34
ARC_ART_c5542	120	AAG41902	0.0	GO:0015419	92	57.7658	39
ARC_ART_c5552	137	XP_008450745	0.4	-	47	33.4982	40
ARC_ART_c5554	119	XP_004976016	0.1	-	56	35.8094	39
ARC_ART_c5555	176	ABA98150	0.6	-	54	33.4982	55
ARC_ART_c5559	201	EMT18568	0.0	GO:0044763	56	55.8398	65
ARC_ART_c5562	106	XP_001422565	0.2	-	68	33.8834	35
ARC_ART_c5563	129	ADU04388	0.0	GO:0005737	91	56.6102	34
ARC_ART_c5564	265	XP_005651032	0.0	GO:0006259	72	103.99	88
ARC_ART_c5565	231	XP_010919735	0.7	-	61	33.4982	42
ARC_ART_c5569	160	XP_003616487	0.4	-	62	33.8834	37
ARC_ART_c5587	217	EYU31197	0.0	GO:0004089	71	51.9878	42
ARC_ART_c5598	123	EAY93131	0.0	GO:0019829	84	53.1434	38
ARC_ART_c5608	215	XP_002979867	0.0	GO:0003676	72	72.0182	61
ARC_ART_c5620	185	XP_002537954	0.0	GO:0016747	79	63.1586	48
ARC_ART_c5623	139	XP_002537108	0.0	-	85	68.9366	42
ARC_ART_c5626	127	XP_001765721	0.0	-	62	38.5058	40
ARC_ART_c5635	175	XP_002535988	0.0	-	55	36.965	56
ARC_ART_c5642	142	CCO19465	0.0	-	55	37.3502	45
ARC_ART_c5643	130	AHN93011	0.0	GO:0030170	83	64.6994	43
ARC_ART_c5654	115	XP_001759947	0.0	-	64	38.5058	37
ARC_ART_c5674	273	EMT00280	0.0	GO:0006164	71	112.849	92
ARC_ART_c5679	185	XP_009350069	0.0	GO:0003723	78	60.077	41
ARC_ART_c5687	144	XP_002539604	0.0	-	65	41.2022	41
ARC_ART_c5694	157	KFM28560	0.0	GO:0008152	76	59.6918	47
ARC_ART_c5699	111	EEC76122	0.0	GO:1901363	64	43.1282	37
ARC_ART_c5704	107	AEY83985	0.0	GO:0019538	94	59.3066	34

ARC_ART_c5711	114	EMT33284	0.9	-	52	32.3426	36
ARC_ART_c5726	158	EEC79944	0.0	GO:0032991	76	53.5286	42
ARC_ART_c5732	140	EMS45415	0.6	-	55	33.113	34
ARC_ART_c5736	142	EEC76122	0.0	GO:0050660	80	56.9954	40
ARC_ART_c5740	137	AJK93569	0.0	GO:0044710	77	51.2174	35
ARC_ART_c5757	161	KCW83605	0.2	-	56	33.8834	41
ARC_ART_c5761	165	AHB17747	0.2	-	50	35.039	46
ARC_ART_c5762	152	AAM65185	0.8	-	55	32.7278	43
ARC_ART_c5770	108	XP_002536310	0.0	-	61	40.4318	36
ARC_ART_c5771	135	XP_006493801	0.1	-	57	35.039	35
ARC_ART_c5772	138	XP_002500461	0.0	-	70	36.1946	37
ARC_ART_c5773	148	XP_002502894	0.0	-	58	37.3502	34
ARC_ART_c5777	123	XP_002536160	0.9	-	55	31.9574	34
ARC_ART_c5780	131	XP_005650702	0.0	-	51	38.1206	43
ARC_ART_c5782	120	XP_002539615	0.3	-	70	33.4982	37
ARC_ART_c5785	104	XP_005648103	0.0	-	71	35.8094	35
ARC_ART_c5786	218	XP_002539699	0.0	-	58	41.9726	63
ARC_ART_c5787	106	XP_004289516	0.0	-	70	37.7354	34
ARC_ART_c5804	120	EEC83292	0.0	GO:0046872	92	86.2705	40
ARC_ART_c5805	152	XP_002966923	0.0	GO:0016874	63	59.3066	49
ARC_ART_c5812	266	XP_002535353	0.0	-	68	88.5817	86
ARC_ART_c5814	212	XP_002951029	0.1	-	66	35.4242	39
ARC_ART_c5819	157	CCO18051	0.3	-	55	33.8834	47
ARC_ART_c5832	117	XP_002539165	0.0	-	64	39.2762	39
ARC_ART_c5833	180	XP_002540194	0.0	GO:0044763	76	76.6406	51
ARC_ART_c5838	101	XP_010911627	0.1	-	60	33.8834	41

ARC_ART_c5842	104	XP_004490739	0.0	-	72	40.0466	36
ARC_ART_c5851	113	XP_002538653	0.2	-	55	32.7278	34
ARC_ART_c5860	126	XP_002540107	0.0	GO:0004872	100	79.7221	41
ARC_ART_c5862	105	XP_002960029	0.0	GO:0016740	76	45.0542	34
ARC_ART_c5869	276	XP_002269802	0.0	-	59	41.9726	54
ARC_ART_c5871	164	CDY63598	0.0	-	69	43.1282	36
ARC_ART_c5873	204	XP_002536388	0.4	-	43	33.8834	58
ARC_ART_c5881	204	CAN77973	0.0	-	57	38.1206	54
ARC_ART_c5882	114	XP_009350805	0.0	GO:0000166	78	57.3806	37
ARC_ART_c5891	210	KDO85632	0.0	GO:0006108	81	76.2554	53
ARC_ART_c5901	261	CEF97137	0.0	GO:0008652	75	118.627	85
ARC_ART_c5905	110	XP_002536342	0.0	-	71	40.4318	35
ARC_ART_c5906	176	XP_003057732	0.0	GO:0008152	76	81.6481	55
ARC_ART_c5911	159	EEC81163	0.0	GO:0016021	97	69.3218	39
ARC_ART_c5922	152	XP_002534675	0.0	-	63	41.2022	38
ARC_ART_c5926	285	XP_002488912	0.0	GO:0006412	73	60.4622	46
ARC_ART_c5928	190	XP_003081906	0.0	GO:0005739	71	60.4622	59
ARC_ART_c5940	161	XP_002535042	0.0	GO:0008236	79	72.0182	53
ARC_ART_c5941	172	EEC76877	0.0	GO:0046961	83	80.4925	49
ARC_ART_c5942	174	XP_002535303	0.5	-	65	33.4982	35
ARC_ART_c5944	183	AIA92249	0.0	GO:0097159	67	58.151	59
ARC_ART_c5954	209	XP_003064457	0.0	GO:0019543	70	69.3218	65
ARC_ART_c5982	104	XP_003055243	0.0	GO:0030170	85	55.8398	34
ARC_ART_c5989	269	XP_009601201	0.4	-	48	34.6538	66
ARC_ART_c6005	205	XP_007008895	0.2	-	66	35.039	42

ARC_ART_c6008	135	XP_001693179	0.0	GO:0044763	78	57.7658	38
ARC_ART_c6009	108	EDQ48480	0.1	-	61	34.6538	34
ARC_ART_c6011	160	EEC76122	0.0	GO:0050660	82	78.1814	52
ARC_ART_c6019	113	EEE52320	0.0	GO:0055085	97	77.411	37
ARC_ART_c6036	178	XP_005643459	0.0	GO:0005622	79	47.7506	34
ARC_ART_c6042	209	XP_002537754	0.0	-	72	55.0694	37
ARC_ART_c6048	188	KFM27113	0.0	-	64	38.5058	34
ARC_ART_c6056	154	XP_005649243	0.0	-	73	39.6614	45
ARC_ART_c6065	120	XP_002540262	0.0	GO:0016020	73	43.5134	38
ARC_ART_c6068	135	BAK02541	0.0	GO:0008270	88	47.3654	42
ARC_ART_c6071	192	XP_008801668	0.1	-	54	35.8094	42
ARC_ART_c6081	113	ABA01126	0.0	GO:0019752	65	45.8246	35
ARC_ART_c6092	123	XP_002891090	0.1	-	67	35.039	34
ARC_ART_c6096	107	XP_002536247	0.0	-	68	37.7354	35
ARC_ART_c6098	193	XP_002536159	0.0	-	77	75.485	57
ARC_ART_c6102	166	EAZ02189	0.0	-	58	50.8322	50
ARC_ART_c6109	150	XP_002537941	0.0	GO:0016020	90	73.9442	42
ARC_ART_c6117	152	XP_008222738	0.0	-	59	43.5134	49
ARC_ART_c6121	138	CAA72795	0.0	GO:0050660	95	78.5666	45
ARC_ART_c6142	147	EXC19452	0.2	-	52	33.8834	38
ARC_ART_c6149	171	EMT02835	0.4	-	51	33.8834	54
ARC_ART_c6157	112	XP_001417046	0.0	GO:0042558	86	54.6842	37
ARC_ART_c6171	234	XP_009391403	0.4	-	39	34.6538	73
ARC_ART_c6181	168	XP_002540147	0.0	-	66	55.4546	56
ARC_ART_c6185	197	CDP02827	0.3	-	44	34.2686	70
ARC_ART_c6191	135	XP_002539047	0.0	-	66	44.2838	39
ARC_ART_c6194	115	AAV67885	0.0	GO:0004028	75	52.373	36

ARC_ART_c6195	107	XP_001701311	0.0	GO:0050660	80	51.2174	35
ARC_ART_c6204	209	EEE50470	0.0	-	68	83.5741	69
ARC_ART_c6207	107	CCO14338	0.0	GO:0044765	76	43.8986	34
ARC_ART_c6214	132	XP_002538359	0.4	-	61	33.113	36
ARC_ART_c6220	171	ERN19185	0.0	-	64	69.707	56
ARC_ART_c6232	192	AAT40303	0.5	-	52	33.4982	42
ARC_ART_c6234	166	EEC76953	0.0	GO:0003849	80	81.2629	50
ARC_ART_c6236	155	NP_001274865	0.9	-	47	32.7278	48
ARC_ART_c6239	122	XP_002537672	0.0	GO:0044780	87	69.707	40
ARC_ART_c6240	310	XP_002536446	0.0	GO:0030554	88	66.2402	51
ARC_ART_c6243	169	XP_009107428	0.9	-	52	32.7278	40
ARC_ART_c6245	167	XP_005843931	0.0	GO:0003723	85	92.8189	55
ARC_ART_c6246	176	XP_003612206	0.5	-	53	31.9574	49
ARC_ART_c6251	193	XP_003611046	0.0	-	57	38.5058	40
ARC_ART_c6257	143	XP_009350056	0.0	GO:0050896	68	51.2174	44
ARC_ART_c6262	171	XP_001787082	0.0	-	61	45.8246	60
ARC_ART_c6272	252	EEC68548	0.0	GO:0003824	67	97.0561	84
ARC_ART_c6275	258	EEC68217	0.0	GO:0016020	67	68.5514	80
ARC_ART_c6281	170	EXB95899	0.0	GO:0009630	79	70.4774	54
ARC_ART_c6285	140	NP_001056307	0.3	-	62	33.8834	35
ARC_ART_c6292	150	XP_003055972	0.0	-	56	38.1206	46
ARC_ART_c6293	191	XP_002535323	0.0	GO:0008270	94	68.1662	35
ARC_ART_c6295	146	XP_002535867	0.0	GO:0004731	97	93.2041	48
ARC_ART_c6301	190	AFW84560	0.0	-	74	58.151	43
ARC_ART_c6302	115	XP_002538125	0.0	GO:0008152	68	46.595	38
ARC_ART_c6313	183	EAZ02189	0.1	-	58	34.6538	36
ARC_ART_c6316	158	XP_002539900	0.0	-	65	49.6766	49
ARC_ART_c6321	128	XP_001417123	0.0	GO:0004834	78	60.077	42
ARC_ART_c6324	147	XP_002534966	0.0	-	68	45.4394	45

ARC_ART_c6326	144	XP_011093139	0.9	-	53	32.7278	47
ARC_ART_c6327	146	XP_002537965	0.0	-	88	60.8474	35
ARC_ART_c6332	202	CAO02550	0.0	-	64	43.5134	53
ARC_ART_c6352	122	BAJ98290	0.5	-	61	33.113	34
ARC_ART_c6353	176	XP_002534790	0.0	-	78	81.2629	57
ARC_ART_c6354	122	XP_001758472	0.0	-	50	35.8094	38
ARC_ART_c6362	149	XP_004134699	0.0	-	70	38.1206	34
ARC_ART_c6366	200	XP_001786955	0.0	GO:0009451	75	67.0106	61
ARC_ART_c6370	187	XP_002535815	0.0	GO:0032550	66	57.3806	59
ARC_ART_c6372	151	KEH15288	0.0	-	60	46.595	50
ARC_ART_c6377	155	XP_006580792	0.8	-	54	31.9574	37
ARC_ART_c6395	162	XP_002538477	0.0	GO:0008152	89	69.3218	39
ARC_ART_c6408	135	XP_003061384	0.0	-	76	51.6026	39
ARC_ART_c6412	129	XP_006828850	0.0	-	66	49.2914	42
ARC_ART_c6419	150	XP_002536800	0.0	GO:0044763	72	67.0106	47
ARC_ART_c6433	160	XP_002535693	0.3	-	61	33.8834	39
ARC_ART_c6444	171	XP_009780734	0.6	-	60	33.4982	43
ARC_ART_c6450	119	XP_003064098	0.9	-	57	31.9574	35
ARC_ART_c6474	171	EEE52320	0.0	GO:1901576	50	46.9802	56
ARC_ART_c6480	122	XP_002537063	0.0	-	74	37.3502	39
ARC_ART_c6487	141	XP_001692205	0.0	GO:0006952	75	51.2174	41
ARC_ART_c6498	156	AAQ07255	0.0	GO:0007165	66	49.2914	51
ARC_ART_c6514	135	XP_010061748	0.7	-	50	33.113	38
ARC_ART_c6516	139	YP_002808625	0.4	-	62	33.113	37
ARC_ART_c6518	180	BAJ86034	0.2	-	45	34.2686	61
ARC_ART_c6520	201	EEC83290	0.0	GO:0016021	93	92.8189	47
ARC_ART_c6521	128	EEE52324	0.0	-	87	70.0922	41

ARC_ART_c6526	135	XP_003533272	0.5	-	60	33.113	45
ARC_ART_c6531	127	XP_002535742	0.0	-	62	37.7354	37
ARC_ART_c6548	271	BAJ11784	0.0	-	66	44.2838	36
ARC_ART_c6566	123	XP_002538597	0.0	-	67	46.2098	40
ARC_ART_c6570	124	XP_002953839	0.0	-	66	44.669	45
ARC_ART_c6572	151	XP_002536966	0.1	-	51	35.039	49
ARC_ART_c6574	148	XP_002536222	0.0	GO:0006260	80	58.5362	42
ARC_ART_c6580	173	XP_002537107	0.0	-	85	48.9062	34
ARC_ART_c6589	123	XP_006606052	0.0	-	63	38.1206	44
ARC_ART_c6600	152	CAH66108	0.7	-	53	33.113	39
ARC_ART_c6603	120	XP_002536251	0.0	-	75	47.3654	36
ARC_ART_c6613	134	EEE52318	0.0	GO:0022891	100	88.5817	44
ARC_ART_c6626	148	XP_003056374	0.0	GO:0006099	90	74.3294	42
ARC_ART_c6627	174	KCW59583	0.9	-	59	31.9574	52
ARC_ART_c6630	150	XP_006418325	0.0	-	67	51.2174	49
ARC_ART_c6636	139	XP_002535223	0.0	GO:0005488	76	58.9214	43
ARC_ART_c6640	115	XP_002318162	0.0	GO:0016779	66	42.3578	36
ARC_ART_c6641	128	XP_002536059	0.0	-	65	45.4394	41
ARC_ART_c6644	116	XP_002534905	0.0	-	84	56.225	38
ARC_ART_c6646	223	KIY98372	0.0	GO:0006812	74	58.5362	54
ARC_ART_c6647	139	CCO15527	0.2	-	55	34.6538	45
ARC_ART_c6656	150	XP_002950857	0.6	-	63	32.7278	38
ARC_ART_c6657	157	XP_002279505	0.0	-	64	38.1206	48
ARC_ART_c6661	121	XP_006854446	0.0	GO:0006541	75	53.9138	36
ARC_ART_c6669	189	CDX73647	0.3	-	51	34.6538	54
ARC_ART_c6676	188	XP_001786589	0.0	-	69	51.2174	53
ARC_ART_c6679	144	ERN02829	0.5	-	52	33.113	44
ARC_ART_c6682	120	XP_001757403	0.0	GO:0015996	84	41.9726	39

ARC_ART_c6685	113	XP_010247133	0.7	-	52	32.7278	34
ARC_ART_c6691	121	YP_001382206	0.0	GO:0006412	79	47.7506	39
ARC_ART_c6695	103	KEH15313	0.4	-	70	32.3426	34
ARC_ART_c6701	141	KDD74009	0.0	GO:0005488	77	57.7658	36
ARC_ART_c6702	155	XP_002538735	0.0	-	54	39.6614	46
ARC_ART_c6713	121	XP_011014247	0.0	-	70	42.3578	40
ARC_ART_c6715	123	XP_003081309	0.0	-	71	44.669	38
ARC_ART_c6718	155	AAL57651	0.0	GO:0009072	66	43.8986	39
ARC_ART_c6723	160	XP_001786556	0.0	GO:0004129	89	75.485	49
ARC_ART_c6724	146	XP_002459967	0.0	GO:0009088	91	71.633	45
ARC_ART_c6743	143	AIY55209	0.0	GO:0044723	74	58.9214	43
ARC_ART_c6744	145	EPS66469	0.0	-	64	35.039	34
ARC_ART_c6751	126	XP_005652149	0.0	GO:0016798	69	50.447	39
ARC_ART_c6760	124	YP_006234312	0.0	-	73	43.5134	38
ARC_ART_c6764	179	KJB09764	0.0	-	52	35.8094	53
ARC_ART_c6769	122	XP_001787012	0.0	GO:0009295	70	51.6026	40
ARC_ART_c6784	179	EEC78702	0.0	GO:0044763	74	57.7658	51
ARC_ART_c6787	200	AGS12494	0.1	-	54	35.4242	57
ARC_ART_c6788	182	XP_004507174	0.0	GO:0004222	86	60.4622	38
ARC_ART_c6805	266	YP_001019096	0.0	GO:0044763	60	45.0542	46
ARC_ART_c6811	240	CDX72708	1.0	-	56	33.113	55
ARC_ART_c6817	180	XP_008811577	0.0	-	70	37.3502	37
ARC_ART_c6824	114	XP_002539959	0.1	-	64	35.039	37
ARC_ART_c6828	111	XP_002957290	0.1	-	65	34.6538	35
ARC_ART_c6836	275	XP_004507946	0.0	GO:0006310	83	78.9518	48

ARC_ART_c6844	214	BAJ94909	0.0	GO:0008152	72	92.8189	70
ARC_ART_c6845	111	XP_009350055	0.0	-	62	40.817	35
ARC_ART_c6861	132	XP_010326162	0.0	-	72	39.2762	36
ARC_ART_c6869	173	XP_005842602	0.0	-	63	41.9726	36
ARC_ART_c6876	303	XP_009350051	0.0	-	54	67.781	106
ARC_ART_c6883	104	XP_005647728	0.0	-	77	38.5058	35
ARC_ART_c6895	428	XP_011088385	0.6	-	53	35.8094	67
ARC_ART_c6909	108	XP_004253462	0.0	-	68	41.5874	35
ARC_ART_c6912	118	EMT22893	0.0	-	78	37.3502	41
ARC_ART_c6915	205	AJE71484	0.1	-	54	35.8094	50
ARC_ART_c6919	258	XP_011016603	0.9	-	49	33.4982	81
ARC_ART_c6925	161	XP_002536222	0.0	GO:0006260	83	77.411	48
ARC_ART_c6939	181	AEX32522	0.0	GO:0006810	53	43.5134	60
ARC_ART_c6940	149	XP_005652006	0.0	-	62	41.9726	50
ARC_ART_c6944	115	XP_009337105	0.0	GO:0003723	77	51.2174	36
ARC_ART_c6966	166	XP_005847078	0.0	-	61	38.5058	36
ARC_ART_c6971	159	XP_008350252	0.3	-	60	33.8834	38
ARC_ART_c6974	136	XP_010467227	0.0	GO:0005829	74	58.5362	35
ARC_ART_c6987	163	XP_010922478	0.0	-	58	38.1206	48
ARC_ART_c6996	204	XP_007154471	0.0	GO:0006546	73	69.707	60
ARC_ART_c6998	205	XP_002537276	0.0	-	77	50.0618	40
ARC_ART_c7000	113	XP_002988013	0.0	GO:0010155	88	64.6994	36
ARC_ART_c7001	158	XP_008813253	0.0	GO:0044763	60	55.8398	55
ARC_ART_c7004	114	KEH15576	0.1	-	59	34.6538	37
ARC_ART_c7006	116	XP_002535428	0.0	-	71	47.3654	38
ARC_ART_c7015	134	XP_004514017	0.0	GO:0016021	80	55.8398	42

ARC_ART_c7024	193	AAD20634	0.8	-	44	33.113	38
ARC_ART_c7028	129	KEH15643	0.0	-	70	43.1282	40
ARC_ART_c7037	123	KIZ07913	0.0	-	72	47.7506	40
ARC_ART_c7039	159	XP_002536890	0.0	-	70	40.4318	50
ARC_ART_c7043	121	EEC68421	0.0	GO:0006810	92	73.9442	39
ARC_ART_c7046	127	AFK36966	0.0	GO:0016620	85	48.1358	34
ARC_ART_c7050	137	XP_002535408	0.0	GO:0016301	80	58.9214	45
ARC_ART_c7060	115	XP_010911369	0.1	-	68	35.039	38
ARC_ART_c7067	219	XP_001786560	0.0	GO:0006281	77	71.2478	72
ARC_ART_c7068	138	XP_002976165	0.0	GO:0009074	73	55.4546	42
ARC_ART_c7077	136	KDD72511	0.0	GO:0016491	83	70.0922	43
ARC_ART_c7084	122	XP_002537246	0.0	GO:0055114	79	60.077	39
ARC_ART_c7088	153	XP_005845976	0.0	GO:0005829	82	68.1662	50
ARC_ART_c7090	106	XP_001764907	0.3	-	61	33.8834	34
ARC_ART_c7097	161	EEE68528	0.0	GO:0006879	82	80.4925	52
ARC_ART_c7103	115	XP_001775197	0.0	-	63	41.2022	38
ARC_ART_c7134	275	XP_002537492	0.0	GO:0016491	68	60.077	48
ARC_ART_c7137	107	AFW82471	0.8	-	47	32.3426	34
ARC_ART_c7144	146	KFM25320	0.0	GO:0008152	72	60.8474	48
ARC_ART_c7158	185	XP_009350054	0.0	-	75	83.9593	61
ARC_ART_c7164	107	XP_001775412	0.0	GO:0006950	85	61.2326	35
ARC_ART_c7171	192	XP_006587982	0.4	-	44	34.2686	56
ARC_ART_c7172	259	BAK22529	0.0	GO:0016829	75	72.4034	58
ARC_ART_c7176	186	NP_038383	0.0	GO:0003899	86	86.6557	53
ARC_ART_c7182	122	YP_009057757	0.0	GO:0003899	92	72.4034	39
ARC_ART_c7197	148	EXB81509	0.4	-	58	33.4982	34
ARC_ART_c7204	160	KJB54149	0.0	-	60	37.7354	48

ARC_ART_c7207	116	XP_004979277	0.2	-	63	34.2686	36
ARC_ART_c7217	127	AAT46463	0.0	GO:0003333	100	87.0409	42
ARC_ART_c7218	212	XP_005851635	0.0	-	62	46.595	51
ARC_ART_c7220	111	XP_001783993	0.0	GO:0047652	85	50.8322	35
ARC_ART_c7223	219	XP_001422307	0.0	-	58	56.9954	72
ARC_ART_c7224	108	XP_005648735	0.0	-	68	40.0466	35
ARC_ART_c7232	139	YP_001019104	0.0	GO:0003899	82	54.6842	39
ARC_ART_c7239	124	EEC76877	0.0	GO:0046034	72	53.1434	36
ARC_ART_c7241	178	CCO19474	0.0	GO:0016874	74	69.3218	54
ARC_ART_c7244	116	EPS67479	0.0	-	71	53.9138	38
ARC_ART_c7251	195	AGV09141	0.1	-	50	35.4242	52
ARC_ART_c7252	151	ABS87674	0.0	GO:0046872	71	54.6842	49
ARC_ART_c7256	212	XP_005644833	0.0	-	62	55.8398	66
ARC_ART_c7260	117	KFM24082	0.0	GO:0005618	92	76.6406	39
ARC_ART_c7266	107	XP_001777328	0.0	-	80	38.891	35
ARC_ART_c7271	134	XP_011070578	0.7	-	61	32.7278	36
ARC_ART_c7274	117	XP_002539810	0.0	GO:0042626	94	65.855	38
ARC_ART_c7275	138	CCO14056	0.4	-	66	33.8834	36
ARC_ART_c7276	104	XP_005649750	0.0	GO:0006546	88	52.7582	34
ARC_ART_c7286	114	XP_009418049	0.7	-	50	31.5722	42
ARC_ART_c7288	188	ADV16380	0.0	-	63	38.891	38
ARC_ART_c7310	153	XP_009350062	0.0	GO:0005488	79	58.5362	49
ARC_ART_c7313	146	XP_002307976	0.0	GO:0006200	92	67.0106	40
ARC_ART_c7316	211	XP_002503206	0.0	-	68	53.5286	47
ARC_ART_c7323	128	XP_004253340	0.0	-	73	48.9062	42
ARC_ART_c7336	152	XP_003080670	0.1	-	66	35.039	39
ARC_ART_c7339	108	KFM27378	0.0	GO:0046872	85	53.9138	34
ARC_ART_c7341	142	XP_007155205	0.0	GO:0008152	70	55.8398	47

ARC_ART_c7342	217	KFM28987	0.2	-	59	35.4242	37
ARC_ART_c7344	147	ABF85791	0.0	GO:0044435	69	57.7658	46
ARC_ART_c7371	136	XP_011016264	0.0	-	80	43.5134	45
ARC_ART_c7373	125	XP_008804847	0.5	-	55	32.7278	38
ARC_ART_c7374	179	BAK08067	0.9	-	63	30.8018	38
ARC_ART_c7396	196	YP_009105080	0.0	GO:0003899	82	55.0694	34
ARC_ART_c7397	150	XP_001702483	0.0	GO:0009987	69	54.6842	49
ARC_ART_c7413	120	XP_005847368	0.0	GO:0016740	65	42.743	38
ARC_ART_c7419	112	XP_002945926	0.0	-	66	41.2022	36
ARC_ART_c7422	135	XP_002538544	0.0	-	59	37.3502	37
ARC_ART_c7425	120	XP_002536709	0.0	GO:0009987	76	51.2174	39
ARC_ART_c7427	257	XP_002537093	0.0	-	56	41.9726	46
ARC_ART_c7428	139	XP_001786381	0.0	-	68	47.7506	45
ARC_ART_c7429	168	EDQ48091	0.0	-	63	45.4394	46
ARC_ART_c7441	128	XP_001700547	0.9	-	60	32.3426	38
ARC_ART_c7449	228	XP_001692598	0.0	GO:0004832	80	60.077	35
ARC_ART_c7451	143	AAX95392	0.0	-	58	36.5798	43
ARC_ART_c7454	159	XP_002537364	0.0	-	63	40.817	46
ARC_ART_c7469	209	XP_002488912	0.0	GO:0006413	76	51.2174	39
ARC_ART_c7472	118	XP_002508526	0.0	-	75	54.6842	37
ARC_ART_c7480	127	XP_002536475	0.0	-	70	45.4394	41
ARC_ART_c7488	138	BAJ93395	0.6	-	61	33.113	42
ARC_ART_c7493	165	XP_002535209	0.0	GO:0044763	74	50.8322	43
ARC_ART_c7498	145	XP_001702917	0.0	GO:0016491	72	65.4698	54
ARC_ART_c7509	119	XP_002538274	0.0	-	63	36.1946	41
ARC_ART_c7514	118	XP_009350067	0.0	-	66	46.2098	39
ARC_ART_c7520	123	XP_009350808	0.0	-	100	68.5514	40
ARC_ART_c7529	103	XP_001786671	0.0	-	70	38.891	34
ARC_ART_c7530	187	XP_002504510	0.0	-	55	42.3578	60

ARC_ART_c7532	151	XP_006854095	0.5	-	52	33.4982	36
ARC_ART_c7538	117	XP_002507813	0.0	-	58	44.2838	39
ARC_ART_c7542	152	XP_002963212	0.0	GO:0043231	80	58.5362	41
ARC_ART_c7548	220	XP_007022579	0.0	-	59	41.2022	62
ARC_ART_c7551	118	KJB09764	0.0	-	97	80.4925	39
ARC_ART_c7562	142	EEC75666	0.0	-	70	36.5798	34
ARC_ART_c7570	166	XP_001768560	0.2	-	51	35.039	47
ARC_ART_c7587	152	AFO38380	0.6	-	60	33.4982	43
ARC_ART_c7588	147	XP_002539989	0.0	-	68	36.1946	38
ARC_ART_c7606	124	XP_002504456	0.1	-	60	34.6538	41
ARC_ART_c7615	161	XP_002535370	0.0	-	62	36.1946	37
ARC_ART_c7618	104	KCW84883	0.0	GO:0047427	82	63.1586	34
ARC_ART_c7620	120	XP_002538769	0.0	-	69	38.1206	39
ARC_ART_c7628	146	AGO03824	0.5	-	57	33.4982	35
ARC_ART_c7634	166	XP_002503794	0.0	GO:0071704	68	45.4394	38
ARC_ART_c7643	148	XP_002966130	0.3	-	55	33.8834	45
ARC_ART_c7645	304	CDX69174	0.0	GO:0019752	52	60.4622	70
ARC_ART_c7652	112	XP_009351102	0.0	GO:0008152	85	53.5286	35
ARC_ART_c7658	145	DAA52289	0.0	GO:0009414	79	70.4774	48
ARC_ART_c7663	134	XP_008350950	0.6	-	58	33.113	39
ARC_ART_c7664	115	CCO19717	0.6	-	61	32.7278	34
ARC_ART_c7676	224	AAV44205	0.0	GO:0009536	60	45.4394	50
ARC_ART_c7679	210	CDP02681	0.0	GO:0009987	53	45.4394	63
ARC_ART_c7681	134	KCW44113	0.0	GO:0009086	92	70.8626	39
ARC_ART_c7688	172	XP_007135229	0.2	-	56	35.039	39

ARC_ART_c7692	108	XP_006285797	0.0	-	62	39.6614	35
ARC_ART_c7699	138	XP_004981303	0.0	GO:0009536	76	59.6918	46
ARC_ART_c7704	151	EMT16671	0.0	GO:0009630	79	74.7146	49
ARC_ART_c7712	165	EMT02208	0.6	-	60	33.4982	40
ARC_ART_c7719	242	XP_010238691	0.5	-	44	34.2686	75
ARC_ART_c7721	121	XP_001690361	0.0	GO:0004601	73	58.9214	38
ARC_ART_c7743	124	XP_002540197	0.0	-	55	36.5798	40
ARC_ART_c7747	135	XP_006847847	0.0	-	61	45.4394	39
ARC_ART_c7771	128	EEE68527	0.0	GO:0009982	97	75.485	37
ARC_ART_c7772	105	XP_005851332	0.0	-	73	39.2762	34
ARC_ART_c7780	141	XP_003637074	0.0	GO:0008152	95	55.0694	43
ARC_ART_c7792	151	XP_003629958	0.0	GO:0051536	76	60.8474	46
ARC_ART_c7795	159	EPS63870	0.0	-	63	36.965	49
ARC_ART_c7799	496	XP_008459498	0.0	-	56	46.2098	78
ARC_ART_c7810	126	XP_006291199	0.1	-	63	35.8094	38
ARC_ART_c7812	202	XP_009350817	0.0	-	76	51.2174	34
ARC_ART_c7813	169	XP_009350065	0.0	GO:0003723	75	71.2478	54
ARC_ART_c7814	223	KDD73118	0.0	GO:0009853	85	57.7658	40
ARC_ART_c7815	287	CDY70253	0.0	GO:0005768	81	104.375	72
ARC_ART_c7838	187	XP_002536964	0.0	-	69	36.965	42
ARC_ART_c7853	123	YP_009057515	0.0	GO:0008270	88	56.9954	35
ARC_ART_c7860	106	AFK38157	0.4	-	67	31.187	34
ARC_ART_c7861	114	XP_003569216	0.8	-	52	31.5722	38
ARC_ART_c7866	144	XP_008234905	0.7	-	65	32.7278	35
ARC_ART_c7874	158	XP_001786972	0.1	-	60	34.6538	43

ARC_ART_c7881	112	XP_005850252	0.0	-	59	42.3578	37
ARC_ART_c7893	161	XP_011015719	0.0	-	55	41.5874	43
ARC_ART_c7901	265	XP_002537558	0.0	-	71	47.7506	42
ARC_ART_c7909	163	P10792	0.0	-	57	43.5134	49
ARC_ART_c7912	204	XP_002991687	0.3	-	56	34.6538	39
ARC_ART_c7913	168	EEC77966	0.0	GO:0030170	96	86.6557	50
ARC_ART_c7918	185	EEE54984	0.0	GO:0050826	95	93.5893	47
ARC_ART_c7921	124	CBI22452	0.0	GO:0003887	76	53.1434	39
ARC_ART_c7947	139	XP_011098320	0.0	GO:0044699	67	43.8986	40
ARC_ART_c7948	127	EYU43272	0.0	GO:0008152	76	43.8986	34
ARC_ART_c7979	123	XP_005651412	0.0	GO:0006164	65	49.2914	41
ARC_ART_c7980	163	CDL72943	0.0	-	65	38.891	38
ARC_ART_c7984	161	XP_003061168	0.0	GO:0009113	75	69.707	52
ARC_ART_c7986	134	XP_002537752	0.0	GO:0016491	70	49.2914	37
ARC_ART_c7988	107	XP_002535709	0.4	-	59	33.113	37
ARC_ART_c7997	170	XP_002535367	0.0	-	70	46.9802	41
ARC_ART_c8004	113	CDX90915	0.0	GO:0055114	78	46.9802	37
ARC_ART_c8012	201	XP_002536324	0.0	GO:0004341	80	70.4774	50
ARC_ART_c8014	192	XP_002534891	0.0	-	65	56.225	58
ARC_ART_c8024	168	AGV54820	0.0	-	61	41.9726	54
ARC_ART_c8061	227	EEC76877	0.0	GO:0044763	51	41.9726	52
ARC_ART_c8067	260	XP_006283121	0.0	-	54	37.7354	51
ARC_ART_c8085	107	XP_004305641	0.4	-	65	33.113	35
ARC_ART_c8092	145	ADZ24714	0.0	-	57	37.3502	45
ARC_ART_c8108	394	CDX71648	0.0	-	69	58.151	52
ARC_ART_c8111	122	XP_002448141	0.0	GO:0052624	80	55.8398	40
ARC_ART_c8112	121	XP_001415432	0.0	GO:0044249	71	50.447	39

ARC_ART_c8122	192	XP_002505163	0.0	GO:0008152	69	68.1662	52
ARC_ART_c8123	143	ADE77025	0.2	-	62	34.6538	35
ARC_ART_c8127	113	XP_006342106	0.0	-	70	36.965	37
ARC_ART_c8134	149	XP_010911369	0.0	-	65	41.9726	46
ARC_ART_c8154	127	XP_009782015	0.1	-	55	35.4242	45
ARC_ART_c8222	169	XP_009350814	0.0	-	59	38.5058	47
ARC_ART_c8230	186	EAY88134	0.0	-	55	42.743	58
ARC_ART_c8238	224	XP_002540158	0.0	-	53	38.5058	43
ARC_ART_c8244	116	KJB47089	0.3	-	62	33.4982	37
ARC_ART_c8245	148	XP_002538274	0.0	-	63	44.2838	41
ARC_ART_c8260	124	XP_009355056	0.2	-	71	34.6538	39
ARC_ART_c8267	139	KIZ00274	0.0	GO:0016747	73	53.1434	46
ARC_ART_c8272	144	XP_002535815	0.0	GO:0016208	95	78.9518	44
ARC_ART_c8283	200	XP_002538424	0.0	GO:0050794	77	57.7658	44
ARC_ART_c8294	168	XP_005651800	0.0	GO:0009836	89	86.6557	55
ARC_ART_c8297	278	BAJ11784	0.2	-	66	35.039	36
ARC_ART_c8303	173	XP_001415725	0.0	GO:0008233	64	44.669	50
ARC_ART_c8317	126	ERN03158	0.0	GO:0006629	67	46.9802	37
ARC_ART_c8358	108	KIZ07342	0.4	-	57	33.4982	35
ARC_ART_c8367	107	XP_005843402	0.0	GO:0050660	80	44.2838	35
ARC_ART_c8368	160	XP_001787086	0.0	GO:0006259	77	69.3218	53
ARC_ART_c8372	113	XP_001753273	0.0	-	68	49.2914	35
ARC_ART_c8373	155	XP_010044201	0.6	-	52	33.113	40

ARC_ART_c8387	128	XP_011016509	0.0	GO:0003735	82	65.855	41
ARC_ART_c8398	141	XP_005646623	0.0	GO:0044763	66	55.4546	51
ARC_ART_c8399	135	XP_002535681	0.0	GO:0005975	74	51.9878	43
ARC_ART_c8402	158	XP_002983288	0.0	GO:0004028	74	62.3882	51
ARC_ART_c8430	242	XP_003084074	0.6	-	73	33.8834	38
ARC_ART_c8434	209	KEH15495	0.0	-	51	41.2022	64
ARC_ART_c8440	153	XP_002322430	0.0	GO:0003723	81	72.7886	49
ARC_ART_c8444	160	EEC68217	0.0	GO:0044763	65	51.6026	52
ARC_ART_c8451	208	XP_006585221	0.5	-	50	33.8834	46
ARC_ART_c8457	111	XP_008238865	0.3	-	62	33.113	35
ARC_ART_c8459	165	XP_001786506	0.0	GO:0008152	78	71.633	51
ARC_ART_c8463	256	XP_002969283	0.0	GO:0016740	55	49.2914	84
ARC_ART_c8468	227	XP_002536302	0.0	GO:0051287	79	85.1149	68
ARC_ART_c8482	277	EDQ48453	0.0	-	67	83.5741	88
ARC_ART_c8486	104	XP_002534841	0.0	GO:0016021	85	54.6842	34
ARC_ART_c8496	187	XP_002535323	0.0	GO:0005975	73	41.9726	42
ARC_ART_c8498	203	XP_002539048	0.0	-	61	53.9138	65
ARC_ART_c8535	273	XP_002536601	0.0	-	80	53.5286	45
ARC_ART_c8541	187	AAV44205	0.2	-	57	34.6538	35
ARC_ART_c8544	225	XP_002513064	0.2	-	52	35.4242	48
ARC_ART_c8560	283	XP_004253340	0.0	-	63	63.929	63
ARC_ART_c8561	346	XP_002535661	0.0	-	62	72.4034	78
ARC_ART_c8562	127	XP_001697321	0.1	-	56	34.6538	37
ARC_ART_c8563	184	XP_002540155	0.0	-	56	37.7354	50
ARC_ART_c8565	249	ADN34251	0.1	-	64	29.261	34
ARC_ART_c8584	109	XP_002537961	0.0	GO:0016020	73	41.2022	34

ARC_ART_c8598	156	XP_002962977	0.0	GO:0016799	72	55.4546	48
ARC_ART_c8605	204	KDD75558	0.0	-	60	35.8094	40
ARC_ART_c8615	142	XP_008802337	0.5	-	54	33.4982	42
ARC_ART_c8632	128	KDO73193	0.0	-	60	39.6614	40
ARC_ART_c8637	186	BAA96252	0.0	GO:0016874	65	56.6102	61
ARC_ART_c8641	115	XP_001773138	0.0	-	67	36.965	34
ARC_ART_c8642	154	XP_002536270	0.0	-	72	38.5058	43
ARC_ART_c8648	168	XP_010322802	0.0	GO:0044765	69	55.8398	46
ARC_ART_c8659	119	EEC75666	0.0	GO:0032508	84	58.5362	39
ARC_ART_c8661	190	XP_002537989	0.0	-	72	38.891	37
ARC_ART_c8664	116	EEC76122	0.0	GO:0044763	73	53.5286	38
ARC_ART_c8666	108	XP_005843628	0.0	GO:0005524	91	58.151	34
ARC_ART_c8684	122	XP_002538704	0.0	-	61	40.817	39
ARC_ART_c8699	126	XP_005849294	0.0	GO:0003824	92	74.3294	42
ARC_ART_c8707	260	AAD28476	0.8	-	48	33.4982	41
ARC_ART_c8713	135	XP_002536424	0.0	-	82	58.5362	41
ARC_ART_c8723	128	XP_009351046	0.0	-	71	48.1358	42
ARC_ART_c8728	151	ERN13324	0.0	GO:0006164	70	51.2174	50
ARC_ART_c8744	118	XP_002538274	0.0	-	70	44.2838	37
ARC_ART_c8765	149	KDO55940	0.0	GO:0005840	79	67.3958	49
ARC_ART_c8778	178	BAF01627	0.0	-	62	33.4982	37
ARC_ART_c8793	209	XP_009785662	0.3	-	56	34.6538	44
ARC_ART_c8809	147	EDQ48466	0.0	-	70	42.743	37
ARC_ART_c8810	135	EEC78702	0.0	GO:0015413	80	56.6102	42
ARC_ART_c8811	113	XP_002517199	0.5	-	50	33.113	36
ARC_ART_c8812	206	XP_009350072	0.0	GO:0003735	88	76.2554	45
ARC_ART_c8825	157	AFK40414	0.0	-	52	38.5058	40
ARC_ART_c8832	131	NP_817264	0.0	-	65	41.5874	38
ARC_ART_c8835	116	XP_002539786	0.0	-	64	38.5058	37

ARC_ART_c8848	169	XP_002463993	0.6	-	53	33.4982	39
ARC_ART_c8849	176	XP_002539886	0.0	-	55	43.1282	58
ARC_ART_c8857	115	XP_001769120	0.4	-	67	32.7278	37
ARC_ART_c8859	186	EEC76122	0.0	-	50	41.9726	62
ARC_ART_c8868	133	XP_006428561	0.1	-	57	33.4982	35
ARC_ART_c8888	241	XP_002538424	0.0	GO:0016301	72	72.4034	76
ARC_ART_c8901	122	XP_002980554	0.0	-	64	41.5874	37
ARC_ART_c8905	104	AAS87594	0.0	GO:0017111	91	63.1586	34
ARC_ART_c8915	158	NP_001176614	0.4	-	40	33.8834	45
ARC_ART_c8924	147	XP_002539900	0.0	-	54	39.6614	44
ARC_ART_c8928	130	XP_005848152	0.0	GO:0098655	82	65.4698	41
ARC_ART_c8935	111	XP_002536196	0.0	GO:0008233	83	50.447	36
ARC_ART_c8936	127	KEH15576	0.0	-	59	41.5874	37
ARC_ART_c8943	108	XP_002539886	0.0	-	71	42.3578	35
ARC_ART_c8945	114	XP_001787063	0.0	-	80	50.447	35
ARC_ART_c8952	131	CDO99324	0.0	GO:0016556	82	66.2402	41
ARC_ART_c8953	108	EDQ48524	0.0	GO:0003824	82	59.3066	35
ARC_ART_c8972	237	EEC80862	0.0	GO:0009536	83	94.3597	68
ARC_ART_c8978	110	EEC68420	0.0	-	67	36.965	34
ARC_ART_c8989	122	ADE77266	0.0	GO:0004174	78	54.299	38
ARC_ART_c9000	156	XP_009350065	0.0	GO:0006810	84	46.9802	45
ARC_ART_c9015	153	XP_002536031	0.0	-	54	37.3502	37
ARC_ART_c9021	145	XP_002536620	0.0	-	57	38.1206	45
ARC_ART_c9036	101	XP_002536940	0.0	GO:0001882	77	40.817	35
ARC_ART_c9038	140	KEH15665	0.0	-	67	46.2098	46

ARC_ART_c9045	139	XP_001765367	0.3	-	57	34.2686	35
ARC_ART_c9049	110	XP_009359083	0.4	-	62	33.113	35
ARC_ART_c9051	109	XP_002534677	0.0	-	63	38.5058	36
ARC_ART_c9060	121	XP_002960036	0.0	-	65	40.817	38
ARC_ART_c9085	113	XP_002539732	0.1	-	61	33.4982	36
ARC_ART_c9090	193	XP_002977262	0.0	-	64	39.2762	42
ARC_ART_c9094	123	XP_004513992	0.5	-	56	31.5722	41
ARC_ART_c9102	150	XP_002539002	0.7	-	64	32.7278	34
ARC_ART_c9106	231	NP_201144	0.9	-	54	33.113	35
ARC_ART_c9109	218	XP_010278555	0.3	-	61	34.2686	36
ARC_ART_c9112	113	XP_009400935	0.5	-	57	32.7278	35
ARC_ART_c9116	110	AAB22587	0.0	GO:0005774	100	74.7146	36
ARC_ART_c9117	154	XP_008387702	0.0	GO:0006259	61	50.0618	55
ARC_ART_c9119	173	KEH15214	0.0	-	71	45.0542	38
ARC_ART_c9122	115	XP_001786874	0.0	-	67	37.7354	37
ARC_ART_c9126	179	XP_002264403	0.2	-	57	35.039	40
ARC_ART_c9146	104	EPS68915	0.0	GO:0044249	76	43.5134	34
ARC_ART_c9155	194	XP_010911626	0.0	GO:0006457	89	113.235	64
ARC_ART_c9157	133	KDD76196	0.0	GO:0009987	73	54.6842	42
ARC_ART_c9177	282	KEH15547	0.0	GO:1902192	62	56.225	90
ARC_ART_c9180	143	EAY87977	0.0	-	64	38.1206	37
ARC_ART_c9225	170	BAA25069	0.0	GO:0004129	83	77.7962	56
ARC_ART_c9226	233	XP_003060993	0.0	-	72	55.4546	47
ARC_ART_c9265	131	KIY99636	0.0	-	65	39.2762	35
ARC_ART_c9266	199	EMT05074	0.0	GO:0005618	77	49.6766	44
ARC_ART_c9272	144	CAA75386	0.0	GO:0009536	70	65.855	48
ARC_ART_c9282	126	EEC67125	0.0	-	67	46.9802	40
ARC_ART_c9300	119	XP_004253332	0.0	-	68	40.0466	38
ARC_ART_c9319	180	BAJ86385	0.0	-	65	42.3578	41

ARC_ART_c9321	236	CDY12056	0.7	-	58	33.8834	46
ARC_ART_c9338	120	CDY33090	0.0	-	68	36.1946	38
ARC_ART_c9359	110	XP_002537093	0.0	-	76	44.669	34
ARC_ART_c9363	194	EYU41774	0.0	GO:0009536	61	55.0694	67
ARC_ART_c9375	115	XP_008811580	0.0	-	71	47.3654	35
ARC_ART_c9391	174	XP_005844291	0.0	-	62	43.1282	45
ARC_ART_c9395	192	BAB10031	0.0	GO:0010027	61	33.8834	42
ARC_ART_c9399	161	XP_008788101	0.0	-	58	36.1946	36
ARC_ART_c9400	138	XP_002536738	0.0	-	79	46.9802	34
ARC_ART_c9402	140	AAL32456	0.0	-	65	35.8094	44
ARC_ART_c9409	108	KDD76331	0.6	-	55	32.3426	34
ARC_ART_c9413	134	CDX67969	0.3	-	61	32.3426	36
ARC_ART_c9423	162	EAY93131	0.2	-	55	26.1794	34
ARC_ART_c9455	126	KIZ01661	0.0	-	65	43.1282	35
ARC_ART_c9463	140	AHZ63825	0.7	-	50	33.113	46
ARC_ART_c9467	108	XP_005645178	0.0	-	67	36.5798	34
ARC_ART_c9470	174	XP_004297435	0.2	-	55	34.6538	47
ARC_ART_c9483	196	XP_002536999	0.0	-	61	63.1586	57
ARC_ART_c9484	124	EEC77998	0.0	-	68	36.965	38
ARC_ART_c9504	207	XP_011463569	0.9	-	57	33.113	45
ARC_ART_c9512	204	XP_002974016	0.5	-	53	33.8834	49
ARC_ART_c9515	205	AFK37255	0.0	-	65	39.2762	41
ARC_ART_c9526	142	KFK28695	0.0	GO:0010363	79	60.4622	43
ARC_ART_c9534	122	XP_001702324	0.0	GO:0046872	85	68.9366	40
ARC_ART_c9548	140	XP_004497767	0.5	-	55	33.113	38
ARC_ART_c9551	202	XP_001787071	0.0	-	77	39.2762	35
ARC_ART_c9563	153	EEC69860	0.0	-	71	40.817	35
ARC_ART_c9581	189	XP_005849685	0.0	GO:0016787	61	58.9214	62

ARC_ART_c9584	116	XP_002537613	0.0	GO:0016020	75	54.299	37
ARC_ART_c9596	148	XP_002960003	0.0	-	73	55.0694	41
ARC_ART_c9599	104	EMT12469	0.0	-	67	41.9726	34
ARC_ART_c9600	220	XP_008792176	0.9	-	48	33.113	54
ARC_ART_c9638	109	KDD74620	0.0	-	74	40.4318	35
ARC_ART_c9640	127	KDP32562	0.2	-	58	32.7278	39
ARC_ART_c9641	101	XP_008448288	1.0	-	55	31.5722	34
ARC_ART_c9642	210	XP_010270665	0.4	-	52	34.2686	65
ARC_ART_c9656	190	XP_008654990	0.5	-	56	33.4982	46
ARC_ART_c9671	189	EDQ48537	0.0	GO:0006284	79	90.1225	59
ARC_ART_c9687	151	KIY98601	0.0	GO:0008446	88	93.9745	50
ARC_ART_c9688	127	XP_002540597	0.0	-	69	36.1946	36
ARC_ART_c9693	123	XP_002537029	0.0	-	66	52.373	39
ARC_ART_c9708	135	AAF16526	0.0	GO:0044267	84	66.2402	45
ARC_ART_c9718	231	XP_004146950	0.0	GO:0044699	56	47.3654	50
ARC_ART_c9725	206	BAJ86385	0.0	-	61	62.3882	68
ARC_ART_c9727	108	XP_001421937	0.0	-	74	40.817	35
ARC_ART_c9729	184	XP_002966928	0.0	-	50	41.2022	53
ARC_ART_c9745	133	ABB69848	0.0	-	69	39.6614	36
ARC_ART_c9767	123	BAJ94909	0.0	GO:0006189	82	64.6994	39
ARC_ART_c9769	133	XP_008653671	0.3	-	55	32.7278	36
ARC_ART_c9776	112	EAZ10012	0.0	GO:0006537	86	63.929	36
ARC_ART_c9778	173	DAA53363	0.5	-	46	33.8834	47
ARC_ART_c9785	168	XP_002954794	0.0	-	60	48.521	46

ARC_ART_c9788	185	EEE67873	0.0	-	58	37.3502	43
ARC_ART_c9792	183	XP_002537608	0.0	-	61	39.6614	52
ARC_ART_c9798	190	EEC76122	0.0	GO:0044763	78	56.9954	41
ARC_ART_c9799	143	XP_001786908	0.5	-	56	32.7278	44
ARC_ART_c9802	103	CBI23954	0.0	GO:0009941	70	44.2838	34
ARC_ART_c9815	122	XP_001755170	0.0	-	70	38.5058	40
ARC_ART_c9841	127	XP_001703237	0.2	-	56	33.4982	37
ARC_ART_c9855	153	XP_009350805	0.0	GO:0006399	78	73.559	51
ARC_ART_c9861	210	XP_002539628	0.0	GO:0003677	69	46.2098	56
ARC_ART_c9877	236	EYU26907	0.0	GO:0009295	78	77.7962	51
ARC_ART_c9900	139	CEF98142	0.2	-	58	34.2686	41
ARC_ART_c9906	122	CCO18629	0.0	GO:0000166	72	56.225	40
ARC_ART_c9909	116	XP_001752799	0.0	-	68	46.595	38
ARC_ART_c9921	178	XP_002984851	0.0	GO:0005773	80	63.1586	47
ARC_ART_c9952	141	KCW63739	0.1	-	65	34.2686	35
ARC_ART_c9964	181	XP_002540444	0.0	GO:1901363	76	44.669	39
ARC_ART_c9968	147	KFM22551	0.1	-	56	33.113	46
ARC_ART_c9970	197	KIY94117	0.0	GO:0008652	77	73.9442	58
ARC_ART_c9975	125	EEC74306	0.5	-	66	33.113	36
ARC_ART_c9983	146	ABC24948	0.0	GO:1901576	79	58.9214	39
ARC_ART_c9984	127	XP_006450386	0.0	-	70	37.7354	34
ARC_ART_c9992	108	XP_002537860	0.0	GO:0050794	72	42.743	36
ARC_ART_c9993	220	KIZ00619	0.0	GO:0009086	84	61.6178	39
ARC_ART_c9997	216	XP_002537864	0.0	-	61	47.7506	62
ARC_ART_c10000	158	XP_002536320	0.0	-	68	49.2914	44
ARC_ART_c10003	125	XP_009624585	0.0	GO:0048767	81	58.151	37
ARC_ART_c10026	129	XP_011014727	0.0	GO:0003747	78	56.225	41
ARC_ART_c10030	118	XP_002539702	0.2	-	64	33.4982	34
ARC_ART_c10032	180	XP_003616487	0.0	GO:0008152	84	86.6557	51

ARC_ART_c10040	158	XP_002535911	0.2	-	50	34.2686	51
ARC_ART_c10054	127	KEH42674	0.0	GO:0008152	71	40.817	35
ARC_ART_c10060	111	EEC80776	0.0	GO:0016020	69	45.0542	36
ARC_ART_c10078	169	XP_001701444	0.0	-	59	36.5798	37
ARC_ART_c10080	138	XP_002467123	0.0	GO:0005524	79	51.6026	44
ARC_ART_c10094	151	XP_007018903	0.9	-	51	32.3426	41
ARC_ART_c10097	163	KDD77116	0.0	GO:0005488	71	43.1282	35
ARC_ART_c10105	139	AFW74701	0.0	GO:0044249	68	46.595	41
ARC_ART_c10136	136	XP_002539125	0.0	-	58	40.0466	43
ARC_ART_c10140	113	CBI29921	0.0	-	62	38.1206	37
ARC_ART_c10150	127	XP_011016741	0.0	-	100	85.5001	39
ARC_ART_c10151	135	EMS47955	0.0	-	78	48.9062	37
ARC_ART_c10186	250	XP_005645067	0.0	GO:0043231	61	70.4774	90
ARC_ART_c10190	170	EEC79492	0.0	GO:0004519	72	62.3882	51
ARC_ART_c10195	146	XP_002501910	0.0	-	57	37.3502	47
ARC_ART_c10198	133	XP_007009023	0.2	-	68	34.6538	38
ARC_ART_c10208	208	ACN59916	0.0	GO:0071704	66	43.1282	39
ARC_ART_c10215	115	EEE52318	0.0	GO:0022891	89	42.3578	37
ARC_ART_c10216	146	BAJ99191	0.7	-	60	32.3426	38
ARC_ART_c10221	172	XP_004486276	0.6	-	53	33.4982	43
ARC_ART_c10243	151	XP_001783027	0.0	GO:0016874	77	46.595	35
ARC_ART_c10244	119	XP_002539059	0.0	-	69	36.1946	39
ARC_ART_c10285	120	EAY93131	0.0	-	67	40.817	40
ARC_ART_c10292	110	XP_003057817	0.0	-	64	35.4242	34
ARC_ART_c10300	118	KDO60871	0.3	-	58	33.8834	39
ARC_ART_c10308	112	XP_002951676	0.0	-	75	48.1358	37
ARC_ART_c10311	156	XP_010060644	0.0	GO:0016491	67	51.6026	46

ARC_ART_c10328	137	XP_002539100	0.0	GO:2001289	88	70.0922	45
ARC_ART_c10334	129	XP_010911674	0.0	GO:0008047	69	55.4546	42
ARC_ART_c10339	138	KIZ04301	0.0	GO:0019363	77	55.4546	35
ARC_ART_c10347	161	CCO15906	0.4	-	54	32.3426	51
ARC_ART_c10352	202	YP_009105848	0.0	GO:0044763	68	56.6102	54
ARC_ART_c10358	115	XP_002537700	0.0	GO:0005215	81	57.3806	38
ARC_ART_c10361	245	CCO15415	0.0	-	60	72.7886	81
ARC_ART_c10372	152	EEE68528	0.0	GO:0055085	100	78.9518	37
ARC_ART_c10382	116	XP_002537571	0.0	GO:0016020	76	44.2838	38
ARC_ART_c10388	116	EMT00263	0.0	GO:0016491	72	50.447	37
ARC_ART_c10390	238	EMT14667	0.2	-	47	35.039	51
ARC_ART_c10401	123	NP_001173707	0.7	-	51	32.7278	37
ARC_ART_c10406	140	CDY12956	0.8	-	48	32.3426	39
ARC_ART_c10411	266	XP_008670825	0.1	-	47	36.1946	48
ARC_ART_c10412	451	YP_001152215	0.0	-	71	53.5286	39
ARC_ART_c10417	228	EEC70905	0.0	GO:0003857	79	72.7886	68
ARC_ART_c10442	129	EMT14578	0.0	-	65	40.817	38
ARC_ART_c10454	248	XP_002534643	0.0	-	48	38.1206	81
ARC_ART_c10471	124	BAJ96594	0.7	-	57	32.7278	40
ARC_ART_c10475	262	XP_003599135	0.5	-	48	32.3426	66
ARC_ART_c10483	181	XP_004966333	0.4	-	56	33.8834	48
ARC_ART_c10491	141	CDY17739	0.9	-	55	32.7278	34
ARC_ART_c10503	125	XP_011016758	0.0	-	78	57.3806	38
ARC_ART_c10505	150	KIZ06014	0.0	GO:0005975	83	72.4034	48
ARC_ART_c10511	107	XP_002535390	0.0	-	71	42.743	39
ARC_ART_c10534	145	XP_002265166	0.0	-	60	40.0466	46
ARC_ART_c10537	395	XP_010024865	0.4	-	48	35.8094	110

ARC_ART_c10543	173	KIZ07365	0.0	GO:0005488	72	50.8322	43
ARC_ART_c10564	155	XP_002537961	0.0	GO:0016020	72	42.3578	36
ARC_ART_c10568	120	XP_001786560	0.0	GO:0006281	73	41.2022	38
ARC_ART_c10569	154	XP_006485755	0.6	-	47	33.4982	44
ARC_ART_c10570	104	XP_007199623	0.2	-	61	34.6538	34
ARC_ART_c10579	151	XP_001758782	0.0	GO:0006810	72	63.929	48
ARC_ART_c10586	120	XP_001786560	0.0	GO:0006289	92	65.855	38
ARC_ART_c10596	122	XP_010060195	0.6	-	60	32.7278	35
ARC_ART_c10607	177	XP_002534939	0.0	GO:1901363	71	59.3066	59
ARC_ART_c10609	217	XP_002537107	0.0	-	67	85.5001	73
ARC_ART_c10613	117	XP_011043647	0.0	-	70	41.2022	40
ARC_ART_c10615	198	AAF16526	0.0	GO:0016036	73	72.7886	64
ARC_ART_c10632	179	XP_001763219	0.2	-	59	34.6538	44
ARC_ART_c10637	144	XP_002464694	0.0	-	59	41.5874	44
ARC_ART_c10639	122	XP_004498303	0.0	GO:0032508	80	56.225	40
ARC_ART_c10654	170	CEF98039	0.0	-	65	48.9062	46
ARC_ART_c10657	157	XP_009363537	0.5	-	53	33.4982	45
ARC_ART_c10659	167	XP_002459635	0.5	-	41	33.8834	55
ARC_ART_c10660	204	XP_002540107	0.0	GO:0004872	88	68.1662	45
ARC_ART_c10668	330	XP_001419465	0.9	-	45	33.8834	70
ARC_ART_c10670	136	XP_002537713	0.0	-	85	72.4034	41
ARC_ART_c10673	198	XP_009142072	0.0	-	58	38.891	55
ARC_ART_c10675	147	CAD71256	0.0	GO:0008652	68	44.2838	47
ARC_ART_c10694	113	EMT00267	0.0	GO:0005524	89	59.6918	37
ARC_ART_c10699	299	XP_005645808	0.0	-	47	39.2762	84
ARC_ART_c10715	114	XP_002535442	0.0	-	69	42.3578	36
ARC_ART_c10721	125	XP_002536189	0.0	GO:0003824	68	51.2174	41

ARC_ART_c10735	115	EEC76122	0.0	GO:0044763	72	51.6026	37
ARC_ART_c10751	123	XP_002539959	0.1	-	60	35.4242	35
ARC_ART_c10753	107	KJB52284	0.0	-	68	37.7354	35
ARC_ART_c10791	115	YP_009106863	0.0	-	80	47.7506	35
ARC_ART_c10795	200	XP_011070837	0.5	-	72	33.8834	40
ARC_ART_c10807	147	XP_003557145	0.1	-	65	35.8094	35
ARC_ART_c10809	123	XP_009111084	0.0	GO:0005777	72	45.4394	40
ARC_ART_c10819	145	XP_001786560	0.0	GO:0006281	74	49.2914	47
ARC_ART_c10833	136	XP_002538224	0.0	GO:0004871	78	51.6026	41
ARC_ART_c10834	154	XP_010026242	0.0	-	66	40.0466	42
ARC_ART_c10838	294	EEC76877	0.0	GO:0044763	68	116.701	106
ARC_ART_c10857	127	XP_002317660	0.1	-	63	35.8094	36
ARC_ART_c10867	121	KDO54096	0.0	-	68	37.3502	38
ARC_ART_c10887	200	XP_002436837	0.6	-	49	33.4982	57
ARC_ART_c10929	175	CAN82657	0.3	-	46	33.113	52
ARC_ART_c10932	138	ADZ15230	0.0	GO:0016020	76	45.0542	38
ARC_ART_c10937	110	XP_002538424	0.2	-	65	33.8834	35
ARC_ART_c10939	164	XP_005649017	0.0	GO:0055114	88	100.523	54
ARC_ART_c10940	181	NP_001105965	0.4	-	48	33.8834	49
ARC_ART_c10942	110	XP_002503307	0.0	GO:0003824	69	42.3578	36
ARC_ART_c10962	143	CAB90679	0.0	GO:0003824	71	47.3654	46
ARC_ART_c10965	154	XP_009350076	0.0	GO:0003746	95	45.4394	46
ARC_ART_c11007	136	XP_002537190	0.0	GO:0006810	75	45.4394	37
ARC_ART_c11008	187	XP_002534658	0.0	-	55	42.743	59
ARC_ART_c11012	131	XP_001786560	0.0	GO:0006281	74	57.3806	43
ARC_ART_c11016	116	XP_006353220	0.0	GO:0005739	75	44.669	40
ARC_ART_c11017	157	XP_001417951	0.0	GO:0006783	88	80.4925	51
ARC_ART_c11028	169	XP_002992823	0.0	-	63	39.2762	38
ARC_ART_c11039	112	XP_007214562	0.0	GO:0010048	77	43.8986	36

ARC_ART_c11046	128	XP_001690386	0.0	GO:0071704	65	42.3578	40
ARC_ART_c11062	109	XP_011005310	0.0	GO:0016070	74	43.5134	35
ARC_ART_c11078	128	XP_003558125	0.9	-	65	31.9574	35
ARC_ART_c11079	133	XP_009350051	0.0	GO:0030529	77	61.2326	44
ARC_ART_c11098	152	XP_010910071	0.0	-	59	40.0466	44
ARC_ART_c11116	141	XP_001773279	0.0	GO:0003824	70	42.3578	48
ARC_ART_c11121	215	EPS64887	0.7	-	49	33.4982	59
ARC_ART_c11141	129	XP_002538310	0.0	-	58	43.1282	48
ARC_ART_c11157	132	XP_002979914	0.0	GO:0000166	77	55.4546	40
ARC_ART_c11160	144	XP_002536879	0.0	-	69	48.9062	49
ARC_ART_c11161	153	XP_002537186	0.0	-	53	37.3502	47
ARC_ART_c11165	117	XP_005642926	0.0	-	58	37.7354	36
ARC_ART_c11185	121	KEH15576	0.1	-	61	35.4242	39
ARC_ART_c11187	177	XP_002536377	0.0	-	54	36.5798	51
ARC_ART_c11212	103	ACU17171	0.0	GO:0005507	94	51.6026	34
ARC_ART_c11213	197	XP_006653128	0.2	-	57	35.039	49
ARC_ART_c11223	122	XP_002538475	0.0	GO:0015419	85	52.7582	35
ARC_ART_c11226	143	KDD75281	0.0	GO:0003824	88	67.781	35
ARC_ART_c11235	214	KDD77186	0.0	GO:0005524	80	80.1073	61
ARC_ART_c11243	143	KIZ01665	0.6	-	59	32.7278	42
ARC_ART_c11246	153	XP_005648112	0.0	GO:0005488	66	41.9726	39
ARC_ART_c11255	122	KFM26532	0.1	-	52	34.6538	40
ARC_ART_c11262	135	XP_001695450	0.0	GO:1901701	65	47.3654	44
ARC_ART_c11267	198	YP_009105280	0.0	GO:0044444	67	67.0106	56
ARC_ART_c11270	123	BAE20401	0.0	-	67	39.6614	37
ARC_ART_c11275	249	XP_004514127	0.0	-	58	37.3502	46
ARC_ART_c11307	160	XP_002536193	0.0	GO:0015416	85	75.0998	49

ARC_ART_c11313	148	XP_002536930	0.5	-	58	33.113	36
ARC_ART_c11314	119	KDO73255	0.0	GO:0044272	97	64.3142	35
ARC_ART_c11319	177	XP_002537745	0.0	-	62	36.1946	35
ARC_ART_c11331	193	KJB44141	0.0	-	62	40.0466	35
ARC_ART_c11333	109	EEC77111	0.0	GO:0004871	77	53.1434	36
ARC_ART_c11334	113	XP_002500330	0.2	-	72	33.8834	36
ARC_ART_c11337	162	EEE67873	0.0	GO:0016740	86	59.3066	53
ARC_ART_c11355	130	KGN48828	0.0	GO:0006629	72	52.373	43
ARC_ART_c11360	126	EEE51040	0.0	GO:0005507	100	91.2781	41
ARC_ART_c11369	164	AGT17256	0.3	-	47	34.2686	48
ARC_ART_c11394	186	XP_001418781	0.0	GO:0016020	71	60.077	56
ARC_ART_c11398	105	XP_002537560	0.0	GO:0003677	82	48.521	34
ARC_ART_c11401	199	EEC71809	0.3	-	65	34.6538	46
ARC_ART_c11410	151	CCO14210	0.8	-	56	33.113	44
ARC_ART_c11417	160	XP_002535771	0.0	GO:0006184	97	75.0998	42
ARC_ART_c11434	117	XP_009804862	0.0	GO:0098655	73	47.7506	38
ARC_ART_c11436	162	XP_002540263	0.0	-	89	72.7886	46
ARC_ART_c11437	148	XP_011016603	0.0	GO:0004591	85	80.8777	49
ARC_ART_c11443	110	AAQ73136	0.0	GO:0009630	82	60.4622	35
ARC_ART_c11445	162	XP_009350069	0.0	GO:0005840	78	71.633	52
ARC_ART_c11518	106	XP_008381810	0.0	-	71	46.2098	35
ARC_ART_c11522	153	XP_002534960	0.1	-	60	35.8094	46
ARC_ART_c11525	187	AGC78943	0.0	-	73	49.6766	34
ARC_ART_c11530	133	ABI18050	0.0	-	55	34.2686	43
ARC_ART_c11538	117	EMT28156	0.0	GO:0006950	79	51.9878	34
ARC_ART_c11542	180	CBB36473	0.0	GO:0009987	68	35.4242	38
ARC_ART_c11543	104	XP_005851680	0.0	GO:0004451	79	50.0618	34

ARC_ART_c11548	106	XP_008441168	0.0	GO:0046872	76	50.0618	34
ARC_ART_c11553	157	XP_002540107	0.5	-	59	33.113	49
ARC_ART_c11555	106	EDQ48662	0.0	-	77	48.521	35
ARC_ART_c11570	160	ABW89596	0.0	-	62	38.891	51
ARC_ART_c11610	160	XP_011030772	0.0	-	60	36.965	45
ARC_ART_c11617	120	XP_005844252	0.0	GO:0005507	80	51.6026	36
ARC_ART_c11623	110	EEC76919	0.0	GO:0016740	97	69.3218	36
ARC_ART_c11635	132	EEE51121	0.3	-	50	31.5722	46
ARC_ART_c11641	107	XP_011016264	0.3	-	88	33.4982	35
ARC_ART_c11645	120	CAW30992	0.0	GO:0004872	83	44.2838	36
ARC_ART_c11648	118	XP_002538193	0.0	-	89	72.0182	38
ARC_ART_c11675	203	XP_003599577	0.0	-	67	41.5874	34
ARC_ART_c11678	134	BAK00705	0.0	-	61	37.7354	39
ARC_ART_c11682	206	XP_009356293	0.0	GO:0006259	71	46.2098	39
ARC_ART_c11689	162	XP_009765598	0.0	GO:0004252	79	77.0258	49
ARC_ART_c11697	127	XP_001786938	0.0	-	65	38.5058	38
ARC_ART_c11712	121	ADE76842	0.0	GO:0006633	79	58.5362	39
ARC_ART_c11716	171	XP_002536715	0.0	-	57	57.7658	57
ARC_ART_c11734	154	EDQ48366	0.0	GO:0016853	75	65.855	48
ARC_ART_c11754	187	XP_002501855	0.0	GO:0016638	57	60.4622	61
ARC_ART_c11759	326	XP_003588355	0.8	-	62	34.2686	35
ARC_ART_c11761	273	XP_009130250	0.1	-	68	36.1946	38
ARC_ART_c11780	125	XP_003542073	0.9	-	54	32.3426	37
ARC_ART_c11781	126	XP_009350057	0.0	-	66	46.595	42
ARC_ART_c11782	106	EEE68528	0.0	GO:0055085	100	70.4774	35
ARC_ART_c11803	147	XP_002954563	0.0	-	68	51.9878	48
ARC_ART_c11817	129	XP_005649907	0.1	-	51	33.113	43

ARC_ART_c11824	121	XP_003064423	0.5	-	58	32.7278	41
ARC_ART_c11881	164	XP_002881366	0.6	-	58	33.4982	50
ARC_ART_c11883	179	XP_009618502	0.2	-	54	35.039	50
ARC_ART_c11893	111	XP_003635968	0.0	GO:0006200	86	47.7506	36
ARC_ART_c11895	134	EEC76122	0.0	GO:0050660	83	55.0694	37
ARC_ART_c11920	136	XP_005652229	0.0	-	63	38.891	44
ARC_ART_c11942	127	AFJ72983	0.0	-	64	45.8246	42
ARC_ART_c11962	165	XP_002534816	0.0	GO:0016772	70	64.3142	57
ARC_ART_c11968	125	XP_002975644	0.0	-	64	40.4318	39
ARC_ART_c11977	143	XP_005646096	0.0	-	69	40.817	36
ARC_ART_c11985	267	AAU90319	0.0	-	64	45.4394	37
ARC_ART_c11986	119	AAL56243	0.0	GO:0006457	74	52.373	39
ARC_ART_c12004	155	XP_005644522	0.0	-	67	45.0542	40
ARC_ART_c12008	110	EYU26952	0.7	-	74	32.3426	35
ARC_ART_c12038	132	XP_002534764	0.0	GO:0004871	73	44.669	34
ARC_ART_c12040	150	XP_011028659	0.6	-	56	33.113	51
ARC_ART_c12066	130	XP_011470057	0.6	-	55	32.7278	40
ARC_ART_c12073	184	CAN68832	0.8	-	55	33.113	38
ARC_ART_c12074	143	EEE52322	0.0	GO:0016810	80	67.781	46
ARC_ART_c12076	123	XP_005652102	0.2	-	69	34.2686	39
ARC_ART_c12081	173	EEC76877	0.0	GO:0046034	73	53.1434	45
ARC_ART_c12099	157	XP_007023919	0.3	-	61	31.5722	34

ARC_ART_c12100	169	CDY11017	0.6	-	64	33.4982	37
ARC_ART_c12105	172	XP_001773753	0.0	-	70	46.595	41
ARC_ART_c12109	123	ACA52205	0.0	GO:0030976	84	40.817	39
ARC_ART_c12129	146	XP_005851729	0.0	GO:0044710	71	54.299	45
ARC_ART_c12130	197	XP_004306973	0.0	GO:0003746	82	55.4546	34
ARC_ART_c12132	119	XP_002539707	0.0	-	61	36.1946	39
ARC_ART_c12134	154	YP_009047459	0.1	-	49	35.4242	51
ARC_ART_c12138	128	XP_001786955	0.0	-	65	35.8094	38
ARC_ART_c12142	108	KDD73007	0.0	GO:0005622	71	44.669	35
ARC_ART_c12163	137	BAD26579	0.0	-	68	36.965	35
ARC_ART_c12174	177	EDQ48526	0.0	-	76	50.0618	38
ARC_ART_c12176	134	KJB44141	0.0	-	62	42.3578	35
ARC_ART_c12179	189	BAJ93795	0.0	GO:0001101	76	64.3142	50
ARC_ART_c12186	141	KFK35070	0.0	GO:0044249	65	42.743	44
ARC_ART_c12203	128	XP_002979857	0.0	GO:0016021	80	51.2174	35
ARC_ART_c12219	130	CDP20508	0.0	-	67	39.6614	34
ARC_ART_c12222	103	KIZ02350	0.2	-	73	34.2686	34
ARC_ART_c12241	118	KIY95357	0.0	-	72	45.4394	36
ARC_ART_c12247	150	XP_010932021	0.5	-	48	33.4982	35
ARC_ART_c12258	143	XP_006430245	0.2	-	63	34.6538	41
ARC_ART_c12268	160	XP_002535822	0.0	-	67	55.8398	53
ARC_ART_c12273	155	XP_002536193	0.0	GO:0015408	76	73.559	50
ARC_ART_c12278	183	XP_008347341	0.7	-	46	33.113	43
ARC_ART_c12281	422	KGN49989	0.5	-	58	35.4242	46
ARC_ART_c12285	190	XP_005647813	0.0	GO:0051287	80	52.373	51
ARC_ART_c12295	190	EAY95695	0.0	-	56	40.817	46
ARC_ART_c12299	155	XP_002536715	0.4	-	62	33.4982	43
ARC_ART_c12307	148	ERN06846	0.0	GO:0004018	87	77.7962	48
ARC_ART_c12315	128	XP_002537877	0.0	GO:0008233	66	47.3654	42

ARC_ART_c12325	108	KFM25838	0.0	GO:0097159	75	41.5874	36
ARC_ART_c12329	155	XP_011014867	0.0	-	100	76.2554	36
ARC_ART_c12330	153	KDP20116	0.4	-	48	33.4982	37
ARC_ART_c12334	155	AJR29335	0.0	-	66	40.817	45
ARC_ART_c12338	201	XP_003560841	0.6	-	53	33.4982	39
ARC_ART_c12341	124	XP_009336961	0.8	-	48	32.7278	37
ARC_ART_c12351	180	XP_009370147	0.2	-	70	33.8834	34
ARC_ART_c12360	119	XP_003605652	0.3	-	59	33.8834	37
ARC_ART_c12364	193	XP_004975291	0.9	-	44	33.113	52
ARC_ART_c12384	145	EEC68217	0.0	-	57	36.5798	47
ARC_ART_c12386	166	XP_002980069	0.0	GO:0009987	58	40.0466	53
ARC_ART_c12392	115	XP_001694486	0.8	-	50	32.3426	40
ARC_ART_c12401	127	XP_002537872	0.0	-	83	39.6614	42
ARC_ART_c12402	156	EEC80776	0.0	GO:0042450	82	48.521	39
ARC_ART_c12405	170	XP_011022727	0.7	-	53	33.113	39
ARC_ART_c12413	134	XP_002535815	0.0	GO:0016208	78	59.6918	42
ARC_ART_c12418	203	XP_002538359	0.0	-	61	47.7506	67
ARC_ART_c12432	108	XP_011016735	0.0	-	66	38.1206	36
ARC_ART_c12441	106	XP_003083193	0.0	GO:0044763	74	47.3654	35
ARC_ART_c12444	147	XP_009350805	0.0	-	77	62.7734	49
ARC_ART_c12445	170	XP_002538394	0.0	-	52	37.3502	46
ARC_ART_c12446	162	KIZ05661	0.0	GO:0006631	71	41.5874	38
ARC_ART_c12467	154	XP_002538660	0.0	-	70	46.2098	40
ARC_ART_c12468	112	EMT17544	0.0	-	71	45.8246	35
ARC_ART_c12470	117	CDX72049	0.0	-	65	36.1946	40
ARC_ART_c12482	146	ABM05481	0.0	-	65	45.4394	44
ARC_ART_c12484	123	XP_001787070	0.0	-	76	48.1358	39

ARC_ART_c12489	133	EEC84801	0.2	-	47	32.7278	38
ARC_ART_c12491	222	AGT17357	0.3	-	56	33.113	41
ARC_ART_c12495	226	AAR99497	0.0	GO:0016020	54	49.6766	71
ARC_ART_c12505	242	XP_007220241	0.9	-	46	33.4982	69
ARC_ART_c12512	132	XP_009337928	0.7	-	59	32.3426	42
ARC_ART_c12515	152	XP_002994527	0.0	GO:0030170	77	54.299	44
ARC_ART_c12520	112	XP_002534847	0.0	GO:0008152	74	47.7506	35
ARC_ART_c12538	103	XP_002536849	0.0	-	79	42.3578	34
ARC_ART_c12542	124	XP_011098546	0.6	-	47	33.113	38
ARC_ART_c12555	113	XP_002539991	0.1	-	62	34.2686	35
ARC_ART_c12583	130	EMS63384	0.5	-	62	32.7278	37
ARC_ART_c12588	113	XP_005851884	0.0	GO:0006537	91	61.2326	34
ARC_ART_c12599	229	EXC01440	0.7	-	63	33.8834	36
ARC_ART_c12608	134	XP_001422164	0.0	GO:0019543	70	63.5438	44
ARC_ART_c12612	194	XP_006420815	0.4	-	47	33.8834	55
ARC_ART_c12616	145	XP_003062090	0.0	GO:0009570	70	49.2914	47
ARC_ART_c12632	156	BAJ90553	0.0	GO:0008152	79	64.3142	43
ARC_ART_c12639	125	EYU44535	0.0	GO:0008270	92	68.9366	41
ARC_ART_c12659	252	XP_010436467	0.1	-	44	36.5798	74
ARC_ART_c12664	168	XP_003595723	0.0	-	80	46.595	35
ARC_ART_c12670	183	XP_002536273	0.0	GO:0016627	66	63.5438	59
ARC_ART_c12681	205	KFM28096	0.6	-	45	33.4982	59
ARC_ART_c12682	130	EEE52320	0.0	GO:0019752	72	43.8986	40
ARC_ART_c12685	268	XP_010244402	0.6	-	47	34.2686	61
ARC_ART_c12686	127	EXB52559	0.0	-	70	41.9726	37
ARC_ART_c12720	117	AAR87159	0.9	-	61	31.5722	36
ARC_ART_c12738	121	XP_002522716	0.3	-	60	33.113	38
ARC_ART_c12741	114	XP_002538398	0.0	GO:0004871	76	44.669	38
ARC_ART_c12756	124	XP_002539902	0.0	-	64	40.817	34
ARC_ART_c12764	154	XP_002974782	0.0	-	61	39.2762	47
ARC_ART_c12769	139	XP_007051207	0.7	-	60	30.8018	40
ARC_ART_c12772	152	YP_001152204	0.0	-	70	40.4318	34

ARC_ART_c12786	186	KCW50568	0.0	-	70	42.3578	41
ARC_ART_c12787	207	KEH15262	0.0	GO:0006810	89	56.225	37
ARC_ART_c12788	163	XP_002536227	0.0	GO:0003677	75	58.151	45
ARC_ART_c12790	103	CCO18895	0.7	-	61	32.7278	36
ARC_ART_c12791	128	AJD87506	0.3	-	62	33.8834	37
ARC_ART_c12797	150	XP_011014867	0.0	-	54	53.1434	62
ARC_ART_c12800	111	XP_002534887	0.1	-	61	34.6538	36
ARC_ART_c12812	154	XP_008240797	0.2	-	56	34.2686	39
ARC_ART_c12814	158	BAH57009	0.7	-	50	32.7278	38
ARC_ART_c12846	170	XP_004511872	0.0	-	55	47.3654	56
ARC_ART_c12847	109	CDP17106	0.0	GO:0016597	80	54.6842	36
ARC_ART_c12857	204	XP_002538868	0.0	GO:0044765	69	57.7658	53
ARC_ART_c12878	164	AFK94155	0.0	-	58	39.2762	51
ARC_ART_c12903	159	XP_002456686	0.3	-	56	34.2686	37
ARC_ART_c12921	144	ACA30301	0.1	-	52	33.113	36
ARC_ART_c12952	141	KEH15576	0.0	-	75	58.151	44
ARC_ART_c12956	167	XP_007163586	0.1	-	61	35.4242	36
ARC_ART_c12960	129	EEC81324	0.0	GO:0044763	72	57.3806	40
ARC_ART_c12967	154	CDP21815	0.0	GO:0016661	64	54.6842	51
ARC_ART_c12980	142	XP_009397928	0.0	-	60	43.1282	41
ARC_ART_c12987	199	XP_005652121	0.6	-	54	31.9574	37
ARC_ART_c13009	164	CDY27425	0.4	-	43	31.9574	44
ARC_ART_c13034	123	XP_010276004	0.0	GO:0004834	80	58.9214	40
ARC_ART_c13059	218	XP_001786955	0.0	GO:0009451	72	73.1738	70
ARC_ART_c13072	110	XP_004148833	0.2	-	55	33.8834	43
ARC_ART_c13093	115	XP_002500636	0.0	-	67	39.2762	37

ARC_ART_c13105	110	XP_001419815	0.0	GO:0006200	85	48.9062	35
ARC_ART_c13109	153	XP_002537042	0.4	-	69	33.4982	39
ARC_ART_c13123	121	AAF16526	0.0	GO:0007010	87	75.8702	41
ARC_ART_c13125	202	XP_010930164	0.1	-	50	34.6538	58
ARC_ART_c13147	131	XP_011016263	0.0	-	83	59.6918	42
ARC_ART_c13149	158	CDP04961	0.0	-	68	40.4318	47
ARC_ART_c13151	127	XP_002538952	0.0	-	89	60.4622	37
ARC_ART_c13209	110	XP_002538205	0.0	GO:0071704	77	50.8322	35
ARC_ART_c13253	148	XP_007133686	0.9	-	56	32.3426	44
ARC_ART_c13254	150	EYU34372	0.0	GO:0030529	65	45.0542	35
ARC_ART_c13262	213	XP_008775992	0.1	-	58	35.8094	46
ARC_ART_c13263	223	CCO19089	0.0	-	70	40.0466	44
ARC_ART_c13265	134	XP_002892041	0.0	-	65	37.3502	46
ARC_ART_c13280	106	XP_001786872	0.0	GO:0004489	80	53.9138	35
ARC_ART_c13282	108	KDO41527	0.0	GO:0046872	82	52.7582	35
ARC_ART_c13285	126	NP_001242401	0.2	-	63	34.6538	36
ARC_ART_c13293	109	XP_002536964	0.0	-	68	41.5874	35
ARC_ART_c13299	107	XP_002537219	0.0	-	65	36.5798	35
ARC_ART_c13302	118	XP_002535867	0.0	GO:0004731	97	72.7886	39
ARC_ART_c13308	111	XP_003635968	0.5	-	70	32.7278	34
ARC_ART_c13323	182	XP_002500056	0.1	-	68	36.5798	35
ARC_ART_c13329	161	XP_007216420	0.0	GO:0097159	60	46.9802	55
ARC_ART_c13342	149	XP_002538196	0.0	-	52	40.817	53
ARC_ART_c13349	140	EEC78702	0.7	-	61	32.7278	34
ARC_ART_c13350	111	XP_005847833	0.0	-	74	45.4394	35
ARC_ART_c13377	177	EPS68541	0.5	-	62	33.4982	40

ARC_ART_c13383	177	XP_002537144	0.0	-	62	43.8986	48
ARC_ART_c13403	130	XP_001696953	0.0	GO:0017038	86	74.7146	43
ARC_ART_c13413	122	KEH15495	0.0	GO:0044710	77	60.077	40
ARC_ART_c13416	132	XP_003082198	0.0	GO:0009630	83	63.1586	37
ARC_ART_c13427	190	XP_002441075	0.4	-	52	33.8834	36
ARC_ART_c13428	112	KDP36699	0.0	GO:0003723	81	46.595	38
ARC_ART_c13429	136	XP_002535045	0.0	-	88	76.6406	45
ARC_ART_c13431	120	AHJ08570	0.1	-	70	35.4242	34
ARC_ART_c13436	115	XP_009615004	0.0	-	74	44.669	35
ARC_ART_c13437	171	XP_007020098	0.3	-	48	34.2686	43
ARC_ART_c13446	126	XP_009773256	0.5	-	64	33.113	39
ARC_ART_c13460	143	XP_010911304	0.0	GO:0016021	89	51.2174	47
ARC_ART_c13478	282	XP_002537871	0.0	-	73	41.2022	38
ARC_ART_c13486	115	XP_005847566	0.0	-	59	39.2762	37
ARC_ART_c13489	215	ADE77849	0.2	-	50	35.039	52
ARC_ART_c13513	237	XP_011015834	0.0	-	98	142.895	72
ARC_ART_c13525	131	XP_002535408	0.0	-	69	38.891	43
ARC_ART_c13533	146	XP_004253340	0.0	-	68	47.7506	41
ARC_ART_c13545	161	CDP15242	0.0	GO:0071704	61	53.1434	52
ARC_ART_c13551	150	AAT46463	0.0	GO:0003333	82	51.9878	34
ARC_ART_c13555	109	XP_005844292	0.0	GO:0043167	73	42.743	34
ARC_ART_c13570	147	XP_005647813	0.0	-	67	38.891	34
ARC_ART_c13582	171	XP_009406447	0.0	GO:0003824	64	47.7506	45
ARC_ART_c13597	171	KDD71878	0.0	GO:0016491	67	44.2838	40
ARC_ART_c13603	145	XP_003619610	0.2	-	52	32.7278	40

ARC_ART_c13604	139	XP_010485949	0.1	-	58	35.039	36
ARC_ART_c13610	154	Q9FVG8	0.0	GO:0004478	91	56.9954	34
ARC_ART_c13611	203	XP_003614789	0.7	-	42	33.4982	66
ARC_ART_c13617	145	BAC99343	0.4	-	57	31.9574	35
ARC_ART_c13623	263	XP_002951308	0.0	GO:0046872	72	40.4318	37
ARC_ART_c13631	105	XP_005644522	0.0	-	70	43.8986	34
ARC_ART_c13632	237	XP_011014867	0.0	-	71	51.2174	39
ARC_ART_c13657	180	XP_002538452	0.1	-	54	34.2686	44
ARC_ART_c13664	122	XP_011016603	0.0	GO:0004591	88	61.2326	35
ARC_ART_c13668	117	XP_002535351	0.0	GO:0004735	83	55.8398	37
ARC_ART_c13676	127	EDQ48467	0.0	GO:0006281	82	63.929	41
ARC_ART_c13689	130	EEC77854	0.0	-	60	41.5874	43
ARC_ART_c13699	172	EPS70023	0.0	-	60	44.669	41
ARC_ART_c13712	167	CDY36727	0.0	GO:0006979	66	44.2838	48
ARC_ART_c13714	121	XP_011016758	0.0	-	82	65.855	40
ARC_ART_c13734	119	KIZ07660	0.0	-	58	39.2762	43
ARC_ART_c13749	325	KGN54736	0.0	-	50	42.3578	56
ARC_ART_c13750	143	XP_002535126	0.0	-	63	47.3654	47
ARC_ART_c13773	168	ERN00555	0.0	-	61	40.4318	44
ARC_ART_c13774	139	XP_006393623	0.2	-	52	32.3426	42
ARC_ART_c13786	145	XP_009350812	0.0	-	91	86.6557	48
ARC_ART_c13795	116	XP_011025303	0.8	-	55	32.3426	34
ARC_ART_c13796	179	XP_011077624	0.5	-	62	33.8834	43
ARC_ART_c13802	144	EAZ18903	0.3	-	46	34.2686	43
ARC_ART_c13803	142	XP_010039663	0.5	-	61	32.3426	39
ARC_ART_c13809	171	XP_002537101	0.0	-	69	59.3066	55

ARC_ART_c13812	181	XP_004228950	0.0	GO:0009744	59	52.7582	57
ARC_ART_c13822	139	CEG01389	0.0	GO:0016070	64	50.8322	42
ARC_ART_c13828	171	EPS74531	1.0	-	62	31.187	35
ARC_ART_c13834	150	XP_007198833	0.0	-	65	36.1946	38
ARC_ART_c13853	114	EEC75666	0.0	GO:0090305	78	46.9802	38
ARC_ART_c13857	227	XP_006359026	0.0	GO:0016740	65	36.5798	38
ARC_ART_c13883	348	KJB76688	0.0	-	56	41.5874	41
ARC_ART_c13891	136	XP_002536745	0.0	-	75	49.6766	40
ARC_ART_c13894	154	XP_001703001	0.0	GO:0009735	62	49.6766	54
ARC_ART_c13898	113	XP_003588326	0.0	-	67	40.4318	34
ARC_ART_c13912	181	XP_005647217	0.0	GO:0003723	87	99.7525	58
ARC_ART_c13919	135	XP_009351406	0.0	-	72	46.595	44
ARC_ART_c13925	105	KIZ03451	0.0	-	61	37.3502	34
ARC_ART_c13927	170	XP_010421825	0.0	GO:0009569	60	56.9954	63
ARC_ART_c13933	119	XP_006589740	0.0	GO:0016020	78	58.151	38
ARC_ART_c13970	341	EPS74505	0.0	-	52	56.6102	93
ARC_ART_c14020	106	XP_001689455	0.0	GO:0009536	64	43.1282	34
ARC_ART_c14022	142	XP_002539058	0.0	-	66	45.4394	39
ARC_ART_c14023	134	XP_001786560	0.0	GO:0006289	85	48.521	34
ARC_ART_c14028	114	XP_003078290	0.0	GO:0044763	75	53.5286	37
ARC_ART_c14030	136	KFM23132	0.0	GO:0009793	76	58.5362	43
ARC_ART_c14038	214	XP_001786904	0.5	-	55	33.113	67
ARC_ART_c14060	106	EEC78702	0.0	GO:0006200	85	61.2326	35
ARC_ART_c14074	266	XP_002536967	0.0	GO:0004871	77	96.2857	79
ARC_ART_c14119	105	BAJ94909	0.0	GO:0006189	79	51.6026	34
ARC_ART_c14147	124	XP_002535483	0.7	-	67	32.7278	34

ARC_ART_c14149	131	EEC76122	0.0	GO:0004149	95	80.4925	43
ARC_ART_c14157	135	XP_002539363	0.0	-	63	40.0466	41
ARC_ART_c14159	120	EDQ48453	0.0	-	71	37.3502	38
ARC_ART_c14161	124	XP_010943405	0.4	-	61	33.4982	39
ARC_ART_c14185	133	KGN56689	0.5	-	40	33.4982	54
ARC_ART_c14200	134	XP_010066016	0.0	GO:0008270	90	86.2705	44
ARC_ART_c14208	117	XP_003635968	0.0	GO:0017111	78	58.5362	38
ARC_ART_c14213	155	XP_002959790	0.0	-	55	43.5134	49
ARC_ART_c14223	136	XP_002537827	0.0	-	70	44.2838	40
ARC_ART_c14237	272	EPS59220	0.9	-	51	33.113	60
ARC_ART_c14241	171	EEC70904	0.0	-	47	36.5798	51
ARC_ART_c14242	133	YP_009106192	0.0	GO:0016020	78	55.0694	41
ARC_ART_c14280	192	XP_003077991	0.0	GO:0006952	72	81.6481	66
ARC_ART_c14307	118	XP_007198966	0.5	-	56	33.113	37
ARC_ART_c14310	129	XP_005847629	0.0	GO:0008152	75	54.6842	41
ARC_ART_c14320	123	KJB33327	0.6	-	67	32.7278	34
ARC_ART_c14323	116	EEE52318	0.0	GO:0022891	100	82.8037	38
ARC_ART_c14338	146	KCW71549	0.7	-	47	32.7278	40
ARC_ART_c14341	384	ACQ90971	0.0	GO:0003735	76	80.1073	60
ARC_ART_c14353	126	EEC75945	0.0	GO:0006200	88	59.3066	34
ARC_ART_c14411	155	AFW81840	0.0	-	54	38.5058	50
ARC_ART_c14414	126	XP_011079370	0.4	-	55	33.113	40
ARC_ART_c14437	122	XP_010935892	0.8	-	52	32.3426	36
ARC_ART_c14451	115	XP_001758097	0.0	-	60	42.3578	40
ARC_ART_c14465	155	XP_002534942	0.0	-	69	43.1282	36

ARC_ART_c14468	111	XP_004973442	0.7	-	58	31.9574	34
ARC_ART_c14474	110	CCA60844	0.0	GO:0004611	75	50.0618	37
ARC_ART_c14482	144	XP_002982914	0.2	-	56	34.2686	46
ARC_ART_c14483	154	XP_005649128	0.0	GO:0044763	70	50.447	51
ARC_ART_c14484	153	XP_007221805	0.0	-	66	50.0618	45
ARC_ART_c14485	119	XP_008222312	0.6	-	45	32.7278	35
ARC_ART_c14487	111	XP_004253348	0.0	-	70	45.0542	37
ARC_ART_c14525	121	XP_011464061	0.7	-	61	32.3426	36
ARC_ART_c14532	149	XP_005650027	0.0	-	65	38.5058	46
ARC_ART_c14539	235	XP_010267781	0.0	-	60	41.9726	70
ARC_ART_c14542	122	XP_002536196	0.0	GO:0016021	79	50.8322	34
ARC_ART_c14548	164	KCW69904	0.4	-	50	33.4982	54
ARC_ART_c14557	122	XP_002537126	0.2	-	66	34.6538	36
ARC_ART_c14560	105	XP_004491593	0.9	-	60	31.9574	35
ARC_ART_c14567	124	XP_009350817	0.0	GO:0006098	85	50.0618	40
ARC_ART_c14571	193	XP_009118960	0.0	GO:0016491	81	60.077	37
ARC_ART_c14579	189	EEC70905	0.0	GO:0016020	63	48.1358	46
ARC_ART_c14583	138	KEH15314	0.0	GO:0016620	83	65.855	37
ARC_ART_c14585	162	XP_002535353	0.0	GO:0004540	88	54.299	34
ARC_ART_c14587	183	XP_011016737	0.0	-	100	120.168	57
ARC_ART_c14612	128	XP_002539401	0.0	-	71	47.7506	39
ARC_ART_c14621	159	EEC70278	0.0	-	69	38.1206	36
ARC_ART_c14622	109	XP_002958615	0.0	GO:0004252	91	58.9214	37

ARC_ART_c14641	160	KEH16995	0.6	-	59	33.113	42
ARC_ART_c14665	183	XP_002538363	0.0	GO:0051287	79	84.3445	54
ARC_ART_c14674	138	XP_002537487	0.1	-	64	35.4242	42
ARC_ART_c14693	111	XP_001697305	0.0	-	64	35.039	37
ARC_ART_c14716	127	XP_010466928	0.3	-	61	33.113	39
ARC_ART_c14719	113	XP_002540217	0.5	-	67	32.3426	34
ARC_ART_c14729	214	EEC81066	0.0	-	52	48.9062	71
ARC_ART_c14743	111	XP_002948368	0.0	GO:0044763	72	50.447	36
ARC_ART_c14763	138	ABV04296	0.0	-	67	39.2762	34
ARC_ART_c14793	147	XP_002538077	0.0	-	62	39.2762	48
ARC_ART_c14813	121	XP_006305050	0.7	-	57	32.7278	38
ARC_ART_c14815	181	XP_009418871	0.2	-	66	33.4982	36
ARC_ART_c14822	190	XP_007221182	0.3	-	48	34.2686	56
ARC_ART_c14840	108	KIZ00800	0.0	GO:0016614	72	48.1358	36
ARC_ART_c14842	172	XP_009350805	0.0	-	75	54.6842	40
ARC_ART_c14848	105	XP_006577529	0.3	-	65	31.9574	35
ARC_ART_c14851	105	KIY96331	0.2	-	68	33.4982	35
ARC_ART_c14855	278	XP_001786885	0.0	-	59	38.891	54
ARC_ART_c14859	184	KIZ05603	0.0	GO:0016020	54	46.595	55
ARC_ART_c14867	150	XP_003579836	0.9	-	55	32.7278	38
ARC_ART_c14869	178	EYU17437	0.1	-	54	35.8094	50
ARC_ART_c14876	116	XP_002982181	0.3	-	63	33.113	36
ARC_ART_c14880	120	XP_002535552	0.0	-	67	40.817	37
ARC_ART_c14881	102	XP_002534563	0.0	GO:0003959	85	51.6026	34
ARC_ART_c14921	164	XP_005647439	0.0	-	56	40.817	53

ARC_ART_c14923	109	XP_002536292	0.0	GO:0046872	100	48.1358	35
ARC_ART_c14925	165	XP_002539331	0.0	-	66	45.8246	51
ARC_ART_c14930	152	DAA38917	0.0	GO:0017038	80	63.5438	45
ARC_ART_c14936	143	EAZ00026	0.0	GO:0016023	86	56.6102	44
ARC_ART_c14941	121	XP_011016720	0.0	-	65	39.2762	38
ARC_ART_c14944	151	XP_008793934	0.3	-	63	33.8834	36
ARC_ART_c14950	180	XP_006645748	0.0	GO:0044763	67	60.8474	59
ARC_ART_c14965	122	XP_003591245	0.8	-	55	32.7278	34
ARC_ART_c14971	112	EEC70905	0.0	GO:0003857	91	62.3882	36
ARC_ART_c14999	138	XP_011014867	0.0	-	76	55.4546	42
ARC_ART_c15012	133	EEC70905	0.0	GO:0044763	75	58.5362	41
ARC_ART_c15054	148	XP_011016696	0.0	GO:0005488	81	70.0922	49
ARC_ART_c15059	152	XP_008344463	0.0	-	64	43.8986	34
ARC_ART_c15064	113	AAF16525	0.0	GO:0071704	75	48.9062	37
ARC_ART_c15077	101	XP_009588089	0.0	GO:0044763	72	45.4394	36
ARC_ART_c15100	113	XP_002536536	0.0	-	62	36.1946	37
ARC_ART_c15113	106	CEF98150	0.0	GO:0016491	74	45.0542	35
ARC_ART_c15116	146	XP_002539229	0.0	GO:0004871	79	70.0922	48
ARC_ART_c15121	134	XP_011072466	0.0	GO:0016023	82	71.2478	45
ARC_ART_c15123	207	EPS57530	0.0	GO:0009536	69	77.0258	69
ARC_ART_c15131	147	EDQ49166	0.0	-	60	41.9726	45
ARC_ART_c15144	159	XP_002539628	0.1	-	53	35.039	52
ARC_ART_c15145	154	XP_002505433	0.0	-	55	35.8094	52
ARC_ART_c15166	158	XP_002946538	0.2	-	52	34.6538	48
ARC_ART_c15179	106	XP_002536407	0.0	GO:0000155	79	46.9802	34
ARC_ART_c15210	208	XP_002963340	0.0	-	53	43.1282	56
ARC_ART_c15214	186	XP_001696856	0.6	-	50	33.4982	51
ARC_ART_c15221	175	XP_001786560	0.0	GO:0044763	64	46.9802	50
ARC_ART_c15225	111	XP_002983415	0.0	GO:0044763	63	43.1282	36

ARC_ART_c15228	154	XP_002538318	0.0	GO:0016853	75	62.7734	44
ARC_ART_c15267	193	XP_003080549	0.0	-	56	55.4546	71
ARC_ART_c15283	200	XP_005844251	0.9	-	53	33.113	43
ARC_ART_c15309	143	XP_004253340	0.0	-	75	65.855	45
ARC_ART_c15322	183	XP_001696199	0.9	-	53	32.7278	49
ARC_ART_c15332	171	CCO19131	0.0	GO:0030170	75	62.3882	52
ARC_ART_c15344	145	XP_010237678	0.0	GO:1902582	78	70.0922	46
ARC_ART_c15346	182	XP_002537910	0.4	-	60	32.7278	35
ARC_ART_c15355	122	XP_002537415	0.1	-	61	35.039	36
ARC_ART_c15365	112	XP_009350065	0.0	-	86	60.8474	36
ARC_ART_c15366	185	KDP21500	0.6	-	50	33.113	57
ARC_ART_c15386	146	XP_002535817	0.1	-	60	35.8094	46
ARC_ART_c15433	138	ABB47515	0.4	-	47	33.4982	42
ARC_ART_c15445	123	CDY44779	0.9	-	56	30.8018	39
ARC_ART_c15462	187	XP_001752199	0.0	GO:0044710	68	60.4622	47
ARC_ART_c15482	175	BAD44751	0.3	-	54	34.2686	42
ARC_ART_c15483	173	XP_010056074	0.0	-	74	39.2762	35
ARC_ART_c15547	131	ACP30588	0.3	-	62	34.2686	40
ARC_ART_c15551	127	XP_002539909	0.5	-	59	32.3426	37
ARC_ART_c15552	145	XP_004253376	0.0	-	66	45.8246	39
ARC_ART_c15560	139	XP_002320427	0.0	-	61	40.4318	44
ARC_ART_c15564	128	XP_002519063	0.9	-	57	32.3426	35
ARC_ART_c15571	141	XP_005850093	0.0	GO:0009536	68	44.669	45
ARC_ART_c15574	119	XP_002537889	0.0	-	73	38.5058	34
ARC_ART_c15601	138	AFY10150	0.0	-	70	43.1282	41
ARC_ART_c15618	129	XP_004253332	0.5	-	83	33.113	42
ARC_ART_c15652	122	XP_006845262	0.0	GO:0044763	72	43.1282	37
ARC_ART_c15663	227	AAK49119	0.2	-	46	35.8094	76

ARC_ART_c15691	228	XP_005847833	0.0	GO:0006355	84	74.7146	52
ARC_ART_c15717	186	AAP53939	0.7	-	54	33.4982	50
ARC_ART_c15726	102	KJB54287	0.6	-	58	32.7278	34
ARC_ART_c15732	174	ERN03820	0.0	GO:0006098	84	90.1225	58
ARC_ART_c15754	137	EEE52320	0.0	GO:0055085	87	63.1586	41
ARC_ART_c15779	144	XP_008645660	0.4	-	53	33.8834	39
ARC_ART_c15788	125	XP_002977537	0.0	-	58	40.4318	41
ARC_ART_c15793	107	XP_002538445	0.0	-	61	37.3502	34
ARC_ART_c15819	158	XP_004499954	0.0	-	60	41.9726	46
ARC_ART_c15840	120	XP_002539023	0.0	-	74	41.5874	39
ARC_ART_c15842	197	XP_002537989	0.0	-	60	37.7354	51
ARC_ART_c15846	276	XP_002994726	0.0	-	54	39.2762	48
ARC_ART_c15877	173	BAP46307	0.0	-	57	41.2022	47
ARC_ART_c15879	128	KIY99636	0.0	-	68	38.5058	41
ARC_ART_c15895	131	XP_001417485	0.0	-	66	41.5874	36
ARC_ART_c15896	209	XP_002503674	0.0	GO:0042558	83	87.4261	67
ARC_ART_c15900	227	XP_011016263	0.0	GO:0006415	93	105.916	58
ARC_ART_c15911	208	XP_001419756	0.0	GO:0008152	60	58.9214	73
ARC_ART_c15915	167	XP_002536997	0.0	GO:0016020	74	53.9138	51
ARC_ART_c15920	111	XP_002540584	0.0	GO:0006508	91	63.929	36
ARC_ART_c15925	144	P53385	0.0	GO:0019557	95	75.485	42
ARC_ART_c15971	194	EEC79491	0.0	-	70	38.1206	40
ARC_ART_c15972	195	EMS63575	0.0	GO:0016655	100	143.28	65
ARC_ART_c16016	164	XP_004982481	0.2	-	52	34.6538	42
ARC_ART_c16017	118	XP_002539102	0.0	GO:0030170	78	51.9878	37
ARC_ART_c16030	104	ABI54652	0.0	GO:0046961	91	65.0846	34

ARC_ART_c16037	128	KEH15495	0.0	GO:0044710	75	58.9214	40
ARC_ART_c16040	196	XP_010911679	0.0	GO:0020037	80	103.99	63
ARC_ART_c16043	115	XP_001771411	0.0	GO:0005254	72	44.669	37
ARC_ART_c16047	151	XP_002539558	0.0	GO:0003824	70	56.6102	47
ARC_ART_c16062	108	1B1Y_A	0.8	-	70	32.3426	34
ARC_ART_c16067	229	XP_002535016	0.0	-	69	81.6481	75
ARC_ART_c16076	127	XP_005847592	0.0	GO:0008152	90	68.1662	41
ARC_ART_c16079	120	XP_006588355	0.3	-	51	33.4982	39
ARC_ART_c16083	178	XP_002537786	0.0	-	76	38.1206	34
ARC_ART_c16100	177	XP_004290516	0.8	-	56	33.113	53
ARC_ART_c16102	138	KDP30715	0.4	-	54	33.8834	44
ARC_ART_c16111	121	XP_002501910	0.1	-	66	35.8094	39
ARC_ART_c16112	248	AAK96700	0.2	-	34	35.4242	106
ARC_ART_c16116	130	AHI49897	0.4	-	69	32.3426	39
ARC_ART_c16129	136	XP_002540415	0.0	-	53	35.8094	45
ARC_ART_c16131	151	XP_002539815	0.2	-	57	34.2686	47
ARC_ART_c16133	196	XP_003556120	0.2	-	57	35.039	42
ARC_ART_c16136	168	XP_003578199	0.9	-	53	32.7278	43
ARC_ART_c16149	120	BAC42726	0.0	GO:0005829	61	45.8246	39
ARC_ART_c16177	113	KIZ06930	0.0	-	63	36.1946	36
ARC_ART_c16190	139	XP_002535814	0.2	-	60	34.2686	46
ARC_ART_c16193	118	KIY99235	0.0	GO:1901564	76	45.0542	34
ARC_ART_c16196	118	EEE52320	0.1	-	76	36.1946	38
ARC_ART_c16197	116	XP_001694090	0.0	GO:0016491	67	43.1282	37
ARC_ART_c16204	187	EEE66267	0.7	-	57	33.4982	42
ARC_ART_c16234	111	YP_009106658	0.0	GO:0046961	75	49.6766	36
ARC_ART_c16238	201	XP_005648430	0.0	-	56	36.965	65

ARC_ART_c16266	136	EXB74820	0.3	-	53	34.2686	45
ARC_ART_c16297	137	XP_002949714	0.0	GO:0016020	62	47.7506	43
ARC_ART_c16333	141	CDY40951	0.2	-	58	35.039	43
ARC_ART_c16359	160	XP_002966336	0.0	GO:0003824	71	51.2174	53
ARC_ART_c16362	172	XP_005851875	0.0	GO:0055114	81	84.3445	59
ARC_ART_c16372	156	KJB76688	0.0	-	62	35.039	37
ARC_ART_c16392	118	XP_009350065	0.0	GO:0009987	83	58.151	36
ARC_ART_c16400	185	XP_002540111	0.0	GO:0006259	79	59.3066	43
ARC_ART_c16406	186	XP_002539123	0.0	-	55	40.817	59
ARC_ART_c16413	134	XP_006855240	0.0	GO:0044237	68	43.1282	44
ARC_ART_c16442	189	BAE45851	0.6	-	55	33.4982	38
ARC_ART_c16450	132	XP_006492393	0.7	-	57	32.7278	35
ARC_ART_c16452	176	XP_007008973	0.6	-	48	33.113	47
ARC_ART_c16491	140	EAY84569	0.0	GO:0050660	80	54.6842	35
ARC_ART_c16492	147	XP_009350076	0.0	GO:0034660	82	67.781	46
ARC_ART_c16493	165	XP_004974731	0.4	-	45	33.8834	37
ARC_ART_c16506	187	XP_002534643	0.0	-	67	47.3654	43
ARC_ART_c16507	141	XP_004230064	0.0	GO:0009536	77	48.521	40
ARC_ART_c16522	138	XP_011040746	0.1	-	52	35.4242	42
ARC_ART_c16532	115	XP_009350065	0.0	-	73	38.891	34
ARC_ART_c16535	182	XP_006408721	0.2	-	62	35.039	45
ARC_ART_c16540	105	EPS66624	0.2	-	58	34.2686	34
ARC_ART_c16557	127	KIY97710	0.0	-	71	53.1434	42
ARC_ART_c16573	124	KEH15984	0.2	-	60	34.6538	41

ARC_ART_c16579	147	XP_003569141	0.1	-	65	35.039	35
ARC_ART_c16583	106	EEE52319	0.0	GO:0006378	100	70.4774	35
ARC_ART_c16625	192	XP_002537142	0.0	-	88	107.457	63
ARC_ART_c16633	106	XP_004977893	0.8	-	58	32.3426	34
ARC_ART_c16639	304	KDD74677	0.2	-	58	36.1946	50
ARC_ART_c16652	142	XP_008230686	0.5	-	50	32.7278	50
ARC_ART_c16655	184	AGC78945	0.0	-	58	37.7354	50
ARC_ART_c16677	125	XP_002536517	0.0	GO:0003824	78	63.1586	41
ARC_ART_c16693	131	EPS66367	0.3	-	58	33.4982	34
ARC_ART_c16696	179	XP_005652313	0.0	GO:0043231	81	90.1225	59
ARC_ART_c16728	167	CAC51690	0.9	-	57	32.7278	49
ARC_ART_c16748	126	BAJ94072	0.0	-	71	45.4394	42
ARC_ART_c16780	109	EPS74201	0.0	GO:0009536	75	43.5134	36
ARC_ART_c16783	114	EAY81384	0.4	-	91	32.7278	35
ARC_ART_c16786	109	XP_010942632	0.0	-	61	38.5058	34
ARC_ART_c16787	202	EEC77966	0.0	-	62	53.1434	67
ARC_ART_c16795	211	XP_001786933	0.1	-	60	36.1946	56
ARC_ART_c16798	107	XP_002967245	0.1	-	74	34.6538	35
ARC_ART_c16803	108	XP_011016757	0.0	-	80	50.8322	35
ARC_ART_c16814	131	KJB65057	0.7	-	55	32.3426	36
ARC_ART_c16824	145	AAR02225	0.0	GO:0046961	97	92.0485	48
ARC_ART_c16850	149	BAD21778	0.1	-	59	33.8834	42
ARC_ART_c16853	174	NP_904228	0.0	GO:0003735	85	74.7146	48
ARC_ART_c16857	107	XP_004486904	0.0	GO:0044710	71	45.8246	35
ARC_ART_c16864	185	CBI25078	0.5	-	56	33.4982	48
ARC_ART_c16871	114	AEP33255	0.0	GO:0046872	94	67.0106	37
ARC_ART_c16873	169	EYU27592	0.3	-	52	33.8834	40

ARC_ART_c16913	195	CDY47978	0.4	-	54	34.2686	46
ARC_ART_c16914	290	XP_001786560	0.0	GO:0044763	70	54.299	51
ARC_ART_c16917	135	XP_002500063	0.0	GO:0003723	72	56.6102	37
ARC_ART_c16929	101	XP_005644117	0.1	-	65	35.4242	35
ARC_ART_c16939	235	XP_002966763	0.1	-	47	35.8094	63
ARC_ART_c16941	125	XP_002534946	0.0	-	62	38.1206	35
ARC_ART_c16953	131	XP_009603098	0.1	-	63	35.4242	36
ARC_ART_c16985	134	XP_002536715	0.0	GO:0005524	84	63.929	38
ARC_ART_c16988	156	EEC76953	0.0	GO:0003849	80	46.2098	40
ARC_ART_c16993	267	XP_011017330	0.0	GO:0044763	60	69.707	93
ARC_ART_c16996	284	YP_006666485	0.0	GO:0005840	80	59.3066	36
ARC_ART_c17004	111	AFK39161	0.0	GO:0016151	94	70.4774	37
ARC_ART_c17014	114	XP_001417473	0.0	GO:0008152	76	44.669	34
ARC_ART_c17041	113	XP_003079070	0.3	-	51	33.8834	37
ARC_ART_c17046	115	KFK40375	0.4	-	67	32.7278	34
ARC_ART_c17057	147	XP_001778187	0.0	-	65	48.1358	49
ARC_ART_c17061	229	XP_002540194	0.0	GO:0030170	82	95.1301	62
ARC_ART_c17069	139	XP_002537289	0.0	-	58	36.5798	39
ARC_ART_c17075	110	ACO50725	0.0	GO:0046872	100	45.0542	36
ARC_ART_c17082	164	XP_005851680	0.0	GO:0006099	91	51.2174	35
ARC_ART_c17083	117	XP_006645523	0.0	GO:0043168	72	54.6842	37
ARC_ART_c17085	142	XP_002539849	0.0	-	85	53.9138	35
ARC_ART_c17089	137	XP_001759956	0.0	GO:0016208	88	78.5666	44
ARC_ART_c17100	131	XP_003059819	0.0	-	64	36.5798	37
ARC_ART_c17104	120	XP_001417006	0.0	GO:0005507	79	55.0694	39
ARC_ART_c17110	151	XP_009384493	0.0	-	68	43.8986	35

ARC_ART_c17122	129	XP_002534710	0.3	-	57	33.4982	38
ARC_ART_c17130	245	XP_002534939	0.0	-	72	56.9954	51
ARC_ART_c17162	316	EEE52320	0.0	GO:0009987	49	43.5134	104
ARC_ART_c17167	150	EPS62044	0.0	-	52	40.4318	53
ARC_ART_c17174	239	ABH09321	0.0	-	74	43.8986	39
ARC_ART_c17185	109	ACY30625	0.0	GO:0003866	78	51.6026	37
ARC_ART_c17211	186	ABA96699	0.9	-	45	33.113	77
ARC_ART_c17226	140	XP_009408858	0.5	-	52	33.4982	38
ARC_ART_c17239	178	XP_002538952	0.0	-	83	85.8853	54
ARC_ART_c17241	108	XP_002506839	0.0	GO:0016208	82	68.5514	35
ARC_ART_c17255	146	XP_002989901	0.8	-	56	32.7278	39
ARC_ART_c17257	135	XP_002539231	0.0	-	61	38.891	36
ARC_ART_c17260	115	KFM28346	0.0	-	64	39.6614	37
ARC_ART_c17264	274	XP_005645176	0.0	GO:0003824	67	53.9138	64
ARC_ART_c17272	147	XP_005643061	0.1	-	62	35.039	43
ARC_ART_c17311	187	XP_002298732	0.1	-	59	35.4242	42
ARC_ART_c17325	311	XP_009350051	0.0	GO:0005840	85	147.902	97
ARC_ART_c17356	229	KIZ03654	0.2	-	44	35.039	69
ARC_ART_c17362	105	XP_002537310	0.0	-	61	35.4242	34
ARC_ART_c17368	198	XP_003548539	0.0	-	53	37.7354	49
ARC_ART_c17369	124	AAM65143	0.0	-	66	40.0466	39
ARC_ART_c17373	107	AHI49897	0.0	GO:0098655	97	55.4546	35
ARC_ART_c17377	299	AGB85059	0.0	GO:0044434	64	78.1814	84
ARC_ART_c17388	136	XP_004139723	0.9	-	45	31.187	48
ARC_ART_c17389	198	XP_009125239	0.2	-	46	35.039	45
ARC_ART_c17390	123	AHI49897	0.0	GO:0006812	75	46.595	37
ARC_ART_c17392	284	YP_001152204	0.0	-	72	37.7354	36
ARC_ART_c17394	150	XP_009406657	0.3	-	64	34.2686	34
ARC_ART_c17395	108	XP_002535682	0.0	GO:0019134	82	50.8322	34

ARC_ART_c17404	114	XP_002507787	0.0	GO:0004450	84	58.151	38
ARC_ART_c17405	109	XP_002452377	0.7	-	61	32.3426	34
ARC_ART_c17421	126	BAH57117	0.0	-	69	37.3502	42
ARC_ART_c17422	124	XP_005648626	0.0	GO:0008270	90	68.1662	41
ARC_ART_c17426	132	KFM27474	0.1	-	52	33.4982	44
ARC_ART_c17434	239	XP_001422022	0.0	GO:0009295	83	77.0258	48
ARC_ART_c17441	140	XP_002539706	0.6	-	60	32.7278	41
ARC_ART_c17446	197	EPS70026	0.0	-	67	36.1946	34
ARC_ART_c17466	139	EEE68528	0.0	GO:0004672	100	98.9821	46
ARC_ART_c17469	108	EEC76774	0.0	GO:0042886	91	61.2326	35
ARC_ART_c17489	132	XP_002537029	0.0	-	72	52.373	44
ARC_ART_c17491	116	XP_002950038	0.0	GO:0046872	70	42.743	37
ARC_ART_c17508	113	YP_009105428	0.0	GO:0005840	97	65.0846	36
ARC_ART_c17513	156	EPS74490	0.1	-	51	35.4242	39
ARC_ART_c17519	167	XP_008803349	0.0	-	70	39.2762	40
ARC_ART_c17520	114	XP_011016757	0.0	-	76	47.7506	38
ARC_ART_c17530	129	EEE52320	0.0	-	75	36.965	41
ARC_ART_c17546	188	XP_001694441	0.0	GO:0044710	64	58.5362	62
ARC_ART_c17547	121	XP_005643141	0.0	-	70	38.1206	37
ARC_ART_c17554	121	XP_002534563	0.1	-	72	35.4242	37
ARC_ART_c17561	128	EEC77998	0.0	GO:0003700	94	71.2478	35
ARC_ART_c17574	135	XP_002528060	0.0	-	70	39.2762	41
ARC_ART_c17588	109	XP_002536731	0.0	-	70	42.3578	34
ARC_ART_c17598	158	XP_001698580	0.6	-	57	33.4982	40
ARC_ART_c17618	120	XP_008353721	0.6	-	55	32.7278	45
ARC_ART_c17619	200	XP_009351044	0.0	-	75	78.5666	66
ARC_ART_c17633	104	XP_002505619	0.0	GO:0016020	79	43.8986	34
ARC_ART_c17642	111	XP_010027488	0.4	-	55	31.5722	36
ARC_ART_c17651	136	XP_010046113	0.8	-	50	32.7278	34

ARC_ART_c17667	176	XP_002983660	0.0	GO:0044249	76	51.2174	39
ARC_ART_c17680	169	XP_002464695	0.0	-	60	38.1206	43
ARC_ART_c17706	145	EEC76774	0.0	GO:0006810	75	47.3654	44
ARC_ART_c17715	222	YP_009105271	0.0	GO:0003723	72	76.2554	73
ARC_ART_c17719	138	AAL82216	0.0	GO:0016020	55	42.743	45
ARC_ART_c17727	162	XP_002538274	0.0	GO:0009451	76	55.0694	47
ARC_ART_c17728	264	XP_010458427	0.3	-	58	35.039	46
ARC_ART_c17740	153	XP_005645383	0.0	GO:0044267	77	58.9214	44
ARC_ART_c17759	102	XP_002537593	0.0	-	76	46.595	34
ARC_ART_c17761	164	XP_002953969	0.0	GO:0008152	72	57.7658	47
ARC_ART_c17794	161	XP_002966274	0.0	-	66	43.5134	50
ARC_ART_c17795	157	EAY84569	0.0	GO:0050660	96	95.5153	50
ARC_ART_c17805	178	XP_002536582	0.0	GO:0005215	86	58.5362	36
ARC_ART_c17826	261	XP_009362550	0.9	-	52	33.4982	53
ARC_ART_c17847	175	EXB92316	0.0	-	61	44.669	60
ARC_ART_c17892	204	CDP05391	0.0	-	60	37.7354	43
ARC_ART_c17896	121	XP_009350051	0.0	GO:0030529	72	43.8986	37
ARC_ART_c17908	149	XP_002452909	0.0	-	55	26.1794	34
ARC_ART_c17914	173	KIZ03279	0.0	GO:0019538	72	56.9954	50
ARC_ART_c17943	152	XP_002441599	0.7	-	58	30.8018	53
ARC_ART_c17952	207	YP_009057878	0.0	GO:0016779	73	38.5058	34
ARC_ART_c17987	106	EEC80776	0.0	GO:0044763	74	42.743	35
ARC_ART_c17993	145	XP_009351046	0.0	GO:0019538	97	65.855	34
ARC_ART_c17995	129	KJB14970	0.1	-	51	34.2686	39
ARC_ART_c17998	173	XP_006280112	0.0	GO:0005618	80	63.929	42
ARC_ART_c18009	105	CEG01178	0.5	-	58	33.113	34

ARC_ART_c18010	135	XP_002488904	0.0	GO:0046872	84	59.3066	45
ARC_ART_c18017	109	EEE52322	0.0	GO:0016020	80	55.4546	36
ARC_ART_c18027	132	XP_009386639	0.4	-	50	33.4982	38
ARC_ART_c18033	125	XP_010500539	0.0	-	81	56.225	38
ARC_ART_c18038	177	XP_004301745	0.4	-	48	33.113	58
ARC_ART_c18058	125	XP_002535421	0.0	GO:0055114	89	69.707	37
ARC_ART_c18080	170	KDO70915	0.0	GO:0044765	69	63.929	53
ARC_ART_c18095	120	EPS74494	0.0	-	68	48.1358	38
ARC_ART_c18122	133	XP_009370577	0.9	-	51	32.3426	41
ARC_ART_c18164	141	XP_010266773	0.0	GO:0043231	78	53.5286	37
ARC_ART_c18174	110	KIY99354	0.0	-	74	41.5874	35
ARC_ART_c18191	108	XP_002540074	0.0	GO:0000155	85	54.299	34
ARC_ART_c18217	118	EEC77111	0.0	GO:0050896	77	45.0542	36
ARC_ART_c18223	105	CDX77158	0.6	-	58	32.7278	34
ARC_ART_c18237	129	XP_006577564	0.3	-	55	33.4982	34
ARC_ART_c18272	105	EEC75666	0.0	-	68	39.2762	35
ARC_ART_c18274	264	KIZ02967	0.2	-	46	35.039	88
ARC_ART_c18304	142	Q42662	0.4	-	58	33.8834	43
ARC_ART_c18324	124	CAA03961	0.0	GO:0009536	70	41.9726	40
ARC_ART_c18349	111	EDQ48453	0.0	-	62	43.5134	37
ARC_ART_c18354	155	BAA98166	0.0	-	52	36.965	44
ARC_ART_c18369	157	XP_009420185	0.4	-	53	33.4982	45

ARC_ART_c18400	114	EPS72616	0.5	-	61	33.113	34
ARC_ART_c18406	121	EEE52319	0.0	GO:0006378	100	82.4185	39
ARC_ART_c18407	137	EPS74505	0.0	-	71	46.595	39
ARC_ART_c18421	170	XP_010482466	0.2	-	53	34.6538	52
ARC_ART_c18424	247	EPS74505	0.0	-	64	38.891	39
ARC_ART_c18436	123	XP_002536716	0.7	-	58	31.187	36
ARC_ART_c18481	176	ABN50032	0.0	-	55	42.743	54
ARC_ART_c18498	172	XP_002536967	0.0	GO:0009987	58	42.3578	55
ARC_ART_c18506	108	EEE52320	0.0	GO:0055085	97	71.2478	36
ARC_ART_c18513	269	XP_009350076	0.0	GO:0008270	83	127.487	81
ARC_ART_c18518	153	XP_002536999	0.0	-	71	49.2914	52
ARC_ART_c18569	215	BAJ11784	0.0	-	73	43.1282	38
ARC_ART_c18572	182	XP_007099639	0.0	GO:0060003	75	68.9366	54
ARC_ART_c18580	160	NP_680210	0.0	GO:0006412	61	50.8322	57
ARC_ART_c18594	229	XP_003064804	0.0	-	60	48.9062	60
ARC_ART_c18605	172	XP_001764544	0.3	-	62	34.2686	37
ARC_ART_c18630	151	XP_002539738	0.0	GO:0050794	75	48.9062	40
ARC_ART_c18631	107	XP_002958632	0.5	-	60	33.113	35
ARC_ART_c18651	105	XP_002536832	0.0	GO:0055114	91	57.3806	34
ARC_ART_c18652	191	XP_002535893	0.0	GO:0016020	87	87.0409	63
ARC_ART_c18653	155	XP_002536045	0.1	-	61	35.039	39
ARC_ART_c18664	162	XP_008366766	0.4	-	50	31.5722	36
ARC_ART_c18675	255	XP_009351044	0.0	-	75	83.5741	60
ARC_ART_c18688	139	XP_002538546	0.6	-	61	32.7278	34
ARC_ART_c18711	216	XP_002538423	0.0	-	67	45.8246	53

ARC_ART_c18719	158	XP_006403748	0.1	-	54	35.039	42
ARC_ART_c18731	140	XP_002962254	0.0	-	82	38.1206	41
ARC_ART_c18748	130	XP_010942302	0.0	GO:0003824	66	56.225	42
ARC_ART_c18759	123	XP_002538205	0.0	GO:0071704	76	43.5134	34
ARC_ART_c18763	169	ABB89020	0.0	-	57	53.5286	54
ARC_ART_c18779	110	XP_005852165	0.3	-	69	33.8834	36
ARC_ART_c18789	214	XP_010911665	0.0	GO:0043229	87	54.6842	47
ARC_ART_c18794	120	EEC80862	0.0	GO:0006184	92	68.1662	39
ARC_ART_c18795	192	XP_007132740	0.7	-	50	31.187	36
ARC_ART_c18814	132	XP_011014727	0.0	GO:0005737	97	76.6406	44
ARC_ART_c18824	158	XP_009106733	0.0	GO:0009414	78	66.6254	50
ARC_ART_c18839	210	XP_002539855	0.2	-	59	34.6538	37
ARC_ART_c18900	118	XP_011015256	0.1	-	58	35.039	39
ARC_ART_c18911	117	EEC77966	0.3	-	58	33.8834	34
ARC_ART_c18912	128	BAI58870	0.0	GO:0003824	61	47.3654	42
ARC_ART_c18921	107	XP_002952441	0.0	-	68	43.1282	35
ARC_ART_c18926	116	XP_002508933	0.0	-	48	36.965	39
ARC_ART_c18931	163	XP_002537659	0.0	-	62	51.2174	53
ARC_ART_c18945	173	EEE67873	0.0	GO:0016740	91	93.2041	57
ARC_ART_c18954	184	XP_009350062	0.0	-	84	65.855	44
ARC_ART_c18959	232	XP_011045140	0.3	-	47	34.6538	61
ARC_ART_c18964	164	XP_006300670	0.8	-	55	33.113	40
ARC_ART_c18971	105	XP_002535815	0.0	GO:0008152	82	53.1434	34
ARC_ART_c18977	133	XP_011016264	0.0	-	67	37.7354	43
ARC_ART_c19020	124	XP_008810175	0.7	-	54	32.3426	37
ARC_ART_c19025	124	EAY95405	0.0	GO:0009941	85	70.8626	40
ARC_ART_c19030	152	EEE51040	0.0	GO:0006259	65	43.8986	41

ARC_ART_c19043	202	CDY45547	0.0	-	60	36.965	40
ARC_ART_c19058	339	EXC01915	0.0	-	52	37.3502	51
ARC_ART_c19064	115	XP_004253362	0.0	GO:0046961	92	68.9366	38
ARC_ART_c19066	141	XP_006282864	0.3	-	60	33.4982	35
ARC_ART_c19077	189	XP_005644366	0.5	-	57	33.4982	40
ARC_ART_c19096	149	XP_002540074	0.0	GO:0004871	77	52.7582	36
ARC_ART_c19109	142	KIY96160	0.0	GO:0071704	75	57.3806	44
ARC_ART_c19116	135	XP_008246471	0.1	-	60	35.039	38
ARC_ART_c19157	129	KFM27113	0.0	-	64	40.4318	39
ARC_ART_c19164	128	XP_002969533	0.0	GO:0019538	88	59.6918	36
ARC_ART_c19231	130	XP_002505274	0.0	-	60	41.9726	41
ARC_ART_c19336	223	YP_001152204	0.0	GO:0009536	76	57.3806	39
ARC_ART_c19339	239	EPS70027	0.2	-	58	33.4982	34
ARC_ART_c19372	199	KEH28415	0.6	-	58	33.8834	34
ARC_ART_c19388	201	YP_001152204	0.0	-	58	35.039	46
ARC_ART_c19418	226	KEH17697	0.0	-	70	40.817	34
ARC_ART_c19425	167	XP_003608262	0.0	-	57	41.9726	54
ARC_ART_c19445	169	AGC78943	0.0	-	56	36.5798	55
ARC_ART_c19476	122	KFM28548	0.0	GO:0016740	70	47.3654	40
ARC_ART_c19479	117	XP_002537280	0.0	-	69	43.1282	39
ARC_ART_c19480	122	EEC74306	0.0	GO:0016020	92	43.1282	40
ARC_ART_c19483	107	EEE67873	0.0	-	73	38.891	34
ARC_ART_c19485	121	XP_002538492	0.0	-	63	48.1358	38
ARC_ART_c19490	138	XP_001787129	0.0	GO:0006810	74	54.6842	39
ARC_ART_c19493	142	XP_008447683	0.0	-	58	37.7354	36
ARC_ART_c19507	182	KEH43224	0.0	-	59	36.965	57
ARC_ART_c19511	138	XP_004253383	0.0	-	93	77.411	45
ARC_ART_c19523	131	XP_010911593	0.0	-	86	70.8626	43

ARC_ART_c19529	129	XP_002535010	0.0	GO:0004871	78	62.7734	42
ARC_ART_c19533	167	KFM26565	0.0	-	60	37.7354	40
ARC_ART_c19546	160	EEE60756	0.0	GO:0055114	100	87.8113	44
ARC_ART_c19550	125	XP_002537336	0.0	-	63	39.6614	41
ARC_ART_c19555	190	KIY94764	0.0	GO:0006200	79	83.5741	62
ARC_ART_c19563	174	XP_002466670	0.4	-	60	33.8834	35
ARC_ART_c19564	186	XP_001758664	0.0	-	58	34.2686	39
ARC_ART_c19571	132	EAY76207	0.7	-	53	32.7278	45
ARC_ART_c19573	169	XP_010497721	1.0	-	54	31.5722	50
ARC_ART_c19574	124	KJB46013	0.9	-	62	31.9574	37
ARC_ART_c19575	117	XP_002969860	0.0	-	66	38.1206	39
ARC_ART_c19577	106	XP_009350805	0.0	GO:0048731	85	57.7658	35
ARC_ART_c19579	283	XP_008449136	0.0	GO:0005488	68	82.0333	74
ARC_ART_c19580	247	XP_002535087	0.3	-	62	33.113	40
ARC_ART_c19585	182	XP_001763917	0.0	-	64	39.2762	48
ARC_ART_c19588	141	XP_002948128	0.4	-	62	31.5722	37
ARC_ART_c19592	248	EMS45826	0.0	GO:0005829	66	80.8777	80
ARC_ART_c19610	169	XP_007132417	0.0	GO:0050662	57	46.9802	63
ARC_ART_c19614	125	EEC78702	0.0	GO:0015408	85	59.3066	41
ARC_ART_c19621	127	XP_002538475	0.0	GO:0009536	85	69.707	41
ARC_ART_c19651	128	XP_002536174	0.0	GO:0006313	80	58.5362	41
ARC_ART_c19652	170	CBI39990	0.0	GO:0005622	83	60.4622	43
ARC_ART_c19667	154	KIZ03401	0.0	GO:0016627	62	44.2838	50
ARC_ART_c19677	117	XP_003064457	0.0	GO:0047547	85	58.151	34
ARC_ART_c19679	104	KFM27553	0.0	-	55	38.1206	34
ARC_ART_c19682	252	XP_008802534	0.0	-	58	40.0466	51
ARC_ART_c19687	183	CCO20723	0.1	-	65	35.4242	38

ARC_ART_c19693	147	XP_006852858	0.0	GO:0016811	67	49.2914	46
ARC_ART_c19695	169	XP_004962577	0.7	-	50	32.7278	58
ARC_ART_c19696	122	ACU23330	0.0	GO:0045239	80	49.6766	36
ARC_ART_c19698	130	EEC72170	0.0	-	69	40.817	43
ARC_ART_c19700	125	EMT23426	0.0	GO:0016772	73	46.2098	34
ARC_ART_c19702	144	NP_001066070	0.5	-	48	33.4982	39
ARC_ART_c19703	125	XP_002540234	0.0	-	70	48.521	41
ARC_ART_c19704	139	XP_002536815	0.1	-	56	34.2686	48
ARC_ART_c19708	105	CCO14606	0.1	-	61	35.4242	34
ARC_ART_c19710	231	CDX70684	0.2	-	55	35.8094	45
ARC_ART_c19719	127	XP_009405183	0.2	-	65	34.2686	38
ARC_ART_c19722	151	XP_004494951	0.0	GO:0016787	71	50.0618	39
ARC_ART_c19740	253	XP_010911627	0.0	GO:0071704	65	75.485	82
ARC_ART_c19742	134	XP_002954766	0.0	GO:0005737	73	58.5362	42
ARC_ART_c19762	237	XP_002535069	0.0	-	67	43.8986	49
ARC_ART_c19766	110	XP_008344505	0.0	-	65	36.965	35
ARC_ART_c19772	309	XP_002965213	0.0	GO:0005737	67	67.0106	58
ARC_ART_c19777	131	AFA36508	0.0	GO:0044763	69	45.8246	42
ARC_ART_c19779	174	XP_001418570	0.0	-	62	42.3578	53
ARC_ART_c19783	113	EMT00267	0.0	GO:0046148	78	55.4546	37
ARC_ART_c19800	102	XP_005643475	0.0	-	70	36.5798	34
ARC_ART_c19802	147	EEC81066	0.1	-	58	36.5798	43
ARC_ART_c19810	166	XP_001417572	0.0	GO:0006457	92	91.6633	54
ARC_ART_c19813	140	XP_002500649	0.0	-	70	45.0542	47
ARC_ART_c19814	107	EMT18568	0.0	GO:0016787	76	44.2838	34
ARC_ART_c19818	133	XP_010051609	0.1	-	60	35.039	41

ARC_ART_c19820	191	XP_011016736	0.0	-	60	42.743	60
ARC_ART_c19829	129	EEE68528	0.0	GO:0055085	95	91.2781	43
ARC_ART_c19839	118	XP_003060526	0.0	-	67	38.891	37
ARC_ART_c19840	144	XP_003055117	0.0	-	63	41.2022	47
ARC_ART_c19850	156	XP_009350806	0.0	GO:0030288	91	82.0333	45
ARC_ART_c19861	267	XP_003591472	0.0	GO:0006979	75	78.1814	61
ARC_ART_c19877	151	XP_003058549	0.1	-	61	35.4242	39
ARC_ART_c19892	180	XP_002538735	0.0	-	66	38.1206	39
ARC_ART_c19898	171	XP_005844939	0.6	-	55	32.7278	34
ARC_ART_c19899	186	XP_010044732	0.6	-	52	33.113	50
ARC_ART_c19902	143	XP_001786859	0.1	-	76	35.039	34
ARC_ART_c19903	178	EEC70905	0.0	GO:0044763	74	62.3882	51
ARC_ART_c19904	119	XP_002466135	1.0	-	55	32.3426	34
ARC_ART_c19906	262	EEC67123	0.0	GO:0009987	57	42.3578	57
ARC_ART_c19909	106	XP_002539123	0.1	-	70	33.113	34
ARC_ART_c19922	158	XP_002540576	0.0	-	76	40.817	34
ARC_ART_c19932	120	KDD77116	0.0	GO:0042558	85	60.077	40
ARC_ART_c19940	138	BAJ21440	0.0	GO:0003746	95	94.7449	46
ARC_ART_c19941	220	XP_002535385	0.5	-	45	23.8682	53
ARC_ART_c19952	160	XP_002505348	0.0	-	67	48.521	43
ARC_ART_c19954	128	XP_006448547	0.0	GO:0006541	92	67.0106	39
ARC_ART_c19968	189	XP_008453702	0.4	-	51	34.2686	45
ARC_ART_c19971	190	XP_002539079	0.0	GO:0016836	93	115.546	63
ARC_ART_c19972	129	CCO18857	0.5	-	61	32.7278	36
ARC_ART_c19973	207	XP_001765555	0.0	-	50	38.891	81
ARC_ART_c19978	117	KEH15439	0.0	GO:0004735	80	52.373	35
ARC_ART_c19991	131	XP_009350054	0.0	GO:0005737	86	69.707	43
ARC_ART_c20010	189	KDD73869	0.0	GO:0008750	81	68.5514	48

ARC_ART_c20014	138	XP_005650418	0.0	GO:0009450	71	55.4546	45
ARC_ART_c20015	162	XP_010262181	0.0	-	63	40.0466	49
ARC_ART_c20026	115	EEE52323	0.1	-	58	35.039	36
ARC_ART_c20030	163	AAQ15121	0.0	-	52	39.2762	57
ARC_ART_c20034	196	EYU25721	0.0	GO:0009873	80	69.3218	42
ARC_ART_c20049	187	XP_006401145	0.0	GO:0009507	70	67.781	57
ARC_ART_c20064	118	XP_002537447	0.0	-	78	45.4394	37
ARC_ART_c20068	184	XP_002959989	0.4	-	50	33.8834	53
ARC_ART_c20070	112	DAA36438	0.8	-	50	31.5722	34
ARC_ART_c20074	121	KDP40214	0.0	-	65	43.1282	43
ARC_ART_c20079	217	KDD76578	0.0	GO:0044763	56	60.4622	72
ARC_ART_c20080	118	XP_011015250	0.0	-	71	48.521	39
ARC_ART_c20081	114	XP_005843931	0.0	GO:0009451	83	56.6102	37
ARC_ART_c20091	148	KFM27415	0.0	GO:0009570	63	46.595	55
ARC_ART_c20095	124	XP_002507787	0.0	GO:0008152	67	42.743	37
ARC_ART_c20097	199	XP_002536181	0.0	GO:0004871	70	49.6766	44
ARC_ART_c20101	108	EAY84177	0.1	-	75	34.6538	37
ARC_ART_c20118	158	XP_006360793	0.3	-	50	34.2686	48
ARC_ART_c20120	175	XP_002535368	0.0	-	54	38.1206	48
ARC_ART_c20123	169	XP_002535676	0.0	GO:0007165	69	53.1434	46
ARC_ART_c20129	139	EDQ49166	0.0	-	76	50.447	42
ARC_ART_c20137	107	XP_002960029	0.0	GO:0046912	85	53.1434	34
ARC_ART_c20145	171	XP_011016757	0.0	GO:0009735	82	82.4185	56
ARC_ART_c20147	223	EDQ48498	0.0	-	77	100.138	74

ARC_ART_c20148	177	CDY26051	0.3	-	46	34.2686	45
ARC_ART_c20156	279	XP_005649800	0.0	GO:0005975	85	65.4698	55
ARC_ART_c20160	112	CEF99626	0.1	-	61	35.4242	34
ARC_ART_c20167	139	XP_008445382	0.0	GO:0016772	90	70.4774	41
ARC_ART_c20168	127	P53385	0.0	GO:0019557	90	74.3294	41
ARC_ART_c20173	153	XP_001695791	0.3	-	61	33.4982	36
ARC_ART_c20176	132	KDD72406	0.0	GO:0008152	78	50.8322	37
ARC_ART_c20178	139	XP_008337406	0.9	-	59	31.9574	42
ARC_ART_c20180	113	XP_002534739	0.0	-	64	41.2022	37
ARC_ART_c20181	163	XP_009351044	0.0	-	84	68.1662	44
ARC_ART_c20195	180	EDQ48547	0.0	-	66	55.8398	59
ARC_ART_c20196	220	XP_009391806	0.0	-	69	53.1434	53
ARC_ART_c20207	155	XP_001770083	0.8	-	47	33.113	34
ARC_ART_c20210	182	EEC68416	0.6	-	67	33.113	56
ARC_ART_c20211	219	XP_011016757	0.0	GO:0070011	70	64.3142	71
ARC_ART_c20212	177	XP_002536290	0.0	-	59	45.0542	47
ARC_ART_c20222	106	XP_004964433	0.0	-	65	36.5798	38
ARC_ART_c20230	129	XP_002502394	0.0	GO:0004519	73	52.7582	41
ARC_ART_c20239	188	XP_002955541	0.0	GO:0044699	61	53.9138	57
ARC_ART_c20240	150	ABA06483	0.1	-	57	35.039	40
ARC_ART_c20246	105	XP_002537184	0.6	-	67	32.3426	34
ARC_ART_c20257	212	AEI00885	0.0	GO:0004129	85	96.2857	70
ARC_ART_c20258	207	EEE52322	0.0	GO:0016020	98	66.2402	52
ARC_ART_c20265	218	EEC77111	0.0	GO:0016757	77	50.8322	35
ARC_ART_c20275	261	XP_002956187	0.2	-	56	35.4242	48

ARC_ART_c20281	138	XP_001761707	0.0	GO:0016787	68	48.1358	44
ARC_ART_c20292	112	XP_002539028	0.0	GO:0008880	97	74.7146	37
ARC_ART_c20300	133	XP_011015254	0.0	-	66	44.2838	39
ARC_ART_c20304	202	EEC80862	0.0	GO:0009536	96	123.635	66
ARC_ART_c20306	168	XP_008809863	0.6	-	47	33.4982	48
ARC_ART_c20309	136	XP_010025808	0.4	-	51	33.4982	43
ARC_ART_c20313	123	XP_002537510	0.0	GO:0008152	76	53.5286	39
ARC_ART_c20315	141	EEC84194	0.0	GO:0006457	84	76.6406	46
ARC_ART_c20329	148	XP_001421754	0.0	GO:0009536	74	54.6842	43
ARC_ART_c20337	121	XP_002985058	0.0	GO:0005975	61	43.1282	39
ARC_ART_c20346	119	P53385	0.0	GO:0019557	92	77.0258	39
ARC_ART_c20354	196	KIY93756	0.0	GO:0098655	70	73.1738	64
ARC_ART_c20355	141	ABY77748	0.0	GO:0009561	66	52.7582	48
ARC_ART_c20365	214	XP_001786576	0.0	-	55	38.891	56
ARC_ART_c20369	108	ABR17881	0.1	-	62	35.039	35
ARC_ART_c20370	121	XP_002535747	0.0	-	70	46.595	37
ARC_ART_c20384	189	XP_002960003	0.0	GO:0008152	86	82.0333	61
ARC_ART_c20397	254	XP_002536999	0.0	-	57	47.3654	69
ARC_ART_c20414	129	XP_001755830	0.0	GO:0009750	85	66.6254	40
ARC_ART_c20417	109	XP_005650043	0.0	GO:0005737	80	56.9954	36
ARC_ART_c20429	142	CAH67737	0.3	-	55	34.2686	47
ARC_ART_c20442	177	XP_002536145	0.0	-	75	81.2629	57
ARC_ART_c20445	128	XP_011077990	0.4	-	50	33.4982	38

ARC_ART_c20446	176	XP_011098802	0.2	-	57	35.039	45
ARC_ART_c20450	145	XP_002540084	0.0	-	56	49.2914	48
ARC_ART_c20463	105	Q9FVG8	0.0	-	65	39.2762	35
ARC_ART_c20475	175	XP_002538281	0.0	-	79	74.7146	58
ARC_ART_c20493	177	XP_003058412	0.0	GO:0051536	67	44.669	58
ARC_ART_c20499	207	CEF98862	0.0	GO:0016616	65	50.0618	46
ARC_ART_c20502	102	XP_002536199	0.1	-	58	35.039	34
ARC_ART_c20514	133	XP_002535792	0.0	GO:0008675	88	59.6918	34
ARC_ART_c20516	153	XP_002538998	0.0	GO:0016740	62	40.4318	51
ARC_ART_c20528	237	YP_009105593	0.0	-	62	40.817	40
ARC_ART_c20536	105	KIZ00647	0.0	-	76	51.2174	34
ARC_ART_c20552	148	XP_002537860	0.0	GO:1901363	67	58.5362	46
ARC_ART_c20591	126	XP_008440426	0.8	-	43	32.7278	44
ARC_ART_c20601	212	EEC81066	0.0	-	58	48.1358	68
ARC_ART_c20607	139	XP_002535920	0.0	GO:0032450	86	82.0333	45
ARC_ART_c20608	125	XP_002456330	0.0	-	58	38.5058	39
ARC_ART_c20614	123	XP_003544879	0.2	-	55	33.8834	40
ARC_ART_c20624	146	KEH15415	0.0	GO:0003677	75	63.5438	48
ARC_ART_c20631	132	XP_006843780	0.0	GO:0005975	60	45.8246	43
ARC_ART_c20633	148	XP_002536715	0.0	-	63	47.3654	49
ARC_ART_c20635	105	KJB75687	0.0	GO:0016740	76	49.2914	34
ARC_ART_c20645	154	KIY99730	0.1	-	63	35.039	41
ARC_ART_c20653	111	AAF16526	0.0	GO:0007010	88	59.3066	35

ARC_ART_c20655	131	ERN02635	0.0	GO:0046686	87	74.3294	41
ARC_ART_c20671	119	XP_002535367	0.1	-	71	35.8094	35
ARC_ART_c20677	104	XP_011016735	0.0	-	79	44.2838	34
ARC_ART_c20682	111	KFM27055	0.0	GO:0016740	69	40.817	36
ARC_ART_c20685	189	KGN56358	0.8	-	52	33.4982	44
ARC_ART_c20694	121	CBI25284	0.7	-	55	31.5722	38
ARC_ART_c20703	129	CDX92587	0.8	-	53	32.7278	45
ARC_ART_c20707	136	XP_009391385	0.0	-	67	40.4318	34
ARC_ART_c20747	122	XP_002535390	0.0	-	77	52.7582	40
ARC_ART_c20764	138	KFM27087	0.0	GO:0042558	80	70.0922	46
ARC_ART_c20778	242	XP_008792473	0.1	-	64	36.965	34
ARC_ART_c20782	159	XP_002537714	0.8	-	64	32.7278	34
ARC_ART_c20808	120	XP_001698486	1.0	-	53	32.3426	39
ARC_ART_c20820	193	XP_002984596	0.0	-	72	38.891	40
ARC_ART_c20824	117	DAA38917	0.0	-	64	38.891	39
ARC_ART_c20843	235	KGN60194	0.3	-	50	35.039	76
ARC_ART_c20858	234	CCO16715	0.0	-	58	43.1282	56
ARC_ART_c20872	183	XP_006650366	0.0	GO:0005737	66	66.2402	59
ARC_ART_c20880	112	XP_001418781	0.0	GO:0016020	75	45.8246	36
ARC_ART_c20883	120	XP_002534657	0.0	GO:0015109	89	73.9442	39
ARC_ART_c20886	250	XP_002539420	0.0	GO:0016740	69	76.2554	69
ARC_ART_c20887	167	AAZ99674	0.0	GO:0006629	83	70.8626	49
ARC_ART_c20893	113	ABK96687	0.0	-	69	35.8094	36
ARC_ART_c20908	127	XP_005645067	0.0	GO:0006412	71	53.1434	42
ARC_ART_c20918	111	XP_003078729	0.0	GO:0008184	80	53.9138	36
ARC_ART_c20920	142	EEE54984	0.0	GO:0005737	95	58.9214	47

ARC_ART_c20922	128	KDD74062	0.0	GO:0051287	88	69.3218	43
ARC_ART_c20943	166	XP_002503674	0.0	GO:0005488	70	69.707	51
ARC_ART_c20948	158	XP_005846776	0.1	-	67	35.039	37
ARC_ART_c20956	142	XP_002538871	0.0	-	61	43.1282	44
ARC_ART_c20957	151	XP_003062944	0.0	GO:0009228	80	43.1282	35
ARC_ART_c20963	104	XP_002535042	0.0	GO:0008236	82	62.003	34
ARC_ART_c20965	112	XP_001786668	0.6	-	66	31.9574	36
ARC_ART_c20988	181	XP_004506833	0.0	-	56	39.6614	58
ARC_ART_c20993	185	KDD72971	0.0	GO:0006568	70	49.2914	40
ARC_ART_c21025	113	XP_002264829	0.2	-	67	33.8834	37
ARC_ART_c21042	138	XP_002538815	0.0	-	65	36.965	43
ARC_ART_c21063	227	XP_005842602	0.0	-	62	57.3806	61
ARC_ART_c21091	110	XP_001770921	0.1	-	63	35.039	36
ARC_ART_c21099	136	XP_002534501	0.0	GO:0032440	80	58.5362	42
ARC_ART_c21106	158	EEC68421	0.1	-	61	35.4242	42
ARC_ART_c21109	150	ERN13812	0.0	-	66	43.8986	45
ARC_ART_c21113	154	ACJ84509	0.0	GO:0003723	80	77.0258	51
ARC_ART_c21121	184	XP_002535088	0.0	-	71	59.6918	60
ARC_ART_c21124	113	CCO17918	0.7	-	61	32.7278	36
ARC_ART_c21133	146	XP_001752199	0.0	GO:0020037	83	70.4774	48
ARC_ART_c21139	123	XP_002536403	0.0	-	65	36.1946	35
ARC_ART_c21162	137	XP_002534885	0.0	-	69	43.5134	39
ARC_ART_c21170	158	XP_002538477	0.0	-	77	55.0694	49
ARC_ART_c21171	184	XP_002540589	0.0	GO:0055114	85	93.9745	60
ARC_ART_c21188	134	CAN71629	0.1	-	55	36.1946	45
ARC_ART_c21208	114	KFM27087	0.0	-	60	41.2022	38

ARC_ART_c21214	123	XP_007220763	0.0	GO:0016874	73	41.9726	38
ARC_ART_c21231	128	AJV88588	0.0	-	63	42.3578	41
ARC_ART_c21242	122	XP_002538154	0.0	GO:0009060	90	72.4034	40
ARC_ART_c21253	145	EXB97656	0.2	-	59	32.7278	37
ARC_ART_c21259	126	XP_002538134	0.0	GO:0016301	87	59.3066	39
ARC_ART_c21269	134	XP_002536193	0.0	GO:0015416	100	88.1965	44
ARC_ART_c21284	167	CDO98000	1.0	-	44	31.187	38
ARC_ART_c21288	146	AFK34317	0.0	-	67	36.5798	34
ARC_ART_c21289	104	XP_001775725	0.0	GO:0016485	97	69.3218	34
ARC_ART_c21291	155	CAN72098	0.0	-	63	36.965	38
ARC_ART_c21305	119	EAZ12893	0.9	-	50	32.3426	42
ARC_ART_c21306	131	XP_001695168	0.0	GO:0043169	71	47.3654	39
ARC_ART_c21318	202	CEF97351	0.9	-	54	33.4982	59
ARC_ART_c21327	132	XP_001416295	0.0	GO:0005739	86	70.0922	43
ARC_ART_c21330	142	XP_004962246	0.1	-	68	35.8094	35
ARC_ART_c21342	190	XP_005646805	0.0	GO:0016491	61	46.595	52
ARC_ART_c21343	144	EEC70905	0.0	GO:0044763	73	50.0618	38
ARC_ART_c21348	116	XP_006282546	0.2	-	61	34.2686	36
ARC_ART_c21350	108	XP_002538274	0.1	-	67	33.8834	34
ARC_ART_c21352	117	XP_001420863	0.0	GO:0005737	75	48.1358	37
ARC_ART_c21364	144	KIZ06381	0.1	-	68	35.8094	35
ARC_ART_c21367	133	XP_004981117	0.0	-	63	39.2762	41
ARC_ART_c21373	162	XP_003600627	0.0	GO:0009735	75	56.6102	44
ARC_ART_c21387	146	AAG13516	1.0	-	60	32.3426	40
ARC_ART_c21395	120	BAK00178	0.0	GO:0003857	84	64.3142	39
ARC_ART_c21401	145	XP_001694415	0.0	GO:0003677	72	51.6026	36
ARC_ART_c21418	163	XP_001690955	0.0	GO:0055114	80	67.781	55
ARC_ART_c21422	178	AFJ52927	0.3	-	50	34.2686	63
ARC_ART_c21427	161	KGN61807	0.2	-	56	32.7278	41
ARC_ART_c21430	143	XP_008382379	0.0	GO:0005829	80	51.2174	41

ARC_ART_c21435	134	EDQ48467	0.1	-	55	34.6538	49
ARC_ART_c21439	123	EPS59581	0.0	-	51	36.1946	37
ARC_ART_c21444	126	XP_003081716	0.0	GO:0008094	85	56.6102	42
ARC_ART_c21455	108	XP_002537877	0.0	GO:0008237	74	44.669	35
ARC_ART_c21456	172	XP_011016696	0.0	GO:0016020	67	51.6026	58
ARC_ART_c21458	110	EYU18108	0.0	GO:0003824	63	42.743	38
ARC_ART_c21461	134	XP_002535814	0.0	-	71	51.6026	42
ARC_ART_c21480	174	NP_001167840	0.8	-	45	33.113	48
ARC_ART_c21490	119	ADB85507	0.0	GO:0044723	78	50.8322	38
ARC_ART_c21503	127	ABU94808	0.1	-	56	32.7278	39
ARC_ART_c21506	153	XP_011468888	0.5	-	58	33.4982	34
ARC_ART_c21507	116	XP_003074121	0.0	GO:0008976	82	50.0618	34
ARC_ART_c21515	117	KDO52721	0.0	GO:0005507	92	70.4774	39
ARC_ART_c21519	173	XP_002537713	0.0	-	76	63.1586	50
ARC_ART_c21523	149	XP_002536583	0.1	-	60	35.8094	48
ARC_ART_c21526	181	XP_002535949	0.0	GO:1901363	91	102.064	60
ARC_ART_c21533	161	AAX18752	0.0	-	56	36.5798	51
ARC_ART_c21543	106	XP_002959312	0.0	GO:0008152	76	46.2098	34
ARC_ART_c21545	267	XP_008442218	0.0	-	67	38.891	40
ARC_ART_c21550	147	XP_009391487	0.1	-	51	35.039	49
ARC_ART_c21556	124	XP_002534959	0.0	GO:0008233	87	69.3218	40
ARC_ART_c21558	193	KDO69764	0.8	-	61	31.187	36
ARC_ART_c21562	146	EMT00318	0.0	-	78	63.929	41
ARC_ART_c21567	159	XP_002536203	0.0	-	63	49.6766	52
ARC_ART_c21578	161	XP_002535661	0.0	-	70	40.817	37
ARC_ART_c21582	138	EPS70706	0.2	-	53	33.8834	43
ARC_ART_c21585	150	AEB78732	0.0	GO:0005774	86	74.3294	43
ARC_ART_c21589	170	XP_002960015	0.0	GO:0008750	79	71.633	44
ARC_ART_c21598	173	ABH09321	0.0	-	65	40.4318	44

ARC_ART_c21624	128	XP_002539997	0.0	-	65	40.0466	35
ARC_ART_c21628	130	XP_002532585	0.3	-	56	34.2686	39
ARC_ART_c21640	120	KEH15415	0.0	-	66	37.3502	36
ARC_ART_c21649	107	XP_002537471	0.1	-	57	34.6538	35
ARC_ART_c21651	139	XP_005850617	0.0	-	68	36.5798	35
ARC_ART_c21670	111	XP_007048914	0.1	-	67	35.8094	34
ARC_ART_c21671	136	KIY92308	0.9	-	60	30.0314	35
ARC_ART_c21674	143	EAZ37385	0.0	-	74	45.0542	47
ARC_ART_c21677	158	EEE68528	0.0	GO:0055085	98	101.679	52
ARC_ART_c21683	254	XP_004499749	0.0	GO:0009536	78	91.6633	66
ARC_ART_c21686	270	XP_009142483	0.1	-	61	36.965	42
ARC_ART_c21694	112	XP_004253340	0.0	-	86	56.6102	36
ARC_ART_c21701	123	BAJ99459	0.0	GO:0050660	86	59.6918	36
ARC_ART_c21702	215	XP_002539327	0.0	GO:0008152	82	70.8626	50
ARC_ART_c21710	131	KGN47311	0.0	GO:0009451	72	47.3654	43
ARC_ART_c21713	125	XP_001689759	0.0	-	70	48.1358	41
ARC_ART_c21717	147	XP_007010544	0.0	GO:0016020	75	60.077	53
ARC_ART_c21721	158	CDX74434	0.4	-	53	33.4982	41
ARC_ART_c21732	130	XP_008651928	0.2	-	57	34.2686	38
ARC_ART_c21734	129	CCO16742	0.7	-	57	32.7278	35
ARC_ART_c21739	134	XP_002954941	0.2	-	52	34.6538	48
ARC_ART_c21740	107	EMS61245	0.6	-	58	32.3426	36
ARC_ART_c21777	167	XP_002540215	0.0	-	58	41.2022	46
ARC_ART_c21784	117	EAY84808	0.0	-	66	43.5134	39
ARC_ART_c21787	143	XP_002537159	0.0	-	85	46.2098	35

ARC_ART_c21794	154	XP_002535268	0.0	-	69	71.2478	49
ARC_ART_c21796	131	KDP20901	0.0	GO:0005829	79	59.6918	43
ARC_ART_c21812	216	XP_002951087	0.0	GO:0006629	88	85.1149	52
ARC_ART_c21836	107	XP_002968257	0.0	-	71	39.6614	35
ARC_ART_c21841	247	XP_002540458	0.0	-	54	44.2838	64
ARC_ART_c21842	135	KEH15262	0.0	GO:0009536	75	62.7734	45
ARC_ART_c21845	153	XP_002536223	0.0	-	73	49.2914	49
ARC_ART_c21856	123	XP_001692833	0.0	GO:0005737	75	48.9062	37
ARC_ART_c21858	196	XP_005850263	0.0	GO:0006066	96	94.7449	52
ARC_ART_c21860	116	XP_008466644	0.1	-	63	35.4242	38
ARC_ART_c21868	137	XP_002535367	0.0	-	56	37.7354	37
ARC_ART_c21871	107	CAB90608	0.0	GO:0071704	73	51.9878	34
ARC_ART_c21873	179	CAN69401	0.4	-	45	33.8834	59
ARC_ART_c21885	140	XP_002537571	0.0	-	63	46.9802	44
ARC_ART_c21891	131	XP_003064457	0.0	GO:0019543	76	63.5438	42
ARC_ART_c21902	133	XP_002540139	0.0	-	65	51.6026	44
ARC_ART_c21922	136	AAO42474	0.0	GO:0006869	71	58.9214	52
ARC_ART_c21930	118	XP_002535920	0.0	GO:0090599	77	49.2914	35
ARC_ART_c21934	212	BAJ86385	0.0	-	69	59.6918	59
ARC_ART_c21941	202	XP_002537713	0.0	-	74	58.5362	54
ARC_ART_c21947	107	XP_001786609	0.4	-	58	30.8018	34
ARC_ART_c21951	202	XP_002537348	0.0	-	82	55.8398	39
ARC_ART_c21964	149	XP_002446496	0.3	-	45	33.8834	44
ARC_ART_c21971	112	XP_005850567	0.0	-	63	35.4242	36
ARC_ART_c21972	104	XP_007014005	0.0	-	64	39.6614	34
ARC_ART_c21974	140	CDP11143	0.7	-	52	33.113	38
ARC_ART_c21984	111	NP_001159131	0.4	-	65	33.4982	35

ARC_ART_c21985	143	XP_002537784	0.0	GO:0016021	80	50.0618	47
ARC_ART_c21993	112	XP_009350076	0.0	GO:0005525	97	76.2554	37
ARC_ART_c21995	186	KFM29251	0.0	GO:0009507	66	56.9954	62
ARC_ART_c21999	164	EYU19642	0.0	-	60	38.1206	45
ARC_ART_c22000	151	XP_002539339	0.0	-	67	42.743	46
ARC_ART_c22014	188	EEC76404	0.0	-	75	72.7886	57
ARC_ART_c22022	166	XP_009350814	0.0	-	70	46.9802	40
ARC_ART_c22026	129	XP_005646096	0.1	-	70	35.8094	37
ARC_ART_c22036	125	KCW75591	0.0	-	65	40.4318	38
ARC_ART_c22048	139	XP_005646097	0.0	-	50	37.7354	48
ARC_ART_c22049	106	KDD75389	0.0	-	67	39.6614	34
ARC_ART_c22054	143	ABX00565	0.0	-	57	37.3502	47
ARC_ART_c22065	173	EMT23017	0.3	-	52	34.6538	42
ARC_ART_c22068	145	AEL29851	0.7	-	48	30.8018	47
ARC_ART_c22078	239	NP_001235796	0.6	-	50	33.8834	81
ARC_ART_c22080	151	XP_011016741	0.0	-	94	88.1965	50
ARC_ART_c22090	140	XP_002537519	0.0	GO:0008233	68	50.8322	44
ARC_ART_c22094	116	XP_002539813	0.0	GO:0000160	88	53.9138	36
ARC_ART_c22107	117	XP_009350052	0.0	GO:0044260	69	49.2914	39
ARC_ART_c22115	136	XP_001762999	0.0	GO:1901363	75	62.3882	44
ARC_ART_c22121	155	XP_002979709	0.1	-	62	35.039	35
ARC_ART_c22129	124	XP_002488870	0.0	-	97	77.0258	38
ARC_ART_c22133	186	Q4R0I0	0.0	-	67	39.2762	40
ARC_ART_c22153	191	XP_002536209	0.0	GO:0016020	80	87.4261	63
ARC_ART_c22154	103	XP_009350065	0.0	GO:0003735	100	68.5514	34
ARC_ART_c22173	191	XP_006580082	0.2	-	48	35.039	49
ARC_ART_c22215	142	XP_009350057	0.0	-	60	41.2022	40
ARC_ART_c22222	137	CCO18705	0.6	-	61	33.113	44
ARC_ART_c22227	186	XP_006299930	0.4	-	50	34.2686	55
ARC_ART_c22232	156	XP_002508595	0.0	-	59	42.3578	47

ARC_ART_c22244	142	KJB66163	0.8	-	60	32.7278	35
ARC_ART_c22262	163	ADD09599	0.0	-	59	35.4242	42
ARC_ART_c22265	142	XP_002951117	0.8	-	62	32.7278	37
ARC_ART_c22268	115	XP_002534891	0.9	-	58	30.4166	36
ARC_ART_c22269	229	XP_004968853	0.4	-	50	34.2686	38
ARC_ART_c22279	156	XP_009350054	0.0	GO:0005737	90	82.0333	51
ARC_ART_c22280	172	CBI21529	0.2	-	54	35.039	35
ARC_ART_c22286	125	XP_007146040	0.0	GO:0009086	90	79.7221	41
ARC_ART_c22313	206	XP_009350076	0.0	GO:0005525	86	118.627	68
ARC_ART_c22326	157	CDP03297	0.4	-	61	33.8834	39
ARC_ART_c22342	140	EEC77966	0.0	GO:0005524	86	71.633	45
ARC_ART_c22345	106	NP_001174008	0.4	-	61	31.187	34
ARC_ART_c22362	138	ADO16105	0.0	GO:0009735	75	61.6178	45
ARC_ART_c22371	117	XP_002539919	0.0	GO:0009987	74	42.743	35
ARC_ART_c22385	128	XP_006491765	0.3	-	58	33.113	43
ARC_ART_c22386	125	XP_004985104	0.0	GO:0004527	72	47.7506	40
ARC_ART_c22388	122	XP_002539627	0.0	GO:0055114	82	53.1434	40
ARC_ART_c22390	148	XP_002537783	0.0	-	59	38.891	49
ARC_ART_c22409	156	XP_004511756	0.0	-	62	37.3502	37
ARC_ART_c22425	155	AAF16526	0.0	GO:0006807	81	53.1434	38
ARC_ART_c22426	118	EEC79194	0.0	-	64	43.8986	39
ARC_ART_c22437	158	XP_002535074	0.0	GO:0000166	76	70.0922	50
ARC_ART_c22442	166	XP_011043155	0.0	-	69	43.8986	46
ARC_ART_c22510	153	XP_001417123	0.0	-	62	45.4394	51
ARC_ART_c22518	153	XP_002534653	0.0	-	70	51.2174	50
ARC_ART_c22553	154	XP_002953705	0.1	-	57	35.8094	42
ARC_ART_c22555	125	BAJ97253	0.0	-	69	50.0618	39

ARC_ART_c22559	123	XP_010437899	0.0	GO:0005829	78	56.6102	37
ARC_ART_c22563	268	KIZ07228	0.1	-	45	36.5798	51
ARC_ART_c22570	138	ACO88948	0.1	-	62	33.113	40
ARC_ART_c22576	167	XP_002536967	0.0	GO:0004871	77	70.0922	54
ARC_ART_c22585	109	EMT29677	0.0	-	51	36.5798	35
ARC_ART_c22596	165	XP_009383349	0.0	-	51	41.5874	52
ARC_ART_c22612	124	XP_002537217	0.0	GO:0003824	63	46.595	41
ARC_ART_c22624	106	XP_005644007	0.2	-	70	33.8834	34
ARC_ART_c22628	121	XP_010024975	0.0	-	68	40.817	41
ARC_ART_c22645	103	XP_011074822	0.0	GO:0015031	67	43.5134	34
ARC_ART_c22657	113	XP_002488904	0.0	GO:0003676	69	45.8246	36
ARC_ART_c22661	128	CEG00736	0.0	-	70	52.373	41
ARC_ART_c22662	113	XP_002530774	0.4	-	68	32.7278	35
ARC_ART_c22666	146	XP_003056436	0.0	GO:0008152	73	62.003	45
ARC_ART_c22671	156	XP_005846937	0.0	-	57	44.2838	49
ARC_ART_c22697	148	EEC76877	0.0	GO:0046961	80	69.3218	46
ARC_ART_c22698	133	EEE52322	0.0	GO:0016020	100	92.4337	44
ARC_ART_c22701	162	NP_050872	0.0	GO:0044763	71	50.0618	46
ARC_ART_c22709	131	NP_001147527	0.3	-	61	33.8834	42
ARC_ART_c22717	120	XP_002536208	0.0	-	69	37.3502	39
ARC_ART_c22727	106	XP_002539813	0.0	-	65	37.3502	35
ARC_ART_c22751	123	XP_008221830	0.1	-	56	35.039	41
ARC_ART_c22756	135	XP_002538319	0.0	GO:0007165	64	46.2098	45
ARC_ART_c22774	155	AAB22587	0.0	GO:0071704	80	80.4925	51
ARC_ART_c22825	108	XP_001786633	0.1	-	67	35.8094	34
ARC_ART_c22846	225	XP_002499638	0.0	GO:0004834	79	103.605	72
ARC_ART_c22848	166	EXC65265	0.0	-	64	45.4394	53
ARC_ART_c22868	115	BAA74947	0.0	-	65	38.891	35
ARC_ART_c22876	122	EDQ48693	0.4	-	61	33.4982	36
ARC_ART_c22914	119	XP_002537984	0.0	GO:0005975	89	68.9366	39

ARC_ART_c22923	150	XP_001768407	0.0	-	70	48.521	41
ARC_ART_c22947	156	NP_001172656	0.2	-	55	33.113	34
ARC_ART_c22948	145	XP_001416051	0.0	-	70	41.5874	34
ARC_ART_c22965	146	XP_006290943	0.0	GO:0008802	61	48.521	47
ARC_ART_c22971	130	XP_001760766	0.0	GO:0046872	72	49.2914	40
ARC_ART_c22989	147	XP_002499564	0.0	GO:0016491	77	44.669	36
ARC_ART_c22990	109	XP_002509137	0.0	GO:0008237	71	53.1434	35
ARC_ART_c22992	132	XP_006279312	0.0	-	95	84.3445	44
ARC_ART_c23010	180	XP_002488912	0.0	GO:0000166	73	55.4546	49
ARC_ART_c23014	209	XP_003060882	0.1	-	51	36.5798	56
ARC_ART_c23023	142	XP_001787071	0.4	-	65	33.113	40
ARC_ART_c23043	133	XP_010314956	0.0	-	63	36.1946	36
ARC_ART_c23052	107	XP_002535681	0.7	-	58	32.3426	34
ARC_ART_c23061	158	XP_002508254	0.0	GO:0016772	58	45.8246	50
ARC_ART_c23068	168	XP_009350808	0.0	-	71	51.9878	52
ARC_ART_c23082	152	XP_002437214	0.1	-	61	35.8094	36
ARC_ART_c23087	134	XP_002947189	0.0	GO:0005488	77	61.6178	44
ARC_ART_c23106	172	XP_005652258	0.0	-	64	37.3502	42
ARC_ART_c23145	108	XP_002446391	0.8	-	64	32.3426	37
ARC_ART_c23157	116	XP_002535015	0.0	-	67	37.3502	37
ARC_ART_c23177	139	KIZ01971	0.0	-	71	49.2914	46
ARC_ART_c23185	158	NP_001054097	0.1	-	55	35.4242	36
ARC_ART_c23186	126	ACS49419	0.0	GO:0016740	70	46.595	41
ARC_ART_c23191	198	XP_002535387	0.0	GO:0008152	80	59.3066	50
ARC_ART_c23192	110	KDO55195	0.7	-	50	31.9574	40
ARC_ART_c23198	130	XP_008451413	0.0	-	72	38.5058	36
ARC_ART_c23212	132	AAL05473	0.0	GO:0000015	79	53.1434	44

ARC_ART_c23215	140	EAZ37752	0.9	-	55	32.3426	36
ARC_ART_c23216	220	KEH15576	0.0	-	64	64.3142	70
ARC_ART_c23225	165	XP_002505390	0.0	-	68	39.6614	35
ARC_ART_c23230	244	XP_004491391	0.2	-	59	35.8094	42
ARC_ART_c23237	159	ABA98901	0.4	-	63	33.8834	38
ARC_ART_c23242	170	EEC77111	0.0	GO:0016020	64	45.4394	48
ARC_ART_c23248	169	XP_002539872	0.0	-	76	66.6254	56
ARC_ART_c23254	131	EAY88383	0.0	GO:0003824	62	41.9726	40
ARC_ART_c23255	123	XP_011461060	0.0	-	57	38.5058	40
ARC_ART_c23262	122	XP_007041448	0.1	-	74	34.6538	39
ARC_ART_c23265	144	XP_008359081	0.0	GO:0080041	91	88.5817	47
ARC_ART_c23291	135	XP_001769292	0.0	-	72	38.891	43
ARC_ART_c23306	124	CCO16830	0.0	GO:0044237	71	54.299	39
ARC_ART_c23322	149	XP_001696139	0.0	GO:0004124	83	74.3294	49
ARC_ART_c23325	106	XP_002539251	0.0	GO:0016740	77	46.2098	35
ARC_ART_c23334	105	KDD72292	0.0	GO:0000166	74	45.8246	35
ARC_ART_c23346	107	XP_002536199	0.0	GO:0009987	88	64.3142	34
ARC_ART_c23352	145	EMS50947	0.0	GO:0008237	87	71.2478	49
ARC_ART_c23360	131	XP_002536540	0.0	-	76	40.4318	42
ARC_ART_c23367	156	KJB70195	0.9	-	51	30.4166	47
ARC_ART_c23374	109	XP_010251849	0.0	-	60	39.2762	41
ARC_ART_c23393	245	XP_002536822	0.0	-	73	79.337	68
ARC_ART_c23421	165	XP_008793866	0.2	-	63	34.6538	49
ARC_ART_c23431	157	YP_008963667	0.0	GO:0004129	79	63.1586	48
ARC_ART_c23433	171	XP_005644659	0.0	GO:0006401	63	48.9062	55
ARC_ART_c23440	202	XP_001773470	0.0	GO:0016740	65	37.7354	44
ARC_ART_c23466	131	AEG75665	0.3	-	57	31.9574	40
ARC_ART_c23499	111	BAJ88559	0.0	GO:0009615	79	46.2098	34
ARC_ART_c23516	164	XP_002535215	0.0	GO:0043231	62	48.9062	50
ARC_ART_c23528	101	XP_001692598	0.0	GO:0004832	79	46.2098	34

ARC_ART_c23529	220	XP_003617050	0.0	GO:0003723	74	85.1149	71
ARC_ART_c23530	186	XP_002537896	1.0	-	52	32.7278	57
ARC_ART_c23540	104	XP_002536998	0.0	-	100	68.1662	34
ARC_ART_c23545	165	EEC68548	0.0	GO:0000166	74	65.0846	55
ARC_ART_c23551	119	XP_001757734	0.0	-	68	41.9726	38
ARC_ART_c23577	172	XP_002538023	0.0	-	77	49.2914	36
ARC_ART_c23614	146	EEC76878	0.0	GO:0015078	72	64.3142	55
ARC_ART_c23630	188	XP_010236684	0.1	-	45	36.5798	55
ARC_ART_c23632	159	KDD72606	0.0	GO:0006783	69	66.6254	49
ARC_ART_c23651	169	XP_001787018	0.0	-	60	40.817	53
ARC_ART_c23661	147	XP_002534861	0.0	GO:0030170	81	70.0922	48
ARC_ART_c23671	180	XP_007010980	0.9	-	62	33.113	35
ARC_ART_c23683	145	XP_002536306	0.0	-	62	42.3578	43
ARC_ART_c23687	115	AIX93516	0.0	GO:0006200	86	52.7582	37
ARC_ART_c23690	220	XP_008339803	0.0	-	52	42.3578	42
ARC_ART_c23708	163	XP_010326265	0.4	-	50	32.7278	55
ARC_ART_c23711	126	ABC98213	0.1	-	56	35.039	41
ARC_ART_c23735	137	XP_005643903	0.0	GO:0006950	66	54.299	42
ARC_ART_c23751	151	XP_009615792	0.0	GO:0055114	80	46.9802	36
ARC_ART_c23757	118	AFR62623	0.0	-	60	37.3502	38
ARC_ART_c23760	119	P53385	0.0	GO:0019557	82	64.6994	39
ARC_ART_c23771	119	XP_002538260	0.0	-	71	39.6614	39
ARC_ART_c23781	158	XP_005848152	0.0	GO:0005488	68	47.7506	45
ARC_ART_c23785	136	XP_002535112	0.0	GO:0006810	75	50.447	45
ARC_ART_c23802	112	XP_005844905	0.0	-	63	44.669	38
ARC_ART_c23804	105	KJB46002	0.0	-	64	38.891	34
ARC_ART_c23807	143	XP_002535067	0.1	-	60	34.6538	45

ARC_ART_c23827	182	XP_010442414	0.1	-	71	36.1946	39
ARC_ART_c23837	170	KJB71111	0.3	-	57	31.9574	49
ARC_ART_c23879	109	XP_010239602	0.0	GO:0016491	79	56.225	34
ARC_ART_c23892	112	XP_001770826	0.0	GO:0004252	86	65.0846	37
ARC_ART_c23913	132	KJB80746	0.1	-	62	35.4242	40
ARC_ART_c23923	120	AAF44094	0.1	-	61	33.8834	34
ARC_ART_c23929	210	ABO20851	0.0	-	62	42.743	43
ARC_ART_c23967	108	XP_010503456	0.6	-	51	32.7278	35
ARC_ART_c23992	111	XP_009350805	0.0	-	80	46.2098	36
ARC_ART_c24001	154	EMS48033	0.2	-	58	34.6538	39
ARC_ART_c24028	118	XP_003638791	0.4	-	52	31.187	40
ARC_ART_c24043	109	XP_002535693	0.7	-	67	32.3426	34
ARC_ART_c24058	130	XP_001767436	0.0	-	62	38.891	35
ARC_ART_c24063	126	XP_006852286	0.0	GO:0046872	80	51.9878	36
ARC_ART_c24069	164	XP_002537026	0.0	GO:0007165	61	54.299	49
ARC_ART_c24081	199	CDY14165	0.0	GO:0044444	65	53.9138	46
ARC_ART_c24084	119	XP_005844905	0.0	-	64	39.6614	39
ARC_ART_c24093	110	ADE75948	0.0	GO:0006526	80	55.0694	36
ARC_ART_c24104	203	NP_001105393	0.0	-	45	37.7354	64
ARC_ART_c24135	159	XP_004970392	1.0	-	48	32.7278	41
ARC_ART_c24142	201	KDO59655	0.0	-	48	37.7354	49
ARC_ART_c24148	104	XP_002450072	0.2	-	61	33.4982	36
ARC_ART_c24154	150	XP_003626499	0.2	-	50	33.113	46
ARC_ART_c24170	151	XP_003064509	0.0	GO:0044699	64	48.1358	48

ARC_ART_c24171	124	XP_010934676	1.0	-	55	32.3426	40
ARC_ART_c24195	193	ABK34503	0.0	GO:0005829	64	44.669	51
ARC_ART_c24211	196	XP_001786949	0.0	GO:0005739	81	58.5362	38
ARC_ART_c24231	191	XP_002962254	0.0	GO:0044763	64	38.5058	42
ARC_ART_c24232	175	KDD73200	0.0	GO:0016491	67	56.9954	55
ARC_ART_c24276	110	XP_007008841	0.0	-	66	38.891	36
ARC_ART_c24283	153	XP_011016758	0.0	-	81	72.4034	48
ARC_ART_c24296	138	XP_002966016	0.1	-	70	35.4242	37
ARC_ART_c24298	146	XP_003057632	0.0	GO:0004333	91	62.3882	34
ARC_ART_c24304	141	XP_003078364	0.0	GO:0042558	86	59.6918	37
ARC_ART_c24317	118	KIZ00962	0.7	-	59	32.3426	37
ARC_ART_c24327	104	XP_002540481	0.0	-	67	42.743	34
ARC_ART_c24380	169	XP_009350059	0.2	-	52	34.6538	40
ARC_ART_c24389	112	EAY93131	0.0	GO:0006200	82	56.6102	35
ARC_ART_c24416	236	P51598	0.1	-	64	34.2686	34
ARC_ART_c24445	131	XP_001787070	0.0	-	79	57.3806	43
ARC_ART_c24479	143	XP_002535483	0.1	-	56	35.4242	50
ARC_ART_c24483	180	XP_002535080	0.0	-	64	54.6842	53
ARC_ART_c24496	116	XP_005647813	0.0	GO:0051287	84	60.4622	38
ARC_ART_c24516	147	EMT17334	0.4	-	63	33.8834	41
ARC_ART_c24526	121	XP_002537480	0.0	-	71	39.6614	39
ARC_ART_c24553	151	EEC81324	0.0	GO:0044699	67	50.8322	49
ARC_ART_c24575	141	XP_006663494	0.0	GO:0016020	82	62.3882	39
ARC_ART_c24602	121	KDP37970	0.0	GO:0016740	66	46.2098	45
ARC_ART_c24610	128	EEE52318	0.0	GO:0022891	100	89.3521	42
ARC_ART_c24612	135	XP_002976226	0.1	-	51	34.6538	43

ARC_ART_c24614	115	EPS67857	0.1	-	56	33.113	37
ARC_ART_c24615	160	XP_009396118	0.0	GO:0009628	71	44.2838	39
ARC_ART_c24624	149	XP_005848116	0.9	-	65	32.7278	40
ARC_ART_c24636	137	XP_009343554	0.2	-	47	34.2686	46
ARC_ART_c24655	133	XP_002536757	0.0	-	75	56.9954	44
ARC_ART_c24686	189	XP_002539825	0.0	-	59	44.669	57
ARC_ART_c24702	109	XP_001751994	0.0	-	63	36.5798	36
ARC_ART_c24703	135	XP_002536736	0.0	GO:0006950	84	73.559	44
ARC_ART_c24719	118	EEE68528	0.0	GO:0016020	71	54.299	39
ARC_ART_c24723	164	EEE50470	0.0	-	71	40.4318	35
ARC_ART_c24724	142	XP_002534690	0.0	GO:0009987	64	43.5134	37
ARC_ART_c24730	177	XP_004969123	0.4	-	45	34.2686	51
ARC_ART_c24741	102	XP_002535323	0.3	-	62	33.113	35
ARC_ART_c24757	169	XP_006575465	0.0	GO:0003700	78	51.9878	55
ARC_ART_c24779	162	XP_001762172	0.0	GO:0005618	83	77.411	54
ARC_ART_c24783	150	XP_001422505	0.0	GO:0044763	67	47.3654	49
ARC_ART_c24784	156	XP_011035202	0.1	-	48	35.8094	47
ARC_ART_c24799	113	XP_006412929	0.0	GO:0003700	89	52.7582	37
ARC_ART_c24813	125	XP_001774821	0.0	-	63	42.743	41
ARC_ART_c24814	146	EXB44348	0.1	-	58	34.2686	43
ARC_ART_c24827	120	XP_002538654	0.0	-	69	45.0542	39
ARC_ART_c24831	163	XP_006665177	0.0	GO:0016638	59	43.8986	59
ARC_ART_c24843	146	CEF98966	0.1	-	75	35.4242	40
ARC_ART_c24860	129	XP_010314956	0.0	GO:0016301	67	44.2838	43
ARC_ART_c24871	189	XP_009351044	0.0	-	98	119.783	63
ARC_ART_c24873	117	XP_002539331	0.1	-	56	34.2686	37

ARC_ART_c24898	109	XP_009359775	0.0	-	74	42.743	35
ARC_ART_c24919	126	XP_001417046	0.0	GO:1901564	78	60.8474	42
ARC_ART_c24932	250	XP_005652131	0.3	-	60	34.6538	41
ARC_ART_c24943	106	XP_001761573	0.0	GO:0016757	85	59.6918	34
ARC_ART_c24950	110	CDP16523	0.3	-	58	33.4982	39
ARC_ART_c24951	169	KEH15214	0.0	-	54	35.8094	35
ARC_ART_c24952	120	XP_010250738	0.1	-	65	34.6538	40
ARC_ART_c24967	227	KIZ04322	0.0	GO:0003824	54	46.595	68
ARC_ART_c24969	108	XP_002535095	0.0	GO:0006099	91	71.633	35
ARC_ART_c24971	203	XP_002536908	0.0	-	60	35.8094	38
ARC_ART_c24980	217	XP_001416282	0.0	-	57	45.8246	70
ARC_ART_c24984	186	XP_001701329	0.0	-	70	37.3502	34
ARC_ART_c24992	180	XP_009350072	0.0	-	88	39.6614	43
ARC_ART_c24993	118	XP_002536517	0.0	GO:0003824	74	48.9062	39
ARC_ART_c25009	110	ERM97829	0.0	GO:0016679	76	42.3578	34
ARC_ART_c25015	193	CCO16256	0.0	GO:0055114	67	43.5134	37
ARC_ART_c25027	125	KDD76196	0.0	-	64	39.2762	37
ARC_ART_c25029	128	XP_002537183	0.0	-	68	36.965	41
ARC_ART_c25049	156	KDD76113	0.0	-	70	36.1946	34
ARC_ART_c25053	203	XP_010911537	0.0	-	73	70.4774	67
ARC_ART_c25055	163	XP_001696579	0.0	-	63	39.6614	47
ARC_ART_c25057	157	EEC83290	0.0	GO:0016021	96	101.679	52
ARC_ART_c25059	152	XP_009358444	0.0	GO:0046872	73	56.6102	34
ARC_ART_c25090	104	XP_002501695	0.0	GO:0003824	70	43.8986	34
ARC_ART_c25100	166	XP_002535671	0.0	GO:0016836	77	63.929	44
ARC_ART_c25113	147	XP_008237649	0.0	GO:0016020	75	55.0694	44
ARC_ART_c25124	185	YP_009106719	0.5	-	62	33.113	37
ARC_ART_c25127	121	XP_001775553	0.6	-	61	32.7278	34

ARC_ART_c25129	101	XP_002979495	0.8	-	68	32.3426	38
ARC_ART_c25141	119	XP_005647694	0.0	-	68	44.2838	41
ARC_ART_c25164	116	XP_001419597	0.1	-	60	34.6538	35
ARC_ART_c25167	254	ACG44134	0.8	-	51	33.4982	76
ARC_ART_c25173	274	XP_002539909	0.0	GO:0003824	60	70.8626	86
ARC_ART_c25190	173	XP_006360181	0.1	-	51	36.1946	49
ARC_ART_c25192	135	EEC80174	0.0	GO:0050660	85	66.2402	41
ARC_ART_c25201	191	XP_002317003	0.8	-	58	31.5722	39
ARC_ART_c25242	102	XP_002538551	0.0	GO:0004609	79	45.8246	34
ARC_ART_c25284	178	XP_002513108	0.2	-	48	33.113	52
ARC_ART_c25288	159	EDQ48081	0.5	-	60	33.113	53
ARC_ART_c25308	167	XP_002536027	0.0	GO:0005975	84	67.3958	46
ARC_ART_c25325	123	XP_002538571	0.0	-	67	41.5874	40
ARC_ART_c25338	164	KFK31608	0.5	-	69	33.4982	49
ARC_ART_c25343	119	XP_002504531	0.0	GO:0030529	75	43.1282	36
ARC_ART_c25348	134	XP_003060243	0.0	-	54	49.2914	57
ARC_ART_c25356	150	XP_002536715	0.7	-	57	32.7278	38
ARC_ART_c25360	136	XP_001420863	0.0	GO:0009791	77	61.2326	45
ARC_ART_c25363	120	XP_003056064	0.0	GO:0006527	69	47.7506	39
ARC_ART_c25378	184	XP_002535396	0.0	GO:0070548	77	71.2478	54
ARC_ART_c25402	106	XP_010927366	0.7	-	62	32.7278	35
ARC_ART_c25403	190	XP_002505390	0.0	-	54	44.669	71
ARC_ART_c25437	113	CEF97698	0.0	-	61	36.965	36
ARC_ART_c25438	118	CCO14832	0.1	-	61	36.1946	39
ARC_ART_c25455	244	XP_002539701	0.0	-	64	69.3218	68
ARC_ART_c25473	140	XP_006382402	0.8	-	62	32.3426	37

ARC_ART_c25477	136	XP_001753513	0.0	-	56	42.3578	46
ARC_ART_c25517	105	EMT09363	0.1	-	61	34.6538	34
ARC_ART_c25545	206	XP_006352818	0.0	GO:0016740	66	65.4698	65
ARC_ART_c25556	119	EXB51235	0.0	-	59	36.1946	42
ARC_ART_c25562	112	XP_009408484	0.0	GO:0009630	80	53.9138	35
ARC_ART_c25575	147	XP_005848125	0.0	GO:0008152	82	63.929	50
ARC_ART_c25596	119	XP_002539813	0.0	GO:0016301	71	52.373	38
ARC_ART_c25598	138	XP_002976440	0.7	-	53	32.7278	39
ARC_ART_c25605	112	XP_001774097	0.0	-	75	40.0466	36
ARC_ART_c25614	126	XP_006589020	0.1	-	64	33.113	37
ARC_ART_c25637	121	XP_002535042	0.0	GO:0016787	77	48.521	36
ARC_ART_c25647	128	KCW55079	0.9	-	60	30.0314	35
ARC_ART_c25672	129	XP_009351102	0.0	-	62	38.5058	40
ARC_ART_c25738	140	XP_005650663	0.0	-	59	36.5798	42
ARC_ART_c25774	217	KJB49896	0.0	-	50	39.6614	51
ARC_ART_c25797	157	XP_010910724	0.1	-	58	35.8094	50
ARC_ART_c25798	180	XP_001786556	0.0	GO:0055114	76	59.6918	43
ARC_ART_c25847	120	XP_002539813	0.0	GO:0009987	65	45.4394	38
ARC_ART_c25865	105	XP_002534778	0.0	-	61	38.891	34
ARC_ART_c25900	113	ACF78657	0.4	-	54	33.113	35
ARC_ART_c25905	127	XP_001756660	0.0	GO:0071704	64	43.5134	34
ARC_ART_c25940	169	XP_002535099	0.0	-	66	62.003	56
ARC_ART_c25953	129	XP_009351046	0.0	-	77	55.0694	40
ARC_ART_c25954	138	KJB83201	0.6	-	48	32.7278	39
ARC_ART_c25967	213	XP_003595332	0.6	-	61	31.9574	36
ARC_ART_c25973	107	EEC78702	0.0	GO:0015748	82	52.373	34

ARC_ART_c25988	138	XP_007221502	0.0	GO:0016226	83	57.7658	37
ARC_ART_c25999	117	XP_005643555	0.0	GO:0015969	79	48.521	34
ARC_ART_c26003	148	AJB84617	0.0	GO:0008661	82	59.3066	35
ARC_ART_c26009	142	AGL09528	0.3	-	52	33.4982	44
ARC_ART_c26019	225	AAT07580	0.1	-	53	36.5798	47
ARC_ART_c26024	181	EAZ10377	0.0	GO:0006810	100	119.398	57
ARC_ART_c26050	167	XP_002536031	0.0	-	60	40.817	46
ARC_ART_c26104	216	XP_002537439	0.0	GO:0006139	65	63.5438	67
ARC_ART_c26116	112	XP_005648785	0.1	-	62	35.039	35
ARC_ART_c26122	136	XP_005851714	0.0	-	62	44.669	43
ARC_ART_c26160	132	EEC75945	0.0	GO:0006200	97	83.5741	42
ARC_ART_c26162	108	XP_002537367	0.0	-	76	46.9802	34
ARC_ART_c26163	168	XP_002503674	0.0	GO:0016740	62	62.7734	61
ARC_ART_c26166	145	XP_002950762	0.0	GO:0004832	79	72.4034	48
ARC_ART_c26187	110	XP_002960856	0.0	-	68	39.2762	35
ARC_ART_c26190	182	XP_010064094	0.0	GO:0043231	61	51.6026	57
ARC_ART_c26194	149	CBI27940	0.8	-	60	32.7278	45
ARC_ART_c26197	139	ACU20916	0.1	-	71	33.8834	38
ARC_ART_c26204	141	XP_002535669	0.0	-	69	48.1358	42
ARC_ART_c26210	228	ABO20848	0.3	-	66	33.4982	36
ARC_ART_c26214	122	KJB35578	0.0	GO:0043168	60	48.1358	40
ARC_ART_c26216	183	XP_003063241	0.0	-	79	47.7506	39
ARC_ART_c26232	133	EEC70278	0.0	-	70	39.6614	34
ARC_ART_c26240	158	AAS55774	0.5	-	50	33.4982	40
ARC_ART_c26243	130	CCO19552	0.1	-	71	36.1946	35
ARC_ART_c26264	118	XP_009398358	0.4	-	56	33.113	37
ARC_ART_c26266	137	EYU34366	0.5	-	51	33.4982	37
ARC_ART_c26275	125	XP_005646334	0.0	-	65	37.7354	43

ARC_ART_c26289	196	XP_006859134	0.0	-	46	36.965	62
ARC_ART_c26331	112	XP_001787052	0.8	-	56	31.9574	37
ARC_ART_c26391	142	XP_010273360	0.6	-	57	32.7278	42
ARC_ART_c26409	210	XP_003524529	0.8	-	52	31.187	42
ARC_ART_c26433	177	XP_009350073	0.0	-	76	56.9954	55
ARC_ART_c26455	139	XP_001695163	0.0	GO:0016668	73	43.1282	41
ARC_ART_c26458	147	XP_002534945	0.0	-	70	47.3654	44
ARC_ART_c26465	144	XP_002538398	0.4	-	64	33.4982	42
ARC_ART_c26484	124	EHK62748	0.0	-	59	37.3502	37
ARC_ART_c26489	123	XP_005649581	0.0	GO:0016787	67	44.2838	43
ARC_ART_c26498	150	XP_002973927	0.0	GO:0016310	62	45.0542	48
ARC_ART_c26500	187	XP_005850540	0.0	-	51	37.3502	64
ARC_ART_c26506	173	KFM28383	0.0	GO:0005524	85	50.0618	35
ARC_ART_c26516	136	AFW74576	0.8	-	55	31.9574	45
ARC_ART_c26520	122	XP_004287847	0.7	-	55	32.7278	40
ARC_ART_c26568	114	XP_002536181	0.0	-	70	37.7354	37
ARC_ART_c26570	152	EDQ48133	0.0	GO:0098655	65	53.9138	47
ARC_ART_c26581	145	EMS46537	0.4	-	52	33.8834	44
ARC_ART_c26588	163	XP_002540039	0.0	-	56	35.4242	39
ARC_ART_c26612	205	AIU49686	0.7	-	46	33.4982	50
ARC_ART_c26623	138	XP_009392145	0.1	-	53	35.4242	43
ARC_ART_c26626	126	EYU46065	1.0	-	56	31.9574	39
ARC_ART_c26652	194	KDP24866	0.1	-	47	35.4242	44
ARC_ART_c26670	165	CEF98925	0.0	GO:0009570	69	51.9878	52
ARC_ART_c26690	122	EPS63668	1.0	-	52	31.9574	38
ARC_ART_c26705	106	XP_006481914	0.0	-	70	39.2762	34

ARC_ART_c26715	138	XP_002503478	0.0	GO:0019760	65	52.373	52
ARC_ART_c26729	103	XP_005650979	0.0	GO:0016746	85	52.373	34
ARC_ART_c26738	110	XP_011623923	0.0	-	61	36.965	34
ARC_ART_c26750	117	XP_004513153	0.0	GO:0006520	64	41.9726	34
ARC_ART_c26766	142	AAF16525	0.0	GO:0000166	91	78.1814	46
ARC_ART_c26772	145	XP_002540139	0.0	-	65	43.5134	46
ARC_ART_c26795	137	AAV44205	0.0	GO:0009507	100	92.8189	45
ARC_ART_c26814	149	EEC78884	0.0	GO:0000155	100	99.7525	49
ARC_ART_c26826	220	XP_006361381	0.1	-	49	36.965	61
ARC_ART_c26844	154	XP_006471556	0.8	-	55	32.7278	47
ARC_ART_c26849	183	XP_009350065	0.0	GO:0044249	92	64.6994	40
ARC_ART_c26860	137	EEE52320	0.0	GO:0055085	97	87.4261	45
ARC_ART_c26863	110	XP_004514993	0.0	-	64	39.6614	34
ARC_ART_c26877	106	XP_001763306	0.0	GO:0006732	80	50.0618	35
ARC_ART_c26882	142	XP_002538506	0.0	-	62	42.743	37
ARC_ART_c26893	192	XP_011620979	0.0	-	56	38.5058	57
ARC_ART_c26902	162	XP_002537269	0.0	-	68	56.225	54
ARC_ART_c26903	167	XP_002537086	0.0	-	74	46.595	35
ARC_ART_c26911	234	NP_178215	0.1	-	67	35.8094	34
ARC_ART_c26916	118	XP_002539420	0.0	GO:0004871	83	59.6918	37
ARC_ART_c26945	232	AAW57408	0.0	-	74	92.4337	75
ARC_ART_c26949	126	NP_001151329	0.0	-	70	36.1946	34
ARC_ART_c26959	160	EEC69860	0.0	GO:0035556	74	65.4698	51
ARC_ART_c26973	143	XP_002535682	0.0	GO:0019134	87	62.3882	41

ARC_ART_c26981	127	ABP35525	0.0	-	68	38.891	35
ARC_ART_c26982	168	EEC70926	0.0	GO:0007010	96	107.071	55
ARC_ART_c26997	154	XP_002538508	0.0	-	56	40.817	46
ARC_ART_c27002	179	XP_009383471	0.7	-	54	33.4982	51
ARC_ART_c27012	119	XP_002535367	0.2	-	59	34.2686	37
ARC_ART_c27039	144	XP_001769676	0.1	-	57	36.1946	40
ARC_ART_c27047	204	XP_005650698	0.5	-	60	33.4982	45
ARC_ART_c27054	154	XP_002539292	0.0	-	75	53.1434	40
ARC_ART_c27061	144	XP_002536084	0.0	-	77	43.1282	36
ARC_ART_c27081	211	XP_002538092	0.0	GO:0016779	74	40.4318	39
ARC_ART_c27083	126	XP_004147344	0.0	GO:0009536	74	51.6026	39
ARC_ART_c27088	108	XP_002534926	0.0	-	88	63.5438	35
ARC_ART_c27111	132	XP_001758664	0.0	GO:0000038	82	51.6026	34
ARC_ART_c27112	109	XP_002539229	0.0	-	66	46.595	36
ARC_ART_c27115	119	XP_011621052	0.0	GO:0006259	71	52.7582	38
ARC_ART_c27136	114	XP_002536201	0.0	-	71	43.8986	38
ARC_ART_c27141	145	XP_008806274	0.9	-	56	32.3426	46
ARC_ART_c27154	158	XP_003566851	0.0	GO:0016798	59	51.2174	49
ARC_ART_c27159	143	XP_002534946	0.0	GO:0044699	78	57.3806	42
ARC_ART_c27164	183	EXC02512	0.2	-	46	33.8834	47
ARC_ART_c27165	135	NP_001235871	0.1	-	58	35.039	36
ARC_ART_c27173	139	XP_006293699	0.2	-	60	34.2686	35
ARC_ART_c27189	101	XP_010322044	0.7	-	56	32.7278	37
ARC_ART_c27190	134	XP_002538560	0.0	-	65	44.669	44
ARC_ART_c27205	206	KEH15180	0.0	GO:0016020	83	86.2705	66

ARC_ART_c27208	153	ABB55295	0.1	-	55	36.1946	47
ARC_ART_c27213	128	EAZ43720	0.0	GO:0006810	94	65.4698	36
ARC_ART_c27224	188	XP_009396444	0.0	GO:0044763	63	43.5134	55
ARC_ART_c27225	160	XP_006400501	0.0	-	64	43.1282	45
ARC_ART_c27237	157	XP_010439548	0.0	GO:0055114	75	59.6918	49
ARC_ART_c27241	107	CCO18640	0.1	-	61	34.6538	36
ARC_ART_c27247	120	EEE52319	0.0	GO:0006378	86	56.9954	37
ARC_ART_c27258	104	XP_002505421	0.0	-	67	38.891	34
ARC_ART_c27266	218	XP_002536459	0.0	GO:0016020	85	65.855	40
ARC_ART_c27279	124	XP_009786901	0.3	-	62	33.8834	37
ARC_ART_c27293	138	KJB19594	0.7	-	57	32.3426	40
ARC_ART_c27302	131	XP_004253340	0.0	GO:0003824	80	58.151	40
ARC_ART_c27306	136	XP_002535387	0.8	-	60	32.7278	38
ARC_ART_c27309	140	KFM25768	0.0	-	62	38.1206	40
ARC_ART_c27319	108	YP_009033827	0.0	GO:0003723	72	44.2838	36
ARC_ART_c27325	150	KDD76762	0.0	GO:0044260	68	52.7582	47
ARC_ART_c27337	113	KFM28773	0.1	-	57	35.8094	35
ARC_ART_c27354	116	EAZ01443	0.0	GO:0046872	84	49.6766	38
ARC_ART_c27370	256	XP_002960003	0.0	-	50	41.9726	97
ARC_ART_c27383	166	BAL45642	0.0	GO:0005737	74	51.2174	47
ARC_ART_c27407	218	KFK35285	0.7	-	48	33.8834	64
ARC_ART_c27409	122	XP_002540612	0.0	-	64	37.7354	34
ARC_ART_c27414	185	XP_001416550	0.1	-	49	36.5798	55
ARC_ART_c27417	153	XP_002985916	0.4	-	61	32.7278	36
ARC_ART_c27435	184	XP_002960909	0.0	GO:0016874	74	44.2838	43
ARC_ART_c27437	114	BAD25279	0.0	GO:0009409	72	46.2098	36

ARC_ART_c27454	142	XP_001422244	0.0	GO:0019752	76	67.781	46
ARC_ART_c27455	182	XP_004253340	0.0	GO:0003824	75	60.8474	41
ARC_ART_c27464	221	EMT16003	0.7	-	56	33.4982	39
ARC_ART_c27474	129	XP_001779855	0.0	GO:0097159	65	40.817	35
ARC_ART_c27482	121	EDQ49166	0.0	-	71	40.817	39
ARC_ART_c27495	112	XP_003083741	0.0	GO:0006526	94	72.4034	37
ARC_ART_c27504	185	XP_001758646	0.0	-	66	43.8986	48
ARC_ART_c27516	151	XP_005649330	0.5	-	60	33.8834	43
ARC_ART_c27520	140	DAA60665	0.0	-	90	71.633	40
ARC_ART_c27545	131	EPS66370	0.7	-	59	32.7278	37
ARC_ART_c27555	142	XP_002969283	0.0	-	68	40.4318	38
ARC_ART_c27576	113	XP_001786556	0.0	GO:0046914	75	49.2914	37
ARC_ART_c27580	120	XP_003062684	0.0	GO:0005840	76	43.8986	34
ARC_ART_c27614	135	EAZ14329	0.0	GO:0051536	73	49.2914	34
ARC_ART_c27638	102	KEH15495	0.0	GO:0050660	91	62.003	34
ARC_ART_c27639	140	XP_006387410	0.7	-	55	31.5722	40
ARC_ART_c27656	122	XP_001786560	0.0	GO:0044763	71	44.2838	39
ARC_ART_c27658	137	ERN15094	0.0	-	58	34.2686	39
ARC_ART_c27659	185	XP_001786686	0.0	GO:0016020	89	82.8037	57
ARC_ART_c27662	104	XP_002535693	0.0	GO:0000155	79	48.521	34
ARC_ART_c27689	116	XP_011016759	0.6	-	65	32.7278	35
ARC_ART_c27700	145	KEH26635	0.0	GO:0003676	76	45.4394	38
ARC_ART_c27728	122	EEE68528	0.0	GO:0010039	100	80.4925	40
ARC_ART_c27735	263	XP_010916171	0.2	-	58	35.4242	55
ARC_ART_c27773	170	XP_001767418	0.0	GO:0043168	71	70.4774	53
ARC_ART_c27776	125	XP_005648771	0.6	-	51	33.113	37
ARC_ART_c27808	193	XP_002538477	0.0	-	70	39.6614	37

ARC_ART_c27811	142	KDD72463	0.0	GO:0051234	63	46.2098	46
ARC_ART_c27816	150	AIY30182	0.0	GO:0044464	67	54.299	49
ARC_ART_c27831	159	CCO17444	0.0	GO:0016070	66	42.3578	36
ARC_ART_c27835	189	KEH28804	0.1	-	65	35.4242	35
ARC_ART_c27848	107	AFR32946	0.0	-	71	43.8986	35
ARC_ART_c27850	128	EXC10888	0.0	-	61	37.7354	36
ARC_ART_c27868	115	XP_002539002	0.2	-	60	33.8834	35
ARC_ART_c27905	135	XP_001786775	0.0	GO:0005524	93	71.2478	43
ARC_ART_c27919	222	KIZ02693	0.0	-	73	56.6102	46
ARC_ART_c27935	217	KIY94278	0.9	-	56	32.7278	37
ARC_ART_c27965	119	XP_002536609	0.1	-	66	35.8094	36
ARC_ART_c27966	124	XP_002535650	0.0	-	75	51.9878	40
ARC_ART_c27990	140	XP_003060652	0.0	GO:0044765	73	56.225	45
ARC_ART_c27991	112	XP_002535268	0.0	-	72	41.2022	36
ARC_ART_c27995	127	XP_006596688	0.5	-	61	33.113	42
ARC_ART_c27999	124	XP_002539401	0.0	-	69	45.0542	39
ARC_ART_c28023	127	XP_001786429	0.2	-	66	34.2686	39
ARC_ART_c28044	228	BAD34016	0.5	-	50	34.2686	52
ARC_ART_c28061	139	XP_011628751	0.0	GO:0008152	77	56.9954	44
ARC_ART_c28079	160	XP_002540486	0.0	-	70	59.6918	51
ARC_ART_c28085	139	XP_003579106	0.2	-	57	34.6538	45
ARC_ART_c28089	105	XP_002540486	0.2	-	61	32.7278	34
ARC_ART_c28094	149	XP_001417857	0.0	GO:0016788	85	51.6026	35
ARC_ART_c28136	315	XP_001695478	0.0	-	55	64.6994	87
ARC_ART_c28148	125	XP_002536712	0.7	-	54	32.3426	37
ARC_ART_c28153	104	XP_007154101	0.0	GO:0009555	82	56.225	34
ARC_ART_c28163	162	XP_009350056	0.0	-	79	52.373	39

ARC_ART_c28166	118	EAY85922	0.0	GO:0016021	94	69.3218	39
ARC_ART_c28195	120	CDO97595	0.1	-	64	35.039	34
ARC_ART_c28227	116	XP_002537563	0.0	-	68	36.5798	35
ARC_ART_c28228	113	XP_002535792	0.0	GO:0016829	74	50.0618	35
ARC_ART_c28240	134	XP_001763943	0.0	GO:0006952	69	50.8322	43
ARC_ART_c28244	107	ADE77682	0.0	-	65	36.965	35
ARC_ART_c28259	116	XP_002534939	0.0	GO:0006810	83	57.3806	37
ARC_ART_c28268	102	XP_003578660	0.0	GO:0017111	76	41.2022	34
ARC_ART_c28278	120	XP_002534800	0.0	-	69	40.817	36
ARC_ART_c28295	116	XP_002984729	0.0	GO:0004832	81	55.8398	38
ARC_ART_c28322	109	EEC77966	0.0	-	65	44.2838	35
ARC_ART_c28334	138	KJB55249	0.3	-	64	33.8834	34
ARC_ART_c28340	146	EEC69860	0.0	GO:0000155	100	99.3673	48
ARC_ART_c28397	125	XP_002537747	1.0	-	60	31.9574	35
ARC_ART_c28422	172	ADK60808	0.1	-	44	33.113	43
ARC_ART_c28428	130	EEE52318	0.0	GO:0022891	100	83.9593	42
ARC_ART_c28436	118	XP_010034261	0.3	-	55	33.4982	34
ARC_ART_c28444	195	EEC76877	0.0	GO:0046961	91	78.9518	47
ARC_ART_c28451	110	XP_003057564	0.0	GO:0009396	86	49.2914	36
ARC_ART_c28487	130	XP_002537864	0.0	-	55	36.965	38
ARC_ART_c28510	171	KDP25215	0.7	-	48	33.113	60
ARC_ART_c28533	121	CAC80381	0.0	GO:0051287	83	58.5362	37
ARC_ART_c28535	116	XP_002537525	1.0	-	62	31.9574	37
ARC_ART_c28553	103	XP_001697381	0.5	-	76	33.113	34
ARC_ART_c28560	107	CCO66287	0.0	GO:0042558	76	45.8246	34
ARC_ART_c28569	112	AFB33036	0.0	GO:0034660	81	54.6842	37
ARC_ART_c28583	110	AIA92249	0.0	GO:0044763	70	41.2022	34
ARC_ART_c28594	135	XP_002536202	0.1	-	60	33.8834	35
ARC_ART_c28597	108	XP_006373052	0.0	-	65	37.7354	35
ARC_ART_c28608	128	XP_002538887	0.0	-	70	45.8246	41

ARC_ART_c28643	253	YP_001315124	0.0	GO:0046872	83	97.4413	55
ARC_ART_c28646	107	XP_005645707	0.0	GO:0008152	77	50.447	35
ARC_ART_c28658	134	XP_003635953	0.8	-	51	32.3426	39
ARC_ART_c28669	122	KIZ01602	0.0	GO:0016208	75	58.5362	40
ARC_ART_c28683	109	EEC79194	0.0	GO:0008152	65	42.743	35
ARC_ART_c28685	190	XP_003578455	0.5	-	62	33.8834	43
ARC_ART_c28693	167	XP_002509137	0.0	GO:0046872	78	80.1073	52
ARC_ART_c28697	157	EMT06397	0.7	-	61	31.5722	34
ARC_ART_c28716	127	XP_002948411	0.7	-	58	32.7278	39
ARC_ART_c28717	140	XP_010468223	0.2	-	63	33.8834	38
ARC_ART_c28721	147	XP_009350067	0.0	GO:0005840	78	62.003	46
ARC_ART_c28736	140	XP_006446769	0.0	-	62	36.965	35
ARC_ART_c28740	139	XP_002535893	0.0	GO:0006810	80	50.447	35
ARC_ART_c28747	143	XP_002982511	0.0	GO:0043231	74	63.929	43
ARC_ART_c28768	114	XP_001786930	0.0	-	70	40.0466	37
ARC_ART_c28770	209	KJB39443	0.7	-	47	33.113	46
ARC_ART_c28785	233	XP_001696864	0.0	-	52	38.5058	48
ARC_ART_c28786	112	XP_006352743	0.1	-	55	35.4242	34
ARC_ART_c28789	269	XP_002538511	0.0	GO:0006355	90	87.0409	52
ARC_ART_c28803	146	XP_002945774	0.0	GO:0008483	72	46.9802	37
ARC_ART_c28804	174	XP_006307191	0.8	-	57	33.113	42
ARC_ART_c28811	137	XP_003074986	0.0	-	59	35.4242	44
ARC_ART_c28819	159	XP_005843309	1.0	-	65	32.7278	40
ARC_ART_c28846	111	XP_002959939	1.0	-	57	31.9574	35
ARC_ART_c28883	136	XP_002505901	0.0	-	60	39.6614	43
ARC_ART_c28909	136	XP_009350059	0.0	-	82	45.4394	34

ARC_ART_c28964	161	XP_002447232	0.8	-	55	32.7278	38
ARC_ART_c28967	127	BAJ94909	0.0	GO:0043231	87	67.781	41
ARC_ART_c28985	108	XP_002536731	0.0	-	73	35.8094	34
ARC_ART_c28988	173	XP_002535483	0.0	GO:0006266	82	93.2041	57
ARC_ART_c29005	163	XP_009392064	0.5	-	64	33.4982	34
ARC_ART_c29023	154	XP_010911538	0.0	-	90	89.7373	50
ARC_ART_c29025	180	XP_010242706	0.0	GO:0008152	65	47.7506	49
ARC_ART_c29026	144	XP_002536181	0.0	GO:0035556	68	60.4622	50
ARC_ART_c29062	129	XP_002539401	0.1	-	63	34.2686	41
ARC_ART_c29063	107	XP_002536228	0.1	-	65	34.6538	35
ARC_ART_c29083	119	XP_009350805	0.0	GO:0006399	86	65.0846	36
ARC_ART_c29085	150	XP_001697990	0.0	-	56	38.1206	51
ARC_ART_c29097	134	XP_002506222	0.0	GO:0046961	97	83.1889	44
ARC_ART_c29116	140	ABK34503	0.0	GO:0030170	88	67.3958	35
ARC_ART_c29118	134	AAF16526	0.0	GO:0044238	80	60.4622	40
ARC_ART_c29120	123	CDO46012	0.0	GO:0009941	70	52.7582	41
ARC_ART_c29121	173	XP_004503868	0.2	-	50	35.039	48
ARC_ART_c29154	197	XP_002535010	0.4	-	65	34.2686	41
ARC_ART_c29160	149	XP_002536048	0.0	GO:0004386	80	63.929	36
ARC_ART_c29174	152	KDO40386	0.8	-	54	32.7278	35
ARC_ART_c29179	160	XP_010237663	0.0	-	54	39.2762	48
ARC_ART_c29181	117	KFM28906	0.0	GO:0016874	77	55.0694	36
ARC_ART_c29188	105	NP_001041900	0.2	-	61	33.8834	34

ARC_ART_c29212	206	XP_006478581	0.1	-	56	35.8094	48
ARC_ART_c29241	130	EEC69551	0.0	GO:0044710	65	46.9802	41
ARC_ART_c29243	190	XP_001778066	0.1	-	50	35.8094	61
ARC_ART_c29245	163	KEH15984	0.0	GO:0097264	79	67.3958	48
ARC_ART_c29274	129	XP_010243740	0.6	-	61	33.113	34
ARC_ART_c29277	122	XP_002540026	0.0	GO:0004872	95	62.003	40
ARC_ART_c29303	103	CEG00946	0.1	-	64	35.039	34
ARC_ART_c29307	208	EAY93131	0.0	GO:0006812	80	62.7734	47
ARC_ART_c29334	117	XP_011016126	0.0	-	88	52.373	35
ARC_ART_c29335	139	XP_001755612	0.8	-	46	31.5722	49
ARC_ART_c29354	173	KEH30076	0.8	-	55	32.7278	34
ARC_ART_c29357	148	XP_002535868	0.9	-	51	32.3426	43
ARC_ART_c29360	119	ADF43173	0.0	-	69	39.2762	39
ARC_ART_c29362	114	XP_001786925	0.0	GO:0016798	75	46.9802	37
ARC_ART_c29377	118	XP_001780752	0.5	-	57	32.7278	35
ARC_ART_c29395	119	XP_011457537	0.5	-	55	33.113	36
ARC_ART_c29396	139	KEH15665	0.1	-	70	35.4242	40
ARC_ART_c29398	115	XP_002538317	0.0	GO:0008152	77	52.373	35
ARC_ART_c29400	138	XP_003563557	0.0	-	64	36.965	39
ARC_ART_c29428	160	ERN00894	0.0	-	57	34.6538	38
ARC_ART_c29451	132	AFW84361	0.0	GO:0016310	76	53.9138	42
ARC_ART_c29459	160	XP_002537785	0.0	-	61	37.7354	39
ARC_ART_c29507	160	XP_009780359	0.8	-	54	32.3426	44
ARC_ART_c29572	124	KDD74747	0.0	GO:0045252	89	62.003	38
ARC_ART_c29590	113	EEC72535	0.0	GO:0003840	81	56.225	37

ARC_ART_c29591	113	XP_002960029	0.0	GO:0046912	83	62.3882	37
ARC_ART_c29608	149	EYU19809	0.7	-	56	33.113	46
ARC_ART_c29628	164	XP_011016126	0.0	GO:0005525	94	77.411	38
ARC_ART_c29641	103	XP_002539176	0.0	-	85	58.151	34
ARC_ART_c29649	143	XP_002537823	0.0	-	65	48.9062	47
ARC_ART_c29651	147	AET49986	0.0	GO:0009536	75	48.9062	45
ARC_ART_c29701	138	ABH10989	0.0	GO:0019253	86	56.9954	43
ARC_ART_c29751	126	XP_001415394	0.0	GO:0008152	76	65.4698	42
ARC_ART_c29760	114	BAJ98915	0.0	GO:0044249	54	42.3578	50
ARC_ART_c29778	159	KIY99059	0.0	GO:0003677	85	55.4546	40
ARC_ART_c29808	132	XP_002958306	0.0	-	70	47.3654	37
ARC_ART_c29867	133	KEH15665	0.0	-	73	39.2762	42
ARC_ART_c29913	108	XP_001416314	0.0	GO:0006412	72	42.743	36
ARC_ART_c29918	110	XP_005844355	0.0	GO:1901564	73	45.4394	34
ARC_ART_c29936	116	NP_001049933	0.8	-	50	32.3426	38
ARC_ART_c29960	106	KIY95681	0.0	GO:0046872	91	55.4546	34
ARC_ART_c29982	131	XP_007148588	0.0	-	55	39.2762	38
ARC_ART_c30003	113	XP_001698575	0.0	GO:0004601	71	46.9802	35
ARC_ART_c30015	123	XP_006488109	0.0	GO:0003725	81	48.9062	37
ARC_ART_c30028	155	CEO90978	0.1	-	56	34.6538	44
ARC_ART_c30030	110	EAY84569	0.0	GO:0050660	91	65.0846	35
ARC_ART_c30066	175	XP_001771575	0.2	-	48	30.4166	39
ARC_ART_c30160	106	XP_002540107	0.0	-	76	43.1282	34

ARC_ART_c30182	111	CCO14651	0.0	GO:0004819	80	57.3806	35
ARC_ART_c30188	130	AEP14527	0.0	GO:0008152	65	46.595	46
ARC_ART_c30203	221	XP_002872619	0.9	-	60	33.4982	35
ARC_ART_c30210	122	XP_003058365	0.0	-	67	38.891	40
ARC_ART_c30230	138	XP_004253348	0.0	-	56	43.1282	44
ARC_ART_c30239	159	XP_001767852	0.1	-	52	35.8094	57
ARC_ART_c30246	115	XP_011073955	0.0	GO:0044763	67	42.3578	40
ARC_ART_c30250	172	XP_009350817	0.0	-	70	47.7506	55
ARC_ART_c30252	186	XP_002537869	0.0	-	54	38.891	44
ARC_ART_c30287	119	AFK46595	0.0	GO:0016740	71	50.447	39
ARC_ART_c30292	184	XP_008463365	0.4	-	54	33.4982	42
ARC_ART_c30312	131	XP_011016746	0.0	GO:0050660	78	51.6026	41
ARC_ART_c30315	179	XP_006843160	0.9	-	51	32.7278	52
ARC_ART_c30354	149	XP_007034236	0.2	-	60	34.6538	48
ARC_ART_c30355	129	EMS68724	0.0	-	72	41.2022	43
ARC_ART_c30370	116	KFM28648	0.0	GO:0004553	73	45.8246	38
ARC_ART_c30388	121	KEH38046	0.9	-	58	30.8018	36
ARC_ART_c30391	102	CAK22274	0.0	-	76	36.5798	34
ARC_ART_c30410	115	BAC85103	0.3	-	52	31.187	34
ARC_ART_c30450	226	ABH09321	0.0	-	62	51.2174	51
ARC_ART_c30461	165	CCO19745	0.0	GO:0006099	97	82.4185	40
ARC_ART_c30486	119	XP_008678843	0.0	GO:0005874	78	54.299	42
ARC_ART_c30499	105	XP_002505657	0.0	-	65	37.3502	35
ARC_ART_c30533	136	DAA51641	0.2	-	55	34.2686	34
ARC_ART_c30545	123	XP_001700040	0.0	-	64	38.1206	34
ARC_ART_c30549	207	EEE67870	0.0	GO:0009443	97	89.7373	44
ARC_ART_c30584	108	XP_005850491	0.0	GO:0046872	69	42.3578	36

ARC_ART_c30602	183	BAK08232	0.4	-	50	33.8834	46
ARC_ART_c30611	137	XP_010911304	0.0	GO:0016021	77	54.6842	44
ARC_ART_c30616	148	CAN74342	0.0	GO:0003676	77	61.2326	48
ARC_ART_c30639	112	XP_002539639	0.0	GO:0008152	91	67.3958	37
ARC_ART_c30644	154	XP_002537036	0.1	-	52	36.1946	51
ARC_ART_c30655	102	KFM26657	0.0	GO:0016747	88	59.3066	34
ARC_ART_c30663	136	XP_006645957	0.3	-	52	33.8834	42
ARC_ART_c30665	128	KDD72559	0.0	-	69	40.4318	39
ARC_ART_c30671	118	EEE62790	0.0	-	60	40.0466	35
ARC_ART_c30689	172	KEH16995	0.0	-	61	49.6766	57
ARC_ART_c30747	143	EYU31513	0.0	GO:0004013	82	57.7658	40
ARC_ART_c30795	109	EYU20359	0.0	GO:0006546	94	68.9366	36
ARC_ART_c30833	103	XP_009623156	0.0	GO:0044238	73	42.3578	34
ARC_ART_c30843	189	XP_001774199	0.1	-	57	35.4242	35
ARC_ART_c30846	184	XP_009418967	0.0	-	60	41.2022	55
ARC_ART_c30851	119	EEE68527	0.0	GO:0009982	94	72.0182	39
ARC_ART_c30856	133	KJB39913	0.8	-	64	32.3426	39
ARC_ART_c30870	105	XP_002538258	0.0	-	61	38.1206	34
ARC_ART_c30898	166	KDD75843	0.0	-	61	37.7354	36
ARC_ART_c30911	143	EEE52319	0.0	GO:0006378	93	69.3218	43
ARC_ART_c30950	143	EMT00267	0.0	GO:0005524	91	75.0998	47
ARC_ART_c30970	129	XP_002300010	0.0	GO:0004028	78	66.6254	41

ARC_ART_c30975	125	XP_010911304	0.0	GO:0006935	87	63.5438	41
ARC_ART_c30981	298	ACU18314	0.0	GO:0008483	62	56.225	56
ARC_ART_c30984	106	XP_005850918	0.0	-	58	39.6614	34
ARC_ART_c30988	196	XP_002539241	0.0	-	60	50.8322	58
ARC_ART_c30999	160	CDY51119	0.9	-	59	30.8018	42
ARC_ART_c31003	119	EDQ48693	0.4	-	58	33.4982	34
ARC_ART_c31006	182	XP_003081165	0.0	GO:0044763	67	54.299	62
ARC_ART_c31021	111	XP_002534565	0.3	-	62	33.4982	37
ARC_ART_c31124	133	XP_002536391	0.0	-	68	45.4394	41
ARC_ART_c31132	160	XP_001701551	0.0	-	71	47.3654	42
ARC_ART_c31138	107	AEZ35185	0.0	GO:0008661	94	66.6254	35
ARC_ART_c31153	119	XP_002536721	0.0	GO:0015109	100	72.4034	35
ARC_ART_c31180	110	XP_002537677	0.0	-	74	37.7354	35
ARC_ART_c31182	205	XP_003572614	0.0	GO:0044260	64	63.1586	64
ARC_ART_c31243	131	XP_002538617	0.7	-	61	32.3426	36
ARC_ART_c31253	214	EAZ38143	0.0	-	52	41.2022	61
ARC_ART_c31255	159	NP_001148975	0.0	GO:0042744	72	54.299	51
ARC_ART_c31263	226	XP_001762691	0.0	GO:0006097	86	68.1662	44
ARC_ART_c31268	142	XP_011016741	0.0	GO:0016798	100	95.5153	46
ARC_ART_c31290	191	YP_009105628	0.0	-	56	41.9726	51
ARC_ART_c31308	186	XP_001782628	0.0	GO:0044249	66	64.6994	62
ARC_ART_c31337	136	XP_001693679	0.0	GO:0004018	92	75.0998	42
ARC_ART_c31351	106	NP_191969	0.0	GO:0009396	80	50.8322	35
ARC_ART_c31357	122	BAD95114	0.0	GO:0005525	94	77.0258	38
ARC_ART_c31362	134	XP_006577887	0.0	-	66	38.1206	39
ARC_ART_c31369	153	XP_002535793	0.0	GO:0015031	80	71.633	47
ARC_ART_c31398	125	XP_006307046	0.0	GO:0003883	73	50.0618	41
ARC_ART_c31411	174	XP_002535934	0.0	-	57	42.3578	52
ARC_ART_c31484	108	AFW84119	1.0	-	54	32.3426	35

ARC_ART_c31520	107	XP_002536096	0.5	-	61	32.7278	34
ARC_ART_c31531	225	XP_002539885	0.0	GO:0009646	64	58.5362	68
ARC_ART_c31532	107	XP_002535646	0.0	-	71	41.9726	35
ARC_ART_c31538	141	XP_002534961	0.0	-	65	37.3502	44
ARC_ART_c31540	159	XP_002538205	0.0	GO:0071704	76	78.5666	52
ARC_ART_c31549	198	XP_005847305	0.0	GO:0008152	71	65.0846	52
ARC_ART_c31560	118	XP_002538359	0.7	-	60	32.3426	40
ARC_ART_c31580	174	EEC66865	0.0	GO:0009451	77	86.2705	58
ARC_ART_c31587	110	XP_002536340	0.5	-	73	31.5722	34
ARC_ART_c31600	128	XP_002958508	0.2	-	65	34.2686	40
ARC_ART_c31616	117	ACJ24144	0.0	GO:1901363	75	48.1358	36
ARC_ART_c31618	132	XP_002539055	0.5	-	58	32.7278	41
ARC_ART_c31633	118	XP_002536858	0.0	-	71	43.8986	39
ARC_ART_c31653	132	EEE64037	0.0	-	77	45.8246	36
ARC_ART_c31677	107	EEC69860	0.1	-	67	35.4242	34
ARC_ART_c31703	108	XP_011016604	0.0	-	85	55.4546	35
ARC_ART_c31708	125	XP_002535964	0.2	-	60	34.6538	35
ARC_ART_c31716	182	XP_002504270	0.0	GO:0009941	86	89.3521	60
ARC_ART_c31723	125	XP_011626026	0.3	-	52	33.8834	40
ARC_ART_c31737	107	XP_001701845	0.0	GO:0005525	94	62.3882	34
ARC_ART_c31740	148	XP_002536915	0.0	-	71	39.6614	39
ARC_ART_c31744	106	KIY99386	0.1	-	60	34.6538	35
ARC_ART_c31772	140	XP_001756683	0.2	-	46	33.113	43
ARC_ART_c31779	261	AES94792	0.7	-	56	33.8834	37
ARC_ART_c31792	146	XP_009352630	0.1	-	66	35.4242	36
ARC_ART_c31800	147	XP_002536145	0.0	GO:0003824	79	73.9442	48
ARC_ART_c31810	144	XP_002540107	0.2	-	50	33.8834	46
ARC_ART_c31841	265	XP_003057261	0.8	-	50	33.8834	63
ARC_ART_c31865	105	KDD71533	0.1	-	64	32.7278	34

ARC_ART_c31866	185	XP_004238764	0.4	-	55	33.4982	54
ARC_ART_c31935	147	XP_002538741	0.0	-	89	73.559	47
ARC_ART_c31962	243	DAA43248	0.0	GO:0005488	69	70.0922	59
ARC_ART_c31971	113	CCO14873	0.0	GO:0009536	75	43.1282	36
ARC_ART_c31975	135	ERN17573	0.0	-	55	34.2686	34
ARC_ART_c31979	106	XP_002537036	0.0	GO:0006810	88	58.9214	35
ARC_ART_c31983	141	XP_009589066	0.5	-	63	32.7278	36
ARC_ART_c31997	133	CDY38742	0.0	-	69	40.0466	42
ARC_ART_c32031	157	EHK62748	0.0	-	60	38.891	46
ARC_ART_c32032	265	CCO19047	0.2	-	44	35.039	72
ARC_ART_c32071	122	XP_002954421	0.0	-	72	36.965	40
ARC_ART_c32089	182	XP_010065933	0.1	-	53	33.8834	41
ARC_ART_c32097	148	ABK23023	0.7	-	70	32.3426	34
ARC_ART_c32102	140	XP_002538359	0.0	-	71	47.7506	45
ARC_ART_c32136	154	XP_002538265	0.0	GO:0008152	82	70.4774	51
ARC_ART_c32156	125	XP_002535933	0.0	-	58	40.4318	41
ARC_ART_c32179	133	XP_011016511	0.0	GO:0003735	100	81.6481	43
ARC_ART_c32180	121	EEC68548	0.0	GO:0002161	87	64.6994	40
ARC_ART_c32239	119	XP_011016741	0.0	-	97	76.2554	39
ARC_ART_c32257	173	EAZ01273	0.0	GO:0016020	70	55.4546	55
ARC_ART_c32268	123	XP_002539699	0.0	GO:0003700	97	80.8777	40
ARC_ART_c32275	114	XP_001754886	0.1	-	60	34.6538	35
ARC_ART_c32314	121	EEC68569	0.4	-	63	33.113	36
ARC_ART_c32321	113	XP_002536840	0.0	-	67	48.521	37
ARC_ART_c32347	115	XP_002537031	0.0	GO:0016857	78	56.225	38

ARC_ART_c32364	115	KEH15386	0.0	-	71	40.817	35
ARC_ART_c32375	160	XP_002505597	0.0	GO:0008446	76	55.4546	43
ARC_ART_c32383	151	XP_002946381	0.0	-	54	38.5058	50
ARC_ART_c32408	138	XP_002536966	0.0	-	62	38.5058	35
ARC_ART_c32409	169	CAN83097	1.0	-	50	32.7278	50
ARC_ART_c32411	102	XP_001760988	0.3	-	67	33.4982	34
ARC_ART_c32417	133	XP_010460062	0.2	-	45	34.6538	40
ARC_ART_c32419	113	XP_009759241	0.0	-	69	38.891	36
ARC_ART_c32435	142	XP_002948892	0.0	-	67	41.5874	34
ARC_ART_c32436	131	XP_004306011	0.0	GO:0044272	71	47.3654	38
ARC_ART_c32439	127	XP_002536683	0.0	-	69	43.8986	42
ARC_ART_c32442	103	XP_003057193	0.0	-	67	38.1206	34
ARC_ART_c32460	157	XP_002538291	0.0	-	90	74.7146	42
ARC_ART_c32478	178	KFM24803	0.0	GO:0090305	72	63.5438	55
ARC_ART_c32481	191	AAT48249	0.0	-	60	42.3578	38
ARC_ART_c32491	138	ABK23274	0.0	GO:0005829	84	56.225	38
ARC_ART_c32496	122	CEG00732	0.5	-	68	32.7278	35
ARC_ART_c32505	121	AFI48918	0.0	-	60	37.7354	40
ARC_ART_c32519	146	XP_002535791	0.0	-	75	40.0466	36
ARC_ART_c32536	148	XP_002946078	0.0	GO:0016747	73	61.6178	49
ARC_ART_c32562	148	AAA77045	0.0	-	57	39.6614	47
ARC_ART_c32573	151	ADQ38094	0.1	-	53	35.039	45
ARC_ART_c32574	135	XP_002972454	0.1	-	63	35.8094	41
ARC_ART_c32594	102	XP_003079234	0.0	-	64	36.965	34

ARC_ART_c32629	115	XP_002974258	0.2	-	63	34.2686	36
ARC_ART_c32633	135	XP_002534897	0.0	GO:0006526	90	73.1738	44
ARC_ART_c32634	134	XP_008669636	0.1	-	58	35.8094	43
ARC_ART_c32704	126	EEE51040	0.0	GO:0044763	73	53.5286	45
ARC_ART_c32707	175	XP_002538051	0.0	GO:0008152	78	76.2554	51
ARC_ART_c32708	116	EEC68421	0.0	GO:0006810	71	50.0618	38
ARC_ART_c32741	118	XP_002536805	0.0	-	57	35.039	38
ARC_ART_c32742	232	YP_001152214	0.0	-	69	41.9726	49
ARC_ART_c32751	164	XP_003055531	1.0	-	55	32.7278	40
ARC_ART_c32754	254	EEE50471	0.0	GO:0005840	100	127.487	65
ARC_ART_c32759	114	XP_002954054	0.0	GO:0050660	79	44.669	34
ARC_ART_c32768	135	XP_007152458	0.4	-	60	33.4982	38
ARC_ART_c32772	168	XP_008360805	0.1	-	51	35.039	49
ARC_ART_c32774	125	NP_038385	0.0	GO:0003899	90	61.2326	40
ARC_ART_c32777	142	XP_001786703	0.0	GO:0016301	75	53.1434	45
ARC_ART_c32809	162	XP_005647217	0.0	GO:0003723	85	88.5817	54
ARC_ART_c32815	151	XP_009387461	0.0	GO:0005840	82	47.3654	34
ARC_ART_c32847	117	EEC78702	0.0	-	84	37.7354	38
ARC_ART_c32849	122	XP_002539974	0.0	-	70	45.4394	41
ARC_ART_c32856	172	CDP04187	0.0	GO:0060560	77	60.077	44
ARC_ART_c32861	105	KEH25776	0.0	-	67	39.2762	34
ARC_ART_c32863	112	EAY84569	0.0	GO:0050660	80	64.6994	36
ARC_ART_c32870	148	EAZ11189	0.0	-	58	39.2762	36
ARC_ART_c32879	230	XP_002949543	0.0	GO:0004028	70	54.6842	41
ARC_ART_c32892	144	EEE68527	0.0	GO:0009982	97	88.9669	47
ARC_ART_c32918	114	XP_001422113	0.6	-	68	32.3426	35
ARC_ART_c32928	116	XP_005651218	0.1	-	64	34.6538	37

ARC_ART_c32929	117	CBI40069	0.0	-	74	51.6026	39
ARC_ART_c32935	112	XP_002534942	0.5	-	65	33.113	35
ARC_ART_c32945	125	EEC75945	0.0	GO:0046872	87	51.9878	41
ARC_ART_c32955	169	XP_002534887	0.0	-	65	43.5134	41
ARC_ART_c32969	118	KEH15262	0.0	GO:0016020	80	49.6766	36
ARC_ART_c32982	147	KEH15576	0.0	-	65	51.9878	49
ARC_ART_c33009	124	ABB55295	0.0	GO:0009166	88	68.9366	42
ARC_ART_c33044	106	XP_011016515	0.0	-	82	53.1434	35
ARC_ART_c33058	131	KFM28820	0.1	-	58	35.4242	36
ARC_ART_c33059	128	KFM22981	0.0	GO:0033388	85	72.0182	42
ARC_ART_c33085	166	CDX94825	0.0	GO:0071704	68	41.9726	47
ARC_ART_c33118	190	EMT00231	0.0	GO:0006732	94	62.7734	37
ARC_ART_c33132	175	CDP20129	0.0	-	50	35.4242	55
ARC_ART_c33151	126	XP_009350051	0.0	-	76	54.299	42
ARC_ART_c33177	171	EMT00263	0.0	GO:0051920	96	107.457	57
ARC_ART_c33184	109	AIT94565	0.0	GO:0017076	64	41.9726	34
ARC_ART_c33191	176	XP_002538274	0.0	-	55	39.2762	54
ARC_ART_c33203	194	XP_006420294	0.0	-	58	36.1946	46
ARC_ART_c33219	123	KFM22980	0.0	-	64	39.6614	39
ARC_ART_c33239	196	BAK00582	0.0	GO:0016740	78	49.2914	38
ARC_ART_c33262	131	XP_002536247	0.0	-	72	40.4318	40
ARC_ART_c33266	117	XP_005846709	0.0	-	57	38.891	38
ARC_ART_c33276	142	EEC78702	0.0	GO:0005774	78	46.9802	37
ARC_ART_c33278	181	XP_001695331	0.0	GO:0006099	81	92.0485	59
ARC_ART_c33291	157	XP_010040582	0.6	-	53	31.9574	41
ARC_ART_c33293	111	XP_002535650	0.0	-	65	35.8094	35
ARC_ART_c33305	272	XP_009355384	0.0	GO:0005739	77	49.2914	35
ARC_ART_c33340	162	XP_006600398	0.4	-	49	34.2686	51

ARC_ART_c33391	108	XP_005644493	0.0	-	73	37.3502	34
ARC_ART_c33421	223	XP_006583774	0.3	-	46	34.6538	62
ARC_ART_c33423	117	XP_006607119	0.0	-	70	36.5798	34
ARC_ART_c33444	139	XP_003590478	0.4	-	51	31.9574	37
ARC_ART_c33448	196	KIZ05685	0.0	-	78	70.4774	51
ARC_ART_c33475	112	XP_005649026	0.0	-	63	35.8094	36
ARC_ART_c33504	130	KDP45666	0.0	-	63	43.1282	36
ARC_ART_c33519	120	AFK47956	0.0	GO:0005488	62	43.1282	37
ARC_ART_c33527	281	XP_011016757	0.0	GO:0043167	68	70.8626	60
ARC_ART_c33609	106	XP_008361909	0.0	-	79	37.7354	34
ARC_ART_c33611	122	KIY96133	0.0	-	65	36.965	40
ARC_ART_c33620	104	XP_002506683	0.0	GO:0016020	70	44.2838	34
ARC_ART_c33657	130	XP_002502994	0.4	-	56	33.4982	41
ARC_ART_c33659	178	XP_004956363	0.0	-	65	36.1946	35
ARC_ART_c33675	162	EDQ49152	0.0	GO:0004871	69	62.7734	53
ARC_ART_c33707	143	XP_009370488	0.0	GO:0071704	74	46.9802	47
ARC_ART_c33708	185	EEC75666	0.0	GO:0006259	66	53.5286	53
ARC_ART_c33712	168	EYU25141	0.0	-	65	48.521	47
ARC_ART_c33720	162	XP_001786407	0.0	-	56	38.1206	46
ARC_ART_c33723	168	AGV54820	0.0	-	78	69.3218	50
ARC_ART_c33733	148	KEH15199	0.0	GO:0016740	75	56.9954	45
ARC_ART_c33738	108	KJB30417	0.9	-	52	32.3426	38
ARC_ART_c33753	148	EAY97568	0.0	GO:0006863	68	52.7582	47
ARC_ART_c33766	121	XP_002539401	0.0	-	64	38.1206	39
ARC_ART_c33824	157	YP_665673	0.0	GO:0070469	84	69.3218	45
ARC_ART_c33826	128	XP_002536644	0.0	-	66	36.965	36
ARC_ART_c33849	174	XP_002971963	0.0	GO:0006457	77	69.707	58

ARC_ART_c33869	172	CEF96686	0.0	GO:0044249	61	46.595	49
ARC_ART_c33874	114	XP_002947533	0.0	GO:0016903	94	73.1738	38
ARC_ART_c33880	169	CAJ55507	0.0	GO:0009684	72	64.6994	62
ARC_ART_c33895	207	KFK32746	0.0	-	57	42.3578	68
ARC_ART_c34078	125	AHI49897	0.0	GO:0098655	95	73.559	41
ARC_ART_c34113	132	XP_002505675	0.4	-	61	33.8834	36
ARC_ART_c34116	148	XP_002539217	0.0	-	65	34.6538	35
ARC_ART_c34123	120	XP_009628953	0.6	-	61	33.113	34
ARC_ART_c34127	130	XP_001422307	0.0	-	69	44.2838	39
ARC_ART_c34150	137	NP_001170188	0.0	GO:0008152	70	45.8246	34
ARC_ART_c34153	114	XP_001754478	0.0	GO:0090305	76	46.595	38
ARC_ART_c34161	204	XP_002535559	0.0	-	73	40.4318	34
ARC_ART_c34187	178	XP_004486558	0.5	-	58	33.4982	36
ARC_ART_c34189	222	XP_002534193	0.4	-	48	31.9574	39
ARC_ART_c34190	104	KFM24979	0.8	-	58	32.3426	34
ARC_ART_c34204	128	XP_008649970	0.8	-	56	32.7278	39
ARC_ART_c34235	121	XP_002537142	0.0	-	60	36.5798	40
ARC_ART_c34284	119	AAW57408	0.0	-	85	54.299	40
ARC_ART_c34289	130	EEC80776	0.0	-	61	38.891	39
ARC_ART_c34343	191	XP_009350805	0.0	GO:0048731	66	67.0106	60
ARC_ART_c34349	139	BAA09812	0.0	GO:0004129	83	48.9062	36
ARC_ART_c34354	162	XP_004506463	0.0	GO:0071704	58	49.2914	60
ARC_ART_c34358	151	EAY95695	0.0	-	55	40.817	45
ARC_ART_c34360	118	KIY93107	0.0	GO:0004658	83	60.8474	37
ARC_ART_c34364	165	XP_002874576	0.5	-	56	33.4982	39
ARC_ART_c34370	114	XP_002509182	0.7	-	50	31.9574	36
ARC_ART_c34379	172	XP_006850918	0.0	-	65	35.8094	38

ARC_ART_c34383	156	CEG01824	0.0	GO:0006098	85	57.3806	35
ARC_ART_c34414	135	ADE75818	0.1	-	62	35.8094	40
ARC_ART_c34445	108	EEE52320	0.0	GO:0055085	100	73.1738	36
ARC_ART_c34458	137	XP_002538873	0.0	-	68	56.6102	45
ARC_ART_c34459	132	KEH26126	0.8	-	52	30.8018	38
ARC_ART_c34472	157	XP_001787045	0.0	GO:0090501	74	53.9138	47
ARC_ART_c34473	208	XP_002945866	0.0	-	64	57.3806	54
ARC_ART_c34504	105	XP_002538475	0.0	GO:0015419	82	53.9138	35
ARC_ART_c34509	152	EAZ22134	0.2	-	56	34.2686	41
ARC_ART_c34512	155	XP_005645281	0.0	-	62	46.9802	51
ARC_ART_c34529	119	XP_002536731	0.0	GO:0016829	72	44.669	37
ARC_ART_c34553	155	KCW58824	0.0	-	64	51.2174	48
ARC_ART_c34562	123	XP_008676709	0.4	-	51	33.113	37
ARC_ART_c34577	156	ABN50032	0.0	-	84	80.8777	50
ARC_ART_c34582	158	KEH15384	0.0	-	56	39.6614	51
ARC_ART_c34606	156	XP_001418694	0.1	-	68	35.8094	35
ARC_ART_c34617	106	AAY78543	0.0	-	64	39.2762	34
ARC_ART_c34621	103	ABN50053	0.0	-	64	35.8094	34
ARC_ART_c34663	151	XP_001787048	0.2	-	71	32.3426	38
ARC_ART_c34681	139	XP_002537329	0.0	-	95	88.9669	46
ARC_ART_c34689	115	XP_001696420	0.0	GO:0042558	80	50.447	36
ARC_ART_c34690	139	EDQ48677	0.0	GO:0008152	91	67.0106	35
ARC_ART_c34694	118	CEF98345	0.4	-	53	33.8834	39
ARC_ART_c34701	123	XP_001760311	0.0	-	66	40.0466	39
ARC_ART_c34703	112	AIY34668	0.0	-	68	41.5874	35
ARC_ART_c34716	154	XP_001779814	0.0	-	60	43.1282	50

ARC_ART_c34725	157	XP_002437343	0.1	-	50	35.4242	36
ARC_ART_c34748	120	XP_004979784	0.2	-	62	34.2686	35
ARC_ART_c34760	137	XP_005644666	0.0	GO:0006810	66	44.2838	42
ARC_ART_c34782	133	NP_038436	0.0	-	62	40.4318	40
ARC_ART_c34784	132	XP_002535353	0.0	GO:0004540	86	55.8398	44
ARC_ART_c34814	192	XP_002536320	0.0	-	77	70.0922	49
ARC_ART_c34817	158	XP_009602476	0.0	-	61	35.4242	42
ARC_ART_c34918	153	BAK02541	0.0	GO:0008270	84	68.5514	44
ARC_ART_c34944	121	CDP09984	0.3	-	61	33.4982	36
ARC_ART_c34982	128	XP_004976895	0.1	-	64	34.2686	37
ARC_ART_c34989	113	EMT33665	0.4	-	56	33.113	37
ARC_ART_c35005	123	NP_001042890	0.0	GO:0009646	91	58.9214	35
ARC_ART_c35039	141	XP_002963407	1.0	-	56	32.3426	44
ARC_ART_c35058	118	XP_002989827	0.0	-	57	36.5798	35
ARC_ART_c35060	130	XP_002535893	0.0	-	74	56.9954	43
ARC_ART_c35113	139	KIY98411	0.0	-	59	36.5798	47
ARC_ART_c35127	108	XP_002948160	0.1	-	61	34.6538	34
ARC_ART_c35135	134	CDP16362	0.2	-	57	34.2686	40
ARC_ART_c35156	233	XP_005648087	0.2	-	51	35.039	56
ARC_ART_c35166	168	XP_001692596	0.0	-	59	45.4394	49
ARC_ART_c35185	110	EEC69860	0.2	-	66	34.2686	36
ARC_ART_c35195	125	XP_002539614	0.0	-	89	67.0106	38
ARC_ART_c35205	134	KIY96607	0.0	-	55	36.965	43
ARC_ART_c35213	103	XP_003082535	0.0	-	67	39.6614	34
ARC_ART_c35224	114	XP_006852122	0.0	-	71	40.817	38

ARC_ART_c35234	122	XP_001773680	0.0	GO:0006401	62	41.9726	37
ARC_ART_c35240	136	XP_002539959	0.0	-	66	38.5058	39
ARC_ART_c35281	110	EMT00236	0.0	GO:0005737	86	59.3066	36
ARC_ART_c35307	105	XP_002535255	0.0	-	61	39.6614	34
ARC_ART_c35323	115	EEE69878	0.0	GO:0042578	78	51.9878	37
ARC_ART_c35335	127	ABS10724	0.0	GO:0016491	87	70.4774	41
ARC_ART_c35339	128	XP_003081071	0.6	-	53	32.7278	41
ARC_ART_c35342	126	XP_010445296	0.0	-	53	39.2762	41
ARC_ART_c35347	104	XP_002536317	0.0	GO:0050794	70	49.6766	34
ARC_ART_c35408	121	CCO19022	0.2	-	72	34.2686	36
ARC_ART_c35446	118	XP_001787063	0.1	-	53	34.6538	39
ARC_ART_c35454	139	XP_009350808	0.0	-	75	41.9726	37
ARC_ART_c35471	177	XP_002538077	0.0	-	45	40.0466	53
ARC_ART_c35483	111	XP_002535455	0.0	GO:0046872	85	59.3066	35
ARC_ART_c35495	131	KEH15262	0.0	GO:0006094	81	56.9954	37
ARC_ART_c35566	142	XP_002538009	0.0	GO:0052655	93	87.0409	46
ARC_ART_c35592	123	XP_003064708	0.0	GO:0005829	100	96.6709	40
ARC_ART_c35639	179	KDD73633	0.6	-	62	33.113	40
ARC_ART_c35641	161	ABA94619	0.8	-	46	31.187	54
ARC_ART_c35660	132	EEC76966	0.0	-	71	45.0542	38
ARC_ART_c35669	146	XP_008369634	1.0	-	70	31.5722	34
ARC_ART_c35682	142	XP_010271366	0.5	-	48	33.4982	37
ARC_ART_c35685	238	NP_199834	0.8	-	57	33.4982	47
ARC_ART_c35687	145	KIY99354	0.0	-	61	38.1206	34
ARC_ART_c35706	104	AFW80434	1.0	-	71	31.9574	35
ARC_ART_c35723	148	XP_003062944	0.0	GO:0016301	76	42.743	34
ARC_ART_c35730	166	EAY93131	0.0	GO:0044763	75	53.9138	52
ARC_ART_c35736	141	XP_001760228	0.2	-	57	33.113	35
ARC_ART_c35738	129	XP_011015412	0.0	-	58	39.6614	41
ARC_ART_c35764	133	EEC77198	0.0	GO:0044763	65	44.2838	38

ARC_ART_c35766	277	ABA95992	0.1	-	50	36.1946	40
ARC_ART_c35801	164	XP_009336934	0.5	-	63	33.113	36
ARC_ART_c35827	139	XP_005651876	0.2	-	60	34.2686	35
ARC_ART_c35846	153	XP_003055705	0.0	GO:0004672	77	43.5134	35
ARC_ART_c35864	133	XP_001786771	0.0	GO:0006810	68	43.5134	35
ARC_ART_c35912	112	XP_002499911	0.0	GO:0030170	81	62.003	37
ARC_ART_c35942	122	KIY99730	0.0	GO:0004400	73	48.1358	38
ARC_ART_c35953	133	XP_002535918	0.0	GO:0015937	84	55.0694	44
ARC_ART_c35957	179	XP_001781548	0.0	GO:0016787	71	56.9954	49
ARC_ART_c35971	168	YP_025795	0.0	GO:0044425	75	43.5134	37
ARC_ART_c36001	171	ABR26094	0.0	-	73	73.559	57
ARC_ART_c36004	120	EAZ02189	0.0	-	65	44.2838	38
ARC_ART_c36025	136	CEF99881	0.0	GO:0055114	83	64.3142	43
ARC_ART_c36049	132	XP_001786687	0.0	GO:0006810	64	43.5134	50
ARC_ART_c36055	152	XP_002534826	0.4	-	59	33.4982	49
ARC_ART_c36064	172	YP_009106593	0.0	GO:0016740	72	61.6178	51
ARC_ART_c36146	172	CDP22238	0.0	-	60	43.1282	56
ARC_ART_c36152	121	EAY81384	0.0	GO:0055114	89	70.8626	39
ARC_ART_c36168	307	ABR26094	0.0	-	57	43.8986	56
ARC_ART_c36172	117	AAB22587	0.0	GO:0000166	75	46.2098	37
ARC_ART_c36245	164	XP_001699711	0.0	-	68	35.8094	41
ARC_ART_c36290	126	KFM25080	0.1	-	65	35.8094	40
ARC_ART_c36345	117	XP_002538816	0.0	-	59	38.1206	37
ARC_ART_c36398	142	ACJ24144	0.0	GO:0003899	89	72.7886	47
ARC_ART_c36457	133	XP_002535812	0.0	GO:0000155	82	50.0618	40
ARC_ART_c36490	169	XP_002535693	0.1	-	69	35.8094	39
ARC_ART_c36504	125	XP_003056436	0.0	GO:0003861	82	59.6918	40
ARC_ART_c36560	154	AFK39808	0.0	GO:0005509	89	70.0922	46
ARC_ART_c36577	113	XP_001786799	0.0	-	62	36.1946	35

ARC_ART_c36582	126	EEC75945	0.0	GO:0000166	76	41.5874	34
ARC_ART_c36638	108	ABH07502	0.0	GO:0005524	85	51.2174	35
ARC_ART_c36666	109	EMS56529	0.0	GO:0016491	100	71.2478	36
ARC_ART_c36688	103	XP_002538427	0.0	-	61	40.0466	34
ARC_ART_c36706	133	XP_005847592	0.0	GO:0008152	88	78.1814	44
ARC_ART_c36743	121	XP_001689842	0.0	GO:0022900	79	53.1434	34
ARC_ART_c36765	155	XP_002540004	0.0	-	59	42.3578	44
ARC_ART_c36805	127	XP_003081977	0.0	-	70	39.2762	40
ARC_ART_c36823	127	CDY06058	0.5	-	54	32.7278	37
ARC_ART_c36824	111	XP_011016515	0.0	-	77	44.2838	36
ARC_ART_c36881	149	XP_002537910	0.0	-	61	37.3502	47
ARC_ART_c36882	122	XP_011079800	0.0	GO:0008270	80	53.5286	40
ARC_ART_c36911	120	XP_002538058	0.0	GO:0005774	79	49.6766	39
ARC_ART_c36914	132	P00064	0.0	GO:0046872	72	40.0466	36
ARC_ART_c36937	109	XP_002536027	0.0	GO:0005975	82	52.7582	35
ARC_ART_c36976	105	XP_003602329	0.6	-	57	30.8018	35
ARC_ART_c36981	152	KDD72849	0.0	GO:0016740	61	46.9802	55
ARC_ART_c37043	133	XP_006404171	0.0	-	58	37.3502	36
ARC_ART_c37088	109	XP_002501695	0.0	GO:0016874	65	43.1282	35
ARC_ART_c37097	120	EMT26011	0.1	-	73	35.4242	34
ARC_ART_c37150	101	XP_011015255	1.0	-	64	31.9574	34
ARC_ART_c37186	122	XP_002536858	0.0	-	65	36.5798	35
ARC_ART_c37210	140	XP_002535353	0.0	-	67	40.4318	43
ARC_ART_c37220	159	NP_001132894	0.5	-	55	33.113	40
ARC_ART_c37274	137	XP_003082927	0.0	-	80	44.2838	36
ARC_ART_c37353	134	EEC80856	0.0	GO:0050660	94	70.8626	38
ARC_ART_c37366	120	XP_002535087	0.0	-	67	47.7506	37
ARC_ART_c37377	149	XP_002536223	0.0	GO:0006810	80	59.3066	46
ARC_ART_c37423	128	NP_001066619	0.2	-	62	33.4982	35

ARC_ART_c37424	136	XP_001786892	0.0	-	60	38.1206	43
ARC_ART_c37441	156	XP_002506612	0.5	-	50	33.4982	34
ARC_ART_c37456	105	XP_002536731	0.0	GO:0016829	76	42.3578	34
ARC_ART_c37467	185	AAO86692	0.5	-	50	33.113	40
ARC_ART_c37540	192	EYU46721	0.0	-	55	36.5798	38
ARC_ART_c37550	146	XP_002539701	0.0	-	56	40.4318	46
ARC_ART_c37575	155	KEH15495	0.0	-	59	38.1206	47
ARC_ART_c37585	164	EMS60082	0.6	-	53	32.7278	39
ARC_ART_c37612	128	CCO17359	0.0	-	62	36.965	35
ARC_ART_c37631	150	XP_008788653	0.0	GO:0008236	76	56.6102	38
ARC_ART_c37656	150	XP_002535085	0.0	-	58	39.6614	46
ARC_ART_c37720	174	XP_001700521	0.2	-	50	34.2686	54
ARC_ART_c37803	174	CAN67345	0.0	GO:0005829	66	51.6026	51
ARC_ART_c37910	176	XP_002538660	0.0	-	73	53.1434	42
ARC_ART_c37914	110	KDD73114	0.0	GO:0004018	86	63.5438	36
ARC_ART_c37995	121	XP_002536193	0.0	GO:0006810	62	44.669	37
ARC_ART_c38014	113	XP_002985384	0.0	GO:0009414	70	44.669	37
ARC_ART_c38089	192	XP_001787018	0.1	-	54	29.261	42
ARC_ART_c38102	138	XP_001415637	0.0	GO:0008233	92	67.3958	38
ARC_ART_c38119	134	EDQ48365	0.0	GO:0006355	79	51.6026	39
ARC_ART_c38131	110	XP_002951751	0.0	GO:0006098	100	80.1073	36
ARC_ART_c38157	172	ABA06483	0.0	-	71	49.6766	38
ARC_ART_c38188	110	XP_001783885	0.0	GO:0019538	73	45.0542	34
ARC_ART_c38197	114	XP_009365048	0.0	GO:0051536	74	41.2022	35
ARC_ART_c38226	143	EEC75945	0.0	-	71	40.817	38
ARC_ART_c38235	150	EDQ49163	0.0	-	76	54.299	34
ARC_ART_c38281	155	AAD39440	0.9	-	54	32.7278	37

ARC_ART_c38301	172	XP_003058469	0.1	-	57	35.4242	35
ARC_ART_c38365	252	XP_009400268	0.0	-	56	37.7354	48
ARC_ART_c38374	106	XP_010229654	0.7	-	60	32.7278	38
ARC_ART_c38402	108	XP_006360301	0.8	-	61	32.3426	34
ARC_ART_c38473	132	XP_002946019	0.0	-	75	40.817	36
ARC_ART_c38494	111	XP_002538398	0.0	GO:0004871	75	44.2838	36
ARC_ART_c38497	105	XP_001701777	0.0	GO:0008233	82	49.6766	34
ARC_ART_c38503	170	CBI27372	0.0	GO:0016798	80	61.6178	40
ARC_ART_c38587	112	EEC76122	0.0	GO:0050660	86	55.8398	37
ARC_ART_c38595	120	KJB53845	0.0	GO:0005737	85	66.6254	40
ARC_ART_c38619	153	XP_002539909	0.0	-	71	40.0466	35
ARC_ART_c38651	118	XP_002970482	0.8	-	57	31.9574	38
ARC_ART_c38697	128	XP_002535080	0.0	-	62	37.3502	37
ARC_ART_c38753	109	XP_002501803	0.3	-	67	33.4982	37
ARC_ART_c38779	109	KIY95787	0.2	-	62	33.8834	35
ARC_ART_c38825	117	XP_009350065	0.0	-	81	51.9878	37
ARC_ART_c38953	141	XP_001782658	0.0	-	59	38.891	37
ARC_ART_c38956	186	XP_007150103	0.0	-	55	40.0466	54
ARC_ART_c38995	102	XP_008437253	0.0	-	73	40.4318	34
ARC_ART_c39043	104	XP_002537671	0.1	-	68	34.6538	35
ARC_ART_c39048	143	XP_002535739	0.1	-	66	34.6538	45
ARC_ART_c39143	126	XP_009420964	0.0	GO:0006139	75	51.9878	44
ARC_ART_c39148	145	XP_002537786	0.6	-	53	31.9574	43
ARC_ART_c39243	107	XP_002534563	0.0	GO:0016661	73	46.595	34
ARC_ART_c39308	119	XP_002507709	0.0	GO:0003861	94	68.9366	35
ARC_ART_c39317	126	XP_009148435	0.8	-	58	32.7278	34
ARC_ART_c39323	144	EEC79493	0.0	-	60	40.4318	46

ARC_ART_c39358	168	ABK26991	0.0	-	63	41.5874	38
ARC_ART_c39504	175	KFM26772	0.0	GO:0044763	72	61.6178	47
ARC_ART_c39507	164	KIZ00400	0.0	-	69	41.9726	36
ARC_ART_c39545	149	XP_002954670	0.4	-	50	33.4982	42
ARC_ART_c39596	118	XP_001786562	0.0	-	66	41.2022	36
ARC_ART_c39604	169	NP_001142241	0.3	-	56	33.8834	39
ARC_ART_c39623	178	XP_011467651	0.1	-	57	35.039	49
ARC_ART_c39635	206	CDY43221	0.0	-	62	47.7506	51
ARC_ART_c39641	149	EDQ48662	0.0	GO:0009407	79	58.151	49
ARC_ART_c39698	206	KJB14970	0.1	-	50	35.8094	52
ARC_ART_c39721	109	XP_002534942	0.0	-	85	63.929	35
ARC_ART_c39736	143	EEC76917	0.0	-	55	41.5874	45
ARC_ART_c39742	169	EEE67872	0.0	-	92	73.9442	38
ARC_ART_c39756	154	BAD38278	0.6	-	61	30.8018	34
ARC_ART_c39810	116	EEE52320	0.0	GO:0055085	100	77.7962	38
ARC_ART_c39816	112	XP_002537571	0.0	GO:0016020	72	43.5134	37
ARC_ART_c39930	108	XP_009414007	0.2	-	60	33.8834	35
ARC_ART_c39960	107	XP_005649738	0.0	GO:0006085	80	56.9954	35
ARC_ART_c39977	129	CDX73036	0.8	-	56	31.5722	39
ARC_ART_c39980	140	XP_002947189	0.0	-	59	36.5798	42
ARC_ART_c40047	288	XP_004975365	0.3	-	59	35.4242	37
ARC_ART_c40088	248	EAY93455	0.0	-	55	46.595	84
ARC_ART_c40163	129	XP_010472421	0.0	-	67	40.817	43
ARC_ART_c40171	144	KJB40407	0.0	-	66	50.8322	45
ARC_ART_c40195	126	XP_011014253	0.0	-	75	39.2762	37
ARC_ART_c40218	114	YP_006666409	0.2	-	70	33.4982	40

ARC_ART_c40230	156	XP_008645081	0.3	-	43	34.2686	41
ARC_ART_c40231	121	XP_002535282	0.0	GO:0017111	83	55.8398	37
ARC_ART_c40307	132	XP_006838399	0.0	GO:0005488	64	49.2914	42
ARC_ART_c40334	144	CCO16830	0.0	GO:0050660	80	60.077	35
ARC_ART_c40365	156	XP_002501803	0.0	GO:0097159	68	51.6026	41
ARC_ART_c40372	159	XP_006838012	0.0	-	65	40.817	38
ARC_ART_c40446	138	XP_002536292	0.0	GO:0046872	79	70.0922	43
ARC_ART_c40464	120	XP_002956892	0.0	GO:0005960	81	57.3806	37
ARC_ART_c40475	177	KEH42128	0.1	-	51	35.4242	60
ARC_ART_c40507	193	XP_003074121	0.0	GO:0016301	75	78.5666	61
ARC_ART_c40695	145	XP_002537273	0.0	-	65	48.521	44
ARC_ART_c40700	216	XP_001786560	0.0	GO:0006289	81	44.2838	37
ARC_ART_c40729	147	XP_004511876	0.5	-	53	33.4982	39
ARC_ART_c40745	108	XP_002890263	0.3	-	48	33.8834	35
ARC_ART_c40763	146	XP_002537742	0.0	-	95	44.669	48
ARC_ART_c40781	122	XP_002534501	0.0	GO:0044710	67	43.8986	40
ARC_ART_c40785	123	BAA23186	0.2	-	60	31.9574	38
ARC_ART_c40807	103	XP_010042550	0.2	-	64	33.8834	34
ARC_ART_c40825	113	XP_002536769	0.6	-	60	32.3426	35
ARC_ART_c40878	102	XP_010911538	0.0	-	82	58.5362	34
ARC_ART_c40890	118	KDP43088	0.0	-	66	38.891	36
ARC_ART_c40908	134	XP_002535763	0.0	-	60	42.743	41
ARC_ART_c40920	120	XP_006290873	0.0	GO:0004028	75	53.1434	40
ARC_ART_c40940	114	XP_006411004	0.0	GO:0009941	77	46.2098	35
ARC_ART_c40959	132	XP_008233332	0.0	GO:0044238	62	44.669	37

ARC_ART_c41012	113	XP_006430210	0.6	-	57	33.113	35
ARC_ART_c41031	146	EEC68227	0.0	GO:0008152	100	46.595	37
ARC_ART_c41062	143	XP_011016263	0.0	GO:0016149	85	51.2174	34
ARC_ART_c41084	121	BAJ99459	0.0	GO:0008152	77	56.6102	40
ARC_ART_c41263	172	EMS47143	0.8	-	64	33.113	39
ARC_ART_c41273	116	XP_002536045	0.0	GO:0006355	84	57.3806	38
ARC_ART_c41282	174	NP_001176231	0.0	-	62	38.1206	35
ARC_ART_c41298	152	XP_011014724	0.0	GO:0016772	100	90.8929	44
ARC_ART_c41309	130	EDQ48354	0.0	-	64	36.5798	39
ARC_ART_c41319	103	XP_001784257	0.0	GO:0009507	85	54.299	34
ARC_ART_c41326	165	XP_002536223	0.0	GO:0016020	74	66.2402	55
ARC_ART_c41352	131	XP_010499191	0.5	-	67	33.113	34
ARC_ART_c41355	125	XP_011016603	0.0	GO:0004591	95	80.8777	41
ARC_ART_c41361	135	XP_001787011	0.0	-	69	37.3502	39
ARC_ART_c41398	167	EEC78702	0.0	-	69	61.6178	56
ARC_ART_c41472	129	XP_002535096	0.0	-	64	38.891	39
ARC_ART_c41492	121	AFK46877	0.0	-	71	40.4318	39
ARC_ART_c41499	112	XP_007213193	0.4	-	52	33.113	34
ARC_ART_c41525	105	KIY97402	0.0	-	70	38.1206	34
ARC_ART_c41531	152	KIZ02942	0.0	GO:0005524	97	88.1965	46
ARC_ART_c41612	107	XP_009616447	0.0	GO:0007010	88	68.5514	35
ARC_ART_c41618	132	XP_002270819	0.8	-	58	31.9574	36
ARC_ART_c41655	119	XP_011016735	0.0	-	72	46.9802	37
ARC_ART_c41735	201	KFM27378	0.0	-	61	45.4394	60
ARC_ART_c41738	112	XP_002535230	0.0	-	88	65.4698	35
ARC_ART_c41747	140	XP_002534896	0.0	GO:0044237	68	48.9062	45
ARC_ART_c41766	115	AFK33618	0.0	GO:0004550	80	46.9802	36

ARC_ART_c41776	127	BAG91391	0.4	-	44	33.4982	38
ARC_ART_c41853	123	YP_009106588	0.1	-	67	35.039	34
ARC_ART_c41893	127	XP_011022251	0.1	-	66	35.039	36
ARC_ART_c41918	156	XP_003063914	0.8	-	55	32.7278	45
ARC_ART_c41953	111	XP_002537783	0.0	-	68	36.965	35
ARC_ART_c41956	126	XP_011014723	0.0	-	68	49.6766	41
ARC_ART_c41988	107	XP_009350056	0.0	-	97	68.9366	35
ARC_ART_c41995	187	XP_009121197	0.9	-	56	33.113	53
ARC_ART_c42032	120	XP_007161011	0.0	GO:0006457	91	63.5438	36
ARC_ART_c42052	121	AGG09511	0.0	GO:0003899	92	73.1738	39
ARC_ART_c42069	150	EEE52322	0.0	GO:0016020	100	103.99	49
ARC_ART_c42097	120	XP_004502946	0.3	-	76	33.8834	34
ARC_ART_c42154	118	EDQ48523	0.0	GO:0043167	76	45.4394	34
ARC_ART_c42160	109	EEC77997	0.0	GO:0042450	97	70.8626	36
ARC_ART_c42168	112	XP_002536712	0.0	GO:0004089	80	48.521	35
ARC_ART_c42247	130	YP_005089771	0.1	-	64	33.8834	37
ARC_ART_c42302	107	EMT12933	0.8	-	70	31.9574	34
ARC_ART_c42380	153	EEC77110	0.0	GO:0003824	72	55.4546	43
ARC_ART_c42398	162	XP_005648607	0.0	-	67	40.0466	46
ARC_ART_c42424	114	XP_002537394	0.0	-	71	44.2838	35
ARC_ART_c42457	109	XP_002958165	0.0	GO:0008152	65	48.521	35
ARC_ART_c42459	123	XP_002539624	0.0	GO:0031418	87	65.855	40
ARC_ART_c42462	114	XP_002537020	0.0	GO:0003723	76	47.3654	34
ARC_ART_c42491	124	YP_636219	0.0	GO:0003723	75	42.743	40
ARC_ART_c42503	107	XP_003574655	0.0	GO:0046872	85	50.0618	34
ARC_ART_c42554	143	XP_001691612	0.0	-	67	44.2838	37
ARC_ART_c42568	114	KIY96778	0.0	GO:0044260	73	48.1358	38
ARC_ART_c42582	117	KIZ03037	0.0	GO:0005789	79	52.7582	34
ARC_ART_c42594	125	EEE52319	0.0	GO:0006378	91	70.0922	35

ARC_ART_c42608	108	EEC80857	0.0	GO:0016020	91	60.077	35
ARC_ART_c42616	125	KFM27215	0.0	-	70	36.1946	40
ARC_ART_c42618	166	EEC74086	0.3	-	57	34.2686	38
ARC_ART_c42631	136	XP_002501855	0.0	GO:0055114	79	66.2402	43
ARC_ART_c42640	117	XP_002537200	0.2	-	52	33.8834	38
ARC_ART_c42661	129	XP_011016509	0.0	-	88	48.1358	42
ARC_ART_c42682	134	XP_006588502	0.4	-	67	33.4982	34
ARC_ART_c42733	115	Q9G4F5	0.0	GO:0009536	76	49.6766	38
ARC_ART_c42735	142	XP_002537480	0.0	GO:0009507	67	46.9802	40
ARC_ART_c42739	147	XP_002539165	0.1	-	54	34.6538	48
ARC_ART_c42749	145	XP_003082900	0.0	GO:0016842	76	66.2402	47
ARC_ART_c42769	114	KDO45760	0.0	GO:0000166	77	41.5874	35
ARC_ART_c42786	120	XP_003054851	0.0	-	63	37.3502	41
ARC_ART_c42821	147	XP_002535815	0.0	-	64	49.2914	45
ARC_ART_c42885	135	CDY03402	0.7	-	57	30.4166	35
ARC_ART_c42916	160	XP_002538551	0.0	GO:0004609	96	108.227	53
ARC_ART_c42982	119	AEC11026	0.0	-	71	38.1206	35
ARC_ART_c42998	128	XP_011034379	0.0	GO:0071704	82	59.3066	39
ARC_ART_c43034	142	XP_006300621	0.5	-	61	33.4982	34
ARC_ART_c43095	130	XP_009415666	0.3	-	62	33.8834	37
ARC_ART_c43126	124	XP_006305843	0.0	GO:0071704	66	43.8986	39
ARC_ART_c43148	174	XP_008651362	0.0	GO:0044237	57	43.1282	54
ARC_ART_c43160	174	BAC21362	0.3	-	49	34.2686	53
ARC_ART_c43174	119	XP_002536940	0.0	-	60	38.891	38
ARC_ART_c43204	152	XP_002535387	0.0	GO:0008152	86	58.9214	36
ARC_ART_c43308	105	XP_002509137	0.0	GO:0046872	82	46.595	34
ARC_ART_c43318	164	XP_010911369	0.0	GO:0044763	76	53.1434	38

ARC_ART_c43358	103	EEC83292	0.0	GO:0016021	91	72.7886	34
ARC_ART_c43360	126	KIZ03435	0.0	-	67	44.669	37
ARC_ART_c43377	127	XP_009338823	0.4	-	55	33.4982	47
ARC_ART_c43409	114	Q08632	0.0	-	68	41.2022	35
ARC_ART_c43447	156	ERN14440	0.6	-	61	32.3426	36
ARC_ART_c43458	140	XP_003574467	0.5	-	52	33.4982	40
ARC_ART_c43478	105	EDQ48557	0.0	-	70	35.4242	34
ARC_ART_c43486	130	XP_002948755	0.0	-	61	36.1946	42
ARC_ART_c43517	115	CDY35054	0.2	-	70	32.3426	34
ARC_ART_c43550	156	XP_009350072	0.0	GO:0043231	70	56.9954	50
ARC_ART_c43612	110	XP_008378683	0.4	-	66	33.113	36
ARC_ART_c43624	142	EEC76877	0.0	GO:0046961	91	71.633	46
ARC_ART_c43641	141	XP_002537608	0.0	-	64	44.2838	45
ARC_ART_c43680	205	XP_002952128	0.9	-	56	33.113	48
ARC_ART_c43700	120	XP_004984619	1.0	-	59	32.3426	37
ARC_ART_c43818	113	KFK30961	0.0	-	66	49.2914	45
ARC_ART_c43858	141	XP_002951042	0.0	GO:0001101	69	52.373	46
ARC_ART_c43885	136	XP_002535080	0.0	GO:0016020	85	54.6842	34
ARC_ART_c43903	117	XP_006434143	0.0	-	74	41.9726	35
ARC_ART_c43915	156	XP_002993245	0.0	-	64	40.817	42
ARC_ART_c43937	123	XP_008465557	0.5	-	60	32.7278	40
ARC_ART_c43965	161	XP_004235066	0.0	GO:0008270	78	60.8474	41
ARC_ART_c43997	144	XP_002500812	0.0	GO:0051903	83	53.1434	36
ARC_ART_c44028	126	XP_008239705	0.0	GO:0016620	72	51.9878	40
ARC_ART_c44125	131	KEH15576	0.0	-	60	45.8246	43

ARC_ART_c44148	124	KIZ03094	0.2	-	55	34.2686	34
ARC_ART_c44152	180	XP_003525679	0.3	-	53	34.2686	39
ARC_ART_c44214	120	AAA33485	0.0	GO:0016620	69	40.817	36
ARC_ART_c44254	129	KFM27378	0.0	-	74	61.2326	43
ARC_ART_c44274	195	XP_008368720	0.3	-	47	34.2686	59
ARC_ART_c44282	192	YP_001312258	0.2	-	48	32.7278	45
ARC_ART_c44283	125	YP_009057529	0.0	-	73	37.3502	34
ARC_ART_c44370	177	XP_002967373	0.6	-	54	33.113	50
ARC_ART_c44468	179	KDD72882	0.0	GO:0005829	84	67.3958	38
ARC_ART_c44475	111	CDP16278	0.0	GO:0005829	97	73.1738	36
ARC_ART_c44489	105	XP_001698312	0.1	-	67	35.039	34
ARC_ART_c44514	178	XP_003620715	0.0	-	51	36.5798	49
ARC_ART_c44520	138	XP_002507106	0.7	-	48	32.7278	41
ARC_ART_c44588	115	XP_003610132	0.3	-	70	33.8834	37
ARC_ART_c44627	146	XP_003061168	0.0	GO:0009113	82	48.521	34
ARC_ART_c44807	129	XP_002536395	0.0	-	67	43.5134	37
ARC_ART_c44820	109	XP_003588355	0.0	-	97	72.4034	35
ARC_ART_c44821	125	XP_001703001	0.0	GO:0016491	65	43.5134	38
ARC_ART_c44850	105	XP_002538157	0.0	GO:0016829	82	56.225	34
ARC_ART_c44882	147	XP_003080027	0.0	-	65	46.9802	43
ARC_ART_c44887	112	XP_002534890	0.0	-	72	45.8246	36
ARC_ART_c44923	146	KFM28384	0.0	-	64	41.2022	34
ARC_ART_c44927	130	XP_002536317	0.4	-	64	33.113	34
ARC_ART_c44935	140	EMT14373	0.3	-	56	34.2686	37
ARC_ART_c44970	174	XP_002536310	0.0	-	70	56.9954	51
ARC_ART_c44982	184	XP_009405677	0.0	GO:0005996	66	52.7582	45

ARC_ART_c45007	114	XP_002964596	0.0	GO:0005829	80	56.6102	36
ARC_ART_c45013	106	EEE68527	0.0	GO:0009982	100	77.411	35
ARC_ART_c45014	123	XP_001786687	0.0	-	75	55.8398	41
ARC_ART_c45024	114	XP_002534872	0.2	-	70	33.8834	34
ARC_ART_c45060	117	ACQ90797	0.0	-	72	39.6614	40
ARC_ART_c45064	128	KEH15263	0.0	-	100	86.2705	42
ARC_ART_c45107	137	EEE50470	0.0	GO:0005524	97	68.9366	34
ARC_ART_c45129	108	NP_038385	0.0	GO:0003899	88	67.3958	34
ARC_ART_c45174	136	XP_002499629	0.0	-	57	42.743	45
ARC_ART_c45180	143	XP_004963609	0.0	GO:0019676	86	79.337	45
ARC_ART_c45192	106	EEC69598	0.3	-	59	33.4982	37
ARC_ART_c45195	118	XP_002539783	0.0	-	68	39.6614	35
ARC_ART_c45215	134	EEC70906	0.0	-	62	39.2762	35
ARC_ART_c45234	146	ACG27180	0.0	GO:0016491	80	63.929	41
ARC_ART_c45236	150	XP_001690617	0.0	-	61	42.743	44
ARC_ART_c45280	101	XP_010911626	0.0	-	64	35.4242	37
ARC_ART_c45300	104	XP_002539707	0.0	-	67	37.3502	34
ARC_ART_c45305	191	XP_002536031	0.0	GO:0015942	91	100.138	57
ARC_ART_c45348	115	EEE52320	0.0	GO:0006094	100	81.6481	37
ARC_ART_c45373	138	EAY97777	0.0	GO:0009058	82	65.855	45
ARC_ART_c45390	105	XP_001787129	0.0	-	67	37.7354	34
ARC_ART_c45405	119	XP_009350056	0.0	-	82	52.7582	39
ARC_ART_c45463	120	EEE67873	0.0	-	71	47.3654	38
ARC_ART_c45502	160	XP_002539880	0.0	-	56	45.0542	48
ARC_ART_c45508	114	XP_001786976	0.0	GO:0016020	68	41.2022	35
ARC_ART_c45531	140	XP_002539069	0.1	-	70	35.039	37

ARC_ART_c45585	136	XP_009351044	0.0	-	53	38.1206	43
ARC_ART_c45590	113	EEC77966	0.0	GO:0005524	100	65.4698	37
ARC_ART_c45622	124	EPS60518	0.9	-	51	31.9574	41
ARC_ART_c45645	114	XP_003561813	0.4	-	51	33.113	37
ARC_ART_c45655	124	XP_002874779	0.9	-	64	31.187	42
ARC_ART_c45673	118	DAA40622	0.0	GO:0004252	89	68.9366	39
ARC_ART_c45686	141	XP_006836376	0.2	-	66	34.6538	36
ARC_ART_c45690	103	KDD73418	0.0	-	76	37.7354	34
ARC_ART_c45700	104	EAY97777	0.0	GO:0071704	73	44.669	34
ARC_ART_c45754	129	XP_001696745	0.0	-	68	38.5058	38
ARC_ART_c45768	122	EYU22484	0.8	-	52	32.3426	38
ARC_ART_c45805	194	CDP16683	0.0	GO:0004849	75	62.003	52
ARC_ART_c45813	227	XP_002535543	0.0	-	71	59.3066	52
ARC_ART_c45822	147	CBI28493	0.3	-	43	33.4982	41
ARC_ART_c45843	125	KIY94849	0.5	-	52	32.7278	36
ARC_ART_c45893	178	CAD24034	0.9	-	53	30.8018	39
ARC_ART_c45894	109	EEC68395	0.0	GO:0003824	76	46.595	34
ARC_ART_c45920	141	XP_002537218	0.0	-	78	53.1434	42
ARC_ART_c45959	142	XP_005649397	0.0	GO:0043231	75	62.003	40
ARC_ART_c45966	105	EEE52320	0.0	-	70	36.1946	34
ARC_ART_c46035	112	BAD16220	0.9	-	56	30.8018	37
ARC_ART_c46063	108	XP_004308689	0.0	-	60	41.2022	41
ARC_ART_c46068	101	XP_003554124	0.0	GO:0006139	77	49.6766	35
ARC_ART_c46080	111	XP_002539231	0.1	-	69	35.4242	36
ARC_ART_c46099	115	EEE51040	0.0	GO:0005507	91	54.299	35
ARC_ART_c46109	101	XP_005651758	0.0	-	65	36.1946	41

ARC_ART_c46114	178	XP_002538363	0.0	-	55	43.8986	59
ARC_ART_c46129	107	EEE52320	0.0	GO:0055085	100	71.633	34
ARC_ART_c46142	118	XP_006646646	0.8	-	57	31.9574	35
ARC_ART_c46174	114	YP_001019092	0.0	GO:0005840	78	60.8474	38
ARC_ART_c46180	123	XP_002538815	0.0	-	75	56.225	41
ARC_ART_c46185	154	XP_007146879	0.7	-	64	32.3426	34
ARC_ART_c46202	123	XP_002505157	0.0	GO:0051213	82	52.7582	40
ARC_ART_c46204	112	XP_002945838	0.0	GO:0046872	74	41.2022	35
ARC_ART_c46205	114	XP_010028115	0.0	GO:0030127	80	49.2914	36
ARC_ART_c46252	148	KEH15384	0.0	GO:0003824	63	46.9802	49
ARC_ART_c46287	163	XP_006851877	0.0	GO:0016020	74	60.8474	54
ARC_ART_c46322	133	XP_002980177	0.0	GO:0005488	68	50.8322	38
ARC_ART_c46323	114	AJM90199	0.0	GO:0003735	81	55.0694	38
ARC_ART_c46341	237	XP_002540477	0.0	-	57	51.9878	64
ARC_ART_c46364	218	ABK21234	0.2	-	42	34.6538	69
ARC_ART_c46396	105	NP_045832	0.0	GO:0016020	70	40.817	34
ARC_ART_c46397	120	XP_011016737	0.0	-	100	85.8853	39
ARC_ART_c46398	122	XP_001767123	0.7	-	64	32.7278	39
ARC_ART_c46425	228	XP_002502671	0.0	GO:0005215	66	38.5058	36
ARC_ART_c46444	158	XP_001762084	0.0	-	58	41.2022	34
ARC_ART_c46448	181	AAO86633	0.3	-	45	33.8834	42
ARC_ART_c46505	130	XP_007042908	0.0	GO:0071702	82	50.447	40
ARC_ART_c46509	122	XP_005843752	0.0	GO:0003676	76	50.8322	39
ARC_ART_c46557	109	XP_009350810	0.0	-	80	55.0694	36
ARC_ART_c46616	160	EEC73533	0.5	-	62	33.4982	40
ARC_ART_c46654	130	XP_001754925	0.0	GO:0071704	58	56.9954	53

ARC_ART_c46655	105	KJB65191	0.0	GO:0006310	85	54.6842	34
ARC_ART_c46677	143	XP_002538900	0.0	-	58	41.9726	41
ARC_ART_c46698	144	XP_002538705	0.5	-	55	31.187	47
ARC_ART_c46725	103	AAZ78350	0.0	-	76	46.9802	34
ARC_ART_c46734	130	AAT74596	0.0	GO:0051287	78	56.225	42
ARC_ART_c46795	150	KEH15234	0.0	GO:0006096	85	62.003	42
ARC_ART_c46849	106	XP_008438190	0.0	GO:0044699	71	43.5134	35
ARC_ART_c46888	138	KIZ07913	0.1	-	55	35.8094	45
ARC_ART_c46901	131	XP_002958508	0.0	GO:0044238	72	49.6766	37
ARC_ART_c46927	111	XP_002946547	0.0	GO:0003917	91	56.6102	34
ARC_ART_c46948	122	XP_005643526	0.0	GO:0016491	66	45.4394	39
ARC_ART_c46966	163	XP_001776225	0.0	-	71	50.8322	42
ARC_ART_c46980	179	XP_011016510	0.0	GO:0044444	100	93.2041	45
ARC_ART_c47032	124	XP_002534749	0.0	-	68	42.743	41
ARC_ART_c47091	163	XP_011085947	0.0	GO:0005488	66	52.373	54
ARC_ART_c47116	146	XP_011096877	0.3	-	56	33.8834	44
ARC_ART_c47131	110	XP_002535428	0.0	GO:1902358	91	60.8474	36
ARC_ART_c47133	104	XP_001691491	0.0	GO:0006413	85	48.521	34
ARC_ART_c47149	124	XP_001416756	0.0	GO:0044272	75	50.0618	40
ARC_ART_c47189	115	YP_004376486	0.0	GO:0005840	74	48.9062	35
ARC_ART_c47241	141	XP_004957843	1.0	-	57	32.3426	35
ARC_ART_c47313	106	XP_009596630	0.3	-	61	33.4982	34
ARC_ART_c47364	133	XP_002535042	0.0	GO:0016787	78	58.9214	41
ARC_ART_c47384	120	KIZ00996	0.2	-	63	34.2686	41
ARC_ART_c47428	275	EPS63247	0.5	-	47	34.2686	72

ARC_ART_c47433	105	XP_001697013	0.0	GO:0006476	79	44.669	34
ARC_ART_c47440	116	XP_002536300	0.0	-	78	55.0694	38
ARC_ART_c47444	124	XP_005846855	0.0	GO:0008152	65	46.2098	41
ARC_ART_c47502	133	KIZ07461	0.2	-	51	34.6538	41
ARC_ART_c47531	128	XP_010911733	0.0	-	69	42.3578	36
ARC_ART_c47573	124	XP_002947116	0.0	GO:0043168	70	51.6026	41
ARC_ART_c47653	110	ACU17171	0.0	GO:0005507	83	51.2174	36
ARC_ART_c47748	141	XP_002536284	0.3	-	53	33.8834	39
ARC_ART_c47753	128	Q9G4F5	0.0	GO:0071704	76	51.2174	39
ARC_ART_c47764	139	XP_002982998	0.7	-	65	32.7278	38
ARC_ART_c47857	118	ERN20200	0.0	GO:0008236	74	51.6026	39
ARC_ART_c47929	131	BAJ89001	0.0	GO:0005874	91	65.0846	34
ARC_ART_c47930	125	XP_010499957	0.7	-	59	32.7278	37
ARC_ART_c47934	105	KIZ00952	0.0	-	85	50.0618	35
ARC_ART_c47953	115	XP_001769120	0.0	GO:0005774	82	54.6842	35
ARC_ART_c48064	116	NP_001043258	0.0	-	58	36.5798	39
ARC_ART_c48068	108	XP_005847889	0.4	-	57	33.4982	38
ARC_ART_c48074	105	EYU27590	0.0	GO:0051287	88	56.6102	34
ARC_ART_c48097	134	CBI17160	0.0	-	51	36.1946	39
ARC_ART_c48107	111	XP_001786470	0.2	-	62	34.6538	35
ARC_ART_c48235	111	KDD72600	0.0	-	62	37.7354	35
ARC_ART_c48292	151	XP_002499427	0.0	-	67	55.8398	49
ARC_ART_c48294	144	AEA11192	0.0	GO:0016020	66	43.8986	45
ARC_ART_c48304	127	XP_002538097	0.0	GO:0006306	75	48.521	37
ARC_ART_c48305	116	EMT13756	0.0	-	64	38.891	34
ARC_ART_c48312	113	KFM26819	0.0	-	67	38.891	34
ARC_ART_c48400	135	XP_007222341	0.0	GO:0046872	79	59.3066	43
ARC_ART_c48409	121	XP_005643963	0.9	-	61	31.9574	39
ARC_ART_c48460	110	XP_002536208	0.0	GO:0015116	91	61.2326	36

ARC_ART_c48467	102	XP_009350076	0.0	GO:0005525	100	69.3218	34
ARC_ART_c48488	136	CCO17481	0.1	-	66	35.4242	45
ARC_ART_c48519	146	XP_002459109	0.1	-	60	35.8094	45
ARC_ART_c48559	175	KEH16990	0.7	-	53	32.3426	43
ARC_ART_c48583	101	XP_002539020	0.0	-	63	36.5798	38
ARC_ART_c48631	173	XP_003056817	0.0	GO:0006629	88	64.3142	36
ARC_ART_c48667	116	EMT09683	0.0	GO:0008233	67	40.4318	34
ARC_ART_c48710	119	XP_006443427	0.0	-	64	37.3502	34
ARC_ART_c48728	117	XP_001768209	0.0	GO:0016765	74	44.2838	39
ARC_ART_c48756	123	XP_003060297	0.0	GO:0016021	87	59.3066	41
ARC_ART_c48758	130	XP_002538157	0.0	-	69	42.3578	42
ARC_ART_c48833	146	EEC77111	0.0	GO:0006935	81	50.8322	38
ARC_ART_c48876	119	KJB22799	0.7	-	52	32.7278	38
ARC_ART_c48917	133	XP_004956378	0.1	-	67	35.4242	40
ARC_ART_c48924	146	XP_002961230	0.0	-	54	37.3502	44
ARC_ART_c48984	119	CCO18841	0.0	GO:0016208	76	60.077	39
ARC_ART_c48989	114	XP_002539395	0.0	GO:0008152	77	50.447	35
ARC_ART_c48995	117	XP_005650838	0.0	GO:0009536	71	50.0618	39
ARC_ART_c49000	128	CDY67984	0.0	GO:0005802	71	47.3654	39
ARC_ART_c49001	113	YP_008802575	0.0	GO:0046872	94	69.707	37
ARC_ART_c49028	122	EAY87977	0.3	-	58	33.4982	36
ARC_ART_c49160	164	XP_008646667	0.1	-	45	34.6538	44
ARC_ART_c49191	120	EEC68395	0.0	GO:0003824	75	52.373	37
ARC_ART_c49205	121	CCO19335	0.0	GO:0003676	77	50.0618	40

ARC_ART_c49222	143	KIY92252	0.0	-	78	60.8474	47
ARC_ART_c49270	106	XP_002536731	0.1	-	64	34.2686	34
ARC_ART_c49272	128	XP_002537860	0.2	-	61	34.2686	34
ARC_ART_c49343	117	KDO39819	0.0	-	72	40.817	37
ARC_ART_c49352	147	XP_002539941	0.0	GO:0005975	95	69.707	43
ARC_ART_c49468	116	KJB66376	0.0	GO:0044763	70	43.8986	37
ARC_ART_c49471	145	KEH15495	0.0	-	71	58.151	46
ARC_ART_c49618	135	XP_004954325	0.0	-	68	36.5798	35
ARC_ART_c49628	166	XP_002955818	0.0	-	68	53.5286	47
ARC_ART_c49637	112	XP_007052326	0.0	GO:0006426	80	57.7658	36
ARC_ART_c49640	225	XP_006595860	0.7	-	50	33.8834	62
ARC_ART_c49661	102	XP_002536463	0.0	-	67	37.7354	34
ARC_ART_c49677	165	XP_009607448	0.2	-	52	34.6538	50
ARC_ART_c49744	109	KFM26016	0.0	-	80	39.2762	35
ARC_ART_c49764	172	AGA61781	0.1	-	50	33.8834	48
ARC_ART_c49837	118	XP_002536262	0.0	GO:0015698	80	50.447	36
ARC_ART_c49901	143	KGN59552	0.0	GO:0005739	78	67.781	46
ARC_ART_c49915	128	XP_002535972	0.0	-	67	42.3578	40
ARC_ART_c49920	105	XP_002536127	0.6	-	54	30.4166	35
ARC_ART_c49948	134	XP_002537860	0.0	GO:0050794	74	50.447	43
ARC_ART_c50004	193	XP_002539100	0.0	-	58	38.1206	41
ARC_ART_c50013	120	EEC76877	0.0	-	66	39.6614	39
ARC_ART_c50015	103	KEH38742	0.0	GO:0044710	76	41.2022	34
ARC_ART_c50017	151	XP_007227062	0.3	-	50	32.7278	44
ARC_ART_c50071	126	XP_001757824	0.0	-	53	36.5798	41
ARC_ART_c50077	182	XP_003054806	0.5	-	51	33.8834	54
ARC_ART_c50108	180	XP_009797389	0.0	-	73	42.743	34

ARC_ART_c50221	167	KJB09183	0.0	GO:0009086	87	100.523	54
ARC_ART_c50269	119	XP_006664478	0.0	GO:0044710	74	45.8246	39
ARC_ART_c50289	118	XP_001752711	0.0	-	63	38.1206	38
ARC_ART_c50293	108	XP_010261131	0.4	-	54	33.113	35
ARC_ART_c50318	169	XP_009350059	0.0	GO:0044765	70	58.9214	47
ARC_ART_c50349	103	XP_002536180	0.1	-	55	35.4242	38
ARC_ART_c50354	112	XP_002535351	0.5	-	62	32.7278	35
ARC_ART_c50363	160	EXB22640	0.7	-	47	33.113	46
ARC_ART_c50381	265	KJB38963	0.0	-	73	65.855	46
ARC_ART_c50387	104	XP_008777315	0.0	GO:0006200	82	56.9954	34
ARC_ART_c50443	124	XP_010929236	0.4	-	57	33.4982	35
ARC_ART_c50486	107	XP_002534939	0.0	GO:0005524	79	45.0542	34
ARC_ART_c50554	139	XP_003576285	0.0	GO:0004643	73	48.9062	42
ARC_ART_c50640	114	XP_002534793	0.0	-	68	40.4318	35
ARC_ART_c50709	105	EMT24028	0.1	-	64	33.113	34
ARC_ART_c50722	152	AAV65368	0.0	GO:1901564	63	47.7506	44
ARC_ART_c50741	127	ACN27081	0.0	GO:0044763	74	55.4546	39
ARC_ART_c50749	127	KJB66751	0.9	-	51	31.9574	45
ARC_ART_c50771	117	BAO23825	0.0	-	61	36.5798	34
ARC_ART_c50772	159	XP_002539002	0.0	-	58	46.2098	56
ARC_ART_c50793	154	XP_010482994	0.6	-	54	33.113	42
ARC_ART_c50842	134	XP_010036840	0.0	-	66	38.1206	39
ARC_ART_c50848	103	XP_010911770	0.0	-	67	40.0466	34
ARC_ART_c50850	125	XP_002538681	0.0	-	71	36.965	39
ARC_ART_c50864	103	CAJ86191	0.0	GO:0016747	73	45.4394	34

ARC_ART_c50890	170	EAY88134	0.0	GO:0036054	83	102.834	56
ARC_ART_c50919	111	YP_001019064	0.3	-	65	33.113	35
ARC_ART_c50977	166	XP_003056058	0.6	-	60	33.113	45
ARC_ART_c50978	119	XP_003635977	0.0	GO:0008152	73	43.8986	34
ARC_ART_c50980	144	CCO17341	0.5	-	52	33.4982	46
ARC_ART_c50983	138	XP_002535505	0.0	-	62	36.965	45
ARC_ART_c50990	159	XP_009350072	0.0	GO:0003723	87	75.8702	47
ARC_ART_c51044	211	KDO81869	0.4	-	51	34.2686	47
ARC_ART_c51049	133	XP_002540194	0.0	GO:0009414	75	56.225	44
ARC_ART_c51078	107	XP_001417416	0.2	-	61	34.2686	34
ARC_ART_c51083	219	XP_009351044	0.0	-	73	46.595	34
ARC_ART_c51085	108	CEF98227	0.0	-	62	36.5798	37
ARC_ART_c51131	135	XP_009351102	0.0	GO:0048027	97	83.1889	40
ARC_ART_c51147	106	XP_002536785	0.0	-	85	52.7582	35
ARC_ART_c51149	183	CEF99950	0.0	GO:0016740	61	51.6026	60
ARC_ART_c51165	169	XP_005645144	0.1	-	49	36.5798	59
ARC_ART_c51201	138	XP_002871073	0.8	-	45	31.5722	46
ARC_ART_c51202	140	EYU39073	1.0	-	58	32.3426	39
ARC_ART_c51212	129	XP_001691491	0.0	GO:0006413	76	54.299	42
ARC_ART_c51282	146	EDQ48693	0.0	GO:0044763	72	47.7506	36
ARC_ART_c51293	144	KDD74569	0.3	-	62	33.4982	40
ARC_ART_c51319	109	KIZ07365	0.0	-	67	40.0466	34
ARC_ART_c51324	145	XP_001786986	0.0	GO:0005737	82	72.4034	45
ARC_ART_c51331	118	XP_006849895	0.5	-	58	32.3426	36
ARC_ART_c51337	139	CEF98353	0.0	GO:0044763	66	45.0542	45
ARC_ART_c51379	102	XP_002539275	0.0	-	70	37.3502	34

ARC_ART_c51398	122	XP_002539918	0.0	GO:0031514	90	77.7962	40
ARC_ART_c51423	153	XP_008230397	0.0	-	62	36.965	35
ARC_ART_c51424	103	BAD30585	0.8	-	64	32.3426	34
ARC_ART_c51436	128	EEC69922	0.0	GO:0009536	100	72.4034	36
ARC_ART_c51474	204	XP_011626846	0.0	GO:0003678	78	61.2326	50
ARC_ART_c51485	144	XP_002507415	0.0	-	82	51.6026	39
ARC_ART_c51500	215	AGZ19352	0.1	-	54	33.8834	48
ARC_ART_c51516	104	CCO20061	0.0	GO:0046872	70	45.4394	34
ARC_ART_c51535	120	YP_008802566	0.0	-	69	42.3578	39
ARC_ART_c51540	123	EPS59532	0.1	-	65	35.4242	35
ARC_ART_c51550	108	XP_009351045	0.0	GO:0016491	83	55.8398	36
ARC_ART_c51562	114	XP_002536933	0.0	-	86	39.6614	37
ARC_ART_c51580	134	XP_011089625	0.4	-	54	33.113	35
ARC_ART_c51603	111	BAC84394	0.0	-	55	36.1946	36
ARC_ART_c51622	127	XP_002540078	0.0	-	62	35.8094	37
ARC_ART_c51632	113	XP_004498478	0.0	-	54	36.1946	35
ARC_ART_c51635	120	KJB08094	0.7	-	53	32.7278	39
ARC_ART_c51652	162	XP_001694415	0.0	GO:1901363	71	49.2914	38
ARC_ART_c51677	123	EYU35441	0.0	-	61	40.817	36
ARC_ART_c51682	177	XP_002536066	0.0	-	63	44.669	58
ARC_ART_c51699	159	XP_002537786	0.0	-	65	43.5134	40
ARC_ART_c51709	110	XP_005647813	0.0	-	63	40.4318	36
ARC_ART_c51717	120	XP_002535661	0.1	-	58	34.6538	39
ARC_ART_c51724	140	EHK62698	0.0	-	55	37.3502	43
ARC_ART_c51734	162	KDP42953	0.5	-	68	33.8834	38

ARC_ART_c51748	203	CDY19671	0.0	-	57	42.743	47
ARC_ART_c51751	131	XP_002538312	0.0	GO:0008152	77	52.7582	36
ARC_ART_c51758	133	CDX74058	0.4	-	57	33.8834	35
ARC_ART_c51772	129	XP_002281484	0.0	GO:0004834	90	64.6994	40
ARC_ART_c51827	145	XP_001690081	0.0	GO:0003824	70	42.3578	47
ARC_ART_c51847	120	XP_002535024	0.0	GO:0030288	86	66.2402	37
ARC_ART_c51862	105	KDO47904	0.0	-	61	36.965	34
ARC_ART_c51872	105	EPS73483	0.0	GO:0003735	94	62.3882	34
ARC_ART_c51876	238	EXB36957	0.0	-	62	46.2098	50
ARC_ART_c51897	227	EYU40422	0.7	-	53	33.4982	52
ARC_ART_c51906	105	XP_002534939	0.0	GO:0005524	91	59.3066	34
ARC_ART_c51919	121	XP_002536073	0.0	GO:0008152	71	50.447	38
ARC_ART_c51932	182	XP_003616487	0.5	-	44	33.4982	59
ARC_ART_c51946	133	XP_002537713	0.0	-	63	38.1206	44
ARC_ART_c51949	149	XP_001774821	0.0	GO:0030976	79	67.0106	49
ARC_ART_c51955	107	EEE67873	0.0	GO:0016740	100	74.3294	35
ARC_ART_c51979	143	XP_010911656	0.0	-	61	39.6614	47
ARC_ART_c52020	116	XP_002536064	0.0	-	74	41.5874	35
ARC_ART_c52035	132	XP_008459583	0.0	-	61	37.3502	44
ARC_ART_c52036	133	EEC68421	0.0	GO:0006810	76	51.6026	43
ARC_ART_c52062	107	XP_002535947	0.6	-	56	32.7278	37
ARC_ART_c52066	114	XP_005843440	0.1	-	69	35.039	36
ARC_ART_c52109	127	ACR34295	0.3	-	61	33.8834	39
ARC_ART_c52122	120	XP_005849672	0.6	-	55	32.3426	36
ARC_ART_c52136	132	XP_005648272	0.0	GO:0030604	90	70.4774	42
ARC_ART_c52169	115	XP_008777617	0.0	GO:0006098	100	78.5666	38

ARC_ART_c52192	179	XP_002950332	0.0	GO:0006526	84	97.8265	59
ARC_ART_c52252	113	KEH15415	0.1	-	68	35.4242	35
ARC_ART_c52311	131	XP_002535254	0.0	GO:0008236	81	68.9366	43
ARC_ART_c52317	140	XP_002537457	0.0	-	51	38.5058	45
ARC_ART_c52354	163	XP_009350051	0.0	-	72	53.5286	44
ARC_ART_c52393	125	XP_001754299	0.6	-	54	33.113	35
ARC_ART_c52402	137	EEC78702	0.0	GO:0044699	72	43.5134	36
ARC_ART_c52418	112	KIY93810	0.0	GO:0006105	89	56.6102	37
ARC_ART_c52478	137	KDP22564	0.1	-	50	34.6538	42
ARC_ART_c52480	206	KJB69134	0.8	-	50	33.113	55
ARC_ART_c52509	161	XP_009350817	0.0	-	75	60.8474	48
ARC_ART_c52605	125	XP_009350067	0.0	-	77	42.743	36
ARC_ART_c52608	103	XP_002537864	0.0	-	76	45.4394	34
ARC_ART_c52630	183	XP_002534724	0.0	GO:0003824	56	46.2098	60
ARC_ART_c52664	154	XP_002536913	0.0	-	64	57.7658	51
ARC_ART_c52666	163	XP_009388289	0.0	-	67	45.8246	37
ARC_ART_c52679	118	XP_006373370	0.2	-	60	34.2686	41
ARC_ART_c52712	123	XP_002463051	0.4	-	46	33.4982	43
ARC_ART_c52713	155	YP_009106719	0.0	GO:0009536	72	50.447	44
ARC_ART_c52740	123	XP_001762925	0.0	GO:0003878	87	59.3066	41
ARC_ART_c52757	113	XP_002540296	0.0	-	78	53.1434	37
ARC_ART_c52783	138	XP_002958164	0.0	GO:0005975	93	85.5001	45
ARC_ART_c52796	139	XP_010438354	0.4	-	55	33.4982	43
ARC_ART_c52823	154	AAT46463	0.0	-	48	35.8094	45
ARC_ART_c52824	115	XP_005645362	0.0	GO:0090304	83	66.2402	37
ARC_ART_c52836	106	XP_004244905	0.4	-	63	32.7278	36
ARC_ART_c52855	135	EEE52318	0.0	GO:0009085	68	52.7582	45
ARC_ART_c52871	123	XP_002535867	0.0	GO:0006139	76	55.4546	39

ARC_ART_c52885	210	XP_001696348	0.7	-	49	33.4982	55
ARC_ART_c52900	142	XP_002538359	0.0	GO:0006259	71	53.1434	46
ARC_ART_c52915	120	XP_005846494	0.0	GO:0006879	82	62.3882	39
ARC_ART_c52960	159	CCO18618	0.3	-	52	33.8834	46
ARC_ART_c52965	239	XP_002505678	0.0	-	60	44.2838	55
ARC_ART_c52977	150	XP_002536744	0.3	-	63	33.8834	41
ARC_ART_c52983	163	XP_002539447	0.0	-	58	36.1946	55
ARC_ART_c52986	124	XP_002538474	0.0	-	76	55.4546	34
ARC_ART_c52988	172	EEC76122	0.0	GO:1901363	63	54.299	55
ARC_ART_c52995	113	XP_003063400	0.0	GO:0008137	83	63.929	36
ARC_ART_c53037	166	XP_002535911	0.0	-	53	39.6614	56
ARC_ART_c53048	163	BAO57289	0.0	GO:0070011	66	48.521	53
ARC_ART_c53090	121	XP_006660410	0.0	-	66	41.5874	36
ARC_ART_c53101	120	XP_002488912	0.3	-	64	33.8834	34
ARC_ART_c53133	124	AGZ19407	0.0	GO:0045333	84	70.8626	39
ARC_ART_c53136	145	ABK25366	0.0	-	61	41.9726	54
ARC_ART_c53157	129	XP_002539711	0.0	-	62	38.1206	40
ARC_ART_c53179	120	XP_009350076	0.0	GO:0003746	90	65.4698	40
ARC_ART_c53185	116	AFB34216	0.0	GO:0016668	74	48.1358	35
ARC_ART_c53239	138	XP_002964529	0.1	-	67	33.4982	34
ARC_ART_c53240	134	XP_001702976	0.8	-	55	32.3426	34
ARC_ART_c53273	134	XP_004952577	0.0	-	67	39.6614	40
ARC_ART_c53318	122	XP_002534958	0.1	-	66	35.4242	36
ARC_ART_c53333	123	XP_002539090	0.0	GO:0004872	82	53.5286	39
ARC_ART_c53361	149	EXB93351	0.0	GO:0016885	78	45.0542	37

ARC_ART_c53369	252	XP_002538136	0.0	GO:0030170	85	92.8189	57
ARC_ART_c53421	139	XP_007019774	0.5	-	55	30.8018	36
ARC_ART_c53468	107	EEC80862	0.0	GO:0006184	85	48.1358	34
ARC_ART_c53500	128	XP_010929029	0.0	-	63	40.4318	36
ARC_ART_c53534	164	AGG09511	0.0	GO:0003899	87	77.411	47
ARC_ART_c53537	108	EMS35753	0.0	GO:0030170	80	50.0618	35
ARC_ART_c53545	178	XP_002539557	0.0	GO:0008152	82	63.929	39
ARC_ART_c53557	168	XP_002538260	0.3	-	58	34.2686	34
ARC_ART_c53568	122	XP_001691682	0.5	-	61	32.7278	36
ARC_ART_c53578	128	CDY09233	0.3	-	52	33.8834	42
ARC_ART_c53594	183	XP_006395916	0.1	-	55	35.8094	47
ARC_ART_c53599	140	NP_001146352	0.0	GO:0050660	90	78.1814	42
ARC_ART_c53600	139	XP_002536688	0.0	GO:0097159	71	46.9802	38
ARC_ART_c53650	143	EDQ48091	0.1	-	54	34.6538	46
ARC_ART_c53655	110	XP_002538756	0.0	GO:0008152	81	54.6842	38
ARC_ART_c53656	135	EEC70905	0.0	GO:0044699	69	48.521	43
ARC_ART_c53658	131	KIZ03895	0.0	-	73	44.669	34
ARC_ART_c53709	119	XP_002535850	0.0	-	67	38.5058	37
ARC_ART_c53717	114	XP_003055764	0.0	-	72	51.9878	37
ARC_ART_c53727	117	XP_006371445	0.9	-	52	30.4166	34
ARC_ART_c53736	136	AFK45193	0.6	-	54	31.9574	35
ARC_ART_c53758	171	XP_001417046	0.0	GO:1901564	72	70.0922	55

ARC_ART_c53841	176	XP_010314956	0.0	-	69	48.521	39
ARC_ART_c53852	127	EDQ48391	0.0	GO:0031365	83	56.9954	42
ARC_ART_c53877	118	KIY98556	0.0	GO:0004157	81	62.3882	38
ARC_ART_c53879	106	XP_002537699	0.0	-	68	39.2762	35
ARC_ART_c53892	161	XP_002534897	0.0	GO:0043168	100	108.612	53
ARC_ART_c53926	114	BAF79985	0.9	-	58	31.9574	36
ARC_ART_c53942	164	XP_011014867	0.0	-	57	41.2022	52
ARC_ART_c53951	134	XP_002537487	0.0	-	63	39.2762	36
ARC_ART_c53967	162	ACL52872	0.0	GO:0048653	80	75.8702	50
ARC_ART_c53979	121	XP_002538359	0.1	-	59	34.6538	37
ARC_ART_c53981	127	ABO20851	0.0	-	64	35.039	34
ARC_ART_c53983	114	XP_002537961	0.0	GO:0016020	76	54.299	38
ARC_ART_c53990	138	KIZ05017	0.0	-	57	36.1946	45
ARC_ART_c53996	175	XP_006279256	0.0	-	65	42.743	43
ARC_ART_c54015	167	XP_001700058	0.0	GO:0016491	69	65.0846	53
ARC_ART_c54098	120	KFM27617	0.0	GO:0009058	75	47.7506	37
ARC_ART_c54102	112	XP_006599456	0.7	-	57	32.7278	38
ARC_ART_c54105	137	XP_002535478	0.0	GO:0006259	68	44.669	45
ARC_ART_c54110	116	XP_002536262	0.0	GO:0006820	73	48.1358	38
ARC_ART_c54167	187	XP_003057743	0.0	-	50	40.817	63
ARC_ART_c54193	221	EXC03898	0.2	-	45	35.039	59
ARC_ART_c54208	150	EEC77749	0.8	-	57	32.3426	42
ARC_ART_c54232	127	XP_002535328	0.0	-	92	72.0182	42
ARC_ART_c54244	109	XP_003561683	0.0	-	70	37.3502	34
ARC_ART_c54256	135	EAY97777	0.0	GO:0071704	78	50.8322	42
ARC_ART_c54270	135	KDD74973	0.0	-	61	38.891	36
ARC_ART_c54271	127	EDQ48677	0.0	-	64	48.9062	42
ARC_ART_c54315	115	EHK62704	0.0	-	71	43.8986	38
ARC_ART_c54348	121	XP_002537161	0.0	-	61	41.9726	36
ARC_ART_c54361	123	EXC33820	0.0	-	71	37.3502	35

ARC_ART_c54526	144	EEC66960	0.0	GO:0071704	72	49.2914	37
ARC_ART_c54541	134	XP_005648545	0.0	GO:0006807	77	46.595	35
ARC_ART_c54580	170	XP_002960003	0.0	GO:0005739	77	57.7658	40
ARC_ART_c54649	125	XP_002947541	0.0	-	63	40.0466	36
ARC_ART_c54848	133	XP_008222157	0.8	-	61	31.9574	36
ARC_ART_c54898	140	YP_001019103	0.0	GO:0003899	85	63.5438	40
ARC_ART_c54980	106	KJB09183	0.0	GO:0009086	91	65.855	35
ARC_ART_c54989	114	CBI36950	0.0	GO:0006508	78	52.7582	37
ARC_ART_c55019	121	BAJ94909	0.0	GO:0006189	79	51.6026	39
ARC_ART_c55037	144	KDP35193	0.6	-	54	33.113	46
ARC_ART_c55081	126	XP_002534677	0.0	-	69	50.8322	42
ARC_ART_c55123	119	KEH15491	0.0	GO:0006096	84	57.3806	39
ARC_ART_c55159	135	KDP26397	0.1	-	62	35.039	37
ARC_ART_c55177	116	XP_002505070	0.0	GO:0000166	71	44.2838	35
ARC_ART_c55234	120	XP_009350810	0.0	-	73	51.2174	38
ARC_ART_c55253	149	KJB72032	0.4	-	50	33.4982	44
ARC_ART_c55282	114	ACN65414	0.0	GO:0006820	77	41.2022	40
ARC_ART_c55296	150	XP_002534946	0.0	GO:0006879	85	65.0846	41
ARC_ART_c55338	129	CCO14897	0.0	-	69	40.0466	42
ARC_ART_c55347	150	ADE76966	0.0	-	62	52.373	40
ARC_ART_c55361	103	XP_003058797	0.0	-	70	47.3654	34
ARC_ART_c55368	117	XP_002536962	0.0	-	70	37.7354	34
ARC_ART_c55370	103	XP_010423205	0.0	-	70	37.7354	34
ARC_ART_c55409	159	EEC80776	0.0	GO:0016829	76	62.003	43
ARC_ART_c55626	132	XP_006285712	0.0	GO:0005829	64	52.7582	37

ARC_ART_c55714	133	XP_002954798	0.4	-	65	33.4982	38
ARC_ART_c55749	154	XP_002537560	0.8	-	61	31.5722	34
ARC_ART_c55819	107	XP_002535213	0.6	-	70	32.7278	34
ARC_ART_c55833	183	EYU24190	0.2	-	51	32.7278	43
ARC_ART_c55999	154	AHG55342	0.0	GO:0012506	80	77.7962	51
ARC_ART_c56015	156	XP_005844251	0.0	GO:0044765	61	45.0542	54
ARC_ART_c56026	175	EXC01672	0.2	-	50	35.039	44
ARC_ART_c56203	115	CBI25151	0.3	-	64	33.4982	37
ARC_ART_c56266	162	EMS50767	0.1	-	67	35.4242	34
ARC_ART_c56298	122	XP_008345633	0.0	-	65	38.1206	40
ARC_ART_c56302	129	XP_006660914	0.4	-	55	32.3426	34
ARC_ART_c56364	125	AES73618	0.3	-	55	32.3426	34
ARC_ART_c56371	107	XP_010911665	0.0	-	85	54.299	34
ARC_ART_c56380	137	XP_002536769	0.5	-	57	33.113	42
ARC_ART_c56415	110	KDD72682	0.0	GO:0008661	94	67.3958	35
ARC_ART_c56435	121	EEC84778	0.0	GO:0016773	90	65.0846	40
ARC_ART_c56437	114	XP_002536219	0.0	GO:0016020	83	50.0618	37
ARC_ART_c56550	108	XP_005850016	0.5	-	62	31.9574	37
ARC_ART_c56647	127	CDY71190	0.1	-	60	35.039	38
ARC_ART_c56682	116	XP_002488904	0.1	-	83	35.4242	37
ARC_ART_c56683	188	XP_003616487	0.1	-	56	35.4242	60
ARC_ART_c56722	130	CCO15227	0.0	GO:0046872	72	41.2022	37
ARC_ART_c56747	172	XP_002951836	0.0	-	63	38.1206	41
ARC_ART_c56849	109	XP_010919276	0.0	-	66	43.5134	36
ARC_ART_c56907	111	ABS72034	0.0	-	67	38.5058	37
ARC_ART_c56990	111	ERN19561	0.1	-	66	32.7278	36

ARC_ART_c56995	125	XP_003635966	0.0	-	65	42.743	41
ARC_ART_c57067	115	XP_004253462	0.0	GO:0008270	86	56.6102	38
ARC_ART_c57118	124	ACS44642	0.7	-	57	31.9574	42
ARC_ART_c57119	131	XP_002536094	0.0	-	78	42.743	41
ARC_ART_c57225	145	KEH15727	0.0	-	58	38.891	34
ARC_ART_c57253	138	ACJ84593	0.0	GO:0006096	78	61.2326	46
ARC_ART_c57284	139	XP_007046958	0.0	GO:0005749	62	48.521	43
ARC_ART_c57329	110	CDP17828	0.0	-	58	40.0466	39
ARC_ART_c57346	120	KFK32295	0.1	-	55	36.1946	38
ARC_ART_c57347	107	XP_007135028	0.0	-	71	38.1206	35
ARC_ART_c57400	161	XP_002534939	0.1	-	63	35.039	46
ARC_ART_c57413	109	CDP06793	0.7	-	51	32.7278	35
ARC_ART_c57418	108	XP_009351101	0.0	GO:0016787	69	45.8246	36
ARC_ART_c57492	117	XP_003629926	0.1	-	68	34.6538	38
ARC_ART_c57527	131	KDO45761	0.0	GO:0003746	81	55.0694	37
ARC_ART_c57544	207	KEH36277	0.0	-	58	36.965	34
ARC_ART_c57586	116	XP_010485312	0.5	-	61	32.3426	36
ARC_ART_c57642	113	EEC76122	0.0	GO:0050660	100	73.9442	35
ARC_ART_c57649	140	XP_002538951	0.0	-	86	57.7658	37
ARC_ART_c57686	108	XP_005850908	1.0	-	61	32.3426	34
ARC_ART_c57788	150	XP_002540099	0.0	-	60	48.9062	46
ARC_ART_c57833	155	CEG01982	0.4	-	60	33.4982	35
ARC_ART_c57954	187	EEC70005	0.3	-	48	34.6538	49
ARC_ART_c57990	102	KIY93494	0.1	-	65	33.8834	35
ARC_ART_c58026	119	ACF49260	0.6	-	59	32.7278	37
ARC_ART_c58066	136	XP_005645111	0.0	GO:0008152	78	49.2914	41

ARC_ART_c58124	117	XP_001758846	0.0	GO:0004871	76	49.6766	34
ARC_ART_c58151	128	XP_002965254	0.1	-	73	35.8094	34
ARC_ART_c58278	145	XP_011070396	0.2	-	50	34.6538	46
ARC_ART_c58447	147	XP_002953827	0.6	-	54	33.4982	44
ARC_ART_c58514	114	AAG41903	0.0	GO:1902358	88	59.6918	36
ARC_ART_c58522	127	XP_002968438	0.0	GO:0044710	72	56.6102	37
ARC_ART_c58604	131	YP_001687146	0.1	-	50	35.039	42
ARC_ART_c58633	113	AAR15338	0.0	GO:0044699	77	48.521	36
ARC_ART_c58648	117	XP_010234921	0.0	GO:0005737	78	53.1434	37
ARC_ART_c58788	178	XP_009125283	0.0	GO:0000155	82	43.1282	34
ARC_ART_c58896	158	XP_009396657	0.9	-	57	32.7278	38
ARC_ART_c59072	131	BAD23657	0.4	-	52	32.3426	34
ARC_ART_c59139	120	XP_004951713	0.0	-	66	38.891	39
ARC_ART_c59142	136	XP_002453118	0.0	-	58	39.2762	41
ARC_ART_c59154	117	XP_002951224	0.0	-	65	37.7354	35
ARC_ART_c59158	124	CDP05292	0.5	-	57	33.4982	35
ARC_ART_c59234	108	XP_002536620	0.0	GO:0016020	77	44.2838	35
ARC_ART_c59264	154	AGZ19352	0.1	-	50	33.4982	51
ARC_ART_c59437	123	XP_002535814	0.0	-	77	56.225	36
ARC_ART_c59601	108	XP_002539909	0.0	-	70	37.3502	34
ARC_ART_c59605	108	KDO42427	0.0	GO:0009067	77	51.2174	35
ARC_ART_c59745	122	XP_002523655	0.0	GO:0046686	81	66.2402	38
ARC_ART_c59875	114	XP_004486142	0.0	GO:0005507	81	53.9138	37
ARC_ART_c59926	135	KIZ07892	0.0	GO:0003984	78	62.3882	46
ARC_ART_c60043	141	XP_003057205	0.0	GO:0044763	64	43.1282	42
ARC_ART_c60122	106	CCO19401	0.1	-	64	35.8094	34
ARC_ART_c60208	119	XP_002537217	0.0	GO:0016853	71	47.3654	38
ARC_ART_c60265	169	ERN11679	0.8	-	60	33.113	45

ARC_ART_c60267	110	XP_002537173	0.0	-	66	41.2022	36
ARC_ART_c60410	101	AAG51067	0.3	-	54	32.7278	35
ARC_ART_c60457	111	XP_002537670	0.0	-	67	42.743	34
ARC_ART_c60723	107	XP_004253340	0.0	-	88	63.1586	35
ARC_ART_c60725	137	XP_007033597	0.8	-	58	32.3426	36
ARC_ART_c60739	161	XP_005844362	0.2	-	56	33.4982	41
ARC_ART_c60838	106	XP_002538524	0.0	-	88	62.3882	35
ARC_ART_c60941	119	XP_002538900	0.0	-	70	39.6614	37
ARC_ART_c60966	112	KDO54066	0.0	-	67	34.6538	34
ARC_ART_c61013	139	XP_010436089	0.7	-	63	33.113	36
ARC_ART_c61059	151	XP_002537322	0.0	GO:0005488	71	59.3066	49
ARC_ART_c61072	106	XP_009351265	0.0	GO:0008237	74	55.0694	35
ARC_ART_c61104	127	XP_002503873	0.0	GO:0006457	79	53.9138	34
ARC_ART_c61149	106	XP_011007148	0.1	-	62	35.039	35
ARC_ART_c61379	150	EEE54984	0.0	GO:0005524	95	89.3521	49
ARC_ART_c61549	127	XP_002537601	0.0	-	92	83.1889	42
ARC_ART_c61644	101	XP_001416558	0.0	-	67	38.5058	37
ARC_ART_c61676	157	KIZ06379	0.0	GO:0019438	72	51.6026	43
ARC_ART_c61698	112	BAK01092	0.2	-	69	34.2686	36
ARC_ART_c61764	160	KEH15304	0.0	-	69	37.7354	39
ARC_ART_c61787	145	XP_010908123	0.0	GO:0005507	74	43.1282	35
ARC_ART_c61910	150	XP_006409150	0.0	GO:0005743	68	49.2914	50
ARC_ART_c61938	108	ABA54870	0.0	GO:0016829	77	48.521	36
ARC_ART_c61957	118	XP_011015254	0.0	GO:0009086	65	48.521	38
ARC_ART_c61985	117	XP_004296344	0.7	-	66	32.7278	36
ARC_ART_c62016	141	XP_006649179	0.2	-	58	34.2686	39
ARC_ART_c62107	104	XP_006361197	0.0	-	71	39.6614	35
ARC_ART_c62136	134	EEE68526	0.0	-	88	69.3218	42

ARC_ART_c62161	136	XP_002951357	0.0	GO:0003824	72	48.521	37
ARC_ART_c62185	167	AGC78943	0.0	-	69	42.3578	39
ARC_ART_c62308	129	XP_002537063	0.0	GO:0016020	75	42.3578	40
ARC_ART_c62342	132	XP_002535829	0.3	-	51	33.8834	43
ARC_ART_c62422	174	XP_002994587	0.9	-	63	30.8018	38
ARC_ART_c62430	172	XP_001689512	0.0	GO:0001510	84	64.3142	39
ARC_ART_c62432	154	XP_007135028	0.0	-	64	40.4318	45
ARC_ART_c62500	123	XP_001422695	0.3	-	52	33.8834	42
ARC_ART_c62569	110	XP_002538155	0.0	GO:0050896	75	47.7506	36
ARC_ART_c62645	127	EEC77966	0.0	GO:0005524	85	65.0846	42
ARC_ART_c62745	131	XP_002537752	0.0	GO:0016614	72	50.0618	36
ARC_ART_c62755	104	XP_001786556	0.0	GO:0004129	85	50.447	34
ARC_ART_c62776	111	XP_002537391	0.0	-	61	35.039	34
ARC_ART_c63019	104	CEF97174	0.0	-	68	38.1206	35
ARC_ART_c63027	114	CAC12818	0.0	GO:0003746	89	67.3958	38
ARC_ART_c63035	110	AAV59467	0.0	GO:0016021	86	51.2174	36
ARC_ART_c63113	122	KIY98601	0.0	GO:0008446	92	73.9442	38
ARC_ART_c63119	111	KFM26588	0.8	-	61	31.5722	34
ARC_ART_c63204	118	XP_010233157	0.0	GO:0048731	74	48.1358	39
ARC_ART_c63369	194	XP_001775857	0.3	-	44	33.113	61
ARC_ART_c63462	159	XP_002539603	0.5	-	61	32.7278	36
ARC_ART_c63525	164	XP_005842570	0.4	-	63	32.7278	36
ARC_ART_c63544	105	XP_002539628	1.0	-	62	31.5722	35
ARC_ART_c63569	124	P17341	0.8	-	61	30.8018	34
ARC_ART_c63572	153	EPS74490	0.0	-	64	41.2022	37
ARC_ART_c63645	118	XP_002536202	0.1	-	70	33.8834	37
ARC_ART_c63792	115	XP_002540380	0.0	GO:0004871	72	46.9802	37
ARC_ART_c63941	127	XP_002508505	0.0	GO:0005975	71	44.2838	39
ARC_ART_c64012	125	XP_002535674	0.0	-	70	38.5058	41

ARC_ART_c64020	103	XP_001751276	0.0	GO:0016787	67	42.743	34
ARC_ART_c64157	121	XP_009408714	0.3	-	60	33.8834	35
ARC_ART_c64181	138	ERN19620	0.1	-	60	33.113	41
ARC_ART_c64267	126	KJB44121	0.0	GO:0005488	69	45.0542	42
ARC_ART_c64304	115	KDD75725	0.0	GO:0003824	73	48.521	34
ARC_ART_c64310	106	XP_003590707	0.9	-	60	32.3426	35
ARC_ART_c64355	112	EEC75070	0.0	-	76	46.9802	34
ARC_ART_c64397	133	AFW69095	0.7	-	50	30.4166	38
ARC_ART_c64399	111	YP_009094939	0.0	-	66	39.6614	36
ARC_ART_c64416	120	XP_004309846	0.0	GO:0016021	80	56.9954	40
ARC_ART_c64421	157	XP_010925667	1.0	-	64	32.3426	37
ARC_ART_c64422	115	AAM43911	0.0	GO:0015696	75	43.5134	36
ARC_ART_c64441	128	XP_002974782	0.2	-	48	34.2686	37
ARC_ART_c64582	111	XP_002536284	0.1	-	70	35.4242	37
ARC_ART_c64624	123	XP_002535829	0.5	-	65	33.113	35
ARC_ART_c64678	145	XP_010272941	0.3	-	54	32.3426	46
ARC_ART_c64733	112	CDI27994	0.0	GO:0003735	94	57.7658	34
ARC_ART_c64791	110	EEE52319	0.0	GO:0016779	76	50.447	34
ARC_ART_c64815	134	XP_009350817	0.0	-	78	52.373	41
ARC_ART_c64833	107	XP_003636121	0.9	-	57	29.6462	35
ARC_ART_c64841	111	KEH15468	0.0	-	75	47.7506	36
ARC_ART_c64888	113	XP_010257721	0.0	GO:0046872	75	50.0618	37
ARC_ART_c64895	159	NP_001105732	0.0	-	61	43.1282	49
ARC_ART_c64932	113	NP_178634	0.0	GO:0005774	86	56.9954	36
ARC_ART_c64954	142	EPS67479	0.7	-	57	30.8018	35
ARC_ART_c64982	134	ABG22504	0.4	-	63	33.4982	41
ARC_ART_c65013	137	KDP40193	0.9	-	48	32.3426	41
ARC_ART_c65021	233	KJB76688	0.0	-	52	36.5798	40
ARC_ART_c65040	118	KIZ05650	0.0	-	55	36.5798	36
ARC_ART_c65045	122	KJB33798	0.0	GO:0000166	72	45.4394	37

ARC_ART_c65199	113	XP_001692105	0.5	-	58	32.7278	36
ARC_ART_c65200	145	XP_002537310	0.0	-	63	47.7506	47
ARC_ART_c65262	150	XP_002878329	0.6	-	54	33.113	37
ARC_ART_c65270	120	AFK38069	0.0	GO:0008152	71	41.9726	39
ARC_ART_c65508	111	XP_003054871	0.0	GO:0005840	72	44.2838	37
ARC_ART_c65556	109	XP_002539837	0.0	-	91	55.8398	34
ARC_ART_c65563	101	BAM34416	0.0	-	62	35.039	35
ARC_ART_c65587	175	AGG09511	0.0	GO:0003899	90	89.3521	52
ARC_ART_c65600	105	XP_002306559	0.0	-	57	38.5058	35
ARC_ART_c65997	111	KDO61272	0.3	-	57	33.4982	35
ARC_ART_c66015	104	XP_007052446	0.6	-	63	32.7278	36
ARC_ART_c66038	140	XP_002535664	0.0	-	59	42.3578	37
ARC_ART_c66059	110	XP_010513146	0.5	-	57	33.113	35
ARC_ART_c66109	136	XP_002538959	0.0	-	78	53.1434	37
ARC_ART_c66241	161	XP_008226616	0.0	GO:0016829	72	55.8398	54
ARC_ART_c66294	107	AAT72502	0.0	GO:0043169	71	46.595	35
ARC_ART_c66460	133	XP_005848479	0.1	-	61	35.039	34
ARC_ART_c66471	114	KCW67770	0.8	-	52	32.3426	34
ARC_ART_c66551	165	XP_003588337	0.0	-	59	50.8322	54
ARC_ART_c66581	134	EEC78702	0.0	GO:0006200	88	73.9442	44
ARC_ART_c66649	126	CCO17124	0.3	-	55	33.4982	40
ARC_ART_c66659	138	XP_002500781	0.0	-	62	37.3502	37
ARC_ART_c66669	110	XP_002971427	0.0	GO:0034660	77	45.0542	36
ARC_ART_c66760	193	KIZ05678	0.7	-	59	33.4982	49
ARC_ART_c66781	133	XP_001415736	0.0	GO:0008976	81	60.8474	37

ARC_ART_c66853	131	EEC70905	0.0	GO:0003857	80	49.6766	36
ARC_ART_c66945	127	XP_002536223	0.0	GO:0005215	88	65.4698	36
ARC_ART_c66946	115	EEE51040	0.0	GO:0006259	70	48.9062	34
ARC_ART_c67001	105	XP_002538758	0.0	-	61	36.5798	34
ARC_ART_c67055	140	EEC84777	0.0	GO:0005739	86	65.4698	38
ARC_ART_c67101	162	CDP08364	0.0	-	59	39.2762	49
ARC_ART_c67152	126	XP_010480948	0.0	GO:0006783	80	60.4622	41
ARC_ART_c67167	123	XP_002537860	0.0	GO:0005488	68	42.3578	41
ARC_ART_c67248	125	XP_004966143	0.1	-	65	35.8094	40
ARC_ART_c67292	102	XP_011016741	0.0	-	100	75.485	34
ARC_ART_c67338	124	BAB62078	0.0	-	67	38.1206	34
ARC_ART_c67404	302	EPS74531	0.0	-	58	36.965	48
ARC_ART_c67416	125	KEH39540	0.0	GO:0017111	78	55.0694	41
ARC_ART_c67444	125	YP_009105429	0.0	-	72	45.8246	37
ARC_ART_c67527	154	XP_002953279	0.9	-	61	32.7278	52
ARC_ART_c67657	154	YP_635713	0.0	GO:0003899	84	61.2326	44
ARC_ART_c67715	146	XP_008803984	0.0	GO:0008152	76	48.521	34
ARC_ART_c67825	164	CBI27210	0.0	GO:0098655	71	48.1358	42
ARC_ART_c67871	118	XP_009596837	0.7	-	58	32.7278	36
ARC_ART_c67879	147	XP_005845617	0.0	GO:0046872	86	55.0694	37
ARC_ART_c67881	156	XP_009388289	0.0	-	70	50.8322	44
ARC_ART_c67892	109	XP_005850490	0.3	-	64	33.8834	34
ARC_ART_c67989	103	NP_050872	0.0	-	73	38.5058	34
ARC_ART_c68200	122	XP_008340187	0.0	GO:0006499	84	65.4698	38
ARC_ART_c68219	109	CDY00794	0.0	GO:0005829	83	60.077	36
ARC_ART_c68222	159	CEF96573	0.0	GO:0006096	79	68.9366	53
ARC_ART_c68259	128	XP_009757778	0.4	-	61	33.4982	36

ARC_ART_c68289	122	ADE77123	0.0	-	65	36.965	35
ARC_ART_c68350	113	XP_003060155	0.4	-	56	33.113	37
ARC_ART_c68407	116	XP_002536920	0.8	-	63	31.9574	38
ARC_ART_c68412	106	XP_002954798	0.0	GO:0006796	69	42.743	36
ARC_ART_c68415	125	ADP55084	0.0	-	73	36.5798	34
ARC_ART_c68499	134	XP_002535455	0.0	GO:0070011	71	48.9062	35
ARC_ART_c68560	199	CAH68005	0.1	-	53	35.8094	60
ARC_ART_c68640	134	CEF97223	0.0	GO:0005507	93	80.4925	44
ARC_ART_c68819	118	KJB83734	0.3	-	62	33.113	35
ARC_ART_c68842	151	XP_002535152	0.2	-	57	34.6538	40
ARC_ART_c68880	121	XP_001417437	0.0	GO:0009536	81	56.6102	37
ARC_ART_c68901	139	ABA96732	0.7	-	60	32.7278	35
ARC_ART_c68990	115	XP_003082043	0.0	-	72	38.891	36
ARC_ART_c68992	125	EPS74390	0.0	-	76	40.0466	34
ARC_ART_c68998	108	KFM23465	0.1	-	62	34.6538	35
ARC_ART_c69002	127	XP_002538567	0.1	-	65	33.8834	35
ARC_ART_c69014	152	XP_009391698	0.2	-	54	34.2686	51
ARC_ART_c69027	128	AIZ93905	0.0	GO:0006744	78	51.2174	37
ARC_ART_c69040	107	XP_003078193	0.0	-	60	40.4318	41
ARC_ART_c69098	142	XP_002509065	0.0	-	66	46.9802	42
ARC_ART_c69102	105	NP_001064083	0.6	-	55	32.7278	34
ARC_ART_c69149	111	XP_001786560	0.0	GO:0006281	72	51.9878	37
ARC_ART_c69158	145	XP_003628949	0.1	-	54	35.4242	48
ARC_ART_c69201	117	XP_006584106	0.0	GO:0016747	79	51.2174	39
ARC_ART_c69270	162	CCO17351	0.0	-	75	60.8474	48
ARC_ART_c69297	127	XP_001786470	0.1	-	65	36.1946	38
ARC_ART_c69343	129	XP_002536043	0.0	-	60	42.743	45
ARC_ART_c69413	155	XP_002537608	0.4	-	57	33.8834	49
ARC_ART_c69436	195	XP_002509369	0.0	GO:0016020	73	62.003	45

ARC_ART_c69507	118	XP_009378383	0.6	-	54	31.5722	42
ARC_ART_c69541	122	XP_002507415	0.3	-	60	33.113	35
ARC_ART_c69611	123	XP_008238798	0.8	-	57	32.7278	38
ARC_ART_c69663	122	XP_010500137	0.0	-	66	40.817	39
ARC_ART_c69698	118	XP_001782927	0.0	GO:0016556	67	46.595	40
ARC_ART_c69821	158	XP_003056374	0.0	GO:0006099	82	63.1586	41
ARC_ART_c69844	149	XP_001786381	0.0	GO:0050660	89	81.6481	49
ARC_ART_c69885	153	XP_011016509	0.0	GO:0005840	78	60.4622	50
ARC_ART_c69916	124	ABR25604	0.0	GO:0008152	100	89.3521	41
ARC_ART_c69954	187	ABH09321	0.0	-	62	42.3578	50
ARC_ART_c69970	132	XP_002538475	0.0	GO:0009536	74	62.003	43
ARC_ART_c70000	128	XP_002537492	0.0	GO:0016491	71	46.595	35
ARC_ART_c70017	147	XP_008234195	1.0	-	56	32.3426	44
ARC_ART_c70061	121	XP_004507635	0.5	-	56	33.113	37
ARC_ART_c70075	144	NP_001052420	0.8	-	56	32.7278	39
ARC_ART_c70100	122	XP_002960037	0.0	-	71	47.7506	39
ARC_ART_c70130	119	XP_003635977	0.0	-	60	45.4394	38
ARC_ART_c70171	113	XP_002538597	0.0	-	67	42.743	37
ARC_ART_c70173	134	XP_006659526	0.0	GO:0005759	79	49.2914	34
ARC_ART_c70191	128	EEE68527	0.0	GO:0009982	97	86.6557	42
ARC_ART_c70284	115	XP_002537525	0.0	-	67	40.0466	40
ARC_ART_c70350	117	XP_007021079	0.4	-	58	33.4982	39
ARC_ART_c70397	122	XP_005649527	0.0	-	75	36.965	40
ARC_ART_c70445	108	KEH15984	0.0	-	73	44.669	34
ARC_ART_c70455	121	XP_002318420	0.5	-	56	33.113	39
ARC_ART_c70458	144	XP_002537188	0.7	-	64	33.113	34
ARC_ART_c70478	130	XP_002959239	0.0	GO:0005840	73	49.2914	41

ARC_ART_c70494	176	XP_008788882	0.7	-	50	33.113	54
ARC_ART_c70615	106	EEE52318	0.0	GO:0022891	97	67.781	34
ARC_ART_c70660	120	ERN13324	0.0	GO:0006164	71	41.2022	38
ARC_ART_c70680	115	XP_008235315	0.3	-	72	33.4982	37
ARC_ART_c70730	112	YP_009106846	0.6	-	59	32.3426	42
ARC_ART_c70737	145	XP_003565918	0.1	-	71	35.8094	35
ARC_ART_c70869	138	XP_008393921	0.0	GO:0043231	61	41.9726	44
ARC_ART_c71103	149	KDP44554	0.0	GO:0051766	77	67.3958	49
ARC_ART_c71124	211	EAZ10012	0.0	-	61	45.4394	39
ARC_ART_c71139	107	XP_010053280	0.0	GO:0098655	88	58.5362	34
ARC_ART_c71184	124	XP_002977361	0.0	GO:0019752	77	57.7658	40
ARC_ART_c71229	113	XP_007147745	0.0	GO:0004553	72	51.2174	36
ARC_ART_c71351	105	KJB11747	0.3	-	64	33.4982	34
ARC_ART_c71428	108	KDP29794	0.0	GO:0008152	76	48.9062	34
ARC_ART_c71484	111	XP_002535067	0.0	-	74	45.0542	35
ARC_ART_c71603	129	XP_010065188	0.7	-	55	32.7278	34
ARC_ART_c71668	108	KEH15273	0.0	-	71	44.2838	35
ARC_ART_c71669	145	XP_011076983	0.6	-	63	33.113	38
ARC_ART_c71758	111	EEC68395	0.0	GO:0000959	91	67.0106	35
ARC_ART_c71808	123	XP_002514156	0.3	-	63	33.8834	36
ARC_ART_c71905	114	XP_002537747	0.0	-	73	38.1206	34
ARC_ART_c71935	145	XP_002539815	0.0	-	91	78.5666	46
ARC_ART_c72066	138	XP_002509656	0.9	-	56	32.7278	44
ARC_ART_c72147	141	EEC68421	0.0	GO:0055085	97	53.5286	44

ARC_ART_c72405	109	XP_010271971	0.0	-	58	40.0466	34
ARC_ART_c72579	197	XP_002522041	0.0	-	54	36.965	44
ARC_ART_c72598	119	EAY86798	0.0	GO:0044699	74	56.225	39
ARC_ART_c72678	143	XP_002535024	0.0	GO:0030288	79	63.929	43
ARC_ART_c72703	121	XP_009350812	0.0	-	81	58.9214	38
ARC_ART_c72746	113	ADG27885	0.2	-	64	31.9574	37
ARC_ART_c72749	129	EPS71592	0.4	-	55	33.4982	38
ARC_ART_c72961	107	XP_004512605	0.0	-	65	40.0466	35
ARC_ART_c72969	108	DAA56211	0.0	GO:0003723	97	76.2554	36
ARC_ART_c72991	165	XP_002540420	0.0	-	65	55.8398	49
ARC_ART_c72996	110	XP_006379655	0.0	GO:0016491	72	41.5874	36
ARC_ART_c73054	119	XP_002507709	0.0	GO:0008152	78	56.225	37
ARC_ART_c73060	143	XP_002539243	0.0	GO:0006364	82	67.781	41
ARC_ART_c73094	176	XP_002503039	0.4	-	57	33.8834	49
ARC_ART_c73107	113	XP_002536143	0.0	GO:0003840	86	56.225	37
ARC_ART_c73221	110	XP_005644833	0.0	-	77	47.7506	35
ARC_ART_c73271	121	XP_002317832	0.0	-	68	39.6614	35
ARC_ART_c73294	104	EAY92422	0.0	GO:0006412	76	43.8986	34
ARC_ART_c73343	120	EEC77966	0.0	-	62	41.9726	35
ARC_ART_c73381	106	KIZ07491	0.6	-	62	32.7278	35
ARC_ART_c73408	110	XP_002536180	0.0	GO:0009987	63	45.4394	36
ARC_ART_c73454	130	EEC76404	0.0	-	78	61.2326	42
ARC_ART_c73469	108	XP_009350076	0.0	-	67	41.9726	34
ARC_ART_c73554	105	BAJ94909	0.0	GO:0003824	74	55.8398	39
ARC_ART_c73715	144	XP_002537492	0.0	GO:0044710	73	55.4546	46
ARC_ART_c73734	127	XP_001418395	0.0	GO:0050660	73	53.9138	41
ARC_ART_c73744	146	CAH66433	0.0	GO:0004591	82	56.225	39
ARC_ART_c73812	108	ADE77143	0.2	-	55	31.9574	36

ARC_ART_c73870	117	XP_005649298	0.0	GO:0008237	83	61.6178	37
ARC_ART_c73877	136	KFM27067	0.4	-	57	33.4982	45
ARC_ART_c73957	133	XP_005642829	0.0	GO:0005488	78	54.299	38
ARC_ART_c73964	106	ABQ81943	0.0	GO:0005774	88	56.225	34
ARC_ART_c74001	117	XP_003572394	0.4	-	63	33.4982	36
ARC_ART_c74063	138	XP_002535370	0.0	-	64	37.3502	45
ARC_ART_c74076	111	EPS73332	0.0	-	69	42.743	36
ARC_ART_c74086	113	XP_003055563	0.0	-	64	39.6614	37
ARC_ART_c74090	124	XP_002535223	0.0	GO:0016226	92	77.411	41
ARC_ART_c74092	111	XP_002535812	0.0	-	68	40.4318	35
ARC_ART_c74166	111	NP_001267591	0.2	-	62	33.4982	37
ARC_ART_c74278	134	XP_010930702	0.0	GO:0031348	73	53.9138	41
ARC_ART_c74297	125	EYU40687	0.0	-	65	46.595	49
ARC_ART_c74553	149	XP_006597592	0.7	-	57	32.7278	35
ARC_ART_c74575	113	XP_006443716	0.1	-	59	34.6538	37
ARC_ART_c74646	139	CDY23627	0.0	GO:0044763	69	42.3578	43
ARC_ART_c74664	127	XP_002538998	0.0	GO:0003985	92	73.9442	42
ARC_ART_c74753	106	AGB85057	0.0	GO:0005622	67	40.4318	34
ARC_ART_c74773	111	XP_002535681	0.0	-	76	38.5058	34
ARC_ART_c74787	189	NP_001051007	0.2	-	68	35.039	35
ARC_ART_c74847	164	KFK42683	0.5	-	51	33.8834	43
ARC_ART_c74888	141	KDD76694	0.0	GO:0005975	73	47.7506	45
ARC_ART_c74986	133	AGZ13631	0.0	GO:0017111	77	51.6026	40
ARC_ART_c75015	140	XP_002536314	0.0	-	64	41.2022	39
ARC_ART_c75021	152	EMS56103	0.0	GO:0003724	73	48.521	41
ARC_ART_c75032	109	CDP10410	0.2	-	62	33.8834	35
ARC_ART_c75062	114	EEC81066	0.0	-	68	43.5134	38
ARC_ART_c75211	137	XP_002534778	0.0	-	64	40.817	34
ARC_ART_c75218	117	EEC81163	0.0	GO:0016020	72	41.2022	36

ARC_ART_c75226	131	AAL67577	0.0	GO:0003866	85	53.5286	34
ARC_ART_c75349	169	EEC70790	0.3	-	53	34.2686	52
ARC_ART_c75358	236	XP_003607358	0.1	-	58	36.1946	41
ARC_ART_c75374	116	BAB96757	0.0	GO:0055114	64	46.595	37
ARC_ART_c75444	124	XP_002536131	0.0	GO:0003723	80	55.0694	40
ARC_ART_c75721	109	XP_011623499	0.0	-	70	36.5798	37
ARC_ART_c75814	170	XP_002953659	0.0	GO:0071704	62	53.5286	48
ARC_ART_c75819	150	ADB85516	0.0	GO:0050896	67	38.5058	37
ARC_ART_c75834	114	AFW62201	0.0	-	68	37.3502	35
ARC_ART_c75863	113	XP_002536388	0.0	-	64	43.8986	37
ARC_ART_c75914	198	XP_009405296	0.2	-	48	35.4242	47
ARC_ART_c76009	130	EEC69922	0.0	-	62	41.5874	43
ARC_ART_c76027	131	KJB54328	1.0	-	47	31.9574	38
ARC_ART_c76046	151	KGN58106	0.0	-	59	35.4242	49
ARC_ART_c76169	148	XP_003610227	0.0	-	60	36.5798	35
ARC_ART_c76310	128	CDP17959	0.0	-	66	37.3502	36
ARC_ART_c76367	117	NP_001235636	0.4	-	68	32.3426	35
ARC_ART_c76371	149	KDP33479	0.2	-	50	35.039	46
ARC_ART_c76404	140	XP_011461326	0.7	-	54	32.7278	35
ARC_ART_c76418	114	KDO74596	0.0	GO:0000105	81	52.373	37
ARC_ART_c76457	114	XP_002966616	0.0	-	69	38.891	36
ARC_ART_c76485	137	EEC81134	0.0	-	68	36.1946	35
ARC_ART_c76516	154	EPS74345	0.0	-	67	36.1946	34
ARC_ART_c76517	120	XP_003610311	0.8	-	58	32.3426	43
ARC_ART_c76596	127	EEC70331	0.0	GO:0055085	85	64.6994	41
ARC_ART_c76724	118	CCO19045	0.0	GO:0009295	91	70.4774	36
ARC_ART_c76771	177	XP_002523118	0.4	-	55	33.4982	47

ARC_ART_c76798	111	XP_002461194	0.0	GO:0016491	70	45.4394	37
ARC_ART_c76799	138	XP_007029407	0.4	-	65	33.4982	35
ARC_ART_c76800	153	XP_007039355	0.7	-	64	33.113	34
ARC_ART_c76901	102	XP_001777249	1.0	-	67	31.9574	34
ARC_ART_c76985	147	CDX71556	0.8	-	56	30.4166	37
ARC_ART_c76995	125	XP_002505139	0.0	GO:0071704	68	41.9726	41
ARC_ART_c76998	140	XP_009372063	0.4	-	60	33.8834	40
ARC_ART_c77003	165	XP_007225011	0.2	-	56	33.4982	37
ARC_ART_c77267	180	XP_002946384	0.0	-	59	40.0466	42
ARC_ART_c77273	126	XP_003588326	0.1	-	54	35.039	46
ARC_ART_c77278	117	EDQ48498	0.3	-	52	33.4982	38
ARC_ART_c77322	148	XP_002504854	0.0	-	75	59.3066	44
ARC_ART_c77419	141	XP_010937387	0.8	-	67	32.7278	34
ARC_ART_c77442	128	XP_002503468	0.0	-	58	38.891	50
ARC_ART_c77447	107	XP_002540478	0.0	-	64	35.8094	34
ARC_ART_c77556	131	BAK05540	0.0	GO:0005829	81	59.6918	43
ARC_ART_c77611	170	EPS71129	0.7	-	57	31.9574	38
ARC_ART_c77612	270	EPS74531	0.0	-	61	41.5874	42
ARC_ART_c77641	123	CCO18891	0.8	-	56	32.7278	41
ARC_ART_c77659	125	BAJ97253	0.3	-	63	33.8834	36
ARC_ART_c77661	193	XP_002535812	0.1	-	57	36.1946	45
ARC_ART_c77769	131	XP_002538097	0.0	GO:0032775	79	70.8626	43
ARC_ART_c77778	125	AAW57408	0.2	-	58	34.2686	39
ARC_ART_c77793	119	XP_002535989	0.1	-	66	35.4242	36
ARC_ART_c77825	110	ERN08848	0.8	-	64	31.5722	34
ARC_ART_c77837	114	XP_002536984	0.0	-	62	43.1282	37
ARC_ART_c77847	139	XP_001786950	0.9	-	65	32.3426	35

ARC_ART_c77880	131	XP_009351102	0.0	-	75	58.151	40
ARC_ART_c77883	137	XP_005847301	0.0	GO:0005737	74	48.9062	43
ARC_ART_c77900	139	XP_003081465	0.0	-	64	37.3502	42
ARC_ART_c77903	134	XP_002443700	1.0	-	55	32.3426	40
ARC_ART_c77912	129	XP_010264147	0.8	-	52	32.7278	44
ARC_ART_c77953	131	EYU31171	0.2	-	63	34.2686	41
ARC_ART_c78065	123	XP_003541320	0.0	-	53	36.965	41
ARC_ART_c78074	114	XP_003054906	0.1	-	61	35.8094	34
ARC_ART_c78108	143	CDP04365	0.0	GO:0009805	78	73.1738	47
ARC_ART_c78251	129	KJB26551	1.0	-	63	32.3426	36
ARC_ART_c78343	152	XP_002535814	0.0	-	63	36.5798	41
ARC_ART_c78373	116	KDD75933	0.0	-	65	34.2686	38
ARC_ART_c78378	132	XP_002535223	0.0	GO:0005198	83	58.9214	36
ARC_ART_c78510	119	XP_007146093	0.1	-	66	33.113	36
ARC_ART_c78531	160	XP_003578773	0.0	-	56	40.0466	53
ARC_ART_c78548	115	XP_011016369	0.0	-	70	38.891	34
ARC_ART_c78552	118	XP_002508072	0.0	-	64	38.5058	42
ARC_ART_c78769	116	AAA85742	0.0	GO:0043167	76	53.9138	38
ARC_ART_c78829	144	XP_001696797	0.1	-	60	36.1946	41
ARC_ART_c78837	114	XP_009350065	0.0	GO:0016021	86	58.5362	37
ARC_ART_c78861	121	XP_001763917	0.0	-	64	40.0466	34
ARC_ART_c78879	163	EEC76879	0.0	GO:0044272	72	61.2326	51
ARC_ART_c78887	158	CDY10832	0.4	-	53	33.8834	47
ARC_ART_c78969	131	XP_001786985	0.0	GO:0055114	90	73.1738	44
ARC_ART_c79069	119	XP_002306269	0.0	-	60	38.5058	38
ARC_ART_c79073	125	EPS60084	0.5	-	54	33.113	35

ARC_ART_c79186	166	XP_008789244	0.2	-	55	34.6538	56
ARC_ART_c79187	138	ACU18664	0.2	-	62	34.2686	35
ARC_ART_c79189	103	XP_011016366	0.0	-	67	40.817	34
ARC_ART_c79369	131	XP_002503468	0.6	-	63	32.7278	44
ARC_ART_c79407	131	XP_001787043	0.4	-	68	33.113	35
ARC_ART_c79538	137	XP_003061108	0.4	-	53	32.3426	43
ARC_ART_c79554	125	XP_007047713	0.8	-	58	31.5722	34
ARC_ART_c79725	147	XP_003056137	0.0	GO:0006261	72	61.2326	47
ARC_ART_c79781	112	KDP35453	0.9	-	52	30.4166	36
ARC_ART_c79883	128	XP_007014649	0.8	-	58	32.7278	36
ARC_ART_c79980	113	XP_002536445	0.0	-	94	70.0922	36
ARC_ART_c79981	106	XP_002540394	0.2	-	65	33.113	35
ARC_ART_c80061	117	XP_002313868	0.0	GO:0007264	86	55.0694	36
ARC_ART_c80069	177	XP_001700799	0.0	-	66	36.5798	36
ARC_ART_c80106	109	XP_011458535	0.0	GO:0000166	76	43.8986	34
ARC_ART_c80345	107	XP_002949632	0.0	GO:0050660	79	53.9138	34
ARC_ART_c80438	112	EEC76877	0.0	GO:0046961	83	42.3578	36
ARC_ART_c80479	123	XP_002946381	0.0	-	65	39.2762	35
ARC_ART_c80484	103	CDY32498	0.0	GO:0006468	82	59.6918	34
ARC_ART_c80504	119	XP_002540263	0.1	-	66	34.6538	36
ARC_ART_c80547	107	KFM28820	0.0	GO:0006521	82	54.6842	35
ARC_ART_c80556	115	XP_002540393	0.2	-	55	33.8834	36
ARC_ART_c80622	114	XP_002539701	0.0	GO:0003824	70	43.1282	37
ARC_ART_c80653	102	XP_001752938	0.0	GO:0030976	85	62.3882	34
ARC_ART_c80736	144	ADK60808	0.2	-	52	32.3426	36
ARC_ART_c80764	110	XP_002966614	0.2	-	58	34.2686	36
ARC_ART_c80817	130	XP_008670284	0.6	-	58	32.7278	34
ARC_ART_c80855	131	XP_002979806	0.5	-	68	32.7278	35

ARC_ART_c80981	119	EDQ48504	0.0	-	74	53.9138	39
ARC_ART_c81040	162	XP_005843747	0.1	-	58	35.8094	43
ARC_ART_c81188	123	EAY93455	0.0	GO:0006810	100	69.707	34
ARC_ART_c81212	127	XP_002957700	0.0	GO:0008270	88	64.6994	36
ARC_ART_c81329	116	XP_005847015	0.1	-	67	35.4242	34
ARC_ART_c81371	153	XP_002503387	0.0	GO:0031425	94	70.0922	38
ARC_ART_c81383	155	XP_002503817	0.0	GO:0044763	65	43.8986	43
ARC_ART_c81391	132	KEH15576	0.0	-	63	38.891	38
ARC_ART_c81397	119	XP_002539786	0.0	-	70	45.0542	40
ARC_ART_c81516	138	XP_005842715	0.0	GO:0004152	74	48.1358	43
ARC_ART_c81527	118	XP_006837350	0.1	-	54	35.8094	37
ARC_ART_c81576	159	XP_002535020	0.0	GO:0016407	63	46.9802	46
ARC_ART_c81577	147	KFK44954	0.0	-	68	42.3578	41
ARC_ART_c81585	134	EEC68547	0.0	-	87	67.0106	40
ARC_ART_c81631	122	XP_001776739	0.0	-	64	37.3502	39
ARC_ART_c81633	154	XP_009363157	0.0	-	64	36.965	39
ARC_ART_c81673	106	XP_002536962	0.0	-	73	47.3654	34
ARC_ART_c81744	110	KIY91666	0.0	-	61	39.2762	34
ARC_ART_c81800	121	YP_009057862	0.1	-	65	35.039	40
ARC_ART_c81855	138	EEC76877	0.0	GO:0046961	81	58.5362	38
ARC_ART_c81890	102	XP_009102989	0.0	GO:0004871	72	46.2098	37
ARC_ART_c81920	125	XP_008232440	0.0	GO:0016620	67	45.8246	40
ARC_ART_c81929	129	CDP15395	0.5	-	61	33.113	39
ARC_ART_c81953	183	XP_002536422	0.0	-	61	47.3654	54
ARC_ART_c81986	119	XP_002536619	0.0	-	61	36.5798	34
ARC_ART_c82069	118	XP_002959326	0.0	-	62	36.1946	37
ARC_ART_c82081	157	XP_002535664	0.0	-	57	39.6614	54
ARC_ART_c82098	153	XP_010267751	0.5	-	56	33.4982	39

ARC_ART_c82104	146	XP_006294456	0.2	-	48	34.2686	54
ARC_ART_c82135	140	XP_008235765	0.5	-	55	33.4982	45
ARC_ART_c82137	110	XP_002946406	0.0	GO:0016491	65	43.8986	35
ARC_ART_c82172	139	EMS45960	0.0	-	74	39.2762	35
ARC_ART_c82229	115	XP_002538258	1.0	-	62	31.9574	35
ARC_ART_c82231	153	XP_002536283	0.9	-	53	31.9574	45
ARC_ART_c82336	191	EYU37814	0.8	-	66	32.7278	39
ARC_ART_c82555	174	XP_007213052	0.8	-	56	33.113	39
ARC_ART_c82568	134	XP_007033012	0.0	GO:0004832	81	64.6994	37
ARC_ART_c82572	165	XP_001754187	0.0	-	62	46.9802	54
ARC_ART_c82577	115	EDQ48461	0.0	GO:0005829	84	65.0846	38
ARC_ART_c82748	139	EAY87341	0.5	-	53	32.7278	43
ARC_ART_c82900	149	XP_002538285	0.0	-	63	37.3502	46
ARC_ART_c82969	114	XP_001786972	0.0	-	77	39.2762	35
ARC_ART_c83107	171	XP_002951005	0.5	-	53	33.113	56
ARC_ART_c83221	135	EXB81084	0.8	-	54	32.7278	35
ARC_ART_c83274	216	EPS70020	0.0	-	70	43.5134	34
ARC_ART_c83357	113	KJB14995	0.0	GO:0004611	82	46.2098	34
ARC_ART_c83519	126	XP_002505871	0.2	-	65	34.2686	38
ARC_ART_c83567	104	KIZ00420	0.4	-	62	33.113	35
ARC_ART_c83652	116	XP_005647728	0.0	GO:0008152	73	46.2098	34
ARC_ART_c83663	115	AFW78921	0.8	-	58	32.3426	39
ARC_ART_c83700	161	XP_002537471	0.5	-	56	33.4982	48
ARC_ART_c83727	125	XP_006661958	0.0	GO:1901575	73	48.1358	34
ARC_ART_c83735	141	XP_002536203	0.0	-	64	38.5058	34
ARC_ART_c83767	169	YP_009106678	0.0	GO:0019752	76	52.373	42
ARC_ART_c83852	113	XP_002539837	0.0	-	91	36.5798	34

ARC_ART_c83908	107	CCO15507	0.2	-	67	33.8834	34
ARC_ART_c83960	131	XP_002536262	0.0	GO:0044765	59	46.595	42
ARC_ART_c83993	167	XP_006844580	0.1	-	61	35.039	36
ARC_ART_c84161	134	KIZ00400	0.0	GO:0006520	80	59.6918	41
ARC_ART_c84199	137	XP_011016737	0.0	-	97	92.4337	45
ARC_ART_c84248	114	XP_003610994	0.8	-	60	30.4166	35
ARC_ART_c84311	244	CBW45787	0.8	-	59	33.4982	44
ARC_ART_c84321	105	ERN11910	0.1	-	60	33.8834	35
ARC_ART_c84325	137	EEE52322	0.0	GO:0016020	81	59.3066	43
ARC_ART_c84339	139	EEC70303	0.0	GO:0044710	77	69.3218	45
ARC_ART_c84373	123	ERN02133	0.0	GO:0009507	76	53.1434	38
ARC_ART_c84494	137	KIY92562	0.0	GO:0006412	60	43.8986	43
ARC_ART_c84571	116	XP_001774678	0.0	GO:0016757	68	41.5874	35
ARC_ART_c84712	143	EXB92395	0.3	-	55	33.4982	36
ARC_ART_c84719	128	XP_002538285	0.0	-	64	42.3578	37
ARC_ART_c84743	106	XP_002535015	0.0	-	71	40.4318	35
ARC_ART_c85066	128	XP_006350179	0.8	-	53	32.7278	49
ARC_ART_c85306	110	XP_009108968	0.7	-	58	32.7278	36
ARC_ART_c85430	111	KDD76109	0.2	-	56	33.8834	41
ARC_ART_c85465	119	XP_006645734	0.1	-	59	34.6538	37
ARC_ART_c85482	112	XP_002534449	0.7	-	57	32.3426	35
ARC_ART_c85633	110	XP_002536183	0.1	-	65	35.4242	35
ARC_ART_c85642	123	XP_002536034	0.0	-	58	36.5798	39
ARC_ART_c85687	156	CAA74040	0.0	GO:0006281	66	53.9138	48
ARC_ART_c85725	136	CDY34669	0.5	-	54	33.4982	37
ARC_ART_c85755	118	XP_002540244	0.0	-	92	65.855	39
ARC_ART_c85837	137	XP_002507252	0.2	-	59	34.6538	37
ARC_ART_c85853	135	XP_002974602	0.0	GO:0016149	79	62.3882	43

ARC_ART_c85855	117	XP_002455427	0.2	-	56	34.6538	41
ARC_ART_c85922	134	XP_007210156	0.4	-	58	32.3426	43
ARC_ART_c85937	115	XP_008222710	0.4	-	48	33.4982	35
ARC_ART_c85954	104	XP_007017900	0.2	-	73	33.8834	34
ARC_ART_c85959	263	ERN15094	0.0	-	56	44.669	48
ARC_ART_c86008	122	XP_011001702	0.3	-	61	33.8834	34
ARC_ART_c86009	133	EPS64143	0.0	-	58	37.3502	43
ARC_ART_c86043	115	XP_002954238	0.1	-	64	35.4242	37
ARC_ART_c86133	251	XP_006397217	0.5	-	50	34.2686	44
ARC_ART_c86226	121	XP_006577541	0.5	-	50	33.113	34
ARC_ART_c86234	108	XP_003075081	0.0	GO:0006099	88	64.6994	35
ARC_ART_c86360	113	XP_001786955	0.0	-	86	51.9878	37
ARC_ART_c86378	115	EPS74261	0.0	-	71	50.447	38
ARC_ART_c86385	144	EDQ48523	0.0	GO:0005737	85	53.1434	34
ARC_ART_c86432	215	KDP29135	0.1	-	50	34.6538	61
ARC_ART_c86492	116	KEH15469	0.0	-	70	38.891	34
ARC_ART_c86496	211	XP_009351046	0.0	GO:0034605	76	96.2857	69
ARC_ART_c86511	142	CCO66351	0.0	GO:0008152	71	56.6102	46
ARC_ART_c86539	117	NP_001242469	0.8	-	60	32.3426	35
ARC_ART_c86556	121	XP_010247595	0.0	GO:0004018	89	66.6254	38
ARC_ART_c86562	157	KEH15984	0.0	-	48	37.3502	52
ARC_ART_c86650	233	XP_003534606	0.5	-	50	34.2686	50

ARC_ART_c86726	142	XP_002953444	0.0	GO:0044765	65	42.3578	47
ARC_ART_c86732	112	YP_009104941	0.0	GO:0019752	81	58.151	37
ARC_ART_c86735	129	XP_002506889	0.0	-	76	38.891	34
ARC_ART_c86758	105	XP_002537029	0.0	-	76	48.1358	34
ARC_ART_c86777	130	KDP23890	0.3	-	60	33.4982	38
ARC_ART_c86787	120	AFK41523	0.0	GO:0009451	82	59.6918	34
ARC_ART_c86828	108	XP_002535087	0.0	GO:0016491	91	69.3218	35
ARC_ART_c86882	114	XP_001692446	0.0	-	57	35.8094	38
ARC_ART_c87024	135	NP_565948	0.2	-	55	33.8834	45
ARC_ART_c87044	108	CDY21615	0.0	GO:0006200	79	41.5874	34
ARC_ART_c87057	112	XP_003078975	0.0	GO:0005507	89	67.781	37
ARC_ART_c87102	135	XP_006415810	0.0	-	59	42.743	44
ARC_ART_c87114	189	XP_001416412	0.0	-	56	41.9726	55
ARC_ART_c87136	120	XP_009350057	0.0	-	94	70.0922	38
ARC_ART_c87141	128	XP_002537860	0.0	GO:0050794	77	53.9138	40
ARC_ART_c87143	120	EMT00244	0.0	GO:0006355	88	58.9214	34
ARC_ART_c87169	108	XP_010911369	0.0	GO:0005507	94	63.1586	35
ARC_ART_c87183	123	EPS59991	0.0	GO:0008483	73	52.7582	38
ARC_ART_c87187	111	AEQ94099	0.0	GO:0003824	65	43.5134	35
ARC_ART_c87192	108	XP_002536445	0.0	-	82	58.9214	35
ARC_ART_c87202	164	XP_009141188	0.8	-	55	33.113	36
ARC_ART_c87212	147	XP_009412876	0.8	-	65	32.7278	35
ARC_ART_c87244	136	KFM25371	0.0	-	70	39.2762	40
ARC_ART_c87269	111	AID67480	0.0	GO:0003899	70	43.8986	34
ARC_ART_c87275	119	EYU34731	0.9	-	67	31.9574	34
ARC_ART_c87277	130	XP_007204175	1.0	-	58	32.3426	36

ARC_ART_c87307	112	XP_002536273	0.0	GO:0009451	74	49.2914	35
ARC_ART_c87343	177	EYU31663	0.0	GO:0043231	77	62.3882	59
ARC_ART_c87359	101	KFK30150	0.3	-	64	33.4982	34
ARC_ART_c87446	115	KFM28025	0.0	GO:0034227	73	44.669	38
ARC_ART_c87483	136	CDX94747	0.4	-	55	32.3426	38
ARC_ART_c87550	110	XP_001420650	0.0	-	67	36.965	37
ARC_ART_c87645	139	EEC72535	0.0	GO:0003840	91	65.855	34
ARC_ART_c87683	106	KDD71528	0.0	GO:0008152	77	46.9802	35
ARC_ART_c87701	139	XP_001421384	0.0	GO:0044763	73	52.7582	45
ARC_ART_c87734	129	XP_010999683	0.8	-	67	32.7278	37
ARC_ART_c87760	172	XP_002538251	0.0	GO:0003677	73	41.9726	38
ARC_ART_c87804	112	CCO19022	0.0	GO:0004368	69	44.2838	43
ARC_ART_c87879	133	XP_007051230	0.0	-	67	39.2762	34
ARC_ART_c87887	101	XP_001419436	0.0	-	58	35.8094	41
ARC_ART_c88003	108	XP_008466472	0.0	-	67	43.1282	34
ARC_ART_c88019	112	XP_001770516	0.0	-	67	36.965	34
ARC_ART_c88023	112	XP_011016741	0.0	-	100	38.891	34
ARC_ART_c88091	111	DAA39972	0.4	-	58	32.3426	36
ARC_ART_c88224	105	EMS55213	0.1	-	74	35.4242	35
ARC_ART_c88320	107	XP_004253322	0.2	-	58	33.8834	34
ARC_ART_c88350	161	XP_009350806	0.0	-	90	96.6709	52
ARC_ART_c88391	141	XP_002534739	0.0	-	61	40.0466	42
ARC_ART_c88399	169	XP_002539738	0.0	GO:0050794	78	58.5362	46
ARC_ART_c88411	133	XP_011467315	0.0	GO:0006200	88	56.225	34
ARC_ART_c88465	109	EMS53264	0.0	-	76	40.0466	34
ARC_ART_c88467	117	KFM25849	0.0	-	64	40.0466	37
ARC_ART_c88509	126	XP_006437568	0.0	GO:0004553	74	48.521	39

ARC_ART_c88527	126	EEC77111	0.0	-	70	38.1206	40
ARC_ART_c88617	134	XP_010475084	0.4	-	52	31.5722	44
ARC_ART_c88618	121	XP_001701046	0.0	-	74	43.1282	35
ARC_ART_c88627	169	CAC83001	0.0	GO:0004427	82	59.6918	41
ARC_ART_c88629	261	EPS70027	0.0	-	56	42.3578	44
ARC_ART_c88658	131	XP_006415349	0.4	-	57	33.4982	38
ARC_ART_c88683	118	XP_002500776	0.0	-	66	36.1946	36
ARC_ART_c88688	106	XP_009350052	0.0	-	67	40.4318	34
ARC_ART_c88692	124	XP_002536194	0.0	-	63	37.3502	38
ARC_ART_c88697	139	ACN27633	0.0	GO:0044710	64	41.9726	42
ARC_ART_c88732	109	XP_001753347	0.9	-	57	32.3426	35
ARC_ART_c88746	123	AAN63005	0.0	GO:0030529	73	45.4394	34
ARC_ART_c88750	150	XP_009400356	0.4	-	53	31.5722	41
ARC_ART_c88785	212	KJB44141	0.0	-	68	43.5134	41
ARC_ART_c88805	125	EEC83290	0.0	GO:0016020	68	43.5134	41
ARC_ART_c88821	161	XP_009124532	0.9	-	56	32.7278	41
ARC_ART_c88824	122	XP_002501845	0.0	GO:0006412	87	54.6842	40
ARC_ART_c88840	227	EXB36957	0.0	-	74	47.3654	35
ARC_ART_c88844	119	XP_003611453	0.9	-	61	32.3426	39
ARC_ART_c88855	111	XP_002538704	0.0	-	69	45.8246	36
ARC_ART_c88870	121	XP_002536141	0.0	GO:0004872	81	51.9878	37
ARC_ART_c88874	126	NP_862323	0.0	GO:0003887	88	59.3066	34
ARC_ART_c88884	116	XP_002534739	0.0	GO:0008152	86	58.151	36
ARC_ART_c88896	257	XP_007154367	0.0	-	78	48.9062	38
ARC_ART_c88899	153	XP_002534739	0.0	-	59	41.5874	47
ARC_ART_c88907	149	KDP20204	0.5	-	51	33.4982	52
ARC_ART_c88916	159	XP_006485133	0.2	-	47	32.7278	51
ARC_ART_c88945	141	XP_002945666	0.4	-	67	33.113	34
ARC_ART_c88951	232	AFK35083	0.0	-	76	52.7582	39

ARC_ART_c88967	110	XP_011015255	0.1	-	65	35.4242	35
ARC_ART_c88981	133	XP_002538008	0.0	-	68	34.6538	35
ARC_ART_c88996	162	ACJ84656	0.1	-	53	35.039	49
ARC_ART_c88997	212	EXC01914	0.5	-	61	33.113	34
ARC_ART_c89097	122	KFM27684	0.0	GO:0051287	79	46.9802	34
ARC_ART_c89151	116	XP_009348180	0.0	-	76	56.6102	38
ARC_ART_c89154	126	AIU49068	0.1	-	75	34.6538	36
ARC_ART_c89159	167	XP_009350817	0.0	GO:0044763	90	93.5893	55
ARC_ART_c89171	116	XP_011016603	0.0	GO:0016624	100	79.7221	38
ARC_ART_c89172	177	XP_011016366	0.0	GO:0006096	78	53.5286	38
ARC_ART_c89177	106	XP_002534942	0.0	-	67	39.6614	34
ARC_ART_c89199	145	XP_011073658	0.8	-	55	31.5722	36
ARC_ART_c89206	135	XP_002535442	0.0	-	70	58.151	40
ARC_ART_c89226	181	XP_011080818	0.3	-	41	34.2686	58
ARC_ART_c89237	107	DAA55880	0.0	-	61	39.2762	34
ARC_ART_c89262	109	XP_001700672	0.3	-	58	33.4982	34
ARC_ART_c89271	147	EAY87976	0.0	GO:0006935	79	59.6918	44
ARC_ART_c89290	133	EMT05755	0.0	-	70	47.7506	44
ARC_ART_c89334	154	EEC69469	0.0	GO:0046835	92	93.2041	50
ARC_ART_c89335	107	ABR16943	0.6	-	67	30.8018	34
ARC_ART_c89342	145	XP_003057632	0.0	GO:0016829	60	43.5134	48
ARC_ART_c89370	113	XP_003589128	0.4	-	61	33.4982	36
ARC_ART_c89373	282	EPS74505	0.0	-	68	47.3654	35
ARC_ART_c89401	180	XP_002538865	0.9	-	51	32.7278	45
ARC_ART_c89426	111	YP_001019104	0.0	GO:0003899	81	50.8322	37
ARC_ART_c89439	135	XP_007031998	0.8	-	52	32.7278	34
ARC_ART_c89440	148	KFK44750	0.7	-	59	31.9574	47
ARC_ART_c89491	116	XP_002536710	0.0	-	68	41.2022	38

ARC_ART_c89599	113	CEF97060	0.1	-	76	35.8094	34
ARC_ART_c89601	129	KDP38473	0.2	-	61	34.6538	39
ARC_ART_c89616	104	XP_002536504	0.0	-	70	43.8986	34
ARC_ART_c89617	193	XP_007132735	0.0	-	49	41.2022	63
ARC_ART_c89633	113	XP_002537142	0.0	-	91	59.3066	36
ARC_ART_c89636	159	XP_002504597	0.2	-	72	34.6538	36
ARC_ART_c89640	133	XP_005643067	0.0	GO:0005840	72	52.7582	44
ARC_ART_c89656	152	EPS70505	0.1	-	65	36.1946	40
ARC_ART_c89665	148	XP_010911784	0.0	-	68	55.0694	48
ARC_ART_c89673	121	XP_009350062	0.0	-	86	51.9878	37
ARC_ART_c89694	115	XP_004294169	0.0	GO:0071704	72	48.1358	36
ARC_ART_c89729	108	XP_002537644	0.1	-	64	34.6538	34
ARC_ART_c89733	117	AEP40945	0.0	-	63	37.7354	36
ARC_ART_c89756	126	XP_001697320	0.0	GO:0005786	83	63.929	42
ARC_ART_c89766	211	XP_009370548	0.2	-	56	35.039	46
ARC_ART_c89782	186	KEH15473	0.0	-	54	38.1206	46
ARC_ART_c89784	138	XP_001772436	0.0	-	71	40.4318	35
ARC_ART_c89793	139	ADR30718	0.7	-	57	32.7278	35
ARC_ART_c89827	147	KCW73282	0.7	-	48	32.7278	47
ARC_ART_c89833	121	XP_005843970	0.2	-	64	34.2686	34
ARC_ART_c89843	152	ABK23475	0.7	-	53	32.7278	39
ARC_ART_c89844	199	KDO43782	1.0	-	45	32.7278	83
ARC_ART_c89865	160	ABH09321	0.0	-	80	51.6026	36
ARC_ART_c89879	170	XP_011101877	0.0	-	67	56.225	58
ARC_ART_c89883	118	XP_002539680	0.0	-	75	48.9062	36
ARC_ART_c89920	125	XP_002960029	0.0	GO:0046912	89	69.707	38

ARC_ART_c89972	118	KFM29235	0.7	-	60	32.3426	38
ARC_ART_c89973	248	BAB33421	0.0	-	71	39.2762	35
ARC_ART_c89975	259	XP_009414774	0.3	-	54	35.039	37
ARC_ART_c89980	386	KGN55456	0.0	-	53	37.7354	45
ARC_ART_c89981	434	XP_002980775	0.6	-	43	35.8094	76
ARC_ART_c89982	192	AIU49497	0.0	GO:0008026	70	67.781	62
ARC_ART_c89990	190	XP_001697320	0.0	GO:0005786	90	100.138	62
ARC_ART_c89996	281	XP_003610227	0.0	-	53	39.2762	60
ARC_ART_c90032	106	XP_002536065	0.0	-	76	40.0466	34
ARC_ART_c90045	126	XP_003083805	0.0	GO:0009451	76	50.447	34
ARC_ART_c90051	260	Q33439	0.0	GO:0004129	94	64.3142	35
ARC_ART_c90062	128	BAK00178	0.0	GO:0006631	83	65.4698	42
ARC_ART_c90069	185	XP_002537224	0.0	-	77	37.7354	35
ARC_ART_c90081	111	XP_002537492	0.0	GO:0016491	69	43.8986	36
ARC_ART_c90096	175	AGC78943	0.0	-	58	36.965	34
ARC_ART_c90104	227	AAV44205	0.0	-	64	38.5058	37
ARC_ART_c90110	154	XP_002538563	0.0	-	72	62.7734	44
ARC_ART_c90136	107	CDY36319	0.9	-	52	31.9574	34
ARC_ART_c90141	217	ERN15094	0.0	-	62	39.6614	35
ARC_ART_c90149	125	XP_009767468	0.0	-	67	39.2762	34
ARC_ART_c90153	216	XP_002536736	0.0	GO:0006950	86	86.2705	51
ARC_ART_c90170	145	XP_003080220	0.0	-	59	41.2022	47
ARC_ART_c90174	109	XP_001693179	0.0	GO:0044763	77	49.6766	36
ARC_ART_c90177	177	EEC77111	0.0	GO:0006935	86	56.6102	36
ARC_ART_c90179	229	CDX71648	0.0	-	73	42.3578	34
ARC_ART_c90199	122	EEC71809	0.1	-	65	35.8094	35
ARC_ART_c90215	190	XP_011094405	0.1	-	52	35.8094	55
ARC_ART_c90218	123	EPS57405	0.0	-	71	40.0466	35
ARC_ART_c90229	134	XP_007154641	0.0	GO:0008094	92	69.707	40
ARC_ART_c90276	229	EXC01915	0.0	-	64	46.595	45

ARC_ART_c90280	216	KJB09728	0.0	-	63	34.6538	36
ARC_ART_c90291	138	CDY26072	0.0	GO:0006783	84	73.9442	45
ARC_ART_c90297	141	AGV54793	0.1	-	55	35.4242	47
ARC_ART_c90335	167	XP_004253332	0.0	GO:0005525	92	74.3294	42
ARC_ART_c90377	135	EEE52321	0.0	GO:0043565	100	80.1073	38
ARC_ART_c90393	154	CDP12364	0.0	-	61	43.1282	49
ARC_ART_c90409	124	XP_002539165	0.0	-	71	48.521	39
ARC_ART_c90434	105	XP_002535024	0.0	GO:0030288	88	59.3066	35
ARC_ART_c90443	231	CAN73774	0.1	-	47	34.2686	40
ARC_ART_c90460	291	EPS70020	0.0	-	75	49.6766	44
ARC_ART_c90485	212	KEH33422	0.0	-	70	69.3218	54
ARC_ART_c90489	163	XP_009341691	0.5	-	62	33.4982	50
ARC_ART_c90491	345	XP_010262014	0.6	-	42	34.6538	63
ARC_ART_c90492	211	CAN63636	0.6	-	43	33.4982	51

Supplementary Table S2 Annotated transcripts in IR

Transcript_ID	Sequence length	NCBI nrdb Hit Acc	E-Value	GO	Similarity	Bit Score	Alignment length
IRC_IRT_c1	245	XP_004509500.1	0.0	-	95.00	124.41	66
IRC_IRT_c2	321	XP_002488956.1	0.0	-	100.00	122.48	62
IRC_IRT_c3	640	KDP37477.1	0.0	-	61.00	60.85	59
IRC_IRT_c5	347	XP_006423776.1	0.0	-	93.00	98.21	48
IRC_IRT_c6	293	XP_006363638.1	0.0	GO:0004497	100.00	123.64	58
IRC_IRT_c7	557	AGV54820.1	0.0	-	73.00	81.65	64
IRC_IRT_c8	400	CDY45505.1	0.0	GO:0009507	68.00	70.09	63
IRC_IRT_c9	615	BAJ11784.1	0.0	GO:0009507	95.00	93.97	48
IRC_IRT_c10	492	XP_003610227.1	0.0	GO:0044444	69.00	57.77	42
IRC_IRT_c11	280	XP_002467304.1	0.0	-	96.00	97.44	50
IRC_IRT_c12	334	ACR36970.1	0.0	-	77.00	153.68	108
IRC_IRT_c13	274	AAV44205.1	0.0	-	100.00	83.19	39
IRC_IRT_c14	676	CDY19671.1	0.0	GO:0009507	50.00	78.57	111
IRC_IRT_c15	505	AGZ19352.1	0.0	-	70.00	109.00	102
IRC_IRT_c16	551	EPS74511.1	0.0	-	69.00	59.31	53
IRC_IRT_c17	568	XP_003588355.1	0.0	-	82.00	84.34	52
IRC_IRT_c18	273	CDM82334.1	0.0	GO:0009507	77.00	76.26	54
IRC_IRT_c19	365	BAJ11784.1	0.0	-	91.00	69.71	36
IRC_IRT_c21	250	BAJ11784.1	0.0	-	60.00	53.14	56
IRC_IRT_c22	307	XP_002488946.1	0.0	-	91.00	83.57	45
IRC_IRT_c23	417	KEH33422.1	0.0	-	62.00	39.28	35
IRC_IRT_c24	320	KJB09764.1	0.0	-	54.00	43.13	72
IRC_IRT_c26	242	EXC32750.1	0.5	-	48.00	34.27	54
IRC_IRT_c27	511	BAJ11784.1	0.0	-	65.00	67.78	64
IRC_IRT_c28	249	XP_003599577.1	0.0	-	57.00	41.59	54
IRC_IRT_c30	425	BAJ11784.1	0.0	-	86.00	52.37	36
IRC_IRT_c31	275	EPS74511.1	0.0	GO:0005739	66.00	65.86	63
IRC_IRT_c32	356	AGV54820.1	0.0	GO:0008152	85.00	73.17	47
IRC_IRT_c34	205	XP_009366572.1	0.0	-	68.00	73.17	51
IRC_IRT_c36	314	XP_003638717.1	0.0	GO:0009536	75.00	69.71	49

IRC_IRT_c37	277	XP_003064993.1	0.0	-	89.00	117.47	67
IRC_IRT_c38	424	XP_003614387.1	0.0	GO:0004519	78.00	94.36	70
IRC_IRT_c39	294	KJB44141.1	0.0	GO:0006869	92.00	129.41	70
IRC_IRT_c40	201	XP_002489033.1	0.0	-	92.00	101.68	54
IRC_IRT_c41	222	EXC01914.1	0.0	-	64.00	40.82	34
IRC_IRT_c43	267	XP_006405923.1	0.0	-	73.00	52.37	38
IRC_IRT_c44	263	ERN19185.1	0.0	GO:0005739	86.00	106.30	60
IRC_IRT_c45	258	KEH29749.1	0.0	GO:0009521	70.00	69.71	54
IRC_IRT_c46	348	BAJ11784.1	0.0	-	67.00	60.46	49
IRC_IRT_c48	227	XP_003614387.1	0.0	-	80.00	51.60	36
IRC_IRT_c49	638	CDY63598.1	0.0	GO:0009536	73.00	67.01	49
IRC_IRT_c50	356	AGC78890.1	0.0	GO:0008152	71.00	92.82	80
IRC_IRT_c51	286	XP_006380094.1	0.0	-	88.00	74.33	44
IRC_IRT_c52	197	KJB09764.1	0.0	-	98.00	114.78	56
IRC_IRT_c54	246	XP_003638717.1	0.0	GO:0009507	71.00	71.25	66
IRC_IRT_c55	167	ABA98822.1	0.0	GO:0050660	98.00	78.95	50
IRC_IRT_c56	194	AFK35083.1	0.0	GO:0005739	81.00	55.07	38
IRC_IRT_c57	545	AGZ19352.1	0.0	GO:0009507	80.00	84.73	60
IRC_IRT_c58	292	XP_003588326.1	0.0	-	75.00	50.83	41
IRC_IRT_c59	346	EPS70023.1	0.0	-	64.00	40.43	37
IRC_IRT_c60	171	EAY93748.1	0.0	-	54.00	41.59	55
IRC_IRT_c62	393	BAJ11784.1	0.0	-	97.00	83.19	40
IRC_IRT_c64	212	EPS74531.1	0.0	-	82.00	50.83	34
IRC_IRT_c65	262	EPS70027.1	0.0	GO:0009536	67.00	68.94	58
IRC_IRT_c66	204	XP_002489152.1	0.0	-	70.00	42.74	34
IRC_IRT_c67	199	XP_006423776.1	0.0	-	89.00	75.49	39
IRC_IRT_c68	301	AAV44205.1	0.0	GO:0009507	96.00	182.19	95
IRC_IRT_c70	176	XP_002489002.1	0.0	-	53.00	42.36	43
IRC_IRT_c71	314	XP_003637074.1	0.0	-	68.00	39.66	41
IRC_IRT_c73	352	BAJ11784.1	0.0	-	53.00	44.28	58
IRC_IRT_c74	146	XP_002489102.1	0.0	-	97.00	88.58	42
IRC_IRT_c75	252	ABH09321.1	0.0	-	52.00	59.31	82
IRC_IRT_c81	331	XP_003616487.1	0.0	-	57.00	38.89	47

IRC_IRT_c85	198	KEH16146.1	0.1	-	46.00	35.42	62
IRC_IRT_c88	354	KEH29749.1	0.0	-	63.00	59.69	57
IRC_IRT_c90	226	ABH09321.1	0.0	-	58.00	36.19	41
IRC_IRT_c91	290	EPS74505.1	0.0	GO:0009507	73.00	63.16	46
IRC_IRT_c93	186	ABH09321.1	0.0	-	63.00	58.15	55
IRC_IRT_c94	270	XP_003588337.1	0.0	-	65.00	94.36	83
IRC_IRT_c95	245	KJB49896.1	0.0	-	54.00	48.52	64
IRC_IRT_c96	238	ABA95137.1	0.0	GO:0004553	100.00	161.00	73
IRC_IRT_c97	223	EPS74533.1	0.0	-	62.00	55.45	51
IRC_IRT_c100	167	XP_009388207.1	0.0	-	68.00	55.84	51
IRC_IRT_c101	223	AAV44205.1	0.0	-	68.00	45.44	44
IRC_IRT_c103	211	KJB11774.1	0.0	GO:0070330	92.00	71.63	3.80E+01
IRC_IRT_c106	261	YP_001152214.1	0.3	-	61.00	33.88	36
IRC_IRT_c111	295	EPS74511.1	0.0	-	64.00	57.00	51
IRC_IRT_c112	478	AGC78943.1	0.0	GO:0005739	79.00	94.74	68
IRC_IRT_c114	250	XP_004231110.1	0.6	-	47.00	33.88	67
IRC_IRT_c115	276	AGZ19352.1	0.0	GO:0006869	78.00	75.49	55
IRC_IRT_c117	267	XP_009388289.1	0.0	-	76.00	59.31	50
IRC_IRT_c119	521	AGZ19352.1	0.0	GO:0009536	73.00	65.08	65
IRC_IRT_c120	190	XP_002488936.1	0.0	-	100.00	79.72	38
IRC_IRT_c122	288	EXC01912.1	0.0	-	67.00	53.91	43
IRC_IRT_c128	200	KJB48926.1	0.0	-	100.00	70.09	34
IRC_IRT_c131	541	XP_002884233.1	0.3	-	57.00	35.04	49
IRC_IRT_c132	584	XP_009599052.1	0.1	-	59.00	39.28	37
IRC_IRT_c133	302	ADK60808.1	0.0	GO:0009507	78.00	77.03	51
IRC_IRT_c134	525	XP_002979380.1	0.9	-	49.00	35.04	63
IRC_IRT_c135	208	XP_010516347.1	0.4	-	63.00	34.27	41
IRC_IRT_c136	216	EXC34899.1	0.0	-	70.00	55.84	48
IRC_IRT_c137	606	XP_009138278.1	1.0	-	45.00	35.04	61
IRC_IRT_c141	138	CAE75886.1	0.0	GO:0003676	92.00	75.49	39
IRC_IRT_c144	151	XP_011008093.1	0.2	-	60.00	35.04	43
IRC_IRT_c145	193	XP_001759255.1	0.0	-	66.00	44.67	45
IRC_IRT_c147	223	EMS49548.1	0.6	-	55.00	33.88	61

IRC_IRT_c150	404	NP_001063453.2	0.0	GO:0043231	63.00	140.20	138
IRC_IRT_c153	330	ABH09321.1	0.0	-	63.00	43.51	57
IRC_IRT_c154	227	AAV24822.1	0.0	GO:0016301	87.00	72.79	39
IRC_IRT_c155	423	KJB31094.1	0.0	-	72.00	46.98	40
IRC_IRT_c158	302	ABH09321.1	0.0	-	69.00	80.88	78
IRC_IRT_c161	121	AFW79598.1	0.0	GO:0006499	94.00	65.47	35
IRC_IRT_c162	366	EPS74505.1	0.0	-	81.00	82.42	58
IRC_IRT_c165	272	EPS73531.1	0.2	-	53.00	35.42	56
IRC_IRT_c166	146	EMS68864.1	0.1	-	52.00	34.27	44
IRC_IRT_c169	253	CDY20228.1	0.0	GO:0016984	92.00	123.25	7.00E+01
IRC_IRT_c174	217	AGC78945.1	0.0	GO:0005739	85.00	101.68	60
IRC_IRT_c175	144	XP_002538076.1	0.0	-	65.00	46.60	41
IRC_IRT_c177	142	XP_005845244.1	0.2	-	70.00	35.04	34
IRC_IRT_c179	324	AGV54820.1	0.0	GO:0009507	69.00	112.08	92
IRC_IRT_c180	284	XP_003637074.1	0.0	GO:0016787	92.00	97.44	52
IRC_IRT_c181	132	XP_002536322.1	0.0	GO:0005524	85.00	54.30	34
IRC_IRT_c184	181	XP_011073040.1	0.5	-	56.00	33.88	50
IRC_IRT_c188	374	XP_002269413.1	0.4	-	50.00	35.81	101
IRC_IRT_c190	247	EMS45449.1	0.0	GO:0043167	78.00	76.26	60
IRC_IRT_c195	132	XP_008385601.1	0.0	GO:0005975	78.00	54.30	38
IRC_IRT_c198	149	XP_005644245.1	0.1	-	56.00	34.27	41
IRC_IRT_c201	459	AGC78943.1	0.0	-	65.00	87.43	85
IRC_IRT_c206	171	XP_002538425.1	0.7	-	57.00	30.80	42
IRC_IRT_c207	221	XP_002448466.1	0.0	GO:0008152	69.00	57.38	56
IRC_IRT_c212	347	XP_003588337.1	0.0	-	56.00	51.99	60
IRC_IRT_c215	329	XP_003604156.1	0.0	-	41.00	45.05	91
IRC_IRT_c219	217	BAD07868.1	0.0	GO:0009536	98.00	98.98	52
IRC_IRT_c221	278	ERN00555.1	0.0	GO:1901576	78.00	75.87	50
IRC_IRT_c222	471	CDM84611.1	0.0	GO:0005739	88.00	171.01	97
IRC_IRT_c225	242	XP_002538319.1	0.0	GO:0004871	67.00	65.47	77
IRC_IRT_c226	158	CDM84611.1	0.0	-	81.00	64.70	43
IRC_IRT_c227	384	XP_003588337.1	0.0	GO:0005739	94.00	121.32	59
IRC_IRT_c228	120	XP_002536837.1	0.0	-	92.00	76.64	39

IRC_IRT_c231	116	XP_002536229.1	0.0	GO:0008152	74.00	49.29	35
IRC_IRT_c233	302	XP_003599577.1	0.0	-	66.00	42.36	36
IRC_IRT_c234	352	XP_003637074.1	0.0	-	52.00	49.29	74
IRC_IRT_c239	131	XP_001778351.1	0.1	-	62.00	35.42	43
IRC_IRT_c242	162	KFM25362.1	0.0	GO:0008152	72.00	59.31	50
IRC_IRT_c244	758	CAE04765.3	0.0	GO:0004523	84.00	153.30	104
IRC_IRT_c246	185	XP_002967787.1	0.0	GO:0050896	72.00	45.82	36
IRC_IRT_c248	358	BAB63679.1	0.0	-	100.00	196.05	94
IRC_IRT_c251	197	XP_002538077.1	0.0	-	63.00	36.19	38
IRC_IRT_c253	163	EPS74511.1	0.0	-	79.00	71.25	53
IRC_IRT_c257	503	ACN31557.1	0.0	-	96.00	100.14	52
IRC_IRT_c260	120	XP_007227062.1	0.1	-	58.00	33.50	39
IRC_IRT_c263	193	XP_011016263.1	0.0	GO:0006415	93.00	113.62	64
IRC_IRT_c266	356	EXC01912.1	0.0	-	73.00	44.67	34
IRC_IRT_c269	217	XP_002959312.1	0.0	GO:0016740	67.00	78.18	71
IRC_IRT_c270	308	EAY79110.1	0.0	-	82.00	87.43	57
IRC_IRT_c271	150	KEH15180.1	0.4	-	60.00	33.50	38
IRC_IRT_c278	187	AAU90124.1	0.0	-	65.00	49.29	55
IRC_IRT_c279	192	XP_004953629.1	0.7	-	60.00	33.11	40
IRC_IRT_c281	121	EAZ05096.1	0.6	-	100.00	32.73	39
IRC_IRT_c283	151	KIZ06629.1	0.0	GO:0005786	77.00	67.01	48
IRC_IRT_c295	137	CDM81900.1	0.9	-	55.00	32.34	38
IRC_IRT_c308	132	XP_009793545.1	0.0	GO:0003867	62.00	44.67	43
IRC_IRT_c310	242	XP_003605618.1	0.2	-	61.00	34.65	34
IRC_IRT_c312	163	XP_009350810.1	0.0	-	64.00	48.14	50
IRC_IRT_c316	281	ADK60808.1	0.0	-	77.00	56.23	36
IRC_IRT_c324	361	XP_009358397.1	0.4	-	51.00	35.42	45
IRC_IRT_c326	220	XP_009385446.1	0.2	-	56.00	35.04	41
IRC_IRT_c327	154	AGV54793.1	0.1	-	50.00	35.81	51
IRC_IRT_c330	142	EMS47955.1	0.0	-	68.00	36.58	41
IRC_IRT_c331	197	XP_002536340.1	0.0	-	59.00	35.04	47
IRC_IRT_c332	115	XP_002981097.1	0.0	-	57.00	43.13	38
IRC_IRT_c333	314	AAM08568.1	0.0	GO:0009536	66.00	142.90	125

IRC_IRT_c334	212	AGC78890.1	0.0	-	69.00	45.05	49
IRC_IRT_c337	157	XP_002536962.1	0.0	-	63.00	47.75	49
IRC_IRT_c344	251	ERN01761.1	0.9	-	45.00	33.50	68
IRC_IRT_c345	232	ABH09321.1	0.0	-	63.00	46.60	52
IRC_IRT_c350	367	ABA99784.2	0.0	-	98.00	240.35	117
IRC_IRT_c353	292	KJB49896.1	0.0	-	69.00	62.00	52
IRC_IRT_c358	107	XP_002534501.1	0.0	GO:0016491	74.00	44.28	35
IRC_IRT_c360	126	CAE03600.2	0.0	GO:0003676	100.00	85.11	41
IRC_IRT_c361	134	XP_003604154.1	0.0	GO:0009536	75.00	46.60	36
IRC_IRT_c364	110	EPS70023.1	0.0	-	58.00	35.04	36
IRC_IRT_c365	112	XP_002538039.1	0.0	GO:0008658	82.00	54.30	34
IRC_IRT_c370	209	YP_006280978.1	0.0	GO:0016491	77.00	57.38	35
IRC_IRT_c373	183	EEE50471.1	0.0	GO:0009735	87.00	91.28	58
IRC_IRT_c374	155	CEF98371.1	0.0	-	62.00	38.89	48
IRC_IRT_c378	312	BAJ86385.1	0.0	-	77.00	70.86	49
IRC_IRT_c382	388	ABA99568.1	0.0	GO:0005506	98.00	144.05	70
IRC_IRT_c383	420	XP_001785944.1	0.0	-	82.00	55.45	34
IRC_IRT_c389	151	CDY55452.1	0.4	-	64.00	33.11	34
IRC_IRT_c395	238	XP_005842766.1	0.0	-	72.00	65.08	47
IRC_IRT_c396	223	ERN00555.1	0.0	-	68.00	42.74	35
IRC_IRT_c401	140	XP_009350057.1	0.0	-	89.00	58.54	39
IRC_IRT_c404	201	BAC05657.1	0.0	GO:0004523	87.00	101.68	58
IRC_IRT_c405	135	KIY95230.1	0.8	-	55.00	32.73	34
IRC_IRT_c406	515	ABA99952.1	0.0	GO:0006367	92.00	187.96	100
IRC_IRT_c412	280	EEC70277.1	0.0	GO:0044781	88.00	51.60	35
IRC_IRT_c419	147	EDQ48547.1	0.0	-	67.00	45.82	49
IRC_IRT_c423	128	XP_008464045.1	0.4	-	54.00	33.50	50
IRC_IRT_c424	470	ERN19184.1	0.0	GO:0005739	75.00	100.52	77
IRC_IRT_c425	191	XP_002868120.1	0.5	-	54.00	33.50	44
IRC_IRT_c431	185	XP_004491352.1	0.0	-	61.00	40.43	44
IRC_IRT_c433	506	CDP21352.1	0.0	-	61.00	57.00	78
IRC_IRT_c434	158	XP_006283310.1	0.0	GO:0009507	58.00	43.90	46
IRC_IRT_c447	156	XP_002961432.1	0.0	-	56.00	37.35	58

IRC_IRT_c450	256	XP_006279312.1	0.0	-	54.00	35.81	35
IRC_IRT_c451	244	AFW84560.1	0.0	-	77.00	57.00	44
IRC_IRT_c454	470	BAJ11784.1	0.0	-	73.00	45.44	38
IRC_IRT_c456	199	XP_003569141.1	0.5	-	58.00	33.50	41
IRC_IRT_c468	143	XP_009350812.1	0.0	-	72.00	57.00	50
IRC_IRT_c469	499	ABA96812.2	0.0	GO:0005739	90.00	154.07	82
IRC_IRT_c476	325	AFK35083.1	0.0	-	72.00	46.60	40
IRC_IRT_c488	161	XP_002535442.1	0.0	GO:0030254	86.00	63.16	37
IRC_IRT_c490	197	XP_003614391.1	0.0	-	58.00	41.97	41
IRC_IRT_c491	151	AAK55475.1	0.0	GO:0008270	100.00	90.12	42
IRC_IRT_c496	179	CCO19780.1	0.0	GO:0016747	72.00	52.76	55
IRC_IRT_c504	282	ABA96816.1	0.0	GO:0005739	89.00	132.49	95
IRC_IRT_c514	161	EEE67873.1	0.3	-	54.00	34.65	48
IRC_IRT_c517	254	BAJ11784.1	0.1	-	57.00	36.58	38
IRC_IRT_c519	136	AGC78969.1	0.0	-	69.00	55.84	49
IRC_IRT_c526	179	XP_003599576.1	0.0	-	69.00	51.22	39
IRC_IRT_c539	139	XP_002537159.1	0.0	-	65.00	55.45	46
IRC_IRT_c543	212	XP_003627937.1	0.0	-	78.00	49.68	38
IRC_IRT_c545	191	XP_006857298.1	0.0	-	54.00	40.05	55
IRC_IRT_c547	111	XP_002535990.1	0.0	GO:0003887	80.00	51.22	36
IRC_IRT_c550	118	XP_008779884.1	0.8	-	47.00	31.19	40
IRC_IRT_c556	214	AAN04182.1	0.0	GO:0003676	100.00	143.28	70
IRC_IRT_c567	102	XP_002537941.1	0.0	GO:0015413	85.00	50.45	34
IRC_IRT_c569	158	XP_002536645.1	0.0	GO:0000155	90.00	95.13	52
IRC_IRT_c580	175	BAK02440.1	0.0	GO:0020037	87.00	74.71	48
IRC_IRT_c584	247	XP_002535682.1	0.0	GO:0016746	75.00	76.64	62
IRC_IRT_c592	171	ABH09321.1	0.0	-	59.00	37.74	42
IRC_IRT_c594	469	KJB09764.1	0.0	-	71.00	45.82	35
IRC_IRT_c595	239	EPS74505.1	0.0	GO:0005739	70.00	57.38	54
IRC_IRT_c601	127	EEC81066.1	0.1	-	68.00	35.81	35
IRC_IRT_c604	178	XP_009350805.1	0.0	GO:1901363	72.00	77.80	58
IRC_IRT_c605	380	AAM14693.1	0.0	GO:0003964	95.00	142.51	68
IRC_IRT_c607	177	XP_007132206.1	0.0	-	94.00	72.02	36

IRC_IRT_c613	317	ADK60808.1	0.0	-	62.00	43.90	45
IRC_IRT_c614	154	XP_010236186.1	0.7	-	48.00	31.96	43
IRC_IRT_c615	139	XP_002539254.1	0.0	-	82.00	66.24	45
IRC_IRT_c616	127	BAJ95181.1	0.1	-	67.00	34.65	34
IRC_IRT_c619	139	XP_008350175.1	0.0	GO:0017111	70.00	45.44	37
IRC_IRT_c621	135	XP_010314936.1	0.1	-	58.00	35.81	41
IRC_IRT_c623	687	DAA02083.1	0.0	GO:0006508	79.00	342.04	234
IRC_IRT_c627	109	AFW60279.1	0.3	-	67.00	33.50	34
IRC_IRT_c629	127	XP_007212068.1	0.0	-	65.00	42.36	4.30E+01
IRC_IRT_c630	103	XP_002462705.1	0.0	GO:0042802	97.00	68.94	34
IRC_IRT_c634	151	EAY84569.1	0.0	GO:0016627	72.00	52.76	44
IRC_IRT_c648	121	XP_002536403.1	0.1	-	66.00	34.27	39
IRC_IRT_c659	131	ACU24603.1	0.0	GO:0016491	57.00	45.05	45
IRC_IRT_c660	232	EMT11891.1	0.0	GO:0005524	85.00	66.24	40
IRC_IRT_c661	170	XP_011016736.1	0.0	-	71.00	70.09	56
IRC_IRT_c662	179	XP_006665175.1	0.0	GO:0009941	64.00	46.21	45
IRC_IRT_c663	135	EMT22473.1	0.0	GO:0001510	77.00	56.23	44
IRC_IRT_c669	168	KIY99539.1	0.0	-	68.00	69.71	57
IRC_IRT_c673	183	XP_007215540.1	0.0	-	58.00	38.51	53
IRC_IRT_c678	150	XP_001774375.1	0.0	-	64.00	36.97	39
IRC_IRT_c680	262	XP_002502109.1	0.0	GO:0006259	63.00	58.54	86
IRC_IRT_c690	117	CAD40418.3	0.0	GO:0046872	100.00	79.72	38
IRC_IRT_c695	153	EEC81325.1	0.0	GO:0055085	87.00	77.03	48
IRC_IRT_c696	262	KFK23449.1	0.0	-	72.00	60.46	50
IRC_IRT_c701	174	XP_002537871.1	0.0	-	68.00	42.74	48
IRC_IRT_c703	126	XP_002535669.1	0.0	-	68.00	46.98	41
IRC_IRT_c706	137	XP_004982811.1	0.0	-	61.00	42.36	42
IRC_IRT_c707	141	XP_002972578.1	0.3	-	64.00	33.88	42
IRC_IRT_c709	212	KEH22026.1	0.5	-	55.00	31.57	36
IRC_IRT_c713	172	XP_002536617.1	0.2	-	55.00	33.50	38
IRC_IRT_c715	136	XP_009114732.1	0.0	GO:0016798	69.00	44.67	42
IRC_IRT_c717	154	AAG41902.1	0.0	-	56.00	40.05	51
IRC_IRT_c730	119	NP_045890.1	0.8	-	62.00	31.96	37

IRC_IRT_c735	138	CEF99636.1	0.0	-	57.00	38.51	45
IRC_IRT_c741	109	XP_002537545.1	0.0	-	62.00	36.97	35
IRC_IRT_c745	130	XP_002446820.1	0.8	-	55.00	32.34	38
IRC_IRT_c751	122	XP_002538475.1	0.0	GO:0009536	82.00	63.54	39
IRC_IRT_c761	122	EEC77198.1	0.0	GO:0006259	75.00	56.61	40
IRC_IRT_c762	125	AAR15338.1	0.0	GO:0009536	83.00	48.14	36
IRC_IRT_c769	138	XP_002538274.1	0.0	GO:0016866	77.00	51.99	40
IRC_IRT_c772	130	EEC69469.1	0.0	GO:0005975	68.00	55.07	44
IRC_IRT_c775	276	XP_010266663.1	0.5	-	54.00	34.65	37
IRC_IRT_c776	149	XP_001786614.1	0.0	GO:0008152	72.00	47.75	47
IRC_IRT_c777	150	KEH22219.1	0.7	-	54.00	33.11	44
IRC_IRT_c781	145	ABA98594.1	0.0	-	76.00	67.01	47
IRC_IRT_c784	146	XP_005651291.1	0.2	-	61.00	34.65	44
IRC_IRT_c785	119	EEC66960.1	0.4	-	56.00	33.50	37
IRC_IRT_c787	309	XP_003082229.1	0.0	-	59.00	37.35	44
IRC_IRT_c788	127	EEC70332.1	0.0	GO:0006413	94.00	62.39	37
IRC_IRT_c800	175	YP_001152206.1	0.0	-	63.00	43.90	38
IRC_IRT_c801	110	XP_002540005.1	0.0	GO:0043565	80.00	55.45	36
IRC_IRT_c802	161	XP_002535024.1	0.0	GO:0030288	84.00	84.73	51
IRC_IRT_c804	125	AHK22782.1	0.0	GO:0004129	97.00	82.80	41
IRC_IRT_c810	148	XP_002867731.1	0.0	GO:0004357	81.00	76.64	49
IRC_IRT_c811	142	XP_003059702.1	0.0	-	59.00	44.67	47
IRC_IRT_c813	113	EEE52320.1	0.0	GO:0055085	94.00	72.40	37
IRC_IRT_c820	110	XP_002539789.1	0.0	-	77.00	55.07	36
IRC_IRT_c823	139	XP_002538853.1	0.0	-	95.00	89.35	44
IRC_IRT_c825	155	EEC79944.1	0.0	GO:0000278	87.00	68.17	49
IRC_IRT_c830	175	XP_002538398.1	0.0	GO:0004871	72.00	61.23	51
IRC_IRT_c837	124	EEC80696.1	0.0	GO:0005739	100.00	83.96	40
IRC_IRT_c840	330	BAB33421.1	0.0	-	53.00	40.05	63
IRC_IRT_c845	117	XP_005647421.1	0.2	-	51.00	33.88	39
IRC_IRT_c849	205	XP_009350076.1	0.0	GO:0008270	87.00	99.75	62
IRC_IRT_c855	109	XP_009350806.1	0.0	-	79.00	43.51	34
IRC_IRT_c864	230	KEH17348.1	0.0	-	48.00	29.65	41

IRC_IRT_c868	112	XP_009350816.1	0.2	-	71.00	34.27	35
IRC_IRT_c871	124	XP_005643306.1	0.2	-	60.00	34.65	38
IRC_IRT_c872	138	XP_002535695.1	0.3	-	58.00	33.88	39
IRC_IRT_c874	165	ADE77844.1	0.9	-	52.00	32.34	36
IRC_IRT_c876	138	DAA05105.1	0.0	GO:0043565	97.00	91.66	46
IRC_IRT_c880	212	XP_011083900.1	0.0	-	63.00	51.60	57
IRC_IRT_c882	147	KIZ00650.1	0.0	-	80.00	44.28	36
IRC_IRT_c884	120	ABG65886.1	0.0	GO:0030247	100.00	89.35	37
IRC_IRT_c893	125	EEC76122.1	0.0	GO:0050660	83.00	62.77	37
IRC_IRT_c895	211	EXC50692.1	0.7	-	53.00	32.73	43
IRC_IRT_c900	193	XP_002537752.1	0.0	-	55.00	45.44	61
IRC_IRT_c910	137	EPS74704.1	0.3	-	55.00	31.57	40
IRC_IRT_c919	114	NP_001141252.1	0.0	GO:0016021	94.00	73.94	37
IRC_IRT_c920	120	XP_002539333.1	0.0	-	70.00	43.51	37
IRC_IRT_c926	130	XP_003616486.1	0.0	-	85.00	65.08	35
IRC_IRT_c934	121	XP_001782628.1	0.0	GO:0055114	75.00	62.00	40
IRC_IRT_c936	151	XP_002536402.1	0.0	GO:0004871	76.00	42.36	34
IRC_IRT_c937	203	XP_002277415.2	0.0	GO:0005829	81.00	57.00	38
IRC_IRT_c939	167	XP_002959673.1	0.0	GO:0004611	76.00	59.31	43
IRC_IRT_c942	216	ABF95979.1	0.0	GO:0003964	100.00	153.30	72
IRC_IRT_c946	242	XP_002958279.1	0.0	GO:0003746	94.00	78.95	3.80E+01
IRC_IRT_c947	400	ABF93933.1	0.0	GO:0004523	98.00	252.68	127
IRC_IRT_c952	524	ABA97679.1	0.0	GO:0004523	95.00	219.55	156
IRC_IRT_c957	122	KJB10433.1	0.0	-	76.00	47.37	34
IRC_IRT_c958	124	EYU46267.1	0.0	GO:0016491	66.00	46.21	39
IRC_IRT_c966	108	EEC76877.1	0.0	GO:0046961	97.00	74.33	36
IRC_IRT_c968	198	KGN47269.1	0.2	-	60.00	34.65	38
IRC_IRT_c977	116	NP_001176887.1	0.0	GO:0006468	100.00	78.95	38
IRC_IRT_c981	136	BAF01964.1	0.0	-	72.00	43.51	37
IRC_IRT_c995	142	AHZ00655.1	0.0	GO:0004399	76.00	53.14	43
IRC_IRT_c998	142	XP_003627685.1	0.7	-	65.00	33.11	35
IRC_IRT_c1000	168	EEC76122.1	0.0	GO:0044763	76.00	73.94	55
IRC_IRT_c1007	165	XP_002540131.1	0.0	GO:0005488	72.00	70.86	55

IRC_IRT_c1019	378	BAD69364.1	0.0	-	94.00	110.92	59
IRC_IRT_c1028	197	EDQ48547.1	0.0	-	71.00	40.43	64
IRC_IRT_c1029	110	XP_007154371.1	0.0	-	68.00	39.28	35
IRC_IRT_c1030	175	XP_001786560.1	0.0	GO:0044763	67.00	50.45	52
IRC_IRT_c1031	135	XP_003081198.1	0.0	GO:0045454	80.00	50.06	35
IRC_IRT_c1035	419	AAV25049.1	0.0	GO:0004523	83.00	150.60	96
IRC_IRT_c1040	145	XP_002954941.1	0.0	-	61.00	48.52	52
IRC_IRT_c1044	125	EEE52321.1	0.0	-	70.00	55.45	40
IRC_IRT_c1052	121	AFW84372.1	0.0	GO:0006468	100.00	81.65	40
IRC_IRT_c1055	117	XP_001769492.1	0.0	-	70.00	41.97	37
IRC_IRT_c1056	151	XP_004952295.1	0.0	GO:0010035	76.00	62.39	50
IRC_IRT_c1063	210	AAV32166.1	0.0	GO:0004185	91.00	100.91	58
IRC_IRT_c1064	148	EPS74534.1	0.0	-	88.00	65.86	35
IRC_IRT_c1071	127	XP_002534652.1	0.6	-	58.00	33.11	34
IRC_IRT_c1072	125	XP_009350805.1	0.0	GO:0003824	73.00	61.23	41
IRC_IRT_c1079	215	XP_001693504.1	0.1	-	53.00	36.19	56
IRC_IRT_c1083	135	EAZ23921.1	0.0	GO:0047427	92.00	74.71	40
IRC_IRT_c1092	154	XP_005843413.1	0.0	GO:0044763	70.00	57.77	51
IRC_IRT_c1094	144	XP_002536193.1	0.0	GO:0015748	97.00	95.13	48
IRC_IRT_c1102	288	XP_003616487.1	0.0	-	57.00	41.97	49
IRC_IRT_c1112	331	NP_001066389.1	0.2	-	41.00	36.19	90
IRC_IRT_c1117	496	AAP52617.1	0.0	GO:0004523	100.00	157.53	72
IRC_IRT_c1119	432	BAD23659.1	0.0	GO:0003676	100.00	77.80	37
IRC_IRT_c1121	155	NP_001173303.1	0.0	GO:0004553	100.00	112.85	51
IRC_IRT_c1127	186	XP_002538654.1	0.0	-	70.00	58.15	48
IRC_IRT_c1130	496	ABA96008.1	0.0	GO:0003676	66.00	119.01	114
IRC_IRT_c1135	169	BAK06974.1	0.1	-	47.00	35.81	46
IRC_IRT_c1137	123	CAE76021.1	0.0	GO:0003964	100.00	87.04	41
IRC_IRT_c1144	178	XP_011089001.1	0.7	-	50.00	33.11	51
IRC_IRT_c1153	112	XP_002534869.1	0.0	GO:0008152	75.00	45.82	37
IRC_IRT_c1156	297	KFK40658.1	0.0	-	58.00	56.23	68
IRC_IRT_c1159	113	XP_011041626.1	0.3	-	57.00	33.88	35
IRC_IRT_c1160	227	XP_003614382.1	0.5	-	53.00	30.80	39

IRC_IRT_c1162	111	XP_002507974.1	0.4	-	52.00	30.80	36
IRC_IRT_c1169	114	ABA95904.1	0.0	GO:0005840	97.00	75.87	37
IRC_IRT_c1173	125	CAE04438.2	0.0	GO:0004523	100.00	90.51	41
IRC_IRT_c1174	227	XP_002863303.1	0.0	-	52.00	37.35	57
IRC_IRT_c1177	357	KJB06193.1	1.0	-	67.00	32.73	34
IRC_IRT_c1182	110	XP_002536184.1	0.0	-	63.00	33.50	36
IRC_IRT_c1184	140	AAM76355.1	0.0	GO:0016023	100.00	102.06	46
IRC_IRT_c1187	145	XP_002534959.1	0.0	GO:0008233	81.00	78.18	48
IRC_IRT_c1190	125	AAX94936.1	0.0	-	86.00	58.54	36
IRC_IRT_c1192	204	XP_005651412.1	0.0	GO:0005488	77.00	87.04	62
IRC_IRT_c1193	140	XP_003078193.1	0.0	GO:0003861	69.00	66.63	55
IRC_IRT_c1196	150	XP_002537421.1	0.0	-	58.00	48.91	43
IRC_IRT_c1198	156	XP_002534909.1	0.0	-	76.00	39.66	34
IRC_IRT_c1199	195	XP_002536712.1	0.0	-	69.00	52.76	43
IRC_IRT_c1204	134	XP_010501736.1	0.0	GO:0009156	86.00	63.16	43
IRC_IRT_c1206	143	XP_002539091.1	0.3	-	64.00	33.88	34
IRC_IRT_c1214	133	XP_005851284.1	0.0	GO:0005829	79.00	61.23	39
IRC_IRT_c1215	143	XP_002538438.1	0.0	-	68.00	43.51	44
IRC_IRT_c1216	155	CCO17359.1	0.0	GO:0015992	72.00	58.54	51
IRC_IRT_c1219	125	XP_009337347.1	0.5	-	58.00	32.73	39
IRC_IRT_c1220	112	XP_002536630.1	0.0	-	64.00	41.20	37
IRC_IRT_c1222	252	XP_011016603.1	0.0	-	49.00	46.60	79
IRC_IRT_c1223	129	XP_002540234.1	0.0	-	73.00	46.60	41
IRC_IRT_c1224	150	XP_006405922.1	0.0	-	64.00	46.60	48
IRC_IRT_c1225	139	KIZ00619.1	0.0	GO:0016829	77.00	53.91	44
IRC_IRT_c1227	134	XP_001699275.1	0.7	-	56.00	31.57	44
IRC_IRT_c1228	125	XP_002540535.1	0.0	-	97.00	67.01	35
IRC_IRT_c1230	118	KFM28216.1	0.0	GO:0005840	86.00	63.16	37
IRC_IRT_c1231	135	EEC69860.1	0.0	-	74.00	39.66	39
IRC_IRT_c1234	127	XP_002954075.1	0.0	GO:0004824	77.00	60.08	40
IRC_IRT_c1244	169	XP_010942030.1	0.6	-	66.00	33.50	36
IRC_IRT_c1246	177	XP_002538818.1	0.0	GO:0005975	79.00	75.49	48
IRC_IRT_c1251	135	BAD94976.1	0.0	-	60.00	34.27	41

IRC_IRT_c1258	145	XP_001771905.1	0.9	-	56.00	32.73	44
IRC_IRT_c1264	151	CAD40755.2	0.0	GO:0016020	98.00	110.92	50
IRC_IRT_c1267	110	XP_002534942.1	0.0	-	91.00	68.94	36
IRC_IRT_c1268	258	ABA98432.1	0.0	GO:0005739	100.00	191.05	85
IRC_IRT_c1270	166	EHK62689.1	0.0	-	65.00	48.14	46
IRC_IRT_c1280	186	KEH43936.1	0.7	-	55.00	33.50	38
IRC_IRT_c1281	291	XP_005842766.1	0.0	-	82.00	68.94	46
IRC_IRT_c1286	112	XP_009416242.1	0.3	-	58.00	33.88	41
IRC_IRT_c1297	122	AAV65378.1	0.0	GO:0003824	69.00	51.22	42
IRC_IRT_c1306	227	XP_002538508.1	0.0	-	69.00	40.43	36
IRC_IRT_c1309	134	EEC68421.1	0.0	GO:0016021	90.00	73.94	44
IRC_IRT_c1310	127	XP_002539615.1	0.8	-	72.00	31.96	40
IRC_IRT_c1312	123	XP_001693400.1	0.0	GO:0004756	89.00	67.40	38
IRC_IRT_c1316	323	AGC78943.1	0.0	-	72.00	67.01	55
IRC_IRT_c1320	320	AAK92562.1	0.0	GO:0005739	80.00	83.57	56
IRC_IRT_c1322	603	XP_003614394.1	0.0	-	58.00	63.93	72
IRC_IRT_c1324	145	XP_002500999.1	0.0	GO:0008233	91.00	81.65	46
IRC_IRT_c1327	201	XP_007160345.1	0.0	GO:0050896	62.00	54.68	64
IRC_IRT_c1331	144	AAM08509.1	0.0	GO:0090502	86.00	50.45	38
IRC_IRT_c1339	134	CDP22216.1	0.0	GO:0006200	76.00	50.06	42
IRC_IRT_c1355	130	XP_002536189.1	0.0	GO:0016491	78.00	62.77	41
IRC_IRT_c1359	158	XP_003083341.1	0.0	GO:0055114	79.00	66.24	39
IRC_IRT_c1360	121	XP_002536999.1	0.0	-	67.00	50.45	40
IRC_IRT_c1365	119	XP_002535386.1	0.0	-	83.00	57.38	36
IRC_IRT_c1369	152	XP_009799423.1	0.0	-	63.00	36.97	46
IRC_IRT_c1371	266	AAT85260.1	0.0	-	95.00	80.49	48
IRC_IRT_c1374	183	XP_003559826.1	0.1	-	52.00	35.81	44
IRC_IRT_c1379	140	XP_002536108.1	0.1	-	63.00	35.04	36
IRC_IRT_c1382	272	KJB45375.1	0.0	-	62.00	45.82	51
IRC_IRT_c1383	103	EAY82190.1	0.0	GO:0020037	100.00	75.10	34
IRC_IRT_c1390	153	AAX95003.1	0.0	GO:0016023	100.00	105.92	4.70E+01
IRC_IRT_c1392	149	AAP53894.1	0.0	GO:0003964	100.00	113.62	49
IRC_IRT_c1394	118	NP_001059755.1	0.0	GO:0016758	100.00	88.20	39

IRC_IRT_c1396	125	BAJ94340.1	0.0	-	86.00	62.00	37
IRC_IRT_c1409	139	XP_001693179.1	0.0	GO:0044763	78.00	57.00	38
IRC_IRT_c1410	123	XP_004253332.2	0.0	-	79.00	59.69	39
IRC_IRT_c1411	141	XP_011014253.1	0.0	GO:0006810	71.00	46.98	45
IRC_IRT_c1415	152	ABR25380.1	0.0	-	100.00	103.22	50
IRC_IRT_c1418	204	NP_050818.1	0.0	-	76.00	37.35	34
IRC_IRT_c1420	105	AHI62962.1	0.0	GO:0003824	70.00	42.36	34
IRC_IRT_c1431	391	EPS70023.1	0.0	-	51.00	41.20	72
IRC_IRT_c1435	149	CAD40418.3	0.0	GO:0008270	82.00	75.87	46
IRC_IRT_c1439	122	XP_010026486.1	0.0	GO:0016787	91.00	75.49	35
IRC_IRT_c1440	194	XP_007029204.1	0.5	-	52.00	33.88	59
IRC_IRT_c1442	606	EPS74511.1	0.0	-	57.00	70.86	85
IRC_IRT_c1446	156	ABF93516.1	0.0	GO:0005739	68.00	66.24	51
IRC_IRT_c1448	114	AII84791.1	0.0	GO:0016021	83.00	55.84	36
IRC_IRT_c1449	164	EEC70906.1	0.0	-	58.00	50.45	51
IRC_IRT_c1451	145	KDO36686.1	0.0	-	58.00	39.66	43
IRC_IRT_c1452	137	XP_002454240.1	0.0	GO:0016874	97.00	72.02	34
IRC_IRT_c1460	107	NP_001046062.1	0.0	-	97.00	71.25	35
IRC_IRT_c1464	148	KEH15665.1	0.0	GO:0006355	82.00	42.74	34
IRC_IRT_c1468	165	XP_002536914.1	0.0	-	90.00	72.79	42
IRC_IRT_c1470	136	XP_004310053.1	0.0	GO:0016151	78.00	63.93	46
IRC_IRT_c1471	232	XP_005648729.1	0.0	GO:0000166	85.00	123.25	74
IRC_IRT_c1472	177	KGN53990.1	0.0	-	73.00	49.68	42
IRC_IRT_c1473	117	NP_001182860.1	0.0	-	81.00	55.07	3.80E+01
IRC_IRT_c1476	179	XP_006598406.1	0.7	-	47.00	33.11	51
IRC_IRT_c1479	243	XP_002953484.1	0.1	-	58.00	35.81	46
IRC_IRT_c1482	112	BAD67629.1	0.0	-	100.00	74.33	37
IRC_IRT_c1484	248	CDY19671.1	0.0	-	69.00	48.52	42
IRC_IRT_c1487	127	XP_010273111.1	1.0	-	60.00	32.34	41
IRC_IRT_c1489	237	XP_008341498.1	0.6	-	51.00	33.88	52
IRC_IRT_c1495	134	XP_002946605.1	0.0	-	68.00	43.13	44
IRC_IRT_c1497	109	XP_002268463.1	0.0	GO:0005840	77.00	51.60	3.60E+01
IRC_IRT_c1500	125	XP_002955466.1	0.0	GO:0004553	68.00	51.60	41

IRC_IRT_c1507	125	ACN26774.1	0.0	GO:0019253	94.00	61.62	36
IRC_IRT_c1516	263	ABA97388.2	0.0	GO:0004523	96.00	102.45	51
IRC_IRT_c1517	148	XP_005646482.1	0.6	-	61.00	33.11	49
IRC_IRT_c1539	205	CCO18857.1	0.2	-	51.00	34.65	60
IRC_IRT_c1540	140	XP_002987145.1	0.0	GO:0005739	84.00	74.71	45
IRC_IRT_c1545	161	YP_001152206.1	0.0	-	63.00	35.81	44
IRC_IRT_c1550	210	XP_003636121.1	0.0	-	82.00	59.31	39
IRC_IRT_c1559	113	XP_002535893.1	0.0	-	66.00	36.58	36
IRC_IRT_c1570	127	CAN80768.1	0.0	-	75.00	39.66	40
IRC_IRT_c1577	129	XP_002536362.1	0.0	-	66.00	50.45	42
IRC_IRT_c1580	156	EAZ06434.1	0.0	GO:0003676	100.00	113.24	51
IRC_IRT_c1583	139	AAF78755.1	0.0	GO:0006643	100.00	101.29	46
IRC_IRT_c1585	226	BAB61230.1	0.0	GO:0009536	94.00	63.54	35
IRC_IRT_c1588	146	XP_002965338.1	0.4	-	56.00	31.19	41
IRC_IRT_c1593	150	CAD40696.3	0.0	-	95.00	92.43	48
IRC_IRT_c1594	131	XP_001778591.1	0.0	GO:0045893	90.00	78.95	43
IRC_IRT_c1595	222	XP_010058463.1	0.5	-	48.00	33.88	74
IRC_IRT_c1598	315	AAS79740.1	0.0	GO:0004523	98.00	137.89	65
IRC_IRT_c1599	140	XP_010911538.1	0.0	-	76.00	45.44	47
IRC_IRT_c1600	102	YP_009091709.1	0.0	-	73.00	44.28	34
IRC_IRT_c1604	130	KEH15234.1	0.0	GO:0006096	100.00	80.11	42
IRC_IRT_c1614	148	EEC83342.1	0.0	GO:0005737	93.00	51.22	49
IRC_IRT_c1615	112	EEC82199.1	0.0	GO:0004553	100.00	77.41	36
IRC_IRT_c1619	221	ABH10994.1	0.0	GO:0016874	71.00	99.37	73
IRC_IRT_c1621	173	XP_007161040.1	0.0	-	46.00	34.27	60
IRC_IRT_c1622	179	XP_011087402.1	0.4	-	56.00	33.50	50
IRC_IRT_c1628	432	KJB06193.1	0.0	-	97.00	100.91	49
IRC_IRT_c1635	188	ABF97700.1	0.0	-	63.00	51.60	41
IRC_IRT_c1648	119	XP_001695168.1	0.2	-	58.00	34.27	39
IRC_IRT_c1658	106	XP_002536961.1	0.0	-	60.00	38.12	35
IRC_IRT_c1661	125	KIY99960.1	0.0	-	61.00	36.58	39
IRC_IRT_c1664	151	XP_002540406.1	0.0	-	56.00	36.19	53
IRC_IRT_c1665	192	AGV54793.1	0.0	-	68.00	43.51	41

IRC_IRT_c1678	135	KDD72662.1	0.0	GO:0005737	87.00	57.77	40
IRC_IRT_c1682	133	EAZ05241.1	0.0	GO:0008080	100.00	91.28	42
IRC_IRT_c1690	128	XP_002534946.1	0.0	-	68.00	44.67	38
IRC_IRT_c1691	127	KFM24104.1	0.1	-	70.00	34.27	34
IRC_IRT_c1702	226	NP_179501.1	0.0	GO:0005385	56.00	43.51	60
IRC_IRT_c1709	113	XP_009350054.1	0.0	-	91.00	65.86	37
IRC_IRT_c1713	285	EPS74494.1	0.0	-	62.00	36.97	43
IRC_IRT_c1717	120	KEH38046.1	0.5	-	62.00	31.57	35
IRC_IRT_c1725	249	XP_007157256.1	0.0	-	52.00	42.36	76
IRC_IRT_c1734	363	NP_039436.1	0.0	GO:0009507	100.00	217.24	106
IRC_IRT_c1740	294	ABA98134.1	0.0	GO:0004523	94.00	186.81	97
IRC_IRT_c1748	126	XP_003573295.1	0.8	-	61.00	32.34	34
IRC_IRT_c1766	147	BAB33421.1	0.0	-	72.00	49.68	40
IRC_IRT_c1767	211	KEH33422.1	0.0	-	62.00	36.19	35
IRC_IRT_c1768	129	CBI24563.3	0.0	GO:0043169	58.00	41.59	39
IRC_IRT_c1772	204	XP_002488950.1	0.0	-	85.00	69.71	42
IRC_IRT_c1773	132	KFM26447.1	0.0	-	65.00	43.90	41
IRC_IRT_c1778	132	XP_003522503.1	0.0	-	65.00	50.83	43
IRC_IRT_c1784	231	XP_006285797.1	0.0	-	66.00	48.14	42
IRC_IRT_c1788	135	CEF99328.1	0.1	-	57.00	35.42	38
IRC_IRT_c1789	115	ACA21845.1	0.9	-	57.00	31.96	35
IRC_IRT_c1809	262	EAZ41972.1	0.1	-	44.00	35.42	69
IRC_IRT_c1810	109	XP_002535040.1	0.0	GO:0003676	85.00	52.76	35
IRC_IRT_c1812	239	CAH67804.1	0.0	GO:0004523	93.00	113.24	74
IRC_IRT_c1820	136	XP_003081906.1	0.1	-	62.00	35.81	35
IRC_IRT_c1824	123	XP_002505085.1	0.6	-	62.00	32.73	35
IRC_IRT_c1832	152	XP_002534675.1	0.0	GO:0009987	76.00	46.60	38
IRC_IRT_c1833	195	XP_005852100.1	0.0	-	58.00	41.59	48
IRC_IRT_c1835	148	KFK38131.1	0.1	-	67.00	35.81	34
IRC_IRT_c1837	231	EPS74494.1	0.0	GO:0009507	88.00	75.87	42
IRC_IRT_c1856	110	XP_002537519.1	0.0	GO:0016020	79.00	59.69	34
IRC_IRT_c1864	139	XP_002536077.1	0.0	GO:0046872	73.00	47.37	41
IRC_IRT_c1865	178	CAD39362.2	0.0	GO:0003964	98.00	114.01	54

IRC_IRT_c1879	107	XP_002535395.1	0.0	-	65.00	40.05	35
IRC_IRT_c1885	192	EEC76774.1	0.0	GO:0055085	83.00	82.03	55
IRC_IRT_c1887	303	XP_002462027.1	0.7	-	60.00	33.88	38
IRC_IRT_c1892	205	KGN61861.1	0.0	-	60.00	42.74	46
IRC_IRT_c1898	138	ACA04843.1	0.0	-	66.00	40.05	39
IRC_IRT_c1900	204	XP_001690932.1	0.2	-	42.00	35.04	56
IRC_IRT_c1902	291	EPS74533.1	0.0	-	67.00	51.99	43
IRC_IRT_c1907	113	EEC75626.1	0.0	-	97.00	74.71	37
IRC_IRT_c1915	159	XP_002536193.1	0.0	GO:0016020	97.00	98.98	49
IRC_IRT_c1925	156	XP_005851693.1	0.1	-	64.00	35.04	37
IRC_IRT_c1931	150	KIY99572.1	0.0	-	54.00	37.74	46
IRC_IRT_c1933	122	EEE65846.1	0.0	GO:0006468	100.00	94.36	40
IRC_IRT_c1940	141	BAH19674.1	0.0	GO:0009750	71.00	45.82	39
IRC_IRT_c1944	134	XP_002538475.1	0.0	GO:0015419	82.00	51.22	34
IRC_IRT_c1966	160	XP_009761859.1	0.0	GO:0005975	72.00	62.39	48
IRC_IRT_c1968	123	XP_002949131.1	0.0	-	68.00	37.74	35
IRC_IRT_c1974	151	XP_001693179.1	0.0	GO:0044763	78.00	58.54	38
IRC_IRT_c1981	105	KFM27119.1	0.0	-	67.00	39.66	34
IRC_IRT_c1982	124	XP_010440705.1	0.0	GO:0009941	83.00	61.23	37
IRC_IRT_c1984	190	EPS70027.1	0.0	-	64.00	38.89	34
IRC_IRT_c1994	227	XP_002538388.1	0.0	-	64.00	45.05	57
IRC_IRT_c1995	105	XP_002537101.1	0.0	-	67.00	37.35	34
IRC_IRT_c1999	137	EEC71158.1	0.0	GO:0016023	100.00	97.83	45
IRC_IRT_c2000	283	XP_008811020.1	0.2	-	52.00	36.19	57
IRC_IRT_c2003	131	XP_006650036.1	0.1	-	62.00	35.42	35
IRC_IRT_c2009	145	XP_006651228.1	0.1	-	65.00	35.42	35
IRC_IRT_c2010	318	AGC78878.1	0.0	-	77.00	60.08	48
IRC_IRT_c2011	131	XP_002973258.1	0.0	-	55.00	38.89	43
IRC_IRT_c2013	199	KEH17697.1	0.0	-	82.00	46.21	35
IRC_IRT_c2014	124	BAD21935.1	0.0	-	63.00	40.05	41
IRC_IRT_c2029	121	XP_009759241.1	0.0	GO:0000166	70.00	44.67	40
IRC_IRT_c2034	113	EEC79491.1	0.0	GO:0050660	74.00	51.60	35
IRC_IRT_c2035	233	KJB31094.1	0.0	-	72.00	44.67	36

IRC_IRT_c2037	156	BAK02440.1	0.0	GO:0020037	84.00	77.03	46
IRC_IRT_c2041	144	EEE67873.1	0.0	GO:0003824	75.00	48.91	36
IRC_IRT_c2048	168	CDX80916.1	0.8	-	59.00	33.11	42
IRC_IRT_c2051	112	XP_002501855.1	0.0	-	61.00	38.51	34
IRC_IRT_c2054	149	XP_006380095.1	0.0	-	63.00	58.54	58
IRC_IRT_c2056	131	XP_002535088.1	0.0	-	71.00	34.65	35
IRC_IRT_c2062	116	EAY86719.1	0.0	GO:0016023	100.00	82.42	38
IRC_IRT_c2067	180	KDP20490.1	1.0	-	45.00	31.96	42
IRC_IRT_c2069	121	XP_002535540.1	0.0	-	68.00	38.89	35
IRC_IRT_c2080	115	BAD94287.1	0.0	-	86.00	36.58	36
IRC_IRT_c2096	126	XP_002535235.1	0.0	-	67.00	43.51	40
IRC_IRT_c2105	108	XP_002536541.1	0.4	-	62.00	32.73	35
IRC_IRT_c2109	118	XP_001781297.1	0.0	GO:0051213	76.00	53.91	38
IRC_IRT_c2112	168	EXC22243.1	0.0	-	75.00	50.45	36
IRC_IRT_c2115	176	XP_005646157.1	0.0	GO:0016740	60.00	35.81	41
IRC_IRT_c2118	165	EEC76122.1	0.0	GO:0044763	68.00	56.23	54
IRC_IRT_c2125	139	XP_002994587.1	0.0	-	82.00	68.55	39
IRC_IRT_c2136	121	NP_001068332.1	0.0	GO:0016021	100.00	77.41	36
IRC_IRT_c2138	170	CCO17359.1	0.0	GO:0008750	91.00	103.22	56
IRC_IRT_c2140	139	EEE55977.1	0.1	-	58.00	35.81	39
IRC_IRT_c2145	131	XP_002535669.1	0.0	-	67.00	52.76	43
IRC_IRT_c2152	265	EAY79110.1	0.0	-	96.00	122.87	59
IRC_IRT_c2161	166	XP_002538135.1	0.0	-	73.00	51.99	49
IRC_IRT_c2163	184	XP_002537036.1	0.0	GO:0044699	59.00	44.28	49
IRC_IRT_c2167	219	KEH31803.1	0.6	-	51.00	31.57	45
IRC_IRT_c2176	128	CBI23565.3	0.0	GO:0034976	76.00	43.51	42
IRC_IRT_c2179	126	EEC77111.1	0.1	-	62.00	36.19	35
IRC_IRT_c2182	180	KFM28754.1	0.5	-	52.00	33.88	44
IRC_IRT_c2184	156	XP_002536557.1	0.0	-	75.00	39.28	36
IRC_IRT_c2190	120	XP_001694009.1	0.2	-	61.00	34.65	39
IRC_IRT_c2199	138	AAL67586.1	0.0	GO:0004523	100.00	107.84	46
IRC_IRT_c2204	105	P53385.1	0.0	GO:0019557	97.00	70.48	34
IRC_IRT_c2208	178	EEC76877.1	0.0	GO:0046961	94.00	95.90	53

IRC_IRT_c2209	157	XP_002536181.1	0.0	-	55.00	38.51	52
IRC_IRT_c2216	194	XP_011015413.1	0.0	GO:0003677	77.00	77.03	54
IRC_IRT_c2219	151	XP_011016759.1	0.4	-	61.00	33.50	34
IRC_IRT_c2220	147	ABI54732.1	0.0	GO:0046961	93.00	85.11	48
IRC_IRT_c2230	128	XP_003600046.1	0.0	GO:0004834	82.00	56.61	39
IRC_IRT_c2259	114	XP_011016603.1	0.0	GO:0055114	100.00	76.26	37
IRC_IRT_c2266	127	KIY95072.1	0.0	GO:0003824	78.00	59.31	42
IRC_IRT_c2285	135	XP_002538221.1	0.0	-	59.00	39.28	44
IRC_IRT_c2290	131	XP_002540575.1	0.0	-	73.00	53.14	41
IRC_IRT_c2291	158	XP_010506684.1	0.7	-	58.00	33.11	46
IRC_IRT_c2293	169	XP_002538039.1	0.0	-	75.00	53.91	45
IRC_IRT_c2296	139	XP_002960004.1	0.0	GO:0046872	79.00	65.08	44
IRC_IRT_c2301	162	AJM90101.1	0.0	GO:0046961	87.00	81.26	48
IRC_IRT_c2310	148	EEE67517.1	0.0	-	97.00	78.57	41
IRC_IRT_c2314	153	XP_010244809.1	0.6	-	52.00	33.11	36
IRC_IRT_c2318	175	XP_002536189.1	0.0	GO:0051287	79.00	91.28	58
IRC_IRT_c2331	191	XP_003614393.1	0.0	-	65.00	51.99	49
IRC_IRT_c2336	176	XP_003075267.1	0.0	GO:0003824	65.00	53.53	46
IRC_IRT_c2337	108	XP_002535713.1	0.0	-	62.00	41.20	35
IRC_IRT_c2342	144	XP_003057148.1	0.0	GO:0070526	79.00	41.20	43
IRC_IRT_c2353	401	AAM93446.1	0.0	GO:0044260	88.00	89.74	44
IRC_IRT_c2358	270	CAN62678.1	0.2	-	54.00	35.81	35
IRC_IRT_c2359	184	ERN19185.1	0.0	-	62.00	37.35	35
IRC_IRT_c2367	129	XP_008799904.1	0.8	-	51.00	31.96	39
IRC_IRT_c2371	129	XP_011014724.1	0.0	GO:0016757	78.00	50.45	41
IRC_IRT_c2378	116	XP_009350809.1	0.0	-	89.00	66.24	37
IRC_IRT_c2380	168	XP_002538475.1	0.0	GO:0044763	67.00	55.45	53
IRC_IRT_c2381	218	KDP20463.1	0.0	-	97.00	63.93	34
IRC_IRT_c2384	197	XP_007050066.1	0.8	-	56.00	33.11	44
IRC_IRT_c2385	323	BAJ11784.1	0.0	-	44.00	53.53	112
IRC_IRT_c2389	148	ABK23597.1	0.0	GO:0044763	68.00	49.29	44
IRC_IRT_c2396	175	ABA94084.1	0.0	GO:0004523	100.00	72.02	34
IRC_IRT_c2397	151	XP_002467304.1	0.0	-	92.00	66.63	38

IRC_IRT_c2404	161	AAU10741.1	0.0	GO:0004523	100.00	83.57	39
IRC_IRT_c2406	116	XP_004249039.1	0.8	-	52.00	31.96	38
IRC_IRT_c2408	129	XP_009125545.1	0.0	GO:0006261	80.00	50.83	36
IRC_IRT_c2411	161	XP_010911304.1	0.0	GO:0050896	75.00	48.91	41
IRC_IRT_c2414	191	XP_002535741.1	0.0	GO:0007165	75.00	58.54	49
IRC_IRT_c2426	124	XP_001697294.1	0.0	GO:0006098	73.00	57.77	42
IRC_IRT_c2430	134	XP_009350814.1	0.0	-	59.00	40.43	44
IRC_IRT_c2442	198	BAH79998.1	0.0	-	93.00	85.89	45
IRC_IRT_c2447	121	XP_001772189.1	0.9	-	47.00	32.73	36
IRC_IRT_c2465	143	XP_002536465.1	0.4	-	58.00	32.34	34
IRC_IRT_c2470	106	BAD35462.1	0.0	GO:0008270	100.00	79.72	3.50E+01
IRC_IRT_c2495	174	XP_010025537.1	0.0	-	64.00	53.14	57
IRC_IRT_c2497	330	BAJ11784.1	0.3	-	57.00	35.04	47
IRC_IRT_c2498	266	AAV25653.1	0.0	GO:0008270	94.00	61.62	34
IRC_IRT_c2514	149	XP_002539210.1	0.0	-	84.00	47.75	38
IRC_IRT_c2518	107	XP_001786560.1	0.0	GO:0006289	85.00	53.14	35
IRC_IRT_c2528	121	XP_003636120.1	0.3	-	63.00	33.88	36
IRC_IRT_c2530	309	XP_003607358.1	0.0	-	54.00	37.74	53
IRC_IRT_c2532	370	YP_358636.1	0.0	GO:0009536	73.00	47.37	34
IRC_IRT_c2539	111	EAY99093.1	0.0	GO:0003883	100.00	79.34	36
IRC_IRT_c2541	136	XP_008813562.1	0.0	GO:0005739	73.00	58.15	45
IRC_IRT_c2542	138	YP_009091701.1	0.0	GO:0005739	97.00	92.82	45
IRC_IRT_c2543	157	XP_008234621.1	0.0	-	67.00	62.77	52
IRC_IRT_c2547	242	CDY63598.1	0.0	GO:0009536	76.00	60.08	47
IRC_IRT_c2548	185	XP_008651517.1	0.3	-	53.00	34.27	41
IRC_IRT_c2569	150	XP_001703522.1	0.0	-	58.00	38.89	48
IRC_IRT_c2575	140	XP_002539885.1	0.0	GO:0016740	68.00	40.82	44
IRC_IRT_c2579	155	XP_001690070.1	0.0	GO:0003824	67.00	42.36	37
IRC_IRT_c2586	138	BAD53153.1	0.0	-	58.00	39.28	46
IRC_IRT_c2587	117	XP_002538617.1	0.4	-	69.00	33.11	36
IRC_IRT_c2592	120	EEC80863.1	0.0	GO:0015408	83.00	59.69	37
IRC_IRT_c2593	390	CDP03265.1	0.4	-	58.00	35.04	39
IRC_IRT_c2602	197	EHK62689.1	0.0	GO:0043565	87.00	69.71	40

IRC_IRT_c2604	104	XP_002535367.1	0.0	-	70.00	42.74	34
IRC_IRT_c2613	104	XP_002540087.1	0.0	GO:0006865	79.00	40.05	34
IRC_IRT_c2619	165	BAK00396.1	0.0	GO:0019752	75.00	71.63	54
IRC_IRT_c2620	109	XP_011014727.1	0.0	GO:0005737	88.00	62.00	36
IRC_IRT_c2621	102	XP_002538106.1	0.0	GO:0044763	73.00	47.75	34
IRC_IRT_c2626	156	CAN73785.1	0.0	GO:0051287	80.00	74.71	52
IRC_IRT_c2630	118	XP_002952597.1	0.0	GO:0071704	72.00	46.21	37
IRC_IRT_c2631	178	KEH17019.1	0.0	-	80.00	48.91	36
IRC_IRT_c2633	130	XP_001784004.1	0.0	GO:0009536	77.00	64.70	40
IRC_IRT_c2636	107	XP_004489445.1	0.0	-	82.00	42.74	34
IRC_IRT_c2641	130	EEC75898.1	0.0	GO:0016301	100.00	80.11	37
IRC_IRT_c2652	127	DAA05106.1	0.0	GO:0006355	92.00	41.59	40
IRC_IRT_c2655	128	XP_009386211.1	0.0	-	70.00	39.66	34
IRC_IRT_c2660	165	EPS69883.1	0.7	-	64.00	33.11	42
IRC_IRT_c2661	179	AAU89172.1	0.0	GO:0003964	100.00	118.24	52
IRC_IRT_c2664	112	XP_002539661.1	0.0	-	75.00	51.22	37
IRC_IRT_c2665	114	ABA98437.1	0.1	-	58.00	35.42	41
IRC_IRT_c2674	148	NP_001169545.1	0.0	GO:0009536	73.00	58.92	5.30E+01
IRC_IRT_c2684	139	XP_002538056.1	0.0	-	63.00	44.28	46
IRC_IRT_c2690	210	EAZ00158.1	0.0	GO:1901701	69.00	88.97	66
IRC_IRT_c2692	132	XP_010248518.1	0.0	-	62.00	45.82	43
IRC_IRT_c2694	217	YP_009033840.1	0.7	-	44.00	33.50	61
IRC_IRT_c2707	102	CAD39341.2	0.0	GO:0004523	97.00	74.71	34
IRC_IRT_c2708	172	XP_009350076.1	0.0	GO:0034660	96.00	100.14	53
IRC_IRT_c2709	177	CAE02875.1	0.0	GO:0004185	100.00	99.37	58
IRC_IRT_c2716	148	KEH22973.1	0.0	-	51.00	36.58	49
IRC_IRT_c2717	174	XP_002517067.1	0.0	GO:0016491	72.00	53.53	55
IRC_IRT_c2718	374	BAD22424.1	0.0	-	96.00	157.92	83
IRC_IRT_c2721	161	XP_002539394.1	0.4	-	51.00	32.73	47
IRC_IRT_c2723	138	XP_002875070.1	0.6	-	61.00	33.11	34
IRC_IRT_c2726	154	XP_002536362.1	0.0	GO:0004872	84.00	79.34	51
IRC_IRT_c2735	148	XP_004960700.1	0.0	GO:0004316	70.00	49.29	50
IRC_IRT_c2742	175	XP_002946025.1	0.0	GO:0006810	71.00	45.82	42

IRC_IRT_c2744	115	XP_001763508.1	0.0	GO:0016740	70.00	41.97	37
IRC_IRT_c2751	130	CAE01289.2	0.0	GO:0031425	90.00	77.41	43
IRC_IRT_c2755	129	AFW62781.1	0.7	-	48.00	30.80	39
IRC_IRT_c2758	223	XP_002539098.1	0.0	-	72.00	53.14	43
IRC_IRT_c2762	123	XP_002538274.1	0.0	-	67.00	42.36	34
IRC_IRT_c2767	103	AIA86057.1	1.0	-	58.00	30.42	39
IRC_IRT_c2773	132	AJB98438.1	0.0	GO:0009058	86.00	66.63	44
IRC_IRT_c2793	123	EEC77110.1	0.0	GO:0016491	84.00	57.00	39
IRC_IRT_c2800	207	XP_002539738.1	0.0	-	45.00	38.12	72
IRC_IRT_c2804	155	XP_008450755.1	0.0	-	63.00	38.12	36
IRC_IRT_c2808	277	KJB31094.1	0.0	-	66.00	43.13	36
IRC_IRT_c2810	162	BAB33421.1	0.0	-	62.00	48.14	48
IRC_IRT_c2834	139	XP_006837289.1	0.0	GO:0005829	62.00	47.37	45
IRC_IRT_c2856	167	XP_002987092.1	0.0	-	75.00	40.05	36
IRC_IRT_c2859	122	XP_009143298.1	0.7	-	69.00	32.73	36
IRC_IRT_c2863	121	BAK02440.1	0.0	GO:0020037	89.00	55.07	37
IRC_IRT_c2871	119	XP_003590573.1	0.0	-	73.00	38.12	34
IRC_IRT_c2876	149	XP_009350067.1	0.0	GO:0050896	76.00	48.52	42
IRC_IRT_c2885	190	XP_002538475.1	0.0	GO:0015419	87.00	66.24	40
IRC_IRT_c2908	158	ABR18115.1	0.0	GO:0046034	73.00	68.94	52
IRC_IRT_c2920	134	KJB23014.1	0.5	-	62.00	33.11	35
IRC_IRT_c2924	143	AGC78890.1	0.0	GO:0005739	97.00	96.29	47
IRC_IRT_c2934	116	EEC77111.1	1.0	-	75.00	32.34	37
IRC_IRT_c2956	124	NP_001173179.1	0.0	GO:0005829	97.00	68.17	36
IRC_IRT_c2961	176	XP_001784121.1	0.0	GO:0051537	97.00	74.71	39
IRC_IRT_c2986	124	XP_002537175.1	0.0	-	70.00	36.58	34
IRC_IRT_c2991	109	XP_004963701.1	0.0	-	73.00	43.90	34
IRC_IRT_c2992	346	KJB44141.1	0.1	-	61.00	32.73	36
IRC_IRT_c2996	108	EMT00244.1	0.0	-	71.00	37.74	35
IRC_IRT_c2999	139	YP_665666.1	0.0	GO:0048038	80.00	73.94	46
IRC_IRT_c3002	149	KDP20463.1	0.0	-	86.00	58.15	37
IRC_IRT_c3016	127	EEC81325.1	0.0	GO:0006810	76.00	55.84	38
IRC_IRT_c3018	212	KEH41122.1	0.9	-	57.00	33.11	35

IRC_IRT_c3022	121	XP_004290087.1	0.8	-	64.00	32.73	37
IRC_IRT_c3040	193	XP_009388207.1	0.0	-	66.00	45.44	39
IRC_IRT_c3061	138	EEC83292.1	0.0	GO:0016020	78.00	62.39	46
IRC_IRT_c3066	129	AAP53968.1	0.0	GO:0003677	97.00	88.97	42
IRC_IRT_c3077	102	XP_005645099.1	0.0	-	76.00	48.91	34
IRC_IRT_c3086	161	EDQ48453.1	0.6	-	52.00	33.11	46
IRC_IRT_c3095	136	XP_002512017.1	0.0	-	67.00	40.43	34
IRC_IRT_c3098	114	BAD94287.1	0.0	GO:0005829	91.00	63.54	36
IRC_IRT_c3099	146	XP_002965719.1	0.0	GO:0070011	82.00	76.64	46
IRC_IRT_c3100	111	EEE62721.1	0.0	GO:0016788	100.00	80.49	37
IRC_IRT_c3116	125	Q41346.1	0.0	-	64.00	37.74	37
IRC_IRT_c3118	138	EEC83775.1	0.0	-	81.00	71.25	44
IRC_IRT_c3122	110	XP_009351044.1	0.3	-	62.00	33.88	35
IRC_IRT_c3126	124	XP_002960012.1	1.0	-	67.00	31.57	34
IRC_IRT_c3135	217	BAC84749.1	0.0	GO:0005739	77.00	98.98	66
IRC_IRT_c3139	122	CCO17247.1	0.0	-	63.00	37.35	36
IRC_IRT_c3144	280	XP_010503434.1	0.0	GO:0005739	79.00	95.90	63
IRC_IRT_c3146	135	XP_003062093.1	0.5	-	65.00	33.11	38
IRC_IRT_c3158	214	YP_358637.1	0.0	GO:0009536	74.00	48.52	39
IRC_IRT_c3164	166	ACF81592.1	0.0	GO:0003746	100.00	100.14	48
IRC_IRT_c3181	130	XP_010233723.1	0.6	-	47.00	33.11	48
IRC_IRT_c3182	167	AAC37341.1	0.0	GO:0005507	73.00	63.16	46
IRC_IRT_c3199	121	XP_002535839.1	0.0	GO:0042886	75.00	41.97	40
IRC_IRT_c3202	120	KEH17345.1	0.1	-	60.00	33.88	41
IRC_IRT_c3205	115	AFD33354.1	0.0	-	71.00	47.75	35
IRC_IRT_c3207	310	XP_007009890.1	0.8	-	56.00	32.34	41
IRC_IRT_c3209	512	ABA97503.2	0.0	GO:0003964	92.00	174.10	90
IRC_IRT_c3210	252	BAD69364.1	0.0	-	89.00	83.19	49
IRC_IRT_c3213	158	KJB44141.1	0.0	-	64.00	35.42	34
IRC_IRT_c3214	178	AAP06924.1	0.0	-	70.00	39.66	34
IRC_IRT_c3220	214	XP_003614389.1	0.0	-	71.00	54.30	46
IRC_IRT_c3229	210	CAE05461.1	0.0	GO:0005739	95.00	115.16	61
IRC_IRT_c3238	302	AAX95721.1	0.0	GO:0004523	99.00	209.92	100

IRC_IRT_c3243	227	AAM01048.1	0.0	-	71.00	84.34	78
IRC_IRT_c3244	152	XP_003636119.1	0.0	-	72.00	39.66	36
IRC_IRT_c3245	246	BAD10089.1	0.0	GO:0005739	94.00	125.95	67
IRC_IRT_c3246	201	CAI44627.1	0.0	GO:0044260	74.00	55.84	47
IRC_IRT_c3248	444	ABA97917.2	0.0	GO:0003676	100.00	134.81	60
IRC_IRT_c3252	160	ABH09321.1	0.0	-	65.00	43.13	38
IRC_IRT_c3255	242	CAH66289.1	0.0	GO:0003964	97.00	133.65	80
IRC_IRT_c3256	177	KJB31094.1	0.1	-	63.00	33.11	36
IRC_IRT_c3258	224	KJB31094.1	0.0	-	72.00	65.86	48
IRC_IRT_c3259	121	XP_004505235.1	0.3	-	50.00	33.88	38
IRC_IRT_c3263	212	AAU43943.1	0.0	-	85.00	103.99	61
IRC_IRT_c3264	115	CAJ80989.1	0.0	-	69.00	41.97	39
IRC_IRT_c3265	153	AAQ56335.1	0.0	GO:0004523	100.00	78.95	34
IRC_IRT_c3267	201	EYU24190.1	0.0	-	52.00	39.28	57
IRC_IRT_c3268	168	XP_002961274.1	0.0	GO:0008152	69.00	57.00	42
IRC_IRT_c3270	163	AGZ19352.1	0.0	-	65.00	38.89	40
IRC_IRT_c3272	143	XP_002500781.1	0.0	GO:0044710	66.00	51.60	45
IRC_IRT_c3273	338	BAD68068.1	0.0	GO:0003676	89.00	150.98	87
IRC_IRT_c3274	587	CAH67937.1	0.0	GO:0003964	98.00	360.15	179
IRC_IRT_c3275	267	AAX94870.1	0.0	GO:0004523	100.00	192.20	89
IRC_IRT_c3280	438	BAA85414.1	0.0	GO:0003676	91.00	110.15	58
IRC_IRT_c3281	202	NP_001173078.1	0.0	GO:0006259	90.00	121.71	63
IRC_IRT_c3285	234	XP_002488950.1	0.0	-	94.00	71.63	36
IRC_IRT_c3286	175	XP_004955743.1	0.9	-	54.00	32.34	35
IRC_IRT_c3289	170	EEC67124.1	0.0	GO:0006779	78.00	67.40	46
IRC_IRT_c3293	106	XP_002535868.1	0.9	-	61.00	31.96	34
IRC_IRT_c3297	147	XP_002534940.1	0.0	GO:0016810	72.00	41.97	37
IRC_IRT_c3299	171	CDO97356.1	0.0	GO:0005618	84.00	87.81	57
IRC_IRT_c3300	166	ABA95048.1	0.0	GO:0003676	98.00	118.24	55
IRC_IRT_c3304	119	KDO39226.1	0.0	-	42.00	42.36	68
IRC_IRT_c3309	132	BAC84120.1	0.0	-	67.00	47.37	43
IRC_IRT_c3323	107	ABC24948.1	0.0	GO:0005737	80.00	53.53	35
IRC_IRT_c3327	279	BAC83293.1	0.0	GO:0004523	90.00	170.24	93

IRC_IRT_c3332	136	XP_002534684.1	0.0	-	58.00	41.20	43
IRC_IRT_c3336	117	XP_002502546.1	0.0	GO:0044238	65.00	42.36	49
IRC_IRT_c3349	201	AES98804.2	0.3	-	54.00	34.65	62
IRC_IRT_c3353	337	AAT47050.1	0.0	GO:0051287	97.00	207.99	103
IRC_IRT_c3354	103	XP_005646017.1	0.1	-	61.00	34.27	34
IRC_IRT_c3358	387	ABF95990.1	0.0	GO:0003964	98.00	206.45	115
IRC_IRT_c3361	137	XP_002500355.1	0.0	-	71.00	38.51	35
IRC_IRT_c3368	374	XP_010031483.1	0.0	GO:0009570	68.00	93.59	89
IRC_IRT_c3374	126	ADC80652.1	0.0	GO:0050896	73.00	51.99	41
IRC_IRT_c3383	108	XP_006652240.1	0.0	-	70.00	40.43	34
IRC_IRT_c3393	267	CAE54548.1	0.0	GO:0003964	95.00	105.92	71
IRC_IRT_c3404	231	AAK92562.1	0.0	GO:0005739	87.00	85.50	54
IRC_IRT_c3406	164	XP_006370384.1	0.0	GO:0016620	69.00	62.00	55
IRC_IRT_c3409	142	CCO20210.1	0.0	-	68.00	40.82	44
IRC_IRT_c3417	246	XP_005848554.1	0.1	-	50.00	30.03	50
IRC_IRT_c3419	119	XP_002539738.1	0.0	GO:0050794	72.00	40.82	36
IRC_IRT_c3422	142	XP_002538221.1	0.0	-	59.00	40.43	42
IRC_IRT_c3423	121	AAK98730.1	0.0	GO:0004553	97.00	80.11	39
IRC_IRT_c3427	222	XP_002534642.1	0.0	GO:0015031	80.00	100.91	71
IRC_IRT_c3432	215	CAE05462.2	0.0	GO:0003677	92.00	80.49	42
IRC_IRT_c3434	211	KEH17021.1	0.0	-	43.00	46.98	87
IRC_IRT_c3435	167	XP_003635968.1	0.0	GO:0017111	72.00	57.00	50
IRC_IRT_c3437	213	BAC83659.1	0.0	GO:0009536	92.00	73.17	52
IRC_IRT_c3443	116	XP_006295245.1	0.0	GO:0006633	75.00	43.90	37
IRC_IRT_c3446	118	AGG09511.1	0.0	GO:0003899	94.00	71.25	38
IRC_IRT_c3447	146	BAC15779.1	0.0	GO:0005739	93.00	41.97	43
IRC_IRT_c3451	188	NP_001051816.1	0.0	GO:0003677	98.00	124.79	62
IRC_IRT_c3454	176	KFM28844.1	0.0	GO:0046872	86.00	97.06	58
IRC_IRT_c3455	103	EAY89254.1	0.0	GO:0005739	71.00	39.28	35
IRC_IRT_c3469	316	XP_003057253.1	0.0	GO:0005840	71.00	60.85	53
IRC_IRT_c3473	137	XP_002535727.1	0.0	-	57.00	37.74	45
IRC_IRT_c3474	163	AAR01692.1	0.0	GO:0003676	95.00	93.59	46
IRC_IRT_c3476	108	BAD33754.1	0.0	GO:0051287	100.00	83.57	35

IRC_IRT_c3481	132	XP_005847699.1	0.0	-	63.00	38.89	44
IRC_IRT_c3482	125	ABA93648.1	0.0	GO:0004523	97.00	78.18	38
IRC_IRT_c3486	295	XP_003588326.1	0.0	-	58.00	39.66	50
IRC_IRT_c3488	182	AGB85039.1	0.0	-	74.00	43.51	35
IRC_IRT_c3493	130	XP_001420369.1	0.0	GO:0016668	73.00	44.28	41
IRC_IRT_c3505	132	XP_009350805.1	0.0	GO:0006399	78.00	68.17	42
IRC_IRT_c3508	202	CAE04376.1	0.0	GO:0004523	100.00	124.79	60
IRC_IRT_c3510	190	XP_006418762.1	0.0	-	52.00	37.35	61
IRC_IRT_c3511	303	ABF95975.1	0.0	GO:0003964	97.00	201.06	100
IRC_IRT_c3514	276	CAH67707.1	0.0	GO:0003676	100.00	46.60	56
IRC_IRT_c3516	134	XP_002539604.1	0.3	-	78.00	32.34	37
IRC_IRT_c3517	126	NP_001176905.1	0.0	GO:0016021	100.00	89.74	42
IRC_IRT_c3518	114	EMT23941.1	0.0	GO:0005975	67.00	41.20	37
IRC_IRT_c3535	142	YP_764400.1	0.0	GO:0044444	69.00	40.43	42
IRC_IRT_c3536	156	AAT68205.1	0.0	GO:0045252	82.00	73.56	46
IRC_IRT_c3560	109	XP_008792573.1	0.0	GO:0003887	88.00	53.14	36
IRC_IRT_c3562	157	XP_007162158.1	0.4	-	50.00	33.88	52
IRC_IRT_c3564	141	XP_002959991.1	0.0	-	62.00	37.74	37
IRC_IRT_c3566	142	AAN04943.1	0.0	GO:0003964	100.00	85.50	38
IRC_IRT_c3571	157	XP_002536074.1	0.0	GO:0008697	80.00	73.94	52
IRC_IRT_c3574	172	XP_002536861.1	0.0	GO:0006355	80.00	67.40	57
IRC_IRT_c3576	148	XP_002468270.1	0.1	-	63.00	35.42	36
IRC_IRT_c3577	142	XP_010458061.1	0.9	-	50.00	32.34	40
IRC_IRT_c3583	102	XP_002539605.1	0.0	-	64.00	39.66	34
IRC_IRT_c3588	118	XP_005846214.1	0.0	-	60.00	38.12	35
IRC_IRT_c3589	108	KFM24206.1	0.0	GO:0005737	88.00	62.00	35
IRC_IRT_c3593	154	DAA52475.1	0.0	-	69.00	44.67	36
IRC_IRT_c3595	157	BAD87500.1	0.0	GO:0003676	77.00	66.63	49
IRC_IRT_c3601	186	CAH66288.1	0.0	GO:0003964	100.00	130.18	61
IRC_IRT_c3606	184	ABG66296.1	0.0	GO:0004523	94.00	75.49	39
IRC_IRT_c3615	214	KJB09764.1	0.0	-	65.00	41.20	35
IRC_IRT_c3617	130	XP_001775654.1	0.0	-	69.00	36.97	39
IRC_IRT_c3619	126	XP_009351102.1	0.0	GO:0009536	73.00	61.23	41

IRC_IRT_c3624	149	XP_011016741.1	0.0	-	73.00	55.45	42
IRC_IRT_c3629	107	EEC66960.1	0.0	GO:0006810	88.00	55.84	35
IRC_IRT_c3639	147	XP_009351044.1	0.0	-	67.00	49.68	46
IRC_IRT_c3651	148	AAN05397.1	0.2	-	51.00	34.65	43
IRC_IRT_c3661	160	BAJ89898.1	0.9	-	58.00	32.73	34
IRC_IRT_c3665	116	XP_002539670.1	0.0	-	83.00	66.63	37
IRC_IRT_c3667	144	EEC70905.1	0.0	-	82.00	40.05	46
IRC_IRT_c3670	157	XP_001778187.1	0.0	-	65.00	45.82	55
IRC_IRT_c3676	116	YP_002600845.1	0.0	GO:0006412	92.00	65.08	38
IRC_IRT_c3683	176	XP_002539100.1	0.0	GO:0016747	78.00	73.94	55
IRC_IRT_c3685	135	EEC81324.1	0.0	-	56.00	40.43	41
IRC_IRT_c3687	137	XP_006853174.1	0.0	GO:0009084	65.00	45.05	43
IRC_IRT_c3693	119	AAM08507.1	0.0	GO:0008152	97.00	79.72	3.90E+01
IRC_IRT_c3695	178	KEH22088.1	0.0	-	64.00	64.31	56
IRC_IRT_c3698	122	EMT33748.1	0.6	-	60.00	33.11	35
IRC_IRT_c3711	110	XP_002536196.1	0.1	-	64.00	35.42	34
IRC_IRT_c3728	140	CAE02127.2	0.0	GO:0004523	97.00	82.03	37
IRC_IRT_c3738	141	EAZ00597.1	0.0	GO:0005739	97.00	90.51	46
IRC_IRT_c3740	110	XP_009611727.1	0.1	-	63.00	35.04	36
IRC_IRT_c3747	158	BAC84716.1	0.0	GO:0003676	86.00	73.56	43
IRC_IRT_c3753	154	XP_005843402.1	0.0	GO:0016491	70.00	52.37	50
IRC_IRT_c3767	141	XP_003617283.1	0.0	-	67.00	37.35	43
IRC_IRT_c3769	129	XP_002537545.1	0.1	-	57.00	34.65	35
IRC_IRT_c3780	135	KFM27378.1	0.0	-	77.00	46.60	35
IRC_IRT_c3781	182	ABA98740.2	0.0	GO:0005739	76.00	73.56	47
IRC_IRT_c3785	156	CAO02550.1	0.0	-	74.00	38.12	35
IRC_IRT_c3787	134	XP_002539786.1	0.0	-	75.00	50.45	37
IRC_IRT_c3789	140	XP_011016603.1	0.0	-	78.00	39.66	46
IRC_IRT_c3796	103	CDY16584.1	0.0	-	62.00	39.66	35
IRC_IRT_c3800	197	ABA98657.1	0.0	GO:0016023	100.00	78.57	35
IRC_IRT_c3801	145	BAD29459.1	0.0	-	97.00	63.93	48
IRC_IRT_c3803	131	XP_002507650.1	0.6	-	57.00	33.11	40
IRC_IRT_c3804	146	EEE65440.1	0.0	GO:0005234	87.00	73.94	48

IRC_IRT_c3805	128	XP_002539886.1	0.0	GO:0004061	86.00	55.84	36
IRC_IRT_c3809	154	XP_011074972.1	0.0	-	43.00	39.28	57
IRC_IRT_c3813	141	XP_002535552.1	0.9	-	71.00	32.73	38
IRC_IRT_c3824	116	XP_001691660.1	0.0	GO:0044763	65.00	44.28	38
IRC_IRT_c3829	179	XP_006405922.1	0.0	-	76.00	58.15	43
IRC_IRT_c3833	175	XP_008789070.1	0.1	-	54.00	36.58	44
IRC_IRT_c3845	121	XP_002536043.1	0.0	-	65.00	41.97	40
IRC_IRT_c3848	209	XP_004493566.1	0.9	-	55.00	33.11	52
IRC_IRT_c3851	111	XP_001786616.1	0.4	-	63.00	32.73	36
IRC_IRT_c3852	106	XP_005848651.1	0.0	GO:0008270	80.00	50.83	35
IRC_IRT_c3854	131	EEE52320.1	0.0	GO:0019752	72.00	43.90	40
IRC_IRT_c3855	141	YP_002600860.1	0.8	-	60.00	31.96	38
IRC_IRT_c3866	240	XP_002534687.1	0.0	GO:0008137	91.00	86.27	47
IRC_IRT_c3872	140	XP_002536228.1	0.0	-	73.00	48.14	46
IRC_IRT_c3875	113	XP_001699611.1	0.0	-	72.00	36.58	37
IRC_IRT_c3887	221	XP_008449387.1	0.0	-	82.00	53.53	35
IRC_IRT_c3899	211	EEE50470.1	0.0	GO:0006139	75.00	42.74	37
IRC_IRT_c3914	110	XP_010906713.1	0.3	-	61.00	33.50	36
IRC_IRT_c3922	110	CAA53993.1	0.0	GO:0044763	77.00	43.13	36
IRC_IRT_c3923	140	AAT93989.1	0.0	-	84.00	65.86	38
IRC_IRT_c3925	141	XP_001786703.1	0.0	GO:0016301	72.00	47.37	40
IRC_IRT_c3927	106	CAE02229.2	0.0	GO:0003676	82.00	60.08	34
IRC_IRT_c3931	176	CCO16157.1	0.0	GO:0070011	84.00	80.88	50
IRC_IRT_c3935	113	XP_002534890.1	0.0	GO:0015079	83.00	41.97	37
IRC_IRT_c3938	201	XP_002538121.1	0.0	-	64.00	46.21	42
IRC_IRT_c3939	143	KIZ02220.1	0.0	GO:0044260	68.00	44.28	44
IRC_IRT_c3945	107	EDQ48662.1	0.0	GO:0008152	83.00	50.45	36
IRC_IRT_c3953	218	CAE03534.1	0.0	GO:0003964	98.00	151.37	72
IRC_IRT_c3961	133	XP_003546632.1	0.0	GO:0008152	77.00	46.21	35
IRC_IRT_c3989	164	NP_001175469.1	0.0	GO:0050660	100.00	81.65	37
IRC_IRT_c3991	122	NP_001050959.1	0.0	-	100.00	79.34	37
IRC_IRT_c3993	161	XP_008795067.1	0.0	GO:0008152	76.00	50.83	50
IRC_IRT_c3995	115	AAM01032.1	0.0	GO:0003676	84.00	58.54	38

IRC_IRT_c3997	253	ABA95343.1	0.0	GO:0004523	91.00	130.95	69
IRC_IRT_c4000	143	XP_002534842.1	0.0	-	68.00	50.83	44
IRC_IRT_c4004	173	XP_002536131.1	0.0	GO:0003723	80.00	65.08	55
IRC_IRT_c4026	181	BAM13283.1	0.0	GO:0006457	97.00	93.97	47
IRC_IRT_c4029	205	XP_002538951.1	0.0	-	51.00	40.43	60
IRC_IRT_c4031	113	BAJ99459.1	0.0	GO:0043168	75.00	51.22	37
IRC_IRT_c4037	180	NP_001057321.1	0.0	GO:0016788	100.00	109.77	48
IRC_IRT_c4054	107	XP_002960000.1	0.1	-	65.00	34.65	35
IRC_IRT_c4055	112	XP_006472902.1	0.5	-	52.00	33.11	34
IRC_IRT_c4066	124	XP_009599184.1	0.0	GO:0006633	82.00	61.62	41
IRC_IRT_c4072	149	KJB40315.1	0.0	-	65.00	51.60	46
IRC_IRT_c4074	115	EEC76403.1	0.0	GO:0016020	83.00	49.29	37
IRC_IRT_c4079	142	AAX95107.1	0.1	-	59.00	35.04	37
IRC_IRT_c4081	138	XP_002538051.1	0.0	GO:0008152	78.00	63.16	46
IRC_IRT_c4090	129	NP_001048208.1	0.0	GO:0016021	100.00	92.43	42
IRC_IRT_c4096	128	NP_001064098.1	0.0	GO:0003676	95.00	56.23	42
IRC_IRT_c4098	397	ABF96139.1	0.0	GO:0009536	66.00	141.74	124
IRC_IRT_c4101	149	XP_004253462.1	0.0	GO:0008270	80.00	59.31	50
IRC_IRT_c4108	120	EEC77997.1	0.0	GO:0055114	83.00	59.31	37
IRC_IRT_c4112	243	CCO19745.1	0.0	GO:0006099	83.00	67.78	43
IRC_IRT_c4113	144	ABA96887.2	0.0	GO:0006508	100.00	103.99	48
IRC_IRT_c4115	110	XP_002538040.1	0.4	-	60.00	31.96	35
IRC_IRT_c4116	126	XP_010495819.1	0.6	-	58.00	33.11	34
IRC_IRT_c4118	143	XP_001698758.1	0.5	-	54.00	33.50	42
IRC_IRT_c4124	177	CAH66267.1	0.0	GO:0003676	100.00	83.19	35
IRC_IRT_c4127	154	XP_008342266.1	0.9	-	60.00	32.73	38
IRC_IRT_c4132	187	BAD46479.1	0.0	GO:0009536	82.00	62.00	40
IRC_IRT_c4144	140	KFM22691.1	0.0	-	61.00	40.05	39
IRC_IRT_c4159	149	XP_003616487.1	0.3	-	52.00	33.88	42
IRC_IRT_c4166	108	XP_002534501.1	0.1	-	72.00	35.04	36
IRC_IRT_c4168	118	XP_008650732.1	0.0	GO:0008907	84.00	67.78	39
IRC_IRT_c4173	153	KIY99572.1	0.0	-	67.00	56.23	40
IRC_IRT_c4184	210	EEC68664.1	0.0	GO:0043168	75.00	90.89	69

IRC_IRT_c4191	214	EPS74717.1	0.0	-	45.00	39.28	61
IRC_IRT_c4193	131	XP_002966106.1	0.1	-	60.00	35.42	40
IRC_IRT_c4194	110	XP_002508163.1	0.0	GO:0017038	80.00	44.67	35
IRC_IRT_c4214	134	EAY92162.1	0.0	GO:0003735	93.00	84.73	44
IRC_IRT_c4233	143	BAD22095.1	0.0	GO:0004523	91.00	70.09	36
IRC_IRT_c4244	183	XP_002536402.1	0.0	GO:0004871	72.00	42.36	37
IRC_IRT_c4248	112	XP_004239258.1	0.0	-	79.00	36.58	34
IRC_IRT_c4284	132	XP_002949021.1	0.0	GO:0008236	75.00	54.68	41
IRC_IRT_c4290	161	KDO35890.1	0.0	-	69.00	38.89	39
IRC_IRT_c4297	170	AAT07557.1	0.0	GO:0005739	100.00	94.74	44
IRC_IRT_c4307	271	BAD69364.1	0.0	-	94.00	71.25	37
IRC_IRT_c4312	157	KFM27113.1	0.0	GO:0044763	72.00	54.68	44
IRC_IRT_c4316	120	AAT77007.1	0.0	GO:0004553	94.00	78.57	39
IRC_IRT_c4319	146	NP_001049283.1	0.0	GO:0016021	100.00	95.52	48
IRC_IRT_c4324	155	XP_002537308.1	0.0	GO:0004601	72.00	67.78	48
IRC_IRT_c4327	180	BAC84143.1	0.0	GO:0003676	92.00	89.35	50
IRC_IRT_c4340	145	XP_002522876.1	0.0	GO:0016747	71.00	53.14	46
IRC_IRT_c4341	130	ADG38689.1	0.0	GO:0071704	76.00	47.75	38
IRC_IRT_c4356	169	EEC75945.1	0.1	-	55.00	35.81	40
IRC_IRT_c4357	109	XP_002536918.1	0.0	-	68.00	40.05	35
IRC_IRT_c4360	144	KEH15665.1	0.0	-	68.00	47.75	45
IRC_IRT_c4375	109	XP_002537142.1	0.0	-	77.00	48.91	36
IRC_IRT_c4378	267	AAN05503.1	0.0	GO:0016021	86.00	80.49	44
IRC_IRT_c4380	105	EYU19144.1	0.0	GO:0003824	71.00	42.36	35
IRC_IRT_c4381	226	XP_009607159.1	0.0	GO:0016765	76.00	65.47	50
IRC_IRT_c4383	129	CAD40322.2	0.0	GO:0004523	100.00	94.36	42
IRC_IRT_c4391	124	XP_001422223.1	0.0	GO:0043648	73.00	53.91	41
IRC_IRT_c4393	148	EEC82195.1	0.0	GO:0004553	100.00	104.38	49
IRC_IRT_c4397	132	XP_004147858.1	0.0	GO:0044249	72.00	45.05	37
IRC_IRT_c4401	178	XP_002301488.2	0.3	-	49.00	34.27	57
IRC_IRT_c4404	166	XP_009350054.1	0.0	-	85.00	77.80	54
IRC_IRT_c4407	107	EEE50785.1	0.0	GO:0016021	97.00	71.25	35
IRC_IRT_c4410	190	BAC57294.1	0.0	-	100.00	72.79	35

IRC_IRT_c4412	197	CCH47196.1	0.2	-	47.00	32.73	46
IRC_IRT_c4422	278	AAV32176.1	0.0	GO:0004523	100.00	188.73	92
IRC_IRT_c4430	173	XP_002535520.1	0.1	-	56.00	35.04	57
IRC_IRT_c4433	114	XP_002464626.1	0.6	-	54.00	32.73	35
IRC_IRT_c4437	306	KEH17697.1	0.0	GO:0044444	75.00	90.12	66
IRC_IRT_c4439	173	XP_002537069.1	0.0	-	98.00	117.09	57
IRC_IRT_c4448	119	EPS62751.1	0.0	-	68.00	40.05	38
IRC_IRT_c4453	122	KFK44498.1	0.1	-	72.00	35.42	36
IRC_IRT_c4465	178	BAA25069.1	0.0	GO:0005507	84.00	81.65	59
IRC_IRT_c4475	148	BAD53597.1	0.0	GO:0016020	100.00	80.49	39
IRC_IRT_c4477	112	XP_009403575.1	0.0	GO:0016740	71.00	47.37	35
IRC_IRT_c4495	131	XP_007135815.1	0.2	-	72.00	34.27	43
IRC_IRT_c4507	112	BAD35544.1	0.0	GO:0005739	100.00	72.79	37
IRC_IRT_c4511	240	XP_008655591.1	0.0	-	52.00	37.74	68
IRC_IRT_c4514	113	EEE68527.1	0.0	-	69.00	40.43	36
IRC_IRT_c4518	139	KEH15376.1	0.2	-	58.00	34.65	48
IRC_IRT_c4521	140	AAN04909.1	0.0	GO:0003964	97.00	96.67	46
IRC_IRT_c4523	105	XP_001419359.1	0.0	GO:0007017	82.00	46.60	34
IRC_IRT_c4529	163	AAT93989.1	0.0	-	92.00	102.83	53
IRC_IRT_c4539	186	XP_006279324.1	0.0	-	61.00	40.05	42
IRC_IRT_c4542	118	EXB93351.1	0.0	GO:0003989	79.00	62.77	39
IRC_IRT_c4543	176	ACU23499.1	0.0	GO:0044763	64.00	52.37	54
IRC_IRT_c4550	154	CAH68036.1	0.0	GO:0004523	100.00	83.96	42
IRC_IRT_c4555	162	BAD32860.1	0.0	-	93.00	38.89	45
IRC_IRT_c4563	129	XP_009350809.1	0.0	-	62.00	42.36	43
IRC_IRT_c4584	165	NP_001141260.1	0.0	GO:0016853	70.00	66.24	55
IRC_IRT_c4599	153	XP_008788822.1	0.0	GO:0005488	58.00	45.82	60
IRC_IRT_c4602	129	XP_005847586.1	0.0	-	79.00	53.53	34
IRC_IRT_c4603	121	XP_002881739.1	0.8	-	63.00	32.73	38
IRC_IRT_c4605	111	XP_009407420.1	0.0	-	75.00	55.07	37
IRC_IRT_c4610	149	CAE02127.2	0.0	GO:0004523	95.00	103.22	47
IRC_IRT_c4624	129	AAF97259.1	0.7	-	57.00	32.73	35
IRC_IRT_c4625	193	BAD15577.1	0.0	GO:0016020	93.00	118.63	63

IRC_IRT_c4630	110	KIZ00023.1	0.0	-	75.00	51.22	36
IRC_IRT_c4633	114	NP_001044148.1	0.0	-	91.00	77.03	37
IRC_IRT_c4634	287	CAE02453.2	0.0	GO:0004523	100.00	200.68	95
IRC_IRT_c4642	129	NP_001048640.1	0.0	GO:0016023	100.00	76.26	36
IRC_IRT_c4646	134	KDO81115.1	0.0	-	58.00	35.04	41
IRC_IRT_c4652	301	KEH17697.1	0.0	-	62.00	36.58	37
IRC_IRT_c4653	137	XP_002982240.1	0.3	-	63.00	33.88	36
IRC_IRT_c4657	248	BAD88394.1	0.0	GO:0051287	98.00	119.78	56
IRC_IRT_c4670	116	EXC35695.1	0.0	GO:0016829	76.00	51.22	38
IRC_IRT_c4673	228	XP_002540597.1	0.0	-	62.00	50.06	66
IRC_IRT_c4682	171	BAD69326.1	0.0	-	77.00	55.07	35
IRC_IRT_c4685	108	XP_002538475.1	0.0	GO:0015419	85.00	59.31	35
IRC_IRT_c4687	113	NP_001142249.1	0.0	GO:0004013	83.00	57.38	37
IRC_IRT_c4698	176	XP_010240715.1	1.0	-	50.00	32.73	50
IRC_IRT_c4706	139	XP_002488946.1	0.0	-	61.00	38.51	36
IRC_IRT_c4708	109	XP_011016366.1	0.0	GO:0006096	80.00	48.91	35
IRC_IRT_c4717	145	EEC76953.1	0.1	-	59.00	35.81	44
IRC_IRT_c4726	151	BAH89270.1	0.0	GO:0046394	72.00	48.91	43
IRC_IRT_c4728	140	XP_010936076.1	0.0	GO:0006412	77.00	64.31	45
IRC_IRT_c4745	141	XP_002535483.1	0.0	-	57.00	36.58	40
IRC_IRT_c4748	313	KJB31094.1	0.0	-	71.00	43.51	35
IRC_IRT_c4757	162	XP_002539346.1	0.0	GO:0003824	65.00	51.99	47
IRC_IRT_c4761	159	KCW54992.1	0.2	-	73.00	34.27	49
IRC_IRT_c4771	182	NP_001045655.1	0.0	GO:0005840	100.00	104.38	49
IRC_IRT_c4777	123	AAV35813.1	0.0	GO:0004713	100.00	85.89	40
IRC_IRT_c4797	109	EMT15481.1	0.0	GO:0003857	75.00	51.60	36
IRC_IRT_c4801	289	BAD03925.1	0.0	GO:0043170	91.00	57.77	36
IRC_IRT_c4806	217	ABA98389.1	0.0	GO:0009536	68.00	79.34	80
IRC_IRT_c4821	181	ABA98110.1	0.0	GO:0003964	98.00	122.87	59
IRC_IRT_c4827	201	XP_009350076.1	0.0	GO:0016779	91.00	61.62	34
IRC_IRT_c4833	253	AIU41742.1	0.1	-	52.00	36.19	53
IRC_IRT_c4837	204	CAE04378.1	0.0	GO:0004523	100.00	92.82	43
IRC_IRT_c4851	122	XP_007217688.1	0.0	GO:0003724	78.00	52.37	37

IRC_IRT_c4852	118	XP_002539261.1	0.0	-	71.00	40.05	38
IRC_IRT_c4853	345	KDP20462.1	0.0	-	60.00	64.31	63
IRC_IRT_c4861	135	XP_011016735.1	0.0	-	64.00	40.05	37
IRC_IRT_c4862	160	XP_006649715.1	0.0	GO:0006950	100.00	85.11	52
IRC_IRT_c4888	127	AAO18442.1	0.0	GO:0004553	97.00	86.66	42
IRC_IRT_c4894	226	CAE03894.2	0.0	-	95.00	116.70	66
IRC_IRT_c4900	131	CAN80320.1	0.9	-	52.00	32.34	40
IRC_IRT_c4905	171	AAM01039.1	0.0	-	84.00	63.16	50
IRC_IRT_c4918	144	XP_002538551.1	0.0	GO:0004609	100.00	102.83	47
IRC_IRT_c4919	131	XP_004303944.1	0.0	-	65.00	40.43	40
IRC_IRT_c4921	124	AFW76407.1	0.0	-	60.00	36.97	40
IRC_IRT_c4924	180	XP_007030175.1	0.0	-	58.00	47.37	58
IRC_IRT_c4939	120	XP_002538398.1	0.1	-	67.00	35.42	37
IRC_IRT_c4950	122	EAZ11035.1	0.0	GO:0016788	100.00	83.96	40
IRC_IRT_c4952	126	BAD09330.1	0.0	-	87.00	37.74	41
IRC_IRT_c4969	235	ABA98206.1	0.0	GO:0003777	84.00	77.03	45
IRC_IRT_c4986	142	XP_006379765.1	1.0	-	65.00	31.57	35
IRC_IRT_c4994	126	EAY90295.1	0.0	GO:0006508	100.00	77.03	34
IRC_IRT_c4996	196	XP_009350810.1	0.0	-	67.00	80.49	65
IRC_IRT_c4999	112	BAD54468.1	0.0	-	100.00	75.10	35
IRC_IRT_c5010	138	AAX96435.1	0.0	GO:0003964	85.00	66.24	35
IRC_IRT_c5017	110	BAJ93352.1	0.8	-	65.00	32.34	35
IRC_IRT_c5019	233	ABA97592.1	0.0	GO:0005739	98.00	144.44	74
IRC_IRT_c5020	172	XP_010911754.1	0.0	-	50.00	40.82	58
IRC_IRT_c5022	132	CAD40111.2	0.0	GO:0004523	100.00	90.51	43
IRC_IRT_c5028	113	XP_008662994.1	0.0	-	64.00	38.12	34
IRC_IRT_c5035	129	CDY63646.1	0.3	-	60.00	31.57	35
IRC_IRT_c5037	146	KEH15555.1	0.5	-	48.00	33.11	45
IRC_IRT_c5059	103	XP_011016720.1	0.7	-	58.00	32.34	34
IRC_IRT_c5064	150	AFK35665.1	0.0	-	63.00	45.82	49
IRC_IRT_c5069	126	XP_001786416.1	0.0	-	70.00	37.35	40
IRC_IRT_c5082	139	XP_011470593.1	0.7	-	66.00	32.73	42
IRC_IRT_c5084	111	XP_002535489.1	0.0	-	89.00	64.70	37

IRC_IRT_c5086	126	XP_009350054.1	0.0	-	70.00	51.22	37
IRC_IRT_c5092	119	XP_008779849.1	0.0	-	76.00	53.91	38
IRC_IRT_c5097	114	EAY80233.1	0.0	-	64.00	45.82	42
IRC_IRT_c5103	116	AAX96316.1	0.0	GO:0004523	100.00	83.96	38
IRC_IRT_c5105	177	XP_004302282.1	0.5	-	50.00	33.88	34
IRC_IRT_c5110	116	XP_002536752.1	0.0	GO:0004364	92.00	67.40	38
IRC_IRT_c5117	106	CCO19799.1	0.0	GO:0006783	85.00	62.77	35
IRC_IRT_c5118	160	XP_009759241.1	0.0	GO:0044248	65.00	49.68	52
IRC_IRT_c5123	125	EEC69469.1	0.0	GO:0008152	61.00	46.21	39
IRC_IRT_c5130	109	CAN74051.1	0.2	-	58.00	33.88	34
IRC_IRT_c5137	188	BAD36512.1	0.0	GO:0016023	71.00	64.31	49
IRC_IRT_c5142	202	CAI64478.1	0.0	GO:0016023	100.00	120.55	56
IRC_IRT_c5143	122	XP_002953815.1	0.0	GO:0006259	65.00	43.51	38
IRC_IRT_c5152	230	BAH80064.1	0.0	GO:0003676	96.00	114.01	56
IRC_IRT_c5159	250	XP_006409179.1	0.1	-	60.00	35.81	43
IRC_IRT_c5162	111	XP_010238512.1	0.6	-	55.00	32.73	40
IRC_IRT_c5167	112	YP_009057880.1	0.0	GO:0003899	94.00	65.47	37
IRC_IRT_c5168	155	P53385.1	0.0	GO:0019557	90.00	90.12	51
IRC_IRT_c5172	202	XP_009415149.1	0.0	GO:0015930	68.00	55.84	44
IRC_IRT_c5192	136	ABQ59348.1	0.0	-	68.00	55.07	45
IRC_IRT_c5193	115	CEF96753.1	0.0	GO:0044765	73.00	45.05	38
IRC_IRT_c5201	160	XP_002536340.1	0.0	-	64.00	42.74	51
IRC_IRT_c5208	174	EMS48387.1	0.1	-	47.00	36.19	51
IRC_IRT_c5211	194	YP_514656.1	0.0	GO:0005739	89.00	65.08	37
IRC_IRT_c5216	186	ACN30565.1	0.0	-	47.00	36.97	55
IRC_IRT_c5218	128	NP_001174195.1	0.0	GO:0043531	100.00	85.89	42
IRC_IRT_c5222	136	XP_002442180.1	0.0	-	53.00	36.97	45
IRC_IRT_c5223	169	XP_002539318.1	0.0	-	87.00	92.05	55
IRC_IRT_c5225	143	XP_006588110.1	0.2	-	51.00	34.27	41
IRC_IRT_c5227	137	XP_002536193.1	0.0	GO:0015416	90.00	66.63	41
IRC_IRT_c5234	117	XP_011016759.1	0.0	-	73.00	54.30	38
IRC_IRT_c5236	104	XP_004966709.1	0.0	-	64.00	36.97	34
IRC_IRT_c5238	136	XP_002537217.1	0.0	GO:0009684	79.00	56.23	39

IRC_IRT_c5239	106	XP_011072621.1	0.0	GO:0008152	76.00	46.21	34
IRC_IRT_c5244	206	EAY84569.1	0.0	GO:0006996	76.00	102.06	68
IRC_IRT_c5247	121	XP_004253365.2	0.0	GO:0046933	87.00	64.70	39
IRC_IRT_c5249	105	XP_004494951.1	0.0	GO:0003824	82.00	50.45	34
IRC_IRT_c5254	110	XP_002536712.1	0.0	-	69.00	38.51	36
IRC_IRT_c5272	158	XP_002536523.1	0.0	-	76.00	67.78	52
IRC_IRT_c5273	125	KJB77431.1	0.0	GO:0008236	75.00	65.86	41
IRC_IRT_c5276	182	XP_009614702.1	0.2	-	59.00	33.50	52
IRC_IRT_c5285	128	KJB73889.1	0.1	-	52.00	36.19	40
IRC_IRT_c5290	109	KFM27114.1	0.0	GO:0005507	83.00	53.91	36
IRC_IRT_c5306	138	XP_003083193.1	0.0	GO:0006499	93.00	97.06	46
IRC_IRT_c5320	132	EAZ15557.1	0.0	GO:0015238	100.00	76.26	36
IRC_IRT_c5327	174	XP_005843440.1	0.0	GO:0048037	79.00	50.83	34
IRC_IRT_c5330	229	ABA96234.2	0.0	GO:0003964	98.00	167.55	75
IRC_IRT_c5331	153	XP_004977163.1	0.2	-	50.00	34.65	55
IRC_IRT_c5336	164	ABF99578.1	0.0	GO:0009536	95.00	83.96	46
IRC_IRT_c5339	146	ABA98463.1	0.0	GO:0005739	100.00	91.28	36
IRC_IRT_c5342	171	BAH79986.1	0.0	GO:0008234	94.00	113.24	54
IRC_IRT_c5351	109	XP_011016366.1	0.0	GO:0006096	86.00	62.77	36
IRC_IRT_c5354	118	CCO18335.1	0.0	-	64.00	40.43	39
IRC_IRT_c5357	191	XP_003063226.1	0.0	GO:0004450	78.00	71.63	51
IRC_IRT_c5360	229	XP_002536284.1	0.0	-	66.00	40.82	50
IRC_IRT_c5366	163	XP_004253340.1	0.0	GO:0016874	92.00	68.17	38
IRC_IRT_c5368	161	XP_002540539.1	0.0	GO:0005982	86.00	84.34	53
IRC_IRT_c5370	121	XP_002436626.1	0.0	GO:0016740	70.00	57.00	44
IRC_IRT_c5371	127	XP_010039161.1	0.1	-	46.00	33.50	39
IRC_IRT_c5376	110	XP_002538729.1	0.2	-	65.00	33.88	35
IRC_IRT_c5380	172	NP_001050319.1	0.0	GO:0045893	97.00	91.28	44
IRC_IRT_c5383	200	ABA91138.1	0.0	-	89.00	73.94	37
IRC_IRT_c5386	136	KFK35540.1	0.0	GO:0006783	89.00	72.79	39
IRC_IRT_c5397	124	EAY87054.1	0.0	GO:0006855	100.00	88.58	41
IRC_IRT_c5400	140	XP_003558735.1	0.0	-	73.00	55.45	46
IRC_IRT_c5409	151	XP_010918705.1	0.7	-	43.00	33.11	55

IRC_IRT_c5411	148	ABA98432.1	0.0	GO:0005739	91.00	97.06	48
IRC_IRT_c5423	158	AAO24901.1	0.0	GO:0004523	100.00	86.66	52
IRC_IRT_c5427	160	XP_008777791.1	0.0	-	62.00	36.58	51
IRC_IRT_c5433	112	EDQ48154.1	0.0	GO:0008152	70.00	44.28	37
IRC_IRT_c5445	152	EEC77111.1	0.0	GO:0050896	77.00	51.22	48
IRC_IRT_c5448	118	EMS58729.1	0.0	GO:0005829	100.00	80.11	39
IRC_IRT_c5473	268	CAE04969.2	0.0	GO:0003964	100.00	180.26	86
IRC_IRT_c5481	141	XP_002508801.1	0.0	GO:0071704	71.00	55.84	46
IRC_IRT_c5483	157	KFM27260.1	0.0	GO:0008483	68.00	56.23	50
IRC_IRT_c5485	155	KIY95500.1	0.0	GO:0005524	80.00	55.84	40
IRC_IRT_c5492	118	XP_004495927.1	0.0	GO:0008483	68.00	45.05	38
IRC_IRT_c5504	134	EYU46650.1	0.8	-	60.00	32.73	35
IRC_IRT_c5522	130	XP_002534915.1	0.1	-	72.00	35.42	36
IRC_IRT_c5527	149	XP_005646747.1	0.0	-	68.00	39.66	38
IRC_IRT_c5529	118	ABA97779.1	0.0	GO:0003964	100.00	88.97	39
IRC_IRT_c5556	139	XP_002535185.1	0.0	-	97.00	57.77	46
IRC_IRT_c5569	114	XP_009149951.1	0.0	-	73.00	37.35	34
IRC_IRT_c5571	103	XP_002535078.1	0.0	-	97.00	73.94	34
IRC_IRT_c5586	224	XP_002534883.1	0.0	GO:0005488	62.00	60.46	64
IRC_IRT_c5614	147	EEE55237.1	0.0	GO:0017148	100.00	104.38	48
IRC_IRT_c5631	147	XP_002540194.1	0.0	GO:0008483	65.00	51.22	43
IRC_IRT_c5654	166	XP_008465985.1	0.6	-	56.00	33.11	39
IRC_IRT_c5656	135	AAT85205.1	0.0	GO:0008270	100.00	88.20	43
IRC_IRT_c5664	128	ADG37979.1	0.0	GO:0045454	94.00	65.47	36
IRC_IRT_c5673	177	XP_004142492.1	0.7	-	54.00	33.11	42
IRC_IRT_c5674	129	XP_003588347.1	0.0	-	94.00	74.71	37
IRC_IRT_c5676	276	AAX95920.1	0.0	GO:0003964	100.00	199.13	9.10E+01
IRC_IRT_c5678	115	XP_002968304.1	0.0	GO:0046872	76.00	51.99	38
IRC_IRT_c5679	103	BAD52843.1	0.0	GO:0046872	100.00	71.25	34
IRC_IRT_c5680	145	AAT77831.1	0.0	GO:0003676	87.00	73.17	48
IRC_IRT_c5684	120	XP_009361622.1	0.0	GO:0008094	84.00	63.16	39
IRC_IRT_c5712	113	AFW80459.1	0.0	GO:0016021	100.00	75.10	34
IRC_IRT_c5714	128	XP_002539661.1	0.0	-	73.00	45.05	42

IRC_IRT_c5717	109	CCO20244.1	0.1	-	66.00	34.65	36
IRC_IRT_c5727	188	XP_003064993.1	0.0	-	58.00	46.98	60
IRC_IRT_c5730	138	EEE52318.1	0.0	GO:0022891	88.00	79.72	45
IRC_IRT_c5737	158	EEC70905.1	0.0	GO:0044699	68.00	59.31	45
IRC_IRT_c5756	172	XP_009369482.1	0.9	-	62.00	31.96	37
IRC_IRT_c5757	121	XP_002538952.1	0.0	-	85.00	65.08	40
IRC_IRT_c5761	120	XP_005643761.1	0.0	-	71.00	40.43	38
IRC_IRT_c5763	145	BAK02541.1	0.0	-	61.00	38.51	47
IRC_IRT_c5765	339	EXB37177.1	0.0	GO:0006413	86.00	78.57	51
IRC_IRT_c5769	148	KDD72261.1	0.0	GO:0003723	81.00	65.86	48
IRC_IRT_c5783	278	BAH80021.1	0.0	-	92.00	55.45	66
IRC_IRT_c5798	128	CCO15175.1	0.0	-	69.00	38.89	36
IRC_IRT_c5804	147	EEC76774.1	0.0	GO:0055085	85.00	49.29	34
IRC_IRT_c5805	154	EEC73998.1	0.0	GO:0019187	100.00	102.45	51
IRC_IRT_c5807	173	KEH15576.1	0.0	-	67.00	54.68	52
IRC_IRT_c5810	131	XP_002538703.1	0.0	-	64.00	36.97	39
IRC_IRT_c5814	132	KEH15204.1	0.0	-	58.00	36.19	43
IRC_IRT_c5824	116	XP_005651412.1	0.0	-	64.00	37.35	34
IRC_IRT_c5830	103	KJB16593.1	0.1	-	60.00	35.42	41
IRC_IRT_c5831	225	YP_001152218.1	0.0	-	56.00	48.91	53
IRC_IRT_c5846	101	XP_005848159.1	0.0	-	65.00	37.35	35
IRC_IRT_c5851	146	XP_009384113.1	0.2	-	54.00	34.27	48
IRC_IRT_c5861	107	XP_003080549.1	0.0	-	74.00	38.89	35
IRC_IRT_c5869	630	ACU24411.1	0.0	-	77.00	62.00	44
IRC_IRT_c5876	167	XP_011012113.1	0.2	-	57.00	34.65	38
IRC_IRT_c5882	209	EEE52320.1	0.0	GO:0055085	88.00	82.03	59
IRC_IRT_c5884	120	XP_002446972.1	0.0	GO:0016491	100.00	83.57	38
IRC_IRT_c5887	147	XP_010230059.1	0.8	-	53.00	32.73	39
IRC_IRT_c5890	162	XP_010488843.1	0.0	GO:0008033	73.00	45.82	38
IRC_IRT_c5892	115	XP_006827744.2	0.0	-	57.00	39.28	38
IRC_IRT_c5895	118	AGC78945.1	0.0	-	68.00	40.82	35
IRC_IRT_c5912	214	XP_008812963.1	0.0	-	52.00	42.36	50
IRC_IRT_c5918	128	XP_002536230.1	0.0	-	71.00	43.13	35

IRC_IRT_c5921	156	XP_002869278.1	0.8	-	47.00	32.73	48
IRC_IRT_c5923	154	XP_002953691.1	0.0	GO:0016740	72.00	62.00	50
IRC_IRT_c5931	201	XP_010253283.1	0.2	-	42.00	34.65	59
IRC_IRT_c5936	146	KDO70744.1	0.0	GO:0042802	91.00	78.18	48
IRC_IRT_c5947	131	XP_002509369.1	0.0	-	67.00	40.05	37
IRC_IRT_c5952	154	XP_002535099.1	0.0	-	68.00	45.05	51
IRC_IRT_c5954	162	XP_005842766.1	0.0	-	68.00	53.53	45
IRC_IRT_c5955	210	XP_002455376.1	0.9	-	56.00	33.11	41
IRC_IRT_c5961	104	EEE51130.1	0.0	GO:0050660	100.00	74.33	34
IRC_IRT_c5963	260	EMT24921.1	0.1	-	40.00	36.19	81
IRC_IRT_c5968	114	XP_002536183.1	0.5	-	59.00	32.73	37
IRC_IRT_c5971	189	XP_003064278.1	0.0	GO:0005829	66.00	50.83	42
IRC_IRT_c5980	175	EEC73650.1	0.0	-	62.00	40.82	40
IRC_IRT_c5992	125	YP_588403.1	0.0	-	66.00	36.19	36
IRC_IRT_c5997	381	KEH22088.1	0.0	-	76.00	58.54	42
IRC_IRT_c6003	151	EXC01914.1	0.9	-	55.00	31.96	34
IRC_IRT_c6011	114	XP_002539068.1	0.2	-	52.00	33.88	38
IRC_IRT_c6018	144	XP_009378140.1	0.0	GO:0044238	74.00	63.54	47
IRC_IRT_c6033	121	XP_002537941.1	0.0	GO:0044763	72.00	45.05	40
IRC_IRT_c6034	118	BAC80136.1	0.0	-	100.00	83.57	39
IRC_IRT_c6046	227	XP_007050763.1	0.0	-	68.00	47.75	44
IRC_IRT_c6052	139	XP_001418866.1	0.0	-	55.00	37.74	47
IRC_IRT_c6067	248	CCO19499.1	0.0	GO:0006457	74.00	57.00	50
IRC_IRT_c6075	127	CCO14164.1	0.5	-	51.00	33.50	35
IRC_IRT_c6077	109	NP_001064192.1	0.0	GO:0005829	100.00	72.79	3.60E+01
IRC_IRT_c6082	152	XP_003614392.1	0.0	-	71.00	45.44	35
IRC_IRT_c6084	149	EXC34899.1	0.0	-	70.00	40.43	37
IRC_IRT_c6097	159	XP_002871536.1	0.0	GO:0005576	71.00	59.69	52
IRC_IRT_c6107	132	XP_002530453.1	0.8	-	44.00	32.34	36
IRC_IRT_c6112	125	AAN08919.1	0.0	GO:0016151	83.00	56.61	36
IRC_IRT_c6116	168	EMS58925.1	0.5	-	52.00	31.19	38
IRC_IRT_c6120	133	EDQ48547.1	0.0	-	73.00	36.58	34
IRC_IRT_c6135	140	NP_001057209.1	0.0	GO:0016021	100.00	100.14	46

IRC_IRT_c6139	154	CAE04908.2	0.0	GO:0009536	86.00	55.84	36
IRC_IRT_c6141	115	XP_002534939.1	0.0	-	73.00	46.21	38
IRC_IRT_c6143	105	NP_001047698.1	0.0	-	97.00	79.34	34
IRC_IRT_c6149	131	EEC69860.1	0.7	-	62.00	32.73	35
IRC_IRT_c6155	163	XP_002537670.1	0.0	-	74.00	75.87	54
IRC_IRT_c6170	111	CAE02184.2	0.0	GO:0003676	94.00	64.70	34
IRC_IRT_c6174	112	CAE02456.1	0.0	GO:0004523	97.00	76.64	37
IRC_IRT_c6175	168	DAA41613.1	0.0	GO:0016021	100.00	79.34	39
IRC_IRT_c6183	118	EAY93711.1	0.0	GO:0016023	97.00	79.72	39
IRC_IRT_c6205	160	XP_006644300.1	0.0	-	61.00	35.42	36
IRC_IRT_c6207	115	BAD13184.1	0.0	GO:0030599	100.00	79.72	38
IRC_IRT_c6214	312	AGC78945.1	0.0	-	48.00	55.07	77
IRC_IRT_c6220	206	KJB15230.1	0.0	-	65.00	38.12	40
IRC_IRT_c6229	155	XP_002535793.1	0.0	GO:0015031	88.00	84.34	51
IRC_IRT_c6247	233	CAA69903.1	0.0	GO:0005739	66.00	51.60	51
IRC_IRT_c6267	183	XP_005851962.1	0.3	-	54.00	34.27	46
IRC_IRT_c6268	126	KEH15262.1	0.0	GO:0009536	82.00	62.77	39
IRC_IRT_c6274	139	XP_002535771.1	0.0	-	65.00	39.66	44
IRC_IRT_c6284	127	XP_002954930.1	0.0	-	60.00	37.35	35
IRC_IRT_c6288	103	AIT94565.1	0.0	-	70.00	39.28	34
IRC_IRT_c6294	116	KJB33927.1	0.0	GO:0005737	100.00	80.49	38
IRC_IRT_c6299	143	XP_002968236.1	0.0	-	70.00	42.74	34
IRC_IRT_c6306	115	AAR15338.1	0.0	GO:0009536	82.00	56.61	34
IRC_IRT_c6308	251	EEE69435.1	0.0	GO:0016021	98.00	158.30	80
IRC_IRT_c6311	145	CDP22216.1	0.0	GO:0007000	74.00	53.91	39
IRC_IRT_c6315	139	CAH66255.1	0.0	GO:0005739	100.00	101.29	46
IRC_IRT_c6322	158	AAR89870.1	0.0	GO:0003677	100.00	79.72	38
IRC_IRT_c6325	122	XP_001787050.1	0.6	-	57.00	32.73	35
IRC_IRT_c6342	144	XP_002534890.1	0.0	GO:0015079	85.00	72.40	47
IRC_IRT_c6347	228	AAL93077.1	0.0	GO:0044260	82.00	60.08	51
IRC_IRT_c6348	204	XP_009350817.1	0.0	GO:0005840	88.00	90.89	60
IRC_IRT_c6351	114	ABA97820.2	0.0	GO:0004523	82.00	68.55	39
IRC_IRT_c6356	184	XP_004248038.1	0.0	GO:0044699	62.00	48.52	61

IRC_IRT_c6359	111	AAT85299.1	0.0	GO:0016301	100.00	74.71	36
IRC_IRT_c6367	125	XP_005847592.1	0.0	GO:0008152	82.00	63.16	40
IRC_IRT_c6370	126	XP_002535590.1	0.0	-	65.00	43.13	41
IRC_IRT_c6388	185	KIY95857.1	0.0	GO:0046872	74.00	74.71	58
IRC_IRT_c6393	110	XP_006828428.1	0.0	GO:0044699	73.00	44.67	34
IRC_IRT_c6408	127	XP_002537242.1	0.0	-	80.00	51.99	35
IRC_IRT_c6414	125	XP_010466713.1	0.0	-	64.00	42.74	37
IRC_IRT_c6420	146	BAD87807.1	0.0	-	73.00	55.84	38
IRC_IRT_c6431	166	EMT20322.1	0.0	GO:0000786	100.00	115.93	55
IRC_IRT_c6432	115	AAC17055.1	0.3	-	67.00	33.88	34
IRC_IRT_c6434	211	EMS54416.1	0.0	GO:0008233	83.00	102.06	67
IRC_IRT_c6446	153	YP_001315124.1	0.0	GO:0051287	87.00	68.94	39
IRC_IRT_c6455	175	KFK43204.1	0.0	GO:0004553	72.00	50.06	37
IRC_IRT_c6458	117	XP_005851575.1	0.0	GO:0008152	72.00	42.74	37
IRC_IRT_c6460	139	AAM74253.1	0.0	GO:0003964	100.00	100.52	45
IRC_IRT_c6461	119	AIS72813.1	0.6	-	56.00	32.34	39
IRC_IRT_c6477	334	KJB49896.1	0.0	-	57.00	39.28	40
IRC_IRT_c6494	142	EEC74694.1	0.0	GO:0006306	97.00	92.82	47
IRC_IRT_c6505	132	ABD28403.1	0.0	-	58.00	40.05	43
IRC_IRT_c6512	159	KJB09734.1	0.2	-	59.00	33.88	37
IRC_IRT_c6534	110	XP_006653723.1	0.0	GO:0003677	73.00	49.29	41
IRC_IRT_c6546	127	XP_002535781.1	0.0	GO:0016829	73.00	53.53	41
IRC_IRT_c6551	187	XP_003637074.1	0.0	-	63.00	43.51	46
IRC_IRT_c6556	388	AAT73685.1	0.0	GO:0003676	94.00	75.49	37
IRC_IRT_c6560	109	CAE01924.2	0.0	GO:0003676	97.00	68.55	36
IRC_IRT_c6572	280	AAX96712.1	0.0	GO:0003676	65.00	87.43	78
IRC_IRT_c6576	150	CAE02229.2	0.0	-	57.00	38.51	35
IRC_IRT_c6581	172	XP_006402463.1	0.1	-	55.00	35.81	49
IRC_IRT_c6599	171	KJB31094.1	0.0	-	73.00	41.97	34
IRC_IRT_c6607	126	EAY93221.1	0.0	GO:0043565	100.00	87.81	41
IRC_IRT_c6640	145	XP_006596429.1	0.9	-	60.00	32.73	35
IRC_IRT_c6642	201	XP_002536751.1	0.0	-	85.00	87.81	55
IRC_IRT_c6643	120	XP_010423854.1	0.0	GO:0032440	100.00	81.65	36

IRC_IRT_c6646	257	XP_002536037.1	0.0	-	62.00	53.53	54
IRC_IRT_c6652	208	ABA96803.1	0.0	GO:0003964	100.00	107.46	50
IRC_IRT_c6653	153	KFK34823.1	0.0	GO:0048471	61.00	49.29	49
IRC_IRT_c6654	116	XP_002500636.1	0.8	-	64.00	32.73	34
IRC_IRT_c6666	142	XP_002536196.1	0.0	GO:0009642	78.00	59.31	41
IRC_IRT_c6670	134	CAE04483.1	0.0	GO:0090502	93.00	86.66	44
IRC_IRT_c6671	119	XP_010911538.1	0.0	-	84.00	60.08	39
IRC_IRT_c6684	263	XP_003610227.1	0.3	-	49.00	35.04	53
IRC_IRT_c6695	190	YP_588403.1	0.0	-	73.00	52.76	41
IRC_IRT_c6715	262	CDY45505.1	0.0	-	58.00	41.97	41
IRC_IRT_c6733	174	AAV32099.1	0.0	GO:0005739	79.00	63.54	43
IRC_IRT_c6736	110	XP_009349824.1	0.0	-	61.00	38.89	36
IRC_IRT_c6740	108	XP_002538503.1	0.0	-	66.00	46.21	36
IRC_IRT_c6759	108	CCO18981.1	0.0	-	66.00	36.58	36
IRC_IRT_c6760	146	AAM92813.1	0.0	GO:0005634	93.00	84.73	46
IRC_IRT_c6764	156	AAV32160.1	0.4	-	85.00	33.88	47
IRC_IRT_c6775	142	BAC98576.1	0.0	GO:0009536	93.00	91.66	47
IRC_IRT_c6776	108	XP_006657259.1	0.0	GO:0016310	94.00	69.32	34
IRC_IRT_c6808	106	EEC77855.1	0.0	GO:0006935	97.00	59.31	34
IRC_IRT_c6809	148	XP_005650079.1	0.9	-	55.00	32.34	38
IRC_IRT_c6816	130	XP_007204799.1	0.7	-	59.00	33.11	37
IRC_IRT_c6823	238	AAX96499.1	0.2	-	42.00	35.04	59
IRC_IRT_c6835	216	XP_002536382.1	0.0	GO:0003700	91.00	91.28	49
IRC_IRT_c6838	120	XP_002535100.1	0.0	GO:0009058	78.00	51.99	37
IRC_IRT_c6852	136	XP_002538274.1	0.0	-	62.00	42.74	43
IRC_IRT_c6869	131	AAQ56337.1	0.0	-	80.00	64.70	41
IRC_IRT_c6870	116	XP_010272278.1	0.0	-	70.00	43.13	37
IRC_IRT_c6874	161	XP_006405917.1	0.0	-	82.00	52.37	34
IRC_IRT_c6875	121	XP_011002727.1	0.0	GO:0007010	80.00	62.77	40
IRC_IRT_c6878	177	CAE01728.2	0.0	GO:0004523	100.00	119.01	58
IRC_IRT_c6882	135	KEH15204.1	0.0	-	61.00	38.12	44
IRC_IRT_c6883	112	NP_001046627.2	0.0	GO:0009536	100.00	58.92	37
IRC_IRT_c6886	140	BAA83103.1	0.0	GO:0004427	80.00	65.08	45

IRC_IRT_c6894	117	BAD17630.1	0.0	GO:0010363	100.00	87.04	39
IRC_IRT_c6901	155	XP_002539534.1	0.0	GO:0055114	88.00	87.04	51
IRC_IRT_c6920	110	EAY93131.1	0.0	GO:0006200	77.00	51.99	36
IRC_IRT_c6927	106	AFK45685.1	0.0	GO:0016874	79.00	48.14	34
IRC_IRT_c6946	121	EEC76122.1	0.0	GO:0050660	100.00	86.27	40
IRC_IRT_c6950	134	BAD16833.1	0.0	GO:0009941	81.00	59.31	37
IRC_IRT_c6951	173	KJB65027.1	0.0	GO:0006810	71.00	73.56	57
IRC_IRT_c6953	124	ABA95720.1	0.0	GO:0005488	77.00	47.37	36
IRC_IRT_c6959	123	AAX95176.1	0.0	GO:0003676	91.00	72.79	37
IRC_IRT_c6961	155	XP_001786908.1	0.3	-	49.00	33.11	51
IRC_IRT_c6968	142	XP_001762691.1	0.0	GO:0043161	80.00	66.24	46
IRC_IRT_c6971	127	XP_002538791.1	0.0	GO:0016798	70.00	45.44	37
IRC_IRT_c6972	135	XP_001421062.1	1.0	-	46.00	32.34	45
IRC_IRT_c6976	115	XP_002536199.1	0.4	-	64.00	33.11	37
IRC_IRT_c7017	106	EAY93355.1	0.0	GO:0016020	100.00	73.94	35
IRC_IRT_c7020	193	XP_008775398.1	0.4	-	50.00	33.88	44
IRC_IRT_c7038	148	EXB38650.1	0.3	-	54.00	34.27	48
IRC_IRT_c7050	172	XP_010241950.1	0.8	-	53.00	32.34	39
IRC_IRT_c7051	138	KIY95081.1	0.0	-	55.00	35.81	40
IRC_IRT_c7071	152	XP_002534847.1	0.0	GO:0003857	80.00	72.40	50
IRC_IRT_c7083	130	XP_002539112.1	0.0	-	66.00	40.43	36
IRC_IRT_c7088	177	CAE04439.2	0.0	-	92.00	97.44	55
IRC_IRT_c7089	106	XP_006282342.1	0.6	-	68.00	32.73	38
IRC_IRT_c7093	122	XP_004971407.1	0.9	-	55.00	30.03	43
IRC_IRT_c7096	121	XP_002960029.1	0.0	-	62.00	36.19	40
IRC_IRT_c7099	124	BAD03258.1	0.0	-	65.00	38.51	41
IRC_IRT_c7105	124	EMT07829.1	0.0	GO:0046961	100.00	83.19	40
IRC_IRT_c7116	153	XP_002538383.1	0.0	-	72.00	55.07	51
IRC_IRT_c7122	196	BAB90455.1	0.0	GO:0009536	91.00	98.60	56
IRC_IRT_c7123	148	XP_009334076.1	0.0	-	70.00	42.36	34
IRC_IRT_c7131	137	EDQ48122.1	0.0	-	64.00	45.82	34
IRC_IRT_c7140	115	AAX95258.1	0.0	-	63.00	35.81	36
IRC_IRT_c7142	156	BAD82460.1	0.0	-	84.00	57.00	39

IRC_IRT_c7151	160	XP_002540443.1	0.0	GO:0070918	85.00	67.40	42
IRC_IRT_c7154	109	XP_002537081.1	0.3	-	58.00	32.73	41
IRC_IRT_c7159	175	XP_002538833.1	0.0	-	58.00	44.28	43
IRC_IRT_c7168	130	XP_002534642.1	0.0	GO:0006810	76.00	54.68	38
IRC_IRT_c7169	276	XP_002509337.1	0.0	GO:0004061	80.00	63.16	41
IRC_IRT_c7181	136	XP_005648657.1	0.1	-	86.00	35.04	45
IRC_IRT_c7208	140	XP_005651027.1	0.0	GO:0044765	75.00	62.77	45
IRC_IRT_c7211	247	XP_001702121.1	0.0	-	50.00	36.58	60
IRC_IRT_c7223	167	AAX94870.1	0.0	GO:0004523	91.00	98.98	56
IRC_IRT_c7228	186	AET50001.1	0.0	GO:0044763	70.00	63.16	50
IRC_IRT_c7233	125	XP_010911063.1	0.0	-	62.00	40.82	40
IRC_IRT_c7237	220	XP_002977868.1	1.0	-	61.00	33.11	39
IRC_IRT_c7239	134	XP_002537904.1	0.0	-	86.00	74.71	44
IRC_IRT_c7240	134	XP_002536899.1	0.0	GO:0030170	93.00	76.26	44
IRC_IRT_c7242	127	XP_002536890.1	0.0	-	64.00	37.74	34
IRC_IRT_c7250	168	XP_002982817.1	0.0	GO:0005975	76.00	63.93	46
IRC_IRT_c7254	134	ABA97704.2	0.0	-	97.00	70.48	44
IRC_IRT_c7269	125	BAD45229.1	0.0	-	65.00	40.05	38
IRC_IRT_c7275	134	CAD39360.2	0.0	GO:0003964	100.00	93.20	44
IRC_IRT_c7287	215	CAE04969.2	0.0	GO:0003676	100.00	88.97	42
IRC_IRT_c7298	157	XP_010051894.1	0.0	-	65.00	40.05	43
IRC_IRT_c7300	138	XP_008232518.1	0.5	-	59.00	33.50	37
IRC_IRT_c7301	115	XP_003082803.1	0.0	GO:0005618	92.00	72.40	38
IRC_IRT_c7304	176	BAC06248.1	0.0	-	97.00	68.94	34
IRC_IRT_c7316	106	EEC77111.1	0.0	GO:0006935	85.00	46.98	34
IRC_IRT_c7319	172	XP_002500636.1	0.0	-	60.00	38.12	58
IRC_IRT_c7321	142	XP_010912951.1	0.0	-	57.00	38.89	40
IRC_IRT_c7324	125	XP_010911695.1	0.6	-	57.00	31.19	35
IRC_IRT_c7338	110	XP_010314936.1	0.0	-	58.00	36.19	36
IRC_IRT_c7353	110	BAD26252.1	0.0	GO:0005739	69.00	40.82	36
IRC_IRT_c7356	117	XP_004508940.1	0.6	-	57.00	32.34	35
IRC_IRT_c7375	274	BAC84863.1	0.0	GO:0005739	100.00	103.99	50
IRC_IRT_c7380	236	ABA97319.1	0.0	GO:0003964	100.00	110.54	54

IRC_IRT_c7381	119	XP_002540253.1	0.0	GO:0043169	71.00	41.59	35
IRC_IRT_c7384	310	XP_010026486.1	0.2	-	58.00	35.81	41
IRC_IRT_c7387	168	XP_001416185.1	0.0	-	62.00	56.23	56
IRC_IRT_c7395	194	XP_002538511.1	0.0	GO:0006355	80.00	87.43	60
IRC_IRT_c7399	123	XP_001773713.1	0.0	-	62.00	47.75	40
IRC_IRT_c7426	220	AFW84663.1	0.9	-	56.00	30.80	39
IRC_IRT_c7481	103	AAU44099.1	0.0	GO:0004523	97.00	71.63	34
IRC_IRT_c7513	151	XP_002538873.1	0.0	-	62.00	38.12	51
IRC_IRT_c7525	279	EPS70023.1	0.0	-	64.00	37.74	34
IRC_IRT_c7538	111	XP_001418734.1	0.0	-	65.00	36.58	35
IRC_IRT_c7548	459	AIU49871.1	0.2	-	50.00	37.35	75
IRC_IRT_c7560	110	XP_004980087.1	0.0	GO:0005524	88.00	67.78	36
IRC_IRT_c7572	136	AAQ56303.1	0.0	GO:0004523	100.00	75.10	37
IRC_IRT_c7580	155	AIJ28247.1	0.0	-	61.00	31.19	34
IRC_IRT_c7597	141	EEC70905.1	0.0	GO:0003824	58.00	43.51	46
IRC_IRT_c7613	123	XP_003638717.1	0.0	-	64.00	41.20	45
IRC_IRT_c7618	115	NP_001053645.1	0.0	GO:0016567	100.00	84.34	38
IRC_IRT_c7624	108	XP_001418921.1	0.1	-	74.00	35.42	35
IRC_IRT_c7637	107	ABF96674.1	0.0	GO:0003676	97.00	78.18	35
IRC_IRT_c7668	194	XP_002537935.1	0.0	-	80.00	51.60	35
IRC_IRT_c7669	111	XP_009350072.1	0.0	GO:0003735	82.00	51.60	35
IRC_IRT_c7673	144	CDX67406.1	0.8	-	47.00	32.73	42
IRC_IRT_c7676	112	EMS50947.1	0.0	GO:0008237	83.00	57.77	36
IRC_IRT_c7677	155	AAU93936.1	0.0	-	67.00	39.66	49
IRC_IRT_c7688	152	EEE61005.1	0.0	GO:0036459	92.00	101.29	50
IRC_IRT_c7690	106	XP_006842185.2	0.0	GO:0031071	79.00	50.83	34
IRC_IRT_c7725	155	XP_003610227.1	0.0	-	80.00	59.69	36
IRC_IRT_c7731	220	XP_002535080.1	0.0	-	64.00	40.43	39
IRC_IRT_c7748	113	EAZ07843.1	0.0	GO:0008810	94.00	72.79	36
IRC_IRT_c7751	109	XP_001785024.1	0.3	-	51.00	32.73	47
IRC_IRT_c7752	215	XP_002539621.1	0.0	-	80.00	90.51	65
IRC_IRT_c7787	210	YP_001019064.1	0.4	-	47.00	33.88	76
IRC_IRT_c7794	127	XP_011016263.1	0.0	-	78.00	61.23	41

IRC_IRT_c7795	126	XP_005649803.1	0.0	GO:0005739	78.00	58.92	42
IRC_IRT_c7805	118	XP_009351045.1	0.2	-	63.00	34.27	38
IRC_IRT_c7817	117	XP_002538528.1	0.0	-	94.00	84.73	38
IRC_IRT_c7824	143	AFK45435.1	0.0	GO:0016651	64.00	48.14	45
IRC_IRT_c7834	121	YP_009054703.1	0.1	-	52.00	35.04	40
IRC_IRT_c7838	103	EEC71572.1	0.0	GO:0009536	97.00	73.56	34
IRC_IRT_c7840	220	EEE50471.1	0.0	GO:0005840	100.00	90.89	67
IRC_IRT_c7849	149	XP_011016747.1	0.0	GO:0050660	86.00	77.03	46
IRC_IRT_c7858	228	ABF97440.1	0.0	GO:0004523	96.00	113.24	53
IRC_IRT_c7866	115	BAK02198.1	0.0	-	64.00	36.97	34
IRC_IRT_c7873	156	ABF93727.1	0.0	GO:0004523	100.00	72.02	34
IRC_IRT_c7894	130	BAD28818.1	0.0	-	95.00	43.90	41
IRC_IRT_c7914	162	KFM27067.1	0.0	GO:0008750	83.00	75.10	54
IRC_IRT_c7916	158	CAH66219.1	0.0	GO:0004523	89.00	70.09	37
IRC_IRT_c7919	131	CAE04150.1	0.0	GO:0030570	90.00	89.35	4.30E+01
IRC_IRT_c7921	106	NP_001047118.2	0.0	GO:0005739	100.00	82.42	34
IRC_IRT_c7928	111	XP_007015172.1	0.0	GO:0032259	82.00	58.92	35
IRC_IRT_c7943	145	XP_001771825.1	0.0	GO:0005737	88.00	78.95	45
IRC_IRT_c7944	280	XP_010042352.1	0.3	-	55.00	35.04	58
IRC_IRT_c7963	383	EPS74494.1	0.0	-	71.00	56.23	46
IRC_IRT_c7982	156	XP_005648590.1	0.4	-	56.00	33.88	39
IRC_IRT_c8040	108	EEC80776.1	0.0	GO:0042450	80.00	56.23	35
IRC_IRT_c8041	115	XP_001692833.1	0.0	-	62.00	40.05	37
IRC_IRT_c8047	114	XP_002950545.1	0.2	-	60.00	33.88	38
IRC_IRT_c8068	114	XP_002534739.1	0.0	GO:0070918	80.00	49.29	35
IRC_IRT_c8073	138	EEC76877.1	0.0	GO:0046961	92.00	64.70	38
IRC_IRT_c8079	116	XP_002536203.1	0.0	-	68.00	43.13	38
IRC_IRT_c8085	198	XP_006482124.1	0.0	-	54.00	44.28	50
IRC_IRT_c8104	111	KDD71778.1	0.0	GO:0005525	80.00	49.29	36
IRC_IRT_c8134	149	P17784.2	0.0	GO:0005737	100.00	108.61	49
IRC_IRT_c8135	106	CEF97491.1	0.0	GO:0006457	82.00	46.60	35
IRC_IRT_c8158	170	XP_002538463.1	0.0	GO:0006732	74.00	73.94	54
IRC_IRT_c8161	102	XP_003063226.1	0.0	GO:0016491	67.00	43.13	34

IRC_IRT_c8168	177	XP_003560751.2	0.0	GO:0009536	58.00	45.44	53
IRC_IRT_c8171	108	XP_002505919.1	0.0	GO:0005618	97.00	62.00	35
IRC_IRT_c8172	106	CAE04358.1	0.0	GO:0009536	79.00	54.68	34
IRC_IRT_c8173	122	XP_002322900.2	0.7	-	58.00	32.73	34
IRC_IRT_c8185	131	XP_005646636.1	0.6	-	64.00	32.34	34
IRC_IRT_c8187	262	XP_003607358.1	0.0	-	61.00	47.75	49
IRC_IRT_c8188	178	ABA06483.1	0.0	-	55.00	37.35	49
IRC_IRT_c8205	106	XP_004308989.2	0.0	-	97.00	70.09	35
IRC_IRT_c8215	186	KIZ06574.1	0.0	GO:0019427	95.00	98.21	49
IRC_IRT_c8221	121	EEE52320.1	0.0	GO:0055085	84.00	65.86	39
IRC_IRT_c8226	102	AAP68351.1	0.0	GO:0030529	76.00	39.28	34
IRC_IRT_c8240	112	XP_002535829.1	0.0	-	64.00	40.82	37
IRC_IRT_c8257	121	XP_008664856.1	0.0	-	64.00	41.59	34
IRC_IRT_c8264	121	ABA97665.1	0.0	GO:0003677	100.00	75.87	38
IRC_IRT_c8266	112	EAZ02189.1	0.0	-	64.00	41.20	37
IRC_IRT_c8269	153	XP_009350057.1	0.0	-	75.00	50.06	37
IRC_IRT_c8272	143	XP_001700672.1	0.0	GO:1901363	69.00	52.37	49
IRC_IRT_c8276	125	EEC66960.1	0.0	-	67.00	40.05	34
IRC_IRT_c8306	149	KEH15547.1	0.0	GO:0003995	76.00	50.06	39
IRC_IRT_c8325	148	XP_004307365.1	0.9	-	57.00	32.73	38
IRC_IRT_c8330	104	XP_002536223.1	0.2	-	64.00	33.88	34
IRC_IRT_c8353	135	BAA06834.1	0.9	-	60.00	32.34	43
IRC_IRT_c8360	110	NP_001175304.1	0.0	-	91.00	64.70	34
IRC_IRT_c8364	122	EXC20357.1	0.0	-	55.00	36.58	34
IRC_IRT_c8368	167	XP_004958607.1	0.0	-	64.00	41.97	45
IRC_IRT_c8379	238	BAA75236.1	0.0	GO:0003964	100.00	166.78	79
IRC_IRT_c8396	137	AAC49219.1	0.0	GO:0009812	100.00	53.14	45
IRC_IRT_c8404	104	EDQ49172.1	0.4	-	70.00	33.11	34
IRC_IRT_c8418	138	XP_004964759.1	0.4	-	61.00	33.88	36
IRC_IRT_c8421	138	XP_006347707.1	0.0	GO:0009295	90.00	78.18	43
IRC_IRT_c8434	226	EEC78702.1	0.0	GO:0016020	75.00	91.66	74
IRC_IRT_c8439	171	XP_002509391.1	0.0	GO:0003824	64.00	54.30	53
IRC_IRT_c8445	228	EEC67307.1	0.0	GO:0005739	81.00	125.95	75

IRC_IRT_c8450	107	BAD05441.1	0.0	-	74.00	37.74	35
IRC_IRT_c8457	147	EAY97777.1	0.0	GO:0071704	77.00	58.92	48
IRC_IRT_c8458	216	EMT21610.1	0.7	-	58.00	33.11	41
IRC_IRT_c8459	125	XP_001787120.1	0.0	GO:0005737	85.00	61.62	41
IRC_IRT_c8465	192	ABG66149.1	0.0	GO:0004523	94.00	115.55	57
IRC_IRT_c8470	142	XP_011465219.1	0.0	-	57.00	42.36	47
IRC_IRT_c8481	152	AFH58741.1	0.1	-	50.00	35.04	46
IRC_IRT_c8485	118	KDD71936.1	0.5	-	56.00	32.34	37
IRC_IRT_c8508	108	XP_002537046.1	0.0	-	94.00	61.23	35
IRC_IRT_c8509	157	XP_002539889.1	0.2	-	57.00	34.27	38
IRC_IRT_c8520	102	BAD17481.1	0.4	-	52.00	31.19	34
IRC_IRT_c8524	131	XP_002537144.1	0.0	-	71.00	41.20	38
IRC_IRT_c8527	115	XP_007027455.1	0.2	-	44.00	33.88	38
IRC_IRT_c8530	151	EAY93337.1	0.0	-	57.00	41.20	42
IRC_IRT_c8549	144	DAA38572.1	0.1	-	61.00	36.19	34
IRC_IRT_c8559	178	AAV44205.1	0.0	-	70.00	41.59	34
IRC_IRT_c8563	103	XP_002535732.1	0.0	-	79.00	40.43	34
IRC_IRT_c8579	122	KGN61861.1	0.2	-	71.00	33.88	35
IRC_IRT_c8587	163	XP_009408822.1	0.9	-	47.00	32.34	53
IRC_IRT_c8588	132	XP_002538285.1	0.2	-	62.00	33.88	35
IRC_IRT_c8602	213	XP_009351046.1	0.0	-	64.00	64.70	65
IRC_IRT_c8606	187	CAA49327.1	0.5	-	62.00	31.96	37
IRC_IRT_c8607	138	XP_002467719.1	0.8	-	55.00	32.73	40
IRC_IRT_c8613	149	CAB87626.1	0.0	GO:0051287	78.00	74.33	47
IRC_IRT_c8633	181	KJB31094.1	0.0	-	70.00	39.28	34
IRC_IRT_c8638	261	EPS74531.1	0.0	-	74.00	55.45	39
IRC_IRT_c8646	149	EEC70458.1	0.0	GO:0006355	100.00	72.40	35
IRC_IRT_c8657	114	EEC78702.1	0.3	-	73.00	33.88	34
IRC_IRT_c8660	108	KDO46216.1	0.0	GO:0008270	76.00	40.82	34
IRC_IRT_c8667	126	ADZ15222.1	0.1	-	67.00	35.42	34
IRC_IRT_c8673	139	XP_005843919.1	0.0	GO:0006779	75.00	55.45	45
IRC_IRT_c8685	173	EEC66865.1	0.0	GO:0003723	91.00	65.47	36
IRC_IRT_c8689	157	XP_002539401.1	0.0	-	69.00	48.52	49

IRC_IRT_c8712	173	NP_001043700.1	0.0	GO:0046872	100.00	104.76	47
IRC_IRT_c8722	164	AAF16526.1	0.0	GO:0007010	88.00	60.46	34
IRC_IRT_c8733	113	ABA93880.1	0.0	GO:0004523	100.00	77.80	37
IRC_IRT_c8769	134	XP_002538319.1	0.1	-	56.00	34.65	44
IRC_IRT_c8785	135	XP_001758613.1	0.0	-	65.00	36.58	35
IRC_IRT_c8789	113	EYU41510.1	0.2	-	63.00	34.27	41
IRC_IRT_c8795	157	XP_009350076.1	0.0	GO:0009941	94.00	93.20	51
IRC_IRT_c8806	146	P53385.1	0.0	GO:0019557	93.00	92.43	48
IRC_IRT_c8807	107	KFM28067.1	0.0	GO:0005737	91.00	62.00	35
IRC_IRT_c8808	154	XP_009762942.1	0.0	GO:0008760	76.00	66.24	47
IRC_IRT_c8816	138	XP_009351101.1	0.0	GO:0016787	95.00	88.20	45
IRC_IRT_c8819	160	XP_002489151.1	0.0	-	88.00	57.00	34
IRC_IRT_c8820	126	KFM25875.1	0.0	GO:0008152	77.00	54.30	40
IRC_IRT_c8828	187	ERM97545.1	0.9	-	52.00	30.80	40
IRC_IRT_c8836	106	XP_002535009.1	0.9	-	65.00	32.34	35
IRC_IRT_c8851	155	XP_007146093.1	0.0	-	70.00	40.43	34
IRC_IRT_c8861	110	XP_003058143.1	0.0	-	72.00	38.89	36
IRC_IRT_c8873	110	XP_010932557.1	0.7	-	70.00	32.73	34
IRC_IRT_c8875	183	BAD03162.1	0.0	GO:0050660	96.00	122.87	58
IRC_IRT_c8879	113	KDO60187.1	0.1	-	75.00	35.81	37
IRC_IRT_c8891	212	XP_010237678.1	0.3	-	43.00	34.65	65
IRC_IRT_c8894	135	EAY93131.1	0.0	GO:0009536	75.00	59.31	44
IRC_IRT_c8917	126	CCO17359.1	0.0	GO:0044699	76.00	47.37	34
IRC_IRT_c8918	160	XP_002539909.1	0.0	-	69.00	38.89	36
IRC_IRT_c8919	150	BAD87500.1	0.0	GO:0016020	91.00	68.55	36
IRC_IRT_c8929	143	ABY55188.1	0.0	-	65.00	36.19	35
IRC_IRT_c8940	123	NP_001053302.1	0.1	-	64.00	34.65	34
IRC_IRT_c8961	131	XP_002539090.1	0.0	GO:0004872	100.00	85.50	43
IRC_IRT_c8970	139	EEC80776.1	0.0	GO:0016020	65.00	43.51	46
IRC_IRT_c8980	123	EYU31775.1	0.3	-	65.00	33.50	40
IRC_IRT_c8998	116	XP_002539526.1	0.0	-	66.00	40.05	36
IRC_IRT_c9004	103	XP_006468655.1	0.0	GO:0016798	73.00	46.21	34
IRC_IRT_c9006	116	XP_009335667.1	0.7	-	52.00	32.73	36

IRC_IRT_c9015	102	XP_004961199.1	0.1	-	70.00	35.04	34
IRC_IRT_c9023	188	XP_010910728.1	0.9	-	61.00	33.11	36
IRC_IRT_c9027	143	XP_004965711.1	0.0	-	83.00	40.05	36
IRC_IRT_c9028	117	CAE02411.2	0.0	GO:0003964	97.00	68.17	35
IRC_IRT_c9036	139	NP_001055762.1	0.0	GO:0046982	73.00	68.17	52
IRC_IRT_c9039	118	CDP05426.1	0.8	-	64.00	32.73	34
IRC_IRT_c9077	134	NP_001050533.1	0.0	GO:0008270	94.00	65.86	34
IRC_IRT_c9084	126	XP_011100710.1	0.0	-	64.00	40.43	39
IRC_IRT_c9091	327	ABA94348.1	0.0	GO:0004523	95.00	142.51	85
IRC_IRT_c9133	106	EEC77102.1	0.2	-	73.00	33.88	38
IRC_IRT_c9141	126	XP_002960091.1	0.0	-	55.00	36.58	38
IRC_IRT_c9167	241	AGC78945.1	0.0	-	44.00	38.12	81
IRC_IRT_c9180	170	XP_002537224.1	0.0	-	64.00	50.45	56
IRC_IRT_c9184	190	XP_009351044.1	0.0	-	66.00	60.46	63
IRC_IRT_c9189	157	EMT18568.1	0.0	GO:0016787	75.00	67.01	48
IRC_IRT_c9192	130	XP_002270953.1	0.9	-	45.00	31.96	37
IRC_IRT_c9195	315	AAK13123.1	0.0	GO:0003964	85.00	67.78	35
IRC_IRT_c9211	110	XP_005650918.1	0.0	-	70.00	43.13	37
IRC_IRT_c9216	122	XP_011014252.1	0.0	-	67.00	46.21	37
IRC_IRT_c9231	164	EAY85944.1	0.5	-	56.00	31.96	37
IRC_IRT_c9242	125	XP_003055236.1	0.1	-	64.00	35.04	37
IRC_IRT_c9245	129	CAE04056.2	0.0	GO:0003676	100.00	91.28	42
IRC_IRT_c9250	115	ABR16443.1	0.0	-	65.00	43.13	35
IRC_IRT_c9257	142	XP_009351044.1	0.0	-	68.00	43.13	41
IRC_IRT_c9258	145	BAJ93922.1	0.0	GO:0016301	65.00	41.59	3.50E+01
IRC_IRT_c9262	125	XP_002538319.1	0.0	GO:0009987	60.00	43.51	40
IRC_IRT_c9265	106	AAC49170.1	0.0	GO:0004812	76.00	49.68	34
IRC_IRT_c9303	115	EEE67461.1	0.0	GO:0016023	100.00	79.34	37
IRC_IRT_c9304	123	NP_001051662.1	0.0	GO:0005524	100.00	82.42	40
IRC_IRT_c9306	155	CDO97095.1	0.8	-	48.00	32.73	41
IRC_IRT_c9325	118	ADU04388.1	0.0	GO:0016887	92.00	65.47	39
IRC_IRT_c9331	138	AAT76354.1	0.0	GO:0004523	94.00	75.49	38
IRC_IRT_c9368	115	XP_002534637.1	0.0	-	82.00	57.38	35

IRC_IRT_c9385	230	XP_010459435.1	0.1	-	54.00	35.04	50
IRC_IRT_c9386	153	XP_002272942.2	0.1	-	55.00	35.42	40
IRC_IRT_c9388	120	XP_005849584.1	0.2	-	55.00	34.27	38
IRC_IRT_c9405	105	EAY93345.1	0.0	GO:0008152	100.00	73.17	35
IRC_IRT_c9415	148	EAZ14682.1	0.0	GO:0043531	94.00	69.32	34
IRC_IRT_c9419	116	ABA97569.1	0.0	GO:0003676	86.00	68.17	38
IRC_IRT_c9432	132	XP_009121446.1	0.7	-	54.00	32.34	35
IRC_IRT_c9441	142	CAE03226.1	0.0	-	60.00	46.21	4.60E+01
IRC_IRT_c9461	146	EEE61933.1	0.3	-	62.00	33.50	35
IRC_IRT_c9483	117	XP_003605617.1	0.0	-	69.00	41.20	36
IRC_IRT_c9484	222	XP_001785975.1	0.0	-	72.00	57.38	43
IRC_IRT_c9492	168	BAD30751.1	0.0	GO:0008234	80.00	87.04	55
IRC_IRT_c9507	157	XP_005851307.1	0.0	GO:0015992	75.00	55.45	48
IRC_IRT_c9520	121	XP_006649959.1	0.0	GO:0050789	70.00	43.13	37
IRC_IRT_c9523	122	ABK23920.1	0.2	-	70.00	33.50	34
IRC_IRT_c9528	140	XP_008455715.1	0.0	-	65.00	42.36	46
IRC_IRT_c9540	252	XP_001786687.1	0.0	GO:0005215	80.00	51.60	35
IRC_IRT_c9547	125	EPS72996.1	0.0	GO:0008152	65.00	47.37	41
IRC_IRT_c9564	136	EAZ16901.1	0.0	GO:0005739	100.00	92.43	45
IRC_IRT_c9565	105	EAY73930.1	0.0	GO:0016023	97.00	70.86	34
IRC_IRT_c9566	127	ABA99634.1	0.0	GO:0003676	100.00	85.11	42
IRC_IRT_c9591	135	AAW33880.1	0.5	-	53.00	33.11	43
IRC_IRT_c9607	180	AGO06529.1	0.0	GO:0008233	80.00	76.64	51
IRC_IRT_c9612	160	NP_001046951.2	0.0	GO:0016023	70.00	61.62	47
IRC_IRT_c9616	147	AFW59033.1	0.0	GO:0003676	68.00	45.82	50
IRC_IRT_c9620	120	XP_008226643.1	0.0	-	72.00	45.05	36
IRC_IRT_c9638	119	DAA51225.1	0.0	GO:0005737	97.00	78.57	34
IRC_IRT_c9642	138	XP_004291320.2	0.0	-	62.00	43.51	45
IRC_IRT_c9661	114	XP_002536736.1	0.0	GO:0006950	97.00	71.25	37
IRC_IRT_c9668	202	EEC76122.1	0.0	GO:0006139	53.00	45.44	64
IRC_IRT_c9675	145	XP_008448444.1	0.0	-	68.00	41.20	35
IRC_IRT_c9677	180	AFF19043.1	0.9	-	55.00	32.73	38
IRC_IRT_c9687	130	YP_006280981.1	0.0	-	68.00	38.12	35

IRC_IRT_c9694	155	BAB63679.1	0.0	GO:0005739	100.00	81.65	51
IRC_IRT_c9699	149	BAC84555.1	0.0	-	92.00	53.14	38
IRC_IRT_c9700	107	BAD28273.1	0.0	-	80.00	61.62	35
IRC_IRT_c9702	120	XP_001786746.1	0.0	-	61.00	36.97	39
IRC_IRT_c9711	165	XP_002893850.1	0.2	-	61.00	35.04	44
IRC_IRT_c9714	131	XP_011016603.1	0.0	GO:0004591	86.00	73.94	43
IRC_IRT_c9715	120	XP_001786850.1	0.0	-	70.00	34.65	40
IRC_IRT_c9722	128	AAX94990.1	0.0	GO:0006468	90.00	68.17	41
IRC_IRT_c9725	153	BAD81703.1	0.0	-	79.00	56.23	39
IRC_IRT_c9726	144	XP_003074462.1	0.0	GO:0017038	75.00	64.70	48
IRC_IRT_c9758	115	XP_011016366.1	0.0	GO:0006096	97.00	81.26	38
IRC_IRT_c9775	112	BAJ99586.1	1.0	-	54.00	31.96	35
IRC_IRT_c9782	205	XP_008654561.1	0.3	-	47.00	34.65	67
IRC_IRT_c9795	173	KDD76199.1	0.0	GO:1901363	76.00	80.88	60
IRC_IRT_c9798	114	NP_001066388.2	0.0	GO:0046933	100.00	76.64	37
IRC_IRT_c9799	127	XP_005846776.1	0.0	GO:0008152	82.00	52.76	34
IRC_IRT_c9808	130	XP_002536228.1	0.0	-	53.00	37.74	43
IRC_IRT_c9812	117	XP_001754678.1	0.2	-	64.00	33.88	37
IRC_IRT_c9840	197	AAQ56433.1	0.0	GO:0004523	98.00	133.27	65
IRC_IRT_c9863	167	XP_002536422.1	0.0	-	73.00	40.05	52
IRC_IRT_c9865	115	XP_002539765.1	0.0	GO:0007059	83.00	47.37	36
IRC_IRT_c9873	125	XP_005844806.1	0.0	GO:0009908	87.00	73.17	40
IRC_IRT_c9880	156	BAJ97633.1	0.0	-	58.00	38.12	41
IRC_IRT_c9893	107	XP_002540139.1	0.0	GO:0030554	85.00	53.91	34
IRC_IRT_c9905	108	EEC77110.1	0.0	-	60.00	37.35	35
IRC_IRT_c9939	118	XP_010911538.1	0.0	-	89.00	63.93	39
IRC_IRT_c9952	188	NP_001142180.1	0.0	GO:0003735	100.00	126.72	6.20E+01
IRC_IRT_c9998	109	EAY76104.1	0.0	GO:0043531	100.00	76.26	36
IRC_IRT_c10007	171	BAD61287.1	0.0	-	82.00	74.71	50
IRC_IRT_c10014	119	XP_005645020.1	0.0	-	71.00	41.97	38
IRC_IRT_c10016	195	AAX96763.1	0.0	GO:0007010	100.00	105.53	47
IRC_IRT_c10048	152	NP_039436.1	0.0	GO:0005739	97.00	92.43	44
IRC_IRT_c10058	157	CAH65891.1	0.0	GO:0003964	100.00	102.83	48

IRC_IRT_c10065	117	XP_002536034.1	0.0	-	85.00	62.00	35
IRC_IRT_c10075	188	XP_002980060.1	0.0	GO:0015854	70.00	57.00	51
IRC_IRT_c10080	151	CAH66816.1	0.0	GO:0003964	100.00	67.01	50
IRC_IRT_c10092	107	EDQ48693.1	0.6	-	60.00	32.73	38
IRC_IRT_c10094	141	BAB62634.1	0.0	-	62.00	41.97	43
IRC_IRT_c10097	138	EXC01912.1	0.0	-	60.00	44.28	45
IRC_IRT_c10123	133	AAM01117.1	0.0	GO:0003964	97.00	93.59	44
IRC_IRT_c10127	125	BAD07842.1	0.0	-	60.00	34.65	40
IRC_IRT_c10128	128	XP_002536324.1	0.0	GO:0016787	78.00	49.68	41
IRC_IRT_c10129	104	XP_002538041.1	0.0	GO:0006564	100.00	65.08	34
IRC_IRT_c10132	141	XP_008444469.1	0.0	-	65.00	39.28	46
IRC_IRT_c10135	126	AGP04885.1	0.0	GO:0036361	79.00	59.31	39
IRC_IRT_c10142	115	XP_002538205.1	0.0	GO:0071704	73.00	59.31	38
IRC_IRT_c10151	182	CDY45505.1	0.0	-	71.00	47.37	39
IRC_IRT_c10169	294	XP_006841758.1	0.1	-	59.00	36.19	49
IRC_IRT_c10200	115	XP_011091044.1	0.0	GO:0004316	80.00	54.68	35
IRC_IRT_c10205	140	XP_008466606.1	0.8	-	61.00	32.73	36
IRC_IRT_c10210	161	EPS70841.1	0.1	-	60.00	35.42	48
IRC_IRT_c10222	106	XP_004510448.1	0.0	GO:0009451	79.00	53.91	34
IRC_IRT_c10245	140	CDY67157.1	0.1	-	61.00	34.65	34
IRC_IRT_c10287	156	ACE82292.1	0.5	-	51.00	32.73	41
IRC_IRT_c10296	113	EEC75989.1	0.0	GO:0045735	100.00	77.03	37
IRC_IRT_c10298	165	XP_006405917.1	0.0	-	72.00	49.68	36
IRC_IRT_c10312	162	XP_002536731.1	0.0	-	66.00	45.44	50
IRC_IRT_c10314	170	AES77606.2	0.0	GO:0006869	84.00	65.47	38
IRC_IRT_c10316	105	XP_002534652.1	0.0	GO:0000155	79.00	49.29	34
IRC_IRT_c10319	221	BAJ11783.1	0.0	-	76.00	65.08	39
IRC_IRT_c10331	106	NP_001175243.1	0.0	GO:0005488	82.00	53.53	35
IRC_IRT_c10335	152	XP_001758646.1	0.0	-	77.00	45.05	45
IRC_IRT_c10342	128	XP_011016366.1	0.0	GO:0005975	74.00	49.68	39
IRC_IRT_c10344	154	AEW22943.1	0.4	-	50.00	34.27	44
IRC_IRT_c10352	120	KDD75314.1	0.0	GO:0016627	65.00	44.28	40
IRC_IRT_c10358	117	XP_006852882.1	0.0	-	68.00	40.43	35

IRC_IRT_c10359	147	XP_008377334.1	0.7	-	52.00	33.11	44
IRC_IRT_c10376	149	XP_009351044.1	0.0	-	72.00	44.28	36
IRC_IRT_c10389	166	AAB82136.1	0.8	-	61.00	32.34	42
IRC_IRT_c10392	110	XP_001421586.1	0.0	-	72.00	42.74	36
IRC_IRT_c10393	258	AAW57787.1	0.0	-	96.00	174.48	82
IRC_IRT_c10406	270	CAH66287.1	0.0	-	97.00	170.63	82
IRC_IRT_c10426	130	ABB47022.2	0.0	GO:0003676	100.00	95.90	43
IRC_IRT_c10438	114	CAE02127.2	0.0	GO:0004523	97.00	79.72	37
IRC_IRT_c10450	134	XP_002539627.1	0.0	-	60.00	36.58	43
IRC_IRT_c10457	166	XP_009775667.1	0.6	-	50.00	33.50	46
IRC_IRT_c10461	247	XP_006443887.1	0.6	-	56.00	33.88	44
IRC_IRT_c10463	272	AIC76501.1	0.6	-	44.00	34.27	65
IRC_IRT_c10465	261	XP_002489033.1	0.0	-	71.00	49.29	39
IRC_IRT_c10467	167	KJB44141.1	0.0	-	66.00	49.29	51
IRC_IRT_c10479	207	AAV44205.1	0.0	-	65.00	45.44	46
IRC_IRT_c10483	383	BAJ11780.1	0.0	-	77.00	47.37	35
IRC_IRT_c10485	281	XP_011070585.1	0.2	-	51.00	35.81	58
IRC_IRT_c10517	148	ADE76966.1	0.0	-	63.00	50.06	38
IRC_IRT_c10518	218	CAE04764.3	0.0	GO:0005840	100.00	104.76	49
IRC_IRT_c10531	313	ADK60808.1	0.0	-	62.00	50.83	51
IRC_IRT_c10551	157	XP_002539818.1	0.0	-	63.00	41.20	52
IRC_IRT_c10552	132	EMT20296.1	0.3	-	54.00	33.50	44
IRC_IRT_c10568	108	EAY93131.1	0.0	GO:0000166	77.00	46.98	35
IRC_IRT_c10579	106	XP_003079372.1	0.0	-	61.00	36.19	34
IRC_IRT_c10586	122	CDP19707.1	0.8	-	61.00	32.73	34
IRC_IRT_c10591	133	ABL98118.1	0.0	-	54.00	38.89	44
IRC_IRT_c10623	112	XP_002974017.1	0.0	-	57.00	40.43	38
IRC_IRT_c10634	104	AHZ00636.1	0.0	GO:0004399	79.00	45.05	34
IRC_IRT_c10636	261	XP_003616487.1	0.9	-	40.00	33.50	92
IRC_IRT_c10649	124	XP_001761531.1	0.0	GO:0005975	84.00	64.70	38
IRC_IRT_c10652	143	AAS98503.1	0.0	GO:0047911	89.00	87.81	47
IRC_IRT_c10660	178	XP_001756901.1	0.0	GO:0090599	55.00	47.75	5.40E+01
IRC_IRT_c10662	140	XP_005844530.1	0.0	GO:0016874	72.00	42.36	36

IRC_IRT_c10664	133	EAY94487.1	0.0	GO:0043531	100.00	90.12	40
IRC_IRT_c10666	153	XP_007017071.1	0.0	GO:0055114	77.00	62.39	40
IRC_IRT_c10675	302	BAD09499.1	0.0	GO:0090502	93.00	92.43	48
IRC_IRT_c10694	143	EAY93131.1	0.0	GO:0009507	80.00	50.06	46
IRC_IRT_c10700	107	EPS67550.1	0.0	GO:0045893	91.00	62.77	35
IRC_IRT_c10702	138	BAD01321.1	0.0	GO:0005739	80.00	59.69	45
IRC_IRT_c10707	153	CDP16399.1	0.0	GO:0016491	66.00	46.60	51
IRC_IRT_c10745	160	XP_005647342.1	0.0	GO:0005737	80.00	70.86	51
IRC_IRT_c10750	158	EAY84569.1	0.0	GO:0016627	78.00	81.26	51
IRC_IRT_c10752	122	EAY80077.1	0.0	GO:0006952	100.00	87.81	40
IRC_IRT_c10764	113	BAD87478.1	0.0	-	91.00	62.77	36
IRC_IRT_c10769	112	XP_002538398.1	0.0	GO:0004871	78.00	47.75	37
IRC_IRT_c10784	177	NP_001176336.1	0.0	GO:0005840	100.00	102.83	50
IRC_IRT_c10789	136	XP_002438565.1	0.8	-	45.00	32.34	40
IRC_IRT_c10798	122	EEC68634.1	0.0	GO:0016787	76.00	54.30	34
IRC_IRT_c10800	146	KIZ00800.1	0.7	-	55.00	32.73	36
IRC_IRT_c10805	113	AEQ39045.1	0.0	GO:0046872	100.00	76.64	37
IRC_IRT_c10809	133	XP_002536731.1	0.0	GO:0016829	69.00	45.44	36
IRC_IRT_c10825	160	EMS60773.1	0.0	GO:0016021	95.00	78.95	41
IRC_IRT_c10828	147	XP_006430897.1	0.5	-	54.00	33.50	37
IRC_IRT_c10855	136	XP_009350806.1	0.0	GO:0044763	86.00	70.86	44
IRC_IRT_c10870	236	AAY23262.1	0.0	GO:0003723	97.00	107.46	70
IRC_IRT_c10882	106	XP_006478099.1	0.0	-	64.00	43.13	34
IRC_IRT_c10885	156	BAK02440.1	0.0	GO:0020037	84.00	76.26	46
IRC_IRT_c10904	227	BAB33421.1	0.0	-	88.00	91.66	50
IRC_IRT_c10928	109	XP_002537036.1	0.0	GO:0006810	86.00	59.69	36
IRC_IRT_c10930	158	KCW90449.1	0.3	-	60.00	33.88	40
IRC_IRT_c10933	167	BAI39691.1	0.0	GO:0004523	94.00	70.48	35
IRC_IRT_c10939	204	CCO18629.1	0.0	GO:0016874	66.00	66.63	68
IRC_IRT_c10940	108	XP_002958615.1	0.0	GO:0004252	91.00	50.06	34
IRC_IRT_c10959	139	ABA99227.1	0.0	GO:0003964	93.00	83.96	46
IRC_IRT_c10965	137	XP_010238593.1	0.0	-	58.00	38.12	41
IRC_IRT_c10986	215	XP_002535949.1	0.0	-	75.00	51.22	40

IRC_IRT_c11004	114	XP_001415394.1	0.0	GO:0008152	73.00	58.54	38
IRC_IRT_c11022	216	CAH66140.1	0.0	GO:0003676	98.00	113.62	58
IRC_IRT_c11023	109	EMT00243.1	0.0	GO:0005488	76.00	53.14	34
IRC_IRT_c11033	142	XP_007203712.1	0.8	-	67.00	32.34	34
IRC_IRT_c11043	117	AFK40600.1	0.0	GO:0016620	78.00	51.99	38
IRC_IRT_c11057	188	XP_002538072.1	0.0	GO:0043565	96.00	113.24	58
IRC_IRT_c11096	126	XP_011085255.1	0.0	GO:0016151	87.00	63.93	41
IRC_IRT_c11107	133	XP_009803798.1	0.6	-	43.00	32.73	44
IRC_IRT_c11116	120	XP_009416285.1	0.1	-	58.00	36.19	39
IRC_IRT_c11128	196	KIZ05001.1	0.0	-	49.00	40.82	61
IRC_IRT_c11130	126	XP_002953811.1	0.0	GO:0044763	74.00	51.22	39
IRC_IRT_c11136	118	KEH15344.1	0.0	-	57.00	37.74	38
IRC_IRT_c11140	130	CAC39079.1	0.0	-	93.00	82.03	43
IRC_IRT_c11148	155	XP_002870194.1	0.4	-	50.00	31.96	48
IRC_IRT_c11161	166	KFM22494.1	0.0	GO:0000166	75.00	57.38	49
IRC_IRT_c11191	147	XP_011077001.1	0.0	GO:0006633	78.00	61.23	42
IRC_IRT_c11193	150	XP_003595724.1	0.5	-	52.00	32.73	40
IRC_IRT_c11194	157	XP_003575363.1	0.2	-	64.00	35.04	34
IRC_IRT_c11200	108	NP_001052008.1	0.0	GO:0016021	100.00	77.41	36
IRC_IRT_c11213	135	XP_002538230.1	0.0	GO:0004872	86.00	59.31	44
IRC_IRT_c11218	213	AAT85162.1	0.0	GO:0003964	92.00	150.98	70
IRC_IRT_c11219	121	NP_001043974.1	0.0	GO:0003677	97.00	85.11	4.00E+01
IRC_IRT_c11235	111	XP_008371467.1	0.0	GO:0055114	80.00	55.07	36
IRC_IRT_c11254	104	KFM23134.1	0.0	GO:0009536	73.00	50.06	34
IRC_IRT_c11264	178	ABF95973.1	0.0	GO:0003964	91.00	115.93	60
IRC_IRT_c11265	114	EAZ04277.1	0.0	GO:0006468	100.00	47.37	36
IRC_IRT_c11267	107	ACL99707.1	0.0	GO:0004129	80.00	59.31	35
IRC_IRT_c11280	142	XP_002979914.1	0.0	GO:0009295	80.00	68.94	47
IRC_IRT_c11306	179	KFK24393.1	0.0	-	60.00	35.42	43
IRC_IRT_c11313	137	XP_001786580.1	0.0	-	65.00	36.19	43
IRC_IRT_c11319	126	XP_010940487.1	0.0	-	62.00	36.58	35
IRC_IRT_c11321	119	KCW68323.1	0.0	-	55.00	36.19	36
IRC_IRT_c11334	131	ABI54186.1	0.0	-	62.00	43.90	37

IRC_IRT_c11336	122	XP_006663494.1	0.0	GO:0009536	89.00	68.17	39
IRC_IRT_c11370	143	XP_002536181.1	0.0	GO:0004871	72.00	60.08	48
IRC_IRT_c11434	106	XP_010413914.1	0.0	-	73.00	40.05	34
IRC_IRT_c11435	142	XP_010053673.1	0.1	-	60.00	35.42	51
IRC_IRT_c11453	130	KJB73689.1	0.0	-	58.00	40.82	41
IRC_IRT_c11472	127	AEI00875.1	0.0	GO:0004129	88.00	62.00	42
IRC_IRT_c11482	121	XP_002980311.1	0.0	GO:0044249	77.00	43.51	35
IRC_IRT_c11493	104	XP_002953904.1	0.0	GO:0006457	82.00	54.30	34
IRC_IRT_c11510	116	XP_002537492.1	0.0	GO:0044710	78.00	57.00	37
IRC_IRT_c11519	135	KIY94406.1	0.0	-	72.00	44.67	40
IRC_IRT_c11550	108	CAE03019.3	0.0	GO:0003676	100.00	72.02	35
IRC_IRT_c11560	133	XP_008794857.1	0.4	-	52.00	33.50	36
IRC_IRT_c11561	147	BAD03661.1	0.0	GO:0090502	100.00	46.60	37
IRC_IRT_c11571	135	XP_005849242.1	0.1	-	62.00	35.04	35
IRC_IRT_c11580	113	XP_005844736.1	0.0	GO:1901576	70.00	50.83	37
IRC_IRT_c11610	152	ABC72668.1	0.4	-	60.00	33.50	43
IRC_IRT_c11622	133	EEC68471.1	0.8	-	52.00	32.73	44
IRC_IRT_c11624	106	AAF16526.1	0.0	GO:0007010	94.00	73.94	35
IRC_IRT_c11653	172	XP_005850918.1	0.0	GO:0006499	82.00	87.43	57
IRC_IRT_c11655	117	AAN11196.1	0.0	GO:0005739	94.00	65.08	36
IRC_IRT_c11659	142	EMS67619.1	0.1	-	57.00	35.81	47
IRC_IRT_c11661	123	ABA97728.1	0.0	GO:0005840	95.00	83.96	41
IRC_IRT_c11665	163	XP_002489152.1	0.0	-	82.00	66.24	41
IRC_IRT_c11696	129	XP_009350812.1	0.0	-	61.00	46.21	39
IRC_IRT_c11705	123	AAF16526.1	0.0	GO:0007010	100.00	84.73	40
IRC_IRT_c11707	114	EDQ48461.1	0.0	-	64.00	43.13	37
IRC_IRT_c11709	159	XP_002501393.1	0.0	-	64.00	42.36	34
IRC_IRT_c11710	120	AAX96091.1	0.0	-	82.00	61.62	3.90E+01
IRC_IRT_c11715	148	XP_001701046.1	0.0	-	61.00	50.06	49
IRC_IRT_c11721	107	XP_001771969.1	0.0	GO:0004871	73.00	47.37	38
IRC_IRT_c11728	109	CDY26307.1	0.0	-	75.00	37.74	36
IRC_IRT_c11732	132	CAE03841.1	0.0	GO:0005739	88.00	62.00	36
IRC_IRT_c11747	168	KDO78148.1	0.0	GO:0043167	75.00	69.32	49

IRC_IRT_c11759	108	AAV25239.1	0.0	-	85.00	57.77	34
IRC_IRT_c11761	110	AFF18853.1	0.0	GO:0050661	82.00	54.68	34
IRC_IRT_c11800	111	XP_002538756.1	0.0	GO:0006633	78.00	47.37	37
IRC_IRT_c11804	158	BAJ21440.1	0.0	-	60.00	40.43	41
IRC_IRT_c11805	123	XP_002535080.1	0.2	-	63.00	34.27	38
IRC_IRT_c11817	106	XP_002539330.1	0.0	-	85.00	63.93	35
IRC_IRT_c11829	213	CAH65837.1	0.0	GO:0004523	97.00	95.13	47
IRC_IRT_c11849	138	XP_004142102.1	0.0	GO:0016021	71.00	53.14	42
IRC_IRT_c11850	197	EAZ23222.1	0.8	-	64.00	33.11	34
IRC_IRT_c11872	201	EMT24082.1	0.4	-	64.00	34.27	37
IRC_IRT_c11897	125	AFK34218.1	0.0	GO:0004013	80.00	56.61	36
IRC_IRT_c11898	107	XP_011016747.1	0.0	GO:0050660	97.00	67.78	35
IRC_IRT_c11927	140	XP_001690840.1	0.0	GO:0005488	67.00	51.99	4.30E+01
IRC_IRT_c11929	140	EEC73650.1	1.0	-	54.00	32.34	46
IRC_IRT_c11930	264	XP_007157890.1	0.0	-	56.00	39.66	48
IRC_IRT_c11953	249	XP_001758940.1	0.0	GO:0006355	79.00	76.26	59
IRC_IRT_c11955	192	KJB15228.1	0.0	-	79.00	51.60	34
IRC_IRT_c11966	111	AAU44317.1	0.1	-	61.00	35.81	39
IRC_IRT_c11980	106	EAY78349.1	0.0	GO:0016788	100.00	74.33	35
IRC_IRT_c11990	111	XP_010468863.1	0.0	-	75.00	49.29	36
IRC_IRT_c12000	233	XP_002536962.1	0.0	-	62.00	77.41	74
IRC_IRT_c12011	136	EDQ48154.1	0.0	GO:0006631	72.00	57.38	43
IRC_IRT_c12018	114	ABA97611.1	0.0	GO:0003676	83.00	50.45	37
IRC_IRT_c12023	139	BAJ94909.1	0.0	-	61.00	40.43	42
IRC_IRT_c12027	125	XP_002535669.1	0.0	-	63.00	40.82	41
IRC_IRT_c12035	113	AAT07598.1	0.0	-	68.00	42.74	35
IRC_IRT_c12036	180	XP_007213124.1	0.6	-	50.00	33.11	57
IRC_IRT_c12037	126	BAD62223.1	0.0	-	82.00	63.54	41
IRC_IRT_c12043	188	EAY87758.1	0.8	-	46.00	33.11	56
IRC_IRT_c12070	170	AFW89829.1	0.4	-	70.00	33.88	37
IRC_IRT_c12086	114	XP_002977223.1	0.0	-	69.00	40.43	36
IRC_IRT_c12087	173	CAD40517.1	0.4	-	83.00	33.88	36
IRC_IRT_c12106	150	KEH15665.1	0.0	-	61.00	43.51	47

IRC_IRT_c12107	122	XP_002960029.1	0.0	GO:0016740	76.00	55.84	38
IRC_IRT_c12108	109	NP_001146431.1	0.0	GO:0006810	100.00	78.57	36
IRC_IRT_c12153	152	EPS58403.1	0.0	-	61.00	40.82	47
IRC_IRT_c12166	104	KIY98699.1	0.0	GO:0003849	76.00	54.68	34
IRC_IRT_c12177	157	XP_002528242.1	0.0	-	65.00	38.12	49
IRC_IRT_c12192	131	ABA95225.1	0.0	GO:0003964	97.00	76.64	43
IRC_IRT_c12201	141	XP_003599576.1	0.1	-	56.00	34.65	37
IRC_IRT_c12215	109	XP_002534890.1	0.0	-	71.00	42.36	35
IRC_IRT_c12217	130	XP_002954321.1	0.0	-	58.00	40.82	41
IRC_IRT_c12228	103	ABF94049.1	0.0	GO:0003735	100.00	70.09	34
IRC_IRT_c12252	112	KIZ07386.1	0.4	-	62.00	33.11	37
IRC_IRT_c12253	113	XP_003058059.1	0.0	GO:0005515	69.00	41.59	36
IRC_IRT_c12262	166	EPS71095.1	0.0	GO:0044710	62.00	43.51	50
IRC_IRT_c12268	135	CCO14648.1	0.0	-	52.00	41.20	40
IRC_IRT_c12301	144	XP_003081468.1	0.0	-	74.00	43.51	35
IRC_IRT_c12317	126	EDQ48091.1	0.0	-	66.00	44.28	42
IRC_IRT_c12326	115	AFW58586.1	0.2	-	67.00	33.88	37
IRC_IRT_c12353	130	YP_009057497.1	0.0	GO:0044444	68.00	48.52	35
IRC_IRT_c12359	145	EXC01914.1	0.0	-	59.00	45.44	47
IRC_IRT_c12408	164	BAJ90223.1	0.3	-	48.00	33.88	39
IRC_IRT_c12416	138	EDQ48498.1	0.0	-	75.00	47.75	36
IRC_IRT_c12417	119	EMS49554.1	0.0	-	72.00	46.60	36
IRC_IRT_c12422	210	XP_003605652.1	0.0	-	71.00	51.22	42
IRC_IRT_c12438	119	XP_001416314.1	0.0	GO:0006412	72.00	41.20	36
IRC_IRT_c12443	112	EAY93131.1	0.5	-	61.00	32.73	36
IRC_IRT_c12469	120	AFW85628.1	0.4	-	53.00	33.50	41
IRC_IRT_c12484	139	CDP16866.1	0.3	-	58.00	34.27	36
IRC_IRT_c12489	109	ACG45613.1	0.0	GO:0010035	72.00	48.14	36
IRC_IRT_c12493	136	EAY95020.1	0.5	-	50.00	33.50	42
IRC_IRT_c12501	116	XP_002951617.1	0.0	-	67.00	40.82	37
IRC_IRT_c12504	151	XP_001785112.1	0.0	GO:0016301	72.00	55.84	50
IRC_IRT_c12518	103	XP_002536583.1	0.0	-	61.00	38.89	34
IRC_IRT_c12536	143	AHJ80818.1	0.0	GO:0005874	100.00	90.12	37

IRC_IRT_c12544	144	XP_009350069.1	0.0	GO:0043231	68.00	47.37	47
IRC_IRT_c12554	102	XP_005847194.1	0.1	-	70.00	35.42	34
IRC_IRT_c12563	195	AAV31206.1	0.0	-	95.00	92.82	63
IRC_IRT_c12564	186	ABF95899.1	0.0	GO:0003676	98.00	97.83	62
IRC_IRT_c12567	198	AGZ19352.1	0.0	-	56.00	37.74	51
IRC_IRT_c12572	168	BAD23367.1	0.0	-	64.00	41.97	45
IRC_IRT_c12574	109	XP_004984075.1	0.3	-	61.00	33.50	34
IRC_IRT_c12581	279	BAD16168.1	0.0	-	54.00	37.35	53
IRC_IRT_c12584	227	CDY30864.1	0.0	-	67.00	38.12	37
IRC_IRT_c12587	193	CAD39683.1	0.0	GO:0004523	100.00	92.43	43
IRC_IRT_c12613	158	XP_003562358.1	0.0	GO:0005829	76.00	64.70	50
IRC_IRT_c12616	139	CDY19671.1	0.0	-	61.00	40.43	39
IRC_IRT_c12619	202	AAV44205.1	0.0	-	67.00	41.59	34
IRC_IRT_c12623	172	KFM28025.1	0.0	-	58.00	41.59	46
IRC_IRT_c12624	118	CAE17671.1	0.7	-	57.00	32.34	38
IRC_IRT_c12627	131	XP_002537246.1	0.0	GO:0055114	83.00	66.63	42
IRC_IRT_c12639	128	XP_002536281.1	0.0	-	85.00	63.54	41
IRC_IRT_c12647	155	XP_004986817.1	0.3	-	55.00	33.88	38
IRC_IRT_c12686	107	CAE02548.1	0.0	GO:0004523	82.00	59.69	35
IRC_IRT_c12698	141	XP_005645020.1	0.0	-	68.00	42.74	45
IRC_IRT_c12709	116	XP_002536403.1	0.0	-	62.00	35.42	35
IRC_IRT_c12718	136	XP_002536230.1	0.0	-	51.00	39.28	45
IRC_IRT_c12728	138	XP_010239721.1	0.6	-	47.00	32.34	46
IRC_IRT_c12730	109	XP_010911679.1	0.0	GO:0005488	82.00	59.31	35
IRC_IRT_c12733	118	AHL28545.1	0.0	-	64.00	39.28	39
IRC_IRT_c12738	171	CAE05895.1	0.0	GO:0003676	82.00	81.65	50
IRC_IRT_c12744	172	XP_003078976.1	0.0	GO:0019752	65.00	48.91	44
IRC_IRT_c12786	121	XP_002538359.1	0.0	-	62.00	42.74	40
IRC_IRT_c12795	151	XP_002536189.1	0.0	-	61.00	36.58	39
IRC_IRT_c12807	147	XP_003617740.1	0.0	GO:0016021	95.00	76.64	40
IRC_IRT_c12828	111	XP_002965370.1	0.9	-	54.00	32.34	37
IRC_IRT_c12837	134	XP_010908494.1	0.6	-	50.00	33.11	34
IRC_IRT_c12852	147	XP_002534564.1	0.0	GO:0016491	65.00	45.05	46

IRC_IRT_c12854	145	EEC82044.1	0.0	-	100.00	97.06	48
IRC_IRT_c12865	108	XP_005849839.1	0.0	-	65.00	36.58	35
IRC_IRT_c12870	135	BAD82486.1	0.0	-	60.00	37.35	35
IRC_IRT_c12873	119	KEH15415.1	0.0	-	69.00	37.74	36
IRC_IRT_c12878	297	ABH09321.1	0.7	-	61.00	33.88	34
IRC_IRT_c12895	102	XP_002989557.1	0.0	GO:0006807	73.00	46.60	34
IRC_IRT_c12905	142	XP_001786672.1	0.1	-	69.00	35.04	36
IRC_IRT_c12908	129	XP_007039531.1	0.6	-	56.00	32.34	37
IRC_IRT_c12912	169	XP_008447683.1	0.0	-	63.00	38.89	41
IRC_IRT_c12915	138	XP_002538377.1	0.0	-	64.00	45.44	45
IRC_IRT_c12941	121	KFM24025.1	0.0	GO:0008270	84.00	57.77	38
IRC_IRT_c12949	169	XP_001767418.1	0.0	GO:0043168	71.00	73.94	60
IRC_IRT_c12950	191	EXC01914.1	0.0	-	70.00	40.82	34
IRC_IRT_c12956	155	XP_010925406.1	0.8	-	52.00	32.73	40
IRC_IRT_c12969	121	ABA97707.2	0.0	GO:0003677	97.00	86.27	39
IRC_IRT_c12977	166	AAX92776.1	0.0	GO:0003964	94.00	103.22	55
IRC_IRT_c12996	226	XP_008447683.1	0.0	-	58.00	36.19	55
IRC_IRT_c13002	119	XP_006829520.1	0.0	-	72.00	38.89	36
IRC_IRT_c13004	184	XP_002508547.1	0.0	GO:0003676	70.00	48.52	48
IRC_IRT_c13005	215	XP_009388289.1	0.0	-	82.00	53.91	39
IRC_IRT_c13007	139	XP_003607358.1	0.0	-	59.00	40.05	44
IRC_IRT_c13011	167	AAM00958.1	0.0	GO:0044260	85.00	82.80	55
IRC_IRT_c13042	122	EAY73600.1	0.0	GO:0008152	100.00	88.58	40
IRC_IRT_c13059	125	EAZ45042.1	0.0	GO:0016023	90.00	91.28	41
IRC_IRT_c13081	124	EYU45871.1	0.0	GO:0016740	70.00	45.44	40
IRC_IRT_c13088	277	XP_003610225.1	0.0	-	63.00	38.12	36
IRC_IRT_c13093	107	KEH15547.1	0.0	GO:0009451	82.00	54.30	35
IRC_IRT_c13104	179	XP_002509042.1	0.3	-	70.00	33.88	34
IRC_IRT_c13124	111	EPS72270.1	0.0	-	63.00	38.12	41
IRC_IRT_c13129	186	BAH80021.1	0.0	-	62.00	54.68	61
IRC_IRT_c13140	132	BAD68062.1	0.0	GO:0005739	100.00	66.24	34
IRC_IRT_c13166	154	XP_008236982.1	0.5	-	50.00	33.50	42
IRC_IRT_c13184	112	XP_010065639.1	0.3	-	67.00	33.50	34

IRC_IRT_c13193	114	ABR25802.1	0.0	GO:0006355	100.00	76.26	35
IRC_IRT_c13194	169	XP_002505798.1	0.0	GO:0004018	91.00	108.61	56
IRC_IRT_c13203	109	EEC80776.1	0.6	-	61.00	32.73	36
IRC_IRT_c13206	115	XP_002536521.1	0.0	-	83.00	59.31	37
IRC_IRT_c13208	122	AAM00989.1	0.0	GO:0003676	80.00	55.45	35
IRC_IRT_c13217	111	XP_006391732.1	0.0	GO:0003922	82.00	51.60	35
IRC_IRT_c13218	147	EAY90106.1	0.0	-	100.00	91.28	46
IRC_IRT_c13220	119	EMS68947.1	0.6	-	48.00	32.73	35
IRC_IRT_c13222	168	XP_010227842.1	0.0	-	54.00	37.35	57
IRC_IRT_c13244	127	AGT63299.1	0.1	-	64.00	33.88	34
IRC_IRT_c13265	120	EAY72911.1	0.0	GO:0006355	100.00	85.50	40
IRC_IRT_c13266	290	KEH17697.1	0.0	-	63.00	44.28	36
IRC_IRT_c13279	178	XP_002539889.1	0.0	GO:0003824	71.00	63.93	57
IRC_IRT_c13287	110	BAD08993.1	0.0	-	100.00	75.10	36
IRC_IRT_c13305	193	XP_002538477.1	0.0	GO:0008152	87.00	87.04	55
IRC_IRT_c13317	126	ABF94650.1	0.0	GO:0016020	64.00	45.44	34
IRC_IRT_c13334	101	XP_006655354.1	0.0	-	88.00	46.60	34
IRC_IRT_c13336	105	XP_006648457.1	0.4	-	71.00	33.50	35
IRC_IRT_c13347	115	AIU48791.1	0.0	-	66.00	40.05	39
IRC_IRT_c13361	157	CDP18827.1	0.0	GO:0003824	66.00	53.53	50
IRC_IRT_c13379	109	XP_005849660.1	0.0	GO:0000166	75.00	42.74	36
IRC_IRT_c13388	179	CAE02013.3	0.0	-	66.00	40.05	50
IRC_IRT_c13390	168	EMT06890.1	0.6	-	56.00	33.50	37
IRC_IRT_c13420	170	KDO62063.1	0.0	-	55.00	42.36	56
IRC_IRT_c13421	205	AAV24823.1	0.0	GO:0003964	98.00	124.02	60
IRC_IRT_c13435	134	ABG22552.1	0.0	GO:0004523	90.00	83.57	41
IRC_IRT_c13443	106	KFM22902.1	0.0	GO:0005829	65.00	48.14	41
IRC_IRT_c13444	136	XP_001781920.1	0.7	-	52.00	32.73	40
IRC_IRT_c13445	147	NP_001056328.1	0.0	GO:0016023	100.00	104.38	48
IRC_IRT_c13465	118	XP_006841540.1	0.0	-	64.00	38.89	37
IRC_IRT_c13466	186	EEC76877.1	0.0	GO:0044763	66.00	68.17	59
IRC_IRT_c13471	109	ABA99331.1	0.0	-	77.00	50.45	35
IRC_IRT_c13473	115	XP_006647879.1	0.0	GO:0005576	100.00	72.40	35

IRC_IRT_c13483	122	EEE52321.1	0.0	GO:0043565	92.00	73.56	40
IRC_IRT_c13489	110	EEE67873.1	0.0	-	70.00	36.19	34
IRC_IRT_c13505	128	BAJ90968.1	0.0	-	69.00	45.05	42
IRC_IRT_c13513	120	XP_002500761.1	0.0	GO:0019543	75.00	52.76	40
IRC_IRT_c13515	133	XP_001786560.1	0.0	GO:0044763	70.00	42.36	40
IRC_IRT_c13533	137	BAJ99956.1	0.7	-	54.00	32.73	37
IRC_IRT_c13545	119	XP_002535867.1	0.0	GO:0004731	91.00	63.54	35
IRC_IRT_c13548	126	XP_006372250.1	0.3	-	66.00	33.88	42
IRC_IRT_c13594	160	EEC76953.1	0.0	-	71.00	66.24	52
IRC_IRT_c13606	108	EEC69922.1	0.0	GO:0009536	82.00	57.77	35
IRC_IRT_c13649	127	NP_001058075.1	0.0	GO:0016757	100.00	95.13	42
IRC_IRT_c13650	138	XP_011082719.1	0.0	GO:0044763	71.00	49.29	42
IRC_IRT_c13652	180	XP_004969498.1	0.7	-	50.00	33.50	44
IRC_IRT_c13671	130	XP_002536504.1	0.0	-	69.00	52.76	43
IRC_IRT_c13698	173	YP_009045751.1	0.0	GO:0005739	92.00	93.20	51
IRC_IRT_c13709	130	XP_010915994.1	0.5	-	52.00	33.11	44
IRC_IRT_c13713	109	ABF98292.1	0.0	GO:0046961	100.00	70.86	35
IRC_IRT_c13721	198	XP_009595326.1	0.3	-	57.00	33.88	40
IRC_IRT_c13723	148	BAK00396.1	0.0	GO:0044710	71.00	56.23	49
IRC_IRT_c13743	130	XP_002540428.1	0.0	-	78.00	55.07	41
IRC_IRT_c13749	152	DAA02081.1	0.0	GO:0005739	100.00	78.18	50
IRC_IRT_c13762	130	CCO18457.1	0.3	-	51.00	33.88	35
IRC_IRT_c13763	112	XP_002538279.1	0.0	GO:0004177	97.00	74.71	37
IRC_IRT_c13768	139	XP_006492747.1	0.6	-	55.00	31.19	36
IRC_IRT_c13771	204	XP_010484229.1	1.0	-	40.00	33.11	66
IRC_IRT_c13792	239	XP_005652149.1	0.0	GO:0044699	81.00	66.24	44
IRC_IRT_c13801	145	CAD40089.2	0.0	GO:0003964	100.00	105.92	48
IRC_IRT_c13819	109	XP_002537322.1	0.0	GO:0006355	94.00	66.24	36
IRC_IRT_c13829	105	KGN59899.1	0.0	GO:0004553	79.00	56.23	34
IRC_IRT_c13880	274	KJB49896.1	0.0	-	53.00	44.67	58
IRC_IRT_c13908	200	XP_009805057.1	0.3	-	57.00	31.96	52
IRC_IRT_c13930	113	KIY99589.1	0.6	-	67.00	32.73	34
IRC_IRT_c13951	224	XP_009350076.1	0.0	GO:0071704	77.00	56.23	40

IRC_IRT_c13958	161	XP_007134642.1	0.6	-	61.00	33.50	36
IRC_IRT_c13965	268	BAJ11784.1	0.0	-	73.00	45.44	34
IRC_IRT_c14004	135	KGN54736.1	0.1	-	54.00	32.73	46
IRC_IRT_c14018	148	XP_004289853.1	0.0	GO:0046872	76.00	59.31	43
IRC_IRT_c14019	226	XP_003588337.1	0.0	-	52.00	37.74	50
IRC_IRT_c14051	131	AEZ52407.1	0.5	-	53.00	32.73	39
IRC_IRT_c14100	122	XP_002537898.1	0.0	GO:0008152	77.00	48.14	40
IRC_IRT_c14115	165	XP_001786557.1	0.0	GO:0055114	73.00	50.06	45
IRC_IRT_c14122	163	XP_002978237.1	0.0	GO:0044267	71.00	55.84	52
IRC_IRT_c14144	132	Q9G4F5.1	0.0	GO:0016020	80.00	65.08	41
IRC_IRT_c14151	105	XP_006476993.1	0.0	-	68.00	36.97	35
IRC_IRT_c14155	199	XP_008392011.1	0.0	GO:0006796	71.00	80.49	66
IRC_IRT_c14158	110	NP_001182847.1	0.0	GO:0000394	100.00	75.10	36
IRC_IRT_c14165	126	XP_001416565.1	0.0	GO:0016779	70.00	42.36	37
IRC_IRT_c14169	116	CAI44648.2	0.0	GO:0005739	91.00	70.48	36
IRC_IRT_c14185	111	XP_004963310.1	0.0	GO:0005829	85.00	60.85	35
IRC_IRT_c14196	104	BAD19293.1	0.0	-	100.00	67.78	34
IRC_IRT_c14207	121	CDP03071.1	0.0	GO:0032508	85.00	74.33	40
IRC_IRT_c14216	115	AEB38135.1	0.1	-	52.00	32.73	38
IRC_IRT_c14218	154	XP_008245771.1	0.0	-	63.00	35.42	47
IRC_IRT_c14241	142	EEE61323.1	0.0	-	97.00	73.94	35
IRC_IRT_c14257	164	XP_007161040.1	0.0	-	61.00	36.97	34
IRC_IRT_c14278	175	Q9G4F5.1	0.0	GO:0000166	72.00	48.52	40
IRC_IRT_c14303	179	XP_002949757.1	0.0	GO:0005507	77.00	87.04	59
IRC_IRT_c14305	135	XP_001758782.1	0.0	GO:0015851	87.00	64.70	39
IRC_IRT_c14307	107	KCW53221.1	0.5	-	60.00	33.11	38
IRC_IRT_c14314	135	XP_002537986.1	0.7	-	65.00	32.73	38
IRC_IRT_c14319	114	ABF97459.1	0.0	-	65.00	37.35	35
IRC_IRT_c14323	121	XP_006465076.1	0.6	-	69.00	32.73	36
IRC_IRT_c14331	173	EYU28980.1	0.4	-	58.00	34.27	36
IRC_IRT_c14335	111	CCO14840.1	0.0	-	66.00	40.43	36
IRC_IRT_c14356	123	XP_005849215.1	0.0	-	75.00	39.28	36
IRC_IRT_c14359	170	BAJ99466.1	0.0	GO:0044710	75.00	65.47	52

IRC_IRT_c14360	115	BAJ86187.1	0.0	GO:0000325	91.00	63.54	36
IRC_IRT_c14390	113	KDD75171.1	0.2	-	80.00	33.50	36
IRC_IRT_c14398	109	XP_011027157.1	0.0	GO:0043231	68.00	54.68	35
IRC_IRT_c14412	102	XP_002537836.1	0.0	-	67.00	38.89	34
IRC_IRT_c14413	127	XP_003064997.1	0.0	-	62.00	44.67	40
IRC_IRT_c14430	105	XP_002539706.1	0.0	GO:0015749	82.00	46.21	34
IRC_IRT_c14437	123	BAD81981.1	0.0	GO:0005739	100.00	83.57	41
IRC_IRT_c14438	124	YP_008080930.1	0.0	-	70.00	46.21	40
IRC_IRT_c14472	106	CAD40482.1	0.0	GO:0004523	94.00	70.09	3.50E+01
IRC_IRT_c14494	158	AAN05000.1	0.0	-	75.00	53.14	36
IRC_IRT_c14509	129	XP_011014252.1	0.0	GO:0044710	82.00	64.70	41
IRC_IRT_c14539	121	Q9G4F5.1	0.0	-	68.00	39.28	35
IRC_IRT_c14545	153	EAZ24371.1	0.0	GO:0030246	100.00	46.98	38
IRC_IRT_c14584	133	XP_009350062.1	0.0	-	79.00	53.53	43
IRC_IRT_c14594	148	XP_001771522.1	0.0	-	63.00	39.66	36
IRC_IRT_c14632	129	XP_002535903.1	0.0	-	85.00	63.54	42
IRC_IRT_c14657	105	EEC82596.1	0.0	GO:0016023	97.00	72.40	35
IRC_IRT_c14663	107	XP_010237153.1	0.0	GO:0046872	78.00	46.98	37
IRC_IRT_c14680	112	XP_002540125.1	0.0	GO:0009326	91.00	56.23	35
IRC_IRT_c14690	120	KCW82463.1	0.0	GO:0016491	71.00	44.67	39
IRC_IRT_c14704	138	XP_009350056.1	0.0	GO:0045910	85.00	59.69	34
IRC_IRT_c14711	119	EDQ48453.1	0.0	-	76.00	45.44	38
IRC_IRT_c14740	114	Q9FVG8.1	0.0	GO:0004478	85.00	49.29	34
IRC_IRT_c14746	122	XP_009367145.1	0.1	-	61.00	36.19	34
IRC_IRT_c14748	135	BAA75236.1	0.0	GO:0003676	95.00	93.59	44
IRC_IRT_c14755	130	KIZ06066.1	0.3	-	57.00	34.27	35
IRC_IRT_c14762	143	XP_005844162.1	0.0	GO:0044763	65.00	47.75	44
IRC_IRT_c14765	142	XP_011015250.1	0.7	-	53.00	32.73	45
IRC_IRT_c14787	115	KEH15262.1	0.0	GO:0006810	74.00	46.21	35
IRC_IRT_c14790	124	AFK45535.1	0.0	GO:0008236	77.00	45.05	36
IRC_IRT_c14806	139	AFK45219.1	0.0	GO:0005488	67.00	53.91	46
IRC_IRT_c14819	110	XP_006841540.1	0.0	-	66.00	38.12	36
IRC_IRT_c14821	125	XP_005651772.1	0.5	-	54.00	33.11	37

IRC_IRT_c14858	177	KEH29749.1	0.0	-	61.00	44.28	42
IRC_IRT_c14864	159	XP_002539004.1	0.5	-	51.00	32.34	35
IRC_IRT_c14865	236	KEH17697.1	0.0	-	59.00	36.19	37
IRC_IRT_c14873	149	EEC69895.1	0.0	GO:0030247	97.00	77.41	36
IRC_IRT_c14881	141	XP_003607358.1	0.0	-	53.00	37.35	47
IRC_IRT_c14897	119	EYU33320.1	0.6	-	51.00	32.34	39
IRC_IRT_c14911	174	AAO89143.1	0.0	GO:0004129	97.00	82.03	42
IRC_IRT_c14953	157	XP_002537480.1	0.0	-	66.00	38.12	42
IRC_IRT_c14958	127	XP_005644356.1	0.0	-	66.00	45.44	39
IRC_IRT_c14977	187	XP_005650473.1	0.0	GO:0003824	63.00	55.84	58
IRC_IRT_c14983	218	YP_001312258.1	0.6	-	50.00	31.57	42
IRC_IRT_c14988	174	KDP32562.1	0.0	-	65.00	36.58	38
IRC_IRT_c15001	171	XP_002954151.1	0.6	-	61.00	31.19	34
IRC_IRT_c15023	153	XP_003595724.1	0.0	-	70.00	45.82	37
IRC_IRT_c15046	198	XP_002537752.1	0.0	GO:0016491	71.00	57.38	46
IRC_IRT_c15056	122	EEC66780.1	0.0	GO:0017004	95.00	80.88	40
IRC_IRT_c15065	153	CCO14840.1	0.0	-	67.00	39.28	43
IRC_IRT_c15067	129	EDQ49166.1	0.0	-	77.00	46.21	35
IRC_IRT_c15096	171	BAD54552.1	0.0	-	89.00	64.31	37
IRC_IRT_c15116	149	AAY63546.1	0.0	GO:0005576	81.00	79.34	49
IRC_IRT_c15142	153	EPS70023.1	0.0	-	56.00	36.19	39
IRC_IRT_c15151	155	EXC01914.1	0.0	-	58.00	45.44	36
IRC_IRT_c15163	126	ERN16843.1	0.1	-	54.00	33.88	42
IRC_IRT_c15208	225	YP_588403.1	0.0	-	63.00	43.90	41
IRC_IRT_c15246	132	XP_001786506.1	0.0	GO:0016798	70.00	45.44	40
IRC_IRT_c15265	105	XP_010498497.1	0.0	-	65.00	43.51	40
IRC_IRT_c15271	102	XP_007137831.1	0.0	GO:0000166	73.00	45.05	34
IRC_IRT_c15289	147	XP_002540026.1	0.1	-	64.00	34.65	39
IRC_IRT_c15309	152	XP_001786726.1	0.0	-	70.00	49.29	44
IRC_IRT_c15339	435	ABR26094.1	0.0	-	65.00	44.28	49
IRC_IRT_c15351	111	KIY92373.1	0.0	-	82.00	40.05	34
IRC_IRT_c15353	115	XP_002537832.1	0.0	-	82.00	49.29	35
IRC_IRT_c15362	112	AFW76764.1	1.0	-	61.00	31.96	36

IRC_IRT_c15364	123	BAC83310.1	0.0	GO:0016021	100.00	80.49	38
IRC_IRT_c15370	158	CCO16731.1	0.0	-	72.00	54.68	44
IRC_IRT_c15377	130	XP_010314965.1	0.0	-	68.00	45.82	38
IRC_IRT_c15388	183	XP_003608191.1	0.0	-	60.00	37.35	40
IRC_IRT_c15409	106	XP_002538370.1	0.0	-	71.00	35.81	35
IRC_IRT_c15419	156	XP_003520624.1	0.3	-	60.00	32.73	41
IRC_IRT_c15420	165	XP_007024704.1	0.0	-	62.00	43.13	50
IRC_IRT_c15429	127	AAM93715.1	0.0	-	59.00	49.29	47
IRC_IRT_c15487	114	XP_004969834.1	1.0	-	55.00	32.34	34
IRC_IRT_c15492	144	XP_002538040.1	0.0	-	64.00	41.97	34
IRC_IRT_c15500	114	XP_002537026.1	0.0	GO:0004871	76.00	50.83	38
IRC_IRT_c15510	154	XP_006406198.1	0.1	-	51.00	35.81	37
IRC_IRT_c15522	113	XP_008778132.1	0.8	-	50.00	31.96	34
IRC_IRT_c15526	105	AAO19372.1	0.0	GO:0004523	100.00	72.79	34
IRC_IRT_c15532	150	XP_002538605.1	0.0	-	64.00	48.14	50
IRC_IRT_c15541	123	XP_002538147.1	0.0	-	68.00	45.44	41
IRC_IRT_c15547	126	XP_002538422.1	0.0	-	71.00	36.19	38
IRC_IRT_c15551	167	EEE69402.1	0.0	GO:0048519	100.00	119.40	55
IRC_IRT_c15562	115	XP_002540112.1	0.0	-	77.00	48.52	36
IRC_IRT_c15575	108	AJO67231.1	0.0	-	62.00	36.97	35
IRC_IRT_c15581	114	XP_001417789.1	0.0	-	71.00	43.13	38
IRC_IRT_c15598	108	EDQ48091.1	0.0	-	58.00	36.58	34
IRC_IRT_c15605	136	XP_002955962.1	0.0	-	65.00	45.82	44
IRC_IRT_c15622	113	XP_001786560.1	0.0	GO:0006289	97.00	62.77	35
IRC_IRT_c15626	154	EDQ49172.1	0.0	-	76.00	63.16	50
IRC_IRT_c15627	143	XP_002960029.1	0.0	GO:0046912	92.00	63.93	40
IRC_IRT_c15629	156	CAH67737.1	0.0	-	61.00	46.21	52
IRC_IRT_c15636	119	XP_001775197.1	0.5	-	56.00	32.73	39
IRC_IRT_c15653	137	EEC69964.1	0.7	-	56.00	32.73	39
IRC_IRT_c15666	106	EAZ21769.1	0.0	GO:0006950	100.00	76.26	35
IRC_IRT_c15673	156	CAE76059.1	0.0	GO:0005739	98.00	102.45	51
IRC_IRT_c15677	121	XP_001761040.1	1.0	-	41.00	31.19	36
IRC_IRT_c15681	133	KJB27263.1	0.0	GO:0009611	83.00	61.23	42

IRC_IRT_c15682	170	XP_001691702.1	0.0	-	71.00	43.90	5.20E+01
IRC_IRT_c15683	119	XP_002536199.1	0.0	-	97.00	73.17	39
IRC_IRT_c15709	113	XP_006342925.1	0.0	GO:0071704	75.00	44.67	36
IRC_IRT_c15730	108	XP_002535590.1	0.0	GO:0016021	97.00	69.32	36
IRC_IRT_c15745	141	XP_006843179.1	0.3	-	66.00	33.88	36
IRC_IRT_c15770	114	DAA36215.1	0.1	-	55.00	34.27	34
IRC_IRT_c15774	139	EDQ48498.1	0.0	-	77.00	60.08	40
IRC_IRT_c15811	113	ABA98774.1	0.0	-	100.00	79.34	37
IRC_IRT_c15820	107	NP_001062757.1	0.0	GO:0016023	100.00	75.49	35
IRC_IRT_c15847	125	XP_006858513.1	0.0	-	66.00	43.13	36
IRC_IRT_c15848	130	XP_002534960.1	0.0	GO:0019538	76.00	50.45	43
IRC_IRT_c15853	134	ABA99876.2	0.0	GO:0009536	100.00	97.83	44
IRC_IRT_c15869	159	XP_002537860.1	0.0	GO:0050794	77.00	68.17	49
IRC_IRT_c15870	123	XP_006390214.1	0.0	-	64.00	40.05	37
IRC_IRT_c15887	181	XP_007224160.1	0.2	-	56.00	34.27	46
IRC_IRT_c15901	127	XP_010911627.1	0.1	-	61.00	35.42	34
IRC_IRT_c15906	120	XP_011016603.1	0.0	GO:0043169	87.00	67.78	39
IRC_IRT_c15908	159	XP_002538398.1	0.0	-	59.00	38.12	52
IRC_IRT_c15925	228	XP_002488950.1	0.0	-	97.00	69.71	34
IRC_IRT_c15964	153	XP_004965240.1	0.0	GO:0044763	71.00	57.38	45
IRC_IRT_c15995	154	BAA06827.1	0.0	GO:0016020	100.00	102.83	48
IRC_IRT_c16009	126	CCO15570.1	0.0	GO:0003723	78.00	53.91	41
IRC_IRT_c16015	140	EEC76122.1	0.0	GO:0050660	81.00	53.91	37
IRC_IRT_c16146	106	EEE65426.1	0.0	GO:0032259	100.00	73.94	34
IRC_IRT_c16225	126	XP_010453582.1	0.0	GO:0090305	78.00	51.60	42
IRC_IRT_c16228	157	EEE62790.1	0.0	GO:0016021	100.00	110.15	52
IRC_IRT_c16230	208	EAZ13518.1	0.0	GO:1901601	91.00	84.73	46
IRC_IRT_c16261	152	EEC85062.1	0.0	GO:0005739	100.00	85.89	38
IRC_IRT_c16267	119	XP_002536752.1	0.0	-	68.00	38.51	38
IRC_IRT_c16268	144	XP_007222919.1	0.0	GO:0071704	68.00	50.83	45
IRC_IRT_c16279	103	AAP55139.2	0.0	GO:0090502	97.00	68.94	34
IRC_IRT_c16285	129	KJB79865.1	0.0	GO:0008270	88.00	76.64	4.20E+01
IRC_IRT_c16290	120	KDD72801.1	0.0	-	67.00	45.82	40

IRC_IRT_c16291	113	EMS47936.1	0.0	-	75.00	37.35	36
IRC_IRT_c16294	126	KFM27378.1	0.0	-	76.00	51.60	42
IRC_IRT_c16321	119	XP_006842716.1	0.0	GO:0046523	71.00	54.30	39
IRC_IRT_c16331	130	ABA99728.1	0.0	-	83.00	63.16	36
IRC_IRT_c16346	115	NP_001066607.1	0.0	GO:0019253	100.00	73.94	38
IRC_IRT_c16347	159	EEC76877.1	0.0	GO:0046961	92.00	95.52	53
IRC_IRT_c16363	183	XP_002537904.1	0.0	-	65.00	48.14	41
IRC_IRT_c16364	118	XP_002540423.1	0.8	-	54.00	30.42	35
IRC_IRT_c16367	245	BAD61609.1	0.0	GO:0005739	80.00	100.14	70
IRC_IRT_c16374	102	XP_008657455.1	0.0	GO:0046872	70.00	50.06	34
IRC_IRT_c16380	104	AFI48017.1	0.4	-	61.00	31.57	34
IRC_IRT_c16396	102	XP_011071514.1	0.0	GO:0080041	100.00	75.87	34
IRC_IRT_c16402	176	CAH66235.1	0.0	GO:0004523	87.00	95.52	58
IRC_IRT_c16429	133	XP_009350805.1	0.0	-	73.00	45.05	38
IRC_IRT_c16434	193	XP_002538398.1	0.8	-	62.00	33.11	35
IRC_IRT_c16463	148	XP_004503218.1	0.6	-	56.00	33.11	37
IRC_IRT_c16468	105	XP_005651602.1	0.0	-	64.00	36.58	34
IRC_IRT_c16487	171	XP_002488914.1	0.0	-	71.00	39.28	38
IRC_IRT_c16528	127	XP_002535893.1	0.0	GO:0006810	89.00	52.76	37
IRC_IRT_c16572	132	XP_005850067.1	0.0	-	67.00	36.97	34
IRC_IRT_c16581	146	AAV59387.1	0.0	-	79.00	62.39	44
IRC_IRT_c16605	202	CBI30617.3	0.7	-	48.00	33.11	64
IRC_IRT_c16608	149	AAP53739.1	0.0	GO:0003676	97.00	103.61	45
IRC_IRT_c16610	107	KIY92249.1	0.0	GO:0051287	88.00	57.38	34
IRC_IRT_c16612	108	ABR17747.1	0.0	-	69.00	38.51	36
IRC_IRT_c16621	109	XP_006651768.1	0.0	GO:0006355	100.00	86.66	36
IRC_IRT_c16622	111	XP_002488904.1	0.0	GO:0046872	79.00	46.98	34
IRC_IRT_c16650	135	KFM29035.1	0.0	GO:0010368	81.00	67.40	43
IRC_IRT_c16652	102	XP_002456960.1	0.0	-	76.00	44.28	34
IRC_IRT_c16668	113	KJB62847.1	0.0	GO:0007010	94.00	66.24	35
IRC_IRT_c16670	138	XP_002536193.1	0.0	GO:0071702	73.00	47.75	45
IRC_IRT_c16674	238	XP_002536340.1	0.0	-	93.00	82.42	48
IRC_IRT_c16688	158	XP_011014867.1	0.0	-	51.00	36.97	70

IRC_IRT_c16699	130	NP_001176697.1	0.0	GO:0017148	94.00	79.72	38
IRC_IRT_c16702	131	BAD87795.1	0.0	-	86.00	73.17	43
IRC_IRT_c16729	110	BAD34350.1	0.0	GO:0009536	100.00	43.13	36
IRC_IRT_c16731	148	XP_006405913.1	0.0	-	74.00	58.15	39
IRC_IRT_c16732	131	XP_003063724.1	0.2	-	62.00	34.27	51
IRC_IRT_c16754	165	AFU07644.1	0.7	-	60.00	32.34	35
IRC_IRT_c16779	203	EEC76953.1	0.0	GO:0003849	86.00	96.67	67
IRC_IRT_c16835	139	KDP44567.1	0.7	-	59.00	30.80	37
IRC_IRT_c16850	124	AGJ75609.1	0.0	GO:0019253	94.00	73.56	37
IRC_IRT_c16854	115	CEG01650.1	0.5	-	61.00	31.19	34
IRC_IRT_c16867	111	EAZ24144.1	0.0	GO:0005739	100.00	80.49	37
IRC_IRT_c16874	127	NP_001174925.1	0.0	-	63.00	41.97	38
IRC_IRT_c16933	128	EEE51325.1	0.0	GO:0005739	85.00	73.94	42
IRC_IRT_c16947	140	AAV97960.1	0.0	-	59.00	39.66	37
IRC_IRT_c16972	105	CAD40418.3	0.0	-	82.00	54.68	34
IRC_IRT_c17029	105	CCO15760.1	0.2	-	65.00	33.88	35
IRC_IRT_c17061	111	XP_002960009.1	0.0	GO:0005737	86.00	59.69	37
IRC_IRT_c17066	106	EAY75853.1	0.0	GO:0016023	100.00	72.40	35
IRC_IRT_c17070	203	CAE02184.2	0.0	GO:0003676	98.00	135.96	66
IRC_IRT_c17080	141	KDD75269.1	0.0	GO:0055114	84.00	62.39	46
IRC_IRT_c17083	105	XP_001701551.1	0.0	GO:0050660	97.00	60.85	34
IRC_IRT_c17099	109	CAD39388.2	0.0	GO:0003964	100.00	79.34	34
IRC_IRT_c17101	107	ABB46561.1	0.0	GO:0004252	88.00	59.31	34
IRC_IRT_c17104	102	CCO14045.1	0.0	GO:0003735	82.00	53.14	34
IRC_IRT_c17116	109	XP_001416370.1	0.4	-	58.00	32.73	36
IRC_IRT_c17133	157	XP_002500977.1	0.0	GO:0016755	68.00	55.84	50
IRC_IRT_c17149	104	EAY86034.1	0.0	GO:0003899	100.00	73.94	34
IRC_IRT_c17152	162	XP_001755009.1	0.0	GO:0071704	75.00	68.94	54
IRC_IRT_c17159	183	DAA59171.1	0.1	-	50.00	35.81	62
IRC_IRT_c17171	107	AAV59370.1	0.0	GO:0055114	100.00	83.19	35
IRC_IRT_c17176	107	NP_001060443.1	0.0	GO:0008152	100.00	73.56	35
IRC_IRT_c17184	157	XP_006826737.1	0.0	-	62.00	44.28	51
IRC_IRT_c17193	166	XP_002534720.1	0.0	-	95.00	82.03	41

IRC_IRT_c17195	103	DAA57520.1	0.0	-	64.00	40.82	34
IRC_IRT_c17197	162	XP_005848152.1	0.0	GO:0098655	86.00	77.41	50
IRC_IRT_c17211	240	EMS46732.1	0.0	GO:0080041	82.00	132.88	80
IRC_IRT_c17221	164	ACU18628.1	0.0	-	68.00	48.52	47
IRC_IRT_c17224	133	XP_002501910.1	0.0	-	59.00	36.97	44
IRC_IRT_c17233	123	KJB75052.1	0.5	-	53.00	33.11	39
IRC_IRT_c17280	117	XP_005647191.1	0.0	GO:0046872	68.00	48.14	38
IRC_IRT_c17290	106	XP_002539706.1	0.0	-	77.00	39.66	35
IRC_IRT_c17328	116	NP_001063865.1	0.0	GO:0048544	100.00	83.96	38
IRC_IRT_c17333	113	NP_001056747.1	0.0	GO:0016311	100.00	81.65	37
IRC_IRT_c17335	158	EAY84569.1	0.0	GO:0050660	80.00	61.23	40
IRC_IRT_c17338	126	CAA77738.1	0.0	GO:0016192	100.00	82.42	41
IRC_IRT_c17350	307	AAP04393.1	0.0	GO:0003746	100.00	199.52	97
IRC_IRT_c17357	151	XP_010055179.1	0.0	-	76.00	58.15	39
IRC_IRT_c17400	129	XP_003063226.1	0.0	GO:0016616	72.00	48.91	43
IRC_IRT_c17404	119	XP_008806711.1	0.1	-	58.00	34.65	36
IRC_IRT_c17407	183	XP_008223330.1	0.2	-	48.00	34.65	60
IRC_IRT_c17412	102	KJB41959.1	0.0	-	71.00	43.51	35
IRC_IRT_c17436	116	BAD23081.1	0.0	-	53.00	38.51	47
IRC_IRT_c17438	135	EEE62867.1	0.8	-	55.00	32.34	36
IRC_IRT_c17442	126	AAF16526.1	0.0	GO:0044267	82.00	52.37	35
IRC_IRT_c17455	118	XP_005650458.1	0.0	GO:0009295	71.00	44.67	39
IRC_IRT_c17461	141	XP_008645888.1	0.0	GO:0044238	63.00	53.14	47
IRC_IRT_c17465	147	BAC15464.1	0.0	-	79.00	56.61	34
IRC_IRT_c17469	107	ABA97326.2	0.0	GO:0004523	91.00	66.63	35
IRC_IRT_c17480	115	EAY73221.1	0.0	-	76.00	42.36	34
IRC_IRT_c17481	159	XP_007023138.1	0.7	-	58.00	33.11	43
IRC_IRT_c17485	143	EPS64277.1	0.0	GO:0043167	73.00	41.97	34
IRC_IRT_c17489	202	AFK35083.1	0.0	-	72.00	45.05	43
IRC_IRT_c17534	114	XP_010931814.1	0.7	-	58.00	32.73	41
IRC_IRT_c17536	150	XP_002537677.1	0.0	GO:0050794	78.00	46.60	37
IRC_IRT_c17547	105	YP_008802544.1	0.0	GO:0005739	80.00	38.89	35
IRC_IRT_c17551	106	EEC73753.1	0.0	GO:0016023	70.00	53.14	40

IRC_IRT_c17563	130	KIY91917.1	0.0	GO:0006098	95.00	49.29	43
IRC_IRT_c17594	113	XP_002537768.1	0.0	-	70.00	37.35	37
IRC_IRT_c17608	152	XP_002466635.1	0.7	-	52.00	32.73	40
IRC_IRT_c17609	118	KEH15180.1	0.0	-	76.00	41.20	34
IRC_IRT_c17612	102	XP_002535367.1	0.0	-	73.00	38.89	34
IRC_IRT_c17620	108	BAD14922.1	0.0	GO:0006694	100.00	73.56	34
IRC_IRT_c17651	113	EMS56529.1	0.0	GO:0016491	83.00	46.60	36
IRC_IRT_c17659	120	XP_002535815.1	0.0	GO:0003824	67.00	41.59	34
IRC_IRT_c17664	137	XP_006648551.1	0.0	-	94.00	73.94	38
IRC_IRT_c17689	160	XP_009401580.1	0.0	GO:0016772	80.00	73.17	52
IRC_IRT_c17702	106	CCO66287.1	0.0	GO:0042558	82.00	53.53	35
IRC_IRT_c17706	106	XP_002536335.1	0.0	-	67.00	42.36	34
IRC_IRT_c17711	114	ABA95896.1	0.0	GO:0005643	100.00	82.03	37
IRC_IRT_c17715	121	XP_002537905.1	0.0	GO:0008239	95.00	79.34	40
IRC_IRT_c17729	145	KEH15587.1	0.0	GO:0005975	87.00	85.89	47
IRC_IRT_c17756	106	EAY92713.1	0.0	GO:0005739	76.00	51.22	34
IRC_IRT_c17761	142	KIZ00365.1	0.0	-	72.00	44.67	36
IRC_IRT_c17764	111	AJT43268.1	0.0	GO:0003824	72.00	51.99	36
IRC_IRT_c17775	184	ABO20848.1	0.0	-	79.00	53.14	34
IRC_IRT_c17778	107	XP_002504456.1	0.8	-	50.00	31.96	38
IRC_IRT_c17793	109	ADO16010.1	0.0	GO:0050660	88.00	65.86	35
IRC_IRT_c17816	106	XP_002535370.1	0.0	GO:0000155	79.00	42.74	34
IRC_IRT_c17827	159	YP_001019104.1	0.0	GO:0016779	77.00	45.44	35
IRC_IRT_c17850	110	XP_004253340.1	0.0	-	80.00	50.45	36
IRC_IRT_c17854	116	NP_001063038.1	0.0	GO:0010413	97.00	78.95	3.80E+01
IRC_IRT_c17860	103	BAC79770.1	0.1	-	58.00	35.81	34
IRC_IRT_c17879	178	EDQ48417.1	0.0	GO:0044710	58.00	46.60	58
IRC_IRT_c17882	132	XP_008455171.1	0.4	-	60.00	33.88	41
IRC_IRT_c17890	104	KFK25722.1	0.0	GO:0006813	67.00	43.51	34
IRC_IRT_c17911	111	XP_004294417.2	0.9	-	50.00	32.34	36
IRC_IRT_c17916	114	YP_588355.1	0.0	GO:0005739	97.00	76.26	37
IRC_IRT_c17918	120	EEC66865.1	0.0	-	68.00	39.28	38
IRC_IRT_c17940	136	XP_008391164.1	0.6	-	56.00	32.73	37

IRC_IRT_c17960	165	XP_002536712.1	0.0	-	59.00	43.13	54
IRC_IRT_c17979	170	XP_004959959.1	0.5	-	61.00	33.50	36
IRC_IRT_c17983	117	NP_001064795.1	0.0	GO:0008270	100.00	78.95	3.90E+01
IRC_IRT_c17996	118	XP_008365678.1	0.0	-	59.00	37.35	37
IRC_IRT_c18007	147	XP_002488956.1	0.0	-	82.00	51.99	35
IRC_IRT_c18016	114	ABC24948.1	0.0	GO:0005737	80.00	50.45	36
IRC_IRT_c18038	132	EAZ10087.1	0.0	GO:0030247	90.00	67.40	40
IRC_IRT_c18051	113	EEC76877.1	0.0	GO:0046961	97.00	77.03	37
IRC_IRT_c18067	135	XP_001699630.1	0.3	-	58.00	32.34	41
IRC_IRT_c18102	257	AGT17357.1	0.0	-	69.00	51.60	43
IRC_IRT_c18119	147	XP_002539889.1	0.0	-	64.00	53.53	53
IRC_IRT_c18121	158	EAZ19084.1	0.0	-	64.00	41.97	54
IRC_IRT_c18151	102	NP_001147054.1	0.1	-	64.00	35.42	34
IRC_IRT_c18168	109	XP_002538383.1	0.0	-	61.00	36.58	34
IRC_IRT_c18169	156	BAD33863.1	0.0	-	100.00	98.21	51
IRC_IRT_c18178	142	XP_010059233.1	0.2	-	57.00	35.04	35
IRC_IRT_c18179	151	XP_010066735.1	0.4	-	57.00	33.50	42
IRC_IRT_c18189	170	Q8LKI3.1	0.0	GO:0044763	64.00	60.85	50
IRC_IRT_c18194	120	XP_002535704.1	0.0	GO:0008152	75.00	51.22	40
IRC_IRT_c18208	136	XP_009774911.1	0.0	GO:0006261	91.00	64.31	34
IRC_IRT_c18265	106	BAD23368.1	0.0	GO:0005739	82.00	52.37	34
IRC_IRT_c18307	125	CDP10922.1	0.0	-	62.00	37.74	37
IRC_IRT_c18321	137	CAE05779.2	0.0	GO:0004523	93.00	87.04	44
IRC_IRT_c18327	157	XP_011466312.1	0.4	-	52.00	33.88	48
IRC_IRT_c18339	106	EDQ48389.1	0.8	-	61.00	32.34	34
IRC_IRT_c18355	120	EEC68643.1	0.0	GO:0016023	100.00	81.65	39
IRC_IRT_c18357	185	XP_002536256.1	0.0	-	66.00	39.66	36
IRC_IRT_c18375	109	EMS62751.1	0.4	-	54.00	33.11	35
IRC_IRT_c18386	116	EPS72369.1	0.3	-	67.00	33.11	37
IRC_IRT_c18393	303	XP_009350076.1	0.0	GO:0008270	82.00	73.56	45
IRC_IRT_c18413	161	CDY30864.1	0.0	-	60.00	40.43	38
IRC_IRT_c18425	112	CDX87200.1	0.0	-	70.00	56.23	37
IRC_IRT_c18428	107	NP_001063599.1	0.0	GO:0036459	100.00	73.17	35

IRC_IRT_c18429	119	XP_005645734.1	0.5	-	62.00	33.50	37
IRC_IRT_c18431	152	XP_005643067.1	0.0	GO:0005840	82.00	69.32	50
IRC_IRT_c18436	130	XP_002506565.1	0.0	GO:0005737	75.00	50.06	37
IRC_IRT_c18439	102	ABF99560.1	0.0	GO:0016023	73.00	51.60	34
IRC_IRT_c18453	258	XP_003588355.1	0.0	-	70.00	46.98	41
IRC_IRT_c18463	127	XP_011459064.1	0.0	GO:0016301	64.00	42.36	37
IRC_IRT_c18465	109	XP_009362256.1	0.3	-	52.00	33.50	34
IRC_IRT_c18471	141	XP_011627754.1	0.0	GO:0003824	65.00	44.28	38
IRC_IRT_c18485	123	XP_002536731.1	0.0	-	71.00	41.59	38
IRC_IRT_c18524	125	KDO39227.1	0.0	-	56.00	39.28	41
IRC_IRT_c18533	126	XP_010024621.1	0.6	-	58.00	33.11	39
IRC_IRT_c18544	138	NP_038383.1	0.0	GO:0003899	91.00	67.78	37
IRC_IRT_c18551	132	XP_003627937.1	0.7	-	61.00	32.34	34
IRC_IRT_c18552	159	XP_001770563.1	0.0	-	62.00	40.43	54
IRC_IRT_c18575	148	BAD62069.1	0.0	-	63.00	34.27	38
IRC_IRT_c18576	162	XP_003555493.1	0.0	GO:1903046	67.00	47.37	53
IRC_IRT_c18582	140	XP_002540411.1	0.0	GO:0003824	66.00	41.97	42
IRC_IRT_c18583	152	XP_002536314.1	0.0	-	60.00	45.05	41
IRC_IRT_c18585	206	CCO18476.1	0.8	-	55.00	33.50	40
IRC_IRT_c18590	127	CAN80172.1	0.1	-	65.00	34.65	35
IRC_IRT_c18613	127	KFK37993.1	0.0	GO:0008236	72.00	48.52	40
IRC_IRT_c18642	130	KEH15984.1	0.0	-	57.00	41.59	42
IRC_IRT_c18674	248	XP_002539536.1	0.0	-	55.00	46.98	72
IRC_IRT_c18675	108	EAZ03662.1	0.0	GO:0005739	100.00	78.18	36
IRC_IRT_c18679	148	ABB47116.1	0.0	GO:0004523	88.00	77.80	50
IRC_IRT_c18698	171	XP_009350076.1	0.0	GO:0005525	100.00	93.20	44
IRC_IRT_c18715	148	KIY99115.1	0.0	GO:0044249	75.00	66.24	49
IRC_IRT_c18730	200	XP_002537860.1	0.0	GO:0050794	72.00	73.94	59
IRC_IRT_c18733	207	KGN53990.1	0.0	-	72.00	45.05	36
IRC_IRT_c18737	182	KDO48636.1	0.0	-	66.00	37.35	39
IRC_IRT_c18753	121	CDY47875.1	0.0	-	71.00	39.28	38
IRC_IRT_c18758	110	XP_001786858.1	0.6	-	65.00	32.34	35
IRC_IRT_c18764	122	ABR18118.1	0.0	GO:0016311	85.00	68.94	40

IRC_IRT_c18765	118	EEC68416.1	0.0	-	70.00	38.51	34
IRC_IRT_c18780	118	XP_009132208.1	0.0	GO:0005739	86.00	60.46	38
IRC_IRT_c18784	149	EAY77723.1	0.0	GO:0030247	93.00	95.52	49
IRC_IRT_c18812	115	EEC69467.1	0.0	GO:0006952	100.00	82.03	38
IRC_IRT_c18821	220	XP_002537236.1	0.0	-	75.00	65.47	49
IRC_IRT_c18848	126	KJB83525.1	0.1	-	65.00	35.42	40
IRC_IRT_c18849	143	XP_002536388.1	0.0	-	74.00	43.51	35
IRC_IRT_c18880	291	KDD76009.1	0.0	GO:0044763	68.00	58.15	54
IRC_IRT_c18904	121	XP_006493894.1	0.0	-	66.00	35.81	39
IRC_IRT_c18915	175	BAD52968.1	0.0	-	82.00	56.61	34
IRC_IRT_c18921	119	XP_002536300.1	0.0	-	58.00	38.51	34
IRC_IRT_c18923	109	CAN76048.1	0.0	GO:0006952	77.00	56.61	36
IRC_IRT_c18939	103	XP_002537550.1	0.1	-	55.00	33.88	34
IRC_IRT_c18954	212	ACB13094.1	0.0	GO:0046933	91.00	122.87	70
IRC_IRT_c18957	159	CBI31107.3	0.0	-	68.00	47.75	47
IRC_IRT_c18964	149	XP_009116589.1	0.0	-	63.00	41.20	36
IRC_IRT_c18992	122	XP_002535676.1	0.0	-	69.00	44.67	39
IRC_IRT_c18993	132	KIY99879.1	0.0	GO:0016020	89.00	60.85	37
IRC_IRT_c18999	133	EMT26138.1	0.0	-	57.00	38.51	42
IRC_IRT_c19003	110	XP_006417198.1	0.0	GO:0003824	70.00	40.43	34
IRC_IRT_c19006	124	EAZ05375.1	0.0	GO:0004747	97.00	86.27	41
IRC_IRT_c19008	251	XP_002538849.1	0.0	-	68.00	45.82	44
IRC_IRT_c19019	162	XP_002538330.1	0.0	-	61.00	38.89	39
IRC_IRT_c19040	155	XP_002538274.1	0.0	-	65.00	51.60	43
IRC_IRT_c19043	120	ACG26499.1	0.0	GO:0030170	95.00	68.55	40
IRC_IRT_c19051	109	XP_005849184.1	0.6	-	52.00	32.73	36
IRC_IRT_c19052	215	ABA97095.1	0.0	GO:0003676	100.00	138.27	66
IRC_IRT_c19062	182	XP_002534888.1	0.4	-	64.00	31.96	34
IRC_IRT_c19077	211	EEE51198.1	0.0	GO:0009536	80.00	70.86	45
IRC_IRT_c19090	155	XP_003595724.1	0.0	-	63.00	41.97	38
IRC_IRT_c19104	145	XP_004135951.1	0.7	-	62.00	32.73	45
IRC_IRT_c19110	123	XP_002954054.1	0.0	GO:0044237	70.00	50.45	40
IRC_IRT_c19115	152	AFW86832.1	0.0	-	81.00	67.01	38

IRC_IRT_c19141	354	XP_003638717.1	0.3	-	61.00	35.81	34
IRC_IRT_c19155	255	XP_002959031.1	0.0	GO:0000166	71.00	81.26	70
IRC_IRT_c19156	154	KFK34017.1	0.8	-	48.00	33.11	43
IRC_IRT_c19162	256	BAJ11779.1	0.0	-	76.00	45.82	34
IRC_IRT_c19168	159	XP_007161040.1	0.3	-	65.00	31.57	38
IRC_IRT_c19171	308	AGC78945.1	0.0	-	39.00	41.20	105
IRC_IRT_c19177	145	XP_011016758.1	0.0	-	82.00	66.24	41
IRC_IRT_c19179	288	XP_007154367.1	0.0	-	70.00	45.05	37
IRC_IRT_c19192	154	EYU29847.1	0.0	GO:0005739	68.00	45.82	51
IRC_IRT_c19206	103	XP_002535829.1	0.1	-	85.00	35.04	34
IRC_IRT_c19214	113	EEE67873.1	0.0	-	65.00	40.43	35
IRC_IRT_c19226	209	CDX71648.1	0.0	-	62.00	40.43	45
IRC_IRT_c19232	161	ERN15094.1	0.8	-	47.00	31.19	55
IRC_IRT_dn_c19259	210	XP_001698578.1	0.5	-	57.00	33.88	40
IRC_IRT_c19262	128	KJB38963.1	0.0	-	58.00	33.88	41
IRC_IRT_c19263	157	AHG05844.1	0.0	GO:0003676	77.00	51.22	35
IRC_IRT_c19271	119	AAT73674.2	0.0	-	92.00	78.18	39
IRC_IRT_c19272	133	XP_004135671.1	0.3	-	62.00	33.50	35
IRC_IRT_c19311	137	XP_004509500.1	0.1	-	69.00	35.04	36
IRC_IRT_c19324	118	XP_005845301.1	0.0	GO:0005829	79.00	65.08	39
IRC_IRT_c19328	128	XP_008809221.1	0.9	-	57.00	31.96	38
IRC_IRT_c19338	118	EAZ00448.1	0.0	GO:0055114	72.00	46.98	37
IRC_IRT_c19342	104	XP_006859022.1	0.0	GO:0005739	88.00	62.39	34
IRC_IRT_c19347	106	EAZ04676.1	0.0	GO:0055114	100.00	72.02	34
IRC_IRT_c19357	142	XP_002535722.1	0.0	-	62.00	42.74	37
IRC_IRT_c19365	116	EEE52318.1	0.0	GO:0022891	89.00	58.54	38
IRC_IRT_c19374	106	XP_007213378.1	0.0	GO:0003677	97.00	77.80	35
IRC_IRT_c19407	107	YP_009057477.1	0.0	GO:0003735	91.00	63.93	35
IRC_IRT_c19410	143	XP_005844401.1	0.0	GO:0043170	76.00	54.68	46
IRC_IRT_c19412	127	XP_002505901.1	0.0	GO:0009536	74.00	54.30	43
IRC_IRT_c19417	194	CEF99955.1	0.0	-	61.00	43.13	49
IRC_IRT_c19442	260	XP_003588337.1	0.0	-	66.00	41.20	36
IRC_IRT_c19453	309	CDX71648.1	0.0	-	80.00	54.30	36

IRC_IRT_c19463	153	AGV54820.1	0.6	-	47.00	33.11	38
IRC_IRT_c19467	130	XP_006395639.1	0.7	-	52.00	32.73	50
IRC_IRT_c19471	223	ABH09321.1	0.2	-	65.00	35.04	40
IRC_IRT_c19526	106	EEE67621.1	0.0	GO:0006511	88.00	63.54	35
IRC_IRT_c19561	112	XP_001786560.1	0.0	GO:0006289	85.00	54.30	35
IRC_IRT_c19567	191	H9BFQ0.1	0.0	GO:0008152	68.00	48.14	44
IRC_IRT_c19572	241	AGV54820.1	0.0	-	65.00	45.82	40
IRC_IRT_c19583	147	XP_002540121.1	0.0	-	59.00	51.99	44
IRC_IRT_c19585	238	NP_001189792.1	0.5	-	60.00	32.34	38
IRC_IRT_c19596	204	AGZ19352.1	0.0	GO:0009507	80.00	52.76	35
IRC_IRT_c19597	154	KFK37145.1	0.0	GO:0044763	64.00	45.82	50
IRC_IRT_c19613	110	XP_004963609.1	0.0	GO:0019676	88.00	61.23	35
IRC_IRT_c19616	127	EAZ13230.1	0.0	GO:0044249	87.00	64.70	39
IRC_IRT_c19626	126	XP_002538106.1	0.0	GO:0016787	69.00	44.67	39
IRC_IRT_c19650	212	XP_005848152.1	0.0	GO:0005488	67.00	47.75	43
IRC_IRT_c19667	138	XP_002535709.1	0.0	-	82.00	53.91	34
IRC_IRT_c19675	154	ABF94999.1	0.0	GO:0004523	100.00	78.57	36
IRC_IRT_c19677	145	XP_002535647.1	0.0	-	67.00	42.74	43
IRC_IRT_c19680	110	XP_010932244.1	0.0	GO:0010039	88.00	52.37	36
IRC_IRT_c19681	196	XP_009350073.1	0.0	-	74.00	59.69	47
IRC_IRT_c19699	130	XP_010928879.1	0.0	GO:0005515	70.00	55.07	41
IRC_IRT_c19702	116	EEC76122.1	0.0	GO:1901363	71.00	43.13	35
IRC_IRT_c19717	143	XP_007156921.1	0.1	-	43.00	33.11	64
IRC_IRT_c19741	107	XP_003063400.1	0.1	-	62.00	35.04	35
IRC_IRT_c19751	122	NP_001042509.2	0.0	GO:0044763	75.00	49.29	37
IRC_IRT_c19778	160	BAD10558.1	0.0	-	88.00	65.47	36
IRC_IRT_c19780	133	XP_002536193.1	0.0	GO:0000166	75.00	48.91	44
IRC_IRT_c19796	373	BAJ11784.1	0.0	-	58.00	44.28	50
IRC_IRT_c19817	106	XP_003564413.2	0.0	GO:0005975	88.00	59.31	35
IRC_IRT_c19819	221	ABH09321.1	0.0	-	82.00	60.08	39
IRC_IRT_c19855	149	YP_001312258.1	0.3	-	55.00	31.96	40
IRC_IRT_c19857	116	KDO85632.1	0.0	GO:0044262	81.00	56.61	37
IRC_IRT_c19858	335	CAE05318.2	0.0	-	86.00	84.34	51

IRC_IRT_c19881	259	BAJ11781.1	0.0	-	77.00	64.70	40
IRC_IRT_c19887	145	BAD38579.1	0.0	GO:0003676	90.00	73.56	40
IRC_IRT_c19904	125	XP_009350806.1	0.0	GO:0030288	82.00	69.32	41
IRC_IRT_c19915	161	NP_001058984.2	0.0	GO:0004553	95.00	100.14	48
IRC_IRT_c19920	169	CAD40991.2	0.0	GO:0016020	100.00	127.10	56
IRC_IRT_c19921	149	KEH20478.1	0.5	-	46.00	33.50	41
IRC_IRT_c19946	174	YP_358636.1	0.0	GO:0044444	71.00	55.45	42
IRC_IRT_c19965	217	XP_002986021.1	0.3	-	53.00	34.65	49
IRC_IRT_c19971	147	NP_001063934.1	0.0	GO:0051287	97.00	71.25	49
IRC_IRT_c19982	219	ERN00555.1	0.3	-	50.00	33.50	51
IRC_IRT_c19987	190	CDM82334.1	0.0	GO:0009507	85.00	62.00	35
IRC_IRT_c19992	111	AGV54793.1	0.0	-	71.00	40.05	35
IRC_IRT_c20010	180	EAY94788.1	0.0	GO:0016023	94.00	74.33	39
IRC_IRT_c20017	174	XP_001786400.1	0.0	-	64.00	50.83	48
IRC_IRT_c20027	237	ACU24411.1	0.2	-	54.00	33.11	48
IRC_IRT_c20029	121	ACG27632.1	0.0	-	68.00	37.74	35
IRC_IRT_c20037	181	XP_003608262.1	0.0	-	59.00	42.36	49
IRC_IRT_c20040	235	AAK51585.1	0.0	GO:0004523	97.00	97.06	42
IRC_IRT_c20058	244	EPS70026.1	0.0	-	67.00	38.51	40
IRC_IRT_c20113	215	KEH15823.1	0.9	-	53.00	33.11	67
IRC_IRT_c20128	250	BAD28639.1	0.0	GO:0005739	80.00	114.01	76
IRC_IRT_c20135	169	AAV25653.1	0.0	GO:0008270	95.00	88.20	46
IRC_IRT_c20163	180	XP_003595724.1	0.0	-	80.00	51.22	36
IRC_IRT_c20173	197	XP_003595724.1	0.0	-	82.00	49.68	34
IRC_IRT_c20179	177	XP_007158784.1	0.5	-	58.00	31.57	39
IRC_IRT_c20183	140	ABG66016.1	0.0	-	88.00	77.41	45
IRC_IRT_c20190	224	XP_006363639.1	0.0	-	89.00	83.57	47
IRC_IRT_c20223	199	XP_009388289.1	0.0	GO:0008152	80.00	60.85	40
IRC_IRT_c20226	146	AGZ19352.1	0.0	GO:0009507	83.00	50.06	36
IRC_IRT_c20240	294	KGN54736.1	0.0	-	64.00	39.28	34
IRC_IRT_c20264	206	XP_003610227.1	0.0	-	75.00	45.05	40
IRC_IRT_c20281	204	BAJ11784.1	0.0	-	48.00	23.48	54
IRC_IRT_c20287	151	EPS74505.1	0.1	-	57.00	35.42	45

IRC_IRT_c20306	105	XP_002534888.1	0.1	-	64.00	33.11	34
IRC_IRT_c20323	165	AGZ19352.1	0.0	-	62.00	37.35	43
IRC_IRT_c20324	336	KFK34620.1	0.3	-	55.00	35.81	45
IRC_IRT_c20350	243	XP_003595724.1	0.0	-	67.00	46.98	46
IRC_IRT_c20353	103	YP_003795541.1	0.0	GO:0003899	79.00	50.06	34
IRC_IRT_c20356	165	AAM01157.2	0.0	-	71.00	46.60	35
IRC_IRT_c20369	145	EMT16104.1	0.0	-	66.00	38.12	36
IRC_IRT_c20378	258	XP_002464700.1	0.0	-	73.00	50.45	34
IRC_IRT_c20394	270	EPS74531.1	0.0	-	70.00	44.67	41
IRC_IRT_c20408	154	ABH09321.1	0.0	-	61.00	52.37	54
IRC_IRT_c20423	140	KIY97451.1	0.5	-	62.00	33.50	37
IRC_IRT_c20425	140	AGC78943.1	0.0	-	69.00	48.91	39
IRC_IRT_c20431	252	KJB31094.1	0.0	-	72.00	46.98	37
IRC_IRT_c20437	179	CDX76121.1	0.9	-	61.00	32.73	47
IRC_IRT_c20438	166	AGV54820.1	0.0	-	65.00	45.44	44
IRC_IRT_c20442	177	CAN77973.1	0.0	-	61.00	38.12	39
IRC_IRT_c20444	172	EPS74511.1	0.0	-	70.00	45.82	51
IRC_IRT_c20446	205	CDY19671.1	0.0	-	66.00	41.20	36
IRC_IRT_c20450	163	BAJ11781.1	0.1	-	57.00	33.50	38
IRC_IRT_c20486	270	XP_003608262.1	0.0	-	63.00	37.74	36
IRC_IRT_c20492	187	BAJ11784.1	0.0	-	81.00	69.32	43
IRC_IRT_c20496	174	EMT23071.1	0.8	-	65.00	33.11	40
IRC_IRT_c20520	209	BAJ11784.1	0.0	-	95.00	78.18	40
IRC_IRT_c20528	315	CDP13647.1	0.6	-	57.00	34.27	54
IRC_IRT_c20536	243	XP_009388289.1	0.0	-	80.00	46.98	35
IRC_IRT_c20538	173	EPS74494.1	0.0	-	66.00	44.67	42
IRC_IRT_c20542	189	KJB31094.1	0.0	-	82.00	52.76	34
IRC_IRT_c20557	155	EPS70026.1	0.0	-	67.00	37.35	37
IRC_IRT_c20558	215	XP_007161040.1	0.0	-	55.00	44.67	56
IRC_IRT_c20569	214	EPS74490.1	0.0	-	67.00	38.89	34
IRC_IRT_c20595	238	EXC01915.1	0.0	-	68.00	39.28	35
IRC_IRT_c20602	210	XP_004306383.1	0.0	-	49.00	37.74	57
IRC_IRT_c20629	163	XP_007161040.1	0.0	-	57.00	35.81	35

IRC_IRT_c20635	212	ABH09321.1	0.0	-	65.00	42.74	43
IRC_IRT_c20668	114	XP_002539786.1	0.0	-	55.00	35.42	38
IRC_IRT_c20672	185	XP_002966336.1	0.0	-	75.00	40.05	37
IRC_IRT_c20673	129	EEC67124.1	0.0	GO:0004325	83.00	70.09	43
IRC_IRT_c20695	260	XP_002535367.1	0.0	-	68.00	56.61	58
IRC_IRT_c20697	163	XP_002540589.1	0.0	-	59.00	35.42	37
IRC_IRT_c20703	115	KEH15984.1	0.0	-	66.00	39.28	39
IRC_IRT_c20705	136	XP_009350059.1	0.0	GO:0003333	84.00	71.63	45
IRC_IRT_c20711	106	XP_002536043.1	0.0	-	65.00	40.05	35
IRC_IRT_c20712	104	XP_010443489.1	0.6	-	50.00	32.34	34
IRC_IRT_c20729	109	XP_010059762.1	0.0	-	70.00	36.97	34
IRC_IRT_c20740	231	NP_085560.1	0.0	-	56.00	35.42	53
IRC_IRT_c20757	169	XP_002537677.1	0.0	-	66.00	52.37	56
IRC_IRT_c20764	184	ABF97018.1	0.0	-	90.00	93.20	54
IRC_IRT_c20776	177	XP_002988985.1	0.0	GO:0016485	90.00	70.09	40
IRC_IRT_c20783	138	NP_001047916.1	0.0	GO:0003723	100.00	77.41	3.50E+01
IRC_IRT_c20795	218	AAK92560.1	0.0	GO:0003964	97.00	80.11	38
IRC_IRT_c20798	140	XP_002538208.1	0.0	GO:0004617	82.00	67.40	45
IRC_IRT_c20799	132	KEH15218.1	0.0	GO:0055085	81.00	63.93	43
IRC_IRT_c20801	162	XP_005642981.1	0.0	GO:0016620	66.00	61.62	54
IRC_IRT_c20814	253	XP_009388289.1	0.0	-	66.00	48.52	36
IRC_IRT_c20829	145	XP_004303592.1	0.0	GO:0006139	67.00	52.76	52
IRC_IRT_c20875	211	EEC68416.1	0.0	GO:0000166	74.00	74.71	59
IRC_IRT_c20880	118	XP_005847687.1	0.0	-	69.00	37.35	36
IRC_IRT_c20886	204	XP_003057599.1	0.7	-	61.00	33.11	36
IRC_IRT_c20894	137	KFM28773.1	0.0	-	64.00	39.66	45
IRC_IRT_c20928	111	XP_002538133.1	0.0	-	71.00	45.82	35
IRC_IRT_c20933	215	BAD69364.1	0.0	-	85.00	101.68	67
IRC_IRT_c20935	151	XP_009394681.1	0.0	GO:0036094	72.00	52.37	47
IRC_IRT_c20957	137	ABA98123.1	0.0	-	73.00	46.21	34
IRC_IRT_c20976	165	EDQ48365.1	0.0	GO:1901363	65.00	48.91	49
IRC_IRT_c21006	141	XP_009389187.1	0.0	-	65.00	37.74	40
IRC_IRT_c21016	139	ABA93901.1	0.0	GO:0008152	82.00	58.15	45

IRC_IRT_c21021	115	XP_002538398.1	0.0	-	57.00	36.58	38
IRC_IRT_c21030	110	XP_002880916.1	0.0	GO:0003700	72.00	54.30	43
IRC_IRT_c21035	114	XP_002538489.1	0.0	GO:0055114	81.00	57.00	37
IRC_IRT_c21039	117	XP_002540486.1	0.0	-	63.00	35.04	38
IRC_IRT_c21046	135	XP_010429166.1	0.0	GO:0048767	80.00	60.85	41
IRC_IRT_c21052	185	BAD52913.1	0.0	GO:0010287	96.00	108.61	53
IRC_IRT_c21056	143	XP_002540141.1	0.4	-	56.00	32.34	39
IRC_IRT_c21063	152	XP_002539918.1	0.0	GO:0031514	92.00	90.12	51
IRC_IRT_c21074	351	BAB40828.1	0.0	GO:0003964	100.00	242.66	116
IRC_IRT_c21097	167	XP_003064237.1	0.3	-	63.00	34.27	44
IRC_IRT_c21125	102	XP_002538475.1	0.0	GO:0009536	76.00	53.53	34
IRC_IRT_c21129	117	BAD23615.1	0.1	-	52.00	35.81	38
IRC_IRT_c21137	141	XP_002948465.1	0.0	GO:0008750	79.00	60.46	43
IRC_IRT_c21138	212	XP_003616487.1	0.2	-	65.00	35.04	38
IRC_IRT_c21142	114	XP_002538125.1	0.0	GO:0004519	78.00	57.00	38
IRC_IRT_c21144	108	P53385.1	0.0	GO:0019557	85.00	58.15	40
IRC_IRT_c21152	116	XP_002959920.1	0.0	GO:0016779	75.00	45.44	37
IRC_IRT_c21157	124	XP_003059591.1	0.0	-	76.00	54.68	39
IRC_IRT_c21164	130	EDQ49165.1	0.0	GO:0044763	78.00	52.37	42
IRC_IRT_c21172	212	XP_001787133.1	0.1	-	50.00	35.81	62
IRC_IRT_c21173	144	XP_002535671.1	0.0	-	84.00	41.20	46
IRC_IRT_c21178	198	EEE56467.1	0.0	GO:0016758	97.00	70.86	36
IRC_IRT_c21183	125	NP_038384.1	0.0	GO:0003899	97.00	88.58	41
IRC_IRT_c21188	110	CDY52947.1	0.0	-	65.00	36.58	38
IRC_IRT_c21199	106	EEE64959.1	0.0	GO:0003824	100.00	78.95	35
IRC_IRT_c21201	145	NP_173170.2	0.2	-	63.00	34.65	41
IRC_IRT_c21203	104	EDQ49152.1	0.6	-	61.00	32.73	34
IRC_IRT_c21223	111	XP_002966999.1	0.0	-	67.00	44.28	34
IRC_IRT_c21229	158	XP_002537545.1	0.3	-	62.00	34.27	40
IRC_IRT_c21253	104	EEC81752.1	0.0	GO:0006468	100.00	73.17	34
IRC_IRT_c21269	112	XP_002538903.1	0.1	-	62.00	34.65	37
IRC_IRT_c21280	120	XP_010230387.1	0.0	GO:1901363	65.00	43.90	40
IRC_IRT_c21284	163	YP_001019104.1	0.0	GO:0003899	83.00	86.27	53

IRC_IRT_c21287	120	ABF96081.1	0.0	GO:0003676	100.00	83.57	39
IRC_IRT_c21290	220	XP_005651897.1	0.0	-	61.00	63.54	71
IRC_IRT_c21294	107	NP_001169545.1	0.0	GO:0009113	82.00	51.22	3.50E+01
IRC_IRT_c21306	122	EEC78085.1	0.0	-	77.00	57.38	40
IRC_IRT_c21308	232	EPS74494.1	0.0	-	67.00	38.51	37
IRC_IRT_c21312	113	ABF83897.1	0.0	GO:0071704	72.00	50.06	37
IRC_IRT_c21317	118	XP_009351102.1	0.0	GO:0071944	76.00	61.23	39
IRC_IRT_c21327	153	XP_008796260.1	0.6	-	59.00	33.11	37
IRC_IRT_c21358	120	XP_005650085.1	0.0	-	70.00	43.13	40
IRC_IRT_c21375	202	XP_002534565.1	0.0	GO:0006812	69.00	57.00	63
IRC_IRT_c21382	319	ABA94439.1	0.0	GO:0003964	94.00	151.37	79
IRC_IRT_c21395	116	NP_001055870.1	0.0	GO:0009536	92.00	70.09	38
IRC_IRT_c21396	158	XP_002537087.1	0.0	GO:0046872	80.00	63.93	51
IRC_IRT_c21397	116	CAE03096.2	0.0	GO:0004523	100.00	87.43	38
IRC_IRT_c21409	111	XP_006305412.1	0.8	-	55.00	31.96	36
IRC_IRT_c21416	126	XP_002535595.1	0.5	-	62.00	32.73	40
IRC_IRT_c21423	117	XP_002540164.1	0.0	-	73.00	36.58	34
IRC_IRT_c21427	130	XP_002537746.1	0.0	GO:0000155	100.00	58.92	43
IRC_IRT_c21429	146	KIY91775.1	0.0	GO:0055085	75.00	57.77	45
IRC_IRT_c21434	132	BAD45255.1	0.0	GO:0016829	97.00	96.29	44
IRC_IRT_c21447	143	XP_002538603.1	0.0	GO:0007165	83.00	73.56	43
IRC_IRT_c21449	190	XP_002539686.1	0.0	GO:0016301	68.00	55.45	57
IRC_IRT_c21458	197	BAD62248.1	0.0	-	76.00	37.74	34
IRC_IRT_c21462	148	CAE02184.2	0.0	GO:0003964	97.00	76.26	36
IRC_IRT_c21468	136	XP_002539100.1	0.0	GO:0008780	86.00	73.56	44
IRC_IRT_c21472	145	ABF95083.1	0.0	-	88.00	72.02	45
IRC_IRT_c21482	290	XP_003588337.1	0.0	-	74.00	55.84	43
IRC_IRT_c21504	117	XP_008453261.1	0.0	GO:0071704	78.00	53.91	38
IRC_IRT_c21506	137	KFM28399.1	0.0	GO:0005524	91.00	74.33	45
IRC_IRT_c21511	138	XP_002529193.1	0.0	GO:0003973	74.00	57.00	43
IRC_IRT_c21515	130	YP_003495137.1	0.0	GO:0016021	86.00	64.31	43
IRC_IRT_c21527	128	EEC66865.1	0.0	GO:0003723	94.00	76.64	38
IRC_IRT_c21528	154	BAD62017.1	0.0	-	100.00	81.65	37

IRC_IRT_c21534	118	XP_009350810.1	0.0	-	72.00	50.06	37
IRC_IRT_c21540	201	XP_002535007.1	0.0	-	74.00	77.41	66
IRC_IRT_c21541	126	XP_005650255.1	0.0	-	60.00	38.12	41
IRC_IRT_c21555	105	XP_009104543.1	0.0	-	71.00	36.97	35
IRC_IRT_c21568	118	XP_010314405.1	0.9	-	56.00	31.96	41
IRC_IRT_c21571	158	XP_007222159.1	0.2	-	55.00	35.04	47
IRC_IRT_c21588	118	XP_008649241.1	0.0	-	62.00	36.58	35
IRC_IRT_c21619	251	XP_006395130.1	0.0	GO:1901576	59.00	58.15	71
IRC_IRT_c21624	138	EEE65799.1	0.0	GO:0005739	93.00	91.28	46
IRC_IRT_c21640	218	EPS74494.1	0.0	-	59.00	43.90	42
IRC_IRT_c21654	139	XP_002537713.1	0.0	-	60.00	36.58	43
IRC_IRT_c21655	166	AAK92589.1	0.0	GO:0016021	100.00	114.39	55
IRC_IRT_c21672	136	XP_002539897.1	0.0	-	76.00	38.12	34
IRC_IRT_c21687	216	XP_002449547.1	0.0	GO:0036459	93.00	83.96	44
IRC_IRT_c21689	289	XP_002540421.1	0.0	-	71.00	36.97	56
IRC_IRT_c21690	132	CDX95796.1	0.3	-	58.00	33.50	43
IRC_IRT_c21710	130	BAD52808.1	0.0	GO:0004553	100.00	83.96	38
IRC_IRT_c21715	191	XP_005650804.1	0.0	GO:0004871	67.00	52.37	58
IRC_IRT_c21722	124	KEH15548.1	0.0	GO:0008152	68.00	47.75	41
IRC_IRT_c21732	144	XP_002536620.1	0.5	-	67.00	33.11	34
IRC_IRT_c21735	121	EPS62505.1	0.0	-	69.00	50.45	39
IRC_IRT_c21744	114	ADP55094.1	0.0	-	62.00	40.43	37
IRC_IRT_c21767	122	XP_003056797.1	0.0	-	66.00	45.05	39
IRC_IRT_c21809	137	XP_009402779.1	0.0	-	66.00	39.28	39
IRC_IRT_c21816	129	EYU45019.1	0.0	GO:0008760	79.00	52.37	43
IRC_IRT_c21823	105	EEE66150.1	0.0	GO:0016023	91.00	65.86	34
IRC_IRT_c21854	137	XP_003600046.1	0.0	GO:0004834	77.00	63.93	45
IRC_IRT_c21855	143	KDD72103.1	0.0	-	65.00	38.89	44
IRC_IRT_c21865	125	XP_002536964.1	0.0	GO:0004872	88.00	52.76	35
IRC_IRT_c21873	222	EPS74505.1	0.0	-	66.00	52.37	42
IRC_IRT_c21878	111	XP_002534856.1	0.3	-	66.00	31.57	36
IRC_IRT_c21885	107	XP_008455174.1	0.5	-	54.00	32.34	35
IRC_IRT_c21897	136	XP_002535866.1	0.0	GO:0005737	79.00	68.55	44

IRC_IRT_c21898	168	AFW67748.1	0.0	GO:0005874	100.00	78.57	35
IRC_IRT_c21904	147	YP_001152215.1	0.0	GO:0044444	68.00	50.45	35
IRC_IRT_c21909	180	KDD71279.1	0.0	GO:0009536	77.00	74.33	58
IRC_IRT_c21941	154	XP_002538858.1	0.0	-	58.00	45.05	43
IRC_IRT_c21947	125	BAJ94980.1	0.2	-	65.00	34.65	35
IRC_IRT_c21952	103	XP_001786754.1	0.0	GO:0006525	88.00	45.05	34
IRC_IRT_c21955	257	EXC34899.1	0.0	-	68.00	42.36	35
IRC_IRT_c21964	293	XP_009350070.1	0.0	-	64.00	36.19	56
IRC_IRT_c21976	130	XP_002959031.1	0.0	GO:0009507	72.00	48.52	40
IRC_IRT_c21977	191	EEE55237.1	0.0	GO:0055114	96.00	128.64	61
IRC_IRT_c21990	206	XP_008447983.1	0.2	-	56.00	34.65	71
IRC_IRT_c21998	118	XP_001770452.1	0.0	GO:0006200	77.00	46.21	35
IRC_IRT_c22014	124	XP_009350805.1	0.0	GO:0006412	77.00	58.15	40
IRC_IRT_c22024	132	XP_001786775.1	0.0	GO:0005524	90.00	68.17	43
IRC_IRT_c22031	273	KEH29749.1	0.0	-	47.00	41.20	73
IRC_IRT_c22036	157	XP_002536704.1	0.2	-	57.00	34.65	54
IRC_IRT_c22042	166	XP_002535920.1	0.0	GO:0032450	94.00	68.17	37
IRC_IRT_c22048	208	XP_009118560.1	0.7	-	56.00	33.11	41
IRC_IRT_c22056	173	XP_001787129.1	0.0	-	60.00	34.27	35
IRC_IRT_c22069	115	KEH15984.1	0.0	GO:0097264	80.00	55.07	35
IRC_IRT_c22107	153	XP_010320012.1	0.5	-	50.00	33.50	48
IRC_IRT_c22152	113	XP_001701676.1	0.0	GO:0007165	67.00	40.43	37
IRC_IRT_c22153	169	KFM23238.1	0.4	-	54.00	33.88	53
IRC_IRT_c22179	167	XP_001698262.1	0.0	GO:0009570	88.00	100.91	53
IRC_IRT_c22192	110	XP_011016603.1	0.0	-	63.00	36.19	36
IRC_IRT_c22195	148	XP_005647659.1	0.2	-	52.00	33.50	46
IRC_IRT_c22207	169	KFM27113.1	0.0	GO:0009536	67.00	42.74	37
IRC_IRT_c22215	147	BAB07815.1	0.0	GO:0000166	76.00	43.90	39
IRC_IRT_c22219	123	XP_002539628.1	0.1	-	60.00	35.04	35
IRC_IRT_c22225	152	CDO98669.1	0.0	-	68.00	43.51	41
IRC_IRT_c22231	137	NP_001068137.2	0.0	GO:0003677	95.00	80.49	43
IRC_IRT_c22253	131	NP_001057851.1	0.0	GO:0005840	97.00	64.70	42
IRC_IRT_c22265	158	EAZ36087.1	0.2	-	63.00	34.65	44

IRC_IRT_c22273	188	XP_002955040.1	0.0	GO:0033036	75.00	65.08	61
IRC_IRT_c22281	139	BAA94214.1	0.0	-	80.00	60.85	36
IRC_IRT_c22309	116	KIY98601.1	0.0	GO:0008446	86.00	71.63	38
IRC_IRT_c22311	163	XP_010911784.1	0.0	-	55.00	43.90	54
IRC_IRT_c22332	133	EEE59222.1	0.0	GO:0009536	78.00	66.63	38
IRC_IRT_c22337	129	EDQ48466.1	0.0	-	62.00	35.04	43
IRC_IRT_c22340	125	CAE02425.2	0.0	-	60.00	42.74	43
IRC_IRT_c22349	107	XP_001415719.1	0.0	GO:0050660	82.00	51.60	34
IRC_IRT_c22357	113	BAD45730.1	0.0	-	100.00	85.50	37
IRC_IRT_c22368	141	XP_006655127.1	0.0	GO:0003964	85.00	62.77	35
IRC_IRT_c22382	108	EAY78583.1	0.0	-	100.00	49.68	35
IRC_IRT_c22388	206	CAD40406.3	0.0	-	67.00	50.45	68
IRC_IRT_c22406	106	XP_002535010.1	0.0	-	64.00	36.58	34
IRC_IRT_c22413	118	XP_006372203.1	0.0	GO:0003676	89.00	64.31	38
IRC_IRT_c22418	151	XP_002527823.1	0.0	GO:0006457	70.00	72.40	67
IRC_IRT_c22423	118	XP_002534672.1	0.0	-	70.00	35.42	34
IRC_IRT_c22431	113	CAD10505.1	0.0	GO:0004028	85.00	58.54	34
IRC_IRT_c22433	103	EEC84015.1	0.0	GO:0008152	100.00	70.09	34
IRC_IRT_c22439	195	XP_003562082.1	0.2	-	53.00	34.65	49
IRC_IRT_c22448	106	XP_002536324.1	0.0	GO:0004341	80.00	53.91	35
IRC_IRT_c22449	109	KIY93288.1	0.3	-	64.00	31.57	34
IRC_IRT_c22492	109	NP_001056886.1	0.0	GO:0005739	100.00	73.56	36
IRC_IRT_c22496	170	XP_002534675.1	0.0	GO:0035556	70.00	59.31	58
IRC_IRT_c22526	172	EAZ05044.1	0.0	GO:0016491	94.00	100.52	56
IRC_IRT_c22557	342	AGC78943.1	0.0	-	56.00	37.35	37
IRC_IRT_c22586	144	XP_002993040.1	0.9	-	58.00	32.34	41
IRC_IRT_c22603	102	XP_010489548.1	0.0	GO:0005618	85.00	54.68	34
IRC_IRT_c22608	139	XP_002538422.1	0.0	GO:0009908	86.00	73.17	46
IRC_IRT_c22620	135	XP_006409122.1	0.0	-	58.00	41.20	50
IRC_IRT_c22665	139	EEC70630.1	0.0	GO:0043531	91.00	82.80	45
IRC_IRT_c22691	206	AGH32904.1	0.0	GO:0003746	100.00	145.59	68
IRC_IRT_c22712	236	XP_002540134.1	0.0	-	62.00	39.66	77
IRC_IRT_c22714	109	KDP23411.1	0.0	GO:0032508	80.00	51.22	35

IRC_IRT_c22716	149	AAF16526.1	0.0	GO:0007010	81.00	52.37	38
IRC_IRT_c22729	145	XP_006387441.1	0.8	-	51.00	32.73	41
IRC_IRT_c22733	103	XP_009350812.1	0.0	-	70.00	46.21	34
IRC_IRT_c22743	151	XP_002456038.1	0.0	-	52.00	41.20	42
IRC_IRT_c22770	152	XP_002505138.1	0.6	-	53.00	33.11	43
IRC_IRT_c22772	131	KDD73869.1	0.0	GO:0044699	75.00	44.28	36
IRC_IRT_c22784	113	XP_009351046.1	0.0	GO:0008233	82.00	57.00	35
IRC_IRT_c22825	167	XP_006826236.1	0.1	-	50.00	36.19	52
IRC_IRT_c22837	251	XP_002489102.1	0.0	-	60.00	40.05	40
IRC_IRT_c22841	128	BAJ34589.1	0.0	GO:0007031	78.00	59.31	41
IRC_IRT_c22846	116	EAY97805.1	0.0	-	91.00	70.09	37
IRC_IRT_c22882	118	KFM27771.1	0.0	GO:0008152	71.00	44.67	39
IRC_IRT_c22889	160	XP_008448801.1	0.1	-	62.00	35.04	48
IRC_IRT_c22891	164	XP_001786746.1	0.0	-	60.00	38.12	41
IRC_IRT_c22905	158	XP_002540411.1	0.0	GO:0055114	75.00	59.31	45
IRC_IRT_c22909	104	AAB61311.1	0.2	-	57.00	33.88	42
IRC_IRT_c22927	131	XP_003078975.1	0.0	GO:0005507	90.00	75.49	43
IRC_IRT_c22933	163	XP_002534677.1	0.0	GO:0050794	75.00	60.46	48
IRC_IRT_c22942	106	EEC74306.1	0.1	-	64.00	34.65	34
IRC_IRT_c22951	136	XP_002507974.1	0.0	-	53.00	36.97	45
IRC_IRT_c22972	145	XP_002948252.1	0.0	-	56.00	38.89	41
IRC_IRT_c23024	134	AAM08627.1	0.0	GO:0004523	82.00	58.92	34
IRC_IRT_c23028	109	EEC77997.1	0.0	GO:0042450	97.00	65.86	35
IRC_IRT_c23038	117	KIY99313.1	0.0	GO:0071704	66.00	46.21	39
IRC_IRT_c23068	144	KJB70098.1	0.0	-	68.00	36.58	45
IRC_IRT_c23074	145	XP_002963016.1	0.8	-	61.00	32.73	44
IRC_IRT_c23078	166	XP_009351370.1	0.6	-	58.00	33.11	50
IRC_IRT_c23080	183	XP_010917066.1	0.0	GO:0008152	70.00	61.62	51
IRC_IRT_c23115	117	XP_006404419.1	0.0	-	63.00	36.97	36
IRC_IRT_c23147	114	AEA11197.1	0.0	GO:0004129	83.00	62.00	37
IRC_IRT_c23154	124	XP_002536675.1	0.0	-	63.00	48.14	38
IRC_IRT_c23156	222	AAQ56338.1	0.0	GO:0003676	93.00	96.29	61
IRC_IRT_c23174	128	XP_002536583.1	0.4	-	58.00	33.50	41

IRC_IRT_c23175	154	BAK01051.1	0.0	GO:0005840	80.00	58.15	35
IRC_IRT_c23183	152	AAX94899.1	0.0	GO:0008270	94.00	97.44	50
IRC_IRT_c23207	136	XP_006390493.1	0.6	-	64.00	32.73	37
IRC_IRT_c23209	156	EEE67517.1	0.0	GO:0009536	91.00	82.80	45
IRC_IRT_c23228	193	CAH68538.2	0.0	-	60.00	59.31	66
IRC_IRT_c23233	148	XP_006590782.1	0.9	-	51.00	32.34	45
IRC_IRT_c23238	170	XP_002538815.1	0.0	-	81.00	56.23	37
IRC_IRT_c23240	116	EEC79944.1	0.0	-	74.00	41.97	39
IRC_IRT_c23273	119	XP_002536630.1	0.0	-	60.00	37.74	38
IRC_IRT_c23287	113	YP_009045731.1	0.0	-	63.00	35.04	38
IRC_IRT_c23333	127	EEE52320.1	0.0	GO:0055085	88.00	60.85	34
IRC_IRT_c23353	197	ABA98320.1	0.0	GO:0003964	98.00	124.02	57
IRC_IRT_c23373	169	EAZ02160.1	0.0	-	98.00	107.07	56
IRC_IRT_c23380	132	AAU03108.1	0.0	-	85.00	60.46	35
IRC_IRT_c23391	160	XP_002536536.1	0.0	-	75.00	62.77	49
IRC_IRT_c23429	153	XP_005644006.1	0.0	GO:0005982	73.00	67.40	46
IRC_IRT_c23497	168	XP_005846291.1	0.0	-	67.00	59.31	52
IRC_IRT_c23509	115	AAM22008.1	0.0	GO:0003964	100.00	77.41	36
IRC_IRT_c23528	130	KIY97548.1	0.0	-	69.00	37.35	39
IRC_IRT_c23544	134	AAK71558.1	0.0	GO:0004523	100.00	95.52	44
IRC_IRT_c23551	104	NP_001046128.1	0.0	GO:0006071	100.00	74.71	3.40E+01
IRC_IRT_c23556	181	EDQ48547.1	0.0	-	63.00	57.00	60
IRC_IRT_c23576	105	ABA96804.1	0.0	GO:0003676	100.00	68.17	34
IRC_IRT_c23580	234	XP_002537581.1	0.0	-	65.00	60.08	70
IRC_IRT_c23609	110	XP_002985144.1	0.0	GO:0000166	77.00	46.21	35
IRC_IRT_c23649	162	EMS64720.1	0.9	-	54.00	32.73	37
IRC_IRT_c23702	137	XP_002534675.1	0.0	GO:0009987	73.00	41.59	34
IRC_IRT_c23708	159	EAY97655.1	0.0	GO:0003677	100.00	111.31	53
IRC_IRT_c23730	103	XP_002534929.1	0.0	-	100.00	62.77	34
IRC_IRT_c23747	179	XP_001696295.1	0.2	-	52.00	35.04	36
IRC_IRT_c23760	109	ABA97975.1	0.0	GO:0003677	100.00	77.03	34
IRC_IRT_c23763	132	EAY93131.1	0.5	-	66.00	33.11	45
IRC_IRT_c23816	172	XP_011016369.1	0.0	-	62.00	49.29	54

IRC_IRT_c23833	107	CAJ86252.1	0.0	GO:0016021	100.00	73.94	35
IRC_IRT_c23834	142	ABA98827.2	0.0	GO:0003964	93.00	91.28	47
IRC_IRT_c23854	151	XP_001420863.1	0.0	GO:0006457	76.00	66.63	50
IRC_IRT_c23878	172	BAD62358.1	0.0	-	100.00	92.82	44
IRC_IRT_c23915	138	BAD25140.1	0.0	-	100.00	87.43	43
IRC_IRT_c23918	157	EEC77856.1	0.3	-	60.00	34.27	46
IRC_IRT_c23930	171	XP_001702941.1	0.0	GO:0005739	66.00	58.54	54
IRC_IRT_c23936	170	ADN67714.1	0.0	GO:0005488	67.00	55.84	53
IRC_IRT_c23950	106	ABF99402.1	0.0	GO:0005739	100.00	72.79	34
IRC_IRT_c23957	147	AAX92789.1	0.0	GO:0003964	100.00	81.26	3.80E+01
IRC_IRT_c23974	117	NP_001174892.1	0.0	GO:0004523	97.00	72.79	39
IRC_IRT_c23995	153	EMT00231.1	0.0	GO:0006355	80.00	57.77	47
IRC_IRT_c24004	170	XP_008659695.1	0.7	-	62.00	32.73	37
IRC_IRT_c24028	118	XP_011015413.1	0.0	-	61.00	40.05	39
IRC_IRT_c24033	128	BAC10208.1	0.0	-	83.00	66.24	43
IRC_IRT_c24051	108	XP_002507215.1	0.0	-	64.00	36.58	34
IRC_IRT_c24086	186	XP_008439064.1	0.0	-	47.00	38.12	61
IRC_IRT_c24108	109	EEC68416.1	0.0	GO:0005739	75.00	43.51	36
IRC_IRT_c24122	214	EEC82953.1	0.0	-	100.00	85.50	39
IRC_IRT_c24145	117	XP_002540411.1	0.0	GO:0008233	69.00	42.74	39
IRC_IRT_c24151	102	EMT00280.1	0.0	GO:0004019	82.00	52.76	34
IRC_IRT_c24152	145	XP_002534565.1	0.0	GO:0044763	73.00	53.53	42
IRC_IRT_c24166	158	XP_002534909.1	0.0	-	64.00	50.45	51
IRC_IRT_c24178	121	KIY95681.1	0.0	GO:0098655	90.00	63.54	40
IRC_IRT_c24210	107	KIY92252.1	0.0	-	71.00	36.19	35
IRC_IRT_c24214	124	XP_004246113.1	0.2	-	56.00	34.27	37
IRC_IRT_c24229	106	ABR25884.1	0.0	-	100.00	74.33	34
IRC_IRT_c24244	138	XP_002539401.1	0.0	-	62.00	38.89	40
IRC_IRT_c24254	112	CCO18806.1	0.0	-	64.00	38.51	37
IRC_IRT_c24272	219	ABA94454.1	0.0	GO:0006457	82.00	115.55	70
IRC_IRT_c24292	126	NP_001175631.1	0.0	-	100.00	38.51	42
IRC_IRT_c24384	139	AGB51441.1	0.0	GO:0006096	82.00	67.78	46
IRC_IRT_c24432	340	ABR26094.1	0.0	-	54.00	37.35	53

IRC_IRT_c24433	154	XP_008780541.1	0.0	-	60.00	36.58	46
IRC_IRT_c24507	113	ABA92091.1	0.0	-	97.00	68.94	37
IRC_IRT_c24525	334	KGN54736.1	0.0	-	65.00	50.83	46
IRC_IRT_c24573	148	XP_002535563.1	0.0	-	59.00	40.82	47
IRC_IRT_c24599	117	EYU25822.1	0.9	-	63.00	32.34	41
IRC_IRT_c24608	119	XP_002535066.1	0.0	GO:0033608	79.00	48.52	39
IRC_IRT_c24661	186	XP_003578266.2	0.7	-	56.00	33.50	46
IRC_IRT_c24681	158	XP_009620500.1	0.0	GO:0008233	67.00	43.51	43
IRC_IRT_c24682	118	XP_002538077.1	0.0	-	71.00	40.05	38
IRC_IRT_c24714	107	XP_002536843.1	0.0	-	77.00	46.98	35
IRC_IRT_c24717	116	EAY93131.1	0.0	-	71.00	42.36	38
IRC_IRT_c24749	104	NP_001044670.1	0.0	GO:0016020	97.00	68.17	34
IRC_IRT_c24764	152	ABA98871.1	0.0	-	92.00	74.71	38
IRC_IRT_c24774	169	XP_002501397.1	0.1	-	47.00	33.50	48
IRC_IRT_c24785	255	XP_006396377.1	0.3	-	47.00	34.65	42
IRC_IRT_c24818	136	ACN23298.1	0.0	GO:0005840	80.00	61.62	45
IRC_IRT_c24837	124	XP_010451905.1	0.2	-	70.00	34.27	34
IRC_IRT_c24866	121	CAB82618.1	0.9	-	61.00	31.96	34
IRC_IRT_c24872	145	CEF97688.1	0.0	-	68.00	39.66	47
IRC_IRT_c24880	112	AAO66567.1	0.0	-	97.00	82.42	37
IRC_IRT_c24887	107	AAL76087.1	0.0	-	94.00	38.51	35
IRC_IRT_c24924	151	NP_001058397.1	0.0	GO:0004553	100.00	105.15	50
IRC_IRT_c24940	103	EEC68513.1	0.0	GO:0005783	100.00	73.56	34
IRC_IRT_c24951	115	EAZ41256.1	0.0	GO:0016874	100.00	80.11	38
IRC_IRT_c24967	111	KFM27236.1	0.0	GO:0006457	91.00	64.70	35
IRC_IRT_c24975	117	XP_001416942.1	0.0	GO:0016874	71.00	57.00	38
IRC_IRT_c24987	138	AAX96250.1	0.0	-	75.00	55.84	41
IRC_IRT_c25055	152	YP_008816117.1	0.0	GO:0046961	92.00	73.17	38
IRC_IRT_c25063	179	CAE03619.3	0.0	GO:0003964	100.00	123.64	59
IRC_IRT_c25075	180	ABA97429.1	0.0	GO:0003676	97.00	95.90	45
IRC_IRT_c25080	103	CAE03289.2	0.0	GO:0009536	73.00	46.21	34
IRC_IRT_c25099	119	EEC68416.1	0.2	-	58.00	33.88	39
IRC_IRT_c25114	123	XP_004506876.1	0.0	-	71.00	40.82	35

IRC_IRT_c25139	139	ERN20444.1	0.1	-	55.00	33.11	45
IRC_IRT_c25174	128	BAA90506.1	0.0	GO:0016020	100.00	78.18	36
IRC_IRT_c25187	186	KDP20462.1	0.0	-	67.00	43.13	37
IRC_IRT_c25189	116	XP_002536918.1	0.0	-	62.00	40.82	37
IRC_IRT_c25235	124	KGN48534.1	0.4	-	54.00	33.11	35
IRC_IRT_c25254	162	XP_002540423.1	0.0	-	62.00	39.28	48
IRC_IRT_c25270	136	BAD62213.1	0.0	GO:0009536	86.00	69.71	43
IRC_IRT_c25273	147	XP_002953444.1	0.0	GO:0044765	71.00	45.82	38
IRC_IRT_c25282	108	XP_011016515.1	0.0	GO:0003723	80.00	52.37	35
IRC_IRT_c25292	166	AFK33626.1	0.0	GO:0005488	55.00	45.05	54
IRC_IRT_c25294	161	CDP01824.1	0.0	GO:0090305	78.00	64.31	52
IRC_IRT_c25295	137	BAB21179.1	0.0	GO:0016874	95.00	88.97	45
IRC_IRT_c25343	174	XP_001690955.1	0.0	GO:0008152	74.00	59.31	58
IRC_IRT_c25353	118	CAE02453.2	0.0	GO:0004523	97.00	90.51	39
IRC_IRT_c25438	136	CDX78827.1	0.5	-	64.00	33.11	34
IRC_IRT_c25443	109	XP_002539786.1	0.2	-	62.00	33.50	37
IRC_IRT_c25449	104	XP_005648519.1	0.4	-	67.00	33.11	34
IRC_IRT_c25456	141	XP_002536363.1	0.0	-	72.00	64.70	47
IRC_IRT_c25495	125	CAA87756.1	0.0	GO:0005739	67.00	43.51	40
IRC_IRT_c25500	104	XP_002443712.1	0.0	GO:0019538	97.00	66.63	34
IRC_IRT_c25509	155	EDQ48365.1	0.0	GO:0003677	72.00	48.52	36
IRC_IRT_c25525	112	XP_002460956.1	0.0	-	65.00	37.74	35
IRC_IRT_c25531	126	ABA99479.1	0.0	GO:0004523	80.00	64.31	40
IRC_IRT_c25539	217	P04966.1	0.0	GO:0016491	100.00	79.72	37
IRC_IRT_c25604	224	EEC77040.1	0.0	-	68.00	57.00	47
IRC_IRT_c25645	115	BAC05649.1	0.0	-	72.00	51.22	36
IRC_IRT_c25649	107	EEE55243.1	0.0	GO:0003676	100.00	80.11	35
IRC_IRT_c25668	143	AAA17801.1	0.0	-	58.00	36.97	43
IRC_IRT_c25680	104	XP_002536714.1	0.8	-	67.00	32.34	34
IRC_IRT_c25688	125	CDP16756.1	0.0	GO:0044763	70.00	55.45	41
IRC_IRT_c25831	114	EEE69543.1	0.0	GO:0016023	100.00	80.49	38
IRC_IRT_c25878	127	XP_005851875.1	0.0	GO:0055114	90.00	70.86	42
IRC_IRT_c25895	150	XP_006340294.1	0.5	-	44.00	33.50	38

IRC_IRT_c25957	112	XP_002535661.1	0.0	-	76.00	39.28	34
IRC_IRT_c25964	111	AAM76353.1	0.0	GO:0016023	100.00	80.11	3.60E+01
IRC_IRT_c25970	212	AAS07074.1	0.0	GO:0004523	87.00	122.87	65
IRC_IRT_c25974	133	ABF96920.1	0.0	-	65.00	49.29	38
IRC_IRT_c25979	104	XP_001418457.1	0.0	GO:0009845	91.00	57.38	34
IRC_IRT_c26002	155	CDP10488.1	0.0	-	65.00	40.43	52
IRC_IRT_c26042	143	KFM26505.1	0.0	-	65.00	41.20	47
IRC_IRT_c26048	139	AAK13116.1	0.0	GO:0003964	100.00	109.38	46
IRC_IRT_c26069	104	AAL58148.1	0.0	GO:0008270	91.00	66.63	34
IRC_IRT_c26077	132	XP_002536098.1	0.0	-	72.00	42.36	43
IRC_IRT_c26102	130	XP_005846828.1	0.0	GO:0009451	83.00	58.15	37
IRC_IRT_c26105	103	XP_002537142.1	0.0	-	70.00	36.58	34
IRC_IRT_c26151	104	EEC73611.1	0.0	GO:0016021	100.00	73.56	34
IRC_IRT_c26160	131	BAD52733.1	0.0	-	73.00	36.58	38
IRC_IRT_c26181	104	XP_003082663.1	0.3	-	67.00	33.50	34
IRC_IRT_c26198	147	XP_008344792.1	0.0	GO:0044763	80.00	64.31	47
IRC_IRT_c26227	134	XP_005851921.1	0.5	-	54.00	33.11	44
IRC_IRT_c26255	108	KDD72302.1	0.0	GO:0019538	77.00	50.06	35
IRC_IRT_c26291	109	KFM25982.1	0.0	-	69.00	36.58	39
IRC_IRT_c26319	142	XP_009132208.1	0.0	GO:0005739	75.00	58.54	41
IRC_IRT_c26386	112	XP_006578994.1	0.8	-	65.00	32.34	35
IRC_IRT_c26456	114	ACN39938.1	0.0	-	67.00	40.82	34
IRC_IRT_c26479	140	ABD91504.1	0.0	GO:0003964	94.00	74.33	38
IRC_IRT_c26502	130	CAH68356.1	0.0	GO:0006468	100.00	93.97	43
IRC_IRT_c26504	149	XP_006338471.1	0.0	GO:0005975	78.00	53.91	38
IRC_IRT_c26507	126	AAU90132.1	0.6	-	56.00	32.34	37
IRC_IRT_c26527	165	XP_004507162.1	0.3	-	57.00	34.27	38
IRC_IRT_c26559	198	XP_001421219.1	0.0	GO:0006520	68.00	50.83	44
IRC_IRT_c26576	108	AFK31283.1	0.0	GO:0009536	100.00	73.56	34
IRC_IRT_c26630	136	EEC79491.1	0.0	GO:0090305	80.00	58.54	41
IRC_IRT_c26640	119	XP_002456818.1	0.0	-	94.00	64.70	34
IRC_IRT_c26656	145	ABA98371.1	0.0	GO:0003964	100.00	102.83	47
IRC_IRT_c26696	103	AAT47012.1	0.0	-	76.00	48.52	34

IRC_IRT_c26704	102	EEE65135.1	0.0	-	94.00	56.23	34
IRC_IRT_c26714	134	EEE54984.1	0.0	GO:0005524	81.00	64.70	44
IRC_IRT_c26723	145	AAX96404.1	0.0	-	65.00	49.29	47
IRC_IRT_c26734	165	ERN20006.1	0.8	-	58.00	33.11	39
IRC_IRT_c26741	141	BAL41367.1	0.6	-	59.00	33.11	42
IRC_IRT_c26781	171	CAH66809.1	0.0	GO:0005739	94.00	101.68	56
IRC_IRT_c26806	179	AAM74257.1	0.0	GO:0008152	81.00	62.77	37
IRC_IRT_c26931	131	KFK41888.1	0.0	-	70.00	38.51	41
IRC_IRT_c26937	197	XP_002538985.1	0.0	GO:0032508	82.00	85.11	62
IRC_IRT_c26942	161	AAF16526.1	0.0	GO:0007010	84.00	72.40	51
IRC_IRT_c26949	144	AAK16189.1	0.0	GO:0004523	100.00	92.82	4.60E+01
IRC_IRT_c26959	174	AAX96623.1	0.0	GO:0005739	70.00	46.21	37
IRC_IRT_c26964	112	XP_001786908.1	0.0	-	76.00	43.13	34
IRC_IRT_c26972	136	NP_001059039.1	0.3	-	48.00	33.88	39
IRC_IRT_c26981	161	EEE68923.1	0.0	GO:0003677	96.00	100.52	51
IRC_IRT_c26987	110	NP_001052469.1	0.0	GO:0047213	100.00	77.03	36
IRC_IRT_c26990	113	CAE02132.2	0.0	GO:0016740	70.00	44.28	34
IRC_IRT_c27005	139	XP_003080093.1	0.0	GO:0003824	60.00	46.21	45
IRC_IRT_c27011	125	BAJ92941.1	0.0	-	70.00	46.21	34
IRC_IRT_c27045	174	XP_008466644.1	0.0	-	58.00	38.51	56
IRC_IRT_c27069	107	XP_002534653.1	0.0	GO:0016301	85.00	57.77	35
IRC_IRT_c27079	125	XP_011086754.1	0.0	GO:0016151	87.00	66.24	41
IRC_IRT_c27081	115	XP_009350809.1	0.0	-	78.00	49.68	38
IRC_IRT_c27087	230	KDO62339.1	0.4	-	60.00	34.27	43
IRC_IRT_c27105	101	EEE56632.1	0.3	-	53.00	33.11	39
IRC_IRT_c27113	234	ACU24411.1	0.0	GO:0016787	76.00	65.08	51
IRC_IRT_c27143	113	EEC77198.1	0.3	-	57.00	33.50	40
IRC_IRT_c27153	122	NP_001063675.1	0.0	-	100.00	91.66	40
IRC_IRT_c27177	108	XP_002536388.1	0.0	GO:0004872	80.00	53.14	35
IRC_IRT_c27198	108	XP_005843828.1	0.0	GO:0000166	72.00	50.06	36
IRC_IRT_c27205	154	AFZ40241.1	0.0	GO:0046872	100.00	67.01	44
IRC_IRT_c27247	273	KIY99236.1	0.0	GO:0042558	97.00	68.94	36
IRC_IRT_c27259	134	XP_001691110.1	0.4	-	60.00	33.50	45

IRC_IRT_c27266	133	ABK25243.1	0.7	-	54.00	31.96	37
IRC_IRT_c27272	176	EEC71846.1	0.0	GO:0006355	97.00	49.68	40
IRC_IRT_c27297	155	XP_009800814.1	0.9	-	65.00	32.73	40
IRC_IRT_c27321	111	XP_002468235.1	0.1	-	54.00	34.27	35
IRC_IRT_c27328	106	CDX81369.1	0.5	-	67.00	33.11	34
IRC_IRT_c27351	137	Q653V6.1	0.3	-	61.00	33.88	44
IRC_IRT_c27352	108	XP_009378388.1	0.0	-	68.00	38.12	35
IRC_IRT_c27387	138	XP_002536457.1	0.3	-	53.00	34.27	39
IRC_IRT_c27413	138	XP_011088507.1	0.5	-	48.00	33.50	37
IRC_IRT_c27419	143	CAE04518.2	0.0	GO:0016023	100.00	98.21	46
IRC_IRT_c27420	240	XP_002443370.1	0.7	-	52.00	33.88	48
IRC_IRT_c27422	103	CCO66486.1	0.0	GO:0042026	88.00	54.68	34
IRC_IRT_c27455	133	ABA94657.1	0.0	GO:0016020	97.00	86.27	44
IRC_IRT_c27474	144	XP_002540564.1	0.0	-	58.00	36.97	39
IRC_IRT_c27526	144	AAP55184.2	0.0	GO:0003676	100.00	101.68	47
IRC_IRT_c27535	129	EAY81637.1	0.0	-	57.00	39.28	42
IRC_IRT_c27565	110	XP_002535850.1	0.0	-	75.00	38.89	36
IRC_IRT_c27567	124	BAD08827.1	0.0	GO:0006468	100.00	86.27	41
IRC_IRT_c27576	120	XP_009350805.1	0.0	-	72.00	50.45	40
IRC_IRT_c27589	134	AGZ13631.1	0.0	-	78.00	36.97	42
IRC_IRT_c27595	132	EEE56331.1	0.7	-	67.00	32.73	34
IRC_IRT_c27624	105	CAH67755.1	0.0	GO:0004523	94.00	71.25	35
IRC_IRT_c27670	181	KEH15984.1	0.0	GO:0097264	83.00	96.67	60
IRC_IRT_c27694	106	CBL51500.1	0.0	-	70.00	37.74	34
IRC_IRT_c27781	104	XP_002537652.1	0.0	GO:0008124	85.00	52.76	34
IRC_IRT_c27799	126	XP_002539829.1	0.0	GO:0046872	80.00	48.52	41
IRC_IRT_c27822	177	XP_002538023.1	0.0	-	65.00	43.90	47
IRC_IRT_c27830	119	AAG12666.1	0.0	GO:0050789	75.00	52.76	40
IRC_IRT_c27837	111	XP_002968304.1	0.0	GO:0030145	83.00	56.23	36
IRC_IRT_c27839	202	XP_008371677.1	0.2	-	64.00	35.04	39
IRC_IRT_c27889	115	XP_002537069.1	0.0	-	83.00	56.61	36
IRC_IRT_c27901	107	EEE60864.1	0.0	GO:0009536	100.00	77.41	35
IRC_IRT_c27941	103	EEE51370.1	0.0	GO:0006355	100.00	68.17	34

IRC_IRT_c27980	120	XP_010053446.1	0.5	-	57.00	33.11	40
IRC_IRT_c28007	140	EEC71124.1	0.0	-	67.00	52.76	46
IRC_IRT_c28030	129	XP_006353096.1	0.1	-	54.00	36.19	35
IRC_IRT_c28033	226	CBI34502.3	0.3	-	49.00	34.27	85
IRC_IRT_c28045	131	XP_009364931.1	0.0	GO:0016884	73.00	51.60	42
IRC_IRT_c28063	110	XP_002518200.1	0.0	GO:0031977	85.00	50.45	35
IRC_IRT_c28072	178	AAV44188.1	0.0	GO:0006259	74.00	62.39	43
IRC_IRT_c28074	109	AHZ63894.1	0.0	GO:0004672	71.00	41.59	35
IRC_IRT_c28088	150	BAD54184.1	0.0	-	85.00	47.75	34
IRC_IRT_c28123	119	XP_002960018.1	0.0	-	70.00	45.44	37
IRC_IRT_c28148	146	XP_002540262.1	0.0	-	65.00	42.36	44
IRC_IRT_c28193	196	XP_002957537.1	0.8	-	56.00	32.73	41
IRC_IRT_c28242	130	AAO38504.1	0.0	GO:0004523	95.00	82.80	41
IRC_IRT_c28259	173	XP_003629664.1	0.1	-	58.00	36.19	53
IRC_IRT_c28260	111	XP_004142925.1	0.0	GO:0006457	86.00	60.46	36
IRC_IRT_c28301	102	BAD26318.1	0.0	GO:0009536	88.00	58.54	34
IRC_IRT_c28308	120	KFM28098.1	0.0	GO:0005950	78.00	56.23	41
IRC_IRT_c28318	155	XP_002966092.1	0.3	-	46.00	33.50	47
IRC_IRT_c28323	170	XP_004954507.1	0.7	-	51.00	33.11	45
IRC_IRT_c28341	104	AAN05387.1	0.0	GO:0005739	97.00	74.33	34
IRC_IRT_c28346	108	XP_011016758.1	0.0	-	75.00	47.75	36
IRC_IRT_c28383	121	CAH66849.1	0.0	GO:0008270	100.00	87.43	40
IRC_IRT_c28388	126	ABA98432.1	0.0	GO:0005739	100.00	92.05	42
IRC_IRT_c28411	135	ABA95792.1	0.0	-	95.00	90.89	44
IRC_IRT_c28447	132	XP_002540071.1	0.0	-	64.00	45.05	45
IRC_IRT_c28456	163	XP_001696113.1	0.1	-	58.00	35.42	39
IRC_IRT_c28473	155	BAD52968.1	0.0	-	73.00	50.06	45
IRC_IRT_c28492	141	XP_005644625.1	0.0	GO:0016772	68.00	50.45	44
IRC_IRT_c28495	154	ABA97826.1	0.0	GO:0003964	89.00	98.60	48
IRC_IRT_c28516	146	XP_002537190.1	0.1	-	71.00	35.04	35
IRC_IRT_c28517	162	XP_004497127.1	0.8	-	56.00	32.73	37
IRC_IRT_c28524	123	XP_003083931.1	0.0	GO:0006139	70.00	46.21	40
IRC_IRT_c28569	190	XP_002535112.1	0.0	-	65.00	63.16	64

IRC_IRT_c28588	137	XP_002535127.1	0.0	-	73.00	50.83	41
IRC_IRT_c28615	119	XP_010911357.1	0.0	-	71.00	48.91	38
IRC_IRT_c28623	166	XP_002509042.1	0.0	GO:0016812	74.00	73.56	50
IRC_IRT_c28624	130	AAK13139.1	0.0	-	87.00	70.48	39
IRC_IRT_c28636	185	XP_008221619.1	0.2	-	55.00	34.65	43
IRC_IRT_c28652	138	XP_002503191.1	0.0	GO:0044249	62.00	46.21	40
IRC_IRT_c28689	221	XP_001691469.1	0.1	-	52.00	36.58	69
IRC_IRT_c28702	188	AFK36365.1	0.0	-	69.00	52.76	46
IRC_IRT_c28791	134	XP_011087780.1	0.0	-	62.00	41.59	35
IRC_IRT_c28796	113	EAZ38647.1	0.0	-	97.00	76.64	37
IRC_IRT_c28817	147	XP_008666058.1	0.0	-	68.00	38.12	35
IRC_IRT_c28875	174	XP_002536731.1	0.0	-	60.00	55.07	56
IRC_IRT_c28912	125	XP_002508894.1	0.0	-	62.00	38.89	45
IRC_IRT_c28916	105	EEE51040.1	0.0	-	65.00	36.19	35
IRC_IRT_c28943	103	NP_001050269.1	0.0	GO:0004197	94.00	71.25	34
IRC_IRT_c29012	118	XP_002951253.1	0.0	GO:0006952	78.00	58.92	38
IRC_IRT_c29087	162	XP_009351102.1	0.0	GO:0050896	64.00	56.23	50
IRC_IRT_c29089	106	EEC84903.1	0.0	-	100.00	81.65	35
IRC_IRT_c29116	220	NP_001055735.2	0.0	-	72.00	43.51	40
IRC_IRT_c29133	212	XP_009350076.1	0.0	GO:0005525	91.00	120.94	69
IRC_IRT_c29196	163	CEF98819.1	0.0	-	65.00	41.20	46
IRC_IRT_c29198	198	XP_010911647.1	0.0	GO:0044763	63.00	52.37	58
IRC_IRT_c29221	130	XP_011078687.1	0.0	-	54.00	38.12	42
IRC_IRT_c29223	138	EEE56868.1	0.0	-	67.00	44.28	49
IRC_IRT_c29247	114	AFW87085.1	0.0	-	75.00	51.22	37
IRC_IRT_c29282	153	XP_006655424.1	0.0	-	71.00	43.13	35
IRC_IRT_c29329	143	XP_005848690.1	0.0	-	76.00	50.45	38
IRC_IRT_c29376	166	BAC57723.1	0.0	GO:1901363	61.00	55.07	47
IRC_IRT_c29424	273	ERN10593.1	0.0	-	63.00	64.31	69
IRC_IRT_c29433	113	XP_002458773.1	0.0	GO:0009414	100.00	85.11	37
IRC_IRT_c29457	120	AAT93953.1	0.0	GO:0005488	78.00	53.14	42
IRC_IRT_c29479	195	ACG36692.1	0.9	-	62.00	32.73	35
IRC_IRT_c29486	166	EEC79491.1	0.0	GO:0090305	90.00	93.97	55

IRC_IRT_c29496	248	AAF16526.1	0.0	GO:0007010	87.00	133.27	82
IRC_IRT_c29544	147	AJP15821.1	0.0	GO:0050896	78.00	55.07	41
IRC_IRT_c29591	230	XP_001754198.1	0.0	GO:0009058	76.00	86.66	68
IRC_IRT_c29638	177	NP_001148743.1	0.4	-	55.00	33.88	36
IRC_IRT_c29660	220	KDO58105.1	0.7	-	51.00	33.11	56
IRC_IRT_c29672	158	XP_001764007.1	0.0	GO:0008270	82.00	81.26	52
IRC_IRT_c29680	113	YP_665665.1	0.0	GO:0016491	70.00	47.75	34
IRC_IRT_c29683	127	KIY92445.1	0.3	-	57.00	33.50	40
IRC_IRT_c29699	151	BAM28958.1	0.3	-	60.00	32.73	35
IRC_IRT_c29718	130	EAY82994.1	0.0	GO:0008171	100.00	51.60	38
IRC_IRT_c29732	115	XP_002438831.1	0.4	-	57.00	32.73	35
IRC_IRT_c29735	119	XP_002537063.1	0.0	-	71.00	48.14	39
IRC_IRT_c29736	116	BAJ87747.1	0.0	GO:0006631	67.00	43.13	37
IRC_IRT_c29739	155	BAD73664.1	0.0	GO:0003676	82.00	56.61	34
IRC_IRT_c29755	201	XP_002462181.1	0.3	-	61.00	32.73	36
IRC_IRT_c29758	185	CDY34890.1	0.0	-	70.00	37.35	34
IRC_IRT_c29778	121	XP_008437601.1	0.0	-	66.00	47.37	39
IRC_IRT_c29799	110	NP_001046670.2	0.0	-	100.00	80.11	36
IRC_IRT_c29825	184	BAD46011.1	0.0	-	86.00	80.49	45
IRC_IRT_c29843	104	XP_001420333.1	0.1	-	69.00	35.42	36
IRC_IRT_c29892	110	BAC83869.1	0.0	GO:0005739	97.00	67.40	36
IRC_IRT_c29903	169	KEH15415.1	0.0	GO:0003677	72.00	61.23	51
IRC_IRT_c29917	128	XP_002508034.1	0.0	GO:0016746	70.00	41.97	34
IRC_IRT_c29933	147	XP_010938070.1	0.6	-	60.00	33.11	50
IRC_IRT_c29939	147	XP_002539706.1	0.0	GO:0016787	68.00	51.22	44
IRC_IRT_c29954	102	EAZ02135.1	0.0	GO:0016023	70.00	50.83	34
IRC_IRT_c29975	122	ABF94662.1	0.0	GO:0008270	97.00	87.43	40
IRC_IRT_c29991	103	EEC82172.1	0.0	-	100.00	72.40	34
IRC_IRT_c29997	105	XP_002538706.1	0.0	GO:0009908	94.00	67.01	34
IRC_IRT_c30011	119	ABF98839.1	0.0	-	97.00	79.34	39
IRC_IRT_c30014	111	XP_003547607.1	0.0	-	76.00	42.36	34
IRC_IRT_c30015	128	XP_011016741.1	0.0	-	61.00	43.90	42
IRC_IRT_c30033	119	XP_001786845.1	0.0	-	76.00	47.37	38

IRC_IRT_c30042	106	NP_001046392.2	0.0	GO:0046872	100.00	73.17	35
IRC_IRT_c30069	167	KEH15186.1	0.7	-	55.00	32.73	49
IRC_IRT_c30073	118	KDD76630.1	0.0	GO:0016772	71.00	51.22	38
IRC_IRT_c30105	176	EYU37814.1	0.8	-	66.00	32.73	39
IRC_IRT_c30110	109	XP_001785112.1	0.0	GO:0016301	72.00	55.84	36
IRC_IRT_c30124	153	EEE58440.1	0.0	GO:0055085	77.00	57.38	48
IRC_IRT_c30128	114	EAY95687.1	0.0	GO:0046983	100.00	72.40	37
IRC_IRT_c30179	131	XP_009385754.1	0.0	GO:0016021	86.00	71.25	38
IRC_IRT_c30196	129	BAC78591.1	0.0	GO:0048827	67.00	53.91	43
IRC_IRT_c30203	198	XP_002537909.1	0.0	-	52.00	42.36	65
IRC_IRT_c30209	123	XP_002540019.1	0.0	-	69.00	40.82	36
IRC_IRT_c30219	151	XP_002961441.1	0.0	GO:0043231	62.00	47.75	48
IRC_IRT_c30231	241	XP_004292706.1	0.0	GO:0033014	55.00	36.58	54
IRC_IRT_c30232	103	XP_002979914.1	0.0	GO:0009536	76.00	48.91	34
IRC_IRT_c30233	156	XP_006393166.1	0.9	-	58.00	32.73	39
IRC_IRT_c30276	118	CDY33651.1	0.5	-	70.00	32.73	34
IRC_IRT_c30277	138	EEC83292.1	0.0	GO:0016021	82.00	69.71	46
IRC_IRT_c30289	115	KEH15665.1	0.3	-	63.00	33.50	38
IRC_IRT_c30291	138	XP_003078167.1	0.0	GO:0016874	71.00	53.53	45
IRC_IRT_c30309	105	XP_002535483.1	0.0	-	64.00	40.43	34
IRC_IRT_c30345	163	XP_010911665.1	0.0	GO:0009750	96.00	103.99	54
IRC_IRT_c30352	225	XP_002535524.1	0.0	-	58.00	36.58	56
IRC_IRT_c30356	156	XP_002539732.1	0.0	GO:0006355	88.00	80.88	51
IRC_IRT_c30358	146	EEE67870.1	0.0	GO:0009443	84.00	57.77	39
IRC_IRT_c30360	118	XP_009350073.1	0.0	-	73.00	40.43	38
IRC_IRT_c30392	142	KDD74604.1	0.0	GO:0048037	80.00	71.25	47
IRC_IRT_c30407	171	AAV31373.1	0.0	GO:0003676	100.00	83.96	37
IRC_IRT_c30451	132	BAD03640.1	0.0	-	86.00	67.78	43
IRC_IRT_c30496	174	KDD74062.1	0.0	GO:0051287	84.00	77.80	52
IRC_IRT_c30538	201	XP_002540560.1	0.0	-	60.00	35.81	40
IRC_IRT_c30580	104	CAE04656.2	0.0	-	97.00	71.25	34
IRC_IRT_c30598	154	XP_002536557.1	0.0	-	67.00	50.06	49
IRC_IRT_c30620	169	XP_001786775.1	0.0	GO:0005524	89.00	95.13	56

IRC_IRT_c30654	123	XP_001787120.1	0.0	GO:0005737	87.00	68.55	41
IRC_IRT_c30678	112	EEE64035.1	0.0	GO:0005739	70.00	44.67	37
IRC_IRT_c30681	129	KDD71429.1	0.0	GO:0008233	95.00	76.26	42
IRC_IRT_c30683	137	XP_002538039.1	0.0	GO:0003824	78.00	59.69	37
IRC_IRT_c30719	120	XP_001701551.1	0.0	-	77.00	46.98	35
IRC_IRT_c30747	112	EEC67125.1	0.0	GO:0030170	100.00	75.49	37
IRC_IRT_c30771	168	XP_008796093.1	0.0	GO:0006412	75.00	84.34	56
IRC_IRT_c30831	120	XP_011016720.1	0.0	-	70.00	39.28	40
IRC_IRT_c30835	115	XP_001770297.1	0.6	-	63.00	32.73	38
IRC_IRT_c30850	135	BAC82903.1	0.0	-	83.00	71.63	43
IRC_IRT_c30865	124	XP_004972985.1	0.8	-	55.00	32.34	40
IRC_IRT_c30871	155	AAR87260.1	0.0	GO:0003964	94.00	67.40	36
IRC_IRT_c30884	126	AAV59434.1	0.0	GO:0009536	100.00	95.13	42
IRC_IRT_c30974	134	XP_001696746.1	0.0	GO:0016491	69.00	53.53	46
IRC_IRT_c30993	112	XP_002535920.1	0.0	GO:0090599	75.00	52.76	37
IRC_IRT_c31022	130	KFM24683.1	0.0	GO:0050896	76.00	57.00	43
IRC_IRT_c31033	105	BAK02440.1	0.0	-	61.00	39.28	34
IRC_IRT_c31050	161	KFM29290.1	0.6	-	60.00	31.96	35
IRC_IRT_c31061	104	AAL67599.1	0.2	-	100.00	34.65	34
IRC_IRT_c31087	109	XP_001699037.1	0.0	-	80.00	56.61	36
IRC_IRT_c31122	118	EAY84926.1	0.0	GO:0008152	91.00	72.40	36
IRC_IRT_c31124	166	AFW70735.1	0.4	-	53.00	33.88	56
IRC_IRT_c31133	123	XP_002440457.1	0.0	GO:0006164	70.00	49.68	34
IRC_IRT_c31155	133	EEC71766.1	0.0	GO:0003964	100.00	87.04	43
IRC_IRT_c31162	142	KGN62150.1	0.4	-	42.00	33.88	35
IRC_IRT_c31198	111	KDD73232.1	0.0	GO:0005739	83.00	59.31	36
IRC_IRT_c31206	208	CAH66048.1	0.0	GO:0003964	78.00	56.61	41
IRC_IRT_c31221	196	XP_002448285.1	0.0	-	69.00	65.08	52
IRC_IRT_c31230	107	NP_001051386.1	0.0	GO:0009108	100.00	75.10	35
IRC_IRT_c31313	126	XP_002537624.1	0.0	GO:0046394	71.00	46.60	38
IRC_IRT_c31329	117	NP_001048474.1	0.0	GO:0030247	100.00	87.43	3.90E+01
IRC_IRT_c31408	122	AGC78953.1	0.0	GO:0055114	75.00	58.54	40
IRC_IRT_c31452	132	ADQ42104.1	0.0	GO:0009772	100.00	77.80	37

IRC_IRT_c31457	158	XP_003060819.1	0.0	GO:0044249	61.00	55.45	52
IRC_IRT_c31483	104	EEC77111.1	0.0	GO:0006935	79.00	43.51	34
IRC_IRT_c31494	145	XP_002302132.2	0.1	-	60.00	36.19	38
IRC_IRT_c31537	152	XP_002538780.1	0.0	GO:0015700	97.00	92.43	48
IRC_IRT_c31587	180	CAK22274.1	0.0	GO:0016462	79.00	70.09	58
IRC_IRT_c31618	159	CCO17254.1	0.0	-	68.00	44.28	47
IRC_IRT_c31640	108	XP_007134311.1	0.0	-	60.00	36.97	35
IRC_IRT_c31656	176	XP_002538319.1	0.0	GO:0000155	82.00	65.47	45
IRC_IRT_c31673	118	XP_002502938.1	0.3	-	59.00	33.50	37
IRC_IRT_c31720	125	KIZ06735.1	0.5	-	60.00	33.50	43
IRC_IRT_c31730	125	XP_002535858.1	0.0	-	87.00	64.31	41
IRC_IRT_c31820	115	XP_002535090.1	0.0	-	62.00	36.97	35
IRC_IRT_c31858	170	XP_001700950.1	0.0	GO:1903046	71.00	60.85	49
IRC_IRT_c31870	117	XP_002536045.1	0.3	-	55.00	33.11	38
IRC_IRT_c31872	176	XP_010055176.1	0.0	-	64.00	70.09	57
IRC_IRT_c31888	106	XP_002539099.1	0.8	-	64.00	30.80	34
IRC_IRT_c31903	118	XP_002536468.1	0.0	-	76.00	61.62	39
IRC_IRT_c31959	105	KIZ03200.1	0.0	GO:0008236	76.00	50.83	34
IRC_IRT_c31961	164	AAP54515.1	0.0	-	94.00	66.24	37
IRC_IRT_c31984	113	XP_005847629.1	0.3	-	52.00	33.50	36
IRC_IRT_c31985	126	AAX95114.1	0.0	GO:0004523	97.00	88.58	42
IRC_IRT_c31989	108	XP_002534501.1	0.0	-	72.00	39.28	37
IRC_IRT_c31998	169	XP_002539057.1	0.0	-	62.00	48.52	48
IRC_IRT_c32020	147	XP_002979453.1	0.9	-	60.00	32.34	48
IRC_IRT_c32051	107	EAZ31222.1	0.0	-	100.00	74.71	35
IRC_IRT_c32057	137	AAO27881.1	0.0	GO:0044763	61.00	45.05	42
IRC_IRT_c32068	120	CAE04584.2	0.0	GO:0004523	80.00	66.63	40
IRC_IRT_c32079	110	ABA94686.1	0.0	GO:0006468	97.00	46.21	35
IRC_IRT_c32084	155	BAD46573.1	0.0	-	97.00	71.25	34
IRC_IRT_c32087	118	AFB33036.1	0.0	GO:0005525	85.00	50.45	34
IRC_IRT_c32103	103	XP_002536789.1	0.0	-	88.00	52.37	34
IRC_IRT_c32107	174	AAM01090.1	0.0	GO:0006508	95.00	99.37	4.80E+01
IRC_IRT_c32115	285	XP_002981271.1	0.0	GO:0005488	62.00	96.67	96

IRC_IRT_c32119	160	EEC76878.1	0.0	GO:0015986	79.00	61.62	44
IRC_IRT_c32131	105	XP_002537029.1	0.0	GO:0005215	79.00	51.99	34
IRC_IRT_c32142	172	CAD40111.2	0.0	GO:0004523	91.00	78.57	45
IRC_IRT_c32189	133	XP_011016603.1	0.0	-	86.00	72.02	43
IRC_IRT_c32217	220	XP_011000116.1	0.0	GO:0009536	73.00	92.05	73
IRC_IRT_c32233	125	XP_011016126.1	0.0	GO:0003735	82.00	60.85	40
IRC_IRT_c32250	108	XP_009627401.1	0.0	-	91.00	58.92	34
IRC_IRT_c32255	183	ABA96534.1	0.0	GO:0004523	88.00	111.31	53
IRC_IRT_c32270	130	EAY77274.1	0.0	GO:0005509	100.00	88.58	43
IRC_IRT_c32277	116	XP_006600558.1	0.0	-	67.00	36.19	37
IRC_IRT_c32278	121	EEC76877.1	0.0	GO:0046961	79.00	55.45	39
IRC_IRT_c32286	103	EEC81226.1	0.0	GO:0016023	100.00	71.25	34
IRC_IRT_c32297	131	AFW63063.1	0.0	GO:0005737	100.00	88.20	43
IRC_IRT_c32313	124	CAE02543.1	0.0	GO:0003723	92.00	78.57	40
IRC_IRT_c32401	143	EEC76122.1	0.0	-	63.00	38.89	46
IRC_IRT_c32411	103	EEE68528.1	0.0	GO:0055085	97.00	60.08	34
IRC_IRT_c32432	126	NP_001044249.1	0.0	-	55.00	38.89	45
IRC_IRT_c32458	120	AAN11192.1	0.0	GO:0004523	97.00	84.34	39
IRC_IRT_c32490	110	XP_002536223.1	0.0	-	66.00	40.05	36
IRC_IRT_c32498	154	KFM23445.1	0.0	-	56.00	37.35	44
IRC_IRT_c32499	136	KEH15347.1	0.0	GO:0005488	69.00	43.90	42
IRC_IRT_c32507	167	XP_001786560.1	0.0	GO:0044763	69.00	52.76	53
IRC_IRT_c32515	128	ABA94864.1	0.0	-	46.00	42.74	67
IRC_IRT_c32526	157	ABA97233.1	0.0	GO:0004523	74.00	80.11	51
IRC_IRT_c32540	106	XP_003523224.1	0.0	GO:0016624	77.00	48.52	35
IRC_IRT_c32560	133	ABF95774.1	0.0	GO:0016311	100.00	91.66	44
IRC_IRT_c32587	115	XP_002540481.1	0.0	-	77.00	48.52	35
IRC_IRT_c32589	133	XP_009387920.1	0.1	-	62.00	34.65	35
IRC_IRT_c32666	120	EXB32139.1	0.0	GO:0008137	80.00	53.14	35
IRC_IRT_c32693	152	XP_006306291.1	0.0	GO:0005737	85.00	58.92	40
IRC_IRT_c32759	128	BAD53002.1	0.0	GO:0005739	100.00	65.86	34
IRC_IRT_c32770	140	EEC82409.1	0.0	GO:0016021	86.00	84.34	46
IRC_IRT_c32781	170	XP_005849236.1	0.0	-	74.00	58.54	51

IRC_IRT_c32811	106	XP_001786971.1	0.0	-	69.00	36.58	36
IRC_IRT_c32817	111	XP_003080624.1	0.0	GO:0044710	70.00	46.98	37
IRC_IRT_c32818	153	XP_002879833.1	0.0	GO:0006073	48.00	53.53	70
IRC_IRT_c32824	133	NP_001061942.1	0.0	GO:0046854	100.00	95.90	44
IRC_IRT_c32829	125	NP_001237810.1	0.1	-	60.00	34.65	35
IRC_IRT_c32848	108	NP_001057528.1	0.0	GO:0006096	100.00	74.33	34
IRC_IRT_c32856	123	BAD11580.1	0.0	-	67.00	40.05	40
IRC_IRT_c32858	123	BAD87985.1	0.0	-	74.00	45.44	35
IRC_IRT_c32922	111	EMT19896.1	0.3	-	57.00	33.50	35
IRC_IRT_c32949	124	NP_001060230.1	0.0	GO:0006355	100.00	88.20	41
IRC_IRT_c32956	173	EMT14173.1	0.6	-	44.00	33.50	58
IRC_IRT_c32966	103	XP_002455427.1	0.1	-	58.00	35.42	34
IRC_IRT_c33003	141	XP_002960029.1	0.0	GO:0003824	71.00	52.76	46
IRC_IRT_c33013	105	XP_003061391.1	0.0	-	62.00	37.74	37
IRC_IRT_c33021	122	XP_001762156.1	0.0	GO:0016740	72.00	52.37	40
IRC_IRT_c33058	136	EMT00231.1	0.0	GO:0006732	91.00	73.94	45
IRC_IRT_c33063	137	ABA93438.2	0.0	GO:0004523	93.00	77.80	44
IRC_IRT_c33069	157	EEC76877.1	0.0	GO:0046034	74.00	66.63	47
IRC_IRT_c33082	147	BAC10068.1	0.9	-	63.00	30.42	38
IRC_IRT_c33119	113	EEC81325.1	0.0	GO:0006810	78.00	48.91	37
IRC_IRT_c33169	113	KIY97004.1	0.0	GO:0031071	81.00	58.54	37
IRC_IRT_c33178	127	XP_002534653.1	0.0	-	63.00	37.35	38
IRC_IRT_c33191	133	XP_002534652.1	0.0	GO:0007165	70.00	42.36	37
IRC_IRT_c33202	117	AFW77096.1	0.0	GO:0006468	100.00	85.50	39
IRC_IRT_c33212	109	AGI15853.1	0.0	-	74.00	52.76	35
IRC_IRT_c33220	236	XP_007018153.1	0.0	GO:0055114	80.00	59.69	35
IRC_IRT_c33233	111	NP_001046848.1	0.0	-	69.00	50.83	36
IRC_IRT_c33236	300	AGC78943.1	0.0	-	63.00	45.44	55
IRC_IRT_c33259	169	BAD21713.1	0.0	GO:0003677	80.00	65.08	42
IRC_IRT_c33293	212	XP_003057676.1	0.0	GO:0006537	81.00	126.33	72
IRC_IRT_c33299	107	XP_002536847.1	0.0	-	67.00	37.35	34
IRC_IRT_c33308	122	KDD77116.1	0.0	-	67.00	36.58	37
IRC_IRT_c33324	145	XP_002536363.1	0.0	-	55.00	34.65	38

IRC_IRT_c33328	113	EEE62101.1	0.0	GO:0003677	100.00	82.03	37
IRC_IRT_c33334	120	XP_002537948.1	0.0	GO:0003700	91.00	63.54	37
IRC_IRT_c33370	143	BAD25387.1	0.0	-	100.00	93.20	4.40E+01
IRC_IRT_c33387	119	AFK44601.1	0.0	GO:0008152	74.00	50.83	39
IRC_IRT_c33398	152	EMT12954.1	0.8	-	50.00	32.34	36
IRC_IRT_c33424	207	KEH15576.1	0.0	-	71.00	45.05	38
IRC_IRT_c33550	228	CDY30864.1	0.0	-	64.00	39.28	34
IRC_IRT_c33586	163	XP_005646018.1	0.0	GO:0008152	67.00	43.13	43
IRC_IRT_c33593	156	CDY63598.1	0.0	-	59.00	45.44	47
IRC_IRT_c33618	115	EEC75667.1	0.0	GO:0008854	79.00	54.30	34
IRC_IRT_c33623	122	XP_010045476.1	0.6	-	52.00	32.34	34
IRC_IRT_c33628	123	EPS69268.1	0.7	-	64.00	32.73	39
IRC_IRT_c33646	147	EEC69010.1	0.1	-	58.00	33.50	46
IRC_IRT_c33647	167	XP_002538039.1	0.0	GO:0008658	79.00	70.86	49
IRC_IRT_c33682	146	ABA96533.1	0.0	GO:0090502	92.00	79.34	41
IRC_IRT_c33721	127	XP_005842572.1	0.2	-	52.00	32.34	38
IRC_IRT_c33740	104	XP_002540420.1	0.0	-	61.00	38.89	34
IRC_IRT_c33746	141	XP_002983167.1	0.0	GO:0003676	85.00	83.57	47
IRC_IRT_c33827	152	XP_006401630.1	0.0	GO:0044710	67.00	40.43	34
IRC_IRT_c33852	128	BAJ91362.1	0.0	GO:0008152	65.00	40.82	41
IRC_IRT_c33865	144	EEC82857.1	0.0	GO:0016024	100.00	98.21	47
IRC_IRT_c33876	152	ABA98392.1	0.0	GO:0003676	93.00	79.34	45
IRC_IRT_c33921	124	XP_002537026.1	0.0	GO:0000155	81.00	50.83	38
IRC_IRT_c33970	176	XP_011091392.1	0.0	GO:0009506	58.00	45.82	55
IRC_IRT_c33994	122	NP_001062069.1	0.0	-	100.00	73.17	34
IRC_IRT_c34003	106	XP_002280899.1	0.9	-	65.00	32.34	35
IRC_IRT_c34007	108	CAE01996.2	0.0	-	70.00	56.23	34
IRC_IRT_c34045	105	EEC68764.1	0.0	-	100.00	70.86	34
IRC_IRT_c34057	103	BAD20179.1	0.0	-	73.00	38.51	34
IRC_IRT_c34089	129	CDO97424.1	0.1	-	65.00	35.81	41
IRC_IRT_c34128	122	ABB47015.1	0.0	GO:0016021	91.00	64.70	34
IRC_IRT_c34147	195	BAD38496.1	0.0	GO:0005739	80.00	58.54	36
IRC_IRT_c34231	142	EEE70252.1	0.0	GO:0016597	100.00	96.29	46

IRC_IRT_c34326	109	XP_002537329.1	0.0	-	80.00	42.36	36
IRC_IRT_c34386	117	XP_008785479.1	0.2	-	61.00	34.65	42
IRC_IRT_c34391	104	CAD39396.2	0.0	GO:0004523	100.00	75.49	34
IRC_IRT_c34460	163	YP_008802541.1	0.0	GO:0016021	84.00	61.23	46
IRC_IRT_c34467	155	XP_002535357.1	0.1	-	61.00	35.42	44
IRC_IRT_c34494	130	CBI31106.3	0.0	GO:0051287	95.00	80.88	40
IRC_IRT_c34543	130	KDP21323.1	0.0	-	70.00	38.89	44
IRC_IRT_c34546	127	EAY84569.1	0.0	GO:0050660	90.00	77.41	41
IRC_IRT_c34553	183	XP_002501367.1	0.7	-	46.00	33.50	54
IRC_IRT_c34562	105	XP_003080833.1	0.1	-	67.00	35.42	34
IRC_IRT_c34589	124	XP_005650157.1	0.8	-	55.00	32.73	40
IRC_IRT_c34643	113	KDD74521.1	0.0	-	73.00	43.51	34
IRC_IRT_c34680	119	XP_002539630.1	0.0	GO:0005975	76.00	47.75	38
IRC_IRT_c34695	133	XP_007223491.1	0.1	-	63.00	34.65	44
IRC_IRT_c34757	145	XP_002537249.1	0.0	-	88.00	51.22	43
IRC_IRT_c34761	153	XP_007048418.1	0.3	-	54.00	34.27	37
IRC_IRT_c34770	111	XP_002538377.1	0.0	-	72.00	41.97	36
IRC_IRT_c34774	209	XP_002537322.1	0.0	GO:0046872	68.00	73.17	67
IRC_IRT_c34789	129	XP_009619491.1	0.0	GO:0003824	76.00	48.52	38
IRC_IRT_c34790	167	BAD36485.1	0.0	GO:0009536	72.00	57.00	43
IRC_IRT_c34798	103	CAC43287.1	0.7	-	64.00	32.34	34
IRC_IRT_c34863	159	XP_008392011.1	0.0	GO:0043231	81.00	74.33	49
IRC_IRT_c34873	130	XP_002540432.1	0.0	-	73.00	60.08	41
IRC_IRT_c34920	135	NP_001176614.1	0.4	-	54.00	33.50	35
IRC_IRT_c34924	148	XP_002535764.1	0.0	GO:0035556	71.00	56.23	49
IRC_IRT_c34936	156	EAZ22525.1	0.0	GO:0005576	87.00	92.05	48
IRC_IRT_c34948	110	XP_002534926.1	0.0	-	94.00	72.02	36
IRC_IRT_c34960	130	AAO72613.1	0.0	GO:0015833	100.00	50.45	38
IRC_IRT_c34961	171	XP_006647306.1	0.3	-	56.00	34.27	37
IRC_IRT_c34973	119	EAZ43720.1	0.0	GO:0006810	80.00	48.52	35
IRC_IRT_c34988	122	XP_009780312.1	0.0	GO:0008233	100.00	74.33	40
IRC_IRT_c35004	166	BAD29510.1	0.0	-	73.00	50.83	41
IRC_IRT_c35007	207	CAE03484.2	0.0	GO:0003676	96.00	110.15	53

IRC_IRT_c35010	113	BAD37594.1	0.0	-	81.00	46.98	37
IRC_IRT_c35021	110	EEC78702.1	0.0	-	62.00	37.74	35
IRC_IRT_c35035	181	XP_009631217.1	0.5	-	60.00	33.11	56
IRC_IRT_c35054	102	EAY96497.1	0.0	GO:0016491	100.00	72.79	34
IRC_IRT_c35078	157	XP_003525588.1	0.0	-	64.00	40.82	42
IRC_IRT_c35135	106	ACA42756.1	0.0	-	62.00	43.90	35
IRC_IRT_c35153	125	XP_002975758.1	0.0	GO:0005737	66.00	49.29	39
IRC_IRT_c35201	131	AAL75760.1	0.0	GO:0016568	100.00	87.81	43
IRC_IRT_c35240	120	XP_002534842.1	0.4	-	68.00	33.50	35
IRC_IRT_c35241	110	XP_005849524.1	0.0	GO:0031348	88.00	58.15	36
IRC_IRT_c35245	113	ABA98107.1	0.0	GO:0004523	97.00	51.22	34
IRC_IRT_c35248	119	AAF69810.1	0.0	-	79.00	49.29	34
IRC_IRT_c35290	145	XP_005643737.1	0.0	GO:0050897	80.00	62.77	45
IRC_IRT_c35311	145	XP_002538733.1	0.0	-	71.00	35.81	35
IRC_IRT_c35323	117	XP_002950060.1	0.0	-	67.00	38.12	43
IRC_IRT_c35335	130	AAP53981.1	0.0	GO:0005739	84.00	60.85	39
IRC_IRT_c35388	173	CBI17411.3	1.0	-	58.00	32.73	50
IRC_IRT_c35389	133	XP_002534940.1	0.0	GO:0004151	81.00	69.32	43
IRC_IRT_c35422	139	KEH15547.1	0.1	-	56.00	35.81	46
IRC_IRT_c35436	213	XP_011016125.1	0.0	GO:0016779	92.00	106.30	57
IRC_IRT_c35446	167	ABR25518.1	0.0	GO:0005840	100.00	80.11	55
IRC_IRT_c35465	124	KDP21062.1	0.8	-	57.00	30.80	40
IRC_IRT_c35482	111	XP_006659773.1	0.0	-	79.00	52.37	34
IRC_IRT_c35499	224	XP_003638717.1	0.4	-	61.00	34.27	34
IRC_IRT_c35504	136	XP_002962638.1	0.4	-	63.00	33.88	44
IRC_IRT_c35508	155	AIA92249.1	0.0	GO:0003676	70.00	59.69	51
IRC_IRT_c35534	141	AAN65033.1	0.0	GO:0004523	93.00	81.26	43
IRC_IRT_c35538	224	EEC68421.1	0.0	GO:0009536	51.00	50.45	74
IRC_IRT_c35583	117	XP_001766190.1	0.5	-	61.00	32.73	39
IRC_IRT_c35584	106	XP_002535020.1	0.0	GO:0008999	79.00	44.67	34
IRC_IRT_c35652	134	XP_006289129.1	0.4	-	48.00	32.73	41
IRC_IRT_c35656	115	XP_003064794.1	0.0	GO:0016491	71.00	43.90	35
IRC_IRT_c35657	106	EAZ01685.1	0.0	GO:0009220	100.00	70.48	3.50E+01

IRC_IRT_c35661	118	XP_002538976.1	0.0	-	63.00	36.58	36
IRC_IRT_c35694	103	XP_002538133.1	0.0	-	73.00	40.43	34
IRC_IRT_c35710	141	XP_001786539.1	0.8	-	52.00	32.73	38
IRC_IRT_c35738	135	XP_001754074.1	0.0	-	63.00	41.20	41
IRC_IRT_c35798	106	CDP15087.1	0.0	GO:0009941	82.00	45.05	35
IRC_IRT_c35828	142	KIZ07052.1	0.0	GO:0044249	71.00	54.68	46
IRC_IRT_c35831	155	NP_001055378.2	0.7	-	53.00	32.73	43
IRC_IRT_c35877	106	XP_011039577.1	0.1	-	76.00	34.65	34
IRC_IRT_c35878	124	XP_003601698.1	0.0	GO:0003824	70.00	45.05	41
IRC_IRT_c35908	134	KEH15234.1	0.0	GO:0006096	80.00	60.85	42
IRC_IRT_c35979	154	XP_003539582.1	0.6	-	58.00	33.50	36
IRC_IRT_c35999	219	YP_001152215.1	0.0	-	62.00	37.35	35
IRC_IRT_c36017	145	XP_001786976.1	0.5	-	59.00	33.11	37
IRC_IRT_c36035	157	EMT17544.1	0.0	-	60.00	38.51	38
IRC_IRT_c36080	203	XP_002537961.1	0.0	-	58.00	41.97	62
IRC_IRT_c36084	187	AAX93001.1	0.0	GO:0003676	98.00	120.55	55
IRC_IRT_c36158	126	KEH15131.1	0.0	-	54.00	36.97	42
IRC_IRT_c36176	103	ACN27968.1	0.3	-	76.00	33.11	34
IRC_IRT_c36207	131	EAZ02962.1	0.0	-	72.00	48.14	36
IRC_IRT_c36225	124	XP_010233718.1	0.2	-	69.00	34.27	43
IRC_IRT_c36263	118	KEH15984.1	0.0	-	71.00	45.44	35
IRC_IRT_c36266	164	XP_010428906.1	0.0	-	64.00	38.51	39
IRC_IRT_c36298	136	BAJ21551.1	0.0	GO:0055114	68.00	49.29	45
IRC_IRT_c36299	209	ABA99150.1	0.0	GO:0003964	98.00	140.20	69
IRC_IRT_c36369	176	CDX88867.1	0.6	-	52.00	33.50	50
IRC_IRT_c36381	116	AAB80804.1	0.3	-	56.00	33.50	44
IRC_IRT_c36494	143	EMT15229.1	0.2	-	61.00	34.27	39
IRC_IRT_c36531	137	A2YMR0.1	0.0	-	100.00	39.66	45
IRC_IRT_c36549	104	EEC82514.1	0.0	GO:0016023	94.00	67.78	34
IRC_IRT_c36596	138	XP_006853393.1	0.1	-	56.00	35.81	44
IRC_IRT_c36688	109	AAF87897.1	0.3	-	61.00	33.50	36
IRC_IRT_c36742	131	AHH24342.1	0.0	GO:0098655	79.00	47.37	34
IRC_IRT_c36765	206	XP_010911304.1	0.0	GO:0006935	78.00	30.80	38

IRC_IRT_c36783	105	XP_001786556.1	0.0	GO:0004129	85.00	50.45	35
IRC_IRT_c36787	104	XP_002535741.1	0.0	GO:0006935	88.00	46.21	34
IRC_IRT_c36791	204	XP_002537339.1	0.0	-	80.00	91.28	66
IRC_IRT_c36800	131	ABB47187.2	0.0	GO:0090502	97.00	98.60	43
IRC_IRT_c36849	203	XP_004134959.1	0.2	-	48.00	35.04	39
IRC_IRT_c36863	128	XP_002537087.1	0.0	GO:0046872	88.00	73.17	42
IRC_IRT_c36881	179	XP_002537142.1	0.0	-	79.00	69.71	48
IRC_IRT_c36885	113	XP_010054804.1	0.5	-	62.00	33.11	35
IRC_IRT_c36891	106	XP_002534940.1	0.0	GO:0004151	85.00	57.00	35
IRC_IRT_c36905	113	CEF99717.1	0.0	-	81.00	38.51	37
IRC_IRT_c36961	152	BAD28311.1	0.0	-	94.00	65.86	50
IRC_IRT_c36970	152	YP_006666408.1	0.0	GO:0005488	66.00	53.53	50
IRC_IRT_c37026	103	AAD01679.1	0.1	-	61.00	34.27	34
IRC_IRT_c37029	135	XP_002505482.1	0.0	GO:0005488	74.00	50.45	43
IRC_IRT_c37055	163	XP_006286967.1	0.6	-	57.00	33.50	35
IRC_IRT_c37090	109	CAD29284.1	0.0	GO:0006655	100.00	76.26	35
IRC_IRT_c37107	147	XP_008437000.1	0.0	-	57.00	36.58	42
IRC_IRT_c37132	118	EDQ48485.1	0.0	-	71.00	43.13	39
IRC_IRT_c37140	106	ABA96923.1	0.0	GO:0015078	100.00	69.32	34
IRC_IRT_c37168	130	NP_001064098.1	0.0	GO:0003677	100.00	92.43	43
IRC_IRT_c37188	191	XP_002536183.1	0.0	GO:0044763	62.00	56.61	58
IRC_IRT_c37190	107	CEF99124.1	0.0	GO:0016787	77.00	46.60	35
IRC_IRT_c37371	127	XP_002952066.1	0.0	GO:0016556	80.00	51.99	36
IRC_IRT_c37419	108	XP_008358614.1	0.0	-	68.00	38.12	35
IRC_IRT_c37441	141	XP_011014252.1	0.0	-	55.00	40.43	45
IRC_IRT_c37479	152	EEC79491.1	0.0	GO:0090304	74.00	52.76	35
IRC_IRT_c37520	253	EAY99043.1	0.0	GO:0043565	79.00	114.39	86
IRC_IRT_c37532	155	XP_010269090.1	0.8	-	52.00	32.73	34
IRC_IRT_c37544	170	XP_005648464.1	0.0	GO:0016740	72.00	51.22	48
IRC_IRT_c37579	150	EEE68528.1	0.0	GO:0055085	88.00	70.48	44
IRC_IRT_c37598	114	XP_002535442.1	0.0	GO:0030254	89.00	72.40	38
IRC_IRT_c37643	165	XP_001422695.1	0.0	GO:0006260	77.00	43.51	35
IRC_IRT_c37673	151	BAC83432.1	0.5	-	46.00	32.34	45

IRC_IRT_c37691	122	XP_003082104.1	0.3	-	56.00	33.50	39
IRC_IRT_c37741	129	XP_009387331.1	0.0	-	64.00	36.58	34
IRC_IRT_c37767	188	BAD09754.1	0.0	GO:0016023	84.00	70.48	38
IRC_IRT_c37768	107	XP_010029395.1	0.0	GO:0055085	85.00	59.69	35
IRC_IRT_c37887	131	XP_001703326.1	0.0	-	71.00	42.74	35
IRC_IRT_c37933	130	XP_002540420.1	0.0	-	86.00	69.71	43
IRC_IRT_c37952	183	XP_009631450.1	0.0	GO:0016740	73.00	47.75	42
IRC_IRT_c37977	115	XP_008378650.1	0.0	GO:0006259	79.00	44.67	34
IRC_IRT_c37995	225	BAJ94909.1	0.0	GO:0043231	65.00	88.20	78
IRC_IRT_c38033	144	XP_009386971.1	0.0	-	58.00	43.51	48
IRC_IRT_c38035	175	XP_002537105.1	0.0	-	53.00	46.60	56
IRC_IRT_c38040	103	XP_009350817.1	0.0	-	76.00	41.59	34
IRC_IRT_c38060	125	BAD88193.1	0.0	GO:0003677	94.00	65.86	35
IRC_IRT_c38068	104	AFK10053.1	0.0	GO:0003899	85.00	62.39	34
IRC_IRT_c38073	103	XP_009350806.1	0.0	GO:0030288	91.00	58.15	34
IRC_IRT_c38074	103	XP_011016696.1	0.0	GO:0008233	91.00	61.62	34
IRC_IRT_c38084	148	BAD16964.1	0.0	-	97.00	58.54	49
IRC_IRT_c38120	116	XP_008236452.1	0.3	-	55.00	33.88	34
IRC_IRT_c38123	107	XP_001786560.1	0.0	GO:0006289	88.00	54.68	35
IRC_IRT_c38132	129	NP_039379.1	0.0	GO:0046933	100.00	76.26	39
IRC_IRT_c38149	129	XP_002535303.1	0.0	GO:0006810	82.00	55.07	41
IRC_IRT_c38156	115	AAM01000.1	0.0	GO:0006508	100.00	80.49	38
IRC_IRT_c38163	109	CDY69935.1	0.0	-	66.00	36.97	36
IRC_IRT_c38213	106	XP_002536585.1	0.0	-	76.00	45.05	34
IRC_IRT_c38218	106	CAE03363.1	0.0	-	100.00	72.40	35
IRC_IRT_c38231	116	XP_005647217.1	0.1	-	65.00	35.81	38
IRC_IRT_c38246	118	NP_001049943.1	0.0	GO:0016023	94.00	65.86	3.40E+01
IRC_IRT_c38261	145	XP_001416495.1	0.0	-	60.00	38.51	43
IRC_IRT_c38266	151	ABA98373.1	0.0	GO:0003964	100.00	92.82	43
IRC_IRT_c38275	110	EEC76122.1	0.0	GO:0044763	73.00	44.28	34
IRC_IRT_c38321	120	XP_004289305.2	0.0	-	63.00	36.97	41
IRC_IRT_c38322	111	XP_002537826.1	0.0	GO:0008152	74.00	41.97	35
IRC_IRT_c38331	129	AAQ56293.1	0.0	GO:0090502	100.00	95.52	42

IRC_IRT_c38334	196	AFK42539.1	0.0	GO:0044272	67.00	64.31	64
IRC_IRT_c38437	135	KFK39251.1	0.4	-	56.00	33.50	37
IRC_IRT_c38438	107	ABF97159.1	0.0	GO:0004523	97.00	76.64	35
IRC_IRT_c38446	102	BAD73087.1	0.0	-	63.00	38.89	38
IRC_IRT_c38453	174	XP_002538830.1	0.1	-	63.00	35.04	38
IRC_IRT_c38459	161	XP_008340816.1	0.9	-	59.00	32.73	42
IRC_IRT_c38474	114	EAY81794.1	0.0	-	100.00	78.18	38
IRC_IRT_c38485	117	XP_003061503.1	0.0	GO:0005739	77.00	49.29	36
IRC_IRT_c38534	124	XP_002535428.1	0.0	GO:1902358	88.00	59.69	35
IRC_IRT_c38547	207	EPS70027.1	0.0	-	55.00	48.14	52
IRC_IRT_c38548	272	EPS74531.1	0.0	-	68.00	47.37	44
IRC_IRT_c38551	105	EMS65074.1	0.0	GO:0006810	76.00	41.97	34
IRC_IRT_c38556	146	XP_001786560.1	0.0	GO:0006289	80.00	60.85	46
IRC_IRT_c38561	169	XP_009350809.1	0.0	-	75.00	40.82	36
IRC_IRT_c38590	148	XP_002538398.1	0.0	GO:0007165	70.00	48.91	48
IRC_IRT_c38606	163	XP_002536446.1	0.0	GO:0030554	89.00	61.62	47
IRC_IRT_c38610	131	XP_009108342.1	0.8	-	55.00	32.73	38
IRC_IRT_c38614	117	XP_003617621.1	0.0	-	64.00	40.82	37
IRC_IRT_c38622	239	XP_003599577.1	0.0	-	71.00	40.05	35
IRC_IRT_c38630	175	XP_009388207.1	0.2	-	47.00	34.27	40
IRC_IRT_c38697	114	XP_002980311.1	0.0	GO:0044249	73.00	47.37	38
IRC_IRT_c38721	130	XP_006296419.1	0.2	-	68.00	34.65	35
IRC_IRT_c38728	122	XP_005650201.1	0.0	-	75.00	52.37	40
IRC_IRT_c38741	163	ABB47363.1	0.0	GO:0016023	97.00	80.88	40
IRC_IRT_c38824	161	XP_009116558.1	0.1	-	53.00	36.19	52
IRC_IRT_c38837	103	NP_001053168.2	0.0	GO:0016021	100.00	79.34	3.40E+01
IRC_IRT_c38864	109	XP_004486904.1	0.0	GO:0004174	86.00	55.45	36
IRC_IRT_c38892	137	KEH42723.1	0.7	-	56.00	32.73	41
IRC_IRT_c38909	121	XP_002535267.1	0.0	-	58.00	39.28	39
IRC_IRT_c38951	135	BAD22424.1	0.0	GO:0046872	95.00	73.94	40
IRC_IRT_c38966	109	KDD75048.1	0.8	-	58.00	32.34	34
IRC_IRT_c38986	103	AAZ79358.1	0.0	GO:0004029	79.00	53.14	34
IRC_IRT_c39025	111	ACN35500.1	0.0	GO:0016747	74.00	40.82	35

IRC_IRT_c39057	114	XP_009416163.1	0.0	-	64.00	41.59	34
IRC_IRT_c39074	116	ABA97227.2	0.0	-	65.00	41.59	40
IRC_IRT_c39161	122	XP_002968991.1	0.0	-	68.00	50.83	44
IRC_IRT_c39198	112	EEE52320.1	0.0	-	58.00	38.51	39
IRC_IRT_c39210	121	XP_004486300.1	0.8	-	60.00	32.34	35
IRC_IRT_c39238	271	YP_358636.1	0.0	GO:0009536	72.00	50.06	37
IRC_IRT_c39248	166	XP_004293279.1	0.0	GO:0016226	90.00	105.53	55
IRC_IRT_c39261	175	CDP11921.1	0.6	-	64.00	33.50	45
IRC_IRT_c39272	218	BAJ11784.1	0.0	-	66.00	43.90	39
IRC_IRT_c39277	127	XP_004235396.1	0.3	-	52.00	33.50	40
IRC_IRT_c39290	204	KCW80241.1	0.5	-	45.00	33.88	53
IRC_IRT_c39303	106	XP_003635966.1	0.0	GO:0003824	77.00	46.21	35
IRC_IRT_c39332	135	XP_002536202.1	0.0	-	67.00	38.12	40
IRC_IRT_c39343	116	NP_001174680.1	0.0	-	100.00	78.95	36
IRC_IRT_c39351	104	XP_003611421.1	0.0	GO:0008236	76.00	41.97	34
IRC_IRT_c39356	179	BAD54716.1	0.0	-	76.00	38.12	34
IRC_IRT_c39387	122	XP_003063388.1	0.5	-	57.00	33.11	38
IRC_IRT_c39391	117	XP_003057940.1	0.0	GO:0005488	66.00	45.05	39
IRC_IRT_c39441	228	XP_002953179.1	0.0	-	55.00	40.43	45
IRC_IRT_c39450	132	XP_008666057.1	0.4	-	60.00	33.50	40
IRC_IRT_c39460	106	XP_006427807.1	0.0	GO:0045893	88.00	63.16	34
IRC_IRT_c39463	107	XP_002537069.1	0.0	-	74.00	43.13	35
IRC_IRT_c39468	123	EEC78535.1	0.0	GO:0008152	100.00	80.88	40
IRC_IRT_c39545	104	KFM27378.1	0.0	-	76.00	58.15	34
IRC_IRT_c39546	128	XP_003062118.1	0.9	-	45.00	32.34	48
IRC_IRT_c39553	151	XP_002537492.1	0.0	GO:0008152	67.00	46.21	46
IRC_IRT_c39575	128	AAM00947.1	0.6	-	70.00	32.34	34
IRC_IRT_c39597	104	XP_002488912.1	0.0	-	67.00	36.97	34
IRC_IRT_c39675	117	AFQ02691.1	0.0	-	88.00	62.00	35
IRC_IRT_c39764	116	XP_006646324.1	0.0	GO:0010413	100.00	82.80	38
IRC_IRT_c39768	114	XP_002537024.1	0.0	GO:0016788	77.00	55.07	36
IRC_IRT_c39817	140	NP_001047097.1	0.0	GO:0072488	100.00	92.82	43
IRC_IRT_c39821	119	XP_005851230.1	0.0	-	65.00	35.42	35

IRC_IRT_c39879	146	XP_004515575.1	0.1	-	54.00	35.42	44
IRC_IRT_c39896	151	CAH67683.1	0.0	GO:0004523	91.00	78.18	45
IRC_IRT_c39916	129	NP_001152446.1	0.4	-	51.00	33.50	43
IRC_IRT_c39923	152	KIY92252.1	0.0	-	78.00	68.55	47
IRC_IRT_c39974	108	NP_001058958.1	0.0	GO:0010207	97.00	71.63	35
IRC_IRT_c39983	123	XP_006450126.1	0.0	-	62.00	38.89	40
IRC_IRT_c40025	254	CAD79706.2	0.0	GO:0003676	100.00	135.19	62
IRC_IRT_c40070	117	XP_002534909.1	0.0	-	76.00	40.82	39
IRC_IRT_c40108	122	XP_001756720.1	0.1	-	60.00	35.42	38
IRC_IRT_c40113	115	XP_009350056.1	0.0	-	89.00	62.77	37
IRC_IRT_c40221	144	BAD11616.1	0.0	-	94.00	67.01	35
IRC_IRT_c40234	108	EAY99945.1	0.0	GO:0016772	71.00	43.13	35
IRC_IRT_c40235	130	BAD72793.1	0.0	-	52.00	36.58	50
IRC_IRT_c40256	105	CAE05604.2	0.0	-	100.00	71.25	34
IRC_IRT_c40265	140	XP_002535367.1	0.0	-	56.00	36.97	39
IRC_IRT_c40304	105	EEE54182.1	0.0	GO:0007049	100.00	76.26	35
IRC_IRT_c40348	102	XP_005649298.1	0.0	GO:0015238	88.00	68.17	34
IRC_IRT_c40371	131	XP_009378998.1	0.0	GO:0055114	82.00	48.52	34
IRC_IRT_c40422	126	KIZ00647.1	0.0	-	66.00	38.89	42
IRC_IRT_c40437	118	NP_001066271.1	0.0	GO:0045893	100.00	50.06	39
IRC_IRT_c40442	127	KEH15384.1	0.0	-	54.00	41.20	42
IRC_IRT_c40489	149	AAM12321.1	0.0	GO:0046961	100.00	101.29	49
IRC_IRT_c40513	123	AAP53837.2	0.0	GO:0003676	100.00	55.07	40
IRC_IRT_c40544	153	XP_002540005.1	0.0	-	69.00	60.46	46
IRC_IRT_c40576	124	KIZ01828.1	0.0	GO:0009446	73.00	54.68	41
IRC_IRT_c40692	105	NP_001055998.1	0.0	GO:0016301	91.00	64.31	34
IRC_IRT_c40716	109	2Z6C	0.1	-	61.00	33.88	36
IRC_IRT_c40777	102	EEE66471.1	0.0	GO:0017148	100.00	70.48	34
IRC_IRT_c40786	158	XP_003058549.1	0.0	GO:0044763	66.00	52.76	48
IRC_IRT_c40817	105	XP_008779577.1	0.0	GO:0004252	85.00	50.45	34
IRC_IRT_c40827	110	NP_001051458.1	0.1	-	100.00	33.50	36
IRC_IRT_c40870	160	KFK32966.1	0.0	GO:0006457	87.00	82.03	56
IRC_IRT_c40873	108	XP_003608394.1	0.1	-	64.00	34.27	34

IRC_IRT_c40885	108	AAP20850.1	0.1	-	100.00	35.04	34
IRC_IRT_c40887	110	XP_002540284.1	0.0	-	74.00	48.91	35
IRC_IRT_c40898	113	EAY87976.1	0.0	-	71.00	38.12	35
IRC_IRT_c40902	111	XP_002504924.1	0.0	GO:0003824	74.00	45.05	35
IRC_IRT_c40919	107	XP_002538208.1	0.2	-	67.00	33.11	34
IRC_IRT_c40931	122	XP_006604741.1	0.0	GO:0009536	76.00	45.82	34
IRC_IRT_c40932	108	XP_002538058.1	0.0	GO:0044699	71.00	41.97	35
IRC_IRT_c40953	119	XP_002535732.1	0.0	GO:0016021	97.00	80.49	39
IRC_IRT_c40980	135	NP_001062398.1	0.0	GO:0043531	100.00	94.36	44
IRC_IRT_c41002	119	XP_006295711.1	0.0	GO:0008152	82.00	66.63	39
IRC_IRT_c41025	126	BAE98219.1	0.1	-	52.00	33.88	36
IRC_IRT_c41032	146	AAL58146.1	0.0	-	72.00	42.74	40
IRC_IRT_c41039	162	AAK50607.1	0.0	GO:0044260	74.00	54.68	39
IRC_IRT_c41079	107	XP_008789524.1	0.0	GO:0005267	82.00	59.31	35
IRC_IRT_c41081	113	XP_009350812.1	0.0	-	62.00	36.19	40
IRC_IRT_c41136	164	XP_005643467.1	0.0	GO:0004832	79.00	79.72	49
IRC_IRT_c41153	116	XP_002540423.1	0.0	-	66.00	36.19	36
IRC_IRT_c41170	109	XP_002961324.1	0.0	-	64.00	37.35	34
IRC_IRT_c41195	108	XP_002537683.1	0.0	-	69.00	37.74	36
IRC_IRT_c41214	159	XP_009628962.1	0.4	-	57.00	33.88	56
IRC_IRT_c41263	130	XP_002536219.1	0.0	GO:0006935	93.00	65.47	43
IRC_IRT_c41268	151	XP_001751565.1	0.0	GO:0051287	76.00	60.85	47
IRC_IRT_c41299	142	XP_004253364.1	0.0	-	82.00	50.83	34
IRC_IRT_c41305	127	XP_011014727.1	0.0	-	82.00	53.91	41
IRC_IRT_c41316	136	XP_002501910.1	0.1	-	56.00	35.42	44
IRC_IRT_c41394	149	EEC70479.1	0.0	GO:0005524	80.00	69.32	46
IRC_IRT_c41400	126	XP_002537142.1	0.0	-	79.00	57.38	39
IRC_IRT_c41409	115	XP_004957009.1	0.0	-	80.00	44.28	35
IRC_IRT_c41435	138	XP_002536590.1	0.0	GO:0005975	89.00	73.56	38
IRC_IRT_c41474	135	AAT85124.1	0.0	GO:0005840	88.00	83.57	43
IRC_IRT_c41521	144	BAA84461.1	0.0	-	97.00	98.98	47
IRC_IRT_c41538	111	KFM28773.1	0.0	GO:0016462	77.00	51.22	35
IRC_IRT_c41565	145	ABF99143.1	0.0	GO:0090502	82.00	81.65	47

IRC_IRT_c41583	170	CCO15896.1	0.0	-	71.00	43.13	39
IRC_IRT_c41588	107	KIY92422.1	0.0	-	71.00	42.74	35
IRC_IRT_c41596	140	XP_009350067.1	0.0	-	66.00	40.05	36
IRC_IRT_c41665	149	KEH15345.1	0.0	GO:0016787	68.00	51.99	47
IRC_IRT_c41711	116	XP_010058038.1	0.1	-	67.00	35.81	34
IRC_IRT_c41731	111	XP_005646855.1	0.0	-	62.00	38.89	35
IRC_IRT_c41764	109	XP_008785289.1	0.0	GO:0005840	100.00	76.26	36
IRC_IRT_c41786	137	XP_010235017.1	0.4	-	58.00	33.50	34
IRC_IRT_c41830	106	XP_002535478.1	0.0	GO:0004519	77.00	57.00	35
IRC_IRT_c41833	133	ACU19360.1	0.0	GO:0008152	71.00	45.44	38
IRC_IRT_c41881	120	KFM27789.1	0.0	GO:0016866	76.00	46.21	34
IRC_IRT_c41906	133	ABA94864.1	0.0	GO:0009536	69.00	57.38	43
IRC_IRT_c41931	119	ERN17860.1	0.4	-	59.00	31.19	37
IRC_IRT_c41978	116	EAY73221.1	0.0	GO:0003774	81.00	49.68	38
IRC_IRT_c41980	107	AAM74298.1	0.0	GO:0003964	97.00	72.02	35
IRC_IRT_c41997	170	AAQ54877.1	0.0	GO:0000166	76.00	48.52	42
IRC_IRT_c42045	174	XP_009787798.1	0.9	-	52.00	33.11	34
IRC_IRT_c42074	130	XP_002535226.1	0.0	GO:0004316	82.00	51.22	40
IRC_IRT_c42103	111	NP_001041740.2	0.0	GO:0016021	100.00	82.80	37
IRC_IRT_c42114	104	KFM25591.1	0.0	-	67.00	36.19	34
IRC_IRT_c42191	112	XP_002500338.1	0.3	-	62.00	33.50	35
IRC_IRT_c42203	145	XP_011626213.1	0.0	-	59.00	40.05	47
IRC_IRT_c42218	157	XP_002536013.1	0.1	-	67.00	35.42	34
IRC_IRT_c42220	119	EYU35594.1	0.0	GO:0003824	75.00	50.83	37
IRC_IRT_c42281	204	XP_001786560.1	0.0	GO:0006289	81.00	67.78	53
IRC_IRT_c42292	110	NP_001044189.2	0.0	GO:0006629	97.00	73.94	36
IRC_IRT_c42315	110	XP_002537746.1	0.0	GO:0048831	94.00	64.70	36
IRC_IRT_c42321	112	EEC80356.1	0.0	GO:0016747	100.00	81.65	37
IRC_IRT_c42342	217	ABA97545.1	0.0	GO:0004523	100.00	126.33	65
IRC_IRT_c42347	155	XP_002444321.1	0.0	GO:0055085	88.00	57.38	35
IRC_IRT_c42369	160	BAJ90860.1	0.0	-	61.00	41.97	42
IRC_IRT_c42387	111	EEC69718.1	0.0	-	100.00	54.30	36
IRC_IRT_c42402	136	NP_001174657.1	0.0	-	89.00	72.79	38

IRC_IRT_c42474	118	EEC81261.1	0.0	GO:0016021	92.00	72.40	39
IRC_IRT_c42483	135	XP_002535112.1	0.8	-	54.00	32.73	44
IRC_IRT_c42490	104	EEC75769.1	0.0	GO:0009536	100.00	74.71	34
IRC_IRT_c42520	116	XP_002537142.1	0.0	-	72.00	41.20	37
IRC_IRT_c42547	130	XP_002539706.1	0.0	GO:0009507	83.00	62.77	42
IRC_IRT_c42579	172	AIU45506.1	0.0	GO:0016021	87.00	66.24	40
IRC_IRT_c42588	127	EAZ02584.1	0.0	GO:0009536	95.00	90.12	42
IRC_IRT_c42662	214	BAD81488.1	0.0	GO:0004523	98.00	126.33	67
IRC_IRT_c42700	185	AFG63544.1	0.0	-	59.00	47.75	49
IRC_IRT_c42709	106	EEC77998.1	0.1	-	67.00	34.65	34
IRC_IRT_c42749	133	XP_004253340.1	0.0	GO:0016874	88.00	79.34	44
IRC_IRT_c42776	133	XP_009398204.1	0.7	-	48.00	32.34	41
IRC_IRT_c42820	105	XP_004959420.1	0.0	GO:0010090	80.00	53.91	35
IRC_IRT_c42852	148	XP_002536967.1	0.0	-	72.00	40.82	36
IRC_IRT_c42907	119	EMS56529.1	0.0	GO:1901701	75.00	50.45	40
IRC_IRT_c42950	136	XP_002540587.1	0.0	-	64.00	41.59	37
IRC_IRT_c42962	158	XP_002540016.1	0.0	GO:0009306	94.00	62.77	34
IRC_IRT_c42968	154	CCO18989.1	0.0	GO:0009651	76.00	61.23	50
IRC_IRT_c43035	113	XP_003061499.1	0.0	-	54.00	36.19	37
IRC_IRT_c43079	107	CDY12012.1	0.0	-	70.00	37.35	34
IRC_IRT_c43111	111	NP_001062503.1	0.0	GO:0005507	100.00	85.50	37
IRC_IRT_c43145	164	XP_002535080.1	0.0	-	58.00	39.28	48
IRC_IRT_c43168	244	XP_005650640.1	0.0	GO:0009536	69.00	70.48	63
IRC_IRT_c43218	114	BAG91734.1	0.0	GO:0016020	73.00	55.07	42
IRC_IRT_c43241	119	EEC81163.1	0.0	-	72.00	38.12	36
IRC_IRT_c43280	117	XP_004253340.1	0.0	-	89.00	61.62	38
IRC_IRT_c43288	198	XP_002467304.1	0.0	-	73.00	55.84	45
IRC_IRT_c43300	107	XP_001787012.1	0.0	-	100.00	36.58	35
IRC_IRT_c43307	114	XP_001773456.1	0.0	GO:0005840	79.00	39.28	34
IRC_IRT_c43310	189	AEW08215.1	0.0	GO:0006979	84.00	59.31	39
IRC_IRT_c43318	129	XP_009350065.1	0.0	GO:0006412	89.00	57.38	37
IRC_IRT_c43328	111	XP_002539042.1	0.0	GO:0004872	89.00	65.08	37
IRC_IRT_c43358	118	XP_002534939.1	0.0	GO:0006810	84.00	56.61	39

IRC_IRT_c43359	137	AAM19013.1	0.0	GO:0004523	91.00	70.86	35
IRC_IRT_c43371	109	XP_002505678.1	0.0	-	69.00	36.58	36
IRC_IRT_c43395	169	YP_009106890.1	0.0	GO:0005840	92.00	88.97	51
IRC_IRT_c43443	215	XP_006607083.1	0.0	-	60.00	43.13	46
IRC_IRT_c43450	138	YP_001019160.1	0.0	GO:0044444	69.00	47.75	42
IRC_IRT_c43455	199	XP_001772790.1	0.2	-	55.00	35.04	47
IRC_IRT_c43461	182	XP_007151457.1	0.3	-	60.00	34.27	38
IRC_IRT_c43468	180	KJB47628.1	0.0	-	55.00	36.97	58
IRC_IRT_c43481	116	XP_001418926.1	0.0	GO:0016556	81.00	50.06	38
IRC_IRT_c43488	111	ACN23298.1	0.0	GO:0003723	75.00	43.90	36
IRC_IRT_c43492	108	EEC76122.1	0.0	GO:0050660	83.00	61.23	36
IRC_IRT_c43503	290	XP_003614377.1	0.5	-	60.00	34.65	45
IRC_IRT_c43516	114	XP_003629958.1	0.0	GO:0051536	72.00	40.05	36
IRC_IRT_c43519	105	CAE02992.2	0.0	GO:0009536	97.00	72.79	34
IRC_IRT_c43527	123	NP_001172165.1	0.0	GO:0016023	100.00	83.19	41
IRC_IRT_c43534	112	XP_011016747.1	0.0	GO:0050660	94.00	61.62	36
IRC_IRT_c43556	102	XP_006590811.1	0.0	-	68.00	39.66	35
IRC_IRT_c43597	173	KCW57974.1	0.3	-	52.00	34.27	36
IRC_IRT_c43640	109	CAD40482.1	0.0	GO:0004523	100.00	76.26	3.50E+01
IRC_IRT_c43645	129	XP_001751507.1	0.0	GO:0016787	64.00	43.90	39
IRC_IRT_c43721	134	CBI29764.3	0.2	-	66.00	34.65	45
IRC_IRT_c43771	111	EAY94460.1	0.0	GO:0008131	100.00	72.40	34
IRC_IRT_c43807	114	XP_002535596.1	0.0	-	75.00	48.52	36
IRC_IRT_c43815	138	EYU19642.1	0.1	-	59.00	35.04	44
IRC_IRT_c43899	123	XP_002539322.1	0.0	-	72.00	39.28	40
IRC_IRT_c44008	114	YP_001019103.1	0.0	GO:0016779	72.00	46.21	37
IRC_IRT_c44041	155	AAM94925.1	0.0	GO:0008270	100.00	102.06	45
IRC_IRT_c44048	112	DAA59886.1	0.0	-	72.00	36.58	37
IRC_IRT_c44085	150	KJB07029.1	0.1	-	63.00	34.65	41
IRC_IRT_c44165	127	AFW70741.1	0.0	GO:0006633	79.00	54.68	39
IRC_IRT_c44169	114	XP_002538822.1	0.0	GO:0016787	71.00	43.13	35
IRC_IRT_c44171	147	XP_002535112.1	0.0	GO:0055085	81.00	80.11	49
IRC_IRT_c44270	142	XP_009591321.1	0.5	-	54.00	33.50	48

IRC_IRT_c44271	102	XP_005647824.1	0.0	-	88.00	53.53	34
IRC_IRT_c44291	141	CCH14737.1	0.0	-	64.00	34.27	34
IRC_IRT_c44325	102	BAJ97253.1	0.0	-	64.00	37.74	34
IRC_IRT_c44367	117	DAA56411.1	0.0	GO:0016798	76.00	47.75	38
IRC_IRT_c44384	110	EEC83773.1	0.0	GO:0016021	80.00	57.77	36
IRC_IRT_c44401	155	EEC67144.1	0.0	GO:0005215	84.00	74.33	50
IRC_IRT_c44490	114	XP_007146040.1	0.0	GO:0009086	89.00	76.26	38
IRC_IRT_c44577	149	XP_002534939.1	0.1	-	58.00	35.81	48
IRC_IRT_c44585	110	KCW63711.1	0.3	-	58.00	33.11	34
IRC_IRT_c44597	136	XP_009338809.1	0.3	-	54.00	33.88	35
IRC_IRT_c44601	105	CEF98043.1	0.0	-	74.00	40.82	35
IRC_IRT_c44606	106	XP_006409994.1	0.1	-	65.00	34.27	35
IRC_IRT_c44626	135	XP_007015201.1	0.1	-	56.00	35.81	37
IRC_IRT_c44650	107	AEY84979.1	0.0	GO:0005515	71.00	54.68	35
IRC_IRT_c44651	143	AAK92675.1	0.0	GO:0003676	91.00	82.80	47
IRC_IRT_c44661	142	AAF16526.1	0.0	GO:0000166	69.00	49.29	39
IRC_IRT_c44666	110	XP_002537832.1	0.0	-	81.00	41.59	37
IRC_IRT_c44719	109	KGN48828.1	0.0	GO:0044763	71.00	44.28	35
IRC_IRT_c44745	139	KEH24988.1	0.0	GO:0003824	70.00	42.36	44
IRC_IRT_c44765	211	KGN61861.1	0.0	-	76.00	50.06	34
IRC_IRT_c44772	140	CAE05257.2	0.0	GO:0016023	97.00	72.02	34
IRC_IRT_c44843	126	XP_009350076.1	0.0	GO:0005525	97.00	78.57	39
IRC_IRT_c44853	137	XP_002537142.1	0.5	-	51.00	33.11	45
IRC_IRT_c44857	195	XP_008780190.1	0.0	GO:0004553	85.00	86.66	54
IRC_IRT_c44871	105	XP_011014726.1	0.0	-	91.00	55.84	34
IRC_IRT_c44881	102	AAX96177.1	0.0	-	70.00	43.13	34
IRC_IRT_c45056	127	XP_002534672.1	0.0	-	60.00	35.42	40
IRC_IRT_c45072	119	XP_006427342.1	0.2	-	63.00	34.65	38
IRC_IRT_c45075	113	EEE54546.1	0.0	GO:0005739	94.00	72.79	37
IRC_IRT_c45093	134	XP_002537175.1	0.0	-	65.00	38.12	43
IRC_IRT_c45095	117	EEC68421.1	0.0	GO:0006810	77.00	46.21	35
IRC_IRT_c45107	104	XP_006645799.1	0.0	GO:0031359	100.00	72.40	34
IRC_IRT_c45126	188	CCO14733.1	0.0	-	60.00	49.68	63

IRC_IRT_c45132	151	XP_009350072.1	0.0	GO:0003735	91.00	83.19	49
IRC_IRT_c45145	157	XP_005850795.1	0.1	-	77.00	36.19	49
IRC_IRT_c45146	133	XP_002539363.1	0.0	GO:0097159	75.00	46.98	36
IRC_IRT_c45149	128	CCO65929.1	0.0	GO:0003723	75.00	42.36	37
IRC_IRT_c45157	129	XP_009350072.1	0.0	GO:0006412	88.00	77.80	43
IRC_IRT_c45162	138	AFW66836.1	0.0	GO:0042026	68.00	46.60	41
IRC_IRT_c45164	242	AAV44205.1	0.0	-	74.00	41.97	35
IRC_IRT_c45170	152	KIY95293.1	0.4	-	54.00	33.50	35
IRC_IRT_c45176	140	BAK02541.1	0.0	-	63.00	38.89	38
IRC_IRT_c45188	179	KIZ02813.1	0.0	-	73.00	51.99	49
IRC_IRT_c45213	116	BAJ21381.1	0.0	GO:0046933	80.00	65.08	45
IRC_IRT_c45222	223	XP_002488946.1	0.0	-	58.00	38.89	41
IRC_IRT_c45224	215	XP_009388835.1	0.1	-	56.00	35.81	46
IRC_IRT_c45226	196	EYU38116.1	0.2	-	57.00	34.65	45
IRC_IRT_c45227	210	ABR17750.1	0.0	GO:0005488	63.00	54.30	66
IRC_IRT_c45239	248	XP_003610227.1	0.0	-	75.00	43.90	36
IRC_IRT_c45271	127	XP_002536230.1	0.0	-	57.00	37.35	40
IRC_IRT_c45293	134	XP_004253383.1	0.0	-	71.00	40.05	35
IRC_IRT_c45298	114	XP_006574318.1	0.5	-	58.00	30.80	36
IRC_IRT_c45303	115	EMS47108.1	0.7	-	59.00	32.34	37
IRC_IRT_c45304	190	CAE05627.1	0.0	GO:0005739	76.00	80.11	56
IRC_IRT_c45307	144	XP_010911665.1	0.0	GO:0016874	91.00	80.11	46
IRC_IRT_c45377	183	XP_003063513.1	0.1	-	53.00	36.19	45
IRC_IRT_c45405	114	EMT03091.1	0.0	GO:0006281	75.00	39.66	36
IRC_IRT_c45448	131	XP_011099533.1	0.5	-	57.00	33.11	38
IRC_IRT_c45482	111	NP_001183128.1	0.0	GO:0009630	100.00	78.95	37
IRC_IRT_c45527	183	XP_007044166.1	0.0	GO:0044699	59.00	53.53	57
IRC_IRT_c45529	148	XP_007152843.1	0.6	-	47.00	33.11	38
IRC_IRT_c45548	149	CAE02453.2	0.0	GO:0004523	95.00	98.60	45
IRC_IRT_c45561	111	XP_011627293.1	0.0	-	59.00	37.35	37
IRC_IRT_c45572	155	XP_009121307.1	0.5	-	55.00	33.50	34
IRC_IRT_c45606	120	EEC72951.1	0.0	GO:0005739	89.00	65.47	39
IRC_IRT_c45613	185	YP_002601055.1	0.0	-	73.00	37.74	34

IRC_IRT_c45630	124	ABR26215.1	0.0	-	61.00	40.05	36
IRC_IRT_c45660	108	NP_001045604.2	0.0	GO:0016020	100.00	77.80	36
IRC_IRT_c45732	149	XP_009350805.1	0.0	GO:0003824	70.00	55.45	48
IRC_IRT_c45745	106	EEC72917.1	0.0	GO:0055114	100.00	69.71	35
IRC_IRT_c45748	116	AAR06372.1	0.0	-	86.00	60.85	36
IRC_IRT_c45749	102	XP_011014727.1	0.0	-	97.00	58.15	34
IRC_IRT_c45793	131	XP_010911754.1	0.0	-	64.00	38.89	37
IRC_IRT_c45868	117	XP_002276771.1	0.0	GO:0016758	92.00	75.87	38
IRC_IRT_c45925	111	CDP05076.1	0.0	GO:0015930	71.00	41.97	35
IRC_IRT_c45926	121	XP_011461758.1	0.8	-	62.00	32.34	35
IRC_IRT_c45943	130	NP_001078446.1	0.0	GO:0034040	86.00	67.01	43
IRC_IRT_c45964	137	EEE70182.1	0.0	GO:0016023	83.00	60.46	37
IRC_IRT_c46022	110	XP_002537142.1	0.0	-	82.00	51.60	35
IRC_IRT_c46039	157	AAX96554.1	0.0	GO:0004523	90.00	85.89	52
IRC_IRT_c46040	118	AAX96629.1	0.0	GO:0044260	78.00	42.36	37
IRC_IRT_c46045	105	KIZ00023.1	0.0	-	91.00	63.54	35
IRC_IRT_c46093	114	XP_001786972.1	0.0	-	69.00	40.43	36
IRC_IRT_c46094	123	XP_003609401.1	0.0	-	64.00	36.19	34
IRC_IRT_c46114	144	XP_007042245.1	0.5	-	55.00	33.50	49
IRC_IRT_c46124	126	XP_002320110.2	0.3	-	62.00	33.50	35
IRC_IRT_c46188	250	BAD73023.1	0.9	-	48.00	33.11	50
IRC_IRT_c46196	113	XP_001698501.1	0.0	-	67.00	39.28	37
IRC_IRT_c46214	152	EMT20630.1	0.0	GO:0005524	91.00	57.38	35
IRC_IRT_c46243	157	XP_002966336.1	0.0	GO:0016772	67.00	45.44	43
IRC_IRT_c46259	145	BAE93159.1	0.0	-	58.00	37.35	50
IRC_IRT_c46280	146	YP_001109553.1	0.0	-	52.00	37.35	50
IRC_IRT_c46327	107	NP_001172912.1	0.0	GO:0017148	97.00	69.32	35
IRC_IRT_c46334	157	XP_001767506.1	0.3	-	54.00	34.27	44
IRC_IRT_c46339	118	XP_002540527.1	0.0	-	82.00	57.00	39
IRC_IRT_c46416	124	AAR87220.1	0.0	GO:0004523	100.00	90.51	4.00E+01
IRC_IRT_c46443	149	XP_002977223.1	0.0	-	70.00	49.29	40
IRC_IRT_c46471	193	AAV32231.1	0.0	-	76.00	34.65	42
IRC_IRT_c46513	123	EEC68546.1	0.0	-	80.00	55.84	41

IRC_IRT_c46514	130	XP_002959988.1	0.0	-	61.00	38.12	42
IRC_IRT_c46526	127	CAI44611.1	0.0	GO:0003676	80.00	65.08	41
IRC_IRT_c46557	105	EEC71017.1	0.0	GO:0016023	100.00	80.88	34
IRC_IRT_c46559	111	CDY06798.1	0.1	-	60.00	34.27	35
IRC_IRT_c46572	113	XP_009353814.1	0.0	-	59.00	35.04	37
IRC_IRT_c46586	105	NP_001059418.1	0.0	-	100.00	72.40	34
IRC_IRT_c46613	216	AAB61446.1	0.0	-	54.00	37.35	44
IRC_IRT_c46684	130	XP_002539997.1	0.6	-	62.00	32.34	45
IRC_IRT_c46718	116	XP_002537667.1	0.0	-	91.00	71.63	37
IRC_IRT_c46730	232	XP_002540059.1	0.0	-	86.00	74.33	73
IRC_IRT_c46756	112	XP_009149951.1	0.1	-	69.00	35.04	36
IRC_IRT_c46759	108	EYU40481.1	0.0	-	70.00	39.28	34
IRC_IRT_c46794	121	CAN79146.1	0.1	-	68.00	35.04	35
IRC_IRT_c46809	102	XP_009348152.1	0.0	-	77.00	36.19	35
IRC_IRT_c46813	108	XP_002536628.1	0.0	-	67.00	41.59	34
IRC_IRT_c46832	136	XP_002539628.1	0.0	-	66.00	40.05	39
IRC_IRT_c46836	119	XP_002535235.1	0.0	GO:0008152	86.00	65.08	38
IRC_IRT_c46846	131	XP_001692412.1	0.2	-	63.00	33.88	36
IRC_IRT_c46873	231	XP_002535387.1	0.0	-	49.00	42.36	73
IRC_IRT_c46971	112	AAN16331.1	0.0	GO:0003964	100.00	80.49	36
IRC_IRT_c46974	156	EEE51555.1	0.8	-	48.00	31.19	49
IRC_IRT_c47009	115	KEH15190.1	0.0	GO:0006810	72.00	45.05	37
IRC_IRT_c47024	151	BAD81211.1	0.3	-	64.00	33.88	42
IRC_IRT_c47038	154	AFW85603.1	0.0	GO:0007127	75.00	58.15	48
IRC_IRT_c47057	114	XP_002510718.1	0.4	-	54.00	33.50	37
IRC_IRT_c47091	158	XP_002986980.1	0.0	GO:0008152	72.00	53.91	40
IRC_IRT_c47134	104	EEC83566.1	0.0	-	85.00	56.23	34
IRC_IRT_c47140	124	XP_010245330.1	0.2	-	64.00	33.88	34
IRC_IRT_c47169	101	ABR17785.1	0.0	GO:0004834	80.00	51.60	36
IRC_IRT_c47312	131	NP_038351.1	0.0	-	68.00	38.12	38
IRC_IRT_c47329	206	CCO14048.1	0.0	-	66.00	40.82	48
IRC_IRT_c47359	121	CDP14826.1	0.0	GO:0009536	71.00	46.98	38
IRC_IRT_c47369	109	XP_002960003.1	0.0	-	58.00	36.58	36

IRC_IRT_c47373	176	EDQ48677.1	0.0	GO:0008152	81.00	78.57	55
IRC_IRT_c47381	133	XP_006582510.1	0.5	-	62.00	33.50	40
IRC_IRT_c47384	136	KDP39721.1	0.8	-	59.00	32.34	42
IRC_IRT_c47405	136	XP_002535691.1	0.0	-	72.00	39.28	40
IRC_IRT_c47446	105	XP_003064536.1	0.0	-	79.00	38.89	34
IRC_IRT_c47479	108	XP_010466741.1	0.0	GO:0005829	100.00	75.49	36
IRC_IRT_c47523	103	NP_001049712.1	0.0	GO:0005524	100.00	76.26	34
IRC_IRT_c47541	114	XP_002536774.1	0.1	-	63.00	34.65	36
IRC_IRT_c47544	105	NP_001060818.1	0.0	GO:0016020	100.00	77.03	34
IRC_IRT_c47596	108	AAY42573.1	0.0	GO:0006810	73.00	45.82	34
IRC_IRT_c47614	174	XP_001776934.1	0.0	GO:0044249	69.00	56.23	46
IRC_IRT_c47709	118	BAK02349.1	0.4	-	64.00	33.11	37
IRC_IRT_c47712	111	CAD39762.2	0.0	GO:0003676	97.00	76.26	35
IRC_IRT_c47728	178	XP_002536552.1	0.4	-	53.00	33.50	47
IRC_IRT_c47835	103	XP_007137831.1	0.0	-	70.00	38.89	34
IRC_IRT_c47841	145	XP_001695632.1	0.0	GO:0006099	85.00	81.65	4.70E+01
IRC_IRT_c47866	120	KIZ03504.1	0.0	-	61.00	36.58	39
IRC_IRT_c47892	107	XP_008647734.1	0.4	-	63.00	33.50	38
IRC_IRT_c47928	123	XP_002503874.1	0.0	-	80.00	60.85	40
IRC_IRT_c48047	103	AAX95853.1	0.1	-	79.00	35.04	34
IRC_IRT_c48083	120	XP_001700103.1	0.0	GO:0031425	85.00	67.40	40
IRC_IRT_c48093	126	XP_006646404.1	0.0	GO:0016023	83.00	46.21	42
IRC_IRT_c48100	148	XP_008786789.1	0.4	-	51.00	33.88	35
IRC_IRT_c48110	103	XP_010911770.1	0.0	-	94.00	64.70	34
IRC_IRT_c48155	115	XP_002535552.1	0.0	GO:0003824	67.00	44.67	37
IRC_IRT_c48160	173	AAF16525.1	0.0	GO:0000166	91.00	99.37	56
IRC_IRT_c48169	113	XP_008807042.1	0.0	-	62.00	40.43	35
IRC_IRT_c48224	196	BAB08213.2	0.0	GO:0004523	92.00	126.72	65
IRC_IRT_c48229	125	XP_002503140.1	0.5	-	64.00	33.11	39
IRC_IRT_c48246	143	XP_001786560.1	0.0	GO:0006289	87.00	63.93	47
IRC_IRT_c48284	103	BAJ53171.1	0.0	-	70.00	39.28	34
IRC_IRT_c48334	131	XP_001778207.1	0.0	GO:0009941	78.00	58.54	38
IRC_IRT_c48357	108	XP_004969724.1	0.6	-	67.00	32.73	34

IRC_IRT_c48500	111	YP_635949.1	0.0	-	88.00	39.66	36
IRC_IRT_c48501	114	EEC78869.1	0.0	GO:0003676	94.00	75.49	37
IRC_IRT_c48538	109	AAO17005.1	0.0	GO:0003723	94.00	74.71	35
IRC_IRT_c48543	175	XP_002489102.1	0.0	-	94.00	64.70	34
IRC_IRT_c48570	106	EEE68527.1	0.0	-	65.00	36.19	35
IRC_IRT_c48622	103	AAU90142.1	0.3	-	58.00	33.11	34
IRC_IRT_c48657	133	CDP09058.1	0.1	-	57.00	35.42	40
IRC_IRT_c48710	137	XP_002535650.1	0.0	-	62.00	46.21	43
IRC_IRT_c48722	107	XP_002538092.1	0.0	GO:0008928	79.00	51.99	34
IRC_IRT_c48780	108	AAU04862.1	0.1	-	60.00	34.65	35
IRC_IRT_c48831	156	CEG00861.1	0.0	-	73.00	43.13	34
IRC_IRT_c48855	117	EDQ48469.1	0.0	-	70.00	45.05	40
IRC_IRT_c48877	122	Q7XFK2.1	0.0	GO:0005975	97.00	79.72	39
IRC_IRT_c48880	128	EYU25062.1	0.0	-	67.00	36.58	34
IRC_IRT_c48894	158	XP_001694415.1	0.9	-	65.00	31.96	47
IRC_IRT_c48922	106	P45623.1	0.1	-	68.00	35.42	35
IRC_IRT_c48931	140	EEC83603.1	0.8	-	53.00	32.73	39
IRC_IRT_c48958	117	XP_002538677.1	0.0	GO:0008272	74.00	43.13	35
IRC_IRT_c48985	113	EYU40280.1	0.9	-	51.00	31.57	39
IRC_IRT_c49010	110	NP_001055418.1	0.0	GO:0005802	97.00	76.64	36
IRC_IRT_c49067	104	KIY95373.1	0.0	-	64.00	40.82	34
IRC_IRT_c49070	139	NP_001175253.1	0.1	-	61.00	35.04	39
IRC_IRT_c49082	108	EDQ48466.1	0.0	-	71.00	39.66	35
IRC_IRT_c49087	114	CAE05591.1	0.0	GO:0008270	91.00	68.17	37
IRC_IRT_c49143	161	AID67469.1	0.0	GO:0030529	76.00	68.94	51
IRC_IRT_c49167	155	EMS52220.1	0.6	-	46.00	33.11	49
IRC_IRT_c49188	192	ACK56134.1	0.0	GO:0048027	82.00	52.37	35
IRC_IRT_c49222	104	EEC77111.1	0.0	GO:0006935	82.00	44.28	34
IRC_IRT_c49263	105	XP_002538597.1	0.0	-	67.00	35.81	34
IRC_IRT_c49270	249	KJB15865.1	0.0	-	72.00	57.38	44
IRC_IRT_c49305	102	NP_001067957.2	0.0	GO:0006355	79.00	67.40	39
IRC_IRT_c49322	129	CAN83844.1	0.0	GO:0006996	69.00	45.44	39
IRC_IRT_c49369	127	EEE68528.1	0.0	GO:0006810	74.00	43.13	35

IRC_IRT_c49384	154	XP_002539604.1	0.0	-	58.00	36.58	50
IRC_IRT_c49387	110	KIZ05174.1	0.0	GO:0008152	75.00	58.15	36
IRC_IRT_c49489	191	AAT81665.1	0.0	GO:0004523	78.00	77.03	56
IRC_IRT_c49540	124	XP_002536223.1	0.0	-	70.00	36.97	41
IRC_IRT_c49549	128	AAV32160.1	0.0	-	100.00	37.74	40
IRC_IRT_c49552	142	BAM17119.1	0.0	GO:0016740	63.00	43.90	44
IRC_IRT_c49584	133	AAZ29202.1	0.0	GO:0046872	77.00	69.32	44
IRC_IRT_c49643	139	XP_003571638.1	0.0	-	69.00	38.51	36
IRC_IRT_c49656	143	XP_002537908.1	0.0	-	67.00	43.90	43
IRC_IRT_c49672	175	KEH15260.1	0.0	-	72.00	44.67	40
IRC_IRT_c49724	104	XP_004961914.1	0.0	GO:0060919	100.00	81.65	34
IRC_IRT_c49771	134	NP_001054214.1	0.0	GO:0010189	100.00	90.51	44
IRC_IRT_c49802	134	EEC77111.1	0.0	GO:0006935	86.00	59.31	44
IRC_IRT_c49878	122	XP_005846937.1	0.0	-	70.00	41.59	40
IRC_IRT_c49881	184	EEC70767.1	0.4	-	69.00	33.88	46
IRC_IRT_c49898	137	KDD73392.1	0.0	-	65.00	39.28	43
IRC_IRT_c49919	130	XP_006661569.1	0.0	GO:0000272	91.00	73.56	35
IRC_IRT_c49927	162	CCO65884.1	0.0	GO:0016740	66.00	45.82	53
IRC_IRT_c49928	137	XP_010904566.1	0.0	-	65.00	39.28	43
IRC_IRT_c49934	204	XP_002306636.2	0.2	-	53.00	34.27	41
IRC_IRT_c50018	136	EMT02543.1	0.1	-	55.00	34.27	43
IRC_IRT_c50034	156	XP_009350809.1	0.0	-	78.00	46.60	37
IRC_IRT_c50045	112	XP_002535080.1	0.0	-	64.00	38.12	37
IRC_IRT_c50058	112	NP_001068200.1	0.0	-	97.00	74.71	3.50E+01
IRC_IRT_c50189	123	KFM28466.1	0.2	-	57.00	34.27	40
IRC_IRT_c50212	240	EPS74345.1	0.0	-	49.00	36.97	59
IRC_IRT_c50265	123	EEC76347.1	0.0	-	100.00	85.89	40
IRC_IRT_c50272	133	EEC84789.1	0.0	GO:0009536	95.00	67.01	41
IRC_IRT_c50278	187	KJB44121.1	0.0	GO:0005488	71.00	59.31	49
IRC_IRT_c50303	133	XP_002536081.1	1.0	-	81.00	32.34	43
IRC_IRT_c50317	124	EEC75022.1	0.0	GO:0010413	100.00	85.89	40
IRC_IRT_c50348	104	CAE05399.1	0.0	GO:0003676	97.00	70.48	34
IRC_IRT_c50418	153	XP_002540107.1	0.0	-	73.00	50.45	42

IRC_IRT_c50457	140	KEH17345.1	0.4	-	60.00	33.11	35
IRC_IRT_c50485	113	EEC70905.1	0.0	GO:0003857	89.00	56.23	37
IRC_IRT_c50504	119	XP_002537315.1	0.0	GO:0016021	91.00	63.16	35
IRC_IRT_c50557	150	EXC27886.1	0.0	GO:0044763	75.00	67.40	48
IRC_IRT_c50568	131	NP_001174701.1	0.0	-	92.00	38.51	42
IRC_IRT_c50660	103	EAY92168.1	0.0	GO:0016301	100.00	45.44	34
IRC_IRT_c50680	103	KEH15415.1	0.0	-	61.00	36.97	34
IRC_IRT_c50708	127	XP_002965500.1	0.0	-	66.00	45.82	42
IRC_IRT_c50727	128	CAI44641.1	0.0	GO:0004672	67.00	45.82	34
IRC_IRT_c50752	118	XP_010929661.1	0.0	-	71.00	36.19	35
IRC_IRT_c50763	115	EAY97777.1	0.6	-	61.00	32.73	34
IRC_IRT_c50802	101	XP_006656947.1	0.0	GO:0006468	78.00	57.38	37
IRC_IRT_c50825	113	XP_002538274.1	0.0	GO:0016740	86.00	54.68	37
IRC_IRT_c50857	123	NP_001065692.1	0.0	GO:0004725	100.00	44.28	41
IRC_IRT_c50934	170	XP_001775835.1	0.2	-	56.00	34.65	55
IRC_IRT_c50957	124	CAH66260.1	0.0	GO:0005739	100.00	91.28	41
IRC_IRT_c50968	147	XP_008666326.1	0.0	GO:0008907	80.00	75.10	47
IRC_IRT_c51024	121	EMS47644.1	0.4	-	52.00	33.88	36
IRC_IRT_c51124	105	KDD73662.1	0.0	-	57.00	37.74	35
IRC_IRT_c51169	147	XP_002507785.1	0.0	-	63.00	39.28	41
IRC_IRT_c51203	110	XP_010045095.1	0.0	-	69.00	40.43	42
IRC_IRT_c51209	126	XP_010232978.1	0.1	-	48.00	35.04	39
IRC_IRT_c51228	133	AEQ94132.1	0.0	GO:0016746	60.00	42.36	43
IRC_IRT_c51258	138	XP_002539941.1	0.1	-	51.00	34.65	39
IRC_IRT_c51298	112	EXB54601.1	0.0	-	71.00	38.89	35
IRC_IRT_c51307	107	XP_002534896.1	0.0	-	67.00	36.97	34
IRC_IRT_c51317	122	EEE68526.1	0.0	-	82.00	60.08	40
IRC_IRT_c51335	124	EAY84569.1	0.0	GO:0050660	86.00	61.62	38
IRC_IRT_c51338	115	XP_005848152.1	0.0	GO:0019829	75.00	48.52	36
IRC_IRT_c51398	117	XP_006401295.1	0.3	-	66.00	33.88	36
IRC_IRT_c51432	133	BAC65934.1	0.0	GO:0005739	100.00	89.74	44
IRC_IRT_c51451	135	XP_003539456.1	0.6	-	51.00	33.11	45
IRC_IRT_c51466	148	CBY84994.1	0.0	GO:0046872	72.00	57.77	48

IRC_IRT_c51474	141	XP_009350067.1	0.0	GO:0005840	88.00	74.33	45
IRC_IRT_c51493	125	XP_002536964.1	0.3	-	60.00	33.50	35
IRC_IRT_c51528	122	KJB20774.1	0.0	-	62.00	44.67	37
IRC_IRT_c51538	119	YP_009106339.1	0.0	GO:0003735	94.00	62.77	36
IRC_IRT_c51560	127	AAL82208.1	0.0	GO:0008137	78.00	67.40	41
IRC_IRT_c51598	138	XP_002536310.1	0.0	-	58.00	36.58	46
IRC_IRT_c51605	195	KFK29530.1	0.5	-	42.00	33.88	61
IRC_IRT_c51615	112	KDD75881.1	0.9	-	66.00	31.96	36
IRC_IRT_c51616	293	AAV44205.1	0.0	-	80.00	63.16	45
IRC_IRT_c51626	121	XP_002460274.1	0.0	GO:0020037	100.00	85.50	39
IRC_IRT_c51632	181	XP_010910917.1	0.1	-	46.00	35.04	56
IRC_IRT_c51647	184	YP_002600949.1	0.0	GO:0003899	93.00	106.69	60
IRC_IRT_c51654	141	AAV44205.1	0.0	-	94.00	64.70	34
IRC_IRT_c51665	167	XP_001695478.1	0.0	-	76.00	65.47	46
IRC_IRT_c51699	183	ERN00555.1	0.8	-	53.00	31.96	47
IRC_IRT_c51701	103	XP_010252158.1	0.0	GO:0005622	70.00	46.21	34
IRC_IRT_c51702	168	KEH21085.1	0.0	-	71.00	50.83	46
IRC_IRT_c51723	150	ERN16843.1	0.7	-	61.00	31.57	34
IRC_IRT_c51751	135	EEC82178.1	0.0	-	91.00	63.54	34
IRC_IRT_c51755	123	XP_003627937.1	0.0	-	65.00	41.97	35
IRC_IRT_c51772	176	XP_011623084.1	0.6	-	52.00	33.11	42
IRC_IRT_c51778	156	XP_001786886.1	0.0	GO:0006402	100.00	78.18	41
IRC_IRT_c51780	110	XP_005643737.1	0.0	GO:0005739	72.00	49.68	36
IRC_IRT_c51785	107	XP_002535224.1	0.0	GO:0006412	91.00	68.55	35
IRC_IRT_c51813	117	XP_002464338.1	0.6	-	55.00	32.73	36
IRC_IRT_c51815	134	CAH66587.1	0.0	GO:0004523	88.00	56.23	35
IRC_IRT_c51841	120	XP_003081351.1	0.0	-	65.00	36.19	35
IRC_IRT_c51848	139	XP_010491408.1	0.8	-	65.00	32.73	43
IRC_IRT_c51863	129	NP_001176697.1	0.0	GO:0008270	85.00	77.03	42
IRC_IRT_c51866	108	XP_002539099.1	0.0	GO:0005737	82.00	55.84	35
IRC_IRT_c51872	118	EDQ49172.1	0.3	-	64.00	33.88	34
IRC_IRT_c51873	211	XP_003064995.1	0.0	-	56.00	39.28	41
IRC_IRT_c51911	110	XP_006829520.1	0.0	GO:0008152	72.00	44.28	36

IRC_IRT_c51933	109	XP_002535648.1	0.1	-	61.00	34.65	36
IRC_IRT_c51946	112	EEE62395.1	0.0	GO:0009678	100.00	69.32	37
IRC_IRT_c51962	109	EAZ10035.1	0.0	GO:0005739	100.00	77.41	35
IRC_IRT_c51969	125	EDQ48497.1	0.0	-	66.00	38.89	39
IRC_IRT_c52110	114	XP_007207207.1	0.0	GO:0080041	100.00	80.11	37
IRC_IRT_c52173	182	XP_010443506.1	0.1	-	50.00	35.42	51
IRC_IRT_c52183	111	KIY92252.1	0.3	-	61.00	33.11	36
IRC_IRT_c52185	102	XP_002504034.1	0.0	GO:0006413	85.00	51.60	34
IRC_IRT_c52216	117	KDD71878.1	0.0	GO:0003995	74.00	50.45	39
IRC_IRT_c52244	223	AAP44597.1	0.0	GO:0004523	89.00	102.06	56
IRC_IRT_c52317	112	EAZ00187.1	0.0	GO:0016758	100.00	88.97	37
IRC_IRT_c52320	135	ABI48270.1	0.0	-	61.00	38.51	44
IRC_IRT_c52335	105	EYU32409.1	0.9	-	57.00	31.96	38
IRC_IRT_c52341	108	EEC81137.1	0.0	-	85.00	63.16	35
IRC_IRT_c52347	106	XP_002269887.1	0.2	-	57.00	31.96	35
IRC_IRT_c52354	107	EEC77111.1	0.0	GO:0006935	82.00	50.45	35
IRC_IRT_c52359	123	XP_002508801.1	0.0	GO:0006200	82.00	53.14	39
IRC_IRT_c52368	113	CBX24413.1	0.0	GO:0006355	100.00	53.91	37
IRC_IRT_c52383	115	ABF99659.1	0.0	GO:0005739	100.00	79.72	38
IRC_IRT_c52416	118	XP_003571468.1	0.0	GO:0004386	100.00	82.80	38
IRC_IRT_c52418	132	BAJ89001.1	0.0	GO:0005874	88.00	80.88	44
IRC_IRT_c52430	115	XP_002887991.1	0.7	-	52.00	32.73	38
IRC_IRT_c52432	136	CEF97632.1	0.1	-	67.00	35.04	34
IRC_IRT_c52437	136	XP_002536793.1	0.0	GO:0016868	75.00	58.54	45
IRC_IRT_c52453	177	XP_002974782.1	0.0	GO:0006796	61.00	51.99	55
IRC_IRT_c52486	167	XP_002507787.1	0.0	-	61.00	39.28	47
IRC_IRT_c52539	116	XP_002961432.1	0.0	-	68.00	40.43	38
IRC_IRT_c52541	112	EAY98272.1	0.0	GO:0005089	100.00	79.34	37
IRC_IRT_c52547	101	XP_005643261.1	0.0	-	67.00	38.12	34
IRC_IRT_c52562	106	XP_002891370.1	0.2	-	61.00	34.27	36
IRC_IRT_c52584	123	XP_011460718.1	0.9	-	60.00	32.34	35
IRC_IRT_c52612	109	ABB46999.1	0.0	GO:0005739	88.00	67.40	36
IRC_IRT_c52613	154	AAT85796.1	0.0	-	82.00	80.11	50

IRC_IRT_c52619	108	XP_001690955.1	0.0	GO:0016620	80.00	54.68	36
IRC_IRT_c52630	148	XP_002500636.1	0.2	-	54.00	34.65	46
IRC_IRT_c52752	112	XP_002535704.1	0.0	GO:0005975	97.00	71.63	37
IRC_IRT_c52774	103	EEE52320.1	0.0	-	70.00	37.35	34
IRC_IRT_c52796	142	XP_002535413.1	0.0	GO:0000166	73.00	59.69	45
IRC_IRT_c52800	132	DAA50350.1	0.0	GO:0008094	83.00	62.39	43
IRC_IRT_c52808	111	XP_002539513.1	0.0	-	97.00	74.71	36
IRC_IRT_c52867	106	KDD74139.1	0.0	-	68.00	43.90	35
IRC_IRT_c52904	115	ABB47074.2	0.0	GO:0003677	91.00	76.26	37
IRC_IRT_c52931	107	EEE67650.1	0.1	-	55.00	34.27	38
IRC_IRT_c52953	150	XP_002446151.1	0.1	-	42.00	35.42	56
IRC_IRT_c53109	105	XP_002539362.1	0.0	-	68.00	36.19	35
IRC_IRT_c53127	107	XP_009113218.1	0.2	-	67.00	34.27	34
IRC_IRT_c53192	103	EEE66929.1	0.0	-	94.00	65.86	34
IRC_IRT_c53213	119	KEH15665.1	0.1	-	67.00	35.42	37
IRC_IRT_c53245	108	KDD73440.1	0.0	GO:0005737	82.00	47.75	34
IRC_IRT_c53264	146	NP_001053617.1	0.0	GO:0016023	100.00	102.83	48
IRC_IRT_c53273	106	XP_009350059.1	0.6	-	65.00	32.34	35
IRC_IRT_c53275	109	XP_007015755.1	0.0	GO:0032508	80.00	53.53	35
IRC_IRT_c53284	146	XP_006406889.1	0.4	-	58.00	33.88	41
IRC_IRT_c53303	124	EEE53634.1	0.0	GO:0006468	100.00	82.80	40
IRC_IRT_c53313	158	ABG66008.1	0.0	GO:0006952	100.00	89.35	36
IRC_IRT_c53380	106	XP_002539099.1	0.1	-	63.00	33.50	38
IRC_IRT_c53401	125	XP_002954901.1	0.0	GO:0004871	68.00	44.67	41
IRC_IRT_c53438	109	XP_002536999.1	0.0	GO:0006355	81.00	45.44	37
IRC_IRT_c53440	133	XP_002537464.1	0.0	-	65.00	41.20	38
IRC_IRT_c53471	111	XP_002536161.1	0.0	GO:0009941	75.00	46.60	36
IRC_IRT_c53514	117	EAZ14447.1	0.0	GO:0005975	100.00	82.80	39
IRC_IRT_c53533	172	XP_002506367.1	0.0	-	57.00	37.35	47
IRC_IRT_c53545	108	XP_010237559.1	0.4	-	55.00	33.50	34
IRC_IRT_c53561	123	XP_003059953.1	0.7	-	60.00	32.34	38
IRC_IRT_c53571	135	EDQ48365.1	0.0	-	70.00	38.51	37
IRC_IRT_c53577	110	AAK76732.1	0.0	GO:0016747	82.00	47.37	35

IRC_IRT_c53607	118	XP_006650947.1	0.0	-	66.00	42.74	36
IRC_IRT_c53609	111	XP_002537565.1	0.0	-	70.00	43.13	37
IRC_IRT_c53711	127	XP_002538114.1	0.0	GO:0016763	75.00	46.21	36
IRC_IRT_c53723	125	EEC73884.1	0.0	-	100.00	43.13	41
IRC_IRT_c53729	112	ABA26988.1	0.0	-	64.00	40.82	34
IRC_IRT_c53845	133	XP_002504329.1	0.0	-	61.00	48.14	44
IRC_IRT_c53854	115	NP_001053250.1	0.0	GO:0016023	100.00	86.27	37
IRC_IRT_c53915	104	BAA84618.1	0.0	GO:0042545	100.00	72.40	34
IRC_IRT_c53920	108	XP_001695386.1	0.0	GO:0009845	85.00	52.76	35
IRC_IRT_c53939	161	ABA99774.1	0.0	GO:0005739	94.00	65.86	36
IRC_IRT_c53981	116	AAS86399.2	0.0	-	94.00	73.94	35
IRC_IRT_c53982	142	XP_006661149.1	0.0	-	69.00	46.98	42
IRC_IRT_c54003	112	XP_002960029.1	0.0	GO:0046912	80.00	51.60	36
IRC_IRT_c54109	213	CAE02127.2	0.0	GO:0004523	100.00	135.96	63
IRC_IRT_c54168	119	CAD40412.3	0.0	GO:0005488	70.00	41.97	37
IRC_IRT_c54212	143	XP_005847592.1	0.0	GO:0003824	74.00	46.21	35
IRC_IRT_c54226	312	XP_002488946.1	0.0	-	76.00	57.77	42
IRC_IRT_c54249	121	XP_001764850.1	0.0	-	65.00	41.20	38
IRC_IRT_c54311	176	BAD38000.1	0.0	GO:0009536	91.00	53.14	36
IRC_IRT_c54373	121	EEE63229.1	0.0	-	65.00	48.14	40
IRC_IRT_c54398	155	XP_002537435.1	0.0	GO:0043565	92.00	68.55	38
IRC_IRT_c54491	119	EAY93131.1	0.0	-	71.00	40.43	39
IRC_IRT_c54562	117	KFM23437.1	0.0	-	65.00	36.97	38
IRC_IRT_c54578	114	XP_002535428.1	0.0	-	63.00	38.51	36
IRC_IRT_c54663	124	ABA97186.1	0.0	-	69.00	40.05	36
IRC_IRT_c54689	187	KDP23383.1	0.0	GO:0070011	65.00	42.36	43
IRC_IRT_c54745	106	XP_009364804.1	0.0	GO:0008661	91.00	59.31	35
IRC_IRT_c54758	126	XP_002537284.1	0.9	-	50.00	31.57	40
IRC_IRT_c54803	166	XP_002539732.1	0.0	-	66.00	51.60	51
IRC_IRT_c54841	153	XP_009351044.1	0.0	-	72.00	43.13	37
IRC_IRT_c54862	118	CAE02876.1	0.0	-	68.00	43.90	35
IRC_IRT_c54889	118	AAU10686.1	0.0	GO:0009536	100.00	51.22	3.80E+01
IRC_IRT_c54921	112	XP_006848412.1	0.0	GO:0007010	97.00	75.10	36

IRC_IRT_c54964	125	EAZ39712.1	0.0	GO:0030786	97.00	53.91	41
IRC_IRT_c54968	111	BAD33614.1	0.0	GO:0016023	100.00	74.71	35
IRC_IRT_c54969	115	AAU44238.1	0.0	GO:0000145	89.00	68.55	38
IRC_IRT_c55029	165	XP_007017932.1	0.0	-	61.00	42.74	44
IRC_IRT_c55049	106	NP_001061444.1	0.0	GO:0016021	100.00	74.71	35
IRC_IRT_c55058	110	CCO15181.1	0.0	-	73.00	38.51	34
IRC_IRT_c55062	111	EEC83290.1	0.0	GO:0008137	86.00	63.54	36
IRC_IRT_c55072	137	XP_001701446.1	0.8	-	56.00	32.73	48
IRC_IRT_c55078	111	EAZ10333.1	0.0	-	97.00	77.80	34
IRC_IRT_c55111	109	CCO66671.1	0.0	GO:0000166	74.00	41.59	35
IRC_IRT_c55174	112	EAY85554.1	0.0	-	100.00	77.03	37
IRC_IRT_c55195	124	KFM27674.1	0.0	-	61.00	36.19	44
IRC_IRT_c55203	115	XP_011016603.1	0.0	-	71.00	42.74	38
IRC_IRT_c55298	105	XP_002521025.1	0.0	-	65.00	36.97	35
IRC_IRT_c55303	124	KDD72044.1	0.0	-	72.00	43.90	37
IRC_IRT_c55315	123	XP_002537827.1	0.0	-	66.00	39.66	42
IRC_IRT_c55348	154	XP_004253348.1	0.0	-	58.00	38.89	48
IRC_IRT_c55362	104	XP_002534842.1	0.0	-	59.00	36.58	37
IRC_IRT_c55390	109	CCO17359.1	0.0	GO:0044699	76.00	48.14	38
IRC_IRT_c55393	103	XP_009630060.1	0.0	GO:0031425	76.00	55.07	34
IRC_IRT_c55396	163	XP_002536291.1	0.0	-	50.00	38.51	52
IRC_IRT_c55467	105	EEC81163.1	0.0	-	73.00	36.19	34
IRC_IRT_c55483	113	EEE65094.1	0.6	-	55.00	32.34	38
IRC_IRT_c55528	110	EAY92129.1	0.0	GO:0009536	94.00	72.40	34
IRC_IRT_c55547	109	XP_002948784.1	0.0	GO:0008270	74.00	47.37	35
IRC_IRT_c55582	226	BAD81346.1	0.0	GO:0019538	75.00	59.31	41
IRC_IRT_c55614	118	NP_001173826.1	0.0	-	71.00	34.65	39
IRC_IRT_c55670	119	CAE04518.2	0.0	GO:0005739	84.00	59.69	39
IRC_IRT_c55682	123	XP_002538508.1	0.0	-	64.00	36.19	34
IRC_IRT_c55696	102	XP_006653608.1	0.0	-	97.00	67.40	34
IRC_IRT_c55720	134	BAD13198.1	0.0	GO:0055114	100.00	78.18	35
IRC_IRT_c55727	109	BAD37594.1	0.0	-	71.00	35.81	38
IRC_IRT_c55742	148	XP_002538359.1	0.1	-	58.00	35.42	43

IRC_IRT_c55785	156	XP_007204801.1	0.2	-	58.00	32.73	41
IRC_IRT_c55837	110	EEC70646.1	0.0	GO:0030247	80.00	58.92	36
IRC_IRT_c55883	128	XP_002973102.1	0.0	GO:0010048	84.00	52.76	39
IRC_IRT_c55886	104	NP_001043134.1	0.0	GO:0009654	100.00	76.26	34
IRC_IRT_c55895	125	NP_001060895.1	0.0	GO:0003677	97.00	91.28	4.10E+01
IRC_IRT_c55957	113	CBI29517.3	0.0	GO:0005524	97.00	72.79	36
IRC_IRT_c55974	118	CDY24518.1	0.0	GO:0006813	66.00	42.74	36
IRC_IRT_c56018	113	EAY99120.1	0.1	-	100.00	33.50	34
IRC_IRT_c56037	101	XP_003561775.1	0.1	-	76.00	35.04	34
IRC_IRT_c56046	107	EDQ49152.1	0.0	-	76.00	38.89	34
IRC_IRT_c56099	116	ACJ85812.1	0.0	GO:0044444	68.00	45.05	38
IRC_IRT_c56217	130	BAJ93554.1	0.0	GO:0090305	67.00	47.37	34
IRC_IRT_c56218	130	BAD68601.1	0.0	-	90.00	73.17	42
IRC_IRT_c56244	120	KDD75036.1	0.0	GO:0051213	73.00	58.15	38
IRC_IRT_c56252	119	EAZ05444.1	0.0	GO:0016023	100.00	82.03	38
IRC_IRT_c56307	116	XP_002538492.1	0.0	-	61.00	45.44	36
IRC_IRT_c56316	111	KGN43777.1	0.0	GO:0004028	81.00	65.08	37
IRC_IRT_c56354	117	XP_001760128.1	0.4	-	52.00	33.50	36
IRC_IRT_c56410	134	XP_001692459.1	0.0	-	71.00	36.58	42
IRC_IRT_c56428	157	EEC75666.1	0.0	GO:0032508	82.00	59.31	39
IRC_IRT_c56495	108	EEC77229.1	0.0	GO:0008234	97.00	75.87	35
IRC_IRT_c56531	164	XP_011016603.1	0.0	GO:0030976	96.00	99.75	52
IRC_IRT_c56547	105	XP_002536181.1	0.4	-	65.00	32.34	35
IRC_IRT_c56567	134	KFM29074.1	0.0	GO:0000166	72.00	59.69	44
IRC_IRT_c56597	108	XP_001774110.1	0.1	-	61.00	33.50	36
IRC_IRT_c56600	104	XP_005644633.1	0.0	GO:0050660	85.00	57.00	34
IRC_IRT_c56790	114	BAD73826.1	0.0	-	94.00	70.09	36
IRC_IRT_c56816	131	ACN40771.1	0.0	-	73.00	40.82	42
IRC_IRT_c56832	112	CAH66046.1	0.0	GO:0003676	100.00	79.34	37
IRC_IRT_c56833	223	YP_588280.1	0.0	-	77.00	46.60	35
IRC_IRT_c56842	178	XP_003588267.1	0.0	-	64.00	59.69	51
IRC_IRT_c56875	106	XP_003078364.1	0.0	GO:0042558	88.00	54.30	34
IRC_IRT_c56877	119	BAJ99459.1	0.0	GO:0008152	75.00	48.52	36

IRC_IRT_c56942	109	XP_011014723.1	0.0	-	94.00	66.63	36
IRC_IRT_c56944	178	ABA98294.1	0.0	GO:0004523	91.00	105.92	59
IRC_IRT_c56958	120	XP_005649238.1	0.0	GO:0016787	78.00	41.59	37
IRC_IRT_c57002	164	EEC69063.1	0.0	-	84.00	76.26	46
IRC_IRT_c57011	119	ACX71633.1	0.0	GO:0043167	76.00	54.68	39
IRC_IRT_c57026	155	XP_006340563.1	0.0	-	51.00	41.59	54
IRC_IRT_c57042	143	NP_001056829.1	0.0	GO:0006468	90.00	83.57	43
IRC_IRT_c57113	122	XP_002451762.1	0.0	-	63.00	40.82	38
IRC_IRT_c57160	120	XP_001420574.1	0.0	GO:0016671	65.00	46.21	40
IRC_IRT_c57214	103	XP_008775163.1	0.0	-	85.00	48.91	34
IRC_IRT_c57230	164	ABA98374.1	0.0	GO:0003676	96.00	105.15	54
IRC_IRT_c57242	185	XP_002457347.1	0.0	-	48.00	39.66	58
IRC_IRT_c57304	158	AAU90089.1	0.0	GO:0003677	96.00	103.22	52
IRC_IRT_c57450	104	CAE04750.3	0.0	-	67.00	40.05	34
IRC_IRT_c57474	148	XP_001786505.1	0.0	GO:0003824	69.00	43.51	39
IRC_IRT_c57490	150	BAD69364.1	0.0	GO:0003676	91.00	70.86	45
IRC_IRT_c57509	134	AAL93056.1	0.0	GO:0003899	95.00	87.04	4.00E+01
IRC_IRT_c57524	123	KDD73118.1	0.0	GO:0009630	82.00	67.40	39
IRC_IRT_c57527	108	XP_003079205.1	0.0	-	72.00	43.90	36
IRC_IRT_c57531	136	EEC76917.1	0.0	GO:0003677	90.00	75.10	44
IRC_IRT_c57543	111	EAY90106.1	0.0	-	94.00	38.89	34
IRC_IRT_c57545	124	XP_002868768.1	0.0	-	67.00	41.20	37
IRC_IRT_c57598	103	BAB64652.1	0.3	-	52.00	33.88	42
IRC_IRT_c57622	115	AAX95377.1	0.0	GO:0003964	100.00	82.03	3.80E+01
IRC_IRT_c57655	135	Q9G4F5.1	0.0	GO:0016020	78.00	51.22	38
IRC_IRT_c57661	112	BAD15526.1	0.0	GO:0016023	100.00	83.96	3.70E+01
IRC_IRT_c57684	107	EEE52320.1	0.0	GO:0055085	100.00	71.63	35
IRC_IRT_c57686	108	XP_011016736.1	0.0	GO:0003824	82.00	57.38	34
IRC_IRT_c57728	142	Q9M4W3.1	0.0	-	58.00	39.28	36
IRC_IRT_c57751	109	KDO48305.1	0.0	GO:0006007	86.00	51.60	36
IRC_IRT_c57815	161	XP_006339245.1	1.0	-	59.00	32.34	37
IRC_IRT_c57837	112	BAJ98151.1	0.0	GO:0006526	80.00	45.82	36
IRC_IRT_c57841	148	XP_002462376.1	0.0	GO:0005840	97.00	97.44	48

IRC_IRT_c57857	128	CAD39387.2	0.0	GO:0003676	100.00	83.57	42
IRC_IRT_c57902	111	XP_004135582.1	0.0	GO:0010162	80.00	51.60	36
IRC_IRT_c57914	121	XP_008793515.1	0.4	-	61.00	33.50	36
IRC_IRT_c57915	110	XP_001702462.1	0.5	-	70.00	33.11	34
IRC_IRT_c57916	270	XP_003608262.1	0.0	-	46.00	37.74	58
IRC_IRT_c57917	179	XP_003623596.1	0.1	-	54.00	35.04	44
IRC_IRT_c57949	115	YP_665677.1	0.0	GO:0046961	86.00	55.45	37
IRC_IRT_c57950	131	CBI32677.3	0.9	-	58.00	31.96	41
IRC_IRT_c57960	138	XP_002539885.1	0.0	GO:0016740	71.00	40.82	38
IRC_IRT_c57982	115	YP_514656.1	0.0	GO:0005739	100.00	79.72	37
IRC_IRT_c57992	200	EEE52778.1	0.0	GO:0097503	86.00	96.29	51
IRC_IRT_c58029	112	EEC84861.1	0.0	GO:0004739	100.00	76.64	36
IRC_IRT_c58121	177	EMT03129.1	0.8	-	59.00	33.11	37
IRC_IRT_c58125	135	XP_001770207.1	0.0	GO:0009295	87.00	71.25	40
IRC_IRT_c58130	118	XP_006352701.1	0.2	-	58.00	33.50	34
IRC_IRT_c58189	141	EEE68528.1	0.0	GO:0044763	73.00	65.86	46
IRC_IRT_c58209	109	XP_002955373.1	0.0	-	72.00	42.74	36
IRC_IRT_c58266	112	XP_009350063.1	0.0	GO:0003735	88.00	63.93	36
IRC_IRT_c58289	144	XP_002518536.1	0.6	-	58.00	33.11	39
IRC_IRT_c58342	143	EMS64240.1	0.0	-	53.00	38.12	45
IRC_IRT_c58381	130	XP_002538205.1	0.0	-	61.00	39.28	34
IRC_IRT_c58402	135	XP_002539363.1	0.0	GO:0097159	78.00	46.21	38
IRC_IRT_c58440	164	KEH15169.1	0.0	-	65.00	39.66	35
IRC_IRT_c58599	111	XP_002953210.1	0.0	GO:0000160	79.00	50.83	34
IRC_IRT_c58681	121	XP_010278965.1	0.0	GO:0031425	85.00	54.30	35
IRC_IRT_c58766	104	CEF99626.1	0.0	-	70.00	36.58	34
IRC_IRT_c58767	136	XP_002535762.1	0.7	-	57.00	32.34	38
IRC_IRT_c58792	129	XP_002536674.1	0.0	GO:0043565	79.00	54.68	39
IRC_IRT_c58808	104	EEC71028.1	0.0	-	97.00	75.87	34
IRC_IRT_c58831	181	XP_003558154.1	0.0	GO:0006470	60.00	42.74	55
IRC_IRT_c58833	133	ABA98373.1	0.0	GO:0003964	97.00	90.89	43
IRC_IRT_c58857	127	XP_002540253.1	0.0	GO:0046872	90.00	68.55	40
IRC_IRT_c58867	115	EEC70976.1	0.0	GO:0070933	100.00	80.88	38

IRC_IRT_c58873	106	AAK11581.1	0.0	GO:0009944	100.00	76.64	34
IRC_IRT_c58923	121	KIZ00427.1	0.8	-	67.00	32.73	34
IRC_IRT_c58942	109	EAY98848.1	0.0	-	100.00	82.03	36
IRC_IRT_c58946	111	KEH15495.1	0.0	GO:0050660	89.00	65.08	37
IRC_IRT_c58982	120	ABA01126.1	0.0	GO:0016620	71.00	43.90	39
IRC_IRT_c59009	125	AAV24919.1	0.0	GO:0004523	83.00	65.08	36
IRC_IRT_c59105	154	BAD53302.1	0.4	-	64.00	33.11	37
IRC_IRT_c59124	108	XP_002536141.1	0.0	GO:0004872	86.00	51.22	36
IRC_IRT_c59162	114	KFM27617.1	0.0	-	76.00	48.14	34
IRC_IRT_c59168	154	XP_002536230.1	0.0	-	63.00	37.35	36
IRC_IRT_c59205	146	AFG54450.1	0.0	GO:0044699	65.00	40.43	38
IRC_IRT_c59263	103	EEE50838.1	0.0	GO:0005739	100.00	76.64	34
IRC_IRT_c59278	102	BAD35952.1	0.0	-	70.00	35.81	34
IRC_IRT_c59293	112	ACN25343.1	0.3	-	56.00	33.11	37
IRC_IRT_c59337	161	BAD53714.1	0.0	GO:0006952	98.00	105.53	53
IRC_IRT_c59350	128	XP_002536043.1	0.0	-	66.00	44.28	42
IRC_IRT_c59356	126	XP_007014820.1	0.8	-	59.00	32.73	42
IRC_IRT_c59364	122	KEH15604.1	0.0	-	80.00	70.09	40
IRC_IRT_c59468	120	XP_002539337.1	0.0	-	67.00	43.51	37
IRC_IRT_c59476	141	XP_008661772.1	0.0	-	78.00	54.68	3.80E+01
IRC_IRT_c59556	112	KDD75252.1	0.0	-	76.00	39.66	34
IRC_IRT_c59561	138	AAQ56570.1	0.0	GO:0003676	97.00	90.51	45
IRC_IRT_c59562	105	CBI25333.3	0.7	-	52.00	32.34	34
IRC_IRT_c59576	161	XP_002537161.1	0.0	GO:1902358	79.00	74.71	53
IRC_IRT_c59597	142	BAA83439.1	0.0	-	73.00	37.74	34
IRC_IRT_c59603	102	AAO73227.1	0.0	GO:0003677	100.00	77.80	34
IRC_IRT_c59616	120	XP_006650213.1	0.0	GO:0019538	74.00	50.45	39
IRC_IRT_c59641	102	XP_002954135.1	0.6	-	70.00	32.73	34
IRC_IRT_c59662	104	EAY86974.1	0.0	GO:0006450	94.00	66.63	34
IRC_IRT_c59669	150	XP_006661899.1	1.0	-	50.00	32.34	42
IRC_IRT_c59679	108	CEG02197.1	0.0	-	68.00	40.05	35
IRC_IRT_c59680	125	XP_002283154.1	0.7	-	55.00	32.73	34
IRC_IRT_c59704	112	EAY80367.1	0.0	GO:0009536	97.00	70.48	36

IRC_IRT_c59801	136	EAY72671.1	0.0	-	100.00	98.21	45
IRC_IRT_c59813	116	EEC70905.1	0.0	GO:0044763	78.00	56.23	37
IRC_IRT_c59842	115	XP_002539661.1	0.0	-	92.00	66.63	38
IRC_IRT_c59866	125	CAE02394.2	0.0	GO:0006508	92.00	61.62	38
IRC_IRT_c59876	102	EAZ28843.1	0.0	GO:0016023	100.00	69.71	34
IRC_IRT_c59891	112	XP_006839073.1	0.0	GO:0016462	88.00	61.62	36
IRC_IRT_c59924	161	BAJ88866.1	0.0	GO:0017111	90.00	62.77	40
IRC_IRT_c59929	187	XP_003623596.1	0.0	GO:0009536	67.00	51.60	52
IRC_IRT_c59954	323	KJB31094.1	0.0	-	64.00	40.82	37
IRC_IRT_c59982	112	XP_001787073.1	0.0	-	66.00	42.74	36
IRC_IRT_c59993	106	XP_002540432.1	0.0	-	74.00	42.36	35
IRC_IRT_c60012	141	EEC77821.1	0.0	GO:0006355	90.00	73.56	40
IRC_IRT_c60060	127	KDD76926.1	0.0	-	73.00	53.53	34
IRC_IRT_c60065	144	XP_002539781.1	0.1	-	67.00	33.11	37
IRC_IRT_c60066	125	XP_001696722.1	0.0	-	68.00	36.58	41
IRC_IRT_c60069	129	XP_005850548.1	0.7	-	56.00	32.73	44
IRC_IRT_c60071	131	YP_008816069.1	0.0	GO:0006810	65.00	47.75	43
IRC_IRT_c60090	206	XP_002537322.1	0.0	GO:0005488	63.00	71.25	65
IRC_IRT_c60104	148	CDM82334.1	0.0	-	69.00	41.59	36
IRC_IRT_c60131	116	XP_002539363.1	0.0	GO:0000166	84.00	55.84	38
IRC_IRT_c60136	205	XP_004512278.1	1.0	-	57.00	32.73	35
IRC_IRT_c60153	174	EXB63585.1	0.0	GO:0044444	68.00	60.46	51
IRC_IRT_c60154	140	YP_003058290.1	0.0	GO:0003899	81.00	60.46	38
IRC_IRT_c60157	140	XP_007039042.1	0.9	-	65.00	32.73	40
IRC_IRT_c60162	147	EPS70026.1	0.0	-	68.00	38.51	38
IRC_IRT_c60169	134	CDX69096.1	0.5	-	51.00	33.11	45
IRC_IRT_c60222	104	EDQ48526.1	0.0	GO:0019867	79.00	50.06	34
IRC_IRT_c60251	172	YP_005089854.1	0.1	-	56.00	35.42	46
IRC_IRT_c60252	164	BAD68545.1	0.0	GO:0016023	92.00	68.17	38
IRC_IRT_c60262	189	EEC76877.1	0.0	GO:0046034	77.00	53.53	35
IRC_IRT_c60266	197	EPS68588.1	0.5	-	57.00	34.27	61
IRC_IRT_c60298	104	KEH15262.1	0.0	-	67.00	41.59	34
IRC_IRT_c60308	340	EPS74494.1	0.0	-	75.00	57.00	40

IRC_IRT_c60318	113	KIY94521.1	0.0	-	69.00	40.05	36
IRC_IRT_c60327	231	EPS74494.1	0.0	-	65.00	42.36	49
IRC_IRT_c60362	119	XP_008386655.1	0.6	-	64.00	33.11	34
IRC_IRT_c60363	111	EEC78702.1	0.0	GO:0006812	80.00	52.76	36
IRC_IRT_c60383	132	Q84ZM7.1	0.0	-	77.00	37.74	35
IRC_IRT_c60387	119	CAA64808.1	0.0	GO:0009908	87.00	68.94	39
IRC_IRT_c60415	130	XP_001786082.1	0.2	-	61.00	34.65	39
IRC_IRT_c60420	113	A2YMR0.1	0.0	GO:0006355	100.00	46.21	35
IRC_IRT_c60445	114	XP_005651828.1	0.0	-	58.00	35.81	34
IRC_IRT_c60472	114	XP_002537871.1	0.3	-	55.00	33.50	34
IRC_IRT_c60479	108	AAG13493.1	0.0	GO:0001666	85.00	59.69	35
IRC_IRT_c60536	125	CAH68037.1	0.0	GO:0008152	100.00	87.04	40
IRC_IRT_c60572	179	XP_008365638.1	0.0	-	55.00	35.81	45
IRC_IRT_c60581	123	XP_002537955.1	0.0	GO:0016798	82.00	60.85	40
IRC_IRT_c60587	137	XP_004150242.1	0.9	-	53.00	32.34	45
IRC_IRT_c60593	131	XP_011039179.1	0.5	-	71.00	32.73	35
IRC_IRT_c60619	193	KDP21196.1	0.6	-	50.00	33.50	52
IRC_IRT_c60628	150	EYU34372.1	0.0	GO:0044444	67.00	47.37	37
IRC_IRT_c60631	138	XP_004500214.1	0.5	-	54.00	33.11	44
IRC_IRT_c60639	128	XP_010493474.1	0.3	-	67.00	32.73	37
IRC_IRT_c60644	178	ADK60808.1	0.0	-	70.00	43.13	34
IRC_IRT_c60651	164	XP_003082559.1	0.0	-	59.00	41.59	42
IRC_IRT_c60674	129	XP_002539710.1	0.0	-	62.00	44.67	43
IRC_IRT_c60700	132	EAZ23864.1	0.0	GO:0030247	100.00	82.80	35
IRC_IRT_c60732	253	NP_038366.1	0.0	GO:0003735	79.00	79.72	63
IRC_IRT_c60771	108	NP_001142293.1	0.1	-	83.00	35.42	36
IRC_IRT_c60878	124	EEC66806.1	0.0	GO:0050660	100.00	89.35	41
IRC_IRT_c60936	126	AIK66602.1	0.0	-	69.00	43.90	42
IRC_IRT_c61022	118	AAX96575.1	0.9	-	61.00	32.34	36
IRC_IRT_c61065	105	XP_002540070.1	0.0	-	73.00	45.44	34
IRC_IRT_c61120	110	XP_010030571.1	1.0	-	51.00	31.19	37
IRC_IRT_c61123	110	XP_007214652.1	0.7	-	65.00	32.34	35
IRC_IRT_c61146	119	EAY97006.1	0.0	-	79.00	55.45	39

IRC_IRT_c61147	140	KEH15984.1	0.3	-	70.00	34.27	37
IRC_IRT_c61246	138	NP_042247.1	0.0	GO:0016021	88.00	51.60	35
IRC_IRT_c61260	141	XP_003056183.1	0.0	-	65.00	40.82	44
IRC_IRT_c61270	163	ABA98827.2	0.0	GO:0006952	100.00	117.86	54
IRC_IRT_c61287	113	NP_001050308.2	0.0	GO:0008270	97.00	84.73	37
IRC_IRT_c61300	107	XP_002538951.1	0.0	-	76.00	46.60	34
IRC_IRT_c61301	161	XP_008373874.1	0.4	-	59.00	33.88	37
IRC_IRT_c61310	114	XP_006293997.1	0.0	GO:0010581	76.00	47.75	34
IRC_IRT_c61356	107	BAD28700.1	0.0	-	100.00	72.02	35
IRC_IRT_c61370	101	XP_005651836.1	0.0	-	58.00	36.19	36
IRC_IRT_c61383	135	XP_004253349.1	0.0	-	71.00	55.45	45
IRC_IRT_c61438	133	CAH66262.1	0.0	GO:0003676	100.00	95.90	42
IRC_IRT_c61448	148	XP_002535671.1	0.0	GO:0004333	93.00	64.70	49
IRC_IRT_c61449	114	EAY89554.1	0.0	GO:0000280	97.00	82.42	38
IRC_IRT_c61523	132	YP_009105186.1	0.0	-	64.00	38.89	39
IRC_IRT_c61525	101	EEC77288.1	0.0	GO:0000139	85.00	54.30	34
IRC_IRT_c61600	118	XP_001697267.1	0.0	-	69.00	36.58	36
IRC_IRT_c61601	116	KJB13843.1	0.0	-	66.00	36.58	39
IRC_IRT_c61607	107	KFK44985.1	0.0	GO:0008152	79.00	39.66	34
IRC_IRT_c61612	216	XP_006854192.2	0.5	-	47.00	34.27	53
IRC_IRT_c61673	103	XP_002539567.1	0.0	-	76.00	38.12	34
IRC_IRT_c61677	105	XP_003059457.1	0.0	-	70.00	40.43	34
IRC_IRT_c61709	130	XP_002959807.1	0.1	-	56.00	35.04	48
IRC_IRT_c61742	129	NP_001046220.1	0.0	GO:0004553	100.00	90.89	43
IRC_IRT_c61753	130	KJB72579.1	0.0	GO:0006468	100.00	95.90	41
IRC_IRT_c61769	135	XP_001754837.1	0.0	GO:0003824	72.00	45.44	44
IRC_IRT_c61852	179	XP_010478618.1	0.2	-	56.00	34.65	51
IRC_IRT_c61888	154	AAX95069.1	0.0	GO:0016023	70.00	62.00	41
IRC_IRT_c61930	105	BAD31338.1	0.0	-	70.00	45.82	34
IRC_IRT_c61950	111	NP_001055734.1	0.0	-	94.00	43.90	36
IRC_IRT_c61981	109	KFM28122.1	0.0	-	69.00	40.43	36
IRC_IRT_c62012	173	XP_002499889.1	0.0	GO:0005737	83.00	71.25	48
IRC_IRT_c62132	107	EAY83408.1	0.0	GO:0016023	100.00	75.49	35

IRC_IRT_c62155	119	XP_004301263.1	0.0	GO:0044763	77.00	52.76	36
IRC_IRT_c62163	260	NP_001172304.1	0.0	GO:0071704	76.00	63.54	47
IRC_IRT_c62190	155	XP_006589634.1	0.0	-	56.00	35.42	51
IRC_IRT_c62228	109	EAZ01267.1	0.0	GO:0005634	100.00	75.49	35
IRC_IRT_c62251	264	XP_007154367.1	0.0	-	63.00	53.14	55
IRC_IRT_c62257	107	XP_002500524.1	0.0	GO:0046872	79.00	50.83	34
IRC_IRT_c62279	108	XP_011016757.1	0.0	GO:0044763	88.00	56.61	36
IRC_IRT_c62300	132	CCO18705.1	0.6	-	65.00	32.73	35
IRC_IRT_c62324	121	EYU35930.1	0.0	-	52.00	33.88	36
IRC_IRT_c62328	128	XP_002538284.1	0.0	-	68.00	38.51	45
IRC_IRT_c62333	142	XP_011016696.1	0.0	-	63.00	37.35	49
IRC_IRT_c62397	105	XP_002535695.1	0.0	-	77.00	45.82	35
IRC_IRT_c62447	122	XP_002953210.1	0.0	GO:0009536	67.00	48.52	40
IRC_IRT_c62452	110	BAD16516.1	0.0	-	62.00	37.35	35
IRC_IRT_c62454	117	XP_003080652.1	0.0	-	68.00	41.59	38
IRC_IRT_c62473	202	XP_002534193.1	0.0	GO:0006355	94.00	89.35	51
IRC_IRT_c62520	144	XP_007028519.1	0.0	GO:0071266	82.00	44.67	34
IRC_IRT_c62558	129	XP_002535972.1	0.0	GO:0005975	95.00	78.57	42
IRC_IRT_c62612	231	XP_003623596.1	0.0	-	60.00	36.58	45
IRC_IRT_c62616	131	KEH43504.1	0.7	-	50.00	30.42	42
IRC_IRT_c62702	155	CDO98669.1	0.0	GO:0046914	74.00	52.37	35
IRC_IRT_c62718	111	CEF96515.1	0.0	GO:0000166	75.00	48.91	36
IRC_IRT_c62730	138	NP_001061498.1	0.0	-	61.00	37.74	36
IRC_IRT_c62747	114	NP_001173743.1	0.0	-	100.00	77.41	37
IRC_IRT_c62767	231	AFK45797.1	0.9	-	56.00	31.19	41
IRC_IRT_c62786	140	ABA98483.1	0.0	GO:0003676	91.00	69.71	37
IRC_IRT_c62799	138	XP_002488904.1	0.0	GO:0046872	85.00	54.68	41
IRC_IRT_c62807	154	EDQ48479.1	0.0	-	62.00	49.29	53
IRC_IRT_c62809	159	KEH17027.1	0.0	-	57.00	39.66	40
IRC_IRT_c62855	113	XP_006651953.1	0.0	-	61.00	42.74	34
IRC_IRT_c62856	336	EPS74531.1	0.0	-	69.00	49.68	46
IRC_IRT_c62860	181	XP_006664171.1	0.0	-	64.00	45.82	50
IRC_IRT_c62882	109	XP_002443617.1	0.0	-	65.00	41.20	35

IRC_IRT_c62895	122	XP_009351110.1	0.0	-	74.00	36.97	35
IRC_IRT_c62911	111	XP_004300356.1	0.0	GO:0044763	74.00	47.37	35
IRC_IRT_c62927	145	KDD76057.1	0.7	-	64.00	32.73	34
IRC_IRT_c62932	126	XP_010497830.1	0.6	-	54.00	30.42	35
IRC_IRT_c62949	117	KIZ00619.1	0.0	GO:0016846	74.00	53.14	39
IRC_IRT_c62988	207	AGS48265.1	0.0	GO:0003746	90.00	72.79	41
IRC_IRT_c62990	115	NP_001190073.1	0.0	GO:0006007	83.00	65.47	37
IRC_IRT_c62998	127	XP_009351797.1	0.3	-	50.00	33.88	42
IRC_IRT_c63030	133	XP_006405912.1	0.0	-	67.00	37.74	34
IRC_IRT_c63033	231	XP_011016603.1	0.0	-	91.00	90.89	47
IRC_IRT_c63037	204	XP_002540262.1	0.0	-	55.00	36.19	52
IRC_IRT_c63044	123	XP_009350067.1	0.0	-	79.00	57.38	39
IRC_IRT_c63083	117	XP_005644833.1	0.0	-	63.00	36.19	38
IRC_IRT_c63144	146	CDK13032.1	0.5	-	58.00	33.50	34
IRC_IRT_c63152	109	XP_002535351.1	0.0	-	73.00	36.19	34
IRC_IRT_c63193	115	XP_002537008.1	0.0	-	82.00	53.14	35
IRC_IRT_c63202	193	CDY45505.1	0.0	-	62.00	43.51	48
IRC_IRT_c63217	108	AAP44611.1	0.0	GO:0009536	100.00	74.71	35
IRC_IRT_c63307	128	XP_002956902.1	0.4	-	66.00	33.11	39
IRC_IRT_c63321	137	NP_194116.1	0.2	-	61.00	35.04	34
IRC_IRT_c63332	142	CDY53530.1	0.1	-	53.00	35.81	39
IRC_IRT_c63430	103	EPS66189.1	0.0	GO:0046872	88.00	46.98	34
IRC_IRT_c63442	267	EPS74531.1	0.0	-	72.00	42.74	37
IRC_IRT_c63449	123	KDP27082.1	0.3	-	57.00	33.88	35
IRC_IRT_c63465	107	EDQ48403.1	0.0	-	67.00	43.90	34
IRC_IRT_c63514	160	XP_004291058.1	0.0	-	75.00	39.28	36
IRC_IRT_c63535	143	BAA75236.1	0.0	GO:0003676	89.00	90.89	47
IRC_IRT_c63577	125	XP_002986980.1	0.0	GO:0016491	71.00	47.37	39
IRC_IRT_c63589	109	XP_001763219.1	0.4	-	61.00	33.11	36
IRC_IRT_c63607	250	XP_003588355.1	0.0	-	65.00	49.68	46
IRC_IRT_c63623	139	AAU43927.1	0.0	GO:0004523	78.00	70.86	46
IRC_IRT_c63645	125	CEG01981.1	0.0	-	73.00	54.30	34
IRC_IRT_c63671	107	CBI30132.3	0.0	-	65.00	37.74	35

IRC_IRT_c63768	108	XP_001786556.1	0.0	GO:0005488	70.00	42.74	34
IRC_IRT_c63786	122	XP_008356367.1	0.0	-	75.00	37.35	37
IRC_IRT_c63798	105	AAS07367.1	0.1	-	51.00	35.42	35
IRC_IRT_c63808	124	XP_002954981.1	0.0	GO:0004553	68.00	46.60	35
IRC_IRT_c63866	146	BAD10629.1	0.4	-	59.00	33.50	49
IRC_IRT_c63887	124	BAD45224.1	0.0	-	100.00	36.58	34
IRC_IRT_c63925	138	EAZ28054.1	0.0	GO:0015691	76.00	62.00	42
IRC_IRT_c63970	123	XP_002537104.1	0.0	GO:0008784	100.00	85.11	40
IRC_IRT_c64003	103	AAV31236.1	0.0	-	100.00	73.56	34
IRC_IRT_c64041	119	EEC76774.1	0.0	-	70.00	37.35	37
IRC_IRT_c64053	109	XP_002948464.1	0.2	-	64.00	34.27	34
IRC_IRT_c64055	131	XP_002536219.1	0.0	GO:0006935	88.00	55.07	34
IRC_IRT_c64057	146	XP_004253349.1	0.0	-	65.00	55.45	47
IRC_IRT_c64060	105	EEC79596.1	0.0	GO:0005488	68.00	42.74	35
IRC_IRT_c64067	103	BAB40070.1	0.0	-	100.00	71.25	34
IRC_IRT_c64084	132	AAO73287.1	0.0	GO:0008270	100.00	106.30	44
IRC_IRT_c64094	115	EPS68183.1	0.7	-	61.00	32.34	36
IRC_IRT_c64119	147	KIZ01899.1	0.0	GO:0015930	69.00	55.84	52
IRC_IRT_c64207	166	XP_006648849.1	0.0	-	60.00	57.77	51
IRC_IRT_c64225	115	EEE65914.1	0.0	GO:0005975	100.00	76.64	37
IRC_IRT_c64255	156	XP_002540078.1	0.0	-	74.00	40.82	35
IRC_IRT_c64300	113	XP_002535078.1	0.0	-	88.00	65.86	36
IRC_IRT_c64352	118	EEC68548.1	0.0	-	64.00	36.97	39
IRC_IRT_c64445	142	XP_007201174.1	0.0	GO:0004658	89.00	87.04	47
IRC_IRT_c64488	133	EAY85617.1	0.0	GO:0044249	100.00	81.65	38
IRC_IRT_c64493	106	XP_001416617.1	0.0	-	77.00	39.28	36
IRC_IRT_c64500	116	AAX95489.1	0.0	-	100.00	91.66	38
IRC_IRT_c64524	137	XP_005648462.1	0.0	-	64.00	50.83	45
IRC_IRT_c64544	128	EHK62748.1	0.0	-	80.00	45.44	35
IRC_IRT_c64561	114	XP_008245816.1	0.1	-	60.00	34.65	35
IRC_IRT_c64607	124	XP_002505504.1	0.8	-	59.00	32.73	44
IRC_IRT_c64616	118	KFM26016.1	0.0	GO:0035434	81.00	47.75	38
IRC_IRT_c64649	170	XP_006855294.1	0.0	-	58.00	43.13	53

IRC_IRT_c64731	143	KIZ05001.1	0.0	GO:0044763	71.00	60.85	45
IRC_IRT_c64836	109	NP_001062563.2	0.0	GO:0006952	94.00	71.25	36
IRC_IRT_c64841	108	XP_001755583.1	0.0	GO:0050660	80.00	50.06	36
IRC_IRT_c64895	149	AAT44245.1	0.0	GO:0003677	81.00	43.51	48
IRC_IRT_c64919	125	XP_002537783.1	0.0	-	59.00	35.81	37
IRC_IRT_c64950	141	EEE53164.1	0.0	GO:0016023	100.00	78.95	38
IRC_IRT_c64952	116	BAB01176.1	0.1	-	67.00	35.04	34
IRC_IRT_c64957	181	XP_010275250.1	0.0	-	52.00	40.82	53
IRC_IRT_c64992	116	AED89151.1	0.0	-	86.00	36.58	36
IRC_IRT_c65066	121	CDX91834.1	0.0	-	70.00	40.43	37
IRC_IRT_c65098	110	EEC69860.1	0.0	-	61.00	39.28	34
IRC_IRT_c65162	115	XP_002539889.1	0.0	-	68.00	36.97	38
IRC_IRT_c65174	108	XP_010943614.1	0.0	-	68.00	37.35	35
IRC_IRT_c65259	125	ABA97254.1	0.0	-	56.00	43.13	41
IRC_IRT_c65260	129	KJB49294.1	0.0	GO:0003866	85.00	61.62	42
IRC_IRT_c65284	113	AAO41131.1	0.2	-	63.00	34.27	38
IRC_IRT_c65287	114	XP_002488959.1	0.0	-	65.00	40.05	35
IRC_IRT_c65327	135	XP_001756545.1	0.5	-	56.00	31.57	37
IRC_IRT_c65332	154	EPS65342.1	0.2	-	51.00	34.65	49
IRC_IRT_c65353	103	CAH67173.1	0.0	GO:0016023	100.00	70.09	34
IRC_IRT_c65380	118	AAV43870.1	0.0	-	66.00	47.37	39
IRC_IRT_c65409	144	BAD29706.1	0.0	-	100.00	84.73	38
IRC_IRT_c65455	165	CAD40474.2	0.0	GO:0005739	80.00	51.22	35
IRC_IRT_c65494	113	BAD03348.1	0.0	-	75.00	55.84	36
IRC_IRT_c65497	108	XP_001786754.1	0.4	-	75.00	32.73	36
IRC_IRT_c65499	131	XP_002536388.1	0.0	-	60.00	41.97	41
IRC_IRT_c65547	172	XP_010911369.1	0.0	GO:0043167	92.00	100.52	57
IRC_IRT_c65602	107	XP_002538154.1	0.0	GO:0009060	85.00	59.31	35
IRC_IRT_c65611	143	XP_002538908.1	0.0	-	61.00	36.19	47
IRC_IRT_c65636	175	XP_010231779.1	0.2	-	46.00	35.04	64
IRC_IRT_c65648	152	XP_009348170.1	0.0	GO:0006810	68.00	55.84	50
IRC_IRT_c65671	151	XP_002540399.1	0.0	-	63.00	47.37	47
IRC_IRT_c65729	103	BAD03370.1	0.0	-	82.00	52.37	34

IRC_IRT_c65779	105	NP_001236204.1	0.0	-	61.00	36.19	34
IRC_IRT_c65796	102	XP_010229437.1	0.0	GO:0009536	79.00	49.68	34
IRC_IRT_c65805	124	XP_009607810.1	0.6	-	55.00	32.73	34
IRC_IRT_c65818	124	XP_011043712.1	0.0	-	55.00	37.74	40
IRC_IRT_c65857	170	ABJ16394.1	0.7	-	42.00	33.11	52
IRC_IRT_c65912	146	AAX96872.1	0.0	GO:0004523	97.00	68.55	34
IRC_IRT_c65936	102	EEE68692.1	0.0	GO:0016020	97.00	49.29	34
IRC_IRT_c65946	175	XP_005843735.1	0.0	-	55.00	37.74	52
IRC_IRT_c65951	127	XP_002539959.1	0.0	-	62.00	37.74	40
IRC_IRT_c66130	152	AAL58253.1	0.0	GO:0009536	97.00	77.03	35
IRC_IRT_c66158	108	CAD40414.3	0.0	GO:0003964	100.00	80.11	35
IRC_IRT_c66204	105	AAN74951.1	0.2	-	52.00	33.88	34
IRC_IRT_c66223	106	XP_010928601.1	0.0	GO:0008152	71.00	45.05	35
IRC_IRT_c66262	179	XP_008358000.1	1.0	-	52.00	32.73	44
IRC_IRT_c66283	169	XP_007225121.1	0.6	-	42.00	33.50	49
IRC_IRT_c66340	179	CAE05744.1	0.0	-	100.00	90.51	44
IRC_IRT_c66346	140	CAN61156.1	0.6	-	55.00	33.11	40
IRC_IRT_c66365	164	XP_005844383.1	0.0	GO:0009536	65.00	53.91	47
IRC_IRT_c66486	145	XP_002281484.1	0.0	GO:0004834	87.00	63.16	39
IRC_IRT_c66496	135	XP_010497163.1	0.9	-	62.00	32.34	45
IRC_IRT_c66541	109	XP_002984359.1	0.0	GO:0006412	74.00	53.91	35
IRC_IRT_c66553	107	EEC78702.1	0.0	-	73.00	36.58	34
IRC_IRT_c66584	101	XP_002971239.1	0.0	-	72.00	38.51	36
IRC_IRT_c66646	102	EAZ43126.1	0.0	GO:0003700	97.00	68.55	34
IRC_IRT_c66654	130	NP_001060934.2	0.0	GO:0003677	88.00	81.65	42
IRC_IRT_c66680	108	XP_011021440.1	0.6	-	58.00	31.96	36
IRC_IRT_c66682	107	ABA97658.1	0.0	GO:0003964	100.00	75.49	34
IRC_IRT_c66696	134	EXC03121.1	0.5	-	58.00	32.73	36
IRC_IRT_c66759	130	ABA55625.1	0.0	-	60.00	35.81	40
IRC_IRT_c66764	113	BAC83338.1	0.0	-	64.00	40.82	37
IRC_IRT_c66828	168	ABA96071.2	0.0	-	62.00	44.28	51
IRC_IRT_c66893	171	NP_001059426.2	0.0	GO:0048544	97.00	102.45	4.90E+01
IRC_IRT_c66904	112	XP_003063430.1	0.2	-	67.00	33.88	34

IRC_IRT_c66958	103	XP_001701854.1	0.0	GO:0046872	76.00	41.59	34
IRC_IRT_c67003	104	EAY72734.1	0.0	GO:0015035	100.00	76.26	34
IRC_IRT_c67017	106	XP_002536637.1	0.0	-	62.00	39.66	35
IRC_IRT_c67019	109	XP_002535661.1	0.0	-	65.00	36.58	35
IRC_IRT_c67042	113	XP_010026810.1	0.0	-	64.00	46.21	37
IRC_IRT_c67055	134	AAY63549.1	0.0	GO:0005576	100.00	77.03	37
IRC_IRT_c67194	106	EAY92600.1	0.0	GO:0005739	100.00	73.56	35
IRC_IRT_c67195	111	EEE67291.1	0.0	GO:0006810	100.00	78.95	35
IRC_IRT_c67258	148	EEE64036.1	0.0	-	85.00	52.76	35
IRC_IRT_c67287	117	XP_005650831.1	0.0	-	82.00	41.20	39
IRC_IRT_c67342	147	XP_001420863.1	0.0	GO:0042026	79.00	60.85	43
IRC_IRT_c67368	111	EEC81315.1	0.0	GO:0016301	100.00	77.41	36
IRC_IRT_c67385	104	XP_005645853.1	0.0	GO:0003824	67.00	42.36	34
IRC_IRT_c67434	106	XP_002534905.1	0.0	-	77.00	49.29	35
IRC_IRT_c67471	150	XP_002540444.1	0.0	GO:0009536	89.00	75.87	46
IRC_IRT_c67505	117	ABR17747.1	0.0	-	72.00	38.12	36
IRC_IRT_c67555	129	XP_005647813.1	0.0	GO:0036094	78.00	61.23	42
IRC_IRT_c67564	131	XP_004985790.1	0.0	GO:0006007	88.00	65.08	42
IRC_IRT_c67616	141	EEC77465.1	0.0	-	58.00	40.43	36
IRC_IRT_c67640	127	XP_010271971.1	0.0	-	71.00	46.98	35
IRC_IRT_c67647	113	EEE63571.1	0.0	-	85.00	55.07	34
IRC_IRT_c67715	151	EDQ48639.1	0.0	-	84.00	63.93	38
IRC_IRT_c67721	259	XP_011096470.1	0.3	-	56.00	35.04	44
IRC_IRT_c67748	132	XP_002539634.1	0.0	-	62.00	48.14	43
IRC_IRT_c67788	108	XP_001419686.1	0.1	-	71.00	34.27	38
IRC_IRT_c67799	104	XP_006664554.1	0.0	GO:0009734	100.00	71.25	34
IRC_IRT_c67808	109	NP_001042057.2	0.0	GO:0008152	100.00	72.02	36
IRC_IRT_c67851	133	ABA97529.1	0.0	-	97.00	43.13	42
IRC_IRT_c67868	115	NP_001057634.1	0.0	GO:0051536	100.00	78.57	3.80E+01
IRC_IRT_c67886	105	BAG98021.1	0.0	GO:0006355	100.00	75.87	35
IRC_IRT_c67890	115	KIY95899.1	0.5	-	67.00	32.73	37
IRC_IRT_c67905	108	NP_001176966.1	0.0	-	91.00	64.31	35
IRC_IRT_c67940	135	EYU18199.1	0.0	GO:0005488	68.00	49.29	38

IRC_IRT_c67962	136	BAD61987.1	0.0	-	88.00	71.25	42
IRC_IRT_c67981	107	XP_003580320.1	0.0	-	55.00	36.58	34
IRC_IRT_c68002	127	BAD53205.1	0.2	-	61.00	34.65	42
IRC_IRT_c68009	144	EAY77289.1	0.0	GO:0016021	97.00	77.80	38
IRC_IRT_c68033	122	AAK15452.1	0.0	-	77.00	48.52	36
IRC_IRT_c68040	107	CAD40155.1	0.0	GO:0003676	97.00	68.17	34
IRC_IRT_c68057	118	CAE02520.2	0.0	GO:0006310	94.00	78.57	37
IRC_IRT_c68177	131	EEC82972.1	0.0	GO:0043531	100.00	87.04	43
IRC_IRT_c68184	168	XP_010255640.1	0.2	-	46.00	34.65	54
IRC_IRT_c68194	138	EEE59347.1	0.0	GO:0006468	90.00	71.63	40
IRC_IRT_c68196	116	CAD39687.1	0.0	-	83.00	61.62	36
IRC_IRT_c68233	122	XP_011016126.1	0.0	GO:0005525	86.00	67.01	38
IRC_IRT_c68321	154	ADO61796.1	0.0	GO:0005737	100.00	80.49	38
IRC_IRT_c68386	133	XP_005650783.1	0.0	-	47.00	36.97	44
IRC_IRT_c68413	120	XP_002979133.1	0.8	-	55.00	32.34	36
IRC_IRT_c68425	115	XP_006576787.1	0.0	GO:0006537	79.00	60.08	34
IRC_IRT_c68432	124	YP_009105303.1	0.0	-	73.00	44.67	34
IRC_IRT_c68562	185	EMT04070.1	0.1	-	57.00	35.81	47
IRC_IRT_c68644	106	XP_003061336.1	0.1	-	64.00	35.04	34
IRC_IRT_c68659	159	XP_008371852.1	0.0	-	54.00	40.82	53
IRC_IRT_c68686	111	ABA95047.1	0.0	GO:0016747	100.00	77.80	36
IRC_IRT_c68687	136	XP_002536396.1	0.0	GO:0008080	84.00	70.86	44
IRC_IRT_c68784	132	CDY08192.1	0.9	-	52.00	32.73	38
IRC_IRT_c68794	118	XP_002504720.1	0.0	-	58.00	38.89	39
IRC_IRT_c68844	104	AAK52553.1	0.1	-	97.00	34.65	34
IRC_IRT_c68994	104	XP_002536378.1	0.1	-	61.00	35.04	34
IRC_IRT_c69009	104	NP_001048688.1	0.0	GO:0005576	100.00	72.02	34
IRC_IRT_c69038	124	KIZ00619.1	0.0	-	71.00	40.82	42
IRC_IRT_c69106	143	XP_002540139.1	0.0	-	63.00	38.89	46
IRC_IRT_c69113	113	XP_002538605.1	0.0	-	82.00	50.83	34
IRC_IRT_c69182	112	XP_002540575.1	0.0	-	75.00	58.15	37
IRC_IRT_c69247	134	CDY49124.1	0.6	-	57.00	32.73	35
IRC_IRT_c69293	124	EYU31890.1	0.7	-	51.00	32.73	39

IRC_IRT_c69345	149	XP_006407534.1	0.4	-	61.00	33.50	44
IRC_IRT_c69378	110	XP_002537713.1	0.0	-	83.00	57.00	36
IRC_IRT_c69391	110	XP_002536523.1	0.0	-	68.00	42.74	35
IRC_IRT_c69425	106	CDX79785.1	0.0	GO:0005840	70.00	41.97	34
IRC_IRT_c69453	111	EEC66960.1	0.0	GO:0017076	71.00	43.51	35
IRC_IRT_c69468	120	YP_636186.1	0.0	GO:0005840	71.00	47.37	39
IRC_IRT_c69674	110	BAD17336.1	0.0	GO:0005739	88.00	57.38	36
IRC_IRT_c69733	176	KDP35282.1	0.8	-	45.00	33.11	53
IRC_IRT_c69748	108	XP_001767106.1	0.9	-	58.00	31.96	36
IRC_IRT_c69765	103	XP_002538245.1	0.0	GO:0007165	70.00	45.82	34
IRC_IRT_c69850	125	XP_010915426.1	0.1	-	60.00	35.42	41
IRC_IRT_c69901	151	BAB17167.1	0.0	-	58.00	38.51	43
IRC_IRT_c69944	103	EAZ18733.1	0.0	-	100.00	73.17	34
IRC_IRT_c69950	124	XP_010911593.1	0.0	-	53.00	37.74	41
IRC_IRT_c69964	123	XP_002534724.1	0.0	GO:0005737	84.00	63.54	39
IRC_IRT_c70059	119	XP_007134308.1	0.1	-	63.00	35.04	38
IRC_IRT_c70189	137	EEC73824.1	0.0	GO:0010541	100.00	96.67	45
IRC_IRT_c70198	110	XP_002539420.1	0.1	-	67.00	35.04	34
IRC_IRT_c70238	132	KCW84148.1	0.1	-	60.00	33.11	35
IRC_IRT_c70283	176	KGN55456.1	0.0	-	54.00	31.96	37
IRC_IRT_c70374	112	XP_010230621.1	0.0	-	64.00	37.35	37
IRC_IRT_c70450	116	XP_003056817.1	0.0	GO:0006629	86.00	67.40	36
IRC_IRT_c70479	157	KFM23069.1	0.0	-	60.00	40.05	45
IRC_IRT_c70480	113	XP_008665747.1	0.0	GO:0006468	85.00	48.52	35
IRC_IRT_c70507	125	XP_003563083.1	0.0	GO:1903046	65.00	42.74	38
IRC_IRT_c70522	168	XP_002536223.1	0.8	-	57.00	32.73	54
IRC_IRT_c70562	107	XP_005643454.1	0.0	-	76.00	39.66	34
IRC_IRT_c70581	143	XP_002954434.1	0.0	-	72.00	43.90	40
IRC_IRT_c70594	123	ADE77708.1	0.0	-	64.00	40.05	39
IRC_IRT_c70687	273	EPS70023.1	0.0	-	53.00	38.51	52
IRC_IRT_c70726	133	XP_002976830.1	0.0	-	64.00	36.97	34
IRC_IRT_c70734	130	XP_001690220.1	0.0	-	76.00	57.77	43
IRC_IRT_c70771	104	XP_002538396.1	0.4	-	58.00	33.11	34

IRC_IRT_c70773	138	XP_005851032.1	0.0	GO:0046872	69.00	45.82	42
IRC_IRT_c70826	127	Q8S0F0.1	0.0	GO:0016021	100.00	78.57	40
IRC_IRT_c70828	172	XP_003565280.2	0.4	-	53.00	33.50	60
IRC_IRT_c70846	164	XP_008454199.1	0.0	GO:0005829	77.00	75.49	54
IRC_IRT_c71028	150	ABF96298.1	0.0	GO:0003676	88.00	58.92	34
IRC_IRT_c71069	106	XP_002973623.1	0.0	GO:0005739	77.00	45.05	35
IRC_IRT_c71106	110	BAD03117.1	0.0	GO:0016023	100.00	82.03	3.60E+01
IRC_IRT_c71108	190	XP_003635968.1	0.0	GO:0006200	82.00	77.41	62
IRC_IRT_c71137	152	XP_002536013.1	0.0	GO:0005488	63.00	47.75	49
IRC_IRT_c71194	148	XP_001783323.1	0.4	-	51.00	31.96	39
IRC_IRT_c71200	140	AET22416.1	0.0	-	65.00	38.51	40
IRC_IRT_c71201	115	AAT44283.1	0.0	GO:0004523	89.00	64.31	37
IRC_IRT_c71211	106	KEH15491.1	0.0	-	61.00	40.43	34
IRC_IRT_c71212	145	CAE05087.4	0.0	GO:0005739	100.00	82.80	39
IRC_IRT_c71307	117	XP_002503629.1	0.0	-	75.00	36.19	36
IRC_IRT_c71377	113	P49390.1	0.0	GO:0005743	80.00	51.99	35
IRC_IRT_c71433	116	ABA98286.2	0.0	GO:0003964	100.00	79.34	38
IRC_IRT_c71469	117	XP_002540557.1	0.0	-	64.00	35.42	34
IRC_IRT_c71483	107	AAX96628.1	0.0	-	88.00	63.54	35
IRC_IRT_c71495	131	XP_002538691.1	0.0	-	80.00	57.38	40
IRC_IRT_c71544	108	CDP16534.1	0.0	-	64.00	40.05	34
IRC_IRT_c71549	112	XP_002535442.1	0.0	GO:0030254	82.00	52.37	34
IRC_IRT_c71601	131	XP_001690981.1	0.0	GO:0003824	70.00	47.37	44
IRC_IRT_c71611	130	EAY75572.1	0.0	GO:0005739	100.00	85.11	43
IRC_IRT_c71622	126	CAE03536.2	0.0	-	80.00	70.86	4.20E+01
IRC_IRT_c71645	113	XP_002535483.1	0.2	-	60.00	34.27	35
IRC_IRT_c71724	140	XP_002501845.1	0.0	GO:0003723	91.00	75.10	45
IRC_IRT_c71733	150	CAN59721.1	0.7	-	50.00	33.11	54
IRC_IRT_c71738	110	EAZ42372.1	0.0	-	100.00	79.34	36
IRC_IRT_c71774	296	YP_358636.1	0.0	GO:0009536	71.00	52.37	39
IRC_IRT_c71786	151	XP_010025753.1	0.7	-	54.00	32.34	42
IRC_IRT_c71792	157	CDY33074.1	0.0	-	58.00	35.42	39
IRC_IRT_c71841	111	EEE54984.1	0.0	-	64.00	40.82	39

IRC_IRT_c71852	197	XP_003064995.1	0.3	-	53.00	31.96	52
IRC_IRT_c71861	127	CAE04040.2	0.0	-	65.00	44.28	41
IRC_IRT_c71889	108	ACG42605.1	0.9	-	67.00	31.57	34
IRC_IRT_c71917	133	KEH15587.1	0.0	GO:0055114	84.00	66.24	44
IRC_IRT_c71950	123	KFM27595.1	0.1	-	67.00	35.04	40
IRC_IRT_c71990	174	BAJ11784.1	0.0	-	69.00	44.28	36
IRC_IRT_c72009	204	AGV54820.1	0.9	-	58.00	33.11	41
IRC_IRT_c72045	164	XP_008663123.1	0.0	-	60.00	38.51	46
IRC_IRT_c72057	178	KDP39645.1	0.0	-	77.00	39.66	35
IRC_IRT_c72084	171	XP_010046453.1	0.0	GO:0006996	86.00	62.39	43
IRC_IRT_c72091	134	XP_008680659.1	0.3	-	59.00	32.34	37
IRC_IRT_c72118	116	XP_002537739.1	0.0	GO:0000160	72.00	45.44	37
IRC_IRT_c72123	157	XP_003638735.1	0.3	-	58.00	34.27	36
IRC_IRT_c72148	124	XP_002537224.1	0.0	-	97.00	78.57	41
IRC_IRT_c72158	122	EMS47955.1	0.0	GO:0006457	80.00	47.75	35
IRC_IRT_c72162	112	XP_002536964.1	0.0	-	76.00	49.68	34
IRC_IRT_c72165	142	CAH66356.1	0.6	-	56.00	33.50	37
IRC_IRT_c72234	321	EXC34899.1	0.0	-	53.00	40.05	56
IRC_IRT_c72237	120	XP_005846681.1	0.0	-	63.00	44.28	41
IRC_IRT_c72247	127	KFM29176.1	0.4	-	53.00	33.11	39
IRC_IRT_c72283	139	XP_002537405.1	0.0	-	60.00	40.43	43
IRC_IRT_c72288	121	XP_010053425.1	0.0	GO:0005525	86.00	60.08	37
IRC_IRT_c72300	149	XP_009403901.1	0.0	-	66.00	41.20	39
IRC_IRT_c72319	216	XP_011013113.1	0.0	-	66.00	39.28	36
IRC_IRT_c72322	172	XP_006279317.1	0.0	-	60.00	35.04	53
IRC_IRT_c72360	286	XP_008808641.1	0.3	-	45.00	35.42	72
IRC_IRT_c72386	130	EAZ37914.1	0.0	GO:0016023	97.00	65.47	42
IRC_IRT_c72450	202	XP_010035800.1	0.1	-	50.00	36.58	62
IRC_IRT_c72480	163	XP_008338813.1	0.9	-	58.00	32.34	48
IRC_IRT_c72487	146	XP_002537142.1	0.0	-	62.00	45.05	48
IRC_IRT_c72503	147	KIZ07527.1	0.0	-	59.00	37.35	44
IRC_IRT_c72516	176	KJB31094.1	0.0	-	66.00	42.74	36
IRC_IRT_c72521	133	CCO16827.1	0.0	GO:0000166	75.00	50.45	44

IRC_IRT_c72543	180	EEE56285.1	0.0	GO:0009536	73.00	46.98	38
IRC_IRT_c72591	178	XP_007154096.1	0.0	-	67.00	46.21	34
IRC_IRT_c72642	231	XP_003592948.1	0.3	-	67.00	33.88	34
IRC_IRT_c72662	122	EEC77966.1	0.0	GO:0030170	94.00	58.15	34
IRC_IRT_c72737	207	EMT17543.1	0.0	GO:0006355	71.00	76.26	67
IRC_IRT_c72748	189	EPS74494.1	0.0	-	69.00	39.66	36
IRC_IRT_c72752	174	XP_008235401.1	0.7	-	52.00	33.11	48
IRC_IRT_c72794	114	XP_005646550.1	0.1	-	58.00	34.65	34
IRC_IRT_c72797	135	XP_005649925.1	0.0	-	53.00	46.21	45
IRC_IRT_c72837	156	XP_004987327.1	0.0	GO:0016491	100.00	82.80	39
IRC_IRT_c72846	139	CAE05001.2	0.0	GO:0005739	80.00	56.23	40
IRC_IRT_c72870	144	XP_002539620.1	0.0	GO:0003810	86.00	83.57	46
IRC_IRT_c72925	145	KJB09764.1	0.9	-	60.00	30.42	35
IRC_IRT_c72954	117	YP_009054587.1	0.1	-	68.00	35.81	38
IRC_IRT_c72964	116	EAZ22166.1	0.0	GO:0016757	73.00	52.76	38
IRC_IRT_c72994	198	XP_001763726.1	0.1	-	60.00	35.81	46
IRC_IRT_c73012	168	EYU35212.1	0.0	GO:0044444	60.00	46.60	55
IRC_IRT_c73024	112	XP_002535815.1	0.1	-	62.00	35.81	37
IRC_IRT_c73033	121	XP_005851545.1	0.0	-	65.00	40.05	38
IRC_IRT_c73046	110	XP_002534905.1	0.0	-	83.00	50.83	36
IRC_IRT_c73071	103	BAC07057.1	0.0	GO:0006511	79.00	47.75	34
IRC_IRT_c73073	138	YP_009057835.1	0.0	GO:0005840	68.00	52.37	45
IRC_IRT_c73107	204	EXB36957.1	0.0	-	73.00	62.77	46
IRC_IRT_c73189	143	BAK05210.1	0.0	GO:0009825	97.00	76.26	35
IRC_IRT_c73194	113	ADH82119.1	0.6	-	61.00	32.73	34
IRC_IRT_c73243	120	XP_011008596.1	0.5	-	57.00	32.73	38
IRC_IRT_c73278	120	AAX92771.1	0.0	GO:0003676	100.00	80.11	39
IRC_IRT_c73321	137	CCO17359.1	0.0	-	73.00	41.20	34
IRC_IRT_c73392	116	EEC78999.1	0.0	GO:0046872	72.00	50.83	36
IRC_IRT_c73500	137	DAA46640.1	0.0	GO:0030529	77.00	65.86	45
IRC_IRT_c73555	116	BAB07815.1	0.0	-	67.00	44.28	34
IRC_IRT_c73610	109	KDD75324.1	0.0	-	68.00	41.59	35
IRC_IRT_c73615	163	XP_002443133.1	0.0	-	78.00	61.62	38

IRC_IRT_c73674	140	KJB16924.1	0.4	-	55.00	31.96	34
IRC_IRT_c73788	106	EEE69878.1	0.0	-	67.00	43.13	34
IRC_IRT_c73790	141	XP_002536832.1	0.0	-	77.00	64.31	44
IRC_IRT_c73810	116	CAE04695.1	0.0	-	72.00	43.51	37
IRC_IRT_c73856	159	XP_003075257.1	0.0	GO:0006098	75.00	65.08	53
IRC_IRT_c73948	114	KEH15262.1	0.0	GO:0016787	70.00	44.67	34
IRC_IRT_c74004	124	CAH68043.1	0.0	GO:0004523	97.00	73.17	36
IRC_IRT_c74031	175	XP_001786882.1	0.0	GO:0044699	68.00	46.98	41
IRC_IRT_c74047	119	XP_002274997.2	0.7	-	58.00	32.34	36
IRC_IRT_c74108	109	AAY32333.1	0.0	GO:0004672	74.00	41.97	35
IRC_IRT_c74129	109	XP_001694367.1	0.0	-	65.00	37.35	35
IRC_IRT_c74143	116	XP_003063483.1	0.0	GO:0045893	92.00	68.55	38
IRC_IRT_c74145	104	CAH66134.1	0.0	GO:0003676	97.00	70.48	34
IRC_IRT_c74151	112	XP_002954659.1	0.7	-	65.00	32.34	35
IRC_IRT_c74183	122	XP_001780120.1	0.0	-	58.00	40.43	39
IRC_IRT_c74185	127	KIZ05573.1	0.0	-	54.00	37.35	42
IRC_IRT_c74220	104	XP_009415149.1	0.0	-	69.00	41.59	36
IRC_IRT_c74227	113	XP_009417596.1	0.0	-	62.00	37.35	37
IRC_IRT_c74233	129	XP_002504720.1	0.0	-	68.00	36.19	38
IRC_IRT_c74277	128	AAT44283.1	0.0	GO:0004523	97.00	75.87	35
IRC_IRT_c74318	107	XP_009350054.1	0.0	-	85.00	57.77	35
IRC_IRT_c74335	149	BAD34045.1	0.0	GO:0009536	100.00	51.99	46
IRC_IRT_c74376	104	AAQ57264.1	0.0	GO:0006950	82.00	55.07	34
IRC_IRT_c74417	108	BAD94823.1	0.1	-	62.00	34.65	35
IRC_IRT_c74443	111	KDD71279.1	0.0	GO:0005739	83.00	47.75	36
IRC_IRT_c74452	136	AAF16525.1	0.0	GO:0006541	73.00	57.00	45
IRC_IRT_c74471	131	BAD28476.1	0.0	-	59.00	36.19	42
IRC_IRT_c74511	129	EMS65638.1	0.0	GO:0015689	88.00	71.63	42
IRC_IRT_c74543	138	EEC67996.1	0.0	-	100.00	90.89	44
IRC_IRT_c74572	116	XP_001776934.1	0.0	GO:0004585	80.00	54.68	36
IRC_IRT_c74579	103	XP_001759956.1	0.1	-	58.00	34.65	34
IRC_IRT_c74591	139	XP_004294141.2	0.7	-	61.00	32.73	36
IRC_IRT_c74704	126	YP_002860155.1	0.0	GO:0051287	92.00	79.72	40

IRC_IRT_c74721	123	KFM27789.1	0.0	GO:0016866	77.00	51.60	40
IRC_IRT_c74736	110	XP_008777456.1	0.0	-	66.00	38.51	36
IRC_IRT_c74749	152	EEC66675.1	0.0	GO:1901576	78.00	72.40	50
IRC_IRT_c74760	117	ABG66150.1	0.0	GO:0044260	73.00	45.44	38
IRC_IRT_c74836	103	XP_009120972.1	0.0	-	77.00	47.37	35
IRC_IRT_c74873	131	XP_001415810.1	0.0	GO:0008270	89.00	62.39	38
IRC_IRT_c74895	130	EMS45934.1	0.0	GO:0016655	100.00	95.90	43
IRC_IRT_c74944	131	XP_002983522.1	0.0	-	65.00	39.28	46
IRC_IRT_c74990	103	BAD68353.1	0.0	GO:0016023	97.00	67.78	34
IRC_IRT_c75021	110	ABA94261.1	0.2	-	58.00	34.27	36
IRC_IRT_c75090	110	EEC74851.1	0.0	GO:0004553	83.00	61.62	36
IRC_IRT_c75097	126	EAY78216.1	0.0	GO:0044763	73.00	58.92	41
IRC_IRT_c75103	123	ABK55672.1	0.0	GO:0016301	70.00	45.05	41
IRC_IRT_c75323	120	XP_006664149.1	0.0	-	100.00	85.11	39
IRC_IRT_c75331	131	AGU90021.1	0.0	-	59.00	35.04	42
IRC_IRT_c75491	139	ACN54198.1	0.2	-	54.00	34.27	46
IRC_IRT_c75493	135	NP_001170550.1	0.5	-	56.00	31.19	37
IRC_IRT_c75561	116	ABA93737.1	0.0	GO:0003677	100.00	75.87	34
IRC_IRT_c75583	115	NP_001172843.1	0.0	GO:0006355	100.00	52.37	38
IRC_IRT_c75664	153	XP_009396972.1	0.5	-	51.00	33.50	45
IRC_IRT_c75719	142	XP_006649775.1	0.5	-	58.00	33.50	39
IRC_IRT_c75731	109	KIZ07679.1	0.4	-	56.00	33.11	37
IRC_IRT_c75756	107	NP_001053719.2	0.0	GO:0003677	97.00	77.80	35
IRC_IRT_c75782	119	ABA95065.1	0.0	GO:0003676	100.00	75.87	37
IRC_IRT_c75829	103	EAZ18627.1	0.0	GO:0008171	100.00	73.56	34
IRC_IRT_c75854	170	KFK43975.1	0.1	-	56.00	35.81	48
IRC_IRT_c75864	119	XP_006662267.1	0.0	GO:0016021	97.00	78.18	38
IRC_IRT_c75873	118	EMS64867.1	0.5	-	62.00	32.34	37
IRC_IRT_c75876	119	AAP53572.1	0.0	-	59.00	34.27	37
IRC_IRT_c75899	105	XP_002534890.1	0.0	GO:0005773	88.00	53.91	34
IRC_IRT_c75916	195	CDX71648.1	0.0	-	86.00	62.77	37
IRC_IRT_c75958	111	KJB37455.1	0.9	-	60.00	31.96	38
IRC_IRT_c75987	119	XP_009350817.1	0.0	-	76.00	50.45	39

IRC_IRT_c75991	136	CDP10068.1	0.0	-	60.00	39.28	41
IRC_IRT_c75999	200	BAJ11784.1	0.7	-	53.00	33.11	58
IRC_IRT_c76003	223	XP_010517478.1	0.7	-	58.00	33.50	48
IRC_IRT_c76009	108	KEH15587.1	0.0	GO:0005975	80.00	48.52	35
IRC_IRT_c76010	246	AGC78943.1	0.0	-	72.00	57.00	47
IRC_IRT_c76013	185	EXB36957.1	0.0	GO:0009507	85.00	59.69	34
IRC_IRT_c76032	124	EYU34177.1	0.8	-	58.00	32.73	39
IRC_IRT_c76043	143	ACG27180.1	0.0	-	52.00	36.19	44
IRC_IRT_c76070	153	YP_358636.1	0.0	GO:0009536	72.00	50.83	36
IRC_IRT_c76074	115	BAG89721.1	0.0	-	66.00	38.89	42
IRC_IRT_c76094	244	KEH17697.1	0.0	-	70.00	38.51	34
IRC_IRT_c76097	148	EYU23373.1	0.8	-	58.00	32.34	51
IRC_IRT_c76098	107	XP_002540433.1	0.0	-	85.00	56.23	35
IRC_IRT_c76117	148	XP_006352375.1	0.9	-	56.00	32.34	39
IRC_IRT_c76131	167	AGC78943.1	0.0	-	55.00	36.19	40
IRC_IRT_c76137	214	BAJ11779.1	0.0	-	60.00	40.82	41
IRC_IRT_c76158	154	KJB09764.1	0.1	-	55.00	33.50	49
IRC_IRT_c76168	147	XP_008344995.1	0.6	-	62.00	33.11	40
IRC_IRT_c76310	113	EMS55579.1	0.0	GO:0005840	100.00	74.71	35
IRC_IRT_c76311	157	XP_006655150.1	0.0	-	76.00	40.43	39
IRC_IRT_c76346	132	XP_002536209.1	0.0	-	69.00	47.37	43
IRC_IRT_c76354	168	CDY63595.1	0.3	-	62.00	33.88	37
IRC_IRT_c76371	119	XP_001767474.1	0.0	-	62.00	46.21	35
IRC_IRT_c76403	108	XP_002534724.1	0.0	-	68.00	40.82	35
IRC_IRT_c76485	137	XP_002537159.1	0.0	-	65.00	35.42	38
IRC_IRT_c76525	104	XP_009350065.1	0.0	GO:0016021	88.00	45.82	34
IRC_IRT_c76591	141	XP_007204594.1	0.1	-	46.00	35.81	49
IRC_IRT_c76642	127	EAY73387.1	0.0	GO:0016747	94.00	42.36	37
IRC_IRT_c76669	113	BAC55802.1	0.0	-	94.00	70.09	35
IRC_IRT_c76753	147	AGG09511.1	0.0	GO:0003899	87.00	66.63	47
IRC_IRT_c76760	117	AAN65034.1	0.0	GO:0004523	86.00	65.86	36
IRC_IRT_c76794	112	EEC79016.1	0.0	GO:0045893	91.00	68.55	35
IRC_IRT_c76807	113	CAE02983.2	0.0	GO:0016023	94.00	72.40	36

IRC_IRT_c76898	130	NP_001057538.1	0.0	GO:0032259	83.00	71.63	43
IRC_IRT_c77044	118	XP_003062589.1	0.0	-	68.00	43.90	38
IRC_IRT_c77081	116	XP_001701244.1	0.3	-	64.00	32.34	37
IRC_IRT_c77086	101	XP_002505348.1	0.0	-	64.00	34.65	34
IRC_IRT_c77107	107	XP_005644405.1	0.0	-	72.00	38.51	37
IRC_IRT_c77112	124	XP_001786469.1	0.0	-	73.00	42.74	34
IRC_IRT_c77122	117	CAK51544.1	0.0	-	65.00	45.05	35
IRC_IRT_c77167	112	CAN74450.1	0.0	-	64.00	38.12	37
IRC_IRT_c77225	131	BAD46725.1	0.0	GO:0043231	46.00	49.29	67
IRC_IRT_c77300	181	XP_005844407.1	0.0	GO:0043231	62.00	55.07	53
IRC_IRT_c77352	113	EEE64125.1	0.0	-	100.00	71.25	35
IRC_IRT_c77388	118	XP_002536064.1	0.2	-	64.00	34.27	37
IRC_IRT_c77434	136	ABG66008.1	0.0	GO:0046872	75.00	54.30	45
IRC_IRT_c77448	125	XP_002539229.1	0.0	-	65.00	40.43	38
IRC_IRT_c77491	119	XP_002536203.1	0.0	-	79.00	44.67	34
IRC_IRT_c77513	117	AAX96685.1	0.0	GO:0004523	97.00	78.95	37
IRC_IRT_c77558	108	XP_003580608.1	0.0	GO:0046872	74.00	47.37	35
IRC_IRT_c77611	132	BAD54650.1	0.0	GO:0005739	83.00	66.63	43
IRC_IRT_c77617	134	EPS66459.1	0.0	-	68.00	38.12	44
IRC_IRT_c77643	101	AAA33485.1	0.0	-	67.00	36.58	34
IRC_IRT_c77688	121	AAM22006.1	0.0	GO:0003676	79.00	50.06	34
IRC_IRT_c77726	115	XP_006305219.1	0.0	-	67.00	38.12	37
IRC_IRT_c77738	111	XP_002536130.1	0.0	-	62.00	40.05	35
IRC_IRT_c77760	165	XP_010916757.1	0.0	GO:0055114	80.00	70.48	45
IRC_IRT_c77813	118	BAJ90008.1	0.7	-	57.00	32.73	35
IRC_IRT_c77827	111	EAZ19624.1	0.0	-	88.00	67.78	35
IRC_IRT_c77855	112	BAD72395.1	0.1	-	65.00	34.27	35
IRC_IRT_c77884	115	XP_001752658.1	0.1	-	61.00	35.81	39
IRC_IRT_c77892	116	EDQ48479.1	0.0	GO:0020037	91.00	67.01	34
IRC_IRT_c77908	139	XP_002501871.1	0.0	-	55.00	40.05	40
IRC_IRT_c77984	123	EEC70278.1	0.0	GO:0030170	82.00	53.53	35
IRC_IRT_c77993	180	XP_003574338.1	0.0	-	69.00	38.89	36
IRC_IRT_c78089	120	BAB86174.1	0.0	GO:0016023	94.00	74.71	34

IRC_IRT_c78107	133	EEC76122.1	0.0	GO:0050660	82.00	57.77	41
IRC_IRT_c78214	134	XP_003079392.1	0.6	-	62.00	33.11	35
IRC_IRT_c78250	129	XP_004968649.1	0.1	-	60.00	36.19	41
IRC_IRT_c78266	125	KIZ04667.1	0.0	GO:0005739	75.00	56.61	37
IRC_IRT_c78290	156	XP_008383465.1	0.0	GO:0016192	100.00	114.01	51
IRC_IRT_c78299	125	XP_002535157.1	0.0	GO:0016491	76.00	50.06	38
IRC_IRT_c78352	141	EAY93041.1	0.0	GO:0003676	100.00	103.99	47
IRC_IRT_c78353	113	AAG41903.1	0.0	GO:1902358	97.00	61.62	34
IRC_IRT_c78574	129	CAE06006.3	0.0	-	66.00	40.82	42
IRC_IRT_c78616	157	KCW46867.1	0.6	-	52.00	33.11	42
IRC_IRT_c78632	120	XP_002537280.1	0.2	-	64.00	33.88	37
IRC_IRT_c78671	112	XP_002507709.1	0.0	GO:0003861	97.00	70.09	34
IRC_IRT_c78681	117	XP_004253349.1	0.0	-	64.00	40.05	37
IRC_IRT_c78704	129	ABA97569.1	0.0	GO:0003676	97.00	80.11	41
IRC_IRT_c78719	121	ABA96535.1	0.0	-	92.00	46.60	38
IRC_IRT_c78728	183	XP_009629910.1	0.1	-	54.00	34.65	42
IRC_IRT_c78893	113	KDD74693.1	0.0	GO:0016874	91.00	63.93	35
IRC_IRT_c78901	142	XP_005643602.1	0.0	-	64.00	49.68	50
IRC_IRT_c78916	114	XP_002537102.1	0.0	-	78.00	43.13	37
IRC_IRT_c78917	118	XP_002540227.1	0.0	-	58.00	37.35	34
IRC_IRT_c78932	127	XP_005651253.1	0.0	GO:0003824	73.00	51.22	38
IRC_IRT_c78934	111	NP_001061541.1	0.0	GO:0045893	100.00	85.50	37
IRC_IRT_c78970	132	EEC77111.1	0.0	GO:0050896	76.00	43.13	42
IRC_IRT_c78977	111	AAL65397.1	0.0	GO:0005739	69.00	41.97	36
IRC_IRT_c79036	114	XP_005850501.1	0.0	-	64.00	37.74	37
IRC_IRT_c79049	108	XP_003570336.1	0.2	-	58.00	34.27	34
IRC_IRT_c79099	127	ABA96519.2	0.0	GO:0032508	82.00	62.00	34
IRC_IRT_c79157	182	XP_009350067.1	0.0	-	75.00	44.67	37
IRC_IRT_c79175	110	EEC70596.1	0.0	GO:0043531	100.00	77.41	36
IRC_IRT_c79200	151	XP_010277053.1	0.2	-	55.00	33.50	40
IRC_IRT_c79261	127	EMT26036.1	0.0	-	61.00	41.59	42
IRC_IRT_c79312	109	EAZ18783.1	0.0	GO:0042545	97.00	72.40	36
IRC_IRT_c79325	103	XP_002538700.1	0.1	-	70.00	35.04	34

IRC_IRT_c79501	108	AAO37469.1	0.0	GO:0004523	91.00	65.86	3.60E+01
IRC_IRT_c79563	148	BAD25084.1	0.4	-	94.00	33.50	35
IRC_IRT_c79639	128	CAB90608.1	0.0	GO:0008152	68.00	47.75	41
IRC_IRT_c79649	112	EMS50766.1	0.0	GO:0005773	77.00	53.14	35
IRC_IRT_c79711	137	XP_001773781.1	0.0	GO:0046777	78.00	60.08	41
IRC_IRT_c79731	130	XP_003617749.1	0.2	-	54.00	31.96	35
IRC_IRT_c79761	134	KIY97704.1	0.6	-	52.00	33.11	34
IRC_IRT_c79772	124	XP_003615709.1	0.3	-	56.00	34.27	37
IRC_IRT_c79792	135	AFK43044.1	0.2	-	55.00	32.73	38
IRC_IRT_c79797	114	EAZ16455.1	0.0	GO:0005901	100.00	77.03	38
IRC_IRT_c79814	130	NP_001049858.1	0.0	GO:0042545	100.00	100.52	43
IRC_IRT_c79879	108	EMT32379.1	0.0	GO:0004672	77.00	49.68	35
IRC_IRT_c79896	178	EAY81384.1	0.0	-	60.00	41.20	41
IRC_IRT_c79933	109	EMT10240.1	0.3	-	62.00	33.50	37
IRC_IRT_c79971	111	ABA98369.1	0.0	GO:0004252	97.00	72.40	36
IRC_IRT_c79980	164	EAY86051.1	0.6	-	48.00	32.73	37
IRC_IRT_c79982	131	BAJ84923.1	0.0	GO:0000155	75.00	50.06	40
IRC_IRT_c80066	147	ACF83096.1	0.0	GO:0005840	94.00	69.32	3.80E+01
IRC_IRT_c80117	116	EEC77198.1	0.0	GO:0007059	94.00	69.32	38
IRC_IRT_c80160	142	XP_003606040.1	0.5	-	52.00	31.57	40
IRC_IRT_c80191	107	KDD74570.1	0.2	-	61.00	33.88	34
IRC_IRT_c80198	140	XP_001764723.1	0.8	-	54.00	31.19	37
IRC_IRT_c80215	136	XP_002539840.1	0.0	-	89.00	78.57	39
IRC_IRT_c80229	136	NP_001046468.1	0.0	GO:0005975	100.00	93.20	45
IRC_IRT_c80239	105	CAJ86113.1	0.0	GO:0005634	85.00	60.08	34
IRC_IRT_c80260	134	DAA44154.1	0.0	-	60.00	35.42	43
IRC_IRT_c80298	146	XP_001770675.1	0.0	-	71.00	45.05	38
IRC_IRT_c80303	124	EAZ03673.1	0.0	-	100.00	84.73	36
IRC_IRT_c80319	113	NP_001118889.1	0.7	-	52.00	30.03	36
IRC_IRT_c80322	147	ERN10593.1	0.0	-	65.00	42.74	38
IRC_IRT_c80338	120	CDP00801.1	0.0	-	65.00	36.19	35
IRC_IRT_c80347	109	KFM27114.1	0.0	GO:0005507	80.00	51.60	36
IRC_IRT_c80351	282	AGZ19352.1	0.0	GO:0009507	83.00	53.91	36

IRC_IRT_c80377	106	AAF16526.1	0.0	GO:0044763	70.00	47.37	34
IRC_IRT_c80418	133	AIA92249.1	0.0	GO:0097159	70.00	43.90	41
IRC_IRT_c80459	112	CCQ71893.1	0.7	-	66.00	31.96	36
IRC_IRT_c80524	111	AGT78195.1	0.0	GO:0009536	88.00	73.17	35
IRC_IRT_c80529	105	BAC45187.1	0.0	GO:0003677	100.00	73.56	34
IRC_IRT_c80602	137	XP_002537860.1	0.0	GO:0050794	72.00	56.61	40
IRC_IRT_c80637	127	XP_002536215.1	0.1	-	62.00	34.27	37
IRC_IRT_c80672	108	XP_002537871.1	1.0	-	64.00	31.96	34
IRC_IRT_c80691	135	ABF96189.1	0.0	GO:0009536	93.00	84.73	44
IRC_IRT_c80707	133	NP_001288502.1	0.0	-	66.00	53.53	3.90E+01
IRC_IRT_c80720	114	XP_011002134.1	0.0	GO:0016207	76.00	52.76	38
IRC_IRT_c80801	143	EEC76879.1	0.0	GO:0009107	85.00	79.34	47
IRC_IRT_c80821	132	CAN69231.1	0.2	-	51.00	34.27	39
IRC_IRT_c80826	166	KEH29749.1	0.4	-	51.00	33.50	45
IRC_IRT_c80832	142	XP_006492424.1	0.0	GO:0009646	80.00	62.39	41
IRC_IRT_c80858	146	XP_008357876.1	0.1	-	53.00	35.04	45
IRC_IRT_c80869	111	EMT29572.1	0.7	-	53.00	31.96	41
IRC_IRT_c80881	117	AFK46251.1	0.0	GO:0004550	72.00	48.52	36
IRC_IRT_c80934	136	XP_002540253.1	0.0	GO:0050660	93.00	77.03	43
IRC_IRT_c80949	102	XP_004958607.1	0.0	GO:0016491	79.00	52.37	34
IRC_IRT_c80958	122	XP_002981097.1	0.0	-	77.00	51.60	40
IRC_IRT_c80966	130	XP_002500999.1	0.1	-	67.00	35.04	40
IRC_IRT_c80990	104	EMT22121.1	0.0	GO:0044699	75.00	42.74	36
IRC_IRT_c80998	112	XP_010065589.1	0.0	-	70.00	47.75	34
IRC_IRT_c81002	263	ERN16843.1	0.0	-	57.00	42.74	56
IRC_IRT_c81039	132	XP_009351046.1	0.0	-	74.00	59.31	43
IRC_IRT_c81076	112	XP_005851680.1	0.0	-	67.00	40.43	37
IRC_IRT_c81079	103	ABA96051.1	0.0	GO:0036459	97.00	68.17	34
IRC_IRT_c81082	107	NP_683837.1	0.0	-	70.00	39.66	34
IRC_IRT_c81102	148	XP_010938895.1	0.4	-	59.00	33.50	37
IRC_IRT_c81110	120	XP_004976988.1	0.2	-	63.00	34.65	38
IRC_IRT_c81115	212	EPS74511.1	0.0	-	56.00	37.35	46
IRC_IRT_c81121	120	XP_009123987.1	0.3	-	53.00	33.88	39

IRC_IRT_c81144	144	XP_009350051.1	0.0	GO:0003735	80.00	51.60	41
IRC_IRT_c81193	108	EAY89301.1	0.0	GO:0006094	100.00	78.57	36
IRC_IRT_c81228	153	XP_002535157.1	0.0	-	70.00	39.66	34
IRC_IRT_c81236	232	EMT28358.1	0.8	-	45.00	33.50	79
IRC_IRT_c81255	135	XP_006651176.1	0.9	-	54.00	32.34	42
IRC_IRT_c81261	107	XP_001787071.1	0.0	-	67.00	38.51	34
IRC_IRT_c81287	112	BAC20683.1	0.0	-	74.00	43.90	35
IRC_IRT_c81300	150	XP_006478563.1	0.8	-	52.00	32.73	42
IRC_IRT_c81313	211	EEC70905.1	0.0	GO:0016616	69.00	46.60	39
IRC_IRT_c81333	108	XP_003062581.1	0.0	GO:0031071	79.00	46.60	34
IRC_IRT_c81369	201	XP_003616487.1	0.1	-	52.00	36.19	48
IRC_IRT_c81391	125	XP_003079192.1	0.0	GO:0016491	68.00	45.82	41
IRC_IRT_c81412	173	EPS58073.1	0.0	-	59.00	36.19	42
IRC_IRT_c81440	131	ERN20334.1	0.1	-	61.00	33.50	34
IRC_IRT_c81519	151	EAY87012.1	0.0	GO:0016788	100.00	95.13	46
IRC_IRT_c81537	106	XP_001700210.1	0.0	GO:0016874	71.00	52.37	35
IRC_IRT_c81637	105	XP_010056074.1	0.0	GO:0046872	70.00	41.97	34
IRC_IRT_c81659	106	ABA95590.2	0.0	-	77.00	60.08	35
IRC_IRT_c81816	119	CDY63598.1	0.0	-	61.00	37.35	34
IRC_IRT_c81883	122	ABK22261.1	0.4	-	53.00	32.73	49
IRC_IRT_c81920	103	XP_002534770.1	0.2	-	70.00	33.88	34
IRC_IRT_c81934	137	XP_001786560.1	0.0	GO:0044763	68.00	43.51	35
IRC_IRT_c81936	111	XP_002535442.1	0.0	GO:0044763	72.00	52.37	37
IRC_IRT_c81943	115	XP_002535080.1	0.0	GO:0044699	78.00	46.60	37
IRC_IRT_c81945	132	XP_001786598.1	0.8	-	62.00	32.34	37
IRC_IRT_c81950	132	XP_001702533.1	0.1	-	62.00	33.50	45
IRC_IRT_c81958	117	EAY98952.1	0.0	GO:0005484	100.00	79.34	38
IRC_IRT_c81979	122	EEC77855.1	0.0	GO:0050896	78.00	45.05	38
IRC_IRT_c81993	118	XP_005846625.1	0.5	-	58.00	32.73	34
IRC_IRT_c82009	131	ABF95256.1	0.0	-	79.00	58.15	43
IRC_IRT_c82053	112	XP_008462935.1	0.9	-	58.00	32.34	34
IRC_IRT_c82057	109	EPS57968.1	0.0	-	66.00	36.58	36
IRC_IRT_c82067	111	XP_002537471.1	0.1	-	55.00	34.65	36

IRC_IRT_c82069	113	CDM80443.1	0.0	-	64.00	38.89	37
IRC_IRT_c82071	124	AEB33086.1	0.8	-	63.00	30.03	36
IRC_IRT_c82126	109	XP_009350076.1	0.0	GO:0005525	94.00	75.10	36
IRC_IRT_c82154	146	BAC06270.1	0.0	-	87.00	72.02	47
IRC_IRT_c82326	122	XP_011095404.1	0.7	-	62.00	32.73	35
IRC_IRT_c82350	114	XP_007042336.1	0.5	-	62.00	32.73	37
IRC_IRT_c82390	168	CAE04595.2	0.0	GO:0004568	93.00	83.96	43
IRC_IRT_c82525	102	BAD88083.1	0.0	-	100.00	72.40	34
IRC_IRT_c82562	127	XP_005851907.1	0.0	GO:0005507	84.00	69.71	39
IRC_IRT_c82576	106	EAZ05518.1	0.0	GO:0016023	100.00	76.64	35
IRC_IRT_c82592	119	ACF04631.1	0.0	GO:0016620	83.00	56.23	37
IRC_IRT_c82610	127	EMT02573.1	0.0	GO:0005524	83.00	57.38	36
IRC_IRT_c82844	104	CAH66032.1	0.0	-	69.00	42.36	36
IRC_IRT_c82908	102	XP_011000341.1	0.7	-	67.00	32.73	34
IRC_IRT_c82972	116	AAK92670.1	0.0	GO:0004523	97.00	80.11	38
IRC_IRT_c82998	159	XP_001692885.1	0.0	GO:0009630	84.00	92.05	53
IRC_IRT_c83103	112	CAE01940.2	0.0	GO:0004523	100.00	79.34	37
IRC_IRT_c83111	114	XP_006827758.1	0.0	-	70.00	45.82	40
IRC_IRT_c83147	118	EEE70246.1	0.0	GO:0003676	87.00	78.18	39
IRC_IRT_c83152	138	XP_002535283.1	0.0	-	68.00	43.51	35
IRC_IRT_c83161	105	BAD07507.1	0.0	GO:0016023	100.00	71.25	34
IRC_IRT_c83224	160	XP_006664369.1	1.0	-	61.00	32.73	39
IRC_IRT_c83225	140	KEH15576.1	0.0	-	55.00	43.90	45
IRC_IRT_c83257	108	EEE69014.1	0.0	GO:0010368	82.00	62.77	35
IRC_IRT_c83318	120	CBI25687.3	0.7	-	64.00	33.11	34
IRC_IRT_c83344	156	AAS07108.1	0.0	-	59.00	35.81	42
IRC_IRT_c83357	103	XP_003079014.1	0.0	GO:0006499	88.00	68.17	34
IRC_IRT_c83442	118	XP_002440052.1	0.0	-	87.00	64.70	39
IRC_IRT_c83443	134	XP_009350810.1	0.0	-	72.00	64.31	44
IRC_IRT_c83446	163	XP_010941424.1	0.4	-	59.00	33.88	49
IRC_IRT_c83458	109	XP_002534904.1	0.0	GO:0009396	85.00	57.77	35
IRC_IRT_c83575	137	XP_002540540.1	0.0	GO:0003824	79.00	53.53	34
IRC_IRT_c83591	158	KGN48087.1	0.9	-	65.00	32.73	38

IRC_IRT_c83625	202	CAH66115.1	0.0	GO:0016787	75.00	103.99	66
IRC_IRT_c83632	166	EEC76774.1	0.2	-	58.00	32.34	34
IRC_IRT_c83648	114	AGL72331.1	0.0	GO:0055085	70.00	40.82	37
IRC_IRT_c83672	115	BAC79678.1	0.0	GO:0009536	73.00	48.14	34
IRC_IRT_c83676	118	KDD74673.1	0.0	-	64.00	37.74	39
IRC_IRT_c83786	148	EEC84059.1	0.0	GO:0006306	100.00	105.92	49
IRC_IRT_c83803	102	BAC05657.1	0.0	GO:0004523	88.00	59.69	34
IRC_IRT_c83819	123	XP_001768881.1	0.8	-	61.00	32.34	34
IRC_IRT_c83858	132	NP_001052290.1	0.0	GO:0045551	100.00	87.43	39
IRC_IRT_c83882	154	XP_009356911.1	0.1	-	68.00	33.88	44
IRC_IRT_c83890	111	CDY67219.1	0.0	-	60.00	35.81	35
IRC_IRT_c83892	128	XP_002540540.1	0.0	-	75.00	56.23	41
IRC_IRT_c83916	123	XP_011016509.1	0.0	GO:0003735	87.00	68.94	41
IRC_IRT_c83919	116	NP_192386.1	0.0	GO:0006810	78.00	56.61	38
IRC_IRT_c83946	148	XP_009785472.1	0.1	-	62.00	34.65	35
IRC_IRT_c84004	126	XP_008778202.1	0.0	-	57.00	36.19	38
IRC_IRT_c84019	234	EPS70027.1	0.3	-	51.00	32.73	45
IRC_IRT_c84023	194	BAA10929.1	0.0	-	70.00	43.13	40
IRC_IRT_c84055	103	XP_002535771.1	0.0	GO:0006184	91.00	57.00	34
IRC_IRT_c84090	124	XP_009138427.1	0.0	-	58.00	39.28	41
IRC_IRT_c84110	108	NP_001054004.1	0.0	-	100.00	74.71	35
IRC_IRT_c84137	179	NP_001053871.1	0.0	GO:0003676	81.00	63.93	4.30E+01
IRC_IRT_c84167	110	XP_007135146.1	0.0	GO:0003864	80.00	52.37	36
IRC_IRT_c84170	122	XP_004253346.1	0.0	-	67.00	40.43	40
IRC_IRT_c84172	102	XP_010919654.1	0.0	-	64.00	40.05	37
IRC_IRT_c84179	116	XP_001692833.1	0.1	-	60.00	35.81	35
IRC_IRT_c84207	117	CDX73321.1	0.7	-	66.00	31.96	39
IRC_IRT_c84260	116	EAZ06076.1	0.0	-	100.00	89.74	38
IRC_IRT_c84267	117	XP_002537530.1	0.0	GO:0016757	86.00	50.06	38
IRC_IRT_c84329	125	ABA96719.2	0.0	GO:0050660	97.00	54.30	41
IRC_IRT_c84348	112	EAY79487.1	0.2	-	54.00	33.88	35
IRC_IRT_c84351	150	KEH40309.1	0.8	-	65.00	33.11	35
IRC_IRT_c84401	143	KFK33931.1	0.4	-	55.00	33.50	47

IRC_IRT_c84409	113	XP_002536731.1	0.0	-	61.00	36.19	36
IRC_IRT_c84415	126	XP_002946539.1	0.0	GO:0034976	92.00	78.18	41
IRC_IRT_c84520	115	EAY73499.1	0.0	GO:0008270	89.00	67.40	37
IRC_IRT_c84530	130	XP_001415746.1	0.0	GO:0005786	76.00	63.54	43
IRC_IRT_c84538	140	XP_002539422.1	0.0	GO:0016798	59.00	46.60	42
IRC_IRT_c84600	178	AIQ78384.1	0.0	GO:0004519	97.00	65.86	34
IRC_IRT_c84622	112	YP_588321.1	0.0	GO:0005739	94.00	75.49	37
IRC_IRT_c84668	159	CBI40480.3	0.5	-	58.00	33.50	48
IRC_IRT_c84669	149	XP_002953315.1	0.0	-	63.00	40.05	44
IRC_IRT_c84675	130	XP_006293258.1	0.0	-	57.00	36.58	38
IRC_IRT_c84710	103	XP_001696864.1	0.0	GO:0019363	75.00	50.06	36
IRC_IRT_c84736	113	XP_002535867.1	0.5	-	62.00	32.34	35
IRC_IRT_c84762	120	AAC49379.1	0.0	GO:0044249	78.00	57.77	37
IRC_IRT_c84819	110	NP_001059097.1	0.0	-	80.00	53.91	36
IRC_IRT_c84907	142	EEE50456.1	0.0	GO:0008152	97.00	88.97	44
IRC_IRT_c85003	135	XP_009351102.1	0.0	-	88.00	68.55	36
IRC_IRT_c85030	115	AAV59449.1	0.0	GO:0016021	100.00	78.95	3.80E+01
IRC_IRT_c85098	104	XP_003055236.1	0.0	-	76.00	39.28	34
IRC_IRT_c85102	111	XP_002536453.1	0.0	-	77.00	44.67	35
IRC_IRT_c85121	146	KFM23587.1	0.0	-	58.00	41.97	46
IRC_IRT_c85140	121	XP_011086087.1	0.2	-	47.00	34.65	46
IRC_IRT_c85159	109	XP_002505558.1	0.3	-	64.00	33.88	34
IRC_IRT_c85216	120	AAQ56447.1	0.0	-	55.00	36.97	40
IRC_IRT_c85255	102	EEC69818.1	0.7	-	55.00	31.57	38
IRC_IRT_c85314	115	XP_002535829.1	0.1	-	56.00	35.42	37
IRC_IRT_c85345	105	XP_009604409.1	0.8	-	58.00	31.96	34
IRC_IRT_c85409	104	NP_001064787.1	0.0	GO:0009827	100.00	70.09	34
IRC_IRT_c85429	153	XP_001754744.1	0.0	GO:0007165	69.00	47.75	49
IRC_IRT_c85439	133	KCW49639.1	0.0	-	65.00	40.05	43
IRC_IRT_c85453	104	KFM27633.1	0.0	GO:0016747	79.00	47.37	34
IRC_IRT_c85537	128	EEE66382.1	0.0	GO:0016788	100.00	90.51	42
IRC_IRT_c85557	123	XP_006347175.1	0.4	-	59.00	32.73	37
IRC_IRT_c85605	106	XP_004503265.1	0.0	GO:0017111	74.00	42.74	35

IRC_IRT_c85608	115	XP_001417951.1	0.0	GO:0008026	83.00	49.29	36
IRC_IRT_c85651	102	XP_001760834.1	0.7	-	58.00	31.96	34
IRC_IRT_c85669	102	XP_003084003.1	0.0	-	73.00	39.28	34
IRC_IRT_c85742	119	KJB41985.1	0.7	-	55.00	32.73	36
IRC_IRT_c85845	103	CAE05201.3	0.0	GO:0009899	100.00	77.03	34
IRC_IRT_c85865	146	BAD26228.1	0.0	-	100.00	100.91	48
IRC_IRT_c85869	126	EAZ14329.1	0.0	GO:0051536	100.00	73.17	37
IRC_IRT_c85877	114	XP_003060292.1	0.0	-	64.00	39.28	34
IRC_IRT_c85959	127	KFM28025.1	0.1	-	55.00	35.04	38
IRC_IRT_c85985	105	ABA94261.1	0.0	GO:0003676	79.00	50.83	34
IRC_IRT_c85988	112	XP_004502457.1	0.1	-	61.00	34.65	34
IRC_IRT_c86048	105	NP_001174623.1	0.0	GO:0046983	100.00	55.07	34
IRC_IRT_c86116	140	XP_002537036.1	0.0	GO:0006810	86.00	57.77	45
IRC_IRT_c86125	103	XP_002530184.1	0.8	-	76.00	31.96	34
IRC_IRT_c86244	106	NP_001050226.1	0.0	GO:0006310	97.00	68.17	3.50E+01
IRC_IRT_c86368	118	XP_003635968.1	0.0	GO:0017111	77.00	46.60	36
IRC_IRT_c86380	128	NP_001042027.2	0.0	GO:0016310	75.00	48.52	36
IRC_IRT_c86395	101	XP_006586715.1	0.1	-	62.00	35.04	35
IRC_IRT_c86514	113	BAB90519.1	0.5	-	55.00	32.73	36
IRC_IRT_c86520	118	XP_003079879.1	0.0	-	71.00	37.35	35
IRC_IRT_c86669	157	XP_007144170.1	0.3	-	61.00	34.27	34
IRC_IRT_c86680	111	XP_010231165.1	0.8	-	55.00	31.96	36
IRC_IRT_c86721	113	XP_010911538.1	0.0	-	61.00	38.89	34
IRC_IRT_c86761	141	XP_009350805.1	0.0	GO:0048731	86.00	70.09	43
IRC_IRT_c86784	121	XP_002509038.1	0.1	-	52.00	35.81	34
IRC_IRT_c86825	119	XP_002535112.1	0.0	-	64.00	36.19	39
IRC_IRT_c86843	124	DAA39142.1	0.0	-	65.00	42.74	38
IRC_IRT_c86897	138	XP_002535920.1	0.0	GO:0090599	78.00	62.77	42
IRC_IRT_c86955	142	AAR15338.1	0.0	-	71.00	42.74	35
IRC_IRT_c87059	109	BAD54639.1	0.1	-	56.00	34.65	39
IRC_IRT_c87103	111	AAK27822.1	0.0	GO:0004523	94.00	73.94	36
IRC_IRT_c87119	115	CAE02421.2	0.0	GO:0046923	94.00	62.77	34
IRC_IRT_c87137	152	AAN65369.1	0.2	-	50.00	33.11	44

IRC_IRT_c87145	150	XP_009150001.1	0.3	-	54.00	33.88	42
IRC_IRT_c87147	170	XP_005645846.1	0.2	-	53.00	34.65	47
IRC_IRT_c87153	143	XP_005648656.1	0.1	-	52.00	33.88	36
IRC_IRT_c87154	144	KIZ06735.1	0.0	-	74.00	41.59	35
IRC_IRT_c87160	124	XP_011016758.1	0.0	-	76.00	47.75	34
IRC_IRT_c87185	146	EMT23357.1	0.9	-	53.00	32.73	43
IRC_IRT_c87209	104	CAE05392.1	0.0	GO:0003964	97.00	73.17	34
IRC_IRT_c87222	138	AAG12666.1	0.0	GO:0050789	72.00	45.44	40
IRC_IRT_c87271	152	EEC72475.1	0.0	GO:0009536	69.00	59.31	49
IRC_IRT_c87367	147	XP_007214751.1	0.3	-	60.00	33.88	41
IRC_IRT_c87398	118	CCO17359.1	0.1	-	60.00	35.81	35
IRC_IRT_c87413	104	XP_001419083.1	0.0	-	67.00	35.81	34
IRC_IRT_c87443	131	XP_002535044.1	0.3	-	56.00	33.50	39
IRC_IRT_c87473	106	XP_010237399.1	0.3	-	61.00	33.50	34
IRC_IRT_c87483	110	EEC78973.1	0.3	-	75.00	32.34	41
IRC_IRT_c87570	114	EEC82824.1	0.0	GO:0008152	97.00	68.55	34
IRC_IRT_c87629	116	XP_002535966.1	0.0	-	65.00	39.66	35
IRC_IRT_c87690	107	XP_002536943.1	0.0	GO:0006355	80.00	43.90	35
IRC_IRT_c87696	111	XP_010907405.1	0.0	GO:0005524	88.00	61.23	36
IRC_IRT_c87698	162	XP_001762415.1	0.0	-	54.00	36.97	53
IRC_IRT_c87711	148	XP_002512605.1	0.8	-	52.00	32.73	57
IRC_IRT_c87716	134	EEC72914.1	0.0	GO:0020037	100.00	90.89	44
IRC_IRT_c87802	132	XP_006361477.1	0.0	-	53.00	41.97	43
IRC_IRT_c87806	126	XP_002539702.1	0.0	GO:0016620	69.00	56.61	42
IRC_IRT_c87821	106	XP_004253300.1	0.0	-	91.00	64.70	34
IRC_IRT_c87824	121	XP_002536469.1	0.0	GO:0004872	91.00	68.55	37
IRC_IRT_c87853	116	XP_002536283.1	0.1	-	60.00	34.65	35
IRC_IRT_c88033	120	XP_002538133.1	0.1	-	61.00	34.65	39
IRC_IRT_c88048	118	CDP13792.1	0.7	-	57.00	32.73	35
IRC_IRT_c88073	134	CEO91102.1	0.1	-	60.00	35.04	38
IRC_IRT_c88079	114	XP_002537161.1	0.0	GO:1902358	81.00	51.99	38
IRC_IRT_c88168	112	XP_002535378.1	0.0	GO:0016021	86.00	67.78	36
IRC_IRT_c88231	114	XP_002539786.1	0.0	-	68.00	41.97	35

IRC_IRT_c88240	180	NP_001173040.1	0.0	-	100.00	96.29	44
IRC_IRT_c88336	120	CCO18520.1	0.5	-	52.00	33.11	34
IRC_IRT_c88358	183	XP_002958608.1	0.5	-	50.00	33.11	58
IRC_IRT_c88360	120	XP_009420670.1	0.1	-	61.00	34.65	34
IRC_IRT_c88408	104	XP_002538609.1	0.0	GO:0003824	67.00	43.90	34
IRC_IRT_c88443	166	CCO19895.1	0.7	-	67.00	33.11	37
IRC_IRT_c88475	116	NP_001068251.1	0.0	GO:0016023	89.00	71.63	3.80E+01
IRC_IRT_c88486	186	KDP44876.1	0.0	-	62.00	44.67	40
IRC_IRT_c88509	113	EEE66776.1	0.0	GO:0005351	100.00	77.41	36
IRC_IRT_c88541	102	XP_002524496.1	0.0	-	79.00	39.28	34
IRC_IRT_c88587	104	KIZ03936.1	0.0	-	61.00	37.74	34
IRC_IRT_c88635	126	XP_003058676.1	0.4	-	58.00	32.73	46
IRC_IRT_c88645	110	EEC79860.1	0.0	GO:0050661	94.00	72.40	36
IRC_IRT_c88653	108	EEE50684.1	0.0	-	71.00	38.51	35
IRC_IRT_c88692	111	XP_002539970.1	0.0	-	72.00	38.89	37
IRC_IRT_c88724	163	XP_007033359.1	0.3	-	61.00	34.27	44
IRC_IRT_c88749	178	XP_006858053.1	0.0	GO:0004834	76.00	65.86	46
IRC_IRT_c88879	104	BAD81948.1	0.0	-	91.00	57.38	34
IRC_IRT_c88931	104	AAK50597.1	0.0	GO:0004523	94.00	62.77	34
IRC_IRT_c88940	156	EPS68500.1	0.0	-	57.00	38.51	47
IRC_IRT_c88952	111	XP_003057546.1	0.0	GO:0046872	83.00	55.45	36
IRC_IRT_c89012	121	EAY79370.1	0.0	GO:0005739	100.00	83.57	40
IRC_IRT_c89032	116	XP_003565079.1	0.6	-	60.00	32.73	38
IRC_IRT_c89111	124	CBI28923.3	0.7	-	64.00	31.57	34
IRC_IRT_c89119	102	EAY92800.1	0.0	-	71.00	42.36	35
IRC_IRT_c89125	154	NP_001067161.1	0.5	-	58.00	33.11	46
IRC_IRT_c89134	125	DAA05083.1	0.0	GO:0043565	89.00	74.71	39
IRC_IRT_c89158	128	AAK38083.1	0.0	GO:0016491	71.00	38.89	35
IRC_IRT_c89205	125	XP_011016126.1	0.0	GO:0005840	77.00	53.14	40
IRC_IRT_c89216	127	EEE67028.1	0.0	GO:0005739	87.00	75.87	40
IRC_IRT_c89248	216	EPS70026.1	0.0	-	60.00	40.82	50
IRC_IRT_c89252	201	XP_009350817.1	0.0	-	50.00	42.74	74
IRC_IRT_c89261	208	XP_010245798.1	0.8	-	44.00	33.11	50

IRC_IRT_c89271	235	XP_008447683.1	0.0	-	65.00	38.89	40
IRC_IRT_c89290	116	XP_007157025.1	0.7	-	50.00	32.34	42
IRC_IRT_c89322	137	XP_002538154.1	0.0	GO:0044763	74.00	57.77	39
IRC_IRT_c89330	141	XP_009388207.1	0.5	-	58.00	32.73	34
IRC_IRT_c89339	108	NP_001051333.1	0.0	GO:0005507	79.00	54.30	34
IRC_IRT_c89353	200	ERN19185.1	0.8	-	61.00	32.73	42
IRC_IRT_c89376	123	XP_008456821.1	0.0	-	60.00	36.58	41
IRC_IRT_c89393	161	ABF57915.1	0.4	-	50.00	33.50	52
IRC_IRT_c89411	183	KJB31094.1	0.0	-	67.00	43.13	37
IRC_IRT_c89413	116	KJB79847.1	0.0	GO:0048037	86.00	61.62	37
IRC_IRT_c89431	141	XP_002507890.1	0.0	GO:0005829	69.00	48.52	46
IRC_IRT_c89437	164	XP_009388289.1	0.0	-	82.00	48.52	35
IRC_IRT_c89441	118	XP_002523662.1	0.1	-	68.00	35.81	38
IRC_IRT_c89453	134	XP_002537315.1	0.0	-	65.00	50.45	41
IRC_IRT_c89461	176	CDM82334.1	0.0	-	51.00	34.65	56
IRC_IRT_c89463	136	AAY17051.1	0.4	-	62.00	31.57	35
IRC_IRT_c89467	154	EXB64218.1	0.4	-	52.00	33.50	46
IRC_IRT_c89471	115	XP_005644824.1	0.0	-	76.00	41.20	34
IRC_IRT_c89475	123	XP_003056422.1	0.7	-	50.00	32.73	46
IRC_IRT_c89478	121	XP_011045139.1	0.6	-	56.00	33.11	37
IRC_IRT_c89495	116	XP_009802310.1	0.0	GO:0008270	76.00	56.23	38
IRC_IRT_c89500	169	XP_001786556.1	0.0	GO:0004129	92.00	83.57	55
IRC_IRT_c89505	164	CAA75478.1	0.0	-	58.00	38.12	43
IRC_IRT_c89522	280	EXC01914.1	0.7	-	48.00	28.11	50
IRC_IRT_c89523	173	KIY96665.1	0.0	GO:0043231	82.00	61.23	40
IRC_IRT_c89525	115	AFD62278.1	0.0	GO:0055114	76.00	50.45	34
IRC_IRT_c89532	249	KJB31094.1	0.0	GO:0006810	69.00	46.60	36
IRC_IRT_c89533	213	ERN00555.1	0.0	-	72.00	45.82	37
IRC_IRT_c89551	114	XP_010911753.1	0.0	-	72.00	47.75	40
IRC_IRT_c89561	125	XP_001763943.1	0.0	GO:0006952	73.00	51.60	41
IRC_IRT_c89574	120	EDQ48391.1	0.0	GO:0046872	88.00	62.77	36
IRC_IRT_c89616	117	ABA97326.2	0.0	GO:0004523	89.00	71.63	39
IRC_IRT_c89649	184	NP_064083.1	0.5	-	51.00	33.50	54

IRC_IRT_c89666	112	XP_006646980.1	0.0	-	56.00	38.12	37
IRC_IRT_c89688	104	EAZ43153.1	0.0	GO:0080046	100.00	73.17	34
IRC_IRT_c89702	111	XP_006396687.1	0.2	-	61.00	33.88	34
IRC_IRT_c89714	106	XP_002535867.1	0.0	GO:0004731	91.00	68.17	34
IRC_IRT_c89727	116	EYU26003.1	0.0	GO:0009941	74.00	48.91	35
IRC_IRT_c89801	104	XP_002538058.1	0.0	GO:0044699	70.00	40.82	34
IRC_IRT_c89814	200	EPS74531.1	0.0	-	59.00	50.83	61
IRC_IRT_c89833	107	XP_001699137.1	0.0	-	73.00	38.89	34
IRC_IRT_c89843	114	BAD33288.1	0.0	-	72.00	36.58	36
IRC_IRT_c89854	126	EEE68603.1	0.0	-	97.00	66.24	35
IRC_IRT_c90074	114	KIZ05075.1	0.0	-	63.00	36.97	38
IRC_IRT_c90105	139	EEE64410.1	0.0	GO:0016023	100.00	104.38	46
IRC_IRT_c90166	236	XP_009388289.1	0.0	-	77.00	44.28	35
IRC_IRT_c90173	125	AFU96977.1	0.0	GO:0003899	94.00	73.17	39
IRC_IRT_c90197	154	XP_001775276.1	0.5	-	60.00	33.50	40
IRC_IRT_c90214	270	AGZ19352.1	0.0	GO:0006869	78.00	76.26	55
IRC_IRT_c90220	160	XP_010046011.1	0.0	-	55.00	49.29	61
IRC_IRT_c90235	126	XP_001772410.1	0.3	-	57.00	33.88	40
IRC_IRT_c90285	165	XP_009350076.1	0.0	GO:0008270	89.00	92.82	48
IRC_IRT_c90329	172	XP_002539662.1	0.0	GO:0016747	78.00	46.98	37
IRC_IRT_c90386	130	XP_002964495.1	0.0	-	59.00	37.74	37
IRC_IRT_c90392	256	BAJ11784.1	0.0	-	97.00	72.02	35
IRC_IRT_c90409	248	AGZ19352.1	0.0	-	59.00	46.60	74
IRC_IRT_c90431	159	KJB31094.1	0.0	-	56.00	36.58	46
IRC_IRT_c90466	120	XP_002960029.1	0.0	GO:0016740	74.00	42.74	39
IRC_IRT_c90489	103	KEH21177.1	0.0	GO:0050661	82.00	52.37	34
IRC_IRT_c90499	114	ACJ24144.1	0.0	-	66.00	41.97	36
IRC_IRT_c90535	118	XP_003637271.1	0.0	GO:0016747	68.00	44.28	38
IRC_IRT_c90540	105	XP_001419626.1	0.0	-	65.00	36.97	35
IRC_IRT_c90542	113	AAX96864.1	0.0	-	66.00	44.28	36
IRC_IRT_c90575	106	XP_005847241.1	0.0	GO:0004402	100.00	85.50	35
IRC_IRT_c90576	127	XP_002536587.1	0.0	-	65.00	37.74	35
IRC_IRT_c90611	135	BAD17681.1	0.3	-	55.00	33.88	36

IRC_IRT_c90621	115	XP_002534724.1	0.0	GO:0016757	75.00	51.60	36
IRC_IRT_c90652	124	EAZ08702.1	0.0	GO:0003676	100.00	83.96	41
IRC_IRT_c90670	128	XP_002536336.1	0.0	-	68.00	46.60	38
IRC_IRT_c90773	166	XP_002539090.1	0.0	-	70.00	43.13	41
IRC_IRT_c90776	122	XP_001783174.1	0.0	-	62.00	39.28	40
IRC_IRT_c90858	168	ABA96979.1	0.0	GO:0005739	71.00	67.78	53
IRC_IRT_c90924	144	KIZ00241.1	0.0	GO:0005829	80.00	74.71	46
IRC_IRT_c90962	109	KDD72511.1	0.0	GO:0071704	88.00	59.31	34
IRC_IRT_c90977	127	XP_002539729.1	0.0	-	61.00	44.28	42
IRC_IRT_c91153	125	EDQ48133.1	0.0	GO:0098655	89.00	66.24	37
IRC_IRT_c91166	111	XP_002976288.1	0.0	-	62.00	40.05	35
IRC_IRT_c91251	109	XP_006589081.1	0.0	GO:0044765	76.00	48.91	34
IRC_IRT_c91254	123	XP_002535931.1	0.0	-	82.00	45.44	34
IRC_IRT_c91293	107	AFF27500.1	0.0	GO:0004386	82.00	58.92	35
IRC_IRT_c91332	111	XP_002536523.1	0.0	-	62.00	38.89	37
IRC_IRT_c91544	123	XP_011014247.1	0.0	-	64.00	38.12	37
IRC_IRT_c91556	183	ACE96788.1	0.0	-	57.00	38.51	47
IRC_IRT_c91564	121	XP_002540260.1	0.0	GO:0003824	70.00	46.98	34
IRC_IRT_c91597	112	XP_004253340.1	0.0	-	81.00	53.53	37
IRC_IRT_c91611	137	XP_002535357.1	0.0	-	65.00	41.59	35
IRC_IRT_c91663	173	XP_004958938.1	0.4	-	57.00	33.88	47
IRC_IRT_c91667	112	EMT03504.1	0.0	-	63.00	45.05	36
IRC_IRT_c91694	132	AAX94982.1	0.0	GO:0016023	100.00	63.93	41
IRC_IRT_c91730	134	CCO15306.1	0.0	-	70.00	43.51	34
IRC_IRT_c91786	129	KIY94406.1	0.0	GO:0046872	81.00	53.53	38
IRC_IRT_c91851	152	XP_001689885.1	0.9	-	54.00	32.73	48
IRC_IRT_c91864	126	CDY52258.1	0.0	GO:0071704	68.00	48.91	41
IRC_IRT_c92036	120	XP_002538274.1	0.0	GO:0009987	71.00	46.98	38
IRC_IRT_c92087	142	KDD74350.1	0.0	GO:0006457	80.00	58.54	40
IRC_IRT_c92157	123	XP_002536133.1	0.1	-	58.00	34.65	34
IRC_IRT_c92186	146	XP_006304964.1	0.6	-	60.00	33.11	35
IRC_IRT_c92189	107	AAK16174.1	0.0	-	100.00	75.10	35
IRC_IRT_c92277	111	XP_011016126.1	0.0	GO:0044763	81.00	57.77	37

IRC_IRT_c92278	148	NP_001059097.1	0.0	-	61.00	39.28	44
IRC_IRT_c92294	138	XP_001787087.1	0.1	-	51.00	34.65	43
IRC_IRT_c92335	118	ACX71633.1	0.0	GO:0046872	84.00	62.00	39
IRC_IRT_c92367	109	AHZ64051.1	0.0	GO:0000105	77.00	55.07	35
IRC_IRT_c92504	135	EHK62735.1	0.0	GO:0003677	91.00	59.69	37
IRC_IRT_c92521	108	AAU95561.1	0.0	-	76.00	38.12	34
IRC_IRT_c92525	121	XP_002536800.1	0.0	GO:0006259	76.00	44.67	34
IRC_IRT_c92541	115	XP_001703358.1	1.0	-	55.00	31.96	36
IRC_IRT_c92619	149	XP_010229437.1	0.0	-	51.00	37.35	49
IRC_IRT_c92632	117	ABF95218.1	0.0	-	73.00	47.75	38
IRC_IRT_c92711	120	XP_003591924.1	0.1	-	58.00	35.04	34
IRC_IRT_c92724	123	XP_002535989.1	0.3	-	58.00	33.50	34
IRC_IRT_c92728	108	NP_001051102.1	0.0	GO:0016192	100.00	72.40	36
IRC_IRT_c92767	145	XP_004512089.1	0.5	-	59.00	33.50	37
IRC_IRT_c92827	131	XP_011016603.1	0.0	GO:0016624	76.00	47.37	34
IRC_IRT_c92869	125	XP_002954716.1	0.0	-	70.00	39.28	40
IRC_IRT_c92877	110	EEC66960.1	0.0	-	62.00	37.74	37
IRC_IRT_c92888	140	XP_010024342.1	0.1	-	55.00	34.27	36
IRC_IRT_c92899	141	YP_003433844.1	0.0	GO:0046961	100.00	106.69	47
IRC_IRT_c92922	134	XP_006425654.1	0.1	-	59.00	35.04	37
IRC_IRT_c92925	146	XP_002967382.1	0.0	-	59.00	38.12	47
IRC_IRT_c92944	103	AAT77897.1	1.0	-	52.00	30.03	34
IRC_IRT_c92974	112	BAC83471.1	0.0	-	70.00	40.05	37
IRC_IRT_c93129	125	EEE52319.1	0.0	-	60.00	40.82	41
IRC_IRT_c93177	118	EAY90558.1	0.0	GO:0005829	73.00	52.76	38
IRC_IRT_c93232	114	CBI35440.3	0.0	GO:0006457	94.00	59.69	35
IRC_IRT_c93244	117	EAZ01331.1	0.0	GO:0016023	100.00	48.91	37
IRC_IRT_c93261	123	CAE01944.2	0.0	GO:0005739	97.00	74.33	35
IRC_IRT_c93285	123	AAM01057.1	0.1	-	61.00	34.27	36
IRC_IRT_c93287	103	XP_002537784.1	0.0	-	73.00	35.81	34
IRC_IRT_c93304	112	EMS47697.1	0.0	GO:0009067	71.00	47.37	39
IRC_IRT_c93305	117	ABA98328.2	0.0	-	58.00	38.12	36
IRC_IRT_c93329	117	XP_002536180.1	0.0	-	63.00	36.58	36

IRC_IRT_c93355	139	AFW60474.1	0.1	-	63.00	35.04	36
IRC_IRT_c93362	154	XP_002537406.1	0.4	-	65.00	33.50	43
IRC_IRT_c93381	143	BAD27688.1	0.0	GO:0005488	80.00	70.86	46
IRC_IRT_c93411	122	XP_001417438.1	0.1	-	56.00	34.65	39
IRC_IRT_c93428	122	EMT11891.1	0.2	-	59.00	34.27	42
IRC_IRT_c93551	112	XP_004506960.1	0.0	-	76.00	46.60	42
IRC_IRT_c93602	115	XP_001758625.1	0.0	GO:0044699	78.00	55.45	38
IRC_IRT_c93632	159	KDO39741.1	0.0	-	50.00	35.42	52
IRC_IRT_c93711	109	AAB23731.1	0.0	GO:0006468	85.00	53.53	34
IRC_IRT_c93726	127	BAD16260.1	0.7	-	63.00	30.80	38
IRC_IRT_c93730	114	YP_009106495.1	0.1	-	62.00	34.27	37
IRC_IRT_c93758	143	YP_001019064.1	0.2	-	66.00	30.80	36
IRC_IRT_c93760	159	KDO49733.1	0.1	-	57.00	33.88	47
IRC_IRT_c93825	104	BAD30139.1	0.0	-	94.00	65.08	34
IRC_IRT_c93840	165	XP_002537447.1	0.0	-	63.00	47.37	49
IRC_IRT_c93966	123	BAC79815.1	0.0	-	72.00	39.28	40
IRC_IRT_c93969	107	XP_002536668.1	0.1	-	60.00	35.42	35
IRC_IRT_c93975	118	BAD05188.1	0.0	GO:0005739	88.00	55.45	34
IRC_IRT_c94014	123	EXC26217.1	0.9	-	60.00	30.03	38
IRC_IRT_c94058	124	EAZ07075.1	0.0	GO:0010287	97.00	78.18	41
IRC_IRT_c94064	103	BAK02440.1	0.0	GO:0020037	88.00	42.74	34
IRC_IRT_c94095	137	XP_011003534.1	0.8	-	51.00	32.73	35
IRC_IRT_c94096	109	XP_002537423.1	0.3	-	69.00	32.34	36
IRC_IRT_c94110	125	ABR26179.1	0.0	-	56.00	35.04	41
IRC_IRT_c94220	149	KGN54707.1	0.0	-	63.00	43.13	44
IRC_IRT_c94259	113	NP_001044554.1	0.0	-	64.00	36.19	37
IRC_IRT_c94337	122	BAD03164.1	0.5	-	58.00	32.34	34
IRC_IRT_c94341	112	EEC74143.1	0.0	GO:0006950	100.00	76.64	37
IRC_IRT_c94353	138	XP_009137892.1	0.5	-	61.00	33.50	36
IRC_IRT_c94383	109	EEE53437.1	0.0	GO:0043531	100.00	83.96	36
IRC_IRT_c94436	110	XP_009394356.1	0.4	-	52.00	33.11	40
IRC_IRT_c94526	133	XP_001417046.1	0.0	GO:0042558	90.00	72.02	44
IRC_IRT_c94584	184	EMT07488.1	0.0	-	50.00	40.05	52

IRC_IRT_c94614	114	EEC77198.1	0.0	GO:0007059	94.00	73.17	38
IRC_IRT_c94617	106	EAZ39023.1	0.0	GO:0005739	100.00	72.79	35
IRC_IRT_c94639	103	EEC67918.1	0.0	-	100.00	75.87	34
IRC_IRT_c94654	119	XP_004970544.1	0.4	-	66.00	33.50	36
IRC_IRT_c94790	130	NP_001051874.1	0.0	-	95.00	37.74	40
IRC_IRT_c94810	114	NP_001058748.1	0.0	GO:0009062	100.00	76.26	34
IRC_IRT_c94815	120	XP_005842992.1	0.0	-	66.00	40.43	36
IRC_IRT_c94833	125	XP_009802753.1	0.0	-	73.00	43.90	41
IRC_IRT_c94921	143	XP_002535707.1	0.0	-	64.00	40.05	42
IRC_IRT_c94934	115	BAD62050.1	0.0	-	59.00	34.27	37
IRC_IRT_c94977	118	CAD40468.2	0.0	GO:0008270	100.00	77.41	35
IRC_IRT_c94984	123	ABF95969.1	0.0	GO:0005488	70.00	54.30	41
IRC_IRT_c95058	143	XP_003084178.1	0.0	GO:0006099	86.00	61.23	38
IRC_IRT_c95128	113	XP_002539849.1	0.0	-	66.00	41.97	36
IRC_IRT_c95147	102	EEE65987.1	0.0	-	76.00	42.36	34
IRC_IRT_c95165	114	XP_002318707.2	0.0	-	71.00	38.51	35
IRC_IRT_c95194	156	AFW88298.1	0.8	-	62.00	32.73	45
IRC_IRT_c95195	107	XP_002538377.1	0.0	-	77.00	43.13	35
IRC_IRT_c95217	116	KIY93055.1	0.0	-	63.00	39.28	36
IRC_IRT_c95245	103	DAA63101.1	0.0	GO:0009678	100.00	70.09	34
IRC_IRT_c95368	105	KDD74872.1	0.0	-	69.00	38.12	36
IRC_IRT_c95410	130	XP_008657699.1	0.0	GO:0003676	91.00	70.09	37
IRC_IRT_c95412	183	XP_002535205.1	0.5	-	55.00	33.88	45
IRC_IRT_c95442	108	XP_002534896.1	0.0	GO:0006260	91.00	64.31	36
IRC_IRT_c95476	158	KJB76688.1	0.0	-	61.00	36.58	36
IRC_IRT_c95505	114	XP_002537608.1	0.0	-	65.00	39.28	43
IRC_IRT_c95512	146	XP_010049706.1	0.1	-	51.00	35.42	52
IRC_IRT_c95514	110	XP_002974404.1	0.0	-	65.00	38.51	35
IRC_IRT_c95526	199	XP_004958081.1	0.6	-	56.00	33.88	50
IRC_IRT_c95568	113	ADE34464.1	0.0	GO:0034357	72.00	47.75	37
IRC_IRT_c95570	123	EXB89998.1	0.0	-	74.00	44.28	35
IRC_IRT_c95610	142	XP_002507787.1	0.0	GO:0016616	74.00	61.23	47
IRC_IRT_c95618	168	EXC01914.1	0.0	-	56.00	40.43	41

IRC_IRT_c95621	172	YP_001152204.1	0.0	-	54.00	34.65	46
IRC_IRT_c95638	139	XP_002539853.1	0.1	-	68.00	35.81	38
IRC_IRT_c95659	272	XP_003638717.1	0.0	-	63.00	38.51	36
IRC_IRT_c95680	225	XP_009805057.1	0.2	-	67.00	33.11	37
IRC_IRT_c95700	118	XP_003057270.1	0.3	-	73.00	32.34	34
IRC_IRT_c95709	218	XP_002509990.1	0.4	-	64.00	32.34	34
IRC_IRT_c95743	157	XP_007154367.1	0.2	-	62.00	32.34	35
IRC_IRT_c95747	301	NP_001170376.1	0.4	-	51.00	33.11	60
IRC_IRT_c95760	202	AGZ19352.1	0.0	-	71.00	40.43	35
IRC_IRT_c95783	209	EXC01915.1	0.0	-	54.00	38.89	50
IRC_IRT_c95786	248	XP_003608262.1	0.0	-	64.00	43.13	45
IRC_IRT_c95793	116	YP_009104924.1	0.0	-	60.00	37.35	38
IRC_IRT_c95819	121	XP_006575404.1	0.8	-	54.00	31.57	35
IRC_IRT_c95825	217	XP_003616487.1	0.1	-	59.00	36.58	52
IRC_IRT_c95846	111	XP_010248290.1	0.0	GO:0006457	81.00	61.62	37
IRC_IRT_c95852	105	XP_006648129.1	0.0	GO:0004715	100.00	75.49	34
IRC_IRT_c95872	140	XP_003056665.1	0.2	-	70.00	33.88	40
IRC_IRT_c95875	167	XP_009350076.1	0.0	GO:0010467	79.00	79.34	49
IRC_IRT_c95889	115	XP_010911665.1	0.0	-	89.00	59.31	37
IRC_IRT_c95913	172	EEC66489.1	0.8	-	59.00	33.11	52
IRC_IRT_c95935	181	XP_003637074.1	0.0	-	49.00	39.66	55
IRC_IRT_c95965	301	CDX71648.1	0.0	-	71.00	65.86	56
IRC_IRT_c95990	159	XP_002536816.1	0.0	-	59.00	38.89	52
IRC_IRT_c96032	110	XP_003614380.1	0.1	-	71.00	35.04	35
IRC_IRT_c96041	194	YP_358636.1	0.0	GO:0009536	73.00	49.68	34
IRC_IRT_c96048	162	KDD75042.1	0.0	GO:0016651	75.00	58.15	44
IRC_IRT_c96075	119	XP_009350075.1	0.0	GO:0044249	84.00	62.77	39
IRC_IRT_c96080	129	XP_002538927.1	0.8	-	59.00	31.96	37
IRC_IRT_c96092	168	YP_007890199.1	0.0	-	62.00	36.97	43
IRC_IRT_c96102	131	XP_006648003.1	0.0	-	68.00	52.76	41
IRC_IRT_c96105	144	XP_002539867.1	0.0	-	76.00	45.05	34
IRC_IRT_c96107	119	CDP02290.1	0.0	-	60.00	38.12	43
IRC_IRT_c96127	108	XP_004968631.1	0.7	-	61.00	32.34	34

IRC_IRT_c96131	148	EAZ40137.1	0.0	GO:0016023	94.00	71.25	34
IRC_IRT_c96147	109	XP_009350065.1	0.0	GO:0009987	91.00	64.70	36
IRC_IRT_c96156	109	EAY98753.1	0.0	GO:0006468	100.00	73.56	36
IRC_IRT_c96169	140	XP_010314982.1	0.0	-	61.00	39.28	44
IRC_IRT_c96196	122	KEH24604.1	0.3	-	64.00	33.88	37
IRC_IRT_c96223	136	XP_006287338.1	0.3	-	60.00	33.88	35
IRC_IRT_c96231	177	KJB31427.1	0.0	GO:0003735	77.00	43.90	35
IRC_IRT_c96240	146	XP_007201468.1	0.1	-	54.00	33.50	44
IRC_IRT_c96249	124	XP_006386952.1	0.7	-	65.00	32.34	38
IRC_IRT_c96255	106	XP_001702513.1	0.1	-	64.00	35.42	34
IRC_IRT_c96265	115	XP_006290161.1	0.0	GO:0003824	73.00	46.21	34
IRC_IRT_c96267	281	KJB31094.1	0.0	-	70.00	45.44	34
IRC_IRT_c96277	108	KIY97548.1	0.0	GO:0005739	83.00	60.08	36
IRC_IRT_c96280	114	KJB06833.1	0.2	-	60.00	31.57	35
IRC_IRT_c96316	141	XP_002540438.1	0.1	-	52.00	34.65	38
IRC_IRT_c96320	124	XP_003059021.1	0.0	GO:0016226	82.00	53.91	34
IRC_IRT_c96349	138	KJB14078.1	0.0	-	76.00	39.66	38
IRC_IRT_c96364	119	XP_008244621.1	0.0	-	63.00	36.97	36
IRC_IRT_c96412	127	XP_009340927.1	0.5	-	53.00	33.11	41
IRC_IRT_c96425	171	XP_002975510.1	0.2	-	56.00	34.65	51
IRC_IRT_c96433	120	XP_002537847.1	0.0	-	65.00	46.98	38
IRC_IRT_c96475	139	AII99892.1	0.0	-	77.00	45.82	35
IRC_IRT_c96491	112	XP_002540302.1	0.0	-	79.00	52.37	34
IRC_IRT_c96508	125	XP_002537211.1	0.0	-	100.00	83.96	41
IRC_IRT_c96546	187	XP_007099561.1	0.0	-	88.00	64.70	34
IRC_IRT_c96558	218	XP_008447683.1	0.0	-	64.00	35.81	34
IRC_IRT_c96572	169	BAD10229.1	0.0	GO:0005739	92.00	62.39	38
IRC_IRT_c96578	108	XP_002535641.1	0.0	GO:0044238	82.00	49.29	35
IRC_IRT_c96592	109	AFK29285.1	0.0	GO:0044237	74.00	47.75	35
IRC_IRT_c96648	175	XP_009350076.1	0.0	GO:0008270	86.00	96.67	58
IRC_IRT_c96656	209	XP_003638717.1	0.0	-	62.00	57.38	54
IRC_IRT_c96660	146	KFK38421.1	0.0	-	64.00	40.82	48
IRC_IRT_c96679	149	XP_004298671.1	0.1	-	62.00	30.42	37

IRC_IRT_c96708	170	ABF93819.1	0.0	-	65.00	70.48	63
IRC_IRT_c96724	146	CDY20046.1	1.0	-	52.00	32.73	46
IRC_IRT_c96782	145	CDP18239.1	0.1	-	69.00	36.19	36
IRC_IRT_c96839	117	XP_005647851.1	0.2	-	62.00	34.65	35
IRC_IRT_c96886	121	XP_011097853.1	0.1	-	58.00	35.04	34
IRC_IRT_c96927	189	XP_002538424.1	0.0	-	55.00	36.58	52
IRC_IRT_c96960	114	NP_001059204.1	0.0	GO:0006633	97.00	72.02	38
IRC_IRT_c96997	108	AIC77166.1	0.0	GO:0008152	79.00	50.45	34
IRC_IRT_c97059	111	KFM25302.1	0.0	-	75.00	47.37	36
IRC_IRT_c97060	166	EAY82765.1	0.0	GO:0016020	92.00	73.56	40
IRC_IRT_c97070	112	XP_005649017.1	0.0	GO:0055114	89.00	59.31	37
IRC_IRT_c97115	136	XP_010905271.1	0.2	-	61.00	33.50	39
IRC_IRT_c97146	194	XP_010276328.1	0.1	-	50.00	34.27	51
IRC_IRT_c97179	114	XP_008440613.1	0.0	GO:0005488	70.00	44.67	37
IRC_IRT_c97212	134	XP_007013746.1	0.2	-	55.00	34.27	38
IRC_IRT_c97336	115	XP_003061625.1	0.2	-	56.00	34.27	37
IRC_IRT_c97340	133	AAS93431.1	1.0	-	57.00	32.34	38
IRC_IRT_c97424	120	BAD17238.1	0.0	GO:0003677	100.00	49.29	38
IRC_IRT_c97443	109	XP_002537616.1	0.2	-	67.00	33.88	34
IRC_IRT_c97464	124	XP_002313792.2	0.1	-	55.00	35.04	36
IRC_IRT_c97466	107	EEC76774.1	0.0	GO:0016020	65.00	45.05	38
IRC_IRT_c97497	154	CAE03637.1	0.0	-	66.00	39.66	36
IRC_IRT_c97504	151	ACQ99195.1	0.0	GO:0033737	80.00	72.02	50
IRC_IRT_c97523	114	KFM23777.1	0.0	-	61.00	36.58	34
IRC_IRT_c97541	111	XP_005843223.1	0.0	-	68.00	36.19	38
IRC_IRT_c97603	110	XP_002975488.1	0.0	-	65.00	40.05	38
IRC_IRT_c97628	118	ACS68699.1	0.0	GO:0006499	76.00	51.60	39
IRC_IRT_c97687	145	ABA93998.1	0.7	-	53.00	33.11	45
IRC_IRT_c97719	133	XP_011014723.1	0.0	-	69.00	46.21	39
IRC_IRT_c97769	146	XP_002501209.1	0.7	-	64.00	32.73	34
IRC_IRT_c97777	146	XP_010279416.1	0.1	-	63.00	35.81	36
IRC_IRT_c97790	105	XP_006643929.1	0.0	GO:0016301	68.00	43.90	35
IRC_IRT_c97801	105	EEE52320.1	0.0	GO:0055085	79.00	54.30	34

IRC_IRT_c97840	144	XP_002535126.1	0.0	-	75.00	40.82	36
IRC_IRT_c97851	103	BAC80025.1	0.0	-	59.00	39.66	42
IRC_IRT_c97879	138	XP_001786872.1	0.0	GO:0004489	94.00	60.85	34
IRC_IRT_c97907	113	XP_006344829.1	0.7	-	73.00	32.34	34
IRC_IRT_c97941	123	KEH32477.1	0.2	-	63.00	33.88	38
IRC_IRT_c97951	121	KFM28072.1	0.0	-	73.00	38.51	38
IRC_IRT_c98072	104	AAV31383.1	0.0	GO:0003676	97.00	73.94	34
IRC_IRT_c98092	134	XP_008455592.1	0.1	-	63.00	35.42	36
IRC_IRT_c98102	147	CAB53479.1	0.6	-	55.00	33.11	38
IRC_IRT_c98158	128	XP_009116345.1	0.7	-	52.00	31.96	36
IRC_IRT_c98165	123	XP_003056137.1	0.0	GO:0006139	75.00	48.52	37
IRC_IRT_c98174	118	XP_004982183.1	0.0	GO:0080041	88.00	64.31	36
IRC_IRT_c98286	152	EAZ09588.1	0.0	GO:0050662	92.00	73.17	42
IRC_IRT_c98398	118	XP_002535812.1	0.0	-	64.00	40.05	39
IRC_IRT_c98412	135	XP_002539337.1	0.0	-	63.00	39.66	38
IRC_IRT_c98489	117	XP_002534501.1	0.0	GO:0032440	86.00	56.61	38
IRC_IRT_c98494	103	EAY95084.1	0.0	GO:0016021	100.00	69.32	34
IRC_IRT_c98502	125	XP_009381839.1	0.0	GO:0008094	82.00	65.86	40
IRC_IRT_c98571	142	EEE69080.1	0.6	-	61.00	33.11	39
IRC_IRT_c98582	105	KDD72146.1	0.0	GO:0016491	79.00	44.28	34
IRC_IRT_c98634	138	NP_001152732.1	0.3	-	61.00	33.88	34
IRC_IRT_c98661	145	NP_001275120.1	0.0	GO:0044763	77.00	62.39	4.80E+01
IRC_IRT_c98730	147	EAY94268.1	0.0	GO:0005737	71.00	45.82	38
IRC_IRT_c98736	135	XP_011016735.1	0.0	-	74.00	36.58	39
IRC_IRT_c98749	113	KIZ05676.1	0.2	-	61.00	34.27	34
IRC_IRT_c98759	122	XP_002539271.1	0.2	-	68.00	33.50	35
IRC_IRT_c98849	138	XP_005644612.1	0.4	-	58.00	33.88	36
IRC_IRT_c98855	115	XP_002535565.1	0.0	-	71.00	38.12	38
IRC_IRT_c98915	106	EEE60353.1	0.0	GO:0048544	100.00	75.49	35
IRC_IRT_c98981	243	CDY09718.1	0.0	-	57.00	38.12	42
IRC_IRT_c98987	114	XP_011016510.1	0.0	-	78.00	53.14	37
IRC_IRT_c98994	121	XP_011075917.1	0.0	-	64.00	45.82	37
IRC_IRT_c99018	112	XP_004495926.1	0.4	-	55.00	33.11	49

IRC_IRT_c99108	152	CAE02304.2	0.0	GO:0003676	75.00	63.16	45
IRC_IRT_c99127	126	XP_002536801.1	0.0	-	86.00	62.00	38
IRC_IRT_c99152	105	NP_001151894.1	0.0	-	61.00	35.81	36
IRC_IRT_c99159	107	BAC84856.1	0.0	-	74.00	38.89	35
IRC_IRT_c99191	124	ABA98340.1	0.0	GO:0008171	97.00	72.79	37
IRC_IRT_c99339	138	XP_009351044.1	0.0	-	68.00	45.05	38
IRC_IRT_c99347	137	XP_010906776.1	0.0	GO:0009536	78.00	75.49	42
IRC_IRT_c99378	123	EAY93456.1	0.0	GO:0008152	78.00	60.46	38
IRC_IRT_c99412	143	ABA99961.1	0.4	-	47.00	33.88	42
IRC_IRT_c99515	104	XP_002537142.1	0.0	-	67.00	38.12	34
IRC_IRT_c99593	156	ABA94257.1	0.0	GO:0005739	83.00	53.53	37
IRC_IRT_c99673	186	ABG75919.1	0.9	-	63.00	33.11	38
IRC_IRT_c99814	118	XP_003081206.1	0.7	-	60.00	32.73	41
IRC_IRT_c99820	108	XP_002537280.1	0.0	-	71.00	43.51	35
IRC_IRT_c99878	156	XP_009351046.1	0.0	GO:0071704	77.00	69.71	48
IRC_IRT_c99904	120	BAF49677.1	0.1	-	61.00	35.81	42
IRC_IRT_c99930	105	XP_002517898.1	0.0	GO:0005739	82.00	57.77	34
IRC_IRT_c99933	184	DAA45937.1	0.0	-	71.00	50.06	38
IRC_IRT_c100021	117	BAD94946.1	0.0	GO:0050897	66.00	44.28	36
IRC_IRT_c100077	144	CAN72374.1	0.0	GO:0016740	65.00	50.45	44
IRC_IRT_c100120	128	EAZ36460.1	0.0	-	83.00	67.40	42
IRC_IRT_c100143	118	XP_002536843.1	0.0	-	84.00	58.15	39
IRC_IRT_c100267	130	XP_011079703.1	0.0	GO:0015937	82.00	54.68	34
IRC_IRT_c100345	106	BAD03496.1	0.0	GO:0005739	73.00	43.90	34
IRC_IRT_c100374	136	EEC79493.1	0.0	GO:0016740	76.00	52.37	34
IRC_IRT_c100414	127	ABA94840.1	0.0	-	83.00	65.08	42
IRC_IRT_c100423	121	XP_002540481.1	0.0	-	61.00	36.58	36
IRC_IRT_c100593	120	KFK40289.1	0.9	-	72.00	32.34	37
IRC_IRT_c100631	115	XP_002969830.1	0.0	GO:0008152	77.00	49.29	35
IRC_IRT_c100678	137	XP_002537611.1	0.0	GO:0006281	82.00	70.86	45
IRC_IRT_c100713	127	YP_002600949.1	0.0	GO:0003899	87.00	62.00	41
IRC_IRT_c100721	126	XP_002540481.1	0.0	-	71.00	48.91	38
IRC_IRT_c100771	130	XP_006604494.1	0.9	-	50.00	32.34	38

IRC_IRT_c100899	158	AAM19056.1	0.0	-	68.00	45.82	48
IRC_IRT_c100916	120	XP_009407720.1	0.0	GO:0003824	66.00	58.15	51
IRC_IRT_c101001	122	XP_006422293.1	0.0	GO:0043231	70.00	43.51	37
IRC_IRT_c101002	157	XP_006300443.1	0.0	GO:0016787	60.00	47.37	51
IRC_IRT_c101123	126	EEC76404.1	0.0	GO:0016020	82.00	48.91	40
IRC_IRT_c101135	144	KEH15587.1	0.0	GO:0042578	76.00	68.17	47
IRC_IRT_c101140	104	AAT69603.1	0.0	GO:0009536	70.00	41.20	34
IRC_IRT_c101316	135	KDD74581.1	0.0	GO:0044237	72.00	61.62	43
IRC_IRT_c101334	135	AFW75985.1	0.0	GO:0005840	100.00	95.90	44
IRC_IRT_c101386	145	KIY99261.1	0.1	-	63.00	35.42	38
IRC_IRT_c101411	119	XP_002986454.1	0.0	GO:0001510	83.00	59.31	37
IRC_IRT_c101447	105	AAX92771.1	0.3	-	61.00	33.50	36
IRC_IRT_c101454	175	XP_003074741.1	0.0	-	62.00	52.37	51
IRC_IRT_c101485	109	XP_005648383.1	0.0	GO:0009753	66.00	45.05	36
IRC_IRT_c101530	112	XP_003057154.1	0.0	-	76.00	39.28	34
IRC_IRT_c101549	207	XP_002488950.1	0.0	-	97.00	81.65	40
IRC_IRT_c101601	116	XP_009351043.1	0.1	-	67.00	34.65	37
IRC_IRT_c101607	129	XP_002864104.1	0.0	GO:0009560	78.00	54.30	41
IRC_IRT_c101627	173	XP_002538834.1	0.0	GO:0007165	63.00	42.36	49
IRC_IRT_c101631	105	XP_002536366.1	0.0	-	85.00	53.14	34
IRC_IRT_c101750	123	XP_002500964.1	0.3	-	52.00	33.88	40
IRC_IRT_c101778	114	EEE69547.1	0.0	GO:0016023	94.00	77.80	38
IRC_IRT_c101781	109	EAY82190.1	0.0	GO:0046872	73.00	50.45	38
IRC_IRT_c101891	115	AIY30179.1	0.2	-	60.00	34.27	35
IRC_IRT_c101903	128	XP_006653780.1	0.0	GO:0030942	90.00	72.40	40
IRC_IRT_c102008	102	EMT20257.1	0.1	-	60.00	35.04	35
IRC_IRT_c102022	116	XP_003084003.1	0.0	-	74.00	48.91	35
IRC_IRT_c102050	143	KGN53909.1	0.0	GO:0046872	72.00	55.84	47
IRC_IRT_c102053	102	XP_002459967.1	0.0	GO:0004072	79.00	46.60	34
IRC_IRT_c102074	110	AAL83340.1	0.0	-	81.00	55.07	3.70E+01
IRC_IRT_c102148	172	XP_002537860.1	0.0	GO:0050794	75.00	66.63	56
IRC_IRT_c102195	108	XP_003062890.1	0.0	-	68.00	42.74	35
IRC_IRT_c102263	128	XP_002439513.1	0.8	-	62.00	32.34	35

IRC_IRT_c102264	105	XP_002967320.1	0.0	-	67.00	36.58	34
IRC_IRT_c102272	122	BAC20735.1	0.0	-	91.00	45.05	37
IRC_IRT_c102296	105	EEC70905.1	0.0	-	68.00	39.28	38
IRC_IRT_c102319	105	XP_010943758.1	0.0	GO:1901363	70.00	49.29	34
IRC_IRT_c102359	145	XP_002526961.1	0.4	-	52.00	33.88	34
IRC_IRT_c102382	117	EEC76774.1	0.0	GO:0006810	72.00	47.75	37
IRC_IRT_c102392	116	XP_001786874.1	0.1	-	69.00	35.04	36
IRC_IRT_c102456	121	KFK25298.1	0.4	-	53.00	33.50	43
IRC_IRT_c102459	102	BAK00396.1	0.0	GO:0044262	82.00	51.60	34
IRC_IRT_c102557	126	BAC15797.1	0.0	GO:0008233	95.00	69.71	40
IRC_IRT_c102582	133	XP_001762156.1	0.0	-	64.00	43.90	39
IRC_IRT_c102584	103	EAZ01302.1	0.0	GO:0005975	97.00	59.31	34
IRC_IRT_c102616	104	AAM19047.1	0.0	GO:0004523	94.00	67.78	34
IRC_IRT_c102632	120	XP_002537020.1	0.0	GO:0097159	69.00	43.13	39
IRC_IRT_c102644	114	CAR66469.1	0.5	-	60.00	30.80	38
IRC_IRT_c102668	111	AAX96123.1	0.0	-	85.00	58.15	34
IRC_IRT_c102726	109	NP_001174774.1	0.0	GO:0020037	100.00	75.49	3.60E+01
IRC_IRT_c102897	112	BAC83212.1	0.1	-	64.00	34.27	34
IRC_IRT_c102918	121	XP_006581801.1	0.9	-	58.00	32.34	34
IRC_IRT_c102934	130	XP_006353594.1	0.5	-	64.00	33.50	37
IRC_IRT_c103152	121	BAD28269.1	0.0	GO:0008152	89.00	70.09	38
IRC_IRT_c103189	112	ABA94261.1	0.0	-	67.00	38.51	37
IRC_IRT_c103221	153	BAD87150.1	0.0	GO:0008270	94.00	75.87	38
IRC_IRT_c103319	125	XP_009765598.1	0.0	GO:0004252	78.00	61.62	41
IRC_IRT_c103324	107	XP_009418492.1	0.0	-	80.00	41.20	35
IRC_IRT_c103369	128	XP_006307171.1	0.9	-	55.00	32.34	34
IRC_IRT_c103389	110	KJB53954.1	0.0	GO:0043167	73.00	45.82	34
IRC_IRT_c103470	148	KDO50985.1	0.0	GO:0005488	65.00	47.75	49
IRC_IRT_c103478	171	NP_001054670.2	0.3	-	60.00	34.27	41
IRC_IRT_c103489	144	XP_009413675.1	0.0	-	62.00	47.37	43
IRC_IRT_c103507	125	CDY41274.1	0.0	GO:0006468	70.00	46.21	37
IRC_IRT_c103536	126	XP_010234205.1	0.0	GO:0009793	79.00	62.77	39
IRC_IRT_c103551	133	XP_002465730.1	0.0	GO:0046872	73.00	44.67	38

IRC_IRT_c103555	113	ABG22002.1	0.0	-	86.00	62.39	36
IRC_IRT_c103556	114	EYU32542.1	0.0	GO:0019752	72.00	42.36	37
IRC_IRT_c103582	103	EEE67903.1	0.0	GO:0008270	100.00	69.71	34
IRC_IRT_c103632	128	KDO37391.1	0.6	-	51.00	32.73	39
IRC_IRT_c103634	121	ABB47009.1	0.0	-	72.00	43.90	37
IRC_IRT_c103640	122	XP_001691491.1	0.0	GO:0003743	90.00	70.09	40
IRC_IRT_c103800	107	BAC45168.1	0.0	GO:0006629	100.00	68.55	35
IRC_IRT_c103840	121	XP_001787011.1	0.0	-	65.00	36.97	43
IRC_IRT_c103862	116	P53385.1	0.0	GO:0019557	81.00	43.13	37
IRC_IRT_c103867	123	KIY99235.1	0.0	GO:0042558	87.00	65.47	41
IRC_IRT_c103874	119	XP_008458759.1	0.0	GO:0019363	75.00	46.21	36
IRC_IRT_c103973	121	NP_001050677.1	0.0	GO:0006810	60.00	48.52	40
IRC_IRT_c103997	144	XP_002501910.1	0.0	-	59.00	37.35	37
IRC_IRT_c104057	119	XP_001775415.1	0.0	GO:0008270	89.00	55.84	38
IRC_IRT_c104260	106	XP_005650930.1	0.0	GO:0045239	79.00	48.14	34
IRC_IRT_c104277	120	XP_010486941.1	0.0	-	91.00	60.85	37
IRC_IRT_c104283	101	KIY93624.1	0.7	-	61.00	31.19	34
IRC_IRT_c104312	115	KFM29308.1	0.0	GO:0016874	88.00	60.08	36
IRC_IRT_c104405	132	EEC72312.1	1.0	-	60.00	31.96	41
IRC_IRT_c104415	106	CAE03686.2	0.0	GO:0008270	100.00	76.64	35
IRC_IRT_c104461	115	AAB81662.1	0.0	GO:0005576	100.00	78.95	38
IRC_IRT_c104469	161	XP_002536484.1	0.0	GO:0003677	74.00	65.86	51
IRC_IRT_c104574	141	XP_002539815.1	0.0	-	63.00	37.74	38
IRC_IRT_c104580	109	NP_001052035.2	0.0	GO:0043531	100.00	74.71	36
IRC_IRT_c104633	106	XP_006301317.1	0.6	-	57.00	32.34	35
IRC_IRT_c104638	118	XP_009350054.1	0.0	-	82.00	62.00	39
IRC_IRT_c104653	123	XP_009375106.1	0.0	GO:0008233	73.00	41.97	34
IRC_IRT_c104713	110	EEC79491.1	0.0	-	66.00	36.97	36
IRC_IRT_c104745	118	XP_002538218.1	0.0	-	61.00	37.35	34
IRC_IRT_c104763	135	XP_004230003.1	0.8	-	58.00	32.73	34
IRC_IRT_c104781	124	NP_001057587.1	0.0	-	53.00	35.81	39
IRC_IRT_c104830	141	XP_001756027.1	0.1	-	54.00	35.04	37
IRC_IRT_c104833	137	XP_002536306.1	0.1	-	61.00	35.42	36

IRC_IRT_c104883	122	EEE53883.1	0.0	GO:0005739	75.00	57.38	40	
IRC_IRT_c104979	166	CAA63394.1	0.0	-	75.00	37.35	48	
IRC_IRT_c104989	108	EEC77111.1	0.0	GO:0006935	91.00	55.45	35	
IRC_IRT_c105106	117	XP_002538739.1	0.0	-	77.00	44.28	36	
IRC_IRT_c105146	111	CAD37080.3	0.0	GO:0003676	100.00	50.45	35	
IRC_IRT_c105162	145	XP_002535752.1	0.0	GO:0016311	84.00	65.08	39	
IRC_IRT_c105197	129	XP_006647949.1	0.0	-	77.00	37.35	35	
IRC_IRT_c105227	138	KIZ03654.1	0.8	-	57.00	32.34	40	
IRC_IRT_c105235	133	ABN04852.1	0.2	-	51.00	34.27	39	
IRC_IRT_c105308	139	EAY90289.1	0.0	-	61.00	34.27	36	
IRC_IRT_c105423	116	EEC75475.1	0.0	GO:0003964	97.00	76.26	38	
IRC_IRT_c105437	144	XP_010245583.1	0.5	-	51.00	33.11	45	
IRC_IRT_c105497	121	ERN07643.1	0.1	-	57.00	32.34	38	
IRC_IRT_c105527	135	KDO49507.1	0.0	GO:0097159	57.00	46.21	42	
IRC_IRT_c105532	129	XP_002946078.1	0.0	GO:0016746	70.00	43.90	37	
IRC_IRT_c105594	120	EEE55944.1	0.0	GO:0010583	100.00	61.23	36	
IRC_IRT_c105613	116	XP_006294326.1	0.0	GO:0004830	91.00	62.39	34	
IRC_IRT_c105719	105	CDP15949.1	0.0	-	65.00	43.51	35	
IRC_IRT_c105734	115	ABA99980.1	0.0	GO:0050896	97.00	79.34	38	
IRC_IRT_c105775	170	XP_010314956.1	0.0	GO:0016301	88.00	52.37	44	
IRC_IRT_c105782	136	XP_001692598.1	0.0	GO:0004832	80.00	70.09	46	
IRC_IRT_c105801	107	XP_002508298.1	0.2	-	67.00	33.88	34	
IRC_IRT_c105813	107	BAD34162.1	0.0	-	88.00	59.31	35	
IRC_IRT_c105834	135	EEC77111.1	0.0	GO:0050896	74.00	45.44	39	
IRC_IRT_c105849	104	XP_002534724.1	0.0	GO:0016757	76.00	44.67	34	
IRC_IRT_c105854	110	KEH15118.1	0.0	-	58.00	36.58	34	
IRC_IRT_c105855	110	XP_002522976.1	0.2	-	55.00	32.34	36	
IRC_IRT_c105857	103	AAT47104.1	0.0	GO:0004523	97.00	73.94	34	
IRC_IRT_c105930	118	XP_004965101.1	0.6	-	57.00	32.73	38	
IRC_IRT_c105985	106	EAZ31061.1	0.0	-	71.00	50.06	35	
IRC_IRT_c106022	135	KIZ03021.1	0.0	-	69.00	38.51	36	
IRC_IRT_c106049	136	AAP52616.1	0.0	GO:0016020	78.00	58.92	41	
IRC_IRT_c106096	109	XP_001417708.1	0.0	-	61.00	37.74	36	

IRC_IRT_c106102	106	XP_006417300.1	0.0	-	62.00	38.51	45
IRC_IRT_c106117	102	AAT38073.1	0.0	GO:0005739	97.00	78.57	34
IRC_IRT_c106146	110	XP_001762925.1	0.0	GO:0006105	83.00	56.23	36
IRC_IRT_c106286	139	CCO66090.1	0.0	-	55.00	26.56	34
IRC_IRT_c106325	145	XP_010500623.1	0.4	-	58.00	33.50	34
IRC_IRT_c106453	151	XP_002438953.1	0.0	-	66.00	40.05	45
IRC_IRT_c106491	114	XP_005650418.1	0.0	-	71.00	40.43	35
IRC_IRT_c106510	120	XP_003063958.1	0.0	-	78.00	38.51	38
IRC_IRT_c106540	112	XP_011014251.1	0.0	-	74.00	43.90	35
IRC_IRT_c106643	107	AAX92931.1	0.0	-	100.00	73.17	3.40E+01
IRC_IRT_c106780	115	XP_002535112.1	0.0	-	70.00	36.58	37
IRC_IRT_c106791	109	XP_009350075.1	0.0	-	97.00	70.09	35
IRC_IRT_c106819	121	YP_009057781.1	0.0	GO:0003735	97.00	73.94	36
IRC_IRT_c106839	106	AAO37938.1	0.0	GO:0030170	97.00	71.63	3.50E+01
IRC_IRT_c106883	191	XP_002536629.1	0.3	-	47.00	34.27	61
IRC_IRT_c106988	114	XP_002540545.1	0.0	-	81.00	51.60	37
IRC_IRT_c107015	110	XP_002539881.1	0.0	GO:0005975	80.00	43.51	36
IRC_IRT_c107075	105	XP_002536317.1	0.0	GO:0050794	76.00	44.28	34
IRC_IRT_c107137	109	XP_006647603.1	0.0	GO:0003777	94.00	74.71	36
IRC_IRT_c107152	115	XP_001786346.1	0.8	-	58.00	31.19	34
IRC_IRT_c107201	136	KIY91786.1	0.0	-	61.00	37.35	39
IRC_IRT_c107225	137	XP_001785126.1	0.0	GO:0019673	75.00	60.08	41
IRC_IRT_c107317	122	EEE67873.1	0.0	-	70.00	40.43	40
IRC_IRT_c107371	126	EEE62691.1	0.0	GO:0016020	75.00	52.37	41
IRC_IRT_c107407	117	XP_009379721.1	0.4	-	52.00	33.50	36
IRC_IRT_c107469	144	XP_002976717.1	0.0	GO:0017111	75.00	52.76	45
IRC_IRT_c107525	106	EAY80224.1	0.0	-	100.00	38.89	35
IRC_IRT_c107583	107	XP_002538140.1	0.1	-	70.00	34.27	34
IRC_IRT_c107687	104	XP_001699526.1	0.0	GO:0044711	70.00	47.75	34
IRC_IRT_c107862	150	XP_001695391.1	0.4	-	61.00	33.50	34
IRC_IRT_c107866	154	CDO97685.1	0.6	-	51.00	33.11	39
IRC_IRT_c107913	110	XP_003575448.1	0.0	-	63.00	36.97	36
IRC_IRT_c107928	269	BAD27766.1	0.0	GO:0044260	82.00	83.19	67

IRC_IRT_c107932	244	XP_009388289.1	0.0	GO:0009507	84.00	64.31	38
IRC_IRT_c107933	134	XP_010024489.1	0.8	-	57.00	31.96	40
IRC_IRT_c107934	288	XP_003588337.1	0.1	-	61.00	36.19	39
IRC_IRT_c107936	196	CDY19671.1	0.0	-	59.00	37.74	44
IRC_IRT_c107937	321	EXB36957.1	0.0	GO:0009507	79.00	66.63	43
IRC_IRT_c107944	125	XP_006595194.1	0.3	-	64.00	34.27	37
IRC_IRT_c107947	385	EXC34899.1	0.0	-	59.00	43.51	44
IRC_IRT_c107950	162	AAT58826.1	0.0	GO:0009536	74.00	67.01	54
IRC_IRT_c107951	156	EEC78702.1	0.0	GO:0009536	86.00	84.73	51
IRC_IRT_c107955	198	XP_007035831.1	0.9	-	52.00	32.34	48
IRC_IRT_c107962	317	XP_003604156.1	0.2	-	54.00	35.81	44
IRC_IRT_c107965	126	ABA92518.2	0.0	-	90.00	40.05	41
IRC_IRT_c107970	230	XP_011033807.1	0.4	-	56.00	34.27	41
IRC_IRT_c107972	188	AAM74400.1	0.0	GO:0003964	100.00	93.97	41
IRC_IRT_c107980	219	AAO37842.1	0.0	GO:0003964	83.00	104.76	73
IRC_IRT_c107982	112	BAI39649.1	0.0	GO:0003676	88.00	59.31	36
IRC_IRT_c107985	175	AAM08568.1	0.0	GO:0009536	76.00	47.75	39
IRC_IRT_c107990	215	ADK60808.1	0.0	-	79.00	65.08	43
IRC_IRT_c108013	235	KJB31094.1	0.0	-	75.00	57.00	40
IRC_IRT_c108016	118	BAA83455.1	0.0	-	64.00	40.82	37
IRC_IRT_c108019	150	BAD46178.1	0.0	-	75.00	55.45	40
IRC_IRT_c108023	178	XP_003064996.1	0.0	-	66.00	53.53	45
IRC_IRT_c108048	241	KEH16146.1	0.0	-	71.00	41.97	35
IRC_IRT_c108050	136	EPS64233.1	0.0	GO:0010368	88.00	75.87	44
IRC_IRT_c108051	205	XP_010942916.1	0.5	-	52.00	33.88	42
IRC_IRT_c108064	222	XP_001702209.1	1.0	-	52.00	33.11	42
IRC_IRT_c108069	144	NP_001058395.1	0.0	GO:0008270	88.00	63.93	3.50E+01
IRC_IRT_c108070	170	AFS64411.1	0.3	-	61.00	34.27	34
IRC_IRT_c108074	112	XP_002438856.1	0.0	-	88.00	56.61	36
IRC_IRT_c108085	310	BAJ11784.1	0.0	-	89.00	66.63	37
IRC_IRT_c108087	281	EPS74505.1	0.0	-	66.00	64.70	57
IRC_IRT_c108097	134	XP_003599577.1	0.4	-	62.00	32.34	37
IRC_IRT_c108103	250	EPS74494.1	0.0	-	54.00	29.26	37

IRC_IRT_c108123	340	KEH29749.1	0.0	-	48.00	45.05	74
IRC_IRT_c108146	257	XP_003606747.1	0.2	-	50.00	34.65	62
IRC_IRT_c108151	138	EMT15683.1	0.0	-	58.00	43.51	39
IRC_IRT_c108158	178	BAD89443.1	0.0	-	64.00	43.90	37
IRC_IRT_c108163	223	CDM82334.1	0.0	GO:0009507	82.00	55.84	35
IRC_IRT_c108168	227	CDX71650.1	0.4	-	52.00	32.73	44
IRC_IRT_c108170	138	XP_006659659.1	0.0	GO:0008152	81.00	50.06	38
IRC_IRT_c108171	192	XP_003614389.1	0.0	-	68.00	57.77	41
IRC_IRT_c108221	129	KDP44481.1	0.0	-	57.00	35.81	40
IRC_IRT_c108245	112	ABA92066.2	0.0	GO:0046523	85.00	54.68	34
IRC_IRT_c108247	255	EPS74494.1	0.0	-	69.00	47.37	43
IRC_IRT_c108263	160	XP_003614391.1	0.7	-	63.00	33.11	36
IRC_IRT_c108275	178	NP_085475.1	0.0	-	60.00	52.76	55
IRC_IRT_c108292	113	XP_009350817.1	0.0	-	72.00	58.15	37
IRC_IRT_c108325	142	AAR15338.1	0.0	GO:0009536	83.00	57.00	36
IRC_IRT_c108360	241	BAJ11784.1	0.0	-	84.00	84.73	50
IRC_IRT_c108362	240	XP_006363639.1	0.0	-	72.00	63.16	48
IRC_IRT_c108370	146	EPS70027.1	0.0	-	60.00	41.97	50
IRC_IRT_c108371	215	CBI30752.3	0.7	-	53.00	33.50	43
IRC_IRT_c108393	139	XP_002534945.1	0.0	GO:0005737	95.00	71.63	40
IRC_IRT_c108407	180	AGB85039.1	0.0	-	73.00	51.60	42
IRC_IRT_c108413	176	AFK39161.1	0.0	GO:0016151	87.00	92.05	57
IRC_IRT_c108418	139	XP_008801353.1	0.0	-	64.00	36.58	34
IRC_IRT_c108421	231	BAJ11784.1	0.0	-	70.00	41.20	34
IRC_IRT_c108426	206	AGC78890.1	0.0	-	66.00	44.67	39
IRC_IRT_c108441	226	YP_001152204.1	0.1	-	58.00	34.27	34
IRC_IRT_c108448	258	BAD61237.1	0.7	-	40.00	33.88	88
IRC_IRT_c108453	248	KEH33422.1	0.0	-	70.00	57.00	47
IRC_IRT_c108457	210	XP_009388289.1	0.0	-	67.00	40.82	37
IRC_IRT_c108462	364	AGZ19352.1	0.5	-	45.00	34.27	72
IRC_IRT_c108469	215	CBI33621.3	0.9	-	45.00	31.19	55
IRC_IRT_c108472	215	XP_003608262.1	0.0	-	53.00	41.59	54
IRC_IRT_c108478	258	YP_588403.1	0.0	-	60.00	45.82	46

IRC_IRT_c108479	256	XP_003610225.1	0.1	-	67.00	36.58	46
IRC_IRT_c108481	254	ERN19620.1	0.1	-	60.00	34.65	35
IRC_IRT_c108482	201	YP_001152205.1	0.0	-	63.00	41.20	47
IRC_IRT_c108485	251	XP_003588355.1	0.2	-	63.00	35.81	38
IRC_IRT_c108497	279	XP_009388289.1	0.0	GO:0008152	89.00	65.86	39
IRC_IRT_c108508	366	XP_003588355.1	0.0	-	44.00	52.76	124
IRC_IRT_c108510	215	XP_009104253.1	0.2	-	55.00	35.04	36
IRC_IRT_c108514	265	XP_003638717.1	0.0	-	68.00	40.43	35
IRC_IRT_c108515	182	NP_817169.1	0.1	-	55.00	33.88	34
IRC_IRT_c108520	300	EPS74494.1	0.0	-	74.00	54.68	39
IRC_IRT_c108521	216	EPS74490.1	0.0	-	73.00	48.91	34
IRC_IRT_c108528	212	XP_003614273.1	0.0	-	56.00	39.66	53
IRC_IRT_c108534	213	KDP21684.1	0.1	-	66.00	35.04	36
IRC_IRT_c108535	329	XP_009388289.1	0.0	GO:0008152	89.00	67.40	39
IRC_IRT_c108542	278	XP_003637074.1	0.0	-	51.00	44.67	58
IRC_IRT_c108545	276	KGN64863.1	0.5	-	48.00	33.88	43

Supplementary Table S3 Annotated transcripts in TH

Transcript_ID	NCBI nrdb Hit	Sequence length	NCBI nrdb Hit Acc	E-Value	GO	Similarity	Bit Score	Alignment length
THC_THT_c2	senescence-associated protein	525	XP_003064993	1.19E-47	-	94	1.61E+02	88
THC_THT_c3	senescence-associated partial	740	XP_003614392	6.47E-113	-	8.50E+01	339.347	200
THC_THT_c4	protein	322	AGT17357	1.00E-24	GO:0070330	9.70E+01	96.2857	48
THC_THT_c5	senescence-associated protein	696	KEH17348	7.61E-43	-	9.40E+01	106.686	53
THC_THT_c6	hypothetical protein SORBIDRAFT_0070s002020	291	XP_002489102	4.02E-37	-	95	1.29E+02	66
THC_THT_c7	atp synthase subunit beta	489	AGV54793	2.90E-44	-	8.30E+01	155.606	106
THC_THT_c8	rrna intron-encoded homing endonuclease	335	AIQ78384	1.05E-37	GO:0004519	1.00E+02	130.568	65
THC_THT_c9	senescence-associated protein	459	XP_002450733	4.48E-64	-	92	2.01E+02	113
THC_THT_c10	senescence-associated protein	428	XP_010046011	3.51E-37	-	8.60E+01	138.658	83
THC_THT_c11	cytochrome p450 liketbp	383	XP_003614391	1.26E-30	-	6.00E+01	87.8113	87
THC_THT_c12	rrna intron-encoded homing endonuclease	426	XP_003614387	2.38E-25	-	7.60E+01	108.612	96
THC_THT_c13	rrna intron-encoded homing endonuclease	219	XP_003614385	1.25E-04	-	6.60E+01	45.0542	36
THC_THT_c14	cell wall-associated hydrolase	633	AGV54820	3.46E-58	GO:0009507	6.70E+01	197.208	182
THC_THT_c15	rrna intron-encoded homing endonuclease	307	ACG27632	4.93E-32	-	100	114.775	5.60E+01
THC_THT_c16	rrna intron-encoded homing endonuclease	369	XP_003614387	1.17E-26	-	7.30E+01	111.694	84
THC_THT_c17	senescence-associated protein	289	XP_009388207	6.96E-35	GO:0016021	7.40E+01	126.331	100
THC_THT_c18	rrna intron-encoded homing endonuclease	243	XP_009366572	4.14E-14	-	6.80E+01	73.1738	51
THC_THT_c19	protein kinase	218	AGU99598	2.18E-17	GO:0006468	1.00E+02	81.2629	41

THC_THT_c20	hypothetical protein POPTR_0008s21830g	378	XP_006380094	9.12E-10	-	86	5.89E+01	36
THC_THT_c21	chloroplast hypothetical protein	246	YP_588293	5.26E-28	GO:0005739	91	1.05E+02	56
THC_THT_c23	cell wall-associated hydrolase	438	XP_003616487	2.12E-24	GO:0016020	83	8.63E+01	53
THC_THT_c26	orf107_3 gene product	197	YP_006234312	3.23E-05	-	69	4.39E+01	46
THC_THT_c27	ycf68 protein	459	KEH17697	7.57E-40	GO:0003735	7.40E+01	137.887	100
THC_THT_c28	cell wall-associated hydrolase	771	XP_003588355	7.21E-29	GO:0009536	9.60E+01	117.857	58
THC_THT_c29	ycf68 protein	426	YP_001152205	4.27E-22	GO:0030529	78	9.05E+01	57
THC_THT_c30	unknown protein	425	BAD07868	8.16E-29	GO:0009536	1.00E+02	111.309	57
THC_THT_c33	hypothetical protein B456_007G194600	254	KJB43296	2.77E-31	GO:0009507	8.30E+01	112.464	74
THC_THT_c34	gdsl esterase lipase	382	BAD46479	1.20E-09	GO:0008152	8.80E+01	60.8474	36
THC_THT_c35	cell wall-associated partial	344	YP_173415	2.66E-29	GO:0016020	90	1.09E+02	66
THC_THT_c36	ycf68 protein	492	XP_003610227	1.09E-18	GO:0044444	7.10E+01	63.5438	45
THC_THT_c38	cell wall-associated partial	245	ABR25948	2.04E-25	GO:0005739	89	9.71E+01	56
THC_THT_c39	unknow protein	413	XP_003599577	4.46E-38	GO:0009507	73	1.34E+02	107
THC_THT_c41	cell wall-associated hydrolase	267	AGZ19352	6.86E-10	-	6.40E+01	57.7658	56
THC_THT_c42	ribosomal protein s10	230	AGC78945	1.10E-17	GO:0005739	9.50E+01	79.7221	40
THC_THT_c43	dehydration responsive protein	282	EPS74531	4.46E-08	GO:0005739	7.90E+01	52.7582	34
THC_THT_c48	senescence-associated protein	319	KEH17019	5.27E-28	-	7.50E+01	109.383	93
THC_THT_c50	senescence-associated protein	300	XP_003579816	1.38E-14	-	1.00E+02	70.8626	36
THC_THT_c51	cell wall-associated hydrolase	552	AGZ19352	1.58E-61	GO:0006869	9.10E+01	195.282	112
THC_THT_c52	plant invertase pectin methylesterase inhibitor	142	KJB20618	3.96E-04	-	6.20E+01	40.0466	35
THC_THT_c53	hypothetical protein B456_001G163100, partial	253	KJB06193	1.54E-13	-	1.00E+02	66.6254	47
THC_THT_c54	ribosomal protein s10	214	AGC78943	1.38E-17	GO:0005739	9.00E+01	78.1814	43

THC_THT_c58	hypothetical protein MTR_2g012640	219	XP_003593481	0.00411524	-	58	37.7354	41
THC_THT_c61	23s ribosomal rna	391	CDY20228	2.22E-33	GO:0016984	95	119.783	6.40E+01
THC_THT_c62	hypothetical protein (mitochondrion)	185	AGC78986	5.41E-08	-	7.60E+01	51.6026	46
THC_THT_c68	unknow protein	209	AAV44205	1.85E-32	-	9.80E+01	120.168	61
THC_THT_c69	cms-t pore-forming protein	367	CDM84611	1.41E-28	-	8.00E+01	108.997	65
THC_THT_c71	dehydration responsive protein	174	CDY45505	1.65E-07	-	8.00E+01	52.373	35
THC_THT_c73	hypothetical protein VOLCADRAFT_101171	215	XP_002959667	0.807111	-	56	32.7278	41
THC_THT_c77	Mitochondrial protein, putative	131	XP_003588355	9.85E-16	-	9.70E+01	76.2554	37
THC_THT_c78	ribulose bisphosphate carboxylase large chain	359	EPS74717	2.73E-36	-	7.90E+01	127.487	86
THC_THT_c81	dehydration responsive protein	169	BAJ11784	0.0203797	-	64	36.965	37
THC_THT_c83	senescence-associated protein	288	XP_002488946	3.20E-15	-	94	7.16E+01	36
THC_THT_c84	transposable element	173	XP_008780190	1.30E-31	GO:0004553	1.00E+02	119.398	57
THC_THT_c85	hypothetical protein PRUPE_ppa020165mg	210	XP_007212799	0.185617	-	53	35.039	49
THC_THT_c86	unknown	310	ACN36160	1.21E-25	GO:0005739	9.60E+01	98.9821	51
THC_THT_c89	t02955probable cytochrome p450 monooxygenase - maize	163	ACG27180	6.87E-22	GO:0070330	97	8.78E+01	44
THC_THT_c94	unknown protein	245	BAD07868	1.04E-16	GO:0009536	9.40E+01	77.0258	39
THC_THT_c99	senescence-associated protein	275	KEH17022	0.0971807	-	46	36.5798	77
THC_THT_c100	ribosomal protein s10	280	AGC78943	7.88E-32	GO:0005739	9.80E+01	115.931	54
THC_THT_c102	transposon en spm sub-class	269	ABA98463	8.40E-58	GO:0016020	1.00E+02	196.823	89
THC_THT_c104	rrna intron-encoded homing endonuclease	162	ABO20854	2.41E-07	-	7.50E+01	49.2914	36
THC_THT_c106	cytochrome p450 like_tbp	132	BAD26579	2.46E-06	-	6.90E+01	45.8246	42

THC_THT_c111	cell wall-associated partial	296	EXC01912	3.98E-11	-	6.60E+01	60.8474	53
THC_THT_c122	rrna intron-encoded homing endonuclease	445	XP_002507974	9.75E-12	-	6.30E+01	56.9954	52
THC_THT_c134	major facilitator superfamily domain	280	XP_003558975	0.183475	-	53	35.8094	54
THC_THT_c138	metallocarboxypeptidase inhibitor	266	EPS70027	1.16E-13	GO:0009507	6.90E+01	67.0106	55
THC_THT_c144	retrotransposon protein	357	ABR26094	0.0138842	-	61	38.1206	49
THC_THT_c145	ribosomal protein s10	106	EXB92316	7.99E-15	-	9.40E+01	70.0922	35
THC_THT_c147	hypothetical protein MTR_8g040260	266	XP_003627937	0.3807	-	61	34.2686	36
THC_THT_c149	rrna intron-encoded homing endonuclease	126	XP_003614391	0.0106938	-	66	38.1206	42
THC_THT_c152	hypothetical protein POPTR_0001s42200g	323	XP_006370394	0.00158595	-	57	40.0466	49
THC_THT_c154	unknown	144	ACN31676	0.495846	-	48	31.187	37
THC_THT_c155	hypothetical protein SORBIDRAFT_01g024221	199	XP_002467304	5.48E-07	-	65	4.89E+01	44
THC_THT_c156	cell wall-associated hydrolase	313	KJB44141	1.98E-04	-	6.70E+01	42.3578	37
THC_THT_c161	hypothetical protein MTR_5g089230	118	XP_003617224	4.38E-04	-	61	3.97E+01	34
THC_THT_c164	unknow protein	389	ERN00555	3.10E-22	GO:0009507	6.60E+01	92.4337	92
THC_THT_c165	atp synthase subunit alpha	168	AGC78945	1.09E-18	GO:0005739	8.50E+01	81.6481	49
THC_THT_c167	PREDICTED: uncharacterized protein LOC103416061	148	XP_008352560	1.30E-05	-	7.70E+01	46.2098	40
THC_THT_c169	retrotransposon ty3-gypsy subclass	411	CAI44642	3.05E-37	GO:0003676	9.50E+01	139.428	80
THC_THT_c173	transposon en spm sub-class	270	ABA97594	6.71E-35	GO:0005739	9.60E+01	132.494	65
THC_THT_c174	subtilisin-like protease	153	XP_006448873	3.91E-17	GO:0042802	89	7.51E+01	39
THC_THT_c176	hypothetical protein JCGZ_01322	312	KDP44822	0.0014847	-	59	40.817	42

THC_THT_c179	hypothetical protein M569_04734, partial	317	EPS70027	1.62E-06	-	5.50E+01	34.6538	38
THC_THT_c188	retrotransposon ty3-gypsy subclass	304	AAS98447	1.51E-21	GO:0046872	72	9.13E+01	73
THC_THT_c189	taxadien-5-alpha-ol o-acetyltransferase	264	DAA57955	3.52E-11	-	8.20E+01	61.6178	40
THC_THT_c190	dehydration responsive protein	214	BAJ11784	8.67E-05	-	6.00E+01	44.2838	66
THC_THT_c192	protein	185	XP_005842766	8.93E-15	-	82	68.9366	46
THC_THT_c199	cell wall-associated hydrolase	296	CDX71648	4.70E-10	-	7.00E+01	59.6918	50
THC_THT_c204	hypothetical protein SORBIDRAFT_01g024221	174	XP_002467304	1.50E-07	-	68	5.01E+01	45
THC_THT_c209	Ycf68	147	XP_003610227	5.59E-05	-	6.40E+01	45.0542	39
THC_THT_c211	Os02g0176300	160	NP_001046062	1.50E-17	-	97	7.63E+01	38
THC_THT_c212	cytochrome p450 liketbp	398	KDP20463	2.08E-23	-	7.50E+01	95.1301	73
THC_THT_c215	hth-type transcriptional regulator	225	XP_002537104	3.72E-04	GO:0016853	76	4.31E+01	39
THC_THT_c220	receptor-kinase isolog precursor	108	NP_001151891	0.535196	-	58	32.7278	36
THC_THT_c221	unknow protein	401	EPS74505	9.35E-20	-	6.10E+01	67.781	80
THC_THT_c227	hypothetical protein PHAVU_003G142600g	301	XP_007154729	0.00741979	-	63	33.4982	41
THC_THT_c228	Ycf68	265	XP_003610227	8.71E-04	-	5.40E+01	42.743	62
THC_THT_c231	PREDICTED: uncharacterized protein LOC103699371, partial	149	XP_008779617	5.27E-18	-	1.00E+02	83.1889	39
THC_THT_c239	hypothetical protein AMTR_s00061p00179620	365	ERN19184	2.13E-49	GO:0009507	7.90E+01	161.384	112
THC_THT_c240	cytochrome p450 like_tbp	237	XP_003614388	2.42E-06	-	7.60E+01	50.447	34

THC_THT_c241	PREDICTED: uncharacterized protein LOC103416061	173	XP_008352560	0.046037	-	58	36.1946	43
THC_THT_c243	u-box domain-containing protein 2 isoform x2	363	NP_001168873	0.172859	-	46	36.5798	73
THC_THT_c249	gag-pol polyprotein	233	NP_001175469	4.19E-28	GO:0050660	94	7.63E+01	35
THC_THT_c251	BnaCnng12640D	212	CDY45505	0.00502609	-	63	39.6614	41
THC_THT_c255	senescence-associated protein	230	BAB33421	3.66E-09	-	6.50E+01	57.3806	55
THC_THT_c259	senescence-associated partial	237	KEH17345	6.18E-06	-	8.00E+01	47.3654	35
THC_THT_c261	hypothetical protein	416	BAD69364	5.85E-31	-	9.50E+01	121.324	64
THC_THT_c262	hypothetical protein MTR_7g031847	432	KEH22088	1.24E-09	-	5.60E+01	59.6918	116
THC_THT_c267	unnamed protein product	190	CDM84612	2.20E-14	-	9.40E+01	68.5514	35
THC_THT_c269	#NAME?	157	KIY99539	2.81E-12	-	6.50E+01	65.855	58
THC_THT_c272	unknown	202	ACN31557	3.42E-13	-	9.40E+01	64.6994	34
THC_THT_c279	unknow protein	197	EYU24190	1.44E-07	-	6.10E+01	50.0618	55
THC_THT_c286	retrotransposon ty3-gypsy subclass	530	ABA96008	1.57E-61	GO:0005739	9.20E+01	202.216	107
THC_THT_c290	hypothetical protein Csa_4G652690	229	KGN55456	7.73E-16	-	6.40E+01	73.559	76
THC_THT_c297	retrotransposon ty1-copia subclass	247	EEE69435	8.42E-48	GO:0016021	9.80E+01	158.303	82
THC_THT_c299	hypothetical protein SORBIDRAFT_1211s002020	149	XP_002488956	2.14E-05	-	70	43.5134	40
THC_THT_c303	hypothetical protein CICLE_v10013352mg	351	XP_006428621	0.318655	-	50	33.8834	51
THC_THT_c307	leucine rich protein	680	ABH09321	1.00E-04	-	5.00E+01	44.2838	98
THC_THT_c309	hth-type transcriptional repressor	168	XP_002539909	0.377186	-	64	33.113	34
THC_THT_c313	retrotransposon ty3-gypsy subclass	186	ABA98123	2.14E-08	-	7.60E+01	55.4546	38

THC_THT_c315	u-box domain-containing partial	183	CAN64497	6.38E-10	GO:0046872	7.80E+01	59.6918	41
THC_THT_c317	hypothetical chloroplast rf68	449	NP_039436	7.12E-84	GO:0009507	100	251.136	123
THC_THT_c320	retrotransposon protein	581	ABR26094	0.00736421	-	49	40.4318	71
THC_THT_c331	transposon en spm sub-class	227	CAE02323	3.53E-46	GO:0006508	98	1.58E+02	75
THC_THT_c333	calmodulin-binding protein	167	NP_191020	0.894415	-	53	32.7278	45
THC_THT_c336	polyubiquitin	198	AEI29169	9.71E-30	-	9.00E+01	108.227	63
THC_THT_c340	senescence-associated protein	246	KEH16990	0.484936	-	58	33.8834	36
THC_THT_c342	hypothetical protein PHAVU_003G142600g	304	XP_007154729	9.68E-04	-	45	4.04E+01	83
THC_THT_c343	hgwp repeat containing	366	ABA96816	1.98E-41	GO:0016020	9.40E+01	142.895	76
THC_THT_c344	hypothetical protein	151	AAV31236	1.60E-09	-	6.60E+01	55.4546	51
THC_THT_c346	retrotransposon ty3-gypsy subclass	212	ABA92236	2.38E-38	GO:0003964	9.80E+01	142.895	64
THC_THT_c348	ycf68 protein	386	KEH17697	2.31E-24	-	6.30E+01	97.0561	98
THC_THT_c366	hgwp repeat containing	403	AAX95575	3.86E-49	GO:0005739	78	1.71E+02	123
THC_THT_dn_c369	retrotransposon unclassified	386	CAD39341	8.26E-41	GO:0090502	9.40E+01	152.14	70
THC_THT_c375	cell wall-associated partial	916	EPS74533	1.88E-09	-	7.30E+01	59.3066	42
THC_THT_c377	hypothetical protein M569_00248, partial	310	EPS74531	2.99E-04	-	5.10E+01	41.9726	56
THC_THT_c378	dehydration responsive protein	235	BAJ11784	0.0841172	-	52	35.8094	51
THC_THT_c382	predicted protein	241	XP_001773392	0.993496	-	51	33.4982	39
THC_THT_c386	retrotransposon ty3-gypsy subclass	418	BAD03032	2.13E-34	GO:0003964	6.90E+01	124.02	103
THC_THT_c391	methyltransferase pmt5	205	KEH37513	1.87E-05	-	7.10E+01	46.9802	38
THC_THT_c393	hypothetical protein SELMODRAFT_97647	131	XP_002972578	0.0698609	-	65	35.8094	43
THC_THT_c397	hypothetical protein MTR_029s0004	123	XP_003636120	0.12541	-	58	35.039	39

THC_THT_c407	transposon en spm sub-class	257	ABA98787	1.45E-51	GO:0006508	9.60E+01	168.703	84
THC_THT_c411	unknown	310	ACR38454	0.943471	-	62	33.4982	37
THC_THT_c413	ORF137	225	YP_001152206	0.0891878	-	63	35.039	44
THC_THT_c416	AC084295_8hypothetical protein Os03g28210	136	AAK55475	1.68E-22	GO:0008270	9.70E+01	95.1301	45
THC_THT_c418	ribosomal protein s11	128	NP_050818	0.00296447	-	76	37.7354	34
THC_THT_c419	retrotransposon ty3-gypsy subclass	251	AAP52162	1.66E-13	GO:0003676	8.70E+01	72.0182	41
THC_THT_c427	retrotransposon ty3-gypsy subclass	296	AAP53928	1.60E-16	GO:0003964	9.20E+01	81.2629	96
THC_THT_c436	ring-h2 finger protein atl13	116	XP_003537635	0.264455	-	58	33.8834	41
THC_THT_c437	root cap protein 1-like	270	BAD68354	7.79E-11	-	8.90E+01	63.1586	38
THC_THT_c439	hypothetical protein JCGZ_04272	132	KDP38347	0.274782	-	61	31.9574	34
THC_THT_c449	metallocarboxypeptidase inhibitor	317	EPS74345	9.42E-06	-	6.20E+01	46.2098	45
THC_THT_c455	unknow protein	319	EPS74505	7.45E-09	-	5.50E+01	56.9954	103
THC_THT_c467	cell wall-associated partial	244	XP_003610225	0.00827466	-	52	39.2762	51
THC_THT_c472	senescence-associated protein	160	XP_002467304	2.95E-12	-	88	6.24E+01	35
THC_THT_c479	retrotransposon protein	212	ABR26094	7.62E-04	-	5.30E+01	40.0466	69
THC_THT_c483	hgwp repeat containing	143	AAV59434	8.54E-27	GO:0009536	1.00E+02	108.227	47
THC_THT_c484	hypothetical protein Csa_4G243400	163	KGN53992	4.43E-05	-	7.20E+01	42.743	36
THC_THT_c485	hypothetical protein PRUPE_ppa012195mg	132	XP_007212138	0.5232	-	53	32.3426	41
THC_THT_c489	unknown	191	ABK24818	0.665804	-	55	33.4982	47
THC_THT_c493	dehydration responsive protein	261	BAJ11784	2.20E-05	-	5.80E+01	40.817	39
THC_THT_c499	orf108a (mitochondrion)	187	YP_009091701	1.90E-34	GO:0005739	98	1.20E+02	59
THC_THT_c501	nadh-plastoquinone oxidoreductase subunit j	127	AGP51148	2.37E-18	GO:0005886	1.00E+02	79.337	36

THC_THT_c510	retrotransposon ty3-gypsy subclass	878	AAV32172	2.24E-137	GO:0003964	1.00E+02	273.092	133
THC_THT_c514	retrotransposon unclassified	629	CAE02097	4.67E-70	GO:0004523	7.60E+01	156.762	112
THC_THT_c515	cell wall-associated hydrolase	281	XP_003637074	1.75E-06	-	6.00E+01	51.2174	50
THC_THT_c523	hypothetical protein MTR_2g059740	197	XP_003595724	0.00901577	-	55	37.7354	36
THC_THT_c527	transposon mutator sub-class	185	ABA99682	2.36E-24	GO:0016020	9.70E+01	92.4337	40
THC_THT_c528	argininosuccinate lyase	131	XP_003055265	5.21E-11	GO:0055114	80	6.20E+01	41
THC_THT_c529	retrotransposon unclassified	131	CAE03600	9.81E-20	GO:0003676	1.00E+02	88.1965	43
THC_THT_c533	transposon unclassified	318	AAT93918	5.07E-43	-	100	1.31E+02	66
THC_THT_c542	retrotransposon unclassified	299	ABA97386	5.90E-32	-	9.50E+01	123.635	62
THC_THT_c545	ribosomal protein s10	136	XP_003588337	4.55E-07	-	6.30E+01	51.2174	44
THC_THT_c546	salicylic acid-binding protein 2-like	164	BAD46202	3.17E-05	-	6.20E+01	45.0542	59
THC_THT_c551	unknown	140	AFK45453	1.62E-15	-	8.90E+01	70.4774	37
THC_THT_c555	retrotransposon ty3-gypsy subclass	173	CAE02453	2.68E-24	GO:0004523	1.00E+02	98.9821	48
THC_THT_c559	predicted protein	280	XP_002507974	9.45E-05	-	6.60E+01	42.743	42
THC_THT_c565	unknow protein	218	EYU24190	1.07E-07	-	6.20E+01	50.8322	51
THC_THT_c566	minor allergen alt a 7-like	183	XP_002960336	7.12E-12	-	67	6.28E+01	59
THC_THT_c571	retrotransposon ty3-gypsy subclass	435	AAV43985	1.31E-63	GO:0003964	98	1.71E+02	83
THC_THT_c574	hypothetical protein SORBIDRAFT_0531s002010	241	XP_002489002	3.04E-09	-	72	5.47E+01	43
THC_THT_c582	hypothetical protein	193	AAU10722	0.0988028	-	51	36.1946	45
THC_THT_c600	transposon en spm sub-class	203	ABA95153	1.55E-41	GO:0003676	1.00E+02	152.14	67
THC_THT_c602	retrotransposon unclassified	321	ABF93933	3.35E-31	GO:0004523	9.60E+01	123.635	62
THC_THT_c610	lac23_orysj ame: full=laccase	109	EEE52618	5.40E-15	GO:0005507	9.70E+01	73.1738	36

THC_THT_c611	retrotransposon ty3-gypsy subclass	517	ABA94806	7.08E-31	GO:0004523	7.80E+01	121.709	96
THC_THT_c619	senescence-associated protein	201	XP_002488956	1.18E-19	-	80	81.6481	60
THC_THT_c620	unknow protein	362	KJB76688	1.44E-15	-	6.00E+01	74.7146	81
THC_THT_c622	ferrochelatase-chloroplastic-like	124	EEC67124	1.29E-12	GO:0004325	8.20E+01	67.0106	41
THC_THT_c625	hypothetical protein MTR_5g051150	176	XP_003614396	4.01E-04	-	6.50E+01	43.1282	41
THC_THT_c630	retrotransposon ty3-gypsy subclass	196	AAQ56535	1.64E-04	GO:0043231	6.80E+01	43.1282	35
THC_THT_c632	atp synthase subunit alpha	258	YP_001152204	6.37E-08	GO:0009536	72	4.66E+01	36
THC_THT_c634	hypothetical protein JCGZ_12157	185	KDP32865	0.0705928	-	54	36.5798	51
THC_THT_c639	cell wall-associated hydrolase	314	KEH29749	1.76E-14	-	6.40E+01	72.7886	68
THC_THT_c640	unnamed protein product	200	CDP00908	0.609119	-	49	33.4982	51
THC_THT_c642	hypothetical protein LOC_Os11g19890	113	AAX94936	6.77E-10	-	83	5.70E+01	36
THC_THT_c646	nb-arc domain containing protein	176	AAK93796	2.15E-12	GO:0006915	9.50E+01	59.3066	48
THC_THT_c648	hypothetical protein MTR_0021s0160	162	KEH17348	9.72E-04	-	6.50E+01	41.5874	38
THC_THT_c651	hypothetical protein	117	NP_001182860	1.81E-09	-	81	55.0694	3.80E+01
THC_THT_c656	salicylic acid-binding protein 2-like	141	NP_001043328	6.16E-18	GO:0005739	95	79.7221	40
THC_THT_c664	dna repair protein rhp54	113	EXB29273	0.934282	-	60	32.3426	35
THC_THT_c665	hypothetical protein VITISV_018435	169	CAN80172	0.00173233	-	55	41.2022	54
THC_THT_c668	predicted protein	236	XP_001785944	0.0961447	-	40	34.6538	67
THC_THT_c672	atp synthase subunit alpha	154	XP_003588326	6.21E-04	-	5.60E+01	42.3578	48
THC_THT_c677	rrna intron-encoded homing endonuclease	206	KEH17345	5.40E-09	-	6.80E+01	55.4546	47
THC_THT_c682	hypothetical protein PHAVU_001G037800g	211	XP_007161040	6.59E-12	-	57	6.16E+01	69
THC_THT_c692	hypothetical protein	180	AAU43943	2.31E-18	-	8.80E+01	80.8777	45

THC_THT_c693	dna repair rada-like protein	123	XP_002528203	8.16E-06	-	70	4.39E+01	37
THC_THT_c702	hypothetical protein	115	AAO37962	2.05E-10	GO:0006468	84	5.82E+01	38
THC_THT_c709	retrotransposon unclassified	332	AAP06922	1.31E-34	GO:0004523	89	1.30E+02	75
THC_THT_c712	retrotransposon unclassified	312	CAE03549	2.01E-31	GO:0004523	9.10E+01	117.472	96
THC_THT_c719	cell wall-associated partial	419	EPS74717	2.21E-06	-	7.10E+01	48.9062	42
THC_THT_c728	retrotransposon ty3-gypsy subclass	114	ABB47502	1.13E-15	GO:0004185	9.70E+01	75.8702	37
THC_THT_c735	senescence-associated protein	117	KEH16994	0.373398	-	40	33.4982	71
THC_THT_c737	protease do-like 14	117	CDM81305	0.0540208	-	60	35.8094	38
THC_THT_c738	hypothetical protein MTR_0002s0270	132	KEH17697	0.00891251	-	62	36.1946	35
THC_THT_c739	retrotransposon ty3-gypsy subclass	377	AAM93446	3.57E-21	GO:0090502	8.00E+01	93.2041	51
THC_THT_c743	hypothetical protein OsI_34071	205	EEC67196	6.11E-04	-	4.60E+01	41.5874	77
THC_THT_c748	alpha- partial	147	AFK78039	3.57E-28	GO:0005874	1.00E+02	107.457	48
THC_THT_c751	tpa_exp: transposase	211	ABA98432	1.61E-40	GO:0005739	9.80E+01	148.288	69
THC_THT_c754	cytochrome oxidase subunit i	134	ACB41367	1.14E-22	GO:0004129	9.70E+01	90.8929	44
THC_THT_c757	unknow protein	246	EYU24190	4.64E-09	-	5.90E+01	54.6842	62
THC_THT_c759	epstein-barr virus ebna-1-like protein	512	BAD67699	4.12E-64	GO:0006367	8.70E+01	202.986	121
THC_THT_c763	mitochondrial protein	132	AGC78969	1.36E-08	-	68	5.24E+01	47
THC_THT_c769	cell wall-associated partial	230	XP_003637074	0.00649585	-	65	40.0466	38
THC_THT_c774	hypothetical protein MTR_029s0001	119	XP_003636119	0.103603	-	70	32.7278	34
THC_THT_c783	cell wall-associated hydrolase	222	XP_003588355	1.53E-08	-	6.10E+01	56.9954	67
THC_THT_c785	atp synthase cf1 alpha subunit	154	AJM90101	3.97E-20	GO:0046961	8.80E+01	87.0409	51
THC_THT_c789	unknow protein	163	XP_003610227	1.31E-06	-	7.30E+01	50.0618	38
THC_THT_c791	hypothetical protein M569_00274, partial	102	EPS74485	0.171572	-	64	31.9574	34

THC_THT_c794	retrotransposon ty3-gypsy subclass	110	ABA97602	3.91E-05	-	7.30E+01	45.0542	38
THC_THT_c797	hypothetical protein SORBIDRAFT_06g023150	126	XP_002446820	0.858438	-	55	32.3426	38
THC_THT_c799	rrna intron-encoded homing endonuclease	169	AIQ78384	0.00488656	-	58	37.3502	51
THC_THT_c800	hypothetical protein CYtaCp021	248	YP_001312188	4.65E-07	GO:0009536	74	48.9062	35
THC_THT_c802	unknown	303	AFK35083	1.31E-07	-	6.40E+01	51.2174	48
THC_THT_c803	unknow protein	188	XP_003599577	3.74E-06	-	63	4.66E+01	46
THC_THT_c804	hypothetical protein MTR_5g051150	179	XP_003614396	0.31697	-	58	34.6538	34
THC_THT_c806	photosystem ii protein d1	190	ADQ42084	1.01E-32	GO:0009772	1.00E+02	115.161	55
THC_THT_c810	40s ribosomal protein s17-like	116	EAZ16056	2.91E-15	GO:0003735	1.00E+02	70.4774	36
THC_THT_c812	retrotransposon ty3-gypsy subclass	357	ABA98151	6.73E-40	GO:0003676	8.60E+01	149.443	119
THC_THT_c813	transposon unclassified	237	CAE04936	5.63E-18	GO:0003676	97	8.40E+01	34
THC_THT_c826	retrotransposon ty3-gypsy subclass	279	AAM74297	3.78E-47	GO:0003676	9.40E+01	148.673	75
THC_THT_c827	senescence-associated protein	215	XP_005644354	1.31E-17	-	71	76.2554	59
THC_THT_c830	btb poz domain-containing protein at1g30440-like	120	ABR25380	5.09E-19	-	1.00E+02	82.4185	39
THC_THT_c841	tpa: tubulin alpha-1 chain	223	DAA51109	2.00E-30	GO:0005874	1.00E+02	112.079	52
THC_THT_c844	retrotransposon ty3-gypsy subclass	142	CAH66048	8.68E-17	GO:0003964	9.20E+01	79.7221	38
THC_THT_c846	senescence-associated protein	229	KEH17023	1.61E-12	-	5.60E+01	63.929	78
THC_THT_c849	gdsl esterase lipase	177	XP_003595724	4.55E-07	-	7.80E+01	49.2914	37
THC_THT_c853	ycf68 protein	197	CDY63598	9.61E-12	-	72	61.6178	48
THC_THT_c859	polyphenol oxidase chloroplastic	201	EMS49290	1.34E-16	GO:0046872	7.50E+01	76.6406	57
THC_THT_c862	hypothetical protein MTR_5g050490	197	XP_003614355	0.00273488	-	64	40.817	34

THC_THT_c871	retrotransposon ty3-gypsy subclass	217	ABF95975	4.39E-30	GO:0003964	9.30E+01	114.775	61
THC_THT_c873	hypothetical protein SORBIDRAFT_1292s002010	149	XP_002488951	1.88E-07	-	72	4.89E+01	40
THC_THT_c881	hypothetical protein POPTR_0014s19710g	225	XP_006375694	9.44E-14	-	88	6.70E+01	35
THC_THT_c887	PREDICTED: uncharacterized protein LOC104748985 isoform X3	116	XP_010468863	1.22E-06	-	7.50E+01	49.2914	36
THC_THT_c893	hypothetical protein L484_013541	486	EXB37177	7.82E-31	GO:0006413	8.90E+01	114.39	69
THC_THT_c898	senescence-associated protein	151	XP_002489152	7.98E-11	-	85	5.97E+01	35
THC_THT_c904	hypothetical protein AMTR_s00028p00129850	228	ERN10593	2.55E-05	-	4.50E+01	44.669	84
THC_THT_c916	hgwp repeat containing	261	ABA94269	1.84E-22	GO:0003676	9.30E+01	97.8265	46
THC_THT_c920	retrotransposon ty3-gypsy subclass	324	CAE02453	9.17E-49	GO:0004523	8.20E+01	167.933	102
THC_THT_c922	senescence-associated protein	196	XP_002488950	4.25E-21	-	93	8.51E+01	45
THC_THT_c964	hypothetical protein CHLPROCp009, partial	210	AGB85039	3.58E-09	-	7.50E+01	55.0694	44
THC_THT_c966	retrotransposon ty3-gypsy subclass	531	ABA97102	2.05E-82	GO:0004523	9.70E+01	271.552	136
THC_THT_c974	retrotransposon ty3-gypsy subclass	200	ABA99219	5.36E-33	GO:0003964	9.60E+01	127.487	58
THC_THT_c975	hypothetical protein CYtaCp094	223	YP_001312259	0.069624	-	56	34.6538	44
THC_THT_c982	ORF58e	316	YP_001152205	1.63E-10	-	66	5.89E+01	51
THC_THT_c991	hypothetical protein MTR_2g059810	161	KEH38046	4.98E-04	-	6.90E+01	40.0466	36
THC_THT_c997	senescence-associated protein	245	XP_002488950	3.78E-20	-	86	8.32E+01	50
THC_THT_c1009	orf101b	181	YP_588355	7.60E-29	GO:0005739	98	1.07E+02	53

THC_THT_c1023	hypothetical protein SELMODRAFT_138833	119	XP_002994587	1.82E-15	-	86	6.97E+01	38
THC_THT_c1030	23s ribosomal rna	213	XP_007159041	3.28E-09	GO:0005488	65	5.51E+01	60
THC_THT_c1040	legumin b partial	156	AFJ04522	0.437409	-	62	33.4982	45
THC_THT_c1042	senescence-associated protein	192	KEH17348	8.30E-14	-	7.40E+01	70.8626	54
THC_THT_c1043	hypothetical protein	122	BAG55336	0.0393938	-	72	33.8834	40
THC_THT_c1082	predicted protein	120	XP_002507974	0.0622535	-	57	33.4982	35
THC_THT_c1088	hypothetical protein OsI_18211	218	EAY96311	1.13E-10	-	8.80E+01	61.6178	34
THC_THT_c1094	retrotransposon ty3-gypsy subclass	328	AAM01159	3.22E-56	GO:0003964	100	1.91E+02	90
THC_THT_c1100	gdsl esterase lipase	208	XP_003636121	4.14E-06	-	7.10E+01	45.8246	39
THC_THT_c1106	hypothetical protein M569_17308	298	EPS57509	0.140921	-	68	35.8094	35
THC_THT_c1150	retrotransposon ty3-gypsy subclass	202	BAA84457	3.01E-37	GO:0004523	1.00E+02	138.658	66
THC_THT_c1152	vacuolar atp synthase catalytic subunit-	180	XP_007223836	1.86E-08	-	74	5.24E+01	35
THC_THT_c1158	ycf68 protein	198	CDY63598	2.27E-19	GO:0009536	79	81.6481	54
THC_THT_c1162	retrotransposon ty3-gypsy subclass	302	BAC83659	2.47E-36	GO:0009536	9.20E+01	125.946	68
THC_THT_c1166	retrotransposon ty3-gypsy subclass	222	ABA97388	4.21E-41	GO:0004523	9.70E+01	147.517	71
THC_THT_c1167	hypothetical protein MTR_8g085130	164	XP_003629657	0.00120985	-	61	38.891	36
THC_THT_c1168	xs domain containing	220	CAE04226	4.25E-41	GO:0016023	9.50E+01	138.658	72
THC_THT_c1170	retrotransposon unclassified	319	ABA94804	1.45E-23	GO:0004523	9.80E+01	97.8265	101
THC_THT_c1181	hypothetical protein SORBIDRAFT_1180s002020	230	XP_002488959	4.07E-09	-	83	5.47E+01	36
THC_THT_c1183	cell wall-associated hydrolase	256	XP_003638717	6.36E-12	GO:0008152	7.90E+01	66.2402	44
THC_THT_c1194	retrotransposon unclassified	389	AAN08241	9.27E-42	GO:0004523	97	150.599	1.19E+02
THC_THT_c1198	agc kinase	211	KEH41122	0.943988	-	57	33.113	35

THC_THT_c1200	retrotransposon unclassified	356	CAE03704	9.03E-47	GO:0004523	1.00E+02	85.1149	39
THC_THT_c1214	retrotransposon ty3-gypsy subclass	148	ABA97388	8.33E-26	GO:0004523	1.00E+02	104.375	45
THC_THT_c1215	predicted protein	257	XP_001775068	0.404124	-	62	34.6538	40
THC_THT_c1216	transposon en spm sub-class	223	ABA98268	2.54E-41	GO:0005739	1.00E+02	140.969	69
THC_THT_c1217	retrotransposon ty3-gypsy subclass	193	AAS79740	3.57E-25	GO:0004523	9.50E+01	97.8265	47
THC_THT_c1218	leucine rich protein	307	ABH09321	0.0704193	-	60	36.1946	41
THC_THT_c1221	retrotransposon unclassified	150	CAE04097	2.48E-16	GO:0016023	9.30E+01	78.1814	44
THC_THT_c1222	retrotransposon unclassified	496	AAP52619	5.10E-58	GO:0004523	89	1.79E+02	128
THC_THT_c1228	senescence-associated protein	158	ABN50029	2.62E-13	-	7.80E+01	64.3142	38
THC_THT_c1236	translation elongation factor tu family protein	223	BAJ91931	1.02E-44	GO:0003746	1.00E+02	148.673	72
THC_THT_c1240	retrotransposon ty3-gypsy subclass	252	AAQ56521	1.61E-42	GO:0003964	9.70E+01	150.984	73
THC_THT_c1241	retrotransposon ty3-gypsy subclass	351	CAH66140	1.88E-19	GO:0003964	9.00E+01	50.0618	65
THC_THT_c1242	transposon en spm sub-class	267	DAA02095	1.11E-38	-	9.80E+01	141.354	65
THC_THT_c1243	transposon en spm sub-class	107	ABA98432	1.07E-18	-	1.00E+02	84.3445	34
THC_THT_c1247	retrotransposon unclassified	438	AAL93056	2.08E-28	GO:0003899	74	112.464	8.20E+01
THC_THT_c1248	zinc knuckle family protein	155	NP_001173078	3.02E-24	GO:0003676	94	1.01E+02	51
THC_THT_c1257	retrotransposon ty3-gypsy subclass	469	ABA97503	2.56E-30	GO:0003964	8.70E+01	122.479	66
THC_THT_c1259	predicted protein	125	BAJ94340	2.56E-12	-	8.60E+01	62.7734	37
THC_THT_c1266	hypothetical protein	110	BAD87500	0.130865	-	64	32.3426	34
THC_THT_c1274	senescence-associated protein	113	ACJ09634	5.63E-06	-	7.50E+01	45.0542	36
THC_THT_c1277	retrotransposon ty3-gypsy subclass	211	ABA99101	2.23E-19	GO:0003676	8.50E+01	88.1965	54
THC_THT_c1282	pr1-like protein	319	BAD22424	1.74E-31	-	9.50E+01	120.939	64

THC_THT_c1289	hypothetical protein SELMODRAFT_130337	214	XP_002989743	0.0448843	-	62	36.965	35
THC_THT_c1291	conserved hypothetical protein	114	XP_002535691	0.0664039	-	67	35.8094	37
THC_THT_c1294	retrotransposon ty3-gypsy subclass	299	CAH65890	5.93E-63	GO:0003676	9.70E+01	204.912	99
THC_THT_c1299	unknow protein	148	EPS74505	6.95E-13	GO:0009507	7.90E+01	65.4698	43
THC_THT_c1302	hypothetical protein MTR_0986s0010, partial	200	KEH15466	0.0423999	-	61	35.039	34
THC_THT_c1304	ankyrin-2-like isoform x1	101	XP_004970869	1.32E-11	GO:0006468	9.40E+01	63.1586	34
THC_THT_c1310	retrotransposon ty1-copia subclass	288	BAC84722	7.28E-46	GO:0003676	9.50E+01	150.214	80
THC_THT_c1317	retrotransposon unclassified	309	CAE02127	2.25E-64	GO:0004523	1.00E+02	207.608	99
THC_THT_c1320	retrotransposon unclassified	217	CAD40289	6.88E-27	GO:0004523	9.70E+01	110.153	72
THC_THT_c1329	60s ribosomal protein l8	154	DAA47081	2.94E-17	GO:0003735	100	7.39E+01	35
THC_THT_c1333	hypothetical protein OsI_23122	115	EEC80696	8.76E-17	GO:0005739	9.70E+01	75.8702	37
THC_THT_c1336	pr1-like protein	237	AAM01054	2.89E-04	GO:0003964	9.50E+01	43.5134	64
THC_THT_c1337	retrotransposon unclassified	192	BAD61965	7.14E-20	-	8.90E+01	83.5741	47
THC_THT_c1346	retrotransposon ty3-gypsy subclass	211	CAH68025	1.19E-39	GO:0003677	9.80E+01	146.747	70
THC_THT_c1348	retrotransposon unclassified	117	ABA94261	7.92E-04	-	6.50E+01	39.6614	35
THC_THT_c1353	retrotransposon unclassified	172	CAE04107	7.04E-10	GO:0004523	70	6.01E+01	48
THC_THT_c1357	hypothetical protein MTR_0021s0160	118	KEH17348	4.91E-06	-	5.50E+01	47.7506	52
THC_THT_c1365	retrotransposon ty3-gypsy subclass	110	ABA98852	4.26E-11	GO:0003676	8.80E+01	62.7734	34
THC_THT_c1369	retrotransposon ty1-copia subclass	305	ABA99153	1.07E-60	GO:0055114	1.00E+02	207.994	98
THC_THT_c1372	retrotransposon ty3-gypsy subclass	227	AAQ56295	1.05E-26	GO:0004523	9.70E+01	105.145	74

THC_THT_c1373	thiazole biosynthetic enzyme thi4 family	117	BAC79982	5.73E-17	GO:0005829	1.00E+02	77.0258	36
THC_THT_c1374	retrotransposon unclassified	452	AAX94870	2.03E-75	GO:0004523	89	2.42E+02	138
THC_THT_c1389	retrotransposon ty3-gypsy subclass	165	AAQ56439	6.71E-25	GO:0016023	90	9.63E+01	54
THC_THT_c1392	transposon mutator sub-class	218	CAH68172	1.39E-26	GO:0003676	9.80E+01	108.227	50
THC_THT_c1395	transposon en spm sub-class	111	AAX95947	4.04E-12	-	82	6.55E+01	40
THC_THT_c1399	transposon en spm sub-class	170	ABA98787	1.47E-26	GO:0006508	9.80E+01	102.834	55
THC_THT_c1401	rrna intron-encoded homing endonuclease	243	XP_006363639	4.60E-04	-	4.70E+01	43.1282	71
THC_THT_c1402	retrotransposon ty3-gypsy sub-class	168	AAO38011	3.79E-21	GO:0016023	87	8.97E+01	41
THC_THT_c1404	glutaredoxin-related protein	144	XP_003081198	3.19E-10	GO:0051536	80	5.93E+01	40
THC_THT_c1408	hypothetical protein	226	BAD25936	0.0292193	-	81	37.3502	48
THC_THT_c1410	xs domain containing	324	BAD10713	1.79E-39	GO:0005739	9.30E+01	135.576	72
THC_THT_c1413	splicing coactivator subunit-like	145	CAE04971	4.96E-21	GO:0004523	95	8.67E+01	48
THC_THT_c1414	unknow protein	112	YP_001312258	1.22E-04	-	71	40.817	35
THC_THT_c1424	unknown	175	AFK37255	0.0661065	-	67	33.8834	43
THC_THT_c1434	centromere-specific	215	AAT07557	1.43E-30	GO:0005739	1.00E+02	111.694	52
THC_THT_c1436	probable mediator of rna polymerase ii transcription subunit 37c	174	CAA54420	6.90E-27	GO:0005524	9.70E+01	100.908	48
THC_THT_c1439	kynurenine--oxoglutarate transaminase-like	113	XP_004982811	7.82E-04	-	7.00E+01	41.2022	34
THC_THT_c1455	transposon mutator sub-class	317	ABA98631	9.75E-51	GO:0004553	9.80E+01	177.178	87
THC_THT_c1461	ribosomal protein s11	137	YP_009033475	3.30E-25	GO:0006412	100	9.71E+01	45
THC_THT_c1463	transposon en spm sub-class	256	AAP53844	9.59E-16	GO:0008270	9.70E+01	78.5666	34
THC_THT_c1466	retrotransposon unclassified	233	AAU44223	1.44E-20	GO:0090502	9.10E+01	92.0485	49

THC_THT_c1488	fumarate hydratase mitochondrial	129	ACU23330	1.67E-07	GO:0045239	7.70E+01	49.6766	40
THC_THT_c1496	bacterial transferase hexapeptide repeat-containing protein	129	XP_002539100	5.31E-13	GO:2001289	83	65.4698	43
THC_THT_c1497	retrotransposon ty3-gypsy subclass	129	CAI64477	0.00433601	-	89	38.1206	39
THC_THT_c1500	retrotransposon ty3-gypsy subclass	236	AAV31373	2.88E-33	GO:0003964	9.60E+01	129.028	65
THC_THT_c1503	cytochrome p450-like	164	BAD61189	0.603619	-	51	33.113	43
THC_THT_c1507	retrotransposon ty3-gypsy subclass	303	ABF95972	1.62E-13	GO:0003676	9.70E+01	69.707	34
THC_THT_c1509	conserved hypothetical protein	166	XP_002538135	1.50E-07	-	81	5.04E+01	38
THC_THT_c1512	hypothetical protein OsI_28514	117	EEC83227	0.00280853	-	60	36.965	40
THC_THT_c1513	70 kda peptidyl-prolyl isomerase	119	XP_003626838	0.905921	-	50	32.3426	34
THC_THT_c1515	atp synthase cf1 alpha subunit	225	AFK82067	5.95E-25	GO:0046961	100	97.0561	68
THC_THT_c1519	retrotransposon unclassified	186	ABB47238	9.98E-20	GO:0004523	1.00E+02	82.8037	38
THC_THT_c1522	retrotransposon protein	178	BAH80065	4.99E-17	GO:0003676	1.00E+02	80.4925	37
THC_THT_c1525	BnaCnng12640D	234	CDY45505	7.66E-10	-	7.00E+01	60.077	54
THC_THT_c1531	retrotransposon unclassified	171	BAD27688	1.21E-17	GO:0005488	9.00E+01	77.411	44
THC_THT_c1538	reverse transcriptase	200	AAQ56457	1.23E-24	GO:0003964	9.20E+01	103.219	57
THC_THT_c1551	cell wall	367	BAD29085	4.68E-04	-	8.20E+01	43.8986	46
THC_THT_c1554	retrotransposon ty3-gypsy subclass	133	CAD40517	5.14E-16	GO:0004523	94	76.2554	3.70E+01
THC_THT_c1555	retrotransposon ty3-gypsy subclass	347	AAM01157	3.45E-23	GO:0006259	83	9.78E+01	65
THC_THT_c1557	oxysterol-binding protein 1c-like isoform x2	188	ABR25965	5.42E-04	-	5.70E+01	40.0466	49
THC_THT_c1558	retrotransposon unclassified	363	BAD54689	3.57E-22	GO:0005739	85	9.05E+01	60
THC_THT_c1566	transposon en spm sub-class	184	CAE02903	1.65E-14	GO:0006468	1.00E+02	64.6994	38

THC_THT_c1568	conserved hypothetical protein	143	XP_002537027	4.22E-14	GO:0045493	100	7.09E+01	34
THC_THT_c1575	hypothetical protein CHLPROCp009, partial	211	AGB85039	0.00445188	-	56	37.7354	44
THC_THT_c1576	hgwp repeat containing	142	ABF93663	2.07E-26	GO:0005739	1.00E+02	102.064	47
THC_THT_c1584	rrna intron-encoded homing endonuclease	215	XP_003614389	1.49E-06	-	5.90E+01	50.447	47
THC_THT_c1590	protein	153	EEC70731	6.80E-04	GO:0005739	6.90E+01	39.2762	43
THC_THT_c1593	plant disease resistance poly	129	BAD29140	5.45E-11	GO:0005739	84	5.93E+01	38
THC_THT_c1594	hypothetical protein SORBIDRAFT_09g024420	153	XP_002441320	0.474236	-	43	33.4982	41
THC_THT_c1596	retrotransposon unclassified	179	AAT47449	5.07E-22	GO:0003676	9.40E+01	95.5153	53
THC_THT_c1597	senescence-associated protein	122	XP_011013113	0.00276401	-	62	40.0466	35
THC_THT_c1603	BnaAnng22160D, partial	134	CDY66372	0.0268518	-	66	36.965	36
THC_THT_c1607	retrotransposon ty3-gypsy subclass	278	ABA98151	9.72E-22	GO:0003676	9.60E+01	95.9005	59
THC_THT_c1608	nadh dehydrogenase subunit	144	XP_003588352	8.21E-05	-	6.40E+01	45.0542	48
THC_THT_c1610	rrna intron-encoded homing endonuclease	118	XP_003614387	0.0250402	-	55	37.3502	45
THC_THT_c1613	hypothetical protein M569_00407, partial	187	EPS74345	6.72E-05	-	5.70E+01	42.743	54
THC_THT_c1624	OSJNBa0020I02.2	110	CAE02095	0.00312767	-	66	38.891	36
THC_THT_c1632	cell wall-associated partial	226	XP_003637074	1.28E-04	-	6.20E+01	45.0542	48
THC_THT_c1633	retrotransposon unclassified	227	CAE02761	4.17E-41	GO:0004523	98	1.51E+02	75
THC_THT_c1638	senescence-associated protein	262	EMS58925	8.19E-15	-	9.40E+01	69.707	35
THC_THT_c1642	unknown protein	105	BAD17691	6.50E-13	GO:0043631	1.00E+02	65.4698	34
THC_THT_c1648	xs domain containing	137	AAT44151	1.05E-08	-	8.80E+01	53.1434	34
THC_THT_c1656	zinc finger	105	EAY73499	2.07E-12	GO:0008270	9.10E+01	64.6994	34
THC_THT_c1658	ribosomal protein s10	179	XP_003627937	2.48E-06	-	71	4.85E+01	42

THC_THT_c1661	cell wall-associated hydrolase	239	ERN19185	0.0740972	-	60	35.8094	41
THC_THT_c1670	hgwp repeat containing	193	BAD61609	7.66E-17	GO:0005739	7.50E+01	79.7221	62
THC_THT_c1672	fructose-bisphosphate aldolase	192	BAD82730	3.36E-35	GO:0005737	98	125.561	6.30E+01
THC_THT_c1674	lecithin cholesterol acyltransferase-like protein	274	BAD09499	7.45E-36	GO:0043565	9.40E+01	129.413	67
THC_THT_c1698	retrotransposon ty3-gypsy subclass	417	ABF95974	1.81E-64	GO:0003964	9.80E+01	150.599	75
THC_THT_c1706	retrotransposon ty3-gypsy subclass	265	BAA75236	1.60E-36	GO:0003676	8.50E+01	131.724	78
THC_THT_c1716	hypothetical protein MTR_029s0001	120	XP_003636119	0.00405889	-	65	36.965	35
THC_THT_c1744	retrotransposon ty3-gypsy subclass	345	CAE05319	5.87E-72	GO:0003964	9.80E+01	238.039	115
THC_THT_c1746	hypothetical protein	196	BAI94527	1.85E-16	GO:0005739	100	7.39E+01	38
THC_THT_c1749	hypothetical protein B456_009G228100	192	KJB58829	0.410839	-	56	34.2686	39
THC_THT_c1754	psii 47 kda partial	163	AIO04664	2.22E-22	GO:0009523	83	89.3521	54
THC_THT_c1759	retrotransposon ty3-gypsy subclass	262	CAH66117	4.12E-41	GO:0003964	9.80E+01	151.369	72
THC_THT_c1760	unclassified retrotransposon protein	154	BAH79998	4.71E-16	GO:0005739	89	7.12E+01	38
THC_THT_c1764	transposon en spm sub-class	315	CAD40640	4.47E-46	GO:0005507	9.50E+01	122.094	65
THC_THT_c1771	-	112	KIY99539	7.10E-10	GO:0008270	8.30E+01	58.5362	36
THC_THT_c1773	nb-arc domain containing expressed	202	ABA92251	4.94E-20	GO:0043531	1.00E+02	87.8113	40
THC_THT_c1789	retrotransposon ty3-gypsy subclass	322	CAE02295	2.78E-51	GO:0003676	98	1.70E+02	83
THC_THT_c1796	protein	165	XP_005842766	1.25E-10	-	83	57.7658	36
THC_THT_c1800	c4-dicarboxylate transport transcriptional regulatory protein	109	XP_002537860	1.42E-05	GO:1901363	66	4.54E+01	36
THC_THT_c1801	retrotransposon unclassified	217	CAD40289	9.43E-26	GO:0004523	8.50E+01	106.686	62

THC_THT_c1803	unclassified retrotransposon protein	116	BAD22439	9.29E-21	-	1.00E+02	83.9593	38
THC_THT_c1804	hypothetical protein SORBIDRAFT_10g007210	189	XP_002436698	0.295771	-	45	34.2686	42
THC_THT_c1815	hypothetical protein SORBIDRAFT_0016s002240	104	XP_002489152	3.90E-05	-	69	4.39E+01	36
THC_THT_c1819	hypothetical protein M569_00037, partial	146	EPS74712	5.72E-04	-	5.20E+01	39.2762	51
THC_THT_c1820	ndp-l-rhamnose synthase	134	XP_011082935	1.58E-10	GO:0005829	7.90E+01	60.4622	43
THC_THT_c1822	hypothetical protein SORBIDRAFT_0070s002020	203	XP_002489102	1.15E-11	-	65	6.16E+01	52
THC_THT_c1823	abscisic stress ripening protein	179	NP_001065841	0.0201186	-	100	36.1946	53
THC_THT_c1829	transposon en spm sub-class	212	CAE02322	1.21E-34	GO:0005739	100	1.29E+02	57
THC_THT_c1830	hypothetical protein M569_04734, partial	251	EPS70027	1.55E-04	-	6.00E+01	41.9726	38
THC_THT_c1834	light harvesting complex from photosystem i type b	194	AFK40204	1.97E-26	GO:0046872	1.00E+02	102.449	48
THC_THT_c1837	retrotransposon ty3-gypsy subclass	341	AAM74297	4.23E-66	GO:0003676	1.00E+02	221.476	105
THC_THT_c1842	PREDICTED: uncharacterized protein LOC104247622	145	XP_009801990	0.961455	-	56	32.7278	39
THC_THT_c1843	retrotransposon unclassified	140	ABB47432	6.27E-27	GO:0003676	1.00E+02	102.449	46
THC_THT_c1847	ein3-binding f-box protein 1-like	102	EAZ01599	2.27E-14	-	100	7.16E+01	34
THC_THT_c1850	retrotransposon ty3-gypsy subclass	486	CAH66904	1.44E-61	GO:0003964	9.60E+01	169.859	84
THC_THT_c1860	hypothetical protein NitaMp073	221	YP_173415	0.00464956	-	56	38.1206	48
THC_THT_c1875	retrotransposon unclassified	229	NP_001176697	7.82E-27	GO:0050660	97	1.06E+02	74

THC_THT_c1877	probable arabinose 5-phosphate isomerase	137	XP_006857298	0.147857	-	58	34.6538	41
THC_THT_c1879	uncharacterized protein LOC105120237	110	XP_011016720	0.757261	-	57	32.3426	35
THC_THT_c1884	retrotransposon ty3-gypsy subclass	128	AAM93705	2.38E-19	GO:0004523	1.00E+02	84.7297	38
THC_THT_c1889	retrotransposon ty3-gypsy subclass	126	CAE05319	2.22E-19	GO:0003676	1.00E+02	86.6557	39
THC_THT_c1891	3-oxoacyl-	105	KDO39747	6.65E-07	GO:0016747	7.10E+01	47.3654	35
THC_THT_c1893	uncharacterized protein LOC104434851	125	XP_010046010	0.00345596	-	66	38.891	39
THC_THT_c1895	hypothetical protein M569_00248, partial	146	EPS74531	2.55E-09	-	7.40E+01	54.6842	43
THC_THT_c1903	omega-6 fatty acid endoplasmic reticulum isozyme 2	105	XP_006647793	2.69E-14	GO:0005789	1.00E+02	70.4774	34
THC_THT_c1904	senescence-associated protein	108	BAB33421	5.99E-04	-	3.50E+01	41.2022	87
THC_THT_c1905	transposon unclassified	206	BAD62016	0.00130953	-	55	38.891	34
THC_THT_c1906	retrotransposon ty3-gypsy subclass	298	ABA97532	1.22E-07	-	9.20E+01	54.6842	39
THC_THT_c1912	retrotransposon ty3-gypsy subclass	186	AAV24824	5.58E-31	GO:0003964	9.60E+01	121.324	56
THC_THT_c1924	hypothetical protein OsI_20145	116	EEC79310	1.83E-14	GO:0009536	9.20E+01	72.0182	39
THC_THT_c1929	hypothetical protein	152	NP_001159114	5.05E-05	-	63	42.743	38
THC_THT_c1930	hypothetical protein MTR_0021s0160	175	KEH17348	5.56E-14	-	8.00E+01	71.2478	46
THC_THT_c1945	retrotransposon unclassified	252	BAD69387	1.92E-23	GO:0008270	9.30E+01	93.2041	60
THC_THT_c1947	retrotransposon ty3-gypsy subclass	262	BAA75236	8.83E-50	GO:0003964	9.50E+01	166.007	83
THC_THT_c1949	hypothetical protein MTR_029s0005	229	XP_003636121	6.13E-05	-	6.00E+01	42.743	45
THC_THT_c1950	beta- -glucanase	121	NP_001059883	7.52E-20	GO:0004553	100	87.0409	40
THC_THT_c1960	hypothetical protein VOLCADRAFT_85069	156	XP_002959854	4.14E-06	-	63	4.54E+01	47
THC_THT_c1963	twitching mobility	178	XP_002534939	0.00578544	-	57	39.2762	57

THC_THT_c1972	hypothetical protein	127	BAD33792	1.96E-04	-	7.00E+01	40.817	37
THC_THT_c1980	BnaC08g46150D	175	CDY55729	1.47E-05	-	5.90E+01	46.9802	49
THC_THT_c1983	senescence associated protein	150	BAM33974	0.114415	-	57	33.113	38
THC_THT_c1988	retrotransposon ty3-gypsy subclass	220	CAE05894	1.00E-46	GO:0009536	1.00E+02	153.295	72
THC_THT_c1997	retrotransposon ty3-gypsy subclass	139	AAX95754	3.16E-25	GO:0004523	1.00E+02	100.138	45
THC_THT_dn_c2007	hypothetical protein B456_011G084600	157	KJB70635	6.09E-10	-	8.20E+01	56.9954	40
THC_THT_c2008	cell wall	261	BAD17359	3.83E-13	GO:0005739	86	6.86E+01	61
THC_THT_c2010	retrotransposon ty3-gypsy subclass	142	BAH79970	5.74E-26	GO:0003964	1.00E+02	101.679	47
THC_THT_c2012	hypothetical protein JCGZ_13348	149	KDP32423	0.210758	-	57	34.6538	35
THC_THT_c2013	hypothetical protein MTR_5g051110	159	XP_003614392	6.66E-07	-	6.50E+01	50.447	41
THC_THT_c2017	multicopper oxidase family expressed	124	NP_001067650	1.09E-08	GO:0046872	73	5.31E+01	41
THC_THT_c2020	hypothetical protein	177	BAC56826	4.74E-05	-	7.70E+01	44.669	35
THC_THT_c2022	transposon unclassified	160	AAL69430	0.00108106	-	64	39.6614	39
THC_THT_c2039	retrotransposon ty3-gypsy subclass	215	AAL69438	5.16E-19	GO:0009536	9.50E+01	83.9593	65
THC_THT_c2046	probable receptor-like protein kinase at1g11050-like	117	NP_001055591	7.92E-17	GO:0006468	100	78.9518	39
THC_THT_c2059	hypothetical protein MIMGU_mgv1a022450mg	119	EYU33320	0.635777	-	51	32.3426	39
THC_THT_c2077	retrotransposon ty3-gypsy subclass	323	CAH66198	4.83E-49	GO:0003964	9.40E+01	156.762	84
THC_THT_c2079	zinc-binding ribosomal family protein isoform partial	103	CDO98037	1.47E-14	GO:0005840	9.40E+01	68.1662	34
THC_THT_c2080	hypothetical protein MTR_5g051110	141	XP_003614392	3.33E-06	-	7.40E+01	48.521	35
THC_THT_c2085	retrotransposon ty3-gypsy subclass	149	AAP54660	9.82E-08	GO:0016151	7.20E+01	53.5286	37

THC_THT_c2103	retrotransposon ty3-gypsy subclass	220	ABB47116	3.00E-29	GO:0004523	8.20E+01	108.227	68
THC_THT_c2107	retrotransposon ty3-gypsy subclass	252	ABB47022	3.28E-51	GO:0003676	9.80E+01	172.94	84
THC_THT_c2128	predicted protein	112	XP_001786687	0.0831932	-	60	35.039	43
THC_THT_c2142	transposon en spm sub-class	118	CAH68038	9.72E-16	GO:0003676	9.40E+01	73.1738	37
THC_THT_c2148	retrotransposon ty3-gypsy sub-class	178	AAX95200	0.0890168	-	58	36.1946	46
THC_THT_c2149	retrotransposon ty1-copia subclass	126	AAP53968	8.71E-20	GO:0003676	9.70E+01	87.8113	41
THC_THT_c2150	retrotransposon unclassified	297	ABF96139	8.49E-53	GO:0003676	9.50E+01	174.096	85
THC_THT_c2152	ribosomal protein l16	157	ADD63063	4.68E-29	GO:0003735	1.00E+02	106.686	52
THC_THT_c2161	retrotransposon ty3-gypsy subclass	186	ABB47022	1.31E-24	GO:0003676	9.70E+01	99.7525	49
THC_THT_c2171	transposon mutator sub-class	173	CAD40694	2.75E-29	GO:0003676	9.60E+01	115.546	57
THC_THT_c2173	epstein-barr virus ebna-1-like protein	155	AAT01397	7.45E-15	-	9.70E+01	70.8626	36
THC_THT_c2189	hypothetical protein	113	BAD61287	9.09E-10	-	8.50E+01	55.8398	34
THC_THT_c2196	retrotransposon ty3-gypsy subclass	533	ABF95990	2.08E-83	GO:0003964	9.80E+01	253.832	140
THC_THT_c2206	transposon mutator sub-class	138	CAE75999	1.28E-24	GO:0003676	1.00E+02	101.679	45
THC_THT_c2211	pentatricopeptide repeat-containing protein chloroplastic-like	158	CAH66009	4.04E-19	GO:0031425	89	8.63E+01	49
THC_THT_c2219	transposon mutator sub-class	106	CAD40991	5.84E-18	GO:0016020	1.00E+02	82.0333	34
THC_THT_c2221	rrna intron-encoded homing endonuclease	193	XP_002488950	2.94E-14	-	85	6.74E+01	42
THC_THT_c2240	retrotransposon ty3-gypsy subclass	160	AAX95201	1.67E-19	GO:0003676	100	8.20E+01	39
THC_THT_c2242	hypothetical protein	220	BAD36100	2.43E-24	GO:0009536	9.20E+01	93.9745	50
THC_THT_c2243	retrotransposon ty3-gypsy subclass	324	ABB46919	2.43E-25	GO:0004523	9.70E+01	107.457	47
THC_THT_c2244	receptor-like protein kinase hsl1-like	124	EEE64410	1.08E-21	GO:0016023	1.00E+02	93.2041	41

THC_THT_c2252	BnaC09g29270D	182	CDY19671	4.50E-04	-	6.70E+01	41.9726	37
THC_THT_c2254	conserved hypothetical protein	114	XP_002537720	8.60E-06	-	75	4.66E+01	36
THC_THT_c2260	exopolysaccharide production protein	126	EEC83773	9.86E-12	GO:0035434	7.80E+01	64.6994	42
THC_THT_c2302	hypothetical protein	123	BAD17690	5.07E-10	-	8.00E+01	56.6102	35
THC_THT_c2317	transposon en spm sub-class	150	AAX94903	4.24E-26	GO:0005507	95	105.531	4.90E+01
THC_THT_c2318	BnaA09g04420D	132	CDY19789	1.04E-06	-	7.20E+01	49.2914	37
THC_THT_c2330	retrotransposon ty3-gypsy subclass	198	CAH66048	2.08E-20	GO:0003964	8.80E+01	90.8929	52
THC_THT_c2338	hypothetical protein OsJ_14281	124	EAZ30228	3.89E-04	GO:0005739	7.50E+01	39.6614	40
THC_THT_c2341	hypothetical protein SORBIDRAFT_0351s002020	237	XP_002489033	5.32E-25	-	100	9.55E+01	45
THC_THT_c2347	retrotransposon protein	166	BAH80021	4.88E-11	-	9.00E+01	60.8474	50
THC_THT_c2349	orf112a gene product	166	NP_064043	0.01111	-	57	36.1946	45
THC_THT_c2360	PREDICTED: uncharacterized protein LOC105037379, partial	105	XP_010911354	0.0720456	-	67	35.039	34
THC_THT_c2368	glycine-rich rna binding protein	223	AAT85299	1.26E-42	GO:0016301	100	143.665	68
THC_THT_c2375	alcohol dehydrogenase class iii	131	XP_001771825	5.95E-13	GO:0005737	87	6.70E+01	40
THC_THT_c2378	transposon unclassified	427	CAE02129	1.33E-55	GO:0004523	9.70E+01	139.043	68
THC_THT_c2385	probable indole-3-pyruvate monooxygenase yucca10	106	XP_002981097	8.59E-16	-	88	7.55E+01	35
THC_THT_c2386	abc transporter b family member 25-like	164	XP_001786505	7.81E-06	GO:0003824	69	4.81E+01	43
THC_THT_c2387	ribosomal protein s10	229	XP_003627937	1.20E-09	-	79	5.78E+01	43
THC_THT_c2389	atp synthase subunit beta	199	XP_002488946	2.29E-21	-	88	8.63E+01	50

THC_THT_c2397	diguanylate cyclase cyclic diguanylate partial	127	KEH15131	0.00129462	-	60	40.4318	40
THC_THT_c2406	retrotransposon unclassified	434	ABA99331	1.63E-29	GO:0009536	7.50E+01	120.939	87
THC_THT_dn_c2424	protein	195	DAA63564	2.08E-15	GO:0003735	9.20E+01	73.1738	38
THC_THT_c2427	peptidyl-prolyl cis-trans isomerase	119	AAA57046	5.66E-16	GO:0006457	1.00E+02	73.1738	34
THC_THT_c2431	senescence-associated protein	170	XP_001419402	1.54E-12	-	85	63.1586	34
THC_THT_c2437	thromboxane-a synthase	116	BAD17630	1.49E-19	GO:0010363	100	8.55E+01	38
THC_THT_c2451	unknow protein	191	XP_003599577	3.26E-05	-	72	4.39E+01	37
THC_THT_c2452	60s acidic ribosomal protein p0	182	NP_001176336	2.11E-28	GO:0005840	100	1.06E+02	51
THC_THT_c2457	protein phosphatase 2c 5	118	EEC70873	5.94E-15	GO:0080005	1.00E+02	72.4034	35
THC_THT_c2465	hypothetical protein	161	AAT01409	2.93E-19	-	8.90E+01	84.3445	48
THC_THT_c2479	atp synthase subunit beta	245	KFK23449	7.74E-16	-	7.80E+01	72.0182	50
THC_THT_c2487	hypothetical protein	147	BAB43994	1.88E-05	-	7.50E+01	45.8246	37
THC_THT_c2490	transposon mutator sub-class	184	AAX94899	4.69E-34	GO:0008270	9.80E+01	129.413	60
THC_THT_c2496	ribosomal protein s2	176	YP_514652	4.60E-19	GO:0046961	100	85.5001	57
THC_THT_c2497	unknow protein	137	EPS74505	0.0143815	-	66	37.3502	36
THC_THT_c2513	BnaC05g30530D	162	CDY37377	0.757932	-	50	33.113	54
THC_THT_c2517	hypothetical protein AMTR_s00191p00039450	153	ERN12143	0.00346809	-	57	38.1206	45
THC_THT_c2520	retrotransposon ty3-gypsy subclass	324	CAE02184	6.37E-70	GO:0003964	9.80E+01	218.009	108
THC_THT_c2525	retrotransposon ty3-gypsy subclass	137	BAC84692	2.40E-11	GO:0005739	89	5.93E+01	37
THC_THT_c2535	senescence-associated protein	212	XP_002489152	1.34E-14	-	97	7.12E+01	35
THC_THT_c2566	hypothetical protein	117	AAT69625	1.23E-12	GO:0005739	9.10E+01	64.6994	35

THC_THT_c2567	probably inactive leucine-rich repeat receptor-like protein kinase at3g28040-like	121	EAY77289	1.79E-19	GO:0016021	1.00E+02	87.0409	40
THC_THT_c2572	transposon mutator sub-class	138	NP_001063934	9.33E-21	GO:0051287	100	9.13E+01	41
THC_THT_c2581	retrotransposon ty3-gypsy subclass	367	CAH66287	2.33E-66	GO:0003964	9.90E+01	211.46	101
THC_THT_c2584	retrotransposon unclassified	192	BAD11623	1.03E-16	-	94	7.63E+01	37
THC_THT_c2586	cell wall-associated hydrolase	200	CDX71648	1.55E-09	-	7.70E+01	56.9954	40
THC_THT_c2594	atp synthase subunit alpha	172	XP_003595724	0.0223778	-	57	36.5798	35
THC_THT_c2602	OSJNBa0057M08.23	101	CAI44648	1.36E-08	GO:0005739	8.20E+01	53.9138	34
THC_THT_c2623	retrotransposon ty3-gypsy subclass	315	CAH66107	1.02E-58	GO:0003964	9.60E+01	200.675	99
THC_THT_c2644	omt1_orysj ame: full=flavone 3 -o-methyltransferase 1 short= 1 ame: full=quercetin 3 -o-methyltransferase 1 flags: precursor	106	EAZ05653	8.50E-15	GO:0043247	1.00E+02	71.633	34
THC_THT_c2670	transposon en spm sub-class	151	AAV32183	4.17E-07	-	6.80E+01	51.6026	47
THC_THT_c2695	hypothetical protein MIMGU_mgv1a018198mg	142	EYU20954	3.98E-05	-	6.70E+01	42.743	34
THC_THT_c2725	retrotransposon ty3-gypsy subclass	172	CAH67938	3.93E-18	GO:0090502	9.70E+01	83.9593	41
THC_THT_c2731	retrotransposon unclassified	106	CAD40050	1.19E-16	GO:0090502	100	7.82E+01	34
THC_THT_c2736	cation h(+) antiporter 15-like	122	EAY98474	1.37E-08	GO:1902600	1.00E+02	55.4546	40
THC_THT_c2741	transposon unclassified	138	ABF99578	9.10E-17	GO:0009536	9.70E+01	75.0998	38
THC_THT_c2748	carotenoid partial	151	AFN53970	0.966529	-	57	30.4166	40
THC_THT_c2756	hypothetical protein M569_00248, partial	200	EPS74531	0.00367674	-	55	38.1206	40

THC_THT_c2757	hydroxyproline-rich glyco	213	CAE03718	7.73E-18	GO:0006508	1.00E+02	83.5741	38
THC_THT_c2762	hypothetical protein OsJ_07887	110	EAZ24144	2.17E-17	GO:0005739	1.00E+02	78.5666	36
THC_THT_c2771	cobalamin-dependent methionine synthase	106	XP_003078364	5.04E-09	GO:0042558	8.80E+01	56.6102	35
THC_THT_c2774	cbs domain-containing protein mitochondrial	147	XP_011083900	0.185826	-	66	34.2686	36
THC_THT_c2775	transposon en spm sub-class	153	ABF96781	1.56E-27	GO:0006457	9.80E+01	110.538	50
THC_THT_c2778	rnase l inhibitor protein homolog	134	XP_001786560	0.00384372	-	73	39.6614	38
THC_THT_c2781	hypothetical protein SORBIDRAFT_05g016477	136	XP_002450733	5.04E-07	-	68	4.85E+01	45
THC_THT_c2782	upf0481 protein at3g02645-like	102	XP_004977990	7.50E-05	-	6.40E+01	43.5134	34
THC_THT_c2788	hypothetical protein OsI_08103	104	EAY86719	3.95E-16	-	1.00E+02	73.9442	34
THC_THT_c2791	retrotransposon ty3-gypsy subclass	370	CAH65837	5.48E-41	GO:0004523	9.70E+01	76.2554	38
THC_THT_c2795	mitogen-activated protein kinase kinase kinase yoda-like	251	KCW49550	0.167339	-	47	35.8094	74
THC_THT_c2800	hypothetical protein L484_000616	194	EXC50694	0.0160724	-	54	35.8094	48
THC_THT_c2801	cell wall-associated hydrolase	413	ABH09321	1.20E-09	-	6.30E+01	52.373	46
THC_THT_c2813	rrna intron-encoded homing endonuclease	117	AAK13589	5.59E-05	-	6.80E+01	41.9726	35
THC_THT_c2826	ac093181_6 polyprotein	174	AAT73644	1.42E-19	GO:0016023	8.70E+01	87.4261	48
THC_THT_c2834	retrotransposon unclassified	121	AAX95489	4.93E-17	-	100	7.97E+01	39
THC_THT_c2846	heme-regulated cyclic amp	113	XP_002535367	0.0130148	-	74	37.7354	35
THC_THT_c2849	hypothetical protein OsI_32006	105	EAZ09720	8.22E-13	-	8.80E+01	64.3142	35
THC_THT_c2852	transposon en spm sub-class	215	AAM09815	7.13E-40	GO:0008234	1.00E+02	142.51	68

THC_THT_c2854	protein	177	ABR16542	9.54E-08	-	7.20E+01	50.0618	37
THC_THT_c2869	retrotransposon unclassified	123	ABA95825	7.07E-11	GO:0004252	8.60E+01	62.003	37
THC_THT_c2873	sucrose synthase 2 isoform x1	106	ACF80843	2.98E-17	GO:0045893	9.40E+01	76.6406	35
THC_THT_c2876	transposon mutator sub-class	142	BAD33754	3.46E-27	GO:0051287	97	1.07E+02	47
THC_THT_c2884	retrotransposon ty3-gypsy subclass	114	CAJ86273	7.86E-18	GO:0004523	1.00E+02	82.4185	37
THC_THT_c2886	nadh dehydrogenase subunit 5	126	ABA01222	1.59E-19	GO:0016021	9.70E+01	80.1073	41
THC_THT_c2887	transposon en spm sub-class	120	ABA98583	5.10E-07	-	6.80E+01	49.6766	41
THC_THT_c2906	transposon en spm sub-class	225	CAH65845	8.85E-33	GO:0005739	9.70E+01	125.946	74
THC_THT_c2909	hypothetical protein VITISV_001840	141	CAN65763	0.00208019	-	60	40.0466	41
THC_THT_c2918	atp synthase alpha partial	151	EEC76877	1.60E-22	GO:0046961	9.60E+01	96.2857	50
THC_THT_c2928	3-isopropylmalate dehydratase small subunit 3	146	XP_003078193	2.75E-13	GO:0003861	6.90E+01	69.3218	56
THC_THT_c2939	retrotransposon ty3-gypsy subclass	266	CAH66134	1.37E-35	GO:0003676	9.60E+01	124.02	61
THC_THT_c2942	3-ketoacyl- synthase 6-like	103	ACN25352	5.14E-15	GO:0016020	1.00E+02	70.8626	34
THC_THT_c2958	embryogenesis transmembrane	150	CAJ86113	1.83E-14	GO:0016021	8.40E+01	72.7886	46
THC_THT_c2964	retrotransposon ty3-gypsy sub-class	215	AAX95200	0.035148	-	48	37.7354	60
THC_THT_c2975	l-ascorbate oxidase-like	138	XP_001787008	1.04E-10	GO:0046872	75	6.05E+01	44
THC_THT_c2977	hypothetical protein JCGZ_06008	188	KDP20463	1.93E-05	-	6.30E+01	44.2838	41
THC_THT_c2980	retrotransposon unclassified	219	AAU44126	1.89E-33	GO:0016023	9.80E+01	127.872	72
THC_THT_c2981	conserved hypothetical protein	132	XP_002537474	0.00709563	-	65	38.5058	46
THC_THT_c2982	erythrocyte binding	163	CAE76059	1.61E-23	GO:0005739	94	9.55E+01	53

THC_THT_dn_c2994	cytochrome p450 like_tbp	170	AFK45333	0.00342387	-	73	37.7354	34
THC_THT_c2998	ribosomal protein s11	144	CAA30669	2.04E-23	GO:0006412	1.00E+02	90.8929	47
THC_THT_c3008	hypothetical protein VOLCADRAFT_85069	148	XP_002959854	1.58E-06	-	66	4.66E+01	45
THC_THT_c3011	sensory transduction histidine	107	XP_002538284	0.00219016	-	75	39.6614	37
THC_THT_c3021	retrotransposon unclassified	206	AAP06904	3.99E-39	GO:0004523	98	134.806	67
THC_THT_c3023	protein eceriferum 3-like	106	EEC81004	6.54E-16	GO:0006633	1.00E+02	72.0182	35
THC_THT_c3038	hypothetical protein MTR_0021s0120	115	KEH17345	0.667135	-	62	31.9574	37
THC_THT_c3041	rrna intron-encoded homing endonuclease	143	XP_006405923	4.35E-04	-	42	4.00E+01	64
THC_THT_c3048	hypothetical protein VITISV_020092	189	CAN66945	0.00825053	-	59	39.2762	37
THC_THT_c3052	polyadenylate-binding protein 2-like	133	XP_006661030	2.09E-14	GO:0000166	8.70E+01	72.0182	39
THC_THT_c3059	f-box domain containing expressed	120	EEC69307	2.80E-06	-	6.90E+01	48.1358	42
THC_THT_c3061	cell wall	140	AAV31280	3.54E-14	-	9.70E+01	67.781	35
THC_THT_c3068	nadp-dependant malate dehydrogenase	125	XP_002500781	0.0036571	-	62	39.2762	40
THC_THT_c3070	cell wall-associated hydrolase	338	XP_003637074	0.00467213	-	59	41.2022	44
THC_THT_c3072	retrotransposon ty3-gypsy subclass	181	AAX92789	1.40E-33	GO:0003964	95	119.783	6.00E+01
THC_THT_c3090	gdsl esterase lipase	248	YP_001152214	5.48E-04	-	69	4.08E+01	36
THC_THT_c3091	rrna intron-encoded homing endonuclease	116	XP_003614391	4.43E-04	-	4.90E+01	41.9726	61
THC_THT_c3097	metal transporter nramp5	263	EPS74717	9.60E-08	-	7.70E+01	51.6026	36
THC_THT_c3099	retrotransposon ty3-gypsy subclass	309	AAX92789	1.64E-68	GO:0003964	100	211.075	1.01E+02
THC_THT_c3115	transposon mutator sub-class	114	ABA98628	4.26E-14	GO:0008270	8.60E+01	71.2478	36
THC_THT_c3124	hypothetical protein EUTSA_v10029308mg, partial	150	XP_006397054	0.0907984	-	51	33.113	45

THC_THT_c3125	ycf68 protein	254	YP_588403	4.35E-15	-	76	7.16E+01	52
THC_THT_dn_c3126	hypothetical protein SORBIDRAFT_05g016480	183	XP_002449488	5.37E-07	-	70	4.81E+01	37
THC_THT_c3135	transposon en spm sub-class	279	AAU43928	2.31E-42	GO:0005739	9.60E+01	146.747	90
THC_THT_c3147	retrotransposon ty3-gypsy subclass	182	ABA97128	5.09E-07	-	7.70E+01	49.2914	35
THC_THT_c3149	PREDICTED: uncharacterized protein LOC103956333	191	XP_009366572	3.35E-09	-	8.50E+01	58.151	34
THC_THT_c3153	calcineurin b-like	122	AAM14689	1.93E-08	-	82	5.35E+01	40
THC_THT_c3162	cell wall-associated hydrolase	140	EXC01915	4.34E-08	GO:0009521	6.80E+01	51.2174	45
THC_THT_c3177	dihydrodipicolinate reductase chloroplastic	117	EMS58676	0.888605	-	64	31.187	37
THC_THT_c3185	retrotransposon unclassified	168	ABA93438	1.05E-17	GO:0004523	1.00E+02	82.4185	41
THC_THT_c3211	unknown	122	AFK35083	3.16E-05	-	6.70E+01	42.743	37
THC_THT_c3212	nadh-ubiquinone oxidoreductase	160	XP_002534687	4.69E-10	GO:0016491	66	5.93E+01	51
THC_THT_c3222	retrotransposon ty3-gypsy subclass	241	AAM74298	1.11E-45	GO:0003676	9.80E+01	154.836	74
THC_THT_c3227	dehydration responsive protein	186	BAJ11779	0.0685474	-	56	33.8834	41
THC_THT_c3259	calcineurin b-like protein	227	BAD10612	2.71E-13	GO:0044260	77	6.74E+01	48
THC_THT_c3295	secoisolariciresinol dehydrogenase-like	129	XP_005645747	1.64E-05	GO:0008152	71	4.58E+01	38
THC_THT_c3298	d-lactate dehydrogenase	141	ERN01202	0.372553	-	63	33.8834	38
THC_THT_c3299	scarecrow-like protein 3-like	123	ABA95775	5.65E-20	GO:0006355	97	8.70E+01	40
THC_THT_c3303	retrotransposon ty3-gypsy subclass	242	CAH66150	1.41E-37	GO:0003964	1.00E+02	134.035	61
THC_THT_c3321	transposon mutator sub-class	153	AAL58230	1.95E-22	GO:0003676	90	94.7449	5.00E+01

THC_THT_c3329	calcineurin b-like protein	247	BAC83585	4.72E-12	GO:0005739	76	6.43E+01	50
THC_THT_c3333	retrotransposon ty3-gypsy subclass	176	ABA96739	8.65E-32	GO:0016023	1.00E+02	117.472	55
THC_THT_c3353	senescence-associated protein	162	XP_004515586	3.89E-07	-	7.10E+01	49.2914	35
THC_THT_c3355	probable glutamate carboxypeptidase 2	155	XP_003569769	3.04E-04	-	7.10E+01	43.1282	42
THC_THT_c3356	transposon en spm sub-class	242	CAH66542	1.00E-33	GO:0006508	9.70E+01	126.331	77
THC_THT_c3368	nep1-interacting protein 1-like	114	XP_006662021	6.57E-07	GO:0017089	8.10E+01	48.9062	37
THC_THT_c3370	hypothetical protein OsJ_35769	410	EEE53044	2.75E-14	-	6.00E+01	70.4774	85
THC_THT_c3372	retrotransposon unclassified	144	AAK52122	6.84E-22	GO:0009536	9.70E+01	93.2041	47
THC_THT_c3377	transposon mutator sub-class	158	BAD33754	2.74E-29	GO:0003676	100	1.13E+02	52
THC_THT_c3389	retrotransposon ty3-gypsy subclass	245	BAA75236	1.26E-51	GO:0003676	9.80E+01	170.244	81
THC_THT_c3408	hypothetical protein OsI_36556	108	EAY81384	1.86E-08	GO:0055114	8.50E+01	53.5286	35
THC_THT_c3414	glycine dehydrogenase	140	EAZ13293	1.55E-24	GO:0006546	1.00E+02	101.679	46
THC_THT_c3415	ring u-box domain-containing protein	135	NP_001042730	3.14E-26	GO:0016874	100	103.219	4.40E+01
THC_THT_c3438	cytochrome p450 monooxygenase	123	AGT17357	2.90E-07	-	7.20E+01	48.521	36
THC_THT_c3467	sensory transduction histidine	137	XP_002537785	0.00573482	-	57	38.5058	47
THC_THT_c3469	hypothetical protein L484_002553	165	EXC10889	0.197005	-	73	32.7278	41
THC_THT_c3472	retrotransposon unclassified	178	BAB40824	6.10E-33	GO:0004523	1.00E+02	117.857	59
THC_THT_c3491	unknow protein	285	KJB76688	5.98E-07	-	7.70E+01	49.6766	35
THC_THT_c3512	retrotransposon unclassified	137	AAX92799	9.40E-06	-	67	4.62E+01	40
THC_THT_c3539	malic enzyme	124	NP_001183119	0.318373	-	57	33.8834	35
THC_THT_c3562	Protein MYG1, putative	113	XP_002538853	1.86E-16	-	94	7.55E+01	37

THC_THT_c3566	retrotransposon ty3-gypsy subclass	152	CAE02837	1.80E-26	GO:0003964	9.80E+01	102.834	50
THC_THT_c3572	transposon en spm sub-class	119	AAK43519	1.31E-10	GO:0016491	7.60E+01	58.151	39
THC_THT_c3583	conserved hypothetical protein	116	XP_002539322	0.00779115	-	71	36.1946	38
THC_THT_c3590	unknown protein	112	AAW56878	1.47E-16	GO:0009536	1.00E+02	77.411	37
THC_THT_c3627	retrotransposon ty3-gypsy subclass	254	CAH66866	1.92E-31	GO:0004523	1.00E+02	90.1225	43
THC_THT_c3633	nadh dehydrogenase subunit 9	208	AFB35077	1.69E-26	GO:0008137	1.00E+02	100.908	48
THC_THT_c3662	transposon en spm sub-class	174	AAT58820	1.88E-19	GO:0005739	9.70E+01	81.2629	39
THC_THT_c3663	protein kinase domain containing protein	149	EEC71041	3.79E-12	GO:0016772	7.50E+01	63.5438	44
THC_THT_c3708	cell wall-associated partial	177	XP_003637074	9.67E-04	-	5.50E+01	41.9726	63
THC_THT_c3721	hypothetical protein SORBIDRAFT_01g024221	125	XP_002467304	4.07E-04	-	60	3.97E+01	41
THC_THT_c3723	f1-atpase alpha partial	136	ACD71503	1.55E-23	GO:0046961	1.00E+02	94.3597	45
THC_THT_c3729	conserved hypothetical protein	109	XP_002539248	6.98E-13	GO:0004872	94	6.43E+01	36
THC_THT_c3731	glutathione-regulated potassium-efflux system protein	117	XP_002537122	5.63E-07	GO:1902600	88	5.04E+01	36
THC_THT_c3735	cytochrome b6 f complex subunit iv	135	KJB61291	6.79E-24	GO:0016491	1.00E+02	92.8189	44
THC_THT_c3741	PREDICTED: LOW QUALITY PROTEIN: uncharacterized protein LOC105117228	157	XP_011013113	0.467323	-	67	33.8834	34
THC_THT_c3746	hgwp repeat containing	122	BAD23615	4.69E-16	GO:0003676	9.40E+01	75.0998	39
THC_THT_c3768	rrna intron-encoded homing endonuclease	223	EPS74511	7.34E-09	-	6.20E+01	54.6842	56
THC_THT_c3773	transposon en spm sub-class	158	ABA96171	8.50E-24	GO:0006457	9.40E+01	99.7525	52

THC_THT_c3799	retrotransposon ty3-gypsy subclass	116	AAV31373	3.07E-19	GO:0003676	1.00E+02	86.2705	38
THC_THT_c3817	elongation factor 1 alpha	210	AAB58326	1.43E-39	GO:0003746	1.00E+02	134.806	64
THC_THT_c3833	hypothetical protein	171	AAT93987	9.38E-10	-	8.10E+01	58.151	38
THC_THT_c3844	senescence-associated protein	135	XP_004490393	1.59E-09	-	7.20E+01	55.4546	37
THC_THT_c3907	3-ketoacyl- synthase 4-like	135	KFK30085	4.40E-18	GO:0016020	8.40E+01	78.5666	45
THC_THT_c3921	transposon unclassified	135	CAH67051	3.50E-20	GO:0005739	1.00E+02	88.9669	45
THC_THT_c3924	retrotransposon ty3-gypsy subclass	146	AAP53928	1.67E-25	GO:0003964	9.70E+01	104.76	47
THC_THT_c3925	secreted glucosidase, putative	119	XP_002539266	1.50E-07	GO:0008152	78	5.16E+01	37
THC_THT_c3926	hypothetical protein	142	BAD26548	2.67E-14	-	1.00E+02	69.3218	34
THC_THT_c3934	transposon mutator sub-class	180	ABG22596	5.09E-21	GO:0008270	9.50E+01	92.4337	45
THC_THT_c3939	hgwp repeat containing	265	BAD61608	1.82E-25	GO:0005488	8.60E+01	103.99	66
THC_THT_c3957	beta-galactosidase 3	144	BAB86232	6.04E-23	GO:0005975	9.70E+01	96.6709	47
THC_THT_c3964	30s ribosomal protein s12	147	KEH15656	5.54E-15	GO:0005840	8.00E+01	68.9366	45
THC_THT_c3984	g-type lectin s-receptor-like serine threonine-protein kinase rlk1	138	NP_001051778	2.71E-18	GO:0006468	100	83.5741	38
THC_THT_c4031	transcription initiation factor iie subunit beta-like isoform x2	218	XP_009396361	0.442512	-	54	33.8834	53
THC_THT_c4049	senescence-associated protein	179	XP_003614391	0.211011	-	66	34.6538	45
THC_THT_c4053	retrotransposon unclassified	110	NP_001176697	1.36E-17	GO:0017148	97	7.90E+01	36
THC_THT_c4061	retrotransposon unclassified	320	ABA99783	1.03E-48	GO:0004523	8.90E+01	174.481	99
THC_THT_c4062	glutamate-1-semialdehyde aminotransferase	121	CCO19866	3.11E-13	GO:0006779	8.20E+01	68.1662	40

THC_THT_c4074	retrotransposon ty3-gypsy subclass	326	ABA96740	3.10E-48	GO:0003964	9.30E+01	172.555	101
THC_THT_c4109	udp-glucuronate 4-epimerase 6	118	XP_008222861	8.70E-05	GO:0071704	5.80E+01	43.8986	46
THC_THT_c4115	AC022352_8Hypothetical protein	118	AAK51572	4.01E-05	-	75	4.51E+01	37
THC_THT_c4120	retrotransposon unclassified	106	EEE52328	9.78E-18	-	1.00E+02	81.2629	35
THC_THT_c4125	protein	141	XP_002959989	1.23E-09	GO:0008152	78	5.82E+01	47
THC_THT_c4134	retrotransposon unclassified	116	CAD40418	1.20E-15	GO:0006259	9.40E+01	75.485	38
THC_THT_c4146	retrotransposon unclassified	151	EEC66691	3.50E-16	-	9.20E+01	77.0258	38
THC_THT_c4159	extended synaptotagmin-1	103	EAZ27970	3.84E-16	GO:0016757	1.00E+02	77.0258	34
THC_THT_c4196	unknown	127	AFK35083	1.57E-05	-	7.70E+01	43.5134	35
THC_THT_c4209	hypothetical protein CHLREDRAFT_155068	202	XP_001698950	0.00128434	-	61	39.2762	39
THC_THT_c4211	cf1 alpha subunit of atp synthase	116	BAJ21367	9.37E-14	GO:0046961	8.90E+01	67.3958	38
THC_THT_c4234	probable acyl-activating enzyme peroxisomal	119	XP_002990653	0.00173307	-	71	40.4318	38
THC_THT_c4246	kinesin-like protein nack2 isoform x1	215	XP_011074636	0.381292	-	51	34.2686	58
THC_THT_c4296	partial	213	ADG84787	1.29E-26	GO:0009772	9.60E+01	102.834	51
THC_THT_c4297	retrotransposon ty3-gypsy subclass	246	ABA98370	1.93E-26	GO:0003964	9.60E+01	106.686	52
THC_THT_c4300	disease resistance protein rga3 isoform x1	205	KCW48219	0.589666	-	51	33.8834	60
THC_THT_c4301	probable amino-acid acetyltransferase chloroplastic isoform x3	112	XP_001753117	0.01195	-	69	37.7354	36
THC_THT_c4340	retrotransposon ty3-gypsy subclass	125	AAF00146	4.13E-16	GO:0003723	9.40E+01	73.9442	37
THC_THT_c4342	hypothetical protein JCGZ_00417	225	KDP22830	0.849629	-	56	33.4982	41

THC_THT_c4349	retrotransposon ty3-gypsy subclass	230	CAH65890	2.51E-46	GO:0003676	1.00E+02	160.229	76
THC_THT_c4356	retrotransposon ty3-gypsy subclass	276	CAE05071	9.39E-19	GO:0003964	9.70E+01	83.9593	69
THC_THT_c4398	senescence-associated protein	178	ACU14517	2.01E-07	-	6.10E+01	49.2914	47
THC_THT_c4413	Uncharacterized protein TCM_024341	147	XP_007028506	0.327035	-	55	33.8834	40
THC_THT_c4417	c4-dicarboxylate transport transcriptional regulatory protein	103	XP_002537860	5.03E-05	GO:0050794	76	4.39E+01	34
THC_THT_c4425	senescence-associated partial	122	KEH16543	0.99279	-	58	32.3426	36
THC_THT_c4460	root cap protein 1-like	294	BAI39684	3.39E-09	-	8.20E+01	56.6102	86
THC_THT_c4485	atp synthase f0 subunit 1	262	ABD64407	2.08E-53	GO:0046961	1.00E+02	174.866	87
THC_THT_c4495	retrotransposon ty3-gypsy subclass	370	BAB40828	9.85E-83	GO:0003964	9.90E+01	247.669	121
THC_THT_c4497	cytochrome partial	128	EEE59971	2.76E-17	GO:0016023	9.50E+01	77.7962	42
THC_THT_c4507	hypothetical protein	121	AAM93715	8.59E-10	-	6.20E+01	56.225	51
THC_THT_c4509	hypothetical protein SORBIDRAFT_10g027275	130	XP_002438856	4.51E-10	-	88	5.58E+01	36
THC_THT_c4542	transposon mutator sub-class	120	NP_001063934	3.23E-16	GO:0004553	100	7.78E+01	35
THC_THT_c4551	probable dna gyrase subunit chloroplastic mitochondrial-like	146	EPS70841	7.45E-06	-	5.80E+01	47.7506	46
THC_THT_c4581	hypothetical protein SELMODRAFT_139061	130	XP_002994726	1.89E-06	-	73	4.62E+01	34
THC_THT_c4582	PREDICTED: RRP12-like protein-like	120	XP_004971318	0.0423837	-	55	36.5798	38
THC_THT_c4594	ac051624_3 retroelement	104	CAE05536	0.0325275	-	70	36.5798	34
THC_THT_c4610	retrotransposon unclassified	108	CAE04968	9.98E-14	GO:0003676	97	6.59E+01	35
THC_THT_c4626	leucine rich protein	197	ABH09321	0.0921155	-	71	35.4242	35
THC_THT_c4648	transposon en spm sub-class	148	CAH65850	9.00E-19	GO:0005739	9.70E+01	85.1149	41

THC_THT_c4650	bifunctional polymyxin resistance arna protein	111	XP_001783855	0.00153794	-	54	40.0466	37
THC_THT_c4655	hypothetical protein EUTSA_v10028342mg	108	XP_006405913	0.47026	-	56	30.8018	39
THC_THT_c4663	retrotransposon ty3-gypsy subclass	258	ABA97779	2.95E-44	GO:0003964	1.00E+02	154.451	71
THC_THT_c4706	nitrate reductase	113	EEC68274	5.22E-19	GO:0050660	1.00E+02	78.9518	37
THC_THT_c4713	dihydroxy-acid dehydratase	109	XP_002957711	0.0680014	-	60	35.4242	35
THC_THT_c4749	hypothetical protein MTR_2g059740	116	XP_003595724	2.00E-07	-	7.50E+01	49.2914	37
THC_THT_c4777	amp-dependent synthetase and ligase family isoform 2	131	XP_002534739	9.87E-09	GO:0070918	80	5.62E+01	42
THC_THT_c4782	2-nitropropane dioxygenase-like protein	149	XP_002505871	0.0814701	-	63	35.4242	44
THC_THT_c4797	hypothetical protein CHLPROCp009, partial	187	AGB85039	2.07E-09	-	8.50E+01	55.4546	35
THC_THT_c4814	rna polymerase beta subunit	150	XP_002462089	1.88E-17	GO:0003899	95	7.55E+01	42
THC_THT_c4826	low quality protein: histidine kinase 3	124	KGN46466	0.67332	-	64	32.7278	34
THC_THT_c4835	hth-type transcriptional regulator	104	XP_002535661	3.80E-12	GO:0003700	94	6.35E+01	34
THC_THT_c4850	probable copper-transporting atpase hma5	116	XP_009355182	2.99E-08	GO:0005507	8.50E+01	54.299	34
THC_THT_c4865	retrotransposon ty3-gypsy subclass	135	CAE02295	3.13E-19	GO:0003964	97	8.32E+01	40
THC_THT_c4866	hypothetical protein EUGRSUZ_I01857	269	KCW56103	3.13E-04	-	8.00E+01	41.9726	36
THC_THT_dn_c4870	hypothetical protein (mitochondrion)	244	AEZ03741	1.47E-20	GO:0046933	100	8.70E+01	41
THC_THT_c4874	retrotransposon unclassified	155	CAE76059	9.40E-14	-	100	6.82E+01	34
THC_THT_c4892	retrotransposon ty3-gypsy subclass	192	BAH80067	7.82E-39	GO:0003676	9.60E+01	136.346	64

THC_THT_c4915	hypothetical protein CISIN_1g038654mg, partial	141	KDO64261	2.65E-05	-	6.50E+01	43.5134	35
THC_THT_c4929	transposable element	149	AAN74831	3.51E-27	GO:0005739	1.00E+02	106.301	49
THC_THT_c4960	hypothetical protein CARUB_v10019941mg	115	XP_006300848	0.519904	-	59	33.113	44
THC_THT_c4981	enolase	114	ABR25736	8.56E-10	GO:0006096	7.80E+01	56.6102	38
THC_THT_c4984	protein kinase	119	AAX94990	5.05E-12	GO:0006468	89	6.47E+01	37
THC_THT_c4986	Os03g0604700	103	NP_001050633	9.43E-16	-	100	7.05E+01	34
THC_THT_c5062	unknown	105	ACR37398	3.72E-05	-	7.00E+01	42.3578	37
THC_THT_c5066	OSJNBa0028M15.22	156	CAE05830	0.0013625	-	69	41.5874	36
THC_THT_c5080	atp synthase subunit beta	200	XP_003627732	5.74E-04	-	7.20E+01	43.1282	37
THC_THT_c5099	jq0280 hypothetical 12k protein (trna intron) - rice chloroplast	142	YP_052806	2.95E-22	GO:0009507	97	88.5817	44
THC_THT_c5136	P0660F12.26	163	BAB63860	0.0154659	-	57	38.1206	40
THC_THT_c5137	hypothetical protein OsI_01807	109	EEC70591	1.17E-16	GO:0016020	1.00E+02	77.7962	36
THC_THT_c5187	short-chain dehydrogenase reductase 4	123	NP_189570	0.229631	-	55	33.8834	38
THC_THT_c5194	retrotransposon ty3-gypsy sub-class	172	BAD05345	2.37E-16	GO:0005739	9.20E+01	75.0998	39
THC_THT_c5216	H0522A01.8	146	CAH67737	5.04E-09	-	85	5.70E+01	40
THC_THT_c5252	protein	258	XP_002464700	1.12E-15	-	94	72.0182	35
THC_THT_c5257	atp-binding cassette subfamily member 1	104	ERN08207	3.93E-04	-	7.00E+01	41.5874	34
THC_THT_c5261	centromere-specific	144	AAX96117	7.30E-24	GO:0003676	87	9.90E+01	48
THC_THT_c5264	l-type lectin-domain containing receptor kinase -like	118	NP_001044432	2.65E-21	GO:0030247	100	91.6633	39
THC_THT_c5266	protein kinase	181	AGU99598	3.05E-12	GO:0006468	9.40E+01	66.2402	35
THC_THT_c5282	retrotransposon protein	149	ABR26094	0.00555048	-	70	36.965	34
THC_THT_c5292	transposon mutator sub-class	143	CAH67627	2.12E-22	GO:0008270	9.30E+01	95.9005	47

THC_THT_c5328	hypothetical protein	113	BAC84120	1.67E-06	-	7.00E+01	46.595	34
THC_THT_c5329	retrotransposon unclassified	155	AAL31658	1.50E-04	-	84	41.9726	5.00E+01
THC_THT_c5331	predicted protein	146	XP_003064993	0.641254	-	61	31.9574	34
THC_THT_c5339	cell wall	136	BAD22424	1.72E-14	GO:0009536	9.00E+01	71.2478	43
THC_THT_c5343	hypothetical protein	150	BAI39916	0.0197335	-	83	35.039	37
THC_THT_c5349	retrotransposon unclassified	125	CAD40060	3.83E-19	GO:0003676	9.50E+01	83.9593	41
THC_THT_c5383	photosystem i subunit vii	130	YP_008474526	0.0566928	-	52	33.4982	46
THC_THT_c5399	pentatricopeptide repeat-containing protein at4g21300-like	104	EMT11700	1.08E-08	-	8.20E+01	53.5286	34
THC_THT_c5411	ribosomal protein s10	359	XP_003610227	6.04E-04	-	5.60E+01	43.8986	57
THC_THT_c5429	retrotransposon ty3-gypsy subclass	204	CAD40089	1.29E-29	GO:0003964	9.80E+01	114.005	53
THC_THT_c5453	1-deoxy-d-xylulose 5-phosphate synthase	118	XP_002441088	1.11E-17	GO:0046872	100	8.13E+01	39
THC_THT_c5487	omega-6 fatty acid desaturase	149	EXB24274	0.49221	-	55	33.4982	38
THC_THT_c5519	PREDICTED: uncharacterized protein LOC103849935	165	XP_009124891	0.314084	-	61	33.8834	39
THC_THT_c5550	transposon en spm sub-class	174	AAX95003	2.46E-29	GO:0005507	98	112.464	5.40E+01
THC_THT_c5587	retrotransposon ty3-gypsy subclass	109	AAV32123	6.00E-06	GO:0044260	6.80E+01	47.7506	35
THC_THT_c5612	conserved hypothetical protein	183	XP_002524745	0.106445	-	60	34.6538	46
THC_THT_c5618	transposon en spm sub-class	226	AAM74425	4.25E-38	GO:0005739	93	1.40E+02	74
THC_THT_c5623	nac domain-containing protein 21 22-like	168	EAZ02065	1.51E-19	GO:0006355	1.00E+02	84.7297	49
THC_THT_c5665	disease resistance rpp13-like protein 1	173	EAY85175	5.07E-32	GO:0016023	9.80E+01	122.865	57

THC_THT_c5667	uncharacterized mitochondrial protein g00810-like	105	XP_004986808	2.56E-15	GO:0006511	9.70E+01	75.0998	35
THC_THT_c5671	nhl repeat-containing protein 2-like	131	XP_010313619	5.59E-05	GO:0044249	6.90E+01	45.0542	39
THC_THT_c5672	hypothetical protein CHLNCDRAFT_12089, partial	108	XP_005850354	0.116868	-	61	33.8834	34
THC_THT_c5690	50s ribosomal protein l2-b	123	XP_003605596	7.28E-07	-	7.50E+01	50.0618	36
THC_THT_c5711	cell wall	223	AAP53835	2.13E-39	GO:0003676	9.60E+01	136.346	66
THC_THT_c5727	transposon mutator sub-class	251	BAD88394	5.87E-42	GO:0051287	9.00E+01	143.28	77
THC_THT_c5730	aminotransferase-like protein	131	AAT85305	3.51E-17	GO:0008152	97	8.01E+01	41
THC_THT_c5735	hypothetical protein MTR_0082s0080	157	KEH16849	4.98E-12	-	9.10E+01	63.1586	34
THC_THT_c5748	protein	144	XP_002884233	0.00699428	-	65	36.1946	35
THC_THT_c5751	hypothetical protein OsI_16502	121	EEC77565	1.91E-19	-	1.00E+02	82.0333	39
THC_THT_c5804	retrotransposon unclassified	137	CAH67688	2.35E-21	GO:0005739	100	9.24E+01	43
THC_THT_c5833	retrotransposon unclassified	158	AAT69668	4.66E-11	GO:0003676	9.30E+01	62.3882	49
THC_THT_c5887	retrotransposon unclassified	213	ABA94385	1.52E-09	GO:0003723	6.70E+01	59.6918	52
THC_THT_c5891	hypothetical protein	226	BAB17167	1.38E-16	-	91	7.51E+01	37
THC_THT_c5892	hypothetical protein	145	BAC79940	3.98E-17	GO:0003746	8.80E+01	77.0258	45
THC_THT_c5894	hypothetical protein OsJ_24971	210	EEE67517	1.95E-06	-	6.00E+01	31.9574	35
THC_THT_c5899	retrotransposon ty3-gypsy subclass	195	NP_001050456	1.25E-30	GO:0004523	88	1.17E+02	68
THC_THT_c5905	epstein-barr virus ebna-1-like protein	179	AAP50964	3.78E-06	GO:0005739	79	4.93E+01	34
THC_THT_c5914	uncharacterized mitochondrial protein g00810-like	158	NP_001056818	1.72E-28	GO:0016021	100	1.09E+02	50
THC_THT_c5921	retrotransposon ty3-gypsy subclass	181	AAL93077	2.58E-06	GO:0004523	8.70E+01	48.1358	41
THC_THT_c5967	OSJNBb0089K06.8	315	CAD39687	4.21E-37	GO:0016023	9.40E+01	131.339	67

THC_THT_c5975	hypothetical protein CHLREDRAFT_173858	105	XP_001694376	0.0846589	-	65	34.6538	35
THC_THT_c6011	60s ribosomal protein l2-like protein	141	CDY70488	0.00603359	-	65	36.965	43
THC_THT_c6022	nac domain-containing protein 102-like	122	EEE60165	3.05E-18	GO:0006355	1.00E+02	80.8777	35
THC_THT_c6030	fibroin heavy chain-like	157	XP_006646135	0.241465	-	60	34.6538	45
THC_THT_c6046	ac069158_5 transposable element	156	AAK98693	1.56E-10	-	6.70E+01	61.6178	46
THC_THT_c6072	retrotransposon ty3-gypsy subclass	194	CAE04995	5.28E-21	GO:0004523	9.50E+01	79.337	44
THC_THT_c6075	proline oxidase	162	EEE51366	6.89E-10	-	6.40E+01	58.9214	42
THC_THT_c6086	retrotransposon line subclass	104	CAH67349	5.60E-16	GO:0003964	1.00E+02	75.485	34
THC_THT_c6110	abc transporter b family member chloroplastic	161	XP_002539706	8.60E-08	GO:0044699	74	5.16E+01	50
THC_THT_c6145	dihydroxy-acid dehydratase	197	KIY95278	1.83E-18	GO:0044763	7.70E+01	81.2629	61
THC_THT_c6237	senescence-associated protein	134	XP_006279314	5.23E-07	-	68	4.78E+01	35
THC_THT_c6243	hypothetical protein EUTSA_v10028274mg	185	XP_006405909	9.38E-06	-	49	4.54E+01	59
THC_THT_c6252	hgwp repeat containing	165	BAD45243	1.42E-04	-	7.30E+01	43.8986	34
THC_THT_c6270	retrotransposon ty3-gypsy subclass	155	CAE02456	2.96E-17	GO:0004523	8.70E+01	78.1814	47
THC_THT_c6291	conserved hypothetical protein	139	XP_002540423	4.87E-05	-	68	4.24E+01	38
THC_THT_c6305	potassium transporter 5-like	212	XP_003610227	0.00911544	-	65	39.2762	35
THC_THT_c6315	retrotransposon ty3-gypsy subclass	145	XP_008780563	5.40E-15	GO:0004523	9.10E+01	71.2478	35
THC_THT_c6360	retrotransposon ty3-gypsy subclass	183	ABA93544	9.39E-18	GO:0004523	1.00E+02	82.4185	40
THC_THT_c6365	40s ribosomal protein s3a-like	113	NP_001049289	2.05E-18	GO:0003735	100	80.4925	37

THC_THT_c6380	shikimate o-hydroxycinnamoyltransferase-like	114	EEC84594	6.81E-19	GO:0016747	1.00E+02	82.0333	38
THC_THT_c6390	transposon mutator sub-expressed	130	ABF95394	1.75E-12	GO:0008270	9.70E+01	67.0106	34
THC_THT_c6415	pathogenesis-related protein expressed	137	EEC69482	4.10E-06	-	7.00E+01	47.7506	44
THC_THT_c6419	predicted protein	108	XP_002503468	0.272353	-	60	33.4982	41
THC_THT_c6431	retrotransposon unclassified	129	AAV43984	2.10E-12	GO:0090502	88	6.70E+01	36
THC_THT_c6437	voltage-dependent calcium channel type d subunit alpha-1	174	EAY85975	4.03E-17	-	1.00E+02	77.7962	36
THC_THT_c6462	metal transporter nramp5	395	EPS74717	2.01E-08	-	6.10E+01	27.335	36
THC_THT_c6487	senescence-associated partial	133	XP_003614383	0.00394553	-	67	39.6614	34
THC_THT_c6526	conserved hypothetical protein	121	XP_002537558	0.130059	-	71	34.6538	39
THC_THT_c6545	calcineurin b-like protein	132	AAX96179	3.80E-17	GO:0004523	95	7.70E+01	40
THC_THT_c6547	transposon en spm sub-class	128	CAD40282	2.64E-09	GO:0006508	8.30E+01	57.3806	37
THC_THT_c6557	isovaleryl- mitochondrial	122	EMS49058	1.54E-04	GO:0016627	6.60E+01	43.1282	42
THC_THT_c6559	hypothetical protein B456_003G081400	152	KJB19027	0.470533	-	56	33.113	37
THC_THT_c6571	retrotransposon ty3-gypsy subclass	152	CAE05905	1.49E-28	GO:0003964	9.80E+01	114.005	50
THC_THT_c6574	retrotransposon unclassified	312	ABF95985	5.22E-46	GO:0008270	9.70E+01	161.77	93
THC_THT_c6579	retrotransposon ty3-gypsy subclass	208	XP_008779617	4.30E-31	GO:0003964	9.50E+01	121.324	63
THC_THT_c6598	mfs general substrate transporter	141	XP_002535255	1.85E-06	-	73	4.93E+01	38
THC_THT_c6639	succinate semialdehyde dehydrogenase	136	XP_002961441	0.00197446	-	61	40.4318	42

THC_THT_c6645	glutamyl-trna amidotransferase subunit chloroplastic mitochondrial	138	XP_010467088	1.83E-09	GO:0016556	7.30E+01	57.7658	45
THC_THT_c6657	PREDICTED: uncharacterized protein LOC100807658 isoform X3	117	XP_006600374	0.839205	-	52	32.3426	34
THC_THT_c6661	pot family expressed	114	EAY78225	2.38E-16	GO:0016021	1.00E+02	77.0258	37
THC_THT_c6664	transposon mutator sub-class	121	EEC75372	1.06E-18	GO:0016020	1.00E+02	81.2629	39
THC_THT_c6672	tpa: cytidine deoxycytidylate deaminase family protein	103	XP_008666297	0.00984786	-	67	35.8094	34
THC_THT_c6674	retrotransposon unclassified	135	ABF94159	7.86E-16	GO:0004523	9.00E+01	77.0258	43
THC_THT_c6695	unnamed protein product	117	BAG90763	0.714069	-	62	31.5722	35
THC_THT_c6710	pentatricopeptide repeat-containing protein at1g09900- partial	109	EEE63625	1.85E-09	GO:0016023	8.00E+01	56.9954	36
THC_THT_c6715	cytochrome p450	122	AAO34504	1.12E-09	-	72	5.58E+01	40
THC_THT_c6744	phosphoribosyltransferase family protein	121	XP_011014724	1.50E-05	GO:0016757	7.60E+01	46.595	39
THC_THT_c6751	latex-abundant family	116	EAY90295	1.42E-19	GO:0006508	1.00E+02	84.7297	38
THC_THT_c6758	prephenate dehydratase-like	126	EAZ02033	8.54E-20	GO:0009094	9.70E+01	82.4185	40
THC_THT_c6780	retrotransposon unclassified	111	AAV31383	4.06E-16	GO:0003676	9.70E+01	77.0258	36
THC_THT_c6785	isoleucyl-trna cytoplasmic	119	EEE56452	3.45E-17	GO:0005829	1.00E+02	80.1073	34
THC_THT_c6801	PREDICTED: uncharacterized protein LOC105119394	167	XP_011015834	2.09E-09	-	7.50E+01	57.7658	52
THC_THT_c6817	probable uridine nucleosidase 1-like	113	EMT19270	1.48E-14	GO:0045437	8.80E+01	70.4774	36

THC_THT_c6835	probable glutathione s-transferase gstu1-like	108	NP_001059595	7.04E-15	GO:0008152	94	70.4774	36
THC_THT_c6863	hypothetical protein	130	BAD38257	4.74E-15	-	79	7.05E+01	43
THC_THT_c6870	retrotransposon ty3-gypsy subclass	149	AAM14678	4.50E-22	GO:0003964	95	92.4337	4.60E+01
THC_THT_c6872	hypothetical protein	149	BAD27843	1.04E-16	-	92	7.36E+01	40
THC_THT_c6883	hypothetical protein M569_00237	175	EPS74520	1.30E-06	-	7.20E+01	48.1358	44
THC_THT_c6895	conserved hypothetical protein	115	XP_002540045	1.74E-13	GO:0005737	94	6.70E+01	35
THC_THT_c6946	retrotransposon unclassified	158	AAL83340	4.32E-28	GO:0005739	96	104.76	5.20E+01
THC_THT_c6955	hypothetical protein (mitochondrion)	156	YP_009049820	3.64E-13	-	68	65.0846	5.10E+01
THC_THT_c6967	hypothetical protein SORBIDRAFT_1211s002020	139	XP_002488956	8.39E-11	-	80	57.7658	40
THC_THT_c6995	transposon mutator sub-class	283	ABA98631	6.13E-62	GO:0051287	1.00E+02	207.994	93
THC_THT_c7022	hypothetical protein MTR_5g050960	170	XP_003614381	6.21E-07	-	6.20E+01	46.9802	43
THC_THT_c7033	retrotransposon ty3-gypsy subclass	176	ABA93560	1.17E-20	GO:0003676	7.90E+01	91.2781	58
THC_THT_c7050	chloroplast psii associated light-harvesting complex ii protein	112	KCW70610	1.98E-15	GO:0051738	1.00E+02	71.633	36
THC_THT_c7061	hypothetical protein TRIUR3_09014	147	EMS53098	1.52E-15	-	8.10E+01	73.1738	48
THC_THT_c7073	hypothetical protein	120	BAD72370	7.05E-04	-	8.70E+01	39.6614	39
THC_THT_c7098	hypothetical protein B456_007G194600	122	KJB43296	4.31E-04	-	5.60E+01	39.6614	44
THC_THT_c7110	retrotransposon unclassified	104	AAL83336	9.89E-13	GO:0008270	97	66.2402	3.40E+01
THC_THT_c7115	retrotransposon ty3-gypsy subclass	147	ABA98133	2.69E-17	-	9.50E+01	80.8777	43
THC_THT_c7117	retrotransposon ty3-gypsy subclass	144	ABA92247	2.84E-21	GO:0003964	9.50E+01	92.4337	47

THC_THT_c7120	40s ribosomal protein s21	147	EEC75241	6.99E-28	GO:0000461	100	1.04E+02	48
THC_THT_c7124	retrotransposon ty3-gypsy subclass	141	EEC78490	4.13E-25	GO:0004523	1.00E+02	102.449	46
THC_THT_c7136	rrna intron-encoded homing endonuclease	161	XP_003614387	0.371571	-	64	34.2686	37
THC_THT_c7139	xs domain containing	134	ABF97042	9.53E-08	-	8.70E+01	51.9878	41
THC_THT_c7167	senescence-associated protein	173	XP_003614377	1.90E-05	-	7.50E+01	46.9802	36
THC_THT_c7173	PREDICTED: laccase-17-like	128	XP_009595163	5.68E-14	GO:0005507	8.80E+01	70.4774	34
THC_THT_c7187	inositol partial	113	XP_005650085	0.00315571	-	65	38.891	35
THC_THT_c7212	atp synthase subunit beta	237	ACU24256	1.12E-14	-	6.00E+01	69.3218	66
THC_THT_c7215	ac068923_2 membrane-associated protein	162	BAD69019	9.44E-23	GO:0005739	9.00E+01	90.8929	52
THC_THT_c7238	transposon mutator sub-class	212	ABA97178	1.59E-25	GO:0003676	9.80E+01	105.916	57
THC_THT_c7261	hth-type transcriptional repressor	162	XP_002539909	3.27E-08	-	71	5.24E+01	49
THC_THT_c7308	mitochondrial-like isoform x2	118	CCO16193	7.32E-10	GO:0043231	7.80E+01	58.5362	37
THC_THT_c7316	acetolactate synthase	144	XP_002511176	0.00204629	-	68	40.4318	35
THC_THT_c7349	rrna intron-encoded homing endonuclease	241	BAD07869	1.44E-06	-	7.00E+01	45.4394	34
THC_THT_c7352	Os01g0749000	197	NP_001044244	2.10E-17	GO:0005739	100	78.9518	39
THC_THT_c7374	aldehyde dehydrogenase family 7 member a1	115	NP_001063281	1.43E-18	GO:0009873	100	83.1889	38
THC_THT_c7376	predicted protein	108	BAK04968	0.490515	-	57	33.113	35
THC_THT_c7377	hypothetical protein	121	AAV24755	0.00241103	-	67	39.2762	40
THC_THT_c7386	rrna intron-encoded homing endonuclease	263	XP_003614391	7.53E-06	-	6.20E+01	48.9062	50
THC_THT_c7390	60s ribosomal protein l44	137	XP_008812258	8.48E-19	GO:0005840	1.00E+02	79.337	45
THC_THT_c7391	abc transporter i family member chloroplastic	132	XP_010031331	3.88E-04	-	68	41.9726	35

THC_THT_c7409	retrotransposon unclassified	133	AAQ56541	0.0462288	-	92	36.1946	42
THC_THT_c7420	rna polymerase beta subunit	142	EEC75033	2.78E-23	GO:0003899	1.00E+02	97.8265	47
THC_THT_c7445	atp-dependent clp protease proteolytic subunit	176	AFW86530	8.11E-35	GO:0045893	1.00E+02	121.709	58
THC_THT_c7454	conserved hypothetical protein	111	XP_002538815	1.92E-09	-	80	5.66E+01	36
THC_THT_c7457	haloacid dehalogenase-like hydrolase	113	ADF43173	0.103324	-	63	34.6538	36
THC_THT_c7463	retrotransposon ty3-gypsy subclass	132	AAM08548	4.62E-15	GO:0004523	9.40E+01	73.1738	35
THC_THT_c7498	multidrug resistance outer membrane protein mdtp	112	KEH15190	1.19E-05	GO:0006810	7.20E+01	45.4394	37
THC_THT_c7510	retrotransposon ty1-copia subclass	192	ABA97461	8.45E-36	GO:0003676	9.50E+01	127.487	64
THC_THT_c7518	type vi secretion vc-a0108 family partial	124	KEH15576	5.62E-04	-	7.10E+01	41.9726	39
THC_THT_c7522	hypothetical protein OsJ_24971	220	EEE67517	8.59E-10	-	6.90E+01	59.6918	52
THC_THT_c7524	retrotransposon ty3-gypsy subclass	345	AAV35814	6.87E-25	GO:0004523	7.50E+01	105.916	78
THC_THT_c7528	hat family dimerisation domain containing protein	116	NP_001173714	8.25E-12	GO:0003676	91	63.929	36
THC_THT_c7551	ras-related protein rab-7b	124	NP_001269169	0.101237	-	64	34.6538	39
THC_THT_dn_c7561	pr1-like protein	188	BAD22911	4.69E-07	-	6.60E+01	49.2914	50
THC_THT_c7565	retrotransposon ty3-gypsy subclass	133	ABA97516	2.46E-22	GO:0003964	1.00E+02	95.5153	44
THC_THT_c7575	adenine nucleotide alpha hydrolase-like domain kinase	124	XP_004498354	0.0431245	-	58	36.5798	39
THC_THT_c7579	xs domain containing	108	ABF94011	8.07E-16	GO:0016788	1.00E+02	71.2478	35
THC_THT_c7599	predicted protein	109	XP_001786704	2.27E-05	GO:0015031	78	4.54E+01	37

THC_THT_c7602	vacuolar protein sorting-associated protein 2 homolog 3-like	152	XP_009111131	0.110795	-	47	33.113	48
THC_THT_c7606	retrotransposon ty3-gypsy subclass	205	CAD39763	1.19E-30	GO:0003676	9.50E+01	82.8037	41
THC_THT_c7617	37s ribosomal protein mitochondrial	161	XP_002540443	2.07E-14	GO:0070918	85	6.74E+01	42
THC_THT_c7624	reticulon-like protein b2	117	AFW80459	4.62E-14	GO:0016021	8.80E+01	68.5514	35
THC_THT_c7692	unnamed protein product	208	CDM84611	1.97E-12	-	8.60E+01	64.3142	37
THC_THT_c7695	3-isopropylmalate small subunit	129	XP_003078193	1.84E-09	GO:0008152	8.10E+01	58.151	38
THC_THT_c7744	OSJNBb0068N06.23	106	CAE75896	1.52E-13	-	9.40E+01	68.9366	35
THC_THT_c7748	retrotransposon unclassified	123	ABA99730	1.18E-14	-	9.40E+01	72.7886	35
THC_THT_c7778	retrotransposon ty3-gypsy subclass	195	ABA99150	8.45E-21	GO:0003964	1.00E+02	83.5741	36
THC_THT_c7797	hypothetical protein MTR_5g051130	148	XP_003614394	0.876754	-	62	32.7278	40
THC_THT_c7833	orf117_6 gene product	240	XP_002531736	5.22E-09	-	83	5.43E+01	36
THC_THT_c7847	urate oxidase	158	ABD03944	0.667439	-	54	32.7278	42
THC_THT_c7849	transposon unclassified	106	AAN06841	2.17E-16	-	100	7.39E+01	34
THC_THT_c7872	hypothetical protein MTR_5g051170	147	XP_003614398	0.109945	-	62	35.4242	37
THC_THT_c7874	copper amine oxidase	181	XP_001774529	0.00345897	-	62	40.4318	43
THC_THT_c7879	retrotransposon ty3-gypsy subclass	216	ABA97793	4.74E-24	GO:0003964	1.00E+02	101.679	46
THC_THT_c7880	hypothetical protein OsJ_00071	122	EEE53727	6.43E-16	-	9.40E+01	71.2478	37
THC_THT_c7891	ulp1 protease-like	226	BAD53929	3.60E-20	GO:0005739	8.90E+01	83.1889	48
THC_THT_c7917	isocitrate dehydrogenase citrate hydrolyase	187	XP_002507787	1.58E-15	GO:0016616	73	7.66E+01	56
THC_THT_c7933	retrotransposon unclassified	165	CAE04098	2.96E-12	GO:0004523	9.40E+01	67.0106	34
THC_THT_c7937	metallocarboxypeptidase inhibitor	266	EPS70027	9.97E-14	GO:0008152	6.50E+01	67.0106	55

THC_THT_c7938	udp-glycosyltransferase 91c1-like	120	XP_010050452	0.191628	-	59	33.4982	37
THC_THT_c7980	f-box domain containing protein	126	AAM74325	3.20E-19	-	97	8.43E+01	40
THC_THT_c7993	protein aspartic protease in guard cell 1-like	110	NP_001168599	1.54E-16	GO:0004190	100	7.59E+01	36
THC_THT_c7994	endoribonuclease l-psp	109	KDD73550	0.209354	-	73	32.7278	34
THC_THT_c8008	hypothetical protein SORBIDRAFT_1376s002010	145	XP_002488946	0.00721322	-	61	36.1946	34
THC_THT_c8029	predicted protein	148	BAK07175	0.136605	-	51	32.7278	47
THC_THT_c8031	rrna intron-encoded homing endonuclease	125	XP_003614387	0.00133714	-	70	41.2022	37
THC_THT_c8036	senescence-associated partial	133	XP_003614355	0.00718943	-	73	38.891	34
THC_THT_c8057	atp synthase subunit beta	188	XP_002488950	2.19E-13	-	80	6.51E+01	41
THC_THT_c8059	hypothetical protein B456_012G017800	221	KJB74997	0.209051	-	49	35.039	73
THC_THT_c8061	atp synthase subunit c family protein	151	ABA99282	3.29E-14	GO:0015078	1.00E+02	70.4774	34
THC_THT_c8062	retrotransposon ty3-gypsy subclass	117	ABF95973	2.56E-07	-	7.00E+01	51.6026	41
THC_THT_c8065	hgwp repeat containing	182	BAC55985	4.31E-17	GO:0003676	9.50E+01	77.411	43
THC_THT_c8075	hypothetical protein ARALYDRAFT_493634	148	XP_002870446	0.0155376	-	60	37.7354	40
THC_THT_dn_c8098	hypothetical protein B456_005G020800, partial	294	KJB27998	0.023884	-	63	37.3502	41
THC_THT_c8108	retrotransposon unclassified	236	AAO66569	6.76E-15	GO:0003964	81	7.24E+01	43
THC_THT_c8119	rrna intron-encoded homing endonuclease	133	XP_003614394	0.879792	-	56	32.7278	44
THC_THT_c8122	senescence-associated protein	159	BAB33421	3.45E-10	-	8.20E+01	58.9214	34
THC_THT_c8125	hypothetical protein MTR_3g035650	209	XP_003599577	5.10E-11	-	60	6.05E+01	63

THC_THT_c8149	senescence-associated protein	130	XP_003064992	1.20E-12	-	50	6.39E+01	78
THC_THT_c8165	retrotransposon unclassified	307	AAM01178	6.55E-11	-	94	6.16E+01	39
THC_THT_c8184	retrotransposon ty3-gypsy subclass	174	ABA97310	6.95E-11	GO:0003964	7.10E+01	58.5362	42
THC_THT_c8186	hypothetical protein MTR_5g051150	141	XP_003614396	0.2373	-	52	34.6538	38
THC_THT_c8201	retrotransposon ty3-gypsy subclass	189	AAQ56521	9.32E-20	GO:0003964	9.70E+01	87.4261	39
THC_THT_c8213	retrotransposon ty3-gypsy sub-class	150	AAX95930	1.04E-22	GO:0003676	89	9.67E+01	49
THC_THT_c8231	retrotransposon unclassified	229	CAE05321	9.26E-23	-	6.40E+01	97.4413	87
THC_THT_c8304	PREDICTED: uncharacterized protein LOC103704564	117	XP_008786138	0.795494	-	59	32.7278	37
THC_THT_c8323	AF119222_12hypothetical protein	201	AAD27680	5.07E-08	-	8.00E+01	54.6842	35
THC_THT_c8359	splicing coactivator subunit-like protein	163	BAC83409	0.00407132	-	82	37.3502	35
THC_THT_c8373	hypothetical protein VOLCADRAFT_85069	158	XP_002959854	4.26E-10	-	75	5.62E+01	40
THC_THT_c8388	hypothetical protein MTR_5g051050, partial	109	XP_003614388	0.0700928	-	62	35.4242	35
THC_THT_c8486	hypothetical protein POPTR_0008s21830g	159	XP_006380094	9.75E-05	-	65	4.24E+01	41
THC_THT_c8565	senescence-associated protein	136	XP_007099561	5.13E-05	-	67	4.24E+01	34
THC_THT_c8626	predicted protein	151	XP_001785975	7.50E-10	-	85	5.55E+01	34
THC_THT_c8638	rrna intron-encoded homing endonuclease	146	XP_003614387	4.58E-07	-	7.30E+01	51.6026	38
THC_THT_c8682	senescence-associated protein, putative	165	KEH17023	4.33E-11	-	7.70E+01	59.3066	40
THC_THT_c8716	hypothetical protein VITISV_001840	149	CAN65763	0.0278712	-	63	36.965	36
THC_THT_c8717	rrna intron-encoded homing endonuclease	166	XP_006363639	1.18E-11	GO:0044237	6.50E+01	50.447	43

THC_THT_c8766	senescence-associated protein	235	BAB33421	5.96E-14	-	7.00E+01	70.0922	58
THC_THT_c8801	cytochrome p450 like_tbp	138	XP_010046011	7.62E-07	-	7.60E+01	50.0618	34
THC_THT_c8830	stromal 70 kda heat shock-related chloroplastic-like	205	XP_004960697	0.312033	-	52	34.6538	55
THC_THT_c8833	nicotianamine aminotransferase a-like	163	XP_004952311	0.763915	-	55	32.7278	38
THC_THT_c8857	aldose 1-epimerase	119	XP_005643425	0.793127	-	58	32.3426	39
THC_THT_c8873	PREDICTED: uncharacterized protein LOC103933723	125	XP_009341691	0.0178956	-	54	37.3502	35
THC_THT_c8874	hypothetical protein EUTSA_v10028116mg	191	XP_006405921	1.87E-08	-	82	5.24E+01	34
THC_THT_c8877	hypothetical protein B456_011G262200, partial	138	KJB73948	0.00801513	-	57	36.1946	40
THC_THT_c8901	hypothetical protein SORBIDRAFT_0351s002020	148	XP_002489033	1.43E-14	-	94	6.74E+01	36
THC_THT_c8960	hypothetical protein SORBIDRAFT_0016s002240	143	XP_002489152	1.46E-05	-	63	4.54E+01	49
THC_THT_c8973	transposon en spm sub-class	122	BAD67783	7.18E-06	-	6.60E+01	47.7506	39
THC_THT_c8990	PREDICTED: uncharacterized protein LOC103956333	174	XP_009366572	7.33E-04	-	6.70E+01	42.3578	34
THC_THT_c9001	gdsl esterase lipase	240	XP_007146093	5.19E-06	-	61	4.66E+01	59
THC_THT_c9005	cytochrome p450-like tbp protein	145	XP_011013113	2.43E-08	-	6.80E+01	55.4546	47
THC_THT_c9020	PREDICTED: uncharacterized protein LOC101513994	156	XP_004492032	2.95E-09	-	8.80E+01	55.4546	34
THC_THT_c9025	nadh dehydrogenase subunit	114	KJB09779	3.48E-11	-	8.30E+01	60.4622	36

THC_THT_c9030	hypothetical protein SORBIDRAFT_1211s0020 20	162	XP_002488956	1.28E-10	-	88	57.7658	35
THC_THT_c9046	hypothetical protein SORBIDRAFT_1211s0020 20	146	XP_002488956	2.79E-10	-	81	56.6102	38
THC_THT_c9054	senescence-associated protein	154	KEH16995	2.46E-09	-	6.40E+01	58.151	51
THC_THT_c9128	retrotransposon ty3-gypsy subclass	173	AAM01168	0.0391782	-	62	36.5798	45
THC_THT_c9151	retrotransposon unclassified	331	BAD35292	7.37E-15	GO:0005739	9.60E+01	73.1738	80
THC_THT_c9155	transposon unclassified	245	CAE04174	3.19E-35	GO:0004523	9.70E+01	134.42	81
THC_THT_c9160	retrotransposon unclassified	220	AAX95721	3.36E-38	GO:0004523	100	1.38E+02	66
THC_THT_c9161	hypothetical protein LOC_Os10g40210	262	AAP54918	9.55E-42	-	9.70E+01	142.124	69
THC_THT_c9165	histone h4	127	ACG32476	3.84E-06	GO:0005488	7.20E+01	45.4394	37
THC_THT_dn_c9166	hypothetical protein	193	AAT76337	2.17E-08	-	75	5.20E+01	40
THC_THT_c9180	retrotransposon ty3-gypsy subclass	182	ABA95343	4.99E-12	GO:0004523	9.40E+01	66.6254	35
THC_THT_c9184	lipid transfer protein	131	AGT16035	5.24E-04	GO:0016020	6.30E+01	40.817	36
THC_THT_c9186	retrotransposon unclassified	161	BAB61230	1.54E-12	GO:0009536	9.70E+01	63.929	34
THC_THT_c9187	retrotransposon unclassified	139	CAE02127	1.35E-23	GO:0004523	9.50E+01	96.2857	46
THC_THT_c9192	histone	201	BAJ87490	8.17E-34	GO:0046982	9.30E+01	120.553	65
THC_THT_c9193	hgwp repeat containing	139	BAD15596	6.76E-17	GO:0046872	8.90E+01	76.6406	46
THC_THT_c9194	ribulose- -bisphosphate carboxylase oxygenase large partial	144	AAL55283	1.80E-18	GO:0016984	1.00E+02	78.5666	37
THC_THT_c9198	hypothetical protein	121	BAD03276	7.80E-04	-	6.50E+01	39.6614	35
THC_THT_c9199	probable nadh dehydrogenase	182	ABB48039	1.03E-29	GO:0006511	9.60E+01	108.612	51
THC_THT_c9201	transposon unclassified	157	AAT85795	3.48E-13	GO:0003677	8.40E+01	68.5514	39

THC_THT_c9209	atpa	122	XP_004253362	4.32E-14	GO:0046961	9.00E+01	70.8626	40
THC_THT_c9214	atp synthase subunit alpha	123	XP_003588326	1.29E-05	-	7.40E+01	46.9802	35
THC_THT_c9217	OJ1005_B10.14	196	BAC05649	6.30E-09	-	8.00E+01	54.6842	35
THC_THT_c9219	inositol transporter 1 isoform 1	129	KEH15665	2.23E-04	-	6.50E+01	43.1282	41
THC_THT_c9224	retrotransposon ty3-gypsy subclass	167	AAL67581	9.36E-30	GO:0090502	1.00E+02	115.931	55
THC_THT_c9229	ulp1 protease-like	129	BAD53421	7.35E-16	GO:0008233	8.70E+01	72.0182	41
THC_THT_c9232	protein tar1-like	225	ACN31703	0.00241622	-	54	38.5058	55
THC_THT_c9237	transposon mutator sub-class	173	ABA98528	1.31E-28	GO:0003676	9.60E+01	114.005	57
THC_THT_c9253	transposon en spm sub-class	319	DAA02095	3.02E-56	GO:0005739	9.70E+01	190.66	88
THC_THT_c9257	lysyl-trna synthetase	126	XP_002952571	1.51E-11	GO:0004824	91	6.35E+01	34
THC_THT_c9262	putative polyprotein	136	AAV43881	4.41E-10	GO:0004523	8.30E+01	60.077	36
THC_THT_c9268	retrotransposon ty1-copia subclass	158	EEE69435	4.66E-29	GO:0050660	1.00E+02	108.612	52
THC_THT_c9272	2-dehydropantoate 2-	201	XP_002536923	4.24E-05	-	61	4.47E+01	47
THC_THT_c9276	protein nrt1 ptr family -like	155	EEE56048	3.90E-16	GO:0006857	1.00E+02	77.411	40
THC_THT_c9279	conserved hypothetical protein	129	XP_002538508	4.07E-06	-	75	4.74E+01	40
THC_THT_c9296	retrotransposon unclassified	292	CAH68539	2.16E-33	GO:0004523	9.50E+01	130.183	69
THC_THT_c9306	retrotransposon ty3-gypsy subclass	298	AAV25052	3.81E-49	GO:0003964	1.00E+02	175.252	80
THC_THT_c9309	integrase core	143	NP_001175500	5.30E-10	GO:0005739	80	5.62E+01	36
THC_THT_c9312	calcineurin b-like protein	142	AAM01008	2.44E-13	GO:0090502	9.40E+01	65.0846	34
THC_THT_c9313	probable alanine--trna chloroplastic isoform x2	133	XP_008448324	0.276915	-	60	34.2686	50
THC_THT_c9325	transposon unclassified	265	ABA98352	5.30E-42	GO:0004523	8.30E+01	150.599	86
THC_THT_c9327	hypothetical protein L484_018827	156	EXC01915	0.0216293	-	52	35.8094	50
THC_THT_c9329	retrotransposon unclassified	167	BAC16417	0.00634942	-	50	38.891	52

THC_THT_c9330	atpase subunit 4	208	YP_002000550	1.05E-39	GO:0015078	98	1.37E+02	69
THC_THT_c9331	catalase peroxidase	108	KIY98092	0.494598	-	52	32.7278	36
THC_THT_c9332	cyclophilin 1	128	BAM13283	7.67E-21	GO:0006457	1.00E+02	86.2705	42
THC_THT_c9336	retrotransposon ty1-copia subclass	221	AAT93883	2.30E-40	GO:0017148	1.00E+02	148.288	70
THC_THT_c9345	atp binding cassette	192	XP_002537480	5.66E-10	GO:0009536	62	5.85E+01	58
THC_THT_c9346	carotenoid cleavage dioxygenase 8 homolog chloroplastic-like	136	EAZ13518	1.94E-12	GO:1901601	8.90E+01	66.6254	39
THC_THT_c9348	calcineurin b-like protein	216	ABA97118	8.19E-19	GO:0009536	8.40E+01	63.929	39
THC_THT_c9355	altered xyloglucan 8	110	AID60138	0.121106	-	62	35.039	35
THC_THT_c9363	predicted protein	154	XP_001758721	0.622059	-	55	32.7278	34
THC_THT_c9379	hypothetical protein ZEAMMB73_316016	158	AFW83476	1.74E-14	-	9.70E+01	67.3958	35
THC_THT_c9407	hypothetical protein OsI_34304	138	EEC67307	1.68E-19	GO:0016740	8.60E+01	82.8037	46
THC_THT_c9418	jasmonate-induced protein	158	ABA96464	3.30E-21	GO:0030246	1.00E+02	87.0409	39
THC_THT_c9420	unclassified retrotransposon protein	233	BAH79998	1.28E-12	-	88	6.35E+01	34
THC_THT_c9422	plant disease resistance poly	119	BAD25363	0.81072	-	86	31.9574	38
THC_THT_c9428	cytochrome p450 734a6-like isoform x2	271	AGC78957	1.02E-12	GO:0008137	7.70E+01	65.4698	45
THC_THT_c9440	retrotransposon protein	199	CAH65903	1.01E-20	GO:0003676	1.00E+02	88.5817	41
THC_THT_c9442	transposon unclassified	156	ABA97299	2.85E-15	GO:0046872	8.70E+01	75.485	40
THC_THT_c9449	60s ribosomal protein l9	143	XP_002453127	3.78E-24	GO:0005840	100	9.40E+01	47
THC_THT_c9450	wall-associated receptor kinase 3	154	NP_001176887	2.51E-27	GO:0006468	98	1.02E+02	51
THC_THT_c9461	phenylalanine ammonia-lyase-like protein	222	AAL58160	1.45E-30	GO:0016023	8.10E+01	117.857	72
THC_THT_c9465	retrotransposon ty3-gypsy subclass	167	AAM01095	3.59E-08	GO:0003964	1.00E+02	48.1358	43

THC_THT_c9466	general transcription factor 3c polypeptide 2	131	EEC77880	1.69E-21	GO:0003677	1.00E+02	86.6557	43
THC_THT_c9468	Os01g0268000	120	NP_001042689	1.14E-11	GO:0005739	82	6.08E+01	39
THC_THT_c9493	tubulin alpha chain-like	132	NP_001275472	3.12E-26	GO:0000226	97	1.02E+02	43
THC_THT_c9494	hypothetical protein	168	BAD08780	1.29E-12	-	9.40E+01	66.2402	35
THC_THT_c9499	dihydroxy-acid dehydratase	108	KFM27114	4.48E-08	GO:0005507	8.20E+01	50.8322	35
THC_THT_c9518	hypothetical protein	241	BAI39772	3.42E-14	GO:0008270	94	6.78E+01	35
THC_THT_c9529	atpase plasma membrane-type-like	124	CAD29313	1.80E-08	GO:0008553	1.00E+02	55.0694	38
THC_THT_c9534	omega-6 fatty acid endoplasmic reticulum isozyme 2	173	NP_001047927	3.10E-25	GO:0005789	100	101.293	46
THC_THT_c9537	abc transporter i family member chloroplastic-like	113	XP_001416756	6.28E-08	GO:0044272	77	5.12E+01	36
THC_THT_c9550	f-box domain containing protein	162	AAO73209	2.07E-16	-	94	76.6406	3.90E+01
THC_THT_c9577	60s ribosomal protein l23	109	AFW89630	5.06E-16	GO:0005840	9.70E+01	72.0182	35
THC_THT_c9581	transposon en spm sub-class	111	BAD81352	6.52E-13	GO:0003676	91	6.78E+01	35
THC_THT_c9585	hypothetical protein POPTR_0016s04430g	134	XP_002323276	0.387238	-	57	33.4982	38
THC_THT_c9594	cell wall	167	NP_001059418	1.56E-25	GO:0003676	96	1.01E+02	55
THC_THT_c9599	atp synthase subunit alpha	195	CDX71650	1.56E-06	-	5.60E+01	48.521	62
THC_THT_c9616	dihydrolipoyl dehydrogenase	166	EEC76122	1.39E-07	GO:0044763	7.60E+01	53.5286	38
THC_THT_c9647	retrotransposon ty3-gypsy subclass	112	AAV24823	3.48E-16	GO:0003964	1.00E+02	77.411	37
THC_THT_c9648	hypothetical protein OsJ_24971	145	EEE67517	6.28E-12	-	9.10E+01	64.6994	35
THC_THT_c9660	cell wall-associated hydrolase	147	EXC50692	0.00506716	-	57	38.1206	45
THC_THT_c9673	flagellar basal-body rod protein	109	XP_002537068	1.77E-11	GO:0031514	85	5.97E+01	35
THC_THT_c9675	subtilase-like protein	136	BAD82244	1.25E-12	GO:0009536	84	6.62E+01	38

THC_THT_c9681	protein	155	XP_001760735	1.05E-17	GO:0005524	92	8.16E+01	51
THC_THT_c9716	hypothetical protein	107	BAD09240	1.17E-14	GO:0005739	100	6.97E+01	35
THC_THT_c9721	predicted protein	104	EDQ48366	1.22E-08	GO:0003824	8.50E+01	54.6842	34
THC_THT_c9741	cytokinin-o-glucosyltransferase 2	133	EEE68654	6.19E-21	GO:0016758	9.70E+01	88.1965	44
THC_THT_c9750	epstein-barr virus ebna-1-like protein	216	AAM93728	9.31E-16	GO:0005739	7.20E+01	61.6178	48
THC_THT_c9774	ulp1 protease-like protein	170	ABA99571	1.47E-22	GO:0005739	8.20E+01	90.8929	56
THC_THT_c9779	photosystem ii d2 protein	196	AJM90160	1.38E-27	GO:0046872	9.60E+01	106.301	66
THC_THT_c9794	wall-associated receptor kinase-like 16	133	XP_010277630	0.986558	-	48	32.3426	37
THC_THT_c9801	transposon mutator sub-class	109	AAV25047	4.31E-04	GO:0008270	9.40E+01	42.3578	36
THC_THT_c9809	hypothetical protein OsJ_36528	209	EEE53437	1.78E-21	GO:0043531	1.00E+02	92.4337	40
THC_THT_c9825	alcohol dehydrogenase class iii	126	XP_005648729	1.51E-15	GO:0005737	88	7.43E+01	42
THC_THT_c9828	retrotransposon ty3-gypsy subclass	226	CAD40940	4.04E-29	GO:0003676	91	9.32E+01	46
THC_THT_c9848	60s ribosomal protein l7a	148	NP_001061550	1.48E-24	GO:0005840	100	97.8265	47
THC_THT_c9872	epstein-barr virus ebna-1-like protein	236	AAT58886	1.90E-21	GO:0016021	8.00E+01	94.3597	62
THC_THT_c9882	hypothetical protein OsI_20540	121	EEC79491	2.08E-07	GO:0050660	7.40E+01	51.6026	35
THC_THT_c9886	transposable element retrotrans_gag	121	ABA97569	1.25E-18	GO:0008270	9.70E+01	80.4925	39
THC_THT_c9889	alpha tubulin	163	AAD11425	9.31E-26	GO:0005874	1.00E+02	102.449	43
THC_THT_c9914	hypothetical protein	154	AAU43943	1.06E-05	-	6.40E+01	45.8246	50
THC_THT_c9933	hypothetical protein COCSUDRAFT_21900	129	XP_005651344	0.697927	-	59	32.3426	37
THC_THT_c9938	retrotransposon ty3-gypsy subclass	212	ABB47441	5.24E-19	GO:0003964	9.30E+01	83.9593	43

THC_THT_c9955	dihydrolipoamide acetyltransferase component of pyruvate	119	XP_002538756	1.53E-09	GO:0008152	85	5.51E+01	34
THC_THT_c9963	nadh-ubiquinone oxidoreductase chain	195	XP_003588331	7.26E-16	-	8.10E+01	77.0258	49
THC_THT_c9972	bifunctional monodehydroascorbate reductase and carbonic anhydrase nectarin-3-like	126	XP_002539756	3.87E-15	GO:0046872	85	7.12E+01	42
THC_THT_c9977	pentatricopeptide repeat-containing protein mitochondrial-like	102	EAY78393	1.97E-17	GO:0005739	1.00E+02	79.7221	34
THC_THT_c9995	eukaryotic translation initiation factor 3 subunit l-like	113	EEC78744	4.37E-19	GO:0005852	1.00E+02	84.7297	37
THC_THT_c10001	pentatricopeptide repeat-containing protein chloroplastic-like	107	NP_001049766	8.42E-14	GO:0009507	100	70.0922	35
THC_THT_c10019	hypothetical protein B456_010G057000	147	KJB64600	0.777817	-	54	32.7278	37
THC_THT_c10020	hypothetical protein TRIUR3_09014	165	EMS53098	1.91E-17	-	9.80E+01	78.9518	54
THC_THT_c10028	dipeptidyl peptidase	116	XP_002539019	1.06E-04	-	71	4.35E+01	38
THC_THT_c10041	probable beta-glucosidase partial	129	XP_011016741	3.47E-11	-	8.40E+01	63.1586	39
THC_THT_c10045	retrotransposon unclassified	107	CAE02878	8.34E-18	GO:0004523	1.00E+02	82.0333	35
THC_THT_c10059	retrotransposon ty3-gypsy subclass	241	CAH66816	2.02E-36	GO:0003964	9.80E+01	134.806	62
THC_THT_c10064	o-methyltransferase expressed	135	ABA97755	6.27E-24	GO:0008171	1.00E+02	95.9005	45
THC_THT_c10068	magnesium-chelatase subunit chloroplastic isoform x2	130	XP_002956152	0.0132233	-	73	38.1206	34
THC_THT_c10070	n-acetyl-gamma-glutamyl-phosphate reductase	117	AAL24190	4.52E-13	GO:0051287	86	6.74E+01	38

THC_THT_c10090	6-phosphogluconate decarboxylating	107	AHM10416	1.37E-17	GO:0009750	1.00E+02	80.1073	34
THC_THT_c10093	Gluconolactonase precursor, putative	119	XP_002536324	3.83E-04	GO:0016787	72	4.16E+01	36
THC_THT_c10101	hypothetical protein OsI_34215	205	EAY79110	1.44E-09	-	7.80E+01	58.151	38
THC_THT_c10114	ectonucleotide pyrophosphatase	113	XP_002538176	3.68E-13	GO:0003824	97	6.74E+01	36
THC_THT_c10116	60s ribosomal protein l26-1	149	NP_001066165	1.06E-19	GO:0003735	100	83.1889	40
THC_THT_c10120	retrotransposon unclassified	150	CAE02992	3.06E-26	GO:0009536	9.70E+01	106.301	48
THC_THT_c10122	Os03g0349700	136	NP_001050109	8.71E-12	GO:0005739	83	61.2326	4.30E+01
THC_THT_c10141	nadh dehydrogenase	136	XP_005645041	7.68E-08	GO:0071704	69	5.24E+01	43
THC_THT_c10148	dead-box atp-dependent rna helicase 5	182	CAA09197	5.25E-14	GO:0001510	7.00E+01	70.8626	60
THC_THT_c10176	ac092553_17 pol polyprotein	127	EAZ30376	1.91E-20	GO:0003676	1.00E+02	85.5001	42
THC_THT_c10181	cytochrome b6	134	AID16294	2.48E-24	GO:0016491	1.00E+02	93.9745	44
THC_THT_c10200	predicted protein	108	XP_001701551	6.94E-12	GO:0050660	94	6.24E+01	36
THC_THT_c10237	phenylalanyl-trna synthetase beta	116	XP_002534883	9.46E-09	GO:0006432	84	5.51E+01	38
THC_THT_c10241	nadp-dependent oxidoreductase p2	107	BAD35462	7.44E-18	GO:0008270	100	79.7221	3.50E+01
THC_THT_c10242	photosystem i p700 apoprotein a1	140	AIA24221	1.51E-18	GO:0016491	8.20E+01	84.3445	46
THC_THT_c10249	calmodulin-binding transcription activator	125	XP_002531268	0.75591	-	54	32.7278	46
THC_THT_c10256	dcp1-like decapping family expressed	112	NP_001066198	1.50E-11	-	91	62.7734	34
THC_THT_c10272	atp binding cassette	154	XP_002536193	1.43E-26	GO:0016020	98	1.03E+02	51
THC_THT_c10277	hypothetical protein MTR_8g040260	160	XP_003627937	0.520005	-	64	33.113	34
THC_THT_c10279	ycf68 protein	219	EPS74511	1.02E-08	-	6.10E+01	54.299	52
THC_THT_c10283	centromere-specific	192	AAT07556	3.76E-30	GO:0003964	9.60E+01	115.546	60

THC_THT_c10290	UDP-n-acetylmuramate--l-alanine ligase, putative	142	XP_002538076	6.25E-07	-	65	4.81E+01	43
THC_THT_c10300	guanylate kinase	151	XP_005844874	8.09E-06	GO:0006796	64	4.70E+01	48
THC_THT_c10309	retrotransposon ty3-gypsy subclass	202	AAX95754	1.69E-38	GO:0004523	1.00E+02	137.117	67
THC_THT_c10315	26s proteasome non-atpase regulatory subunit 9-like	102	NP_001063197	3.74E-14	GO:0051510	100	68.1662	3.40E+01
THC_THT_c10316	dna repair protein	127	KDD76638	2.22E-10	GO:0008094	8.00E+01	59.6918	42
THC_THT_c10321	conserved hypothetical protein	186	XP_002536928	0.0474266	-	73	36.5798	34
THC_THT_c10322	transposon en spm sub-class	201	CAE02715	5.10E-34	GO:0006508	9.70E+01	128.642	67
THC_THT_c10336	udp-arabinopyranose mutase 1-like	171	EEC73964	7.22E-26	GO:0005829	1.00E+02	97.4413	46
THC_THT_c10339	homeobox-leucine zipper protein roc2-like isoform x2	113	XP_008784553	9.09E-14	-	9.70E+01	70.0922	34
THC_THT_c10350	xyloglucan endotransglycosylase hydrolase protein 8-like	134	EAZ06114	2.48E-22	GO:0004553	1.00E+02	92.0485	44
THC_THT_c10354	hypothetical protein	147	BAD36099	1.73E-11	GO:0006952	9.10E+01	59.6918	34
THC_THT_c10390	orf300 gene product	215	CAN64512	6.23E-34	GO:0016021	9.20E+01	124.405	69
THC_THT_c10396	wrky transcription factor	112	DAA05137	1.21E-17	GO:0006355	100	7.82E+01	37
THC_THT_c10402	leucine rich protein	199	ABH09321	9.80E-09	GO:0016787	7.50E+01	54.299	40
THC_THT_c10403	salicylate carboxymethyltransferase-like	131	EAY75511	7.90E-22	GO:0008757	100	90.8929	43
THC_THT_c10406	hypothetical protein AMTR_s00042p00225140	207	ERN03623	3.08E-09	-	7.30E+01	55.4546	46
THC_THT_c10420	retrotransposon ty3-gypsy subclass	197	BAH80069	3.47E-31	GO:0003964	9.60E+01	122.094	61
THC_THT_c10426	transposon en spm sub-class	125	ABA98431	9.80E-17	GO:0005739	1.00E+02	78.9518	36

THC_THT_c10429	retrotransposon ty3-gypsy subclass	133	BAH80065	2.33E-24	GO:0003964	1.00E+02	100.908	43
THC_THT_c10442	calcium-transporting atpase plasma membrane-type	133	EMT12463	3.86E-20	GO:0005388	1.00E+02	88.9669	44
THC_THT_c10449	polyribonucleotide nucleotidyltransferase	120	XP_010420100	6.47E-06	GO:0090305	7.70E+01	47.7506	40
THC_THT_c10459	transposon en spm sub-class	164	CAD40439	1.32E-26	GO:0008234	9.00E+01	105.145	54
THC_THT_c10475	hgwp repeat containing	102	BAC83459	2.97E-04	-	45	4.16E+01	55
THC_THT_c10497	40s ribosomal protein s3	132	ACN27873	3.40E-22	GO:0003735	100	8.90E+01	43
THC_THT_c10500	xylanase inhibitor protein 1-like	144	EEC83894	1.43E-21	GO:0004568	100	8.82E+01	40
THC_THT_c10522	abc transporter b family member 19	104	AET03262	0.0276917	-	70	36.5798	34
THC_THT_c10534	transposon mutator sub-class	176	CAE02587	1.18E-30	GO:0016020	1.00E+02	120.168	58
THC_THT_c10560	hypothetical protein AMTR_s00058p00215090	140	ERN06704	0.898736	-	52	30.8018	46
THC_THT_c10624	embryo defective 1144	104	KIZ07769	1.43E-04	GO:0016829	7.30E+01	43.1282	34
THC_THT_c10654	two-component sensor histidine kinase	153	XP_002538577	2.42E-05	GO:0004673	73	4.58E+01	42
THC_THT_c10677	ORF124	157	YP_001152214	0.0299463	-	61	35.4242	36
THC_THT_c10689	retrotransposon ty3-gypsy subclass	171	AAM19013	8.62E-19	GO:0090502	9.70E+01	85.8853	43
THC_THT_c10691	ubiquitin-like partial	122	CDY70361	5.31E-21	GO:0006623	1.00E+02	85.1149	40
THC_THT_c10694	transposon mutator sub-class	150	NP_001045854	6.28E-10	GO:0008270	100	5.97E+01	42
THC_THT_c10722	phosphatidylinositol 4-phosphate 5-kinase 1-like	131	NP_001051025	1.23E-20	GO:0046854	100	90.1225	43
THC_THT_c10730	pentatricopeptide repeat-containing protein at1g02420	141	EAY88983	3.46E-21	GO:0005634	1.00E+02	90.8929	46

THC_THT_c10786	hypothetical protein VOLCADRAFT_101541	141	XP_002960029	0.00568399	-	65	38.891	40
THC_THT_c10788	receptor-like protein kinase-like	126	EEC66584	7.94E-20	GO:0004713	95	8.74E+01	40
THC_THT_c10816	hypothetical protein	149	BAD33949	1.32E-06	-	8.20E+01	47.3654	34
THC_THT_c10833	retrotransposon ty3-gypsy subclass	149	CAD40114	5.53E-15	GO:0044260	7.50E+01	74.7146	49
THC_THT_c10842	hypothetical protein	107	BAD10558	2.13E-04	-	9.70E+01	42.743	34
THC_THT_c10846	leucine--trna mitochondrial	118	XP_011014373	3.62E-11	GO:0048731	7.60E+01	63.1586	39
THC_THT_c10859	retrotransposon ty3-gypsy subclass	141	ABB47092	4.52E-18	GO:0004523	1.00E+02	83.1889	36
THC_THT_c10867	gag-pol polyprotein	131	AAQ56338	1.23E-19	GO:0003964	1.00E+02	87.8113	43
THC_THT_c10874	chlorophyllide a oxygenase	120	XP_002536894	8.71E-04	GO:0005488	65	4.08E+01	38
THC_THT_c10884	retrotransposon ty3-gypsy subclass	266	CAE03840	5.44E-29	GO:0003723	9.60E+01	116.316	55
THC_THT_c10944	PREDICTED: uncharacterized protein LOC104740870	139	XP_010459896	0.3484	-	52	33.8834	42
THC_THT_c10945	dead-box atp-dependent rna helicase chloroplastic-like	109	XP_002535074	0.0185299	-	65	36.965	35
THC_THT_c10982	beta subunit of rna polymerase	110	AGG09511	3.26E-13	GO:0003899	9.10E+01	65.4698	36
THC_THT_c10991	protein kinase domain containing protein	102	NP_001066817	0.0897561	-	64	35.039	34
THC_THT_c10996	Os02g0253000	125	NP_001046453	2.80E-07	-	73	50.8322	3.80E+01
THC_THT_c11006	phosphatidate phosphatase pah1	103	NP_001055766	2.84E-16	GO:0016311	100	77.411	34
THC_THT_c11036	elongation factor 1-alpha	108	CBI31495	5.54E-15	GO:0003746	9.70E+01	68.9366	34
THC_THT_c11040	transposable element transposase_28	116	CAD39995	3.13E-16	GO:0004523	9.40E+01	76.6406	38
THC_THT_c11049	c2 domain-containing family protein	132	EAZ27970	2.17E-21	GO:0016757	1.00E+02	92.4337	43
THC_THT_c11082	hypothetical protein OsI_05351	140	EEC72236	2.56E-09	-	1.00E+02	54.299	39

THC_THT_c11098	bifunctional udp-n-acetylglucosamine pyrophosphorylase and glucosamine-1-phosphate n-	150	XP_002535682	5.86E-15	GO:0019134	83	7.12E+01	48
THC_THT_c11101	fasciclin-like arabinogalactan protein 4-like	111	EEC71769	2.28E-16	GO:0009825	1.00E+02	76.6406	37
THC_THT_c11120	hypothetical protein MTR_0002s0260	126	KEH17696	7.71E-22	-	1.00E+02	86.2705	39
THC_THT_c11137	cytochrome c biogenesis fc	167	AEK66734	2.14E-11	GO:0016020	69	6.35E+01	43
THC_THT_c11157	transposon mutator sub-class	147	CAE01499	1.42E-26	GO:0051287	9.50E+01	107.071	48
THC_THT_c11158	protein lurp-one-related 2-like	199	XP_010244534	0.357018	-	55	33.8834	34
THC_THT_c11162	unnamed protein product	146	CDP07597	0.215518	-	55	34.2686	43
THC_THT_c11169	cytosolic phosphoglucose isomerase	142	EEE52320	4.34E-17	GO:0006094	8.70E+01	80.8777	47
THC_THT_c11174	hypothetical protein OsI_26123	109	EEC82107	3.46E-05	GO:0016023	1.00E+02	45.0542	35
THC_THT_c11180	conserved hypothetical protein	115	XP_002535185	4.99E-05	-	97	4.24E+01	38
THC_THT_c11187	beta subunit of rna polymerase	145	YP_001019104	6.36E-05	GO:0016779	77	4.51E+01	35
THC_THT_c11190	hypothetical protein LOC_Os12g16170	122	ABA97353	1.51E-05	-	7.00E+01	43.8986	34
THC_THT_c11195	cinnamyl alcohol dehydrogenase	111	XP_008460922	1.71E-13	GO:0008270	8.60E+01	68.1662	37
THC_THT_c11206	transposon unclassified	133	XP_008780563	9.68E-18	GO:0004523	1.00E+02	78.5666	38
THC_THT_c11209	low quality protein: protein time for coffee-like	104	NP_001060049	1.84E-12	-	100	6.66E+01	34
THC_THT_c11254	isovaleryl-dehydrogenase	125	KEH15547	1.54E-07	GO:0003995	7.70E+01	52.373	40
THC_THT_c11269	hypothetical protein CHLNCDRAFT_28889	149	XP_005842766	0.0179998	-	61	35.039	44

THC_THT_c11285	40s ribosomal protein s17-4	121	EEC74366	1.45E-18	GO:0003735	9.70E+01	80.4925	39
THC_THT_c11290	hypothetical protein OsI_25102	128	EAZ02962	2.86E-05	-	6.80E+01	43.1282	41
THC_THT_c11304	#NAME?	120	XP_005647421	0.0484846	-	52	35.8094	40
THC_THT_c11316	abc transporter i family member 17	136	XP_002535216	3.55E-05	GO:0016787	65	4.39E+01	46
THC_THT_c11331	uncharacterized protein LOC103636081 isoform X1	135	XP_008656661	0.63545	-	60	33.113	35
THC_THT_c11332	photosystem i p700 apoprotein a1	121	AFR13040	1.44E-18	GO:0016491	9.70E+01	80.8777	40
THC_THT_c11351	unnamed protein product	216	CDP21352	1.69E-05	-	5.50E+01	46.2098	72
THC_THT_c11353	root phototropism protein 2-like	198	EEC68739	1.74E-27	GO:0009954	9.50E+01	109.768	62
THC_THT_c11362	AC120497_4Hypothetical protein	119	AAM74404	0.289844	-	58	33.113	39
THC_THT_c11365	retrotransposon unclassified	126	ABA97820	3.14E-12	GO:0004523	8.30E+01	66.2402	37
THC_THT_c11390	protein	114	XP_003062214	8.22E-09	GO:0005739	80	5.62E+01	35
THC_THT_c11400	pseudo response regulator	119	XP_002985946	0.719384	-	61	31.9574	36
THC_THT_c11406	transposon en spm sub-class	148	AAM18163	2.22E-18	GO:0005739	95	8.32E+01	42
THC_THT_c11419	Mitochondrial protein, putative	230	XP_003588355	0.102331	-	61	36.1946	36
THC_THT_c11470	hypothetical protein B456_012G045700	122	KJB75530	0.546139	-	51	33.113	39
THC_THT_c11485	hypothetical protein EUTSA_v10028274mg	154	XP_006405909	0.228154	-	56	33.113	48
THC_THT_c11504	transposon mutator sub-class	155	NP_001172742	1.74E-23	GO:0004553	97	9.94E+01	49
THC_THT_c11510	chloroplast hypothetical protein	265	YP_588293	8.23E-26	GO:0005739	100	9.98E+01	47
THC_THT_c11539	myosin heavy chain kinase b-like	105	EEE55996	1.08E-05	GO:0009536	1.00E+02	45.8246	35
THC_THT_c11550	heat shock cognate 70 kda protein	170	CDY02235	4.61E-09	-	5.80E+01	54.6842	60

THC_THT_c11557	hypothetical protein OsJ_30246	141	EEE70170	3.84E-15	GO:0006468	9.20E+01	74.7146	39
THC_THT_c11563	reticulon-like protein b2	156	EAY75742	3.76E-05	GO:0016021	1.00E+02	45.0542	48
THC_THT_c11580	potassium ion uptake transporter	131	XP_002534890	1.36E-07	GO:0015079	79	5.20E+01	43
THC_THT_c11585	nad -binding partial	113	XP_002953969	1.01E-04	GO:0008152	75	4.31E+01	37
THC_THT_c11621	nadh dehydrogenase subunit 9	112	AAW30332	4.14E-17	GO:0005739	9.70E+01	76.2554	36
THC_THT_c11629	photosystem ii d2 protein	126	XP_004489529	7.61E-20	GO:0009772	1.00E+02	82.4185	41
THC_THT_c11637	two-component system sensor histidine kinase response	184	XP_002534677	1.40E-04	-	55	4.20E+01	49
THC_THT_c11652	transposon mutator sub-class	186	ABA99387	2.37E-15	GO:0003676	9.80E+01	76.2554	60
THC_THT_c11660	cell wall-associated partial	188	EXC01912	3.14E-15	-	7.20E+01	70.0922	50
THC_THT_c11667	PREDICTED: 5-oxoprolinase	129	XP_009361409	0.209369	-	60	34.6538	35
THC_THT_c11668	hypothetical protein	135	BAD23443	1.43E-23	-	1.00E+02	90.8929	45
THC_THT_c11676	60s ribosomal	126	BAD21594	6.42E-18	GO:0005840	100	8.05E+01	39
THC_THT_c11687	predicted protein	158	XP_001764461	0.691964	-	52	33.113	40
THC_THT_c11704	predicted protein	137	XP_001753456	0.361205	-	57	31.9574	40
THC_THT_c11711	acetyl- cytosolic	123	XP_002959312	1.99E-05	GO:0016740	68	4.47E+01	41
THC_THT_c11720	hypothetical protein MTR_3g064150	102	XP_003600604	0.269367	-	58	31.187	34
THC_THT_c11722	conserved hypothetical protein	115	XP_002536320	1.48E-10	-	82	5.97E+01	35
THC_THT_c11728	retrotransposon ty3-gypsy subclass	250	AAX96872	7.31E-31	GO:0004523	97	8.70E+01	40
THC_THT_c11731	putative polyprotein	137	AAW56892	4.22E-12	-	9.10E+01	63.929	34
THC_THT_c11732	putative polyprotein	168	AAT47106	5.10E-17	GO:0004523	9.10E+01	62.7734	34
THC_THT_c11737	enoyl- hydratase isomerase	119	KDP26858	0.0569091	-	63	35.4242	38
THC_THT_c11764	erd1 family protein	128	XP_006653364	1.61E-09	GO:0009941	7.90E+01	58.151	39
THC_THT_c11772	3-ketoacyl- thiolase	223	KEH15548	9.94E-07	GO:0003824	6.10E+01	39.6614	52
THC_THT_c11777	cell wall	158	AAP53837	2.92E-27	GO:0003676	9.80E+01	103.99	52

THC_THT_c11825	primary amine oxidase-like	137	NP_001060056	7.60E-27	GO:0008131	100	105.531	4.50E+01
THC_THT_c11829	histidine kinase 3	101	KCW71221	0.0897576	-	59	35.039	37
THC_THT_c11831	anthocyanidin 3-o-glucosyltransferase-like	105	NP_001067852	5.01E-18	GO:0008152	100	81.2629	3.50E+01
THC_THT_c11835	cytochrome p450 716b1-like	113	EAZ08917	1.39E-17	GO:0020037	1.00E+02	79.7221	37
THC_THT_c11852	pentatricopeptide repeat-containing protein chloroplastic-like	114	EEC76566	1.94E-17	GO:0055114	1.00E+02	80.4925	37
THC_THT_c11856	P0696G06.5	189	BAC06248	4.04E-08	-	6.50E+01	52.7582	47
THC_THT_c11861	conserved hypothetical protein	128	XP_002537069	2.56E-11	-	76	6.28E+01	42
THC_THT_c11880	bifunctional 3-dehydroquinate	102	ERN16858	0.0195821	-	67	36.965	34
THC_THT_c11885	retrotransposon unclassified	143	CAE03137	4.16E-25	GO:0005739	100	1.03E+02	47
THC_THT_c11912	retrotransposon ty3-gypsy subclass	156	AAK13116	4.01E-26	GO:0003964	9.50E+01	107.071	48
THC_THT_c11918	retrotransposon ty3-gypsy subclass	118	CAD40482	6.56E-20	GO:0004523	100	87.0409	3.90E+01
THC_THT_c11931	retrotransposon unclassified	152	AAK52152	4.08E-24	GO:0004523	9.60E+01	100.908	50
THC_THT_c11956	hypothetical protein	193	BAD62185	2.77E-16	GO:0005739	72	7.32E+01	58
THC_THT_c11965	peptide transporter ptr3-a-like	121	EMS64614	2.08E-09	GO:0016020	9.00E+01	57.7658	40
THC_THT_c11968	predicted protein	184	EDQ48453	3.41E-05	-	6.40E+01	45.8246	45
THC_THT_c11973	retrotransposon unclassified	174	ABA98822	2.09E-07	-	6.40E+01	53.1434	53
THC_THT_c11984	urease isoform x2	139	XP_002969412	1.93E-11	GO:0046872	76	6.39E+01	47
THC_THT_c11985	ribosomal protein l5	112	YP_002600845	1.28E-12	GO:0006412	97	6.39E+01	35
THC_THT_c12002	hypothetical protein	124	BAD35764	9.49E-04	-	62	3.89E+01	40
THC_THT_c12021	hypothetical protein SORBIDRAFT_05g016450	146	XP_002449484	9.50E-11	-	82	5.74E+01	35
THC_THT_c12024	Os08g0482500	131	NP_001062069	3.09E-23	-	100	90.8929	43

THC_THT_c12033	glyceraldehyde-3-phosphate dehydrogenase	104	EMT10979	2.38E-14	GO:0005737	1.00E+02	68.9366	34
THC_THT_c12041	b-class lysophosphatidate acyltransferase	121	EPS68411	0.00408488	-	64	38.891	34
THC_THT_c12059	hypothetical protein	151	BAD10744	0.00369177	-	62	37.3502	35
THC_THT_c12066	peroxidase 45-like	103	BAD53899	3.09E-16	GO:0046872	97	74.7146	34
THC_THT_c12069	snf1-related protein kinase catalytic subunit alpha kin10-like	115	NP_001059110	4.84E-16	GO:0004713	100	76.2554	34
THC_THT_c12072	hypothetical protein	161	BAD87492	0.195945	-	54	35.039	48
THC_THT_c12076	prolyl endopeptidase-like	133	XP_002949021	3.10E-08	GO:0008236	75	5.47E+01	41
THC_THT_c12098	delta-aminolevulinic acid dehydratase	104	CAC36225	7.43E-08	GO:0004655	8.50E+01	50.8322	34
THC_THT_c12127	btb poz and math domain-containing protein 2-like	112	EAZ05474	6.24E-18	-	1.00E+02	79.7221	37
THC_THT_c12132	oligopeptide abc	110	EAY93131	0.90943	-	52	31.9574	40
THC_THT_c12133	AC091735_21Hypothetical protein	107	AAM01048	0.10232	-	62	34.2686	35
THC_THT_c12169	Os09g0473900	202	NP_001063453	7.94E-14	-	90	7.16E+01	40
THC_THT_c12183	retrotransposon ty3-gypsy subclass	117	ABA95101	1.52E-18	GO:0003676	1.00E+02	84.3445	35
THC_THT_c12196	unnamed protein product	136	CCO65945	0.472091	-	56	33.4982	37
THC_THT_c12201	hypothetical protein	119	BAC57276	3.30E-17	GO:0016779	9.20E+01	78.9518	39
THC_THT_c12248	d-mannose binding lectin family expressed	102	EEC66627	1.77E-16	GO:0006468	1.00E+02	77.7962	34
THC_THT_c12267	transposon en spm sub-class	164	CAE02501	3.71E-25	GO:0005739	1.00E+02	103.99	46
THC_THT_c12286	udp-glucose 6- expressed	130	NP_001051328	1.40E-22	GO:0003979	100	94.3597	41
THC_THT_c12304	retrotransposon gag protein	124	AAO66560	2.29E-11	GO:0008270	97	6.32E+01	36
THC_THT_c12316	cytochrome c biogenesis	136	AGC78966	1.69E-08	GO:0005739	95	5.35E+01	40

THC_THT_c12342	hth-type transcriptional regulator	163	XP_002535633	2.90E-06	-	67	4.85E+01	49
THC_THT_c12345	flagellar motor switch	190	XP_002535051	6.54E-18	GO:0016020	95	7.90E+01	44
THC_THT_c12354	probable receptor-like protein kinase at1g33260-like	152	NP_001045631	4.89E-15	GO:0016023	100	73.559	36
THC_THT_c12369	putative polyprotein	105	AAS98452	3.09E-15	GO:0003676	9.70E+01	74.3294	34
THC_THT_c12404	pdx1_stelp ame: full=probable pyridoxal biosynthesis protein pdx1 ame: full=h47	138	CAA50602	2.12E-09	GO:0042823	8.00E+01	55.8398	35
THC_THT_c12413	glycerol-3-phosphate dehydrogenase mitochondrial	118	XP_010943403	0.045498	-	81	36.1946	37
THC_THT_c12441	hypothetical protein LOC_Os03g13530	119	ABF94867	2.38E-13	-	8.40E+01	65.0846	39
THC_THT_c12458	spore coat proein-like protein	167	AAM19053	8.79E-17	GO:0009536	8.80E+01	77.7962	44
THC_THT_c12488	phosphoribosylformylglycinamidine cyclo-ligase	112	XP_002865247	1.53E-07	GO:0005507	77	5.04E+01	35
THC_THT_c12497	chorismate synthase	122	AES62175	0.713647	-	60	32.7278	35
THC_THT_c12505	retrotransposon ty3-gypsy subclass	114	ABA99150	3.90E-18	GO:0003964	1.00E+02	82.8037	37
THC_THT_c12523	glyceraldehyde-3-phosphate dehydrogenase	110	XP_006659081	8.62E-16	GO:0005737	9.70E+01	73.9442	35
THC_THT_c12551	hgwp repeat containing	130	BAD46725	4.82E-18	GO:0016023	9.30E+01	82.4185	43
THC_THT_c12554	probable carboxylesterase 17-like	118	EAY79872	1.56E-17	GO:0008152	1.00E+02	79.337	38
THC_THT_c12558	hypothetical protein	112	BAD23608	0.0237323	-	61	34.6538	42
THC_THT_c12563	g-type lectin s-receptor-like serine threonine-protein kinase at2g19130-like	118	EAZ14437	6.01E-21	GO:0006468	1.00E+02	90.8929	39

THC_THT_c12566	abc transporter i family member 1-like	129	EEC68421	9.86E-15	GO:0016021	9.00E+01	72.4034	43
THC_THT_c12571	chaperone protein chloroplastic	112	XP_004489445	3.57E-05	GO:0005737	8.10E+01	45.4394	37
THC_THT_c12589	pleiotropic drug resistance protein 6-like	101	XP_003569645	3.39E-07	GO:0044699	7.00E+01	51.2174	34
THC_THT_c12598	retrotransposon ty1-copia subclass	118	ABA97293	8.14E-18	GO:0004553	9.40E+01	78.9518	39
THC_THT_c12612	u-box domain containing expressed	110	NP_001050813	1.44E-06	GO:0016567	79	49.2914	34
THC_THT_c12625	unknown	176	AFK35083	0.021528	-	56	35.4242	51
THC_THT_c12647	hypothetical protein AALP_AA2G028800	169	KFK40690	0.353405	-	45	31.9574	46
THC_THT_c12660	nf-x1-type zinc finger protein nfxl1-like	258	XP_006656848	0.732831	-	47	33.8834	48
THC_THT_c12668	transposable element	107	CAE75955	5.34E-20	GO:0003676	100	87.4261	3.50E+01
THC_THT_c12695	fad-dependent pyridine nucleotide-disulfide oxidoreductase	127	KIZ05075	0.777985	-	61	32.7278	36
THC_THT_c12698	flagellum-specific atp synthase alpha	127	XP_002535442	1.55E-11	GO:0044763	73	6.20E+01	42
THC_THT_c12710	rna polymerase sigma factor rpos	105	EMT00244	0.0378699	-	70	35.8094	34
THC_THT_c12785	hypothetical protein	110	BAC83138	0.0609837	-	51	35.039	49
THC_THT_c12794	retrotransposon ty3-gypsy subclass	106	NP_001068285	5.15E-13	GO:0004523	94	6.78E+01	34
THC_THT_c12837	s-adenosylmethionine synthetase	126	Q8GTL5	1.62E-18	GO:0004478	9.00E+01	82.8037	41
THC_THT_c12862	stomatin-like protein	138	CAN83015	1.16E-04	-	7.10E+01	44.2838	35
THC_THT_c12891	ribosomal protein s8	122	ACN23298	2.15E-09	GO:0005840	8.00E+01	54.6842	40
THC_THT_c12905	ribosomal protein l22	119	YP_635789	1.26E-09	GO:0003735	82	5.51E+01	39
THC_THT_c12910	calm_fagsy ame: full=calmodulin short=	129	Q39752	2.68E-16	GO:0005509	9.70E+01	73.9442	35
THC_THT_c12972	transposon mutator sub-class	106	ABF98152	1.17E-17	GO:0043531	1.00E+02	81.6481	34
THC_THT_c12976	at3g28860-like partial	107	BAG96130	1.71E-14	GO:0010218	1.00E+02	72.4034	35

THC_THT_c12978	acetyl-coenzyme a chloroplastic glyoxysomal	113	CCO19474	8.62E-11	GO:0016208	8.60E+01	61.6178	37
THC_THT_c12997	transposon en spm sub-class	139	AAU89233	3.05E-18	GO:0005739	95	8.36E+01	41
THC_THT_c13017	orf3 gene product	203	YP_588335	6.20E-28	GO:0005739	83	1.04E+02	66
THC_THT_c13019	thaumatin-like protein	118	EAY83985	1.51E-18	GO:0050832	9.40E+01	80.1073	39
THC_THT_c13027	retrotransposon ty1-copia subclass	118	EEE62552	2.45E-16	GO:0003677	9.20E+01	74.7146	39
THC_THT_c13054	alcohol partial	130	ACM17530	4.01E-23	GO:0008270	1.00E+02	94.7449	43
THC_THT_c13070	PREDICTED: uncharacterized protein LOC100838061	176	XP_010229209	0.806069	-	50	33.113	55
THC_THT_c13083	hypothetical protein	124	BAD32891	5.64E-08	-	78	5.08E+01	37
THC_THT_c13125	retrotransposon unclassified	110	ABA93011	1.33E-17	GO:0090502	1.00E+02	81.2629	36
THC_THT_c13160	orf145 gene product	138	NP_064026	1.93E-17	GO:0005739	86	7.70E+01	45
THC_THT_c13180	hypothetical protein OsI_06604	129	EAY85232	2.69E-11	-	8.60E+01	62.7734	37
THC_THT_c13189	retrotransposon unclassified	126	CAE02129	1.14E-17	GO:0004523	9.70E+01	82.0333	42
THC_THT_c13196	retrotransposon unclassified	116	ABA99769	1.57E-17	GO:0008270	9.40E+01	80.4925	37
THC_THT_c13233	protease do-like chloroplastic isoform x1	124	XP_002961432	0.00137616	-	71	40.4318	38
THC_THT_c13235	Os04g0486950	113	NP_001173993	8.88E-19	GO:0006099	100	7.97E+01	37
THC_THT_c13241	NADPH:quinone oxidoreductase, putative	142	XP_002539707	0.15931	-	63	33.8834	38
THC_THT_c13242	hypothetical protein	144	BAD03427	6.82E-09	-	8.80E+01	52.7582	36
THC_THT_c13299	bacterial-induced peroxidase precursor	131	EAZ37178	1.06E-05	GO:0005488	7.80E+01	46.2098	41
THC_THT_c13309	ubiquitin-60s ribosomal protein l40-like	119	NP_001063509	4.74E-07	GO:0005840	100	48.1358	39

THC_THT_c13321	hypothetical protein SORBIDRAFT_06g002025	129	XP_002446122	9.81E-08	-	88	5.01E+01	35
THC_THT_c13338	hypothetical protein (mitochondrion)	120	AAR91193	3.53E-09	GO:0005739	7.80E+01	54.299	38
THC_THT_c13384	glycerol kinase	109	XP_001416856	1.92E-04	GO:0006952	74	4.31E+01	35
THC_THT_c13396	adp-ribosylation factor	121	ABF99503	8.79E-19	GO:0005525	1.00E+02	79.7221	39
THC_THT_c13400	sucrose synthase 1	180	AFW88069	1.38E-24	GO:0045893	9.70E+01	99.7525	47
THC_THT_c13404	probable xyloglucan endotransglucosylase hydrolase protein 16-like	126	CAH66721	2.52E-13	GO:0004553	82	67.0106	4.00E+01
THC_THT_c13428	transposable element transposase_28	105	ABA95871	7.84E-15	GO:0004523	9.70E+01	73.1738	35
THC_THT_c13432	myb transcription factor	149	BAD36192	5.91E-14	GO:0009751	9.30E+01	69.3218	49
THC_THT_c13488	u-box domain-containing protein 35-like	124	NP_001048340	1.72E-18	GO:0006950	100	83.9593	40
THC_THT_c13512	rna polymerase beta subunit	179	KEH17708	7.83E-28	GO:0003899	1.00E+02	112.464	50
THC_THT_c13513	ac083945_4 gag-pol polyprotein	110	AAM93684	1.97E-13	GO:0003723	8.80E+01	65.4698	35
THC_THT_c13552	brefeldin a-inhibited guanine nucleotide-exchange protein 1-like	129	EEE56489	2.92E-18	GO:0043547	1.00E+02	83.5741	42
THC_THT_c13599	hypothetical protein OsJ_02651	117	EEE55008	5.37E-19	-	1.00E+02	80.4925	37
THC_THT_c13617	hypothetical protein OsJ_19723	147	EEE64866	7.15E-05	-	7.00E+01	31.9574	34
THC_THT_c13630	cdk5rap1-like protein	115	XP_009350810	7.11E-13	GO:0044763	8.90E+01	67.0106	38
THC_THT_c13666	glycerol-3-phosphate acyltransferase 1-like	142	EEC82857	5.78E-21	GO:0008152	9.30E+01	89.3521	47
THC_THT_c13678	retrotransposon unclassified	209	ABA97853	2.37E-26	GO:0019752	9.30E+01	108.227	49

THC_THT_c13687	PREDICTED: uncharacterized protein LOC105037323	112	XP_010911304	0.0052005	-	68	38.1206	35
THC_THT_c13696	ribosomal protein l2	168	YP_003433878	2.82E-28	GO:0005762	100	110.538	5.20E+01
THC_THT_c13720	hypothetical protein Salmi_Mp043 (mitochondrion)	101	YP_008992308	3.11E-07	GO:0005739	82	4.81E+01	34
THC_THT_c13725	rna polymerase beta subunit	142	NP_038385	7.15E-11	GO:0003899	81	6.24E+01	44
THC_THT_c13732	hypothetical protein	125	BAD29423	1.44E-16	GO:0016779	9.00E+01	73.1738	41
THC_THT_c13743	protein	115	CCO18692	3.78E-13	GO:0006635	9.20E+01	67.781	38
THC_THT_c13744	hypothetical protein	108	BAD03617	0.34137	-	57	31.5722	35
THC_THT_c13752	alpha-xylosidase 1-like	125	BAA99366	8.28E-20	GO:0004553	1.00E+02	87.8113	39
THC_THT_c13755	photosystem ii d1 protein	160	AFZ40239	3.49E-19	GO:0046872	1.00E+02	82.0333	53
THC_THT_c13771	hypothetical protein	180	BAC99447	2.49E-08	GO:0009536	7.60E+01	55.0694	43
THC_THT_c13774	sec1 family transport protein sly1-like	188	NP_001050677	6.36E-15	GO:0015031	87	74.3294	41
THC_THT_c13792	retrotransposon ty3-gypsy subclass	123	ABA97909	3.08E-09	GO:0003964	9.50E+01	57.7658	41
THC_THT_c13795	retrotransposon ty3-gypsy subclass	139	BAH79971	5.46E-26	GO:0003964	1.00E+02	105.916	46
THC_THT_c13801	cytochrome p450 like_tbp	141	BAD26579	0.00226717	-	70	37.7354	37
THC_THT_c13854	d-lactate dehydrogenase	232	KIZ07710	2.26E-13	GO:0043168	7.30E+01	70.0922	52
THC_THT_c13875	60s ribosomal protein l38	148	EEC68093	2.05E-26	GO:0005840	1.00E+02	98.5969	48
THC_THT_c13917	hypothetical protein B456_001G027400	172	KJB07499	6.27E-05	-	6.30E+01	42.743	36
THC_THT_c13938	macrolide-specific efflux protein maca	113	XP_002537747	4.88E-07	-	72	5.01E+01	37
THC_THT_c13942	ap2 erf and b3 domain-containing protein os01g0693400-like	113	EAZ13178	3.69E-17	GO:0006355	1.00E+02	77.0258	36
THC_THT_c13943	cell wall	159	BAD69086	1.20E-12	GO:0004523	1.00E+02	66.2402	34

THC_THT_c13944	nadh dehydrogenase subunit partial	108	ADL63608	1.14E-16	GO:0016021	1.00E+02	75.485	36
THC_THT_c13953	ribosomal protein s7	109	AAS46086	1.82E-13	GO:0006364	88	65.4698	36
THC_THT_c13978	ribonuclease iii	159	XP_002535468	3.27E-07	GO:0090501	72	5.04E+01	47
THC_THT_c14001	retrotransposon ty1-copia subclass	156	ABA92016	1.07E-26	GO:0003677	9.80E+01	103.605	51
THC_THT_c14006	ribosomal protein l5	110	XP_009350070	1.58E-12	GO:0005840	9.10E+01	64.3142	36
THC_THT_c14010	hypothetical protein Csa_4G652690	183	KGN55456	1.89E-07	-	6.40E+01	50.0618	51
THC_THT_c14037	porphobilinogen deaminase	112	XP_008677338	2.94E-04	GO:0019684	7.60E+01	42.3578	34
THC_THT_c14047	hypothetical protein SORBIDRAFT_06g027595	144	XP_002448466	0.0127091	-	52	35.8094	53
THC_THT_c14063	hypothetical protein M569_00248, partial	221	EPS74531	4.30E-07	-	6.20E+01	49.2914	54
THC_THT_c14077	leucoanthocyanidin dioxygenase	152	XP_011101868	0.436202	-	57	33.4982	47
THC_THT_c14101	retrotransposon ty3-gypsy subclass	244	ABA98195	1.16E-23	GO:0003676	9.80E+01	75.8702	53
THC_THT_c14117	cytochrome b6	209	ABQ08701	5.49E-39	GO:0045158	9.70E+01	133.65	69
THC_THT_c14168	cytochrome p450 cyp81n4	134	ACG27899	6.08E-04	-	6.40E+01	41.9726	39
THC_THT_c14193	heteroglycan glucosidase 1	142	XP_002883444	2.08E-04	GO:0004553	70	4.35E+01	34
THC_THT_c14218	dihydrolipoyllysine-residue succinyltransferase component of 2-oxoglutarate dehydrogenase complex mitochondrial-like	110	KFM27119	1.54E-09	GO:0045252	8.30E+01	56.6102	36
THC_THT_c14220	probable l-type lectin-domain containing receptor kinase	111	EAZ43324	2.05E-16	GO:0004715	1.00E+02	77.411	37
THC_THT_c14243	phosphoribosylaminoimidazole chloroplastic-like isoform x1	131	XP_001690981	8.67E-06	GO:0003824	70	4.74E+01	44

THC_THT_c14314	Os07g0229200	102	NP_001059226	4.01E-13	-	100	67.0106	34
THC_THT_c14317	conserved hypothetical protein	107	XP_002536215	6.35E-08	-	76	5.04E+01	34
THC_THT_c14331	cytochrome c oxidase subunit 1	150	CAJ18329	1.49E-06	GO:0005743	6.50E+01	48.1358	40
THC_THT_c14344	transcription initiation factor tfiid subunit 5-like	112	NP_001058217	1.62E-04	-	64	43.5134	37
THC_THT_c14359	kynurenine alpha-aminoadipate aminotransferase mitochondrial precursor (kat) (kynurenine--oxoglutarate transaminase ii)	190	XP_002535610	5.94E-04	-	59	4.20E+01	59
THC_THT_c14396	conserved hypothetical protein	121	XP_002536521	2.44E-08	-	79	5.43E+01	39
THC_THT_c14398	mitochondrial inner membrane magnesium transporter mrs2	104	EMT02524	1.03E-14	GO:0016021	1.00E+02	71.2478	34
THC_THT_c14423	fructokinase-2-like	140	EMS57576	4.60E-19	GO:0004747	1.00E+02	82.4185	39
THC_THT_c14445	conserved hypothetical protein	110	XP_002537299	1.87E-05	-	69	4.54E+01	36
THC_THT_c14474	hypothetical protein	136	BAD88226	8.33E-10	GO:0016023	8.10E+01	56.9954	38
THC_THT_c14477	ac074196_24 nucleoid dna binding protein	242	EEC67371	0.728234	-	52	33.4982	40
THC_THT_c14517	cell division protein aaa atpase expressed	129	EEC69251	4.55E-14	GO:0005524	9.40E+01	70.8626	37
THC_THT_c14519	photosystem ii cp47 chlorophyll apoprotein	142	ADD63121	4.17E-14	GO:0009523	8.60E+01	71.2478	44
THC_THT_c14619	40s ribosomal protein sa-like	120	EAY90376	4.60E-18	GO:0003735	1.00E+02	80.1073	39
THC_THT_c14646	transposon mutator sub-class	200	ABA95077	8.86E-23	GO:0016020	8.20E+01	97.8265	63
THC_THT_c14652	sensory transduction histidine	158	XP_002537986	0.933078	-	58	32.7278	43
THC_THT_c14671	#NAME?	127	XP_002536489	2.33E-05	GO:0055114	81	4.58E+01	38

THC_THT_c14676	hypothetical protein OsI_23199	118	EAZ01171	3.26E-18	-	1.00E+02	82.0333	39
THC_THT_c14682	retrotransposon ty3-gypsy subclass	122	ABA95226	1.50E-17	GO:0003964	9.70E+01	78.1814	40
THC_THT_c14736	retrotransposon unclassified	151	CAE05494	3.42E-08	GO:0005739	7.60E+01	54.6842	50
THC_THT_c14746	zinc finger (c3hc4-type ring finger) protein family-like	127	BAD08847	1.56E-17	GO:0005739	92	8.09E+01	42
THC_THT_c14779	hypothetical protein	113	AAM93446	0.0648737	-	88	35.4242	36
THC_THT_c14784	glutamine amidotransferase partial	114	XP_010911794	6.06E-09	-	7.60E+01	53.9138	38
THC_THT_c14855	alpha-amylase, putative	124	XP_002535920	5.35E-07	GO:0032450	82	5.04E+01	35
THC_THT_c14891	hypothetical protein CICLE_v10016510mg	113	XP_006449592	0.0765864	-	73	34.6538	34
THC_THT_c14894	chaperone protein mitochondrial-like	109	NP_001046096	2.11E-10	GO:0017111	91	5.78E+01	36
THC_THT_c14957	retrotransposon ty3-gypsy subclass	178	AAR96234	1.14E-13	GO:0004523	91	7.12E+01	35
THC_THT_c14995	hypothetical protein CISIN_1g047371mg, partial	139	KDO38146	0.629776	-	55	32.3426	38
THC_THT_c15019	retrotransposon unclassified	132	AAP06851	5.91E-10	GO:0044260	76	5.97E+01	43
THC_THT_c15032	hgwp repeat containing	126	BAD61206	0.0267244	-	61	35.8094	39
THC_THT_c15043	protein nrt1 ptr family	106	EAY94442	6.65E-09	GO:0016021	8.50E+01	55.4546	35
THC_THT_c15053	hgwp repeat containing	163	AAM00965	3.28E-13	GO:0005739	7.50E+01	69.3218	53
THC_THT_c15083	low quality protein: gtp-binding protein sar1a	164	XP_009339811	8.20E-08	-	7.40E+01	51.6026	35
THC_THT_c15113	myb-like dna-binding domain containing expressed	116	ABF94842	2.50E-16	GO:0003677	100	74.7146	3.80E+01
THC_THT_c15115	cytochrome c oxidase subunit 1	124	XP_001786556	1.88E-10	GO:0004129	82	6.05E+01	40

THC_THT_c15140	uncharacterized membrane protein at1g75140-like	130	EAZ35605	6.19E-21	GO:0016023	1.00E+02	90.5077	42
THC_THT_c15153	glucose-1-phosphate adenylyltransferase large subunit chloroplastic isoform x1	161	EPS60253	2.67E-09	GO:0005524	8.30E+01	57.7658	37
THC_THT_c15158	retrotransposon ty3-gypsy subclass	129	BAD16909	4.09E-09	GO:0005739	73	5.62E+01	41
THC_THT_c15183	rna polymerase beta subunit	102	XP_002462090	3.06E-15	GO:0003899	97	6.97E+01	34
THC_THT_c15192	salt tolerance expressed	120	XP_006650879	1.81E-21	GO:0005829	1.00E+02	89.3521	39
THC_THT_c15225	ac087852_2 reverse transcriptase	103	AAK71542	0.0345086	-	68	35.8094	35
THC_THT_c15228	malate dehydrogenase (oxaloacetate-decarboxylating)(nadp+)	132	XP_010911538	1.48E-13	-	8.10E+01	67.0106	43
THC_THT_c15243	hypothetical protein	118	BAD11659	2.62E-07	-	7.40E+01	48.9062	39
THC_THT_c15271	cell wall	124	BAD26546	3.19E-08	-	1.00E+02	53.9138	40
THC_THT_c15277	inner membrane amino-acid abc	149	XP_002537035	1.06E-07	GO:0006810	66	5.04E+01	48
THC_THT_c15281	predicted protein	114	XP_001754328	0.722041	-	52	32.7278	34
THC_THT_c15323	retrotransposon unclassified	114	AAM01055	2.99E-15	GO:0003677	9.40E+01	74.7146	35
THC_THT_c15325	hypothetical protein	109	BAC79584	0.274694	-	91	32.3426	36
THC_THT_c15348	conserved hypothetical protein	155	XP_002537682	1.01E-08	-	63	5.58E+01	49
THC_THT_c15389	retrotransposon unclassified	106	AAP54617	1.02E-16	GO:0003676	100	7.90E+01	35
THC_THT_c15398	translational elongation factor tu	133	BAA25893	2.22E-13	GO:0003746	8.20E+01	68.1662	45
THC_THT_c15427	hypothetical protein OsJ_20220	106	EAZ35917	8.12E-17	-	1.00E+02	74.7146	35
THC_THT_c15486	lipoic acid synthetase	109	KDD75287	0.0176879	-	67	36.965	37
THC_THT_c15494	retrotransposon unclassified	117	AAM08722	1.01E-08	GO:0009536	82	5.55E+01	35

THC_THT_c15522	pentatricopeptide repeat-containing protein at5g65560-like isoform x1	131	XP_006659286	2.21E-14	GO:0009536	9.40E+01	72.0182	38
THC_THT_c15532	protein	149	XP_002501845	6.25E-11	GO:0006355	91	6.24E+01	34
THC_THT_c15551	cysteine--trna cytoplasmic-like	129	EAZ10047	0.00233078	-	60	40.0466	38
THC_THT_c15565	H0402C08.8	126	CAJ86232	2.24E-21	-	1.00E+02	92.0485	42
THC_THT_c15574	ribosomal protein l2	129	YP_654244	4.84E-15	GO:0003735	100	69.707	35
THC_THT_c15598	hgwp repeat containing	158	BAD01753	5.07E-25	GO:0008270	95	9.74E+01	49
THC_THT_c15601	40s ribosomal protein s3a-like	139	NP_001066631	2.30E-23	GO:0004553	97	94.7449	4.60E+01
THC_THT_c15640	cis-abienol chloroplastic-like	113	XP_009788452	0.710051	-	58	32.7278	36
THC_THT_c15660	nadh dehydrogenase subunit b	150	BAC77586	4.03E-25	GO:0048038	9.70E+01	97.4413	49
THC_THT_c15686	retrotransposon unclassified	265	AAP50935	4.62E-29	-	93	84.7297	4.80E+01
THC_THT_c15697	pectate lyase	152	NP_001057945	1.74E-13	GO:0030570	98	69.3218	50
THC_THT_c15704	hypothetical protein OsI_31340	105	EAZ09078	3.55E-16	GO:0006355	1.00E+02	74.3294	34
THC_THT_c15711	ribosome inactivating protein	103	EAY80077	2.32E-16	GO:0006952	1.00E+02	77.0258	34
THC_THT_c15712	acetolactate partial	110	EEE60896	1.15E-14	GO:0030976	1.00E+02	72.4034	34
THC_THT_c15721	hypothetical protein LOC_Os03g35769	159	ABF97064	9.01E-05	-	7.20E+01	44.669	37
THC_THT_c15736	nb-arc domain containing expressed	132	EEC83165	1.81E-09	GO:0043531	9.10E+01	56.9954	34
THC_THT_c15766	transposon unclassified	112	CAE04442	2.27E-11	GO:0004523	9.10E+01	63.1586	34
THC_THT_c15798	retrotransposon ty3-gypsy subclass	121	ABA97411	0.0046736	-	83	36.965	37
THC_THT_c15824	hypothetical protein	111	BAD09368	1.53E-15	-	1.00E+02	72.4034	36
THC_THT_c15844	f-box domain containing protein	142	BAD68019	1.04E-17	GO:0010106	97	7.82E+01	39
THC_THT_c15865	abc transporter b family member mitochondrial-like	133	EEE68528	1.55E-14	GO:0009941	9.20E+01	73.1738	42

THC_THT_c15867	alpha-galactosidase 2	137	XP_002955466	3.75E-08	GO:0044763	67	5.43E+01	43
THC_THT_c15877	predicted protein	108	XP_001422606	0.119283	-	67	34.2686	34
THC_THT_c15890	30s ribosomal protein chloroplastic-like isoform x1	136	XP_005850532	1.34E-10	GO:0030529	71	5.74E+01	45
THC_THT_c15915	pentatricopeptide repeat-containing protein at3g49142	108	EEE58673	4.20E-16	GO:0016020	9.70E+01	76.6406	35
THC_THT_c16002	hypothetical protein OsI_15534	134	EEC77111	0.0437595	-	56	36.5798	39
THC_THT_c16022	retrotransposon unclassified	122	ABA99021	1.69E-07	-	7.50E+01	51.9878	40
THC_THT_c16066	pentatricopeptide repeat-containing protein mitochondrial	127	NP_001057622	4.79E-15	GO:0009451	97	7.28E+01	35
THC_THT_c16098	protein lateral root primordium 1- partial	116	EMS63375	1.71E-15	GO:0009855	8.90E+01	72.4034	38
THC_THT_c16108	ribulose- -bisphosphate carboxylase oxygenase large subunit	107	AEN71942	8.60E-19	GO:0019253	1.00E+02	81.6481	35
THC_THT_c16125	atpase subunit 8	118	ACZ65560	1.55E-18	GO:0015992	100	79.7221	39
THC_THT_c16160	14-3-3 protein	160	NP_001051051	9.78E-19	GO:0006588	100	77.0258	37
THC_THT_c16185	retrotransposon ty1-copia subclass	137	NP_001175469	5.49E-22	GO:0016021	100	9.47E+01	43
THC_THT_c16190	subtilisin-chymotrypsin inhibitor-2a	117	EMT11499	5.20E-05	-	7.60E+01	41.9726	34
THC_THT_c16193	hypothetical protein	121	BAD68355	0.152672	-	62	32.3426	35
THC_THT_c16195	ethylene receptor	122	KEH41150	0.592964	-	62	33.113	40
THC_THT_c16228	retrotransposon ty3-gypsy subclass	160	AAX94983	7.09E-22	GO:0004523	90	9.32E+01	50
THC_THT_c16259	PREDICTED: LOW QUALITY PROTEIN: uncharacterized protein LOC105117228	192	XP_011013113	1.20E-10	-	8.50E+01	62.7734	34
THC_THT_c16276	retrotransposon ty3-gypsy subclass	129	AAV32123	1.34E-16	GO:0004523	9.20E+01	78.9518	42

THC_THT_c16278	-tetrahydropyridine- -dicarboxylate n-	113	EEC79116	2.02E-10	GO:0030170	8.90E+01	60.077	37
THC_THT_c16280	outer membrane porin protein 32	131	XP_002539867	0.37914	-	58	33.113	41
THC_THT_c16291	reticuline oxidase-like protein	124	NP_001176553	2.23E-12	GO:0050660	85	65.855	3.40E+01
THC_THT_c16302	hypothetical protein OsI_24281	105	EAZ02189	0.00130623	-	62	39.6614	35
THC_THT_c16328	AC093180_24hypothetical protein	144	AAL93077	1.41E-13	GO:0016023	8.60E+01	67.3958	38
THC_THT_c16329	pgr5-like protein chloroplastic	138	YP_514658	3.62E-25	GO:0005739	100	97.4413	45
THC_THT_c16364	probable transport protein	131	XP_002951087	7.97E-07	GO:0006810	78	5.04E+01	42
THC_THT_c16380	rna polymerase beta subunit	127	ACJ24144	2.13E-12	GO:0003899	8.50E+01	64.6994	42
THC_THT_c16381	retrotransposon ty1-copia subclass	107	AAK91877	1.04E-15	GO:0003676	97	7.24E+01	35
THC_THT_c16386	lipoxygenase homology domain-containing protein 1-like	108	NP_001048145	5.82E-16	-	100	73.1738	35
THC_THT_c16412	hypothetical protein OsI_17111	135	EEC77855	9.52E-04	GO:0016021	8.70E+01	41.2022	40
THC_THT_c16423	retrotransposon unclassified	152	ABB47012	6.56E-14	GO:0004523	8.80E+01	66.2402	36
THC_THT_c16432	acetohydroxyacid dehydratase	166	XP_002957711	0.0200397	-	58	37.7354	48
THC_THT_c16465	wall-associated receptor kinase 3	128	EAZ10087	4.61E-10	GO:0030247	8.40E+01	59.3066	39
THC_THT_c16484	elongation factor 2	110	XP_009608307	7.94E-17	GO:0003746	1.00E+02	78.9518	36
THC_THT_c16496	Os02g0508100	126	NP_001046925	5.35E-17	GO:0016023	100	7.39E+01	38
THC_THT_c16505	o-methyltransferase	110	EAZ05653	3.18E-15	GO:0008171	1.00E+02	72.7886	35
THC_THT_c16563	polypyrimidine tract-binding protein homolog 2-like isoform x2	113	XP_009376254	1.39E-18	GO:0003723	9.40E+01	79.337	37
THC_THT_c16565	cell wall	115	BAG92244	2.00E-10	GO:0009536	8.80E+01	57.7658	35
THC_THT_c16569	wrky transcription factor 64-like protein	129	EEE69587	7.11E-20	GO:0008152	1.00E+02	87.0409	42

THC_THT_c16602	ethylene response element binding protein	140	AEK01082	8.05E-18	GO:0006952	1.00E+02	80.4925	37
THC_THT_c16641	hypothetical protein	111	BAC21335	2.85E-15	GO:0005739	91	7.01E+01	37
THC_THT_c16648	PREDICTED: uncharacterized protein LOC104602546	170	XP_010264577	0.288138	-	51	33.8834	56
THC_THT_c16668	valine--trna mitochondrial	114	XP_001692598	1.12E-10	GO:0004832	84	6.12E+01	38
THC_THT_c16705	hypothetical protein OsI_33993	104	EAY78889	5.25E-18	-	1.00E+02	82.4185	34
THC_THT_c16708	e3 ubiquitin-protein ligase upl2-like	105	EEC69178	6.55E-14	GO:0016567	1.00E+02	70.8626	34
THC_THT_c16718	cytocrome p450 like protein precursor	200	CAK18871	1.27E-11	-	9.10E+01	61.2326	35
THC_THT_c16743	hypothetical protein OsI_25390	128	EEC81740	1.37E-19	GO:0009536	1.00E+02	87.0409	42
THC_THT_c16748	type iv secretory system conjugative dna transfer protein	112	KEH15386	3.41E-09	GO:0016020	8.10E+01	56.9954	37
THC_THT_c16778	transposon en spm sub-class	106	AAP12937	6.52E-17	-	100	7.86E+01	35
THC_THT_c16787	hypothetical protein	124	BAD53467	5.19E-08	-	7.70E+01	50.8322	35
THC_THT_c16790	hypothetical protein M569_00228	162	EPS74511	0.00103195	-	63	39.2762	47
THC_THT_c16800	thioredoxin-related protein	151	XP_002535353	1.04E-08	GO:0004540	84	5.58E+01	39
THC_THT_c16876	amp dependent	129	XP_002540260	9.84E-13	GO:0043231	86	6.51E+01	38
THC_THT_c16878	ribosomal protein l2	125	YP_654244	6.20E-22	GO:0005762	100	87.4261	41
THC_THT_c16899	protein hothead-like	140	NP_001053612	5.24E-25	GO:0006066	100	102.064	46
THC_THT_c16903	hydroquinone glucosyltransferase	102	EEC72810	4.20E-04	GO:0016758	9.70E+01	41.9726	34
THC_THT_c16908	Os12g0185900	126	NP_001176824	8.63E-22	-	97	8.67E+01	41
THC_THT_c16909	initiation factor 2	126	XP_005643492	1.75E-07	GO:0044763	70	5.24E+01	41
THC_THT_c16943	hypothetical protein 11M19.13	108	AFJ66169	2.59E-06	GO:0005488	6.60E+01	48.9062	39
THC_THT_c16950	retrotransposon ty3-gypsy expressed	157	BAC15460	1.57E-15	-	94	70.4774	3.80E+01

THC_THT_c16953	abc transporter g family member 11-like	107	XP_009381814	3.08E-16	-	9.40E+01	76.6406	34
THC_THT_c16965	retrotransposon ty3-gypsy subclass	185	AAO73227	2.89E-32	GO:0003677	91	1.20E+02	61
THC_THT_c16989	retrotransposon ty1-copia subclass	110	AAS93255	4.92E-17	GO:0003964	9.40E+01	77.0258	35
THC_THT_c17007	pr1-like protein	118	BAB17132	2.85E-10	GO:0003676	82	5.89E+01	39
THC_THT_c17081	1603356cf orf 249	114	AAM08577	2.57E-16	GO:0016020	9.70E+01	74.7146	37
THC_THT_c17085	mutator-like transposase	149	AAO18442	5.02E-23	GO:0008270	97	9.44E+01	45
THC_THT_c17087	glucose-6-phosphate dehydrogenase	107	KFM26514	0.063749	-	63	35.4242	36
THC_THT_c17127	phosphoenolpyruvate carboxykinase	106	CAR78984	1.66E-06	GO:0006094	7.60E+01	46.595	34
THC_THT_c17148	probable dolichyl-diphosphooligosaccharide--protein glycosyltransferase subunit 3b	124	EEC75585	1.89E-21	GO:0016021	1.00E+02	89.7373	41
THC_THT_c17151	ribosomal protein s16	104	YP_008963886	6.87E-04	GO:0044444	65	38.891	38
THC_THT_c17152	cell division protein homolog 2- chloroplastic isoform x1	106	XP_009404365	0.00760478	-	71	38.1206	35
THC_THT_c17163	retrotransposon unclassified	130	CAE05743	3.73E-21	GO:0009536	95	8.86E+01	43
THC_THT_c17217	TPA: hypothetical protein ZEAMMB73_498562	137	DAA43628	8.86E-22	-	9.70E+01	87.4261	43
THC_THT_c17227	hypothetical protein	127	BAD69364	0.00772261	-	61	38.5058	34
THC_THT_c17256	retrotransposon ty3-gypsy subclass	136	AAX94881	3.96E-23	GO:0003964	1.00E+02	92.4337	45
THC_THT_c17258	glyoxylate reductase	124	NP_001130965	5.10E-09	GO:0051287	81	5.39E+01	37
THC_THT_c17274	thaumatin-like protein 1b	162	XP_010431809	0.0845885	-	48	35.4242	52
THC_THT_c17293	sugar transport protein 5-like	105	BAD73818	9.12E-14	GO:0022891	100	6.86E+01	35
THC_THT_c17306	retrotransposon ty3-gypsy subclass	108	AAO37471	1.88E-12	GO:0004523	94	66.6254	3.50E+01

THC_THT_c17307	retrotransposon ty3-gypsy subclass	129	ABF93731	2.11E-20	GO:0004523	1.00E+02	90.1225	42
THC_THT_c17355	hgwp repeat containing	116	AAT69664	3.21E-12	GO:0009536	9.10E+01	65.855	37
THC_THT_c17377	gag-pol precursor	104	AAQ56303	1.42E-06	GO:0044260	7.60E+01	49.2914	34
THC_THT_c17378	hypothetical protein	134	AAO38449	1.56E-05	GO:0016023	77	45.8246	3.60E+01
THC_THT_c17413	carbamoyl-phosphate synthase large chloroplastic-like	126	XP_002958251	3.57E-12	GO:0007010	94	6.59E+01	36
THC_THT_c17416	OSJNBa0065J03.15	119	CAD40419	4.68E-04	-	76	3.97E+01	34
THC_THT_c17427	retrotransposon ty3-gypsy subclass	206	ABA95104	4.27E-24	GO:0003964	9.00E+01	101.679	54
THC_THT_c17432	rna polymerase beta subunit	190	ADC53593	1.44E-37	GO:0003899	1.00E+02	130.568	63
THC_THT_c17438	unnamed protein product	164	CBI31234	0.0526097	-	57	36.1946	35
THC_THT_c17470	transposon mutator sub-class	131	AAT47050	5.99E-22	GO:0004553	100	9.28E+01	43
THC_THT_c17489	retrotransposon unclassified	121	NP_001063453	2.83E-17	GO:0017148	97	8.01E+01	40
THC_THT_c17512	centromere-specific	122	AAU89210	2.68E-14	GO:0005739	84	7.16E+01	39
THC_THT_c17546	bowman birk trypsin inhibitor	123	EAZ10363	3.93E-23	GO:0010951	1.00E+02	93.5893	41
THC_THT_c17626	sec-independent protein partial	134	YP_009041155	3.06E-19	GO:0016021	93	8.32E+01	44
THC_THT_c17652	tpa: protein kinase domain superfamily protein	139	XP_002535390	2.14E-13	GO:0016772	87	6.93E+01	40
THC_THT_c17701	hypothetical protein LOC_Os10g23240	128	ABB47398	8.50E-12	-	7.70E+01	60.4622	40
THC_THT_c17719	nadh-ubiquinone oxidoreductase chain	123	AGC78955	2.87E-14	GO:0008137	8.60E+01	67.3958	36
THC_THT_c17725	probable transport protein	112	EEC66960	0.00475624	-	64	39.2762	37
THC_THT_c17758	transposon en spm sub-class	135	AAM01090	6.78E-23	GO:0006952	97	91.2781	4.40E+01
THC_THT_c17802	cytochrome b-559 alpha subunit	112	ADZ93677	3.51E-15	GO:0046872	9.40E+01	68.9366	35
THC_THT_c17837	colicin v secretion abc	146	KEH15345	0.0847865	-	50	35.8094	48

THC_THT_c17867	hth-type transcriptional regulator	109	XP_002539231	0.407454	-	61	32.7278	36
THC_THT_c17871	hypothetical protein OsI_14047	129	EEC76404	1.60E-06	-	7.00E+01	48.9062	40
THC_THT_c17889	HRGP	120	ADB54614	0.831522	-	61	32.3426	36
THC_THT_c17901	cinnamyl alcohol dehydrogenase	110	BAD14921	5.98E-17	GO:0045551	1.00E+02	75.485	36
THC_THT_c17943	gdsl esterase lipase	165	XP_003605652	3.25E-04	-	6.50E+01	43.5134	40
THC_THT_c17954	12-oxophytodienoic acid reductase	105	ABV45434	1.41E-17	GO:0009695	97	7.93E+01	35
THC_THT_c18042	transposon en spm sub-class	114	ABA97945	3.94E-15	GO:0006508	9.10E+01	74.3294	37
THC_THT_c18054	ankyrin repeat-like protein	106	NP_001047352	4.37E-20	-	100	87.8113	35
THC_THT_c18087	unknown	126	ACN29210	0.940383	-	55	30.0314	36
THC_THT_c18108	mediator of rna polymerase ii transcription subunit 33a-like	175	XP_008390958	0.317388	-	62	34.2686	35
THC_THT_c18123	tubulin partial	112	AFK39312	8.39E-19	GO:0005874	9.70E+01	78.5666	37
THC_THT_c18136	hypothetical protein	107	BAC56838	0.289479	-	55	33.113	34
THC_THT_c18140	upf0587 protein c1orf123 homolog	106	XP_006644261	7.16E-05	-	6.70E+01	42.3578	34
THC_THT_c18154	transmembrane 9 superfamily member 4-like	134	EEC78593	1.84E-24	GO:0016023	1.00E+02	100.523	44
THC_THT_c18155	magnesium and cobalt transport	107	XP_002540559	5.43E-15	GO:0030001	100	7.05E+01	34
THC_THT_c18160	glycine rich protein	114	NP_001050943	8.63E-15	GO:0009536	100	72.4034	37
THC_THT_c18186	hypothetical protein	126	BAD15764	4.10E-04	-	7.10E+01	41.5874	35
THC_THT_c18202	hypothetical protein VOLCADRAFT_46111	112	XP_002951635	0.0501979	-	64	35.039	37
THC_THT_c18226	retrotransposon ty3-gypsy subclass	119	ABA94416	5.44E-17	GO:0004523	9.40E+01	80.1073	39
THC_THT_c18230	conserved hypothetical protein	148	XP_002538250	0.293478	-	66	33.4982	36
THC_THT_c18233	abc transporter b family member 25-like	113	XP_009350816	5.11E-16	GO:0055085	1.00E+02	76.6406	36

THC_THT_c18287	PREDICTED: uncharacterized protein LOC103503220	124	XP_008465583	0.523404	-	51	33.113	35
THC_THT_c18302	hypothetical protein ZeamMp101	137	YP_588362	3.10E-22	GO:0005739	95	89.3521	4.50E+01
THC_THT_c18326	retrotransposon unclassified	117	CAD40511	6.46E-10	-	7.80E+01	58.9214	37
THC_THT_c18377	plastid ribosomal protein l24	111	XP_001698149	7.93E-04	GO:0044249	71	3.97E+01	35
THC_THT_c18401	probable isoprenylcysteine alpha-carbonyl methylesterase icmel2 isoform x1	124	XP_009775000	0.0333578	-	55	36.5798	40
THC_THT_c18431	partial	109	NP_001176966	3.55E-11	-	88	5.97E+01	36
THC_THT_c18510	retrotransposon unclassified	103	CAH66344	1.37E-10	GO:0004185	9.40E+01	60.8474	34
THC_THT_c18511	retrotransposon unclassified	140	ABA99729	4.08E-12	GO:0004523	9.10E+01	66.2402	35
THC_THT_c18518	leucine-rich repeat receptor-like kinase	114	EEC77414	7.55E-13	GO:0006468	8.60E+01	67.0106	37
THC_THT_c18528	s-adenosylmethionine synthase-like protein	152	AFK33626	1.63E-18	GO:0009651	8.10E+01	81.2629	48
THC_THT_c18613	flagellar l-ring protein	110	XP_002538377	8.53E-07	GO:0003774	82	4.93E+01	34
THC_THT_c18616	zinc c2h2	126	EEC68717	0.001059	-	75	40.817	41
THC_THT_c18637	PREDICTED: uncharacterized protein LOC101768809	115	XP_004987296	7.12E-19	-	9.70E+01	78.5666	37
THC_THT_c18649	hypothetical protein OsI_03033	103	EAY75138	4.82E-14	GO:0043531	1.00E+02	67.781	34
THC_THT_c18678	beta subunit of rna polymerase	140	AFY10135	7.79E-08	GO:0003899	7.90E+01	50.8322	34
THC_THT_c18691	hypothetical protein MNEG_16000, partial	131	KIY91963	0.466586	-	66	30.8018	39
THC_THT_c18709	retrotransposon unclassified	105	ABA93987	7.83E-13	GO:0003676	9.70E+01	67.781	34
THC_THT_c18724	hypothetical protein	107	AAM93710	9.82E-16	-	9.70E+01	75.485	35

THC_THT_c18738	egf domain-specific o-linked n-acetylglucosamine transferase-like	109	EEC79163	2.43E-15	GO:0008152	1.00E+02	73.1738	35
THC_THT_c18739	dentin sialophospho	109	EAZ01765	1.05E-15	GO:0003746	1.00E+02	75.8702	36
THC_THT_c18769	hypothetical protein B456_001G194600	146	KJB10294	0.398143	-	59	32.3426	42
THC_THT_c18881	ring-h2 finger protein atl2l	109	EAZ31565	6.12E-19	GO:0008270	1.00E+02	81.2629	36
THC_THT_c18899	leucine-rich repeat containing protein isoform 2	127	XP_007030145	0.7842	-	54	32.7278	35
THC_THT_c18966	hypothetical protein LOC_Os10g31610	108	AAP54067	6.26E-16	GO:0016023	9.70E+01	73.9442	35
THC_THT_c18975	sigma-e factor regulatory protein rseb	105	XP_002536915	2.51E-10	-	85	5.93E+01	34
THC_THT_c18986	OSJNBb0061C13.9	104	CAE05627	6.85E-16	GO:0008270	100	7.63E+01	34
THC_THT_c18998	citrate synthase 3	106	CDY49249	6.68E-08	GO:0005829	8.50E+01	52.7582	35
THC_THT_c19007	organic cation carnitine transporter 7-like	132	XP_009800311	0.874867	-	62	32.3426	35
THC_THT_c19133	shikimate o-hydroxycinnamoyltransferase-like	114	EAY94737	8.57E-18	GO:0010363	9.40E+01	77.7962	37
THC_THT_c19144	conserved hypothetical protein	157	XP_002525765	0.662576	-	52	32.7278	40
THC_THT_c19152	cell division control protein 50	113	AFW76091	0.0104617	-	64	38.1206	37
THC_THT_c19155	OSJNBb0085H11.3	124	CAE03954	6.54E-10	-	80	5.74E+01	41
THC_THT_c19170	hypothetical protein OsI_34215	123	EAY79110	0.155612	-	57	34.2686	35
THC_THT_c19206	cbl-interacting serine threonine-protein kinase 21 isoform x1	125	XP_009759142	0.00420233	-	62	39.2762	37
THC_THT_c19247	hypothetical protein	125	BAD01355	1.84E-06	GO:0043229	71	4.85E+01	42
THC_THT_c19252	cdk5rap1-like protein	112	XP_009350810	5.73E-11	GO:0044763	8.60E+01	61.6178	37
THC_THT_c19279	udp-glucuronate 4-epimerase 6-like	134	XP_002537217	2.44E-08	GO:0009684	78	5.20E+01	37

THC_THT_c19304	hypothetical protein OsI_15254	121	EAY93455	7.97E-05	GO:0005739	7.80E+01	44.2838	37
THC_THT_c19335	hypothetical protein	113	BAD46323	6.09E-20	-	100	8.09E+01	37
THC_THT_c19387	hypothetical protein	131	BAD10008	1.92E-12	-	8.80E+01	63.1586	36
THC_THT_c19391	fructose-bisphosphate aldolase	130	BAA78593	3.02E-07	GO:0044724	7.20E+01	49.2914	36
THC_THT_c19431	transposon mutator sub-class	116	NP_001175228	8.25E-09	GO:0071704	75	5.58E+01	40
THC_THT_c19438	retrotransposon ty3-gypsy subclass	104	AAR87220	6.53E-16	GO:0004523	100	76.6406	3.40E+01
THC_THT_c19453	PREDICTED: uncharacterized protein LOC101752526	131	XP_004977864	0.400731	-	61	33.113	34
THC_THT_c19478	conserved hypothetical protein	118	XP_002539431	0.17354	-	63	34.2686	36
THC_THT_c19486	upf0481 protein at3g47200-like	129	NP_001042887	3.38E-08	-	81	53.9138	3.70E+01
THC_THT_c19502	gtp diphosphokinase chloroplastic	108	XP_003079611	0.00803379	-	65	38.5058	35
THC_THT_c19547	copper-transporting atpase chloroplastic	130	XP_006849809	2.42E-04	GO:0098655	71	4.31E+01	35
THC_THT_c19583	retrotransposon ty1-copia subclass	136	AAO37470	2.30E-14	GO:0003677	84	6.97E+01	39
THC_THT_c19612	predicted protein	107	XP_001786771	0.00534159	-	74	38.5058	35
THC_THT_c19639	transposon en spm sub-class	157	BAD38108	6.28E-10	-	86	56.9954	38
THC_THT_c19668	flagellar basal-body rod protein	180	XP_011016721	2.56E-06	-	6.50E+01	46.9802	40
THC_THT_c19674	retrotransposon ty3-gypsy subclass	217	ABA97388	3.09E-30	GO:0004523	8.10E+01	117.472	70
THC_THT_c19730	retrotransposon unclassified	130	ABA93735	1.74E-05	GO:0005488	7.30E+01	46.595	38
THC_THT_c19734	e3 ubiquitin-protein ligase sina-like protein 6	111	BAB21179	8.32E-19	GO:0016874	100	8.20E+01	37
THC_THT_c19748	conserved hypothetical protein	104	XP_002539420	0.0539652	-	67	35.4242	34
THC_THT_c19782	hgwp repeat containing	192	BAC83453	3.75E-13	GO:0005739	86	6.62E+01	37

THC_THT_c19792	c3h36_orysj ame: full=zinc finger ccch domain-containing protein 36 short= 3h36	139	EAY99733	4.82E-20	GO:0044767	95	8.20E+01	45
THC_THT_c19801	rna polymerase beta partial	122	AFM29814	8.28E-14	GO:0003899	9.00E+01	69.707	40
THC_THT_c19833	nad transhydrogenase	101	CCO17359	0.0109608	-	76	38.1206	34
THC_THT_c19869	glucose-6-phosphate cytosolic-like	108	XP_003569006	0.0111283	-	62	37.7354	40
THC_THT_c19873	5 -nucleotidase	170	XP_002540227	4.13E-05	-	70	4.51E+01	34
THC_THT_c19895	glutathione s-transferase	147	EAY79292	1.03E-23	GO:0008152	9.70E+01	95.5153	45
THC_THT_c20014	conserved hypothetical protein	114	XP_002536141	2.87E-07	GO:0004872	88	5.12E+01	35
THC_THT_c20054	hypothetical protein VOLCADRAFT_101515	125	XP_002960004	1.97E-13	GO:0046872	92	6.78E+01	39
THC_THT_c20089	conserved hypothetical protein	113	XP_002538005	0.023569	-	72	36.5798	37
THC_THT_c20119	PREDICTED: uncharacterized protein LOC104212508	161	XP_009760100	0.132731	-	60	35.4242	41
THC_THT_c20183	bowman-birk type bran trypsin inhibitor-like	131	XP_006664940	0.519302	-	50	32.7278	46
THC_THT_c20229	heat shock 70 kda protein	112	XP_005647349	3.38E-09	GO:0006457	91	5.70E+01	36
THC_THT_c20257	collinsiai-like partial	143	ADG37942	7.09E-09	GO:0044763	6.50E+01	53.9138	47
THC_THT_c20300	transposon unclassified	123	AAD27554	7.61E-07	GO:0009536	6.90E+01	49.6766	39
THC_THT_c20308	hypothetical protein SORBIDRAFT_04g012210	104	XP_002451986	0.656759	-	57	32.7278	38
THC_THT_c20340	abc transporter a family member 8	176	KFK34078	0.00856651	-	64	39.2762	39
THC_THT_c20343	alkaline neutral invertase cinv2-like	126	NP_001052830	2.01E-19	GO:0033926	100	86.2705	41
THC_THT_c20369	transposable element transposase_28	122	AAX94807	5.23E-15	GO:0004523	91	7.32E+01	37

THC_THT_c20386	acetylajmalan esterase-like	113	XP_010251983	0.00208577	-	62	39.6614	35
THC_THT_c20392	retrotransposon unclassified	166	CAH68540	1.45E-24	GO:0004523	8.80E+01	101.679	54
THC_THT_c20428	hypothetical protein COCSUDRAFT_16367	122	XP_005647078	2.32E-06	GO:0008233	80	4.74E+01	36
THC_THT_c20471	retrotransposon unclassified	154	AAX92792	1.82E-28	-	98	112.464	5.00E+01
THC_THT_c20476	ap2 erf and b3 domain-containing protein os01g0693400-like	114	CDY13581	8.18E-17	GO:0044212	9.20E+01	78.5666	38
THC_THT_c20528	low quality protein: mitogen-activated protein kinase kinase kinase 7-like	118	XP_006644379	0.0167595	-	62	37.3502	35
THC_THT_c20568	#NAME?	116	KIZ03961	0.905564	-	62	31.9574	37
THC_THT_c20606	gida-domain-containing partial	157	CCO16632	0.0344206	-	58	32.7278	34
THC_THT_c20615	pr1-like protein	151	CAI44644	5.03E-07	GO:0003676	8.60E+01	48.521	45
THC_THT_c20662	atp binding cassette	141	EEC80863	0.0104781	-	64	37.3502	39
THC_THT_c20675	hypothetical protein L484_014713	128	EXC34986	0.581603	-	62	30.8018	35
THC_THT_c20676	retrotransposon unclassified	117	ABA97713	9.29E-21	-	1.00E+02	89.3521	39
THC_THT_c20695	hypothetical protein ZEAMMB73_565759	114	AFW87085	1.94E-16	-	9.70E+01	72.0182	37
THC_THT_c20755	hypothetical protein MTR_7g031847	154	KEH22088	4.68E-04	-	6.40E+01	41.5874	37
THC_THT_c20796	predicted protein	114	BAJ99459	1.60E-08	GO:0008152	7.80E+01	55.0694	37
THC_THT_c20836	retrotransposon unclassified	104	ABG66150	7.07E-13	GO:0004523	9.10E+01	67.0106	34
THC_THT_c20949	hypothetical protein BevumaM_p022	143	YP_004222261	8.58E-06	-	69	45.0542	39
THC_THT_c20999	septum site-determining protein	133	CCO15708	0.0388324	-	61	36.1946	44
THC_THT_c21067	metallocarboxypeptidase inhibitor	137	EPS70023	1.40E-05	-	6.40E+01	43.8986	39
THC_THT_c21098	Uncharacterized protein TCM_024341	150	XP_007028506	0.224621	-	59	34.6538	49

THC_THT_c21126	taf1b_orysj ame: full=tata box-binding protein-associated factor rna polymerase i subunit b ame: full=tata box-binding protein-associated factor 1b short=tbp-associated factor 1b	110	Q5W770	1.03E-09	GO:0001187	77	58.151	35
THC_THT_c21141	retrotransposon ty3-gypsy subclass	153	AAV31373	7.89E-30	GO:0003964	9.80E+01	117.472	51
THC_THT_c21148	hypothetical protein PhapfoPp086	125	YP_358632	0.00248086	-	60	37.7354	38
THC_THT_c21180	hypothetical protein L484_018827	132	EXC01915	0.0113542	-	59	36.5798	42
THC_THT_c21257	glycine-rich rna-binding protein mitochondrial-like	112	EPS69562	1.20E-07	GO:0003676	7.80E+01	50.0618	37
THC_THT_c21271	retrotransposon ty1-copia subclass	102	AAP54013	1.59E-15	-	1.00E+02	71.2478	34
THC_THT_c21282	ORF58e	195	YP_001152205	1.47E-09	GO:0009507	75	5.51E+01	36
THC_THT_c21341	predicted protein	105	XP_001786560	4.91E-12	GO:0006289	94	6.51E+01	35
THC_THT_c21353	hypothetical protein VITISV_008784	132	CAN73774	1.25E-05	GO:0005622	7.00E+01	44.2838	34
THC_THT_c21354	BnaUnng02380D	108	CDY67691	2.24E-15	GO:0005739	9.70E+01	69.3218	35
THC_THT_c21371	conserved hypothetical protein	118	XP_002535933	9.09E-08	-	76	5.08E+01	38
THC_THT_c21376	hypothetical protein JCGZ_01322	114	KDP44822	0.0140561	-	52	36.1946	34
THC_THT_c21383	disease resistance protein rpm1-like	165	XP_006663590	3.49E-13	GO:0000166	6.60E+01	69.3218	57
THC_THT_c21395	leucine-rich repeat transmembrane protein kinase 1	142	BAC22547	5.30E-13	GO:0016301	77	6.86E+01	45
THC_THT_c21488	Os06g0719600	117	NP_001058600	7.49E-07	-	100	50.0618	38
THC_THT_c21531	cleavage and polyadenylation specificity factor subunit 3-ii	112	NP_001063107	3.16E-18	GO:0090305	100	82.4185	37

THC_THT_c21548	transposable element transposase_28	119	AAT77814	2.04E-15	GO:0004523	100	7.51E+01	39
THC_THT_c21574	hypothetical protein	112	BAD11580	1.84E-10	-	9.10E+01	56.225	34
THC_THT_c21631	retrotransposon ty3-gypsy subclass	118	AAQ56544	3.49E-14	GO:0004523	9.40E+01	71.633	35
THC_THT_c21645	hypothetical protein SELMODRAFT_165632	104	XP_002963015	8.72E-06	GO:0008152	73	4.70E+01	34
THC_THT_c21653	predicted protein	126	EDQ48481	0.710093	-	56	32.7278	41
THC_THT_c21691	ribonuclease e g-like chloroplastic	135	XP_002535353	3.64E-09	GO:0016070	77	5.66E+01	44
THC_THT_c21731	hsp100 clpb-type	196	CCO15475	3.26E-24	GO:0005737	8.70E+01	101.679	65
THC_THT_c21752	retrotransposon unclassified	115	ABA92239	1.93E-15	GO:0004523	9.20E+01	75.0998	38
THC_THT_c21765	f-box family-1	113	ACM17569	3.12E-15	GO:0080090	1.00E+02	72.4034	37
THC_THT_c21817	heat shock cognate 70 kda expressed	164	AEW08475	3.21E-20	GO:0005524	93	82.8037	43
THC_THT_c21829	hypothetical protein SORBIDRAFT_05g023790	142	XP_002451074	0.367142	-	54	33.4982	46
THC_THT_c21839	cytochrome p450 family protein	125	CBX25250	4.35E-17	GO:0020037	9.70E+01	79.337	37
THC_THT_c21910	isovaleryl- mitochondrial isoform x2	103	XP_011089325	0.978189	-	58	31.9574	34
THC_THT_c21946	embryogenesis transmembrane	101	EEE65559	0.0960021	-	64	35.039	34
THC_THT_c22021	cytochrome b559 subunit alpha	191	XP_003605589	9.54E-25	GO:0046872	9.30E+01	97.0561	48
THC_THT_c22059	poly polymerase beta-like	144	EEE57878	5.79E-10	GO:0043631	8.60E+01	57.7658	38
THC_THT_c22122	retrotransposon unclassified	105	ABA94767	3.26E-13	GO:0006511	9.10E+01	68.9366	35
THC_THT_c22147	rrna intron-encoded homing endonuclease	134	XP_003614389	2.25E-04	-	6.10E+01	43.1282	47
THC_THT_c22164	retrotransposon unclassified	108	CAH66133	1.32E-15	GO:0090502	1.00E+02	75.485	35
THC_THT_c22183	TPA: hypothetical protein ZEAMMB73_440351	120	DAA55318	2.26E-12	GO:0005739	8.60E+01	62.003	38

THC_THT_c22195	nadh dehydrogenase subunit 5	125	ADE60280	7.92E-15	GO:0016021	8.70E+01	70.4774	41
THC_THT_c22239	protein light-dependent short hypocotyls 3-like	116	AAR24161	6.59E-16	-	100	7.12E+01	34
THC_THT_c22288	retrotransposon ty3-gypsy subclass	113	AAW56896	5.55E-04	GO:0044260	7.70E+01	41.9726	36
THC_THT_c22311	hypothetical protein OsI_38133	107	EEC69169	1.99E-17	-	1.00E+02	79.337	35
THC_THT_c22316	P0035H10.20	121	BAB19778	0.00243406	-	100	38.891	40
THC_THT_c22318	receptor-like kinase	105	NP_001049840	7.89E-15	GO:0004672	100	7.24E+01	34
THC_THT_c22344	ribosomal protein l36	127	YP_008757474	6.84E-12	GO:0044444	71	6.05E+01	42
THC_THT_c22365	heat shock 70 kda protein 8	102	XP_004985174	2.01E-04	GO:0006457	1.00E+02	42.743	34
THC_THT_c22489	hypothetical protein AMTR_s00045p00219490	158	XP_006840556	0.614056	-	50	33.4982	36
THC_THT_c22565	subtilisin-like protease	140	BAI67988	4.27E-23	GO:0016787	97	94.3597	4.60E+01
THC_THT_c22569	40s ribosomal protein s23	133	DAA56964	4.16E-17	GO:0006355	9.20E+01	75.0998	40
THC_THT_c22598	hypothetical protein OsI_10566	102	EEC74783	7.41E-10	-	8.50E+01	58.5362	34
THC_THT_c22617	predicted protein	101	BAJ93181	8.46E-05	GO:0046872	7.90E+01	43.8986	34
THC_THT_c22626	retrotransposon unclassified	125	ABA97713	6.93E-14	-	1.00E+02	70.0922	35
THC_THT_c22638	hypothetical protein F775_27690	169	EMT25147	6.61E-07	-	6.10E+01	49.2914	57
THC_THT_c22718	hypothetical protein OsJ_24971	123	EEE67517	2.66E-14	-	8.70E+01	70.8626	40
THC_THT_c22821	protein kinase	139	BAC15455	1.08E-15	GO:0030247	84	7.09E+01	46
THC_THT_c22852	retrotransposon ty3-gypsy sub-class	118	CAD40023	2.95E-18	GO:0004523	1.00E+02	82.4185	39
THC_THT_c22890	hypothetical protein MIMGU_mgv1a023797mg, partial	155	EYU42810	0.381544	-	55	33.4982	38
THC_THT_c22910	hypothetical protein OsJ_22457	108	EEE66267	0.409049	-	55	33.4982	34
THC_THT_c22950	photosystem ii protein m	142	YP_654205	1.99E-04	GO:0009579	55	4.08E+01	40

THC_THT_c22978	seryl-trna synthetase	120	BAD87949	7.62E-18	GO:0005829	100	80.4925	4.00E+01
THC_THT_c23045	retrotransposon unclassified	141	BAD36667	1.10E-20	GO:0003676	100	8.86E+01	42
THC_THT_c23079	unknown	123	ACR36390	1.30E-07	-	8.20E+01	49.6766	34
THC_THT_c23088	hypothetical protein	103	AAR01678	2.63E-16	-	97	74.3294	34
THC_THT_c23127	beta subunit of rna polymerase	111	AFY10150	1.36E-05	GO:0003899	7.90E+01	45.8246	34
THC_THT_c23236	gdsl esterase lipase	213	XP_003605652	0.0052804	-	54	40.0466	48
THC_THT_c23237	transposon en spm sub-class	140	AAX95922	7.66E-24	GO:0006952	93	9.94E+01	45
THC_THT_c23250	Ycf68	153	XP_003610227	0.020586	-	64	37.7354	45
THC_THT_c23260	glutamine-dependent nad(+)	122	XP_002539889	8.72E-06	-	63	4.62E+01	36
THC_THT_c23301	hypothetical protein	115	XP_005647375	0.258718	-	50	33.8834	36
THC_THT_c23329	retrotransposon ty1-copia subclass	107	CAE05417	8.67E-12	GO:0003676	94	6.47E+01	35
THC_THT_c23478	hypothetical protein M569_00407, partial	147	EPS74345	0.00163727	-	68	38.1206	35
THC_THT_c23512	retrotransposon ty1-copia subclass	143	AAX92941	1.68E-09	GO:0090305	88	5.85E+01	36
THC_THT_c23515	cytochrome c oxidase subunit 1	105	EMT20551	4.28E-06	GO:0005507	7.30E+01	46.595	34
THC_THT_c23516	retrotransposon unclassified	137	CAD39524	3.13E-05	-	9.00E+01	45.8246	43
THC_THT_c23524	aminotransferase-like protein	113	BAD61772	7.58E-16	GO:0004523	97	7.63E+01	37
THC_THT_c23539	ornithine carbamoyltransferase	158	XP_005646583	0.0810326	-	59	35.8094	49
THC_THT_c23564	methylmalonyl-mitochondrial	127	KFM27789	1.90E-14	GO:0046872	9.40E+01	72.4034	39
THC_THT_c23733	serine threonine-protein kinase scy1-like	132	XP_001786469	2.20E-05	-	70	4.58E+01	41
THC_THT_c23736	basic 7s globulin-like	133	EAY97993	1.62E-22	GO:0016023	1.00E+02	92.0485	44
THC_THT_c23750	retrotransposon ty3-gypsy subclass	169	AAL31081	0.227997	-	60	34.6538	35
THC_THT_c23751	40s ribosomal protein s13	116	ACN31452	8.16E-19	GO:0005840	1.00E+02	79.337	38

THC_THT_c23812	d-3-phosphoglycerate dehydrogenase isoform 1	101	XP_007042518	1.85E-08	GO:0051287	82	5.47E+01	34
THC_THT_c23822	predicted protein	118	XP_001786793	0.910461	-	55	31.9574	38
THC_THT_c23837	hypothetical protein MTR_3g028073	142	KEH33208	0.379627	-	55	32.3426	36
THC_THT_c23842	hth-type transcriptional regulator	118	XP_002537878	8.05E-06	GO:0003700	74	4.66E+01	39
THC_THT_c23903	hypothetical protein PRUPE_ppb024773mg, partial	112	XP_007204830	6.22E-06	-	72	4.47E+01	36
THC_THT_c23914	nucleolar protein expressed	109	DAA45360	3.63E-16	GO:0009536	1.00E+02	72.4034	35
THC_THT_c23917	centromere-specific	127	AAX95965	8.72E-19	GO:0005488	95	8.43E+01	41
THC_THT_c24037	uncharacterized protein LOC105037498	166	XP_010911455	5.01E-04	-	5.90E+01	42.3578	49
THC_THT_c24042	OSJNBa0085C10.15	106	CAD40063	3.96E-10	GO:0008270	8.20E+01	58.9214	35
THC_THT_c24061	probable receptor-like serine threonine-protein kinase at4g34500	127	XP_008790440	0.550199	-	55	32.7278	36
THC_THT_c24080	indole-3-acetaldehyde oxidase-like	106	NP_001175066	7.55E-18	GO:0050660	100	7.78E+01	35
THC_THT_c24128	v-type proton atpase catalytic subunit a-like	130	NP_001058280	7.82E-18	GO:0046034	100	81.6481	39
THC_THT_c24144	elongation factor mitochondrial-like	109	XP_008234917	9.73E-10	GO:0008270	9.10E+01	58.151	34
THC_THT_c24173	hgwp repeat containing	131	XP_008780864	1.20E-15	GO:0005739	8.80E+01	70.4774	42
THC_THT_c24178	conserved hypothetical protein	149	XP_002537027	1.14E-05	-	72	4.70E+01	37
THC_THT_c24190	hypothetical protein OsJ_14069	134	EEE60634	1.47E-04	-	5.00E+01	43.5134	44
THC_THT_c24194	hypothetical protein (mitochondrion)	158	AGC79019	2.59E-07	-	64	46.2098	3.70E+01
THC_THT_c24208	cytochrome p450 94a1-like	239	EEC67630	2.22E-21	GO:0020037	9.40E+01	93.2041	77

THC_THT_c24235	probable xyloglucan endotransglucosylase hydrolase protein 32	195	XP_004291126	0.404943	-	47	33.8834	53
THC_THT_c24253	conserved hypothetical protein	105	XP_002540134	3.87E-06	-	85	4.70E+01	34
THC_THT_c24307	transposable element retrotrans_gag	133	CAD39983	6.13E-09	-	7.20E+01	55.8398	40
THC_THT_c24377	ribulose bisphosphate carboxylase oxygenase activase	108	NP_001068556	2.02E-17	GO:0005524	100	78.9518	36
THC_THT_c24405	hypothetical protein OsI_12868	127	EAY91253	3.24E-15	-	97	7.12E+01	36
THC_THT_c24421	t cytoplasm male sterility restorer factor 2	120	NP_001057358	1.24E-19	GO:0004029	100	86.2705	39
THC_THT_c24494	ribose-5-phosphate isomerase	170	XP_001778187	8.74E-06	-	65	4.74E+01	58
THC_THT_c24596	probable transport protein	122	XP_002951691	4.57E-06	GO:0015116	77	4.74E+01	40
THC_THT_c24604	hypothetical protein SORBIDRAFT_04g031250	109	XP_002454447	0.146634	-	66	34.2686	36
THC_THT_c24625	hypothetical protein	170	AAT07638	4.81E-09	-	9.10E+01	57.3806	34
THC_THT_c24636	choline monooxygenase	151	CAE17671	9.17E-04	-	5.60E+01	41.5874	50
THC_THT_c24672	transposon en spm sub-class	112	CAE04655	1.02E-16	GO:0005739	97	7.82E+01	37
THC_THT_c24761	orf82	124	YP_588382	8.75E-20	GO:0003735	100	84.3445	37
THC_THT_c24802	serine threonine-protein kinase kipk-like	121	NP_001066769	4.47E-19	GO:0006468	100	83.9593	3.90E+01
THC_THT_c24868	aminotransferase ybdl	137	EMS66098	6.77E-08	GO:0009536	7.20E+01	53.1434	43
THC_THT_c24909	nadh dehydrogenase subunit 7	111	ACH47055	1.02E-12	GO:0051287	83	66.2402	37
THC_THT_c24917	hypothetical protein	117	BAD15701	5.72E-06	-	7.80E+01	44.669	38
THC_THT_c24925	n-ethylmaleimide reductase	104	XP_003062818	0.00536293	-	64	38.5058	34
THC_THT_c24939	hypothetical protein OsI_17568	105	EEC78089	2.50E-07	GO:0006468	1.00E+02	51.6026	35

THC_THT_c25018	glutathione reductase	116	ABK25815	2.44E-08	GO:0050660	8.00E+01	53.1434	36
THC_THT_c25032	hypothetical protein VOLCADRAFT_85069	164	XP_002959854	0.858588	-	48	30.8018	39
THC_THT_c25124	probable glycerophosphoryl diesterphosphodiesteras e 2-like	111	BAD15424	7.87E-19	GO:0006071	1.00E+02	84.7297	37
THC_THT_c25136	unknown protein	127	AAV31304	2.15E-20	-	100	8.40E+01	41
THC_THT_c25143	rna polymerase beta subunit	159	YP_009057880	1.28E-04	-	57	4.47E+01	54
THC_THT_c25184	retrotransposon unclassified	162	CAH66345	8.02E-20	GO:0003676	1.00E+02	77.7962	37
THC_THT_c25186	hat family dimerisation domain containing protein	104	ABA99411	3.09E-16	GO:0003677	1.00E+02	75.485	34
THC_THT_c25193	hypothetical protein ZEAMMB73_027617, partial	144	AFW67235	0.00127429	-	60	38.5058	45
THC_THT_c25254	programmed cell death 6-interacting partial	124	AEJ07955	3.15E-20	GO:0003676	1.00E+02	88.9669	40
THC_THT_c25267	orf127d (mitochondrion)	126	YP_009045751	1.83E-11	GO:0005739	88	6.05E+01	34
THC_THT_c25295	hypothetical protein MTR_029s0005	191	XP_003636121	3.92E-08	GO:0005576	8.00E+01	51.2174	35
THC_THT_c25303	gram domain-containing protein 1a-like isoform x2	131	XP_006464419	0.528677	-	51	33.113	41
THC_THT_c25341	dna gyrase subunit a	119	AAG51377	0.508007	-	64	33.113	39
THC_THT_c25371	mitochondrial ketoacid dehydrogenase e1 alpha	106	XP_001694054	0.0037688	-	60	38.891	35
THC_THT_c25385	chaperone protein dnaj mitochondrial isoform x3	108	CDP11457	4.05E-04	GO:0005488	7.10E+01	41.9726	35
THC_THT_c25404	protein	136	AAF34812	2.20E-23	GO:0003746	9.70E+01	92.0485	45
THC_THT_c25490	retrotransposon unclassified	111	AAL69437	1.44E-16	GO:0008270	94	76.2554	3.60E+01
THC_THT_c25493	Ribonuclease G, putative	108	XP_002535353	0.0037629	-	74	38.891	35

THC_THT_c25497	atp-dependent transmembrane transporter	106	ACN27668	2.22E-15	GO:0016021	1.00E+02	73.559	35
THC_THT_c25537	probable magnesium transporter nipa6	108	XP_002305489	7.58E-07	GO:0016020	86	4.93E+01	36
THC_THT_c25549	leucine-rich repeat extensin-like protein 6	114	EEE66667	3.82E-15	GO:0016301	9.70E+01	71.2478	34
THC_THT_c25596	pyruvate decarboxylase isozyme	102	AAP54301	2.82E-15	GO:0008152	100	70.8626	3.40E+01
THC_THT_c25609	u-box domain-containing protein 30-like	133	EEE58651	1.27E-20	GO:0016567	1.00E+02	88.5817	38
THC_THT_c25640	grf zinc finger family protein	131	BAD26209	9.33E-22	GO:0008270	9.50E+01	88.5817	43
THC_THT_c25662	hypothetical protein MTR_029s0001	164	XP_003636119	0.308347	-	45	31.9574	48
THC_THT_c25678	hypothetical protein MNEG_7937	105	KIZ00023	6.42E-11	-	9.10E+01	59.6918	34
THC_THT_c25691	putative polyprotein	117	AAU44239	0.10341	-	89	35.4242	38
THC_THT_c25714	hypothetical protein OsJ_17691	106	EEE62887	3.30E-15	-	9.40E+01	70.8626	34
THC_THT_c25735	atp synthase subunit beta	144	XP_003614382	6.51E-04	-	5.40E+01	41.9726	48
THC_THT_c25746	dna-directed rna polymerase v subunit 1	111	EEC72510	5.05E-17	GO:0003899	1.00E+02	79.7221	36
THC_THT_c25784	g-type lectin s-receptor-like serine threonine-protein kinase b120-like	127	EEE61754	1.44E-17	GO:0018108	8.30E+01	81.2629	42
THC_THT_c25785	hypothetical protein ZEAMMB73_632002	141	AFW72337	0.244986	-	65	34.2686	35
THC_THT_c25847	phagocytic receptor 1b	126	BAK08235	6.50E-10	GO:0016021	1.00E+02	58.5362	39
THC_THT_c25889	cytochrome c oxidase subunit 3	113	AAW55733	2.92E-14	GO:0004129	8.60E+01	67.781	37
THC_THT_c25892	hypothetical protein	146	BAD81209	0.00223009	-	71	40.4318	35
THC_THT_c26003	cytochrome p450 77a3-like	125	EMT07121	1.88E-19	GO:0020037	1.00E+02	82.0333	40

THC_THT_c26017	calcineurin b-like protein	131	AAM74404	2.78E-15	GO:0005739	9.00E+01	71.633	41
THC_THT_c26059	maturase r	112	CAR85728	1.33E-10	-	7.20E+01	60.8474	44
THC_THT_c26069	retrotransposon ty3-gypsy subclass	120	ABA96534	2.05E-17	GO:0004523	9.70E+01	80.8777	40
THC_THT_c26113	protein kinase	101	AAX94990	7.11E-09	GO:0003676	70	5.55E+01	41
THC_THT_c26196	transposon mutator sub-class	106	AAX95320	4.96E-13	GO:0003676	94	6.70E+01	34
THC_THT_c26208	malate dehydrogenase	117	XP_006664816	9.56E-17	GO:0005737	1.00E+02	76.6406	36
THC_THT_c26232	p-hydroxybenzoic acid efflux pump subunit	149	XP_002535669	3.53E-10	-	69	5.89E+01	49
THC_THT_c26239	retrotransposon unclassified	124	ABA97878	2.69E-20	GO:0090502	9.70E+01	88.1965	38
THC_THT_c26263	retrotransposon unclassified	155	XP_008378650	1.60E-07	GO:0044260	8.30E+01	52.7582	37
THC_THT_c26382	hypothetical protein F775_42766	110	EMT20563	1.64E-11	-	8.00E+01	60.4622	36
THC_THT_c26406	abc transporter g family member 22-like	116	XP_009409037	1.06E-05	-	7.70E+01	46.9802	36
THC_THT_c26452	transposable element	115	ABA98631	1.44E-17	GO:0008270	1.00E+02	80.8777	37
THC_THT_c26494	hypothetical protein OsJ_24925	134	EAZ40471	0.00129414	-	90	40.4318	44
THC_THT_c26506	hgwp repeat containing	114	AAK52156	5.88E-12	-	8.30E+01	61.6178	37
THC_THT_c26555	retrotransposon unclassified	119	AAG59652	6.95E-17	GO:0090502	9.70E+01	77.0258	39
THC_THT_c26579	cytochrome c oxidase subunit 1	172	AAA70311	2.11E-33	GO:0055114	1.00E+02	117.087	57
THC_THT_c26586	hypothetical protein SORBIDRAFT_1180s002020	189	XP_002488959	2.62E-08	-	75	5.20E+01	40
THC_THT_c26589	senescence-associated protein	141	KEH17021	2.22E-04	-	6.80E+01	43.5134	45
THC_THT_c26590	ulp1 protease-like	125	BAD10562	3.81E-16	GO:0008233	1.00E+02	73.1738	35
THC_THT_c26713	hypothetical protein JCGZ_14402	114	KDP28631	7.54E-12	GO:0000272	8.00E+01	64.6994	36
THC_THT_c26729	hypothetical protein OsI_38446	132	EEC69339	2.68E-15	GO:0009536	9.50E+01	71.2478	44
THC_THT_c26731	retrotransposon ty1-copia subclass	169	AAP44652	2.15E-29	GO:0003677	98	1.10E+02	51

THC_THT_c26744	hypothetical protein B456_004G106700	141	KJB21514	0.861516	-	44	30.8018	38
THC_THT_c26754	retrotransposon unclassified	106	AAU44120	1.29E-09	-	8.60E+01	55.0694	36
THC_THT_c26764	hypothetical protein	115	BAD31363	0.118089	-	61	33.8834	34
THC_THT_c26796	hypothetical protein LOC_Os10g07030	127	ABB46796	3.97E-07	-	6.00E+01	50.0618	53
THC_THT_c26879	selenium-binding protein-like	132	BAC80112	3.67E-18	-	9.70E+01	82.8037	43
THC_THT_c26896	hypothetical protein L484_013911	163	EXB38278	0.788545	-	53	30.8018	43
THC_THT_c26938	disease resistance protein rps2	110	EXB38882	0.814589	-	60	32.3426	38
THC_THT_c26956	clathrin heavy chain 1-like	104	XP_008665970	6.19E-15	GO:0030130	1.00E+02	70.8626	34
THC_THT_c26969	g-type lectin s-receptor serine threonine-protein kinase sd3-1	106	EDQ49165	0.47663	-	68	32.7278	35
THC_THT_c27040	hypothetical protein CARUB_v10007161mg	165	XP_006285691	0.536122	-	52	33.4982	42
THC_THT_c27197	conserved hypothetical protein	105	XP_002540391	1.08E-11	GO:0000155	91	6.16E+01	34
THC_THT_c27198	bkrf1 encodes ebna-1	119	BAD62474	0.00180926	-	58	38.1206	39
THC_THT_c27204	ORF46f	120	NP_817268	4.31E-04	-	64	3.93E+01	34
THC_THT_c27233	hypothetical protein LOC_Os11g33150	111	ABA94092	5.93E-12	GO:0003676	9.40E+01	61.2326	36
THC_THT_c27290	Os12g0455000	125	NP_001066725	3.93E-06	GO:0016023	100	45.4394	41
THC_THT_c27341	betaine aldehyde dehydrogenase	125	AFG28557	0.257275	-	58	33.8834	36
THC_THT_c27355	nadh dehydrogenase subunit 10	112	YP_008994807	1.01E-09	GO:0051788	75	5.58E+01	36
THC_THT_c27360	tmv resistance protein n-like isoform x1	135	KCW59106	0.792938	-	53	32.7278	47
THC_THT_c27372	hypothetical chloroplast rf21	114	YP_588327	5.25E-12	GO:0005524	91	6.24E+01	37
THC_THT_c27438	expressed protein	115	ABF98677	1.81E-18	GO:0010027	1.00E+02	80.8777	38
THC_THT_c27509	tryptophan aminotransferase-related protein 4-like	151	EEE55303	4.39E-28	GO:0030170	1.00E+02	109.768	50

THC_THT_c27537	H0313F03.1	118	CAH68333	2.06E-17	GO:0009734	97	7.78E+01	39
THC_THT_c27603	ascorbate peroxidase	156	AAL08496	5.03E-11	GO:0020037	9.10E+01	60.8474	34
THC_THT_c27614	AC078839_20Hypothetical protein	122	AAK13104	0.040294	-	52	36.5798	55
THC_THT_c27621	conserved hypothetical protein	118	XP_002540486	5.19E-05	GO:0044237	68	4.24E+01	38
THC_THT_c27631	probable cytosolic oligopeptidase a	104	XP_011011724	0.204067	-	67	34.2686	34
THC_THT_c27646	AC087852_14hypothetical protein	136	AAK71554	2.81E-11	-	9.10E+01	61.6178	35
THC_THT_c27696	endo- -beta-xylanase d-like	110	ABF94510	7.12E-16	GO:0004553	9.40E+01	75.8702	36
THC_THT_c27698	predicted protein	107	XP_003062032	0.0963049	-	58	35.039	34
THC_THT_c27701	hnh endonuclease domain-containing protein	114	NP_001064372	6.79E-20	GO:0004519	97	83.5741	37
THC_THT_c27710	serine threonine-protein kinase pbs1	108	NP_001055359	3.15E-04	GO:0006468	100	4.24E+01	36
THC_THT_c27735	retrotransposon unclassified	124	BAD35539	3.15E-07	GO:0005739	8.50E+01	48.9062	41
THC_THT_c27739	family transcriptional partial	127	KEH15204	0.00284781	-	64	37.3502	39
THC_THT_c27753	24-methylene lophenol c24 methyltransferase	118	AAC34989	1.62E-13	GO:0016023	9.10E+01	68.1662	35
THC_THT_c27756	chloroplast inorganic partial	103	ABA01129	3.52E-05	-	7.60E+01	43.8986	34
THC_THT_c27820	transposon mutator sub-class	163	CAE05017	7.71E-14	GO:0008270	8.80E+01	62.7734	35
THC_THT_c27851	hypothetical protein F775_00529	137	EMT15825	0.0619898	-	59	34.2686	44
THC_THT_c27853	putative polyprotein	105	AAV43908	8.31E-08	GO:0003676	8.50E+01	53.1434	34
THC_THT_c27881	sucrose synthase 6-like	133	XP_001693570	0.0233322	-	69	36.965	36
THC_THT_c27912	achain photosystem ii d1 c-terminal processing protease	120	XP_002948230	0.00303837	-	65	39.6614	43
THC_THT_c27922	dihydrolipoyl dehydrogenase mitochondrial	104	EMS67619	8.00E-13	GO:0050660	9.70E+01	66.6254	34

THC_THT_c28032	protein rrp5 homolog	116	EEE50471	4.88E-07	GO:0005840	8.10E+01	50.0618	38
THC_THT_c28072	elongation factor g-chloroplastic	128	XP_009350076	2.16E-16	GO:0005525	9.70E+01	78.1814	41
THC_THT_c28081	probable nitrite transporter at1g68570-like	111	AAT85250	1.76E-16	GO:0005215	1.00E+02	77.411	35
THC_THT_c28108	transposon protein	124	AAQ56484	1.75E-12	GO:0003964	8.50E+01	66.6254	41
THC_THT_c28130	alpha-amylase, putative	141	XP_002535920	1.86E-20	GO:0032450	93	8.90E+01	47
THC_THT_c28174	receptor kinase at5g58300-like	123	NP_001050019	5.46E-21	GO:0016023	100	90.5077	41
THC_THT_c28191	cinnamoyl- reductase	112	NP_001062610	1.50E-16	GO:0006694	100	76.2554	37
THC_THT_c28212	glutelin	113	EEC72834	2.07E-16	GO:0005576	1.00E+02	77.0258	36
THC_THT_c28289	hypothetical protein OsI_30201	114	EAZ07947	0.0050954	-	97	38.1206	38
THC_THT_c28304	hypothetical protein MTR_0021s0160	239	KEH17348	7.58E-13	-	6.50E+01	68.9366	58
THC_THT_c28322	f-box protein fbw2-like	119	NP_001057164	3.20E-19	GO:0016874	100	83.1889	39
THC_THT_c28335	nicotianamine synthase 3-like	121	XP_010938261	0.424656	-	50	33.113	34
THC_THT_c28340	hypothetical protein OsI_09569	106	EAY88134	0.229986	-	69	33.8834	36
THC_THT_c28346	hypothetical protein B456_012G000800	121	KJB74657	0.755097	-	60	32.3426	38
THC_THT_c28357	peptide transporter	125	EEE55151	6.88E-09	GO:0000911	1.00E+02	56.6102	41
THC_THT_c28401	family transcriptional partial	110	KEH15204	0.530758	-	61	31.187	36
THC_THT_c28491	predicted protein	102	XP_001786560	1.47E-12	GO:0006289	91	6.66E+01	34
THC_THT_c28531	hypothetical protein	117	BAC84444	1.37E-14	GO:0008270	100	7.01E+01	35
THC_THT_c28540	hypothetical protein OsI_23645	110	EEC80940	0.963349	-	58	31.9574	39
THC_THT_c28544	retrotransposon ty3-gypsy subclass	116	CAD40314	5.12E-20	GO:0004523	9.40E+01	87.4261	38
THC_THT_c28574	retrotransposon unclassified	102	CAE03952	4.27E-09	GO:0004523	7.90E+01	56.9954	34
THC_THT_c28632	hypothetical protein B456_004G025700	113	KJB22023	0.304835	-	57	33.8834	35

THC_THT_c28640	transcription repressor ofp6-like	105	XP_006645066	2.71E-18	-	1.00E+02	79.7221	34
THC_THT_c28646	btb poz domain containing expressed	115	EAY78560	1.20E-18	GO:0005739	1.00E+02	82.0333	37
THC_THT_c28647	cyclin m	173	EEE64204	3.48E-26	GO:0007049	1.00E+02	105.145	49
THC_THT_c28708	conserved hypothetical protein	142	XP_002537339	1.35E-07	-	82	5.24E+01	34
THC_THT_c28755	formaldehyde dehydrogenase s-formylglutathione hydrolase	156	XP_003059058	4.18E-09	GO:0071704	69	5.20E+01	36
THC_THT_c28779	hypothetical protein OsJ_32196	120	EEE51285	0.0148916	-	64	35.4242	34
THC_THT_c28809	nad transhydrogenase subunit alpha	131	KIY93148	0.0949838	-	60	34.6538	41
THC_THT_c28821	pyridoxine pyridoxamine 5 -phosphate oxidase	106	KEH35735	3.60E-09	GO:0005829	8.50E+01	56.6102	35
THC_THT_c28842	tropinone reductase homolog at1g07440-like isoform x1	105	XP_009766392	0.0697181	-	64	35.039	34
THC_THT_c28911	unknown protein	117	BAD82486	5.99E-07	GO:0005739	7.50E+01	50.0618	36
THC_THT_c28978	methylmalonyl-mitochondrial	165	KFM27789	0.00468805	-	66	39.6614	45
THC_THT_c28983	Beta-glucosidase	170	XP_003636137	0.0336463	-	62	36.965	50
THC_THT_c28984	wall-associated receptor kinase 3	128	EEC84735	1.32E-23	GO:0030247	9.70E+01	98.2117	42
THC_THT_c28991	glutathione s-	130	XP_002516073	0.934825	-	51	32.3426	41
THC_THT_c29021	exopolysaccharide production protein	102	XP_002540131	2.64E-08	GO:0047360	79	5.16E+01	34
THC_THT_c29084	swi snf complex component snf12 homolog	128	XP_009774849	0.68498	-	64	32.7278	39
THC_THT_c29114	carrier protein er-ant1	113	XP_006663650	7.64E-08	GO:0048731	7.70E+01	51.9878	36
THC_THT_c29165	uncharacterized protein LOC104098329	112	XP_009603330	6.79E-12	-	9.70E+01	60.4622	34
THC_THT_c29166	mlo-like protein 5-like	129	XP_003568790	1.26E-22	GO:0016021	1.00E+02	95.1301	42
THC_THT_c29167	retrotransposon ty3-gypsy subclass	144	CAE05919	1.00E-11	GO:0004523	9.40E+01	64.3142	34

THC_THT_c29175	BnaC08g03500D	223	CDX77305	0.0350721	-	57	35.4242	49
THC_THT_c29194	protein chloroplastic isoform x1	117	EEC79187	6.49E-18	GO:0019252	1.00E+02	80.4925	39
THC_THT_c29204	kh domain containing expressed	108	NP_001064596	3.48E-12	GO:0016021	91	65.4698	35
THC_THT_c29278	hypothetical protein OsJ_32591	103	EEE51465	0.0310364	-	100	34.2686	34
THC_THT_c29314	hypothetical protein OsJ_10159	123	EEE58708	1.35E-06	GO:0005488	7.60E+01	48.9062	39
THC_THT_c29341	uncharacterized protein LOC101774143	109	XP_004977302	0.169538	-	65	34.2686	35
THC_THT_c29345	hypothetical protein	148	BAD03914	8.20E-28	GO:0005739	9.70E+01	103.219	49
THC_THT_c29452	aminotransferase y4ub	106	EPS66672	0.0139365	-	64	36.5798	34
THC_THT_c29459	protein	123	XP_001422483	0.0649588	-	56	35.4242	39
THC_THT_c29473	unnamed protein product	120	CBI31099	1.63E-04	-	6.40E+01	43.1282	37
THC_THT_c29501	hypothetical protein	120	BAD52849	2.57E-18	-	1.00E+02	77.0258	37
THC_THT_c29553	cell division protease ftsh-like mitochondrial	114	XP_011016757	2.59E-08	GO:0009735	7.80E+01	54.299	38
THC_THT_c29595	pyrophosphate-energized membrane proton pump 3-like	120	AEW08122	3.62E-05	GO:0044765	72	42.3578	37
THC_THT_c29599	probable lipoxygenase chloroplastic-like	107	NP_001062200	9.80E-17	GO:0031408	100	78.5666	35
THC_THT_c29685	speckle-type poz protein	121	NP_001176513	7.32E-23	GO:0005739	100	93.5893	39
THC_THT_c29694	thaumatin-like protein 1	106	XP_009397988	5.67E-12	-	9.10E+01	63.5438	35
THC_THT_c29742	peptide methionine sulfoxide reductase	115	XP_001698443	2.39E-09	GO:0006979	84	5.58E+01	38
THC_THT_c29814	Os07g0568200	130	NP_001060027	1.13E-07	GO:0003677	97	53.1434	4.10E+01
THC_THT_c29816	retrotransposon ty3-gypsy subclass	112	ABA94586	8.53E-16	GO:0003676	9.70E+01	76.2554	37
THC_THT_c29837	dna gyrase subunit chloroplastic mitochondrial	126	XP_004499749	8.14E-12	GO:0009295	8.70E+01	64.6994	41

THC_THT_c29857	atp-binding cassette superfamily	106	XP_003064737	0.0144424	-	70	37.3502	34
THC_THT_c29894	bkrf1 encodes ebna-1	148	BAD29436	2.34E-20	-	9.30E+01	86.6557	47
THC_THT_c29924	retrotransposon ty3-gypsy subclass	109	AAT44184	9.85E-15	GO:0003676	9.70E+01	70.4774	34
THC_THT_c29928	hypothetical protein SELMODRAFT_134934	111	XP_002992207	5.86E-04	-	70	4.16E+01	34
THC_THT_c29951	sucrose-phosphate synthase family protein	125	AFW61931	2.16E-13	GO:0005986	9.40E+01	65.4698	35
THC_THT_c30016	emb1873 partial	106	AIU50720	0.151669	-	60	34.2686	35
THC_THT_c30041	hypothetical protein M569_00248, partial	134	EPS74531	0.0124752	-	70	35.8094	37
THC_THT_c30042	BnaC09g23750D	102	CDY13281	3.96E-05	-	7.60E+01	45.0542	34
THC_THT_c30063	enolase chloroplastic	120	AAL05456	7.42E-06	GO:0044763	7.50E+01	46.9802	40
THC_THT_c30169	two-component system sensor histidine kinase response	125	XP_002536181	6.95E-14	GO:0000160	92	6.78E+01	40
THC_THT_c30185	retrotransposon ty3-gypsy subclass	123	ABA98154	9.38E-15	GO:0090502	9.40E+01	72.7886	37
THC_THT_c30201	conserved hypothetical protein	106	XP_002537671	0.166052	-	64	33.8834	34
THC_THT_c30204	hydroxyacylglutathione hydrolase cytoplasmic	151	XP_002504268	0.00278259	-	63	40.0466	46
THC_THT_c30363	retrotransposon ty3-gypsy subclass	138	AAT69650	3.57E-24	GO:0003964	1.00E+02	100.908	45
THC_THT_c30456	glycosyl hydrolase family 35 protein isoform 2	115	XP_007048528	0.834168	-	54	32.3426	35
THC_THT_c30464	hypothetical protein	115	BAD28401	0.0288051	-	61	34.2686	36
THC_THT_c30651	PREDICTED: uncharacterized protein LOC103418638	113	XP_008354965	8.79E-04	-	7.10E+01	41.2022	35
THC_THT_c30693	predicted protein	128	XP_001422403	0.188086	-	68	34.2686	35
THC_THT_c30709	zinc finger ccch domain-containing protein 53-like	108	NP_001060660	1.26E-15	GO:0046872	100	75.0998	35

THC_THT_c30723	r2r3 transcription factor	140	AFX98074	0.209865	-	61	34.2686	34
THC_THT_c30930	transposon mutator sub-class	118	NP_001175620	8.12E-17	GO:0003676	100	7.86E+01	38
THC_THT_c30945	bifunctional aspartokinase homoserine chloroplastic	125	XP_001786703	2.80E-05	GO:0016301	76	4.54E+01	34
THC_THT_c31015	ac023240_5 secretory protein	109	EAY79023	9.69E-18	GO:0016023	1.00E+02	78.5666	36
THC_THT_c31020	hypothetical protein OsI_14572	111	EEC76635	3.38E-14	GO:0009536	1.00E+02	70.8626	37
THC_THT_c31047	conserved hypothetical protein	127	XP_002536583	9.28E-04	-	73	4.12E+01	34
THC_THT_c31051	Os06g0226000	107	NP_001057201	9.78E-10	GO:0005739	85	5.62E+01	34
THC_THT_c31092	gmp synthase	117	EEC83379	0.00781602	-	56	38.5058	39
THC_THT_c31184	l-type lectin-domain containing receptor kinase -like	118	NP_001053396	3.18E-15	GO:0016023	92	7.43E+01	39
THC_THT_c31205	disease resistance protein rpm1	147	EMT28991	0.00538751	-	70	39.2762	34
THC_THT_c31236	Tua1	109	AHJ80816	3.92E-11	GO:0005874	8.80E+01	62.003	34
THC_THT_c31242	retrotransposon ty3-gypsy subclass	108	AAP53312	1.26E-08	GO:0003676	7.70E+01	52.373	36
THC_THT_c31258	retrotransposon unclassified	109	CAE04866	4.21E-17	GO:0016772	1.00E+02	80.1073	36
THC_THT_c31359	retrotransposon ty3-gypsy subclass	124	AAV43966	1.47E-17	GO:0003676	9.70E+01	81.6481	39
THC_THT_c31390	photosystem ii protein d2	146	ABM91061	1.62E-20	GO:0016021	9.10E+01	85.1149	48
THC_THT_c31394	hypothetical protein	110	BAD53538	0.0103521	-	67	37.7354	34
THC_THT_c31396	spermidine synthase	105	XP_002500355	0.00291413	-	71	39.2762	35
THC_THT_c31455	hypothetical protein VITISV_024584	131	CAN76368	0.00692158	-	65	38.891	35
THC_THT_c31555	predicted protein	116	BAJ97066	1.05E-07	-	6.50E+01	52.7582	38
THC_THT_c31700	udp-n-acetylglucosamine--	124	XP_002536704	0.699112	-	58	32.3426	39
THC_THT_c31708	syd chromatin remodeling atpase	112	EEC80339	2.44E-16	GO:0010182	1.00E+02	78.1814	36

THC_THT_c31709	hypothetical protein OsI_36947	108	EEC68596	0.235005	-	100	33.8834	35
THC_THT_c31715	at3g26020-like protein	117	XP_001766093	0.137424	-	55	34.6538	38
THC_THT_c31730	Os08g0110800	127	NP_001175357	7.57E-18	-	97	8.05E+01	42
THC_THT_c31837	alpha-xylosidase, putative	128	XP_002535873	0.00166831	-	66	40.0466	39
THC_THT_c31930	tpa: class iii peroxidase 61	105	CAJ86146	1.89E-15	GO:0046872	100	7.28E+01	35
THC_THT_c31999	hypothetical protein OsJ_21868	105	EEE65967	2.73E-13	GO:0003676	9.70E+01	68.5514	34
THC_THT_c32011	lrr and nb-arc domains-containing disease resistance	103	XP_007045385	3.71E-05	-	67	4.54E+01	34
THC_THT_c32055	lecithin-cholesterol acyltransferase-like 1-like	104	NP_001045383	9.01E-15	GO:0008374	100	71.633	34
THC_THT_c32214	uncharacterized protein LOC100788557	113	XP_006599934	0.552981	-	54	33.113	35
THC_THT_c32216	hypothetical protein	104	AAP44698	1.27E-10	-	78	58.5362	38
THC_THT_c32284	rrna intron-encoded homing partial	164	EXB97336	1.89E-12	-	7.00E+01	64.6994	54
THC_THT_c32296	hypothetical protein SORBIDRAFT_07g000710	124	XP_002443712	0.173775	-	61	34.2686	34
THC_THT_c32349	hypothetical protein OsI_20484	108	EEC79467	8.22E-15	GO:0009536	100	7.28E+01	34
THC_THT_c32363	hypothetical protein	117	BAD09503	1.99E-12	GO:0005739	9.10E+01	62.3882	35
THC_THT_c32646	delta-aminolevulinic acid dehydratase chloroplast precursor	124	KIY98724	6.47E-10	GO:0009536	7.50E+01	55.8398	40
THC_THT_c32650	ribosomal protein s10	114	EPS70026	3.53E-05	-	6.60E+01	42.743	36
THC_THT_c32785	uncharacterized protein LOC103643452	117	XP_008664863	4.63E-12	-	8.60E+01	64.3142	38
THC_THT_c32873	atp binding cassette	132	EEC80863	3.50E-18	GO:0009536	9.70E+01	79.7221	42
THC_THT_c32930	uncharacterized protein LOC18444618 isoform X2	103	XP_011627216	0.0545252	-	58	33.8834	34

THC_THT_c33007	ribulose bisphosphate carboxylase large chain	165	EXC35991	2.61E-09	-	7.80E+01	57.7658	38
THC_THT_c33020	annotation was added to scaffolds in november 2011~long chain fatty acid- ligase	112	XP_003592425	0.0127236	-	69	37.7354	36
THC_THT_c33052	hypothetical protein JCGZ_01322	158	KDP44822	0.0420783	-	52	35.4242	46
THC_THT_c33071	pyruvate orthophosphate dikinase	104	KIZ07928	8.42E-05	GO:1901576	7.00E+01	41.9726	34
THC_THT_c33112	AC051624_24Unknown protein	108	AAK92566	1.68E-16	-	1.00E+02	78.1814	35
THC_THT_c33226	cyt-p450 monooxygenase	147	NP_001057709	3.88E-11	GO:0016491	67	62.7734	49
THC_THT_c33242	Protease, putative	125	XP_002536196	6.88E-06	GO:0016787	71	4.74E+01	38
THC_THT_c33309	protein	157	XP_005646550	7.07E-05	-	68	43.8986	38
THC_THT_c33326	multidrug resistance protein	133	XP_010913032	0.642741	-	54	33.113	50
THC_THT_c33335	hypothetical protein F775_27690	153	EMT25147	0.0503532	-	68	35.8094	35
THC_THT_c33479	Os01g0511200	106	NP_001043171	1.76E-14	GO:0005739	100	69.707	35
THC_THT_c33659	conserved hypothetical protein	111	XP_002536490	8.67E-07	-	69	4.97E+01	39
THC_THT_c33661	hlh dna-binding domain superfamily protein	129	AGV02636	2.85E-18	GO:0003677	1.00E+02	82.8037	39
THC_THT_c33725	transposon en spm sub-class	125	ABA96304	2.02E-06	-	1.00E+02	49.2914	37
THC_THT_c33898	cysteine synthase	116	ABF98863	6.77E-17	GO:0005516	9.70E+01	76.2554	37
THC_THT_c33907	hypothetical protein	114	AAR01636	0.154275	-	67	34.2686	34
THC_THT_c33930	transposon en spm sub-class	146	ABA93968	0.0785825	-	62	35.8094	35
THC_THT_c33936	glutathione s-	104	XP_002536752	4.30E-07	GO:0004364	79	4.93E+01	34
THC_THT_c33938	probable alanine--trna chloroplastic isoform x1	111	XP_001753125	7.94E-06	GO:0003723	71	4.74E+01	35

THC_THT_c34145	senescence-associated protein	112	XP_006279313	1.97E-07	-	75	4.89E+01	37
THC_THT_c34201	atp-dependent rna helicase dhx29	121	XP_010930827	0.383889	-	63	33.4982	38
THC_THT_c34215	aquaporin tip1-1-like	120	P50156	5.18E-19	GO:0016021	100	82.4185	39
THC_THT_c34217	hypothetical protein VOLCADRAFT_101504	107	XP_002959991	0.0398019	-	70	36.1946	34
THC_THT_c34264	hypothetical protein	116	BAD29276	6.41E-15	GO:0016023	9.40E+01	68.9366	38
THC_THT_c34309	Os05g0242000	103	NP_001055004	2.27E-15	-	100	73.9442	34
THC_THT_c34367	e3 ubiquitin-protein ligase upl1-like	159	XP_006279888	0.906877	-	56	33.113	44
THC_THT_c34443	choline monooxygenase	115	AAP88973	1.38E-18	GO:0019285	1.00E+02	79.7221	37
THC_THT_c34502	rna binding protein	237	BAC83836	0.00761518	-	68	39.2762	38
THC_THT_c34503	422kdazein-associated intercluster -typepolpolyprotein	117	AAK13102	2.41E-10	GO:0006508	7.50E+01	60.4622	37
THC_THT_c34541	g-type lectin s-receptor-like serine threonine-protein kinase at5g35370-like	105	EAZ09359	1.37E-15	GO:0016023	1.00E+02	75.485	35
THC_THT_c34580	delta-1-pyrroline-5-carboxylate synthase	127	EMS47116	0.0817249	-	63	35.8094	41
THC_THT_c34590	hydroxyacyl dehydrogenase, putative	113	XP_002534847	6.80E-05	GO:0008152	72	4.43E+01	37
THC_THT_c34622	3-deoxy-d-arabino-heptulosonate 7-phosphate synthetase	105	KIZ00500	2.77E-06	GO:0016740	7.40E+01	47.3654	35
THC_THT_c34628	serine threonine protein phosphatase 2a 57 kda regulatory subunit b alpha isoform-like	128	BAC79198	2.90E-07	GO:0008601	97	5.12E+01	42
THC_THT_c34663	hypothetical protein OsI_15534	112	EEC77111	2.42E-05	GO:0050896	7.70E+01	46.2098	35
THC_THT_c34667	anthranilate synthase component	138	XP_005849906	3.75E-09	GO:0009414	73	5.47E+01	42

THC_THT_c34734	type i inositol -trisphosphate 5-phosphatase cvp2-like	105	XP_008777973	8.97E-07	-	7.90E+01	49.6766	34
THC_THT_c34828	retrotransposon unclassified	130	AAM14690	2.33E-08	GO:0003964	7.60E+01	54.6842	42
THC_THT_c34961	at-hook protein 1	110	BAJ91030	1.37E-15	GO:0003677	100	74.7146	3.60E+01
THC_THT_c34973	polyketide hydroxylase-like	132	XP_002964027	3.77E-06	GO:0009793	75	4.85E+01	36
THC_THT_c34990	probable glutamate--trna cytoplasmic	135	EEE54984	1.70E-11	GO:0005737	8.20E+01	64.3142	39
THC_THT_c35029	hypothetical protein	171	BAD54077	1.35E-04	-	8.40E+01	41.9726	46
THC_THT_c35186	hypothetical protein PHAVU_006G011900g	155	XP_007146093	6.94E-07	-	72	4.81E+01	37
THC_THT_c35271	-like zinc-binding alcohol dehydrogenase family protein isoform 1	132	XP_007011409	0.594432	-	58	32.7278	41
THC_THT_c35283	hypothetical protein B456_001G160400	151	KJB09728	0.00497152	-	70	36.965	34
THC_THT_c35344	unnamed protein product	121	BAB03088	0.919789	-	58	32.3426	39
THC_THT_c35407	hypothetical protein MTR_029s0005	119	XP_003636121	0.07527	-	63	33.113	36
THC_THT_c35473	hypothetical protein MTR_029s0004	128	XP_003636120	0.137648	-	61	35.039	44
THC_THT_c35475	succinate semialdehyde dehydrogenase	139	EMS60803	3.51E-14	GO:0051287	8.00E+01	71.633	46
THC_THT_c35499	Putative retroelement	146	AAN04947	0.0446805	-	54	36.1946	48
THC_THT_c35555	metal transporter nramp3-like	167	XP_005650309	4.49E-15	GO:0020037	84	7.43E+01	52
THC_THT_c35673	f-box protein at5g49610-like	124	EAZ23891	6.62E-12	-	8.00E+01	64.3142	41
THC_THT_c35676	transposon mutator sub-class	103	ABA97523	0.00664645	-	58	38.5058	34
THC_THT_c35684	homodimeric type	110	XP_005650755	7.80E-07	GO:0016812	73	4.93E+01	34
THC_THT_c35778	conserved hypothetical protein	141	XP_002535436	0.279562	-	67	34.2686	34
THC_THT_c35807	transketolase	122	EEE69878	2.42E-10	GO:0016787	8.20E+01	60.4622	41
THC_THT_c35823	twitching mobility	153	XP_002534939	0.0132805	-	60	38.1206	40

THC_THT_c35837	hypothetical protein OsI_16222	136	EEC77429	3.04E-09	-	7.50E+01	55.4546	37
THC_THT_c35931	nadh dehydrogenase	104	BAK07097	4.88E-16	GO:0005747	1.00E+02	71.2478	34
THC_THT_c35950	cytokinin dehydrogenase 1 precursor	113	Q6YW51	8.74E-15	GO:0005615	100	7.28E+01	36
THC_THT_c36157	40s ribosomal protein s11-like	106	AFK45967	3.90E-18	GO:0005840	1.00E+02	76.6406	35
THC_THT_c36171	sucrose synthase	205	EAZ00002	7.10E-23	GO:0005985	8.70E+01	97.8265	56
THC_THT_c36234	leucine-rich repeat extensin-like protein 3	149	EEC69256	1.26E-19	GO:0016023	9.70E+01	85.5001	42
THC_THT_c36258	gdsl esterase lipase	165	XP_003605652	0.0243463	-	59	37.7354	37
THC_THT_c36264	uncharacterized protein LOC102715909	109	XP_006664595	0.39697	-	55	33.113	36
THC_THT_c36386	subtilisin-like protease	107	EAZ22112	3.08E-11	GO:0005618	8.80E+01	62.3882	35
THC_THT_c36492	hypothetical protein MTR_029s0004	146	XP_003636120	0.131714	-	51	35.039	39
THC_THT_c36537	12-oxophytodienoic acid reductase	119	AGN53438	1.60E-07	GO:0009695	9.10E+01	50.0618	36
THC_THT_c36555	endoglucanase 11	138	EEC84529	8.41E-24	GO:0008810	1.00E+02	95.5153	46
THC_THT_c36579	dipeptidyl peptidase 8-like	117	XP_001753273	0.00991829	-	60	38.1206	38
THC_THT_c36655	duf1685 family protein	149	XP_006648617	2.86E-17	-	9.70E+01	77.7962	35
THC_THT_c36659	zinc knuckle containing	115	AAT01356	0.00102407	-	48	40.0466	56
THC_THT_c36725	zinc-binding protein	121	EAY84842	2.27E-13	-	9.70E+01	66.2402	34
THC_THT_c36733	rossmann-fold nad -binding domain-containing protein	133	EMT22857	0.0688624	-	55	35.4242	40
THC_THT_c36778	lon protease	102	EYU17851	7.45E-07	GO:0004252	8.50E+01	48.1358	34
THC_THT_c36886	H0306F03.3	104	CAJ86190	5.38E-04	-	6.00E+01	40.0466	38
THC_THT_c36947	retrotransposon unclassified	135	CAE01610	2.56E-22	GO:0090502	1.00E+02	92.0485	44
THC_THT_c36956	cytochrome p450 89a2-like	134	EAY73941	1.02E-20	GO:0020037	9.70E+01	89.7373	44

THC_THT_c36976	aldo keto reductase family protein	122	ACF83482	0.794925	-	65	31.9574	38
THC_THT_c37011	copper-transporting atpase ran1-like	112	XP_003079479	0.00104295	-	78	41.2022	37
THC_THT_c37147	mterf family protein	110	NP_001059486	3.18E-16	-	100	75.8702	36
THC_THT_c37244	hypothetical protein OsI_19865	141	EAY97947	1.97E-21	GO:0009536	9.50E+01	88.1965	47
THC_THT_c37287	tetratricopeptide repeat-like superfamily protein	145	XP_007016541	0.82554	-	50	32.7278	38
THC_THT_c37310	nad transhydrogenase	113	KFM27067	4.26E-05	GO:0044699	7.10E+01	45.4394	35
THC_THT_c37398	uncharacterized protein LOC105043683	131	XP_010919643	0.276781	-	61	33.8834	34
THC_THT_c37404	nadh dehydrogenase	106	CEF99780	4.36E-06	GO:0071704	7.60E+01	47.7506	34
THC_THT_c37515	cytochrome p450	120	EAZ23153	8.54E-20	GO:0004497	1.00E+02	85.5001	39
THC_THT_c37600	hypothetical protein	175	BAD35690	2.64E-22	GO:0009536	95	8.82E+01	46
THC_THT_c37623	fructose- - cytosolic	111	NP_001055659	2.23E-16	GO:0005737	100	7.36E+01	35
THC_THT_c37710	retrotransposon ty3-gypsy subclass	131	XP_008780563	4.88E-20	GO:0004523	1.00E+02	84.7297	42
THC_THT_c37876	hypothetical protein	119	BAD72307	2.76E-18	GO:0016023	100	77.7962	3.90E+01
THC_THT_c37897	hypothetical protein PHAVU_003G142600g	152	XP_007154729	3.94E-05	-	67	4.31E+01	34
THC_THT_c37908	abc transporter g family member 11-like	123	EAZ08292	3.42E-18	GO:0009536	1.00E+02	80.1073	38
THC_THT_c38001	mitochondrial alternative oxidase 1a	133	NP_001046676	2.57E-13	GO:0009916	78	6.62E+01	47
THC_THT_c38006	66 kda stress	120	XP_009347803	0.538821	-	54	33.113	42
THC_THT_c38069	biotin synthase	108	XP_002504367	3.32E-06	GO:0044763	72	4.78E+01	36
THC_THT_c38092	transposon unclassified	160	ABA97639	4.42E-18	GO:0005739	8.30E+01	78.9518	49
THC_THT_c38139	y2641_orysj ame: full=b3 domain-containing protein os02g0764100	106	EEC74055	7.75E-15	GO:0009536	9.40E+01	72.7886	35
THC_THT_c38163	hypothetical protein OsJ_18065	122	EEE63255	8.20E-05	-	7.80E+01	43.8986	37
THC_THT_c38298	unnamed protein product	160	XP_003082223	0.00592254	-	66	36.965	36

THC_THT_c38322	pentatricopeptide repeat-containing protein chloroplastic-like	108	XP_010235742	0.618357	-	50	32.7278	44
THC_THT_c38348	protease do-like partial	102	XP_002962675	9.26E-08	GO:0031977	85	51.2174	34
THC_THT_c38408	carbohydrate-binding-like fold partial	115	AIU49953	0.0278455	-	45	36.965	40
THC_THT_c38590	cytochrome p450 family protein	155	EAY82190	3.51E-08	GO:0046872	7.80E+01	54.299	38
THC_THT_c38618	serine threonine protein kinase	109	XP_010238974	0.0625695	-	58	35.8094	34
THC_THT_c38638	thiol-disulfide oxidoreductase	133	XP_002540313	7.71E-07	GO:0044699	66	4.89E+01	39
THC_THT_c38834	predicted protein	108	XP_001786955	5.47E-06	-	80	4.58E+01	36
THC_THT_c38861	retrotransposon unclassified	105	AAT47012	6.07E-11	GO:0004523	9.40E+01	62.003	34
THC_THT_c38865	gag-pol precursor	145	AAP06922	2.70E-10	GO:0005739	74	6.01E+01	55
THC_THT_c38904	hypothetical protein COCSUDRAFT_28854	106	XP_005648005	2.41E-05	-	77	4.51E+01	35
THC_THT_c38912	aldehyde dehydrogenase family 7 member b4-like	115	XP_009627665	0.011503	-	63	37.7354	36
THC_THT_c38966	unknown protein	119	BAC99374	0.00286384	-	67	38.891	34
THC_THT_c38973	aminotransferase-like	108	BAD30508	3.11E-11	GO:0008483	86	6.16E+01	36
THC_THT_c39017	Os12g0504950	122	NP_001176966	8.12E-12	-	82	6.16E+01	40
THC_THT_c39053	hypothetical protein Csa_4G243390	114	KGN53991	0.96593	-	60	31.9574	40
THC_THT_c39071	disease resistance protein rpp13	125	EMT31810	0.810582	-	64	32.3426	39
THC_THT_c39251	c4-dicarboxylate transport transcriptional regulatory protein	113	KEH15415	1.31E-05	GO:0003677	7.20E+01	45.8246	37
THC_THT_c39262	uncharacterized abhydrolase domain-containing protein ddb_g0269086-like	157	XP_010454677	0.767097	-	46	32.7278	56
THC_THT_c39294	retrotransposon ty3-gypsy subclass	155	ABA92238	4.59E-14	GO:0003676	8.30E+01	71.2478	43

THC_THT_c39298	methyltransferase-like protein 10-like	116	KDD71455	0.129303	-	59	33.113	37
THC_THT_c39341	hypothetical protein OsI_15405	130	EAY93619	6.36E-18	-	1.00E+02	81.2629	36
THC_THT_c39476	phospho-2-dehydro-3-deoxyheptonate aldolase chloroplastic-like	110	XP_008656395	7.35E-13	-	8.60E+01	64.6994	36
THC_THT_c39587	ring-h2 finger protein atl79-like	116	XP_004951209	0.992723	-	56	31.5722	37
THC_THT_c39598	retrotransposon ty3-gypsy subclass	106	AAT44267	4.14E-10	GO:0004523	8.50E+01	59.6918	35
THC_THT_c39614	conserved hypothetical protein	110	XP_002537367	3.40E-04	-	68	4.20E+01	35
THC_THT_c39618	dihydrolipoamide dehydrogenase of glycine decarboxylase from pisum sativum	135	XP_001767995	8.01E-07	GO:0016668	76	5.01E+01	38
THC_THT_c39649	peptidyl-dipeptidase dcp, putative	118	XP_002535455	9.66E-06	GO:0070011	68	4.70E+01	38
THC_THT_c39782	ac074282_6 polyprotein	190	ABA92518	7.16E-15	GO:0005739	8.40E+01	72.7886	53
THC_THT_c39891	retrotransposon unclassified	130	ABA97878	1.35E-09	GO:0090502	1.00E+02	58.151	40
THC_THT_c39924	retrotransposon ty3-gypsy subclass	171	AAP53721	8.46E-14	GO:0004523	8.90E+01	67.3958	37
THC_THT_c39943	retrotransposon unclassified	144	ABA97386	2.30E-25	-	1.00E+02	103.219	43
THC_THT_c39959	senescence-associated protein	118	XP_011013113	0.0124906	-	59	38.1206	37
THC_THT_c39967	cell wall-associated hydrolase	150	XP_003637074	0.00106842	-	71	41.5874	35
THC_THT_c40047	nph3 family domain-like protein	105	AEP03595	0.00262725	-	71	39.6614	35
THC_THT_c40079	PREDICTED: uncharacterized protein LOC101756236	118	XP_004967010	8.59E-04	-	7.80E+01	41.2022	41
THC_THT_c40090	mlo-like protein 10	116	XP_008786683	0.564785	-	54	32.7278	35
THC_THT_c40136	hypothetical protein SELMODRAFT_402549	112	XP_002961003	0.413587	-	58	32.7278	34

THC_THT_c40325	retrotransposon ty3-gypsy subclass	105	NP_001173596	7.86E-13	GO:0004523	88	6.59E+01	35
THC_THT_c40339	uncharacterized protein LOC100275709	118	NP_001143206	0.338966	-	53	33.4982	39
THC_THT_c40354	cysa sulfate abc transporter subunit	116	AFI43875	0.0780533	-	62	35.039	35
THC_THT_c40428	protein tar1-like	161	XP_006279324	1.89E-06	-	63	4.66E+01	47
THC_THT_c40497	tpa: tat pathway signal sequence family protein	109	DAA58360	0.996631	-	67	30.8018	34
THC_THT_c40540	-	115	XP_002534563	0.0228699	-	55	36.965	36
THC_THT_c40542	rna helicase	117	XP_001415624	0.146032	-	58	34.2686	43
THC_THT_c40595	hypothetical protein B456_007G194600	117	KJB43296	0.295905	-	56	31.5722	39
THC_THT_c40682	30s ribosomal protein chloroplastic-like	120	XP_009350065	1.78E-04	GO:0003676	8.10E+01	43.1282	38
THC_THT_c40728	hypothetical protein LOC_Os10g09390	115	ABB46914	5.05E-09	-	8.50E+01	55.4546	34
THC_THT_c40733	gdsl esterase lipase	188	XP_003605652	0.00521453	-	50	40.0466	62
THC_THT_c40779	probable wrky transcription factor 19-like isoform x1	125	EEE65702	0.0585141	-	57	35.8094	35
THC_THT_c40887	oxidoreductase, putative	137	XP_002535383	0.00529398	-	56	38.891	44
THC_THT_c40888	hypothetical protein POPTR_0008s13380g	120	XP_002312458	0.957656	-	52	32.3426	34
THC_THT_c40892	lrr receptor-like serine threonine-protein kinase gso1-like	135	AAU44328	2.21E-13	GO:0016301	1.00E+02	69.707	35
THC_THT_c40924	iron-sulfur assembly mitochondrial-like	119	EMT00243	8.48E-09	GO:0005488	7.60E+01	53.1434	34
THC_THT_c40973	hypothetical protein OsI_08432	120	EEC73764	1.24E-20	GO:0003723	9.70E+01	87.4261	40
THC_THT_c41043	hypothetical protein	106	BAC84132	4.36E-05	-	7.60E+01	42.3578	34
THC_THT_c41056	probable acyl-activating enzyme peroxisomal	127	KFK29403	0.0309233	-	60	36.965	40

THC_THT_c41135	hypothetical protein MtrDRAFT_AC149601g18 v2	149	ABN06041	0.217361	-	55	33.113	49
THC_THT_c41210	transcription factor rf2b-like	108	EAY89914	1.38E-06	GO:0006355	1.00E+02	48.521	36
THC_THT_c41219	aldehyde dehydrogenase family 2 member c4-like	118	XP_003525266	1.67E-04	GO:0008152	7.30E+01	43.1282	34
THC_THT_c41228	trehalase 1	114	CCO15451	0.166013	-	61	34.6538	34
THC_THT_c41312	g-type lectin s-receptor-like serine threonine-protein kinase at2g19130-like	114	EEC70205	4.11E-19	GO:0006468	1.00E+02	84.7297	37
THC_THT_c41480	cysteine-rich receptor-like protein kinase 8-like	124	XP_006599641	0.123966	-	56	35.039	37
THC_THT_c41519	wnk6_orysj ame: full=probable serine threonine-protein kinase wnk6 short= 6	104	NP_001065816	1.67E-12	GO:0006468	97	65.855	3.40E+01
THC_THT_c41526	hypothetical protein	141	BAE98425	0.7368	-	50	30.8018	42
THC_THT_c41556	abc transporter-like protein	107	KIZ04396	6.14E-10	GO:0003700	8.50E+01	58.9214	35
THC_THT_c41625	multidrug resistance	105	EAY84812	6.64E-17	GO:0010541	1.00E+02	79.337	34
THC_THT_c41683	rrna intron-encoded homing endonuclease	154	XP_003614385	0.0152654	-	60	38.1206	46
THC_THT_c41712	cell wall	135	BAD25618	7.40E-22	GO:0003676	95	9.17E+01	43
THC_THT_c41758	mutator-like transposase	108	CAE02515	1.51E-15	GO:0016020	94	7.55E+01	36
THC_THT_c41844	malonate-- ligase	164	KDO56939	1.53E-04	-	6.30E+01	43.8986	44
THC_THT_c41880	hypothetical protein	104	BAI67988	3.37E-14	GO:0005739	100	69.3218	3.40E+01
THC_THT_c41987	retrotransposon unclassified	117	ABA96448	1.74E-09	-	83	5.78E+01	36
THC_THT_c42072	hypothetical protein	136	BAD28385	0.0165583	-	60	35.4242	43
THC_THT_c42148	diguanylate cyclase cyclic diguanylate partial	105	XP_001786955	0.00188438	-	64	38.891	34
THC_THT_c42193	predicted protein	153	XP_001764999	0.189607	-	48	34.2686	39

THC_THT_c42230	hypothetical protein CICLE_v10001436mg	211	XP_006432276	0.72959	-	39	33.113	58
THC_THT_c42301	gdsl esterase lipase at5g37690-like	131	NP_001057321	7.15E-24	GO:0016788	95	96.6709	43
THC_THT_c42321	hypothetical protein	108	BAD25073	6.07E-15	GO:0006355	100	7.24E+01	34
THC_THT_c42351	retrotransposon ty1-copia subclass	114	CAE01741	1.11E-16	GO:0003676	9.40E+01	78.1814	38
THC_THT_c42411	Os08g0110800	130	NP_001175357	1.21E-09	-	78	5.78E+01	42
THC_THT_c42468	retrotransposon unclassified	116	ABB47094	3.18E-04	-	6.80E+01	42.3578	38
THC_THT_c42511	phosphate regulon transcriptional regulatory protein	123	XP_002534677	4.17E-05	-	71	4.27E+01	38
THC_THT_c42533	transposable element retrotrans_gag	145	AAT81689	1.65E-16	-	92	7.63E+01	41
THC_THT_c42586	conserved hypothetical protein	110	XP_002537142	1.26E-04	-	66	4.31E+01	36
THC_THT_c42603	tetratricopeptide repeat protein 38	112	ABF98282	2.22E-19	-	1.00E+02	84.7297	37
THC_THT_c42635	AF132121_1laccase	120	AAK37825	0.973304	-	55	32.3426	40
THC_THT_c42700	peptidase m1 family protein	130	CAZ44325	5.65E-10	GO:0005829	7.60E+01	59.3066	39
THC_THT_c42717	hypothetical protein CHLNCDRAFT_6585, partial	110	XP_005850457	0.142104	-	62	34.6538	35
THC_THT_c42798	Os05g0326650	165	NP_001174354	0.00305044	-	66	40.4318	39
THC_THT_c42874	retrotransposon unclassified	103	ABA93987	8.70E-12	GO:0003676	8.80E+01	64.6994	34
THC_THT_c42878	retrotransposon ty3-gypsy subclass	113	AAQ56570	3.39E-13	GO:0003964	9.40E+01	65.855	34
THC_THT_c42892	hypothetical protein OsI_25390	132	EEC81740	3.06E-20	GO:0009536	9.70E+01	88.9669	44
THC_THT_c42949	protein	113	XP_002946469	4.16E-04	GO:0016491	72	4.20E+01	36
THC_THT_c43081	retrotransposon unclassified	111	EEE59290	4.63E-16	GO:0006468	9.40E+01	76.6406	37
THC_THT_c43137	pentatricopeptide repeat-containing	140	BAD53318	4.23E-18	-	8.80E+01	78.5666	44
THC_THT_c43233	hypothetical protein AALP_AA8G069500	117	KFK25126	0.452063	-	56	33.113	37

THC_THT_c43257	hypothetical protein SORBIDRAFT_02g007935	125	XP_002459644	0.423596	-	55	33.4982	40
THC_THT_c43267	late embryogenesis abundant protein lea14-a-like	116	EAY99203	1.37E-19	GO:0009269	1.00E+02	82.0333	38
THC_THT_c43296	hypothetical protein OsI_21088	134	EEC79740	0.0316236	-	53	36.965	41
THC_THT_c43397	acetyl- carboxylase beta subunit	110	YP_003227083	1.31E-06	GO:0008270	79	4.89E+01	34
THC_THT_c43431	protease do-like chloroplastic	126	KIZ00787	2.78E-04	-	6.80E+01	41.2022	38
THC_THT_c43492	predicted protein	113	XP_001418300	0.284184	-	65	33.8834	35
THC_THT_c43679	granule-bound starch synthase	130	ACH91165	9.37E-06	GO:0044249	6.20E+01	45.8246	43
THC_THT_c43728	vic family transporter: inwardly rectifying potassium ion channel	113	CCO14839	0.00462158	-	74	39.2762	35
THC_THT_c43735	conserved hypothetical protein	116	XP_002536583	0.787216	-	52	32.3426	38
THC_THT_c43782	retrotransposon ty3-gypsy subclass	110	CAE75991	3.36E-16	GO:0090502	1.00E+02	75.8702	36
THC_THT_c43836	phototropin 2	134	XP_002500378	4.10E-06	GO:0007165	71	4.54E+01	38
THC_THT_c43859	hypothetical protein LOC_Os12g11270	139	ABA96756	8.97E-05	-	6.80E+01	41.5874	35
THC_THT_c43946	hypothetical protein	157	AAT01409	8.28E-06	-	7.20E+01	47.3654	40
THC_THT_c43963	cytochrome c biogenesis fn	123	AAR91193	6.62E-21	GO:0017004	1.00E+02	85.8853	40
THC_THT_c43972	hypothetical protein AMTR_s00049p00133800	121	XP_006852216	0.553106	-	51	32.7278	39
THC_THT_c43973	expressed protein	114	ABA94838	2.20E-19	-	1.00E+02	82.8037	37
THC_THT_c44008	unnamed protein product	112	CDP19802	0.00724052	-	62	38.5058	35
THC_THT_c44041	glycoside hydrolase family 2 protein	104	KIY98312	0.00244109	-	67	40.0466	37
THC_THT_c44090	adenylosuccinate chloroplastic	103	XP_001778414	7.65E-05	GO:0004019	79	4.39E+01	34
THC_THT_c44116	predicted protein	133	BAK03025	0.0603762	-	59	36.1946	47

THC_THT_c44151	zeta-carotene desaturase	117	EEE54237	1.60E-07	-	7.50E+01	52.373	37
THC_THT_c44172	wall-associated receptor kinase 2-like	113	EAY77727	1.76E-17	GO:0030247	1.00E+02	80.1073	37
THC_THT_c44229	subtilisin-like protease	126	BAD53008	1.72E-08	GO:0016023	83	5.43E+01	36
THC_THT_c44257	60s ribosomal protein l6	134	EEE61175	1.49E-15	GO:0005840	1.00E+02	72.7886	35
THC_THT_c44269	hypothetical protein OsI_15236	133	EEC76953	0.00540405	-	75	38.891	41
THC_THT_c44288	arylformamidase, putative	127	XP_002536305	1.89E-14	GO:0008152	90	6.93E+01	41
THC_THT_c44295	hypothetical protein Csa_6G408190	228	KGN47860	3.19E-05	-	7.20E+01	44.2838	44
THC_THT_c44347	hypothetical protein	142	BAI68004	1.22E-25	GO:0005739	1.00E+02	99.3673	47
THC_THT_c44416	conserved hypothetical protein	120	XP_002538056	0.0815906	-	65	35.039	38
THC_THT_c44417	hypothetical protein MTR_0021s0200	160	KEH17350	0.506222	-	56	32.3426	37
THC_THT_c44453	retrotransposon unclassified	133	ABA99728	7.66E-17	GO:0016023	8.60E+01	79.337	43
THC_THT_c44481	monocopper oxidase-like protein sku5	114	NP_001065019	9.91E-10	GO:0005507	100	5.55E+01	35
THC_THT_c44518	retrotransposon ty1-copia subclass	102	ABA98225	7.89E-15	GO:0003677	1.00E+02	73.1738	34
THC_THT_c44694	atp binding cassette	112	XP_002538058	3.76E-05	GO:0016020	73	4.43E+01	34
THC_THT_c44695	adenylosuccinate synthetase chloroplastic-like	145	XP_011016369	0.00477704	-	73	39.2762	34
THC_THT_c44731	serine threonine phosphatase	140	EAZ26559	7.94E-19	GO:0046872	9.50E+01	82.0333	40
THC_THT_c44799	probable e3 ubiquitin-protein ligase ari2-like	126	NP_001049474	1.80E-21	GO:0008270	100	92.0485	41
THC_THT_c44830	f-box domain containing protein	112	EEE53318	2.62E-09	-	8.30E+01	56.6102	36
THC_THT_c44880	conserved hypothetical protein	109	XP_002536395	5.09E-06	-	75	4.74E+01	36

THC_THT_c44900	cobalt-zinc-cadmium resistance protein czcc	124	XP_002535850	4.88E-08	-	77	5.31E+01	40
THC_THT_c44908	hypothetical protein CICLE_v10033492mg	150	XP_006438333	6.74E-05	-	64	4.24E+01	42
THC_THT_c44973	grf zinc finger family protein	122	AAT85205	4.89E-06	-	6.50E+01	45.4394	40
THC_THT_c44975	transcription binding factor	134	EAY77922	0.0217762	-	66	36.965	36
THC_THT_c44996	hypothetical protein L484_004636	172	EXB92316	2.37E-05	-	7.40E+01	45.0542	35
THC_THT_c45009	ac090486_23 phosphatidate cytidylyltransferase	106	AAM08813	0.00124502	-	52	40.4318	46
THC_THT_c45014	retrotransposon ty1-copia subclass	125	BAD52830	5.18E-10	-	91	5.62E+01	34
THC_THT_c45021	predicted protein	105	XP_001702648	1.84E-07	GO:0050660	82	5.16E+01	35
THC_THT_c45043	gdsl-motif lipase hydrolase-like	109	BAD22300	3.76E-16	GO:0006629	100	75.0998	36
THC_THT_c45046	conserved hypothetical protein	115	XP_002535544	1.68E-07	-	81	4.93E+01	37
THC_THT_c45080	hypothetical protein	101	BAD25784	0.0144779	-	61	35.039	36
THC_THT_c45099	cation diffusion facilitator family	114	XP_002508320	0.0577012	-	70	35.4242	34
THC_THT_c45111	conserved hypothetical protein	114	XP_002537470	3.55E-05	-	74	4.31E+01	35
THC_THT_c45137	predicted protein	159	XP_001786133	1.66E-10	-	69	5.89E+01	46
THC_THT_c45145	PREDICTED: uncharacterized protein LOC105118890	127	XP_011015250	6.06E-08	-	6.90E+01	53.1434	42
THC_THT_c45281	hypothetical protein OsI_14569	109	EEC76632	1.18E-04	GO:0009536	6.90E+01	43.1282	36
THC_THT_c45304	Os12g0211000	113	NP_001066402	9.08E-17	-	94	7.74E+01	37
THC_THT_c45311	rna polymerase sigma factor sigc-like	171	XP_003551127	1.90E-07	-	6.40E+01	52.7582	56
THC_THT_c45348	aconitate hydratase	131	CCO19745	6.69E-16	GO:0006099	9.30E+01	76.6406	43
THC_THT_c45369	protein aspartic protease in guard cell 2-like	106	XP_010928895	1.00E-07	-	7.70E+01	52.373	35

THC_THT_c45395	atpase family aaa domain-containing protein 1-like	109	NP_001068410	5.57E-16	GO:0005524	100	7.36E+01	35
THC_THT_c45409	pre-mrna-splicing factor cwc22 homolog	121	EEE53027	1.76E-20	GO:0016311	1.00E+02	89.7373	40
THC_THT_c45422	oberon-like protein	126	EEC68513	4.03E-19	GO:0005783	1.00E+02	85.1149	39
THC_THT_c45444	hypothetical protein	123	BAC16477	1.01E-05	-	60	4.47E+01	38
THC_THT_c45461	chaperone protein dnaj 1	103	AFK47956	0.117872	-	64	33.4982	34
THC_THT_c45493	stomatin-like protein mitochondrial isoform x2	121	XP_001703625	2.04E-07	-	74	5.12E+01	39
THC_THT_c45502	metacaspase type ii	110	NP_001151968	1.25E-06	-	77	48.9062	3.60E+01
THC_THT_c45506	unnamed protein product	119	CBI34638	0.0987485	-	60	35.039	38
THC_THT_c45583	outer membrane omp85 family protein	104	BAG92827	2.29E-14	GO:0019867	1.00E+02	71.2478	34
THC_THT_c45593	hypothetical protein VOLCADRAFT_101534	146	XP_002960023	1.57E-18	GO:0003857	91	8.13E+01	46
THC_THT_c45595	hypothetical protein	111	BAD54291	4.20E-10	-	8.80E+01	56.9954	34
THC_THT_c45598	probable beta-d-xylosidase 6	102	ABA95273	3.60E-18	GO:0005975	1.00E+02	82.8037	34
THC_THT_c45601	bifunctional monodehydroascorbate reductase and carbonic anhydrase nectarin-3-like	131	EEC83601	2.45E-14	GO:0016023	9.10E+01	69.3218	36
THC_THT_c45602	chloroplast processing peptidase-like	119	XP_002535590	6.24E-08	GO:0016020	78	5.16E+01	37
THC_THT_c45645	acetyl- synthetase-like protein	131	XP_005643306	7.56E-10	-	75	5.93E+01	41
THC_THT_c45671	retrotransposon ty3-gypsy subclass	105	CAD40007	2.62E-16	GO:0003676	1.00E+02	77.7962	34
THC_THT_c45675	pentatricopeptide repeat-containing protein mitochondrial-like	125	NP_001130698	0.195406	-	60	34.2686	40
THC_THT_c45819	nodulation protein	138	XP_002538438	3.67E-05	-	69	4.27E+01	42
THC_THT_c45845	dentin sialophospho	112	NP_001053905	4.01E-15	GO:0005886	100	7.16E+01	35

THC_THT_c45886	dehydration responsive protein	118	BAJ11784	0.896997	-	61	31.5722	36
THC_THT_c45981	hypothetical protein VITISV_020092	150	CAN66945	0.494394	-	46	33.4982	39
THC_THT_c46008	orf115a	207	XP_002539035	7.74E-17	GO:0005739	82	6.01E+01	34
THC_THT_c46097	glycerol kinase-like	137	XP_010033977	0.00919342	-	67	38.5058	34
THC_THT_c46137	sugar transport protein 5-like	116	EAY86382	3.69E-14	GO:0022891	1.00E+02	70.8626	35
THC_THT_c46290	3-hydroxybutyryl-dehydrogenase	173	XP_005847699	1.11E-10	GO:0008691	82	6.05E+01	46
THC_THT_c46358	aminotransferase-like protein	118	AAM01157	1.43E-16	GO:0008152	100	7.63E+01	35
THC_THT_c46543	hypothetical protein ZEAMMB73_662964	116	AFW74739	5.80E-06	GO:0016491	6.60E+01	46.9802	36
THC_THT_c46555	retrotransposon ty3-gypsy subclass	119	EEE53112	3.28E-20	GO:0004523	1.00E+02	87.8113	38
THC_THT_c46637	unknown protein	112	AAP13011	1.41E-14	GO:0009536	9.70E+01	69.3218	35
THC_THT_c46672	retrotransposon unclassified	136	AAQ56318	1.76E-18	-	9.30E+01	81.6481	45
THC_THT_c46712	transketolase thiamin-binding protein	118	KEH15587	3.11E-11	GO:0005975	9.10E+01	63.1586	34
THC_THT_c46725	aquaporin nip2-1-like	125	XP_006647882	4.36E-19	GO:0005887	1.00E+02	82.8037	41
THC_THT_c46726	hypothetical protein OsJ_32543	121	EEE51446	0.192623	-	48	34.2686	39
THC_THT_c46804	hypothetical protein VOLCADRAFT_32796	103	XP_002949776	0.196885	-	57	33.4982	35
THC_THT_c47000	OSJNBa0029L02.26	103	CAE04485	0.766169	-	52	31.9574	36
THC_THT_c47119	bahd acyltransferase dcr	134	XP_009800795	0.799631	-	51	32.7278	41
THC_THT_c47127	hypothetical protein OsJ_02599	104	EEE54984	6.80E-09	GO:0005737	8.80E+01	56.225	34
THC_THT_c47166	retrotransposon unclassified	105	ABF95977	0.255197	-	70	33.8834	34
THC_THT_c47174	hypothetical protein OsJ_22738	127	EEE66398	0.14277	-	52	35.039	42
THC_THT_c47192	pirin-like protein	108	XP_005847586	4.91E-09	-	82	5.55E+01	34
THC_THT_c47205	mannosylglycoprotein endo-beta-mannosidase isoform x2	110	XP_010489622	0.947977	-	55	32.3426	34

THC_THT_c47317	uncharacterized protein LOC102719475	149	XP_006651215	0.190414	-	58	34.6538	39
THC_THT_c47334	luminal-binding protein 5	130	EMT04333	0.00329999	-	60	39.6614	38
THC_THT_c47336	conserved hypothetical protein	109	XP_002538048	0.0261356	-	61	36.1946	34
THC_THT_c47458	transposon en spm sub-class	105	CAE02227	1.68E-12	GO:0005739	9.70E+01	66.2402	34
THC_THT_c47496	retrotransposon ty3-gypsy subclass	122	ABA98874	2.36E-04	GO:0004523	1.00E+02	43.1282	40
THC_THT_c47536	4-diphosphocytidyl-2-c-methyl-d-erythritol chloroplastic-like isoform x4	136	AAZ80385	4.52E-06	GO:0009416	6.50E+01	47.7506	41
THC_THT_c47551	Os08g0454700	116	NP_001175608	1.02E-07	-	72	5.04E+01	36
THC_THT_c47599	ulp1 protease-like protein	154	BAD26022	2.02E-16	GO:0003676	9.20E+01	75.485	40
THC_THT_c47624	vernalization independence	107	CAJ98738	4.51E-09	GO:0080008	8.00E+01	55.0694	35
THC_THT_c47679	retrotransposon ty1-copia subclass	115	AAP03379	5.13E-17	GO:0003676	97	76.2554	3.40E+01
THC_THT_c47708	gmp synthase	122	KFM28773	3.40E-11	GO:0016462	82	6.28E+01	39
THC_THT_c47709	Os07g0277600	139	NP_001175137	0.985304	-	67	30.4166	37
THC_THT_c47737	predicted protein	140	XP_002508982	0.624616	-	60	32.3426	40
THC_THT_c47784	AC084831_3hypothetical protein	151	AAK52149	2.45E-06	-	69	4.89E+01	46
THC_THT_c47787	transposable element transposase_28	149	CBX24439	3.87E-05	-	6.00E+01	45.0542	40
THC_THT_c47809	tpr domain containing protein	105	EMT08627	0.781584	-	60	31.9574	35
THC_THT_c47874	luminal-binding protein 5	108	XP_001780840	5.37E-09	GO:0005788	85	5.62E+01	34
THC_THT_c47908	uncharacterized protein LOC100837226	107	XP_003580783	1.24E-16	-	9.40E+01	75.0998	35
THC_THT_c48008	protein ultrapetala 1-like	116	EEC79483	8.67E-15	GO:0008270	92	7.05E+01	38
THC_THT_c48030	conserved hypothetical protein	108	XP_002537579	4.35E-04	GO:0055085	82	4.04E+01	34

THC_THT_c48087	transposon unclassified	149	AAP06919	0.950507	-	69	31.9574	49
THC_THT_c48099	early nodulin	115	EAZ35784	6.93E-16	GO:0005739	9.40E+01	71.2478	35
THC_THT_c48134	hypothetical protein	144	BAD05202	3.46E-15	-	100	7.09E+01	37
THC_THT_c48305	hypothetical protein CHLNCDRAFT_57451	107	XP_005849215	0.00991796	-	66	38.1206	36
THC_THT_c48333	retrotransposon ty3-gypsy subclass	114	ABB47490	1.07E-14	GO:0004523	9.40E+01	73.1738	38
THC_THT_c48427	hypothetical protein OsI_05556	123	EAY84177	0.135132	-	57	33.8834	40
THC_THT_c48663	cdk5 regulatory subunit-associated protein 1-like	131	XP_009350810	3.31E-04	-	7.00E+01	42.3578	34
THC_THT_c48669	ubiquitin-conjugating enzyme e2 7	109	AFK34371	5.92E-16	GO:0016881	9.70E+01	71.633	36
THC_THT_c48711	hypothetical protein AMTR_s00151p00074930	106	ERM95510	0.0107858	-	77	36.5798	35
THC_THT_c48797	sulfate thiosulfate import atp-binding protein	128	XP_006388327	4.28E-11	GO:0009536	82	5.93E+01	39
THC_THT_c48807	phosphate chloroplastic	134	XP_002534816	0.00216587	-	61	40.0466	44
THC_THT_c48998	isovaleryl-dehydrogenase mitochondrial-like	129	BAJ99459	3.79E-13	GO:0050660	8.10E+01	68.5514	43
THC_THT_c49004	inositol phosphorylceramide glucuronosyltransferase 1	107	XP_011090499	0.00598963	-	77	38.891	35
THC_THT_c49051	hypothetical protein	110	AAT01409	1.48E-12	-	9.40E+01	65.4698	36
THC_THT_c49055	retrotransposon ty3-gypsy subclass	116	CAD39841	4.63E-05	GO:0004523	8.90E+01	45.4394	38
THC_THT_c49101	mfs transporter	122	KEH15169	8.40E-04	-	6.70E+01	40.817	34
THC_THT_c49213	atp-dependent dna partial	134	XP_005647316	0.253517	-	59	34.2686	42
THC_THT_c49245	Os08g0499100	103	NP_001062146	5.58E-14	GO:0043531	100	70.8626	3.40E+01
THC_THT_c49250	glutamate-1-semialdehyde- -aminomutase	109	XP_007211242	0.307323	-	51	33.4982	35

THC_THT_c49273	transposon unclassified	111	ABG22600	1.86E-04	-	7.30E+01	42.3578	34
THC_THT_c49309	f-box domain containing protein	194	EAY83081	9.32E-04	-	6.80E+01	41.9726	63
THC_THT_c49484	leukotriene a-4 hydrolase	129	XP_002967717	4.03E-14	GO:0005829	85	7.12E+01	41
THC_THT_c49489	nuclear transcription x-box	142	XP_002533849	0.174924	-	67	35.039	46
THC_THT_c49518	sugar abc	141	XP_002535080	0.00511278	-	64	39.2762	37
THC_THT_c49548	zinc knuckle family protein	122	BAC66217	4.10E-16	GO:0008270	1.00E+02	72.0182	38
THC_THT_c49556	speckle-type poz protein	123	NP_001058318	4.47E-21	-	100	88.9669	40
THC_THT_c49579	retrotransposon ty3-gypsy subclass	104	AAN09865	1.72E-10	GO:0004523	8.50E+01	60.8474	34
THC_THT_c49584	hypothetical protein	147	BAC83667	0.0243279	-	64	36.1946	37
THC_THT_c49650	50s ribosomal protein chloroplastic-like	131	XP_001767521	1.63E-08	GO:0005840	74	5.51E+01	43
THC_THT_c49667	hypothetical protein	130	BAD16821	2.56E-18	-	9.50E+01	79.337	41
THC_THT_c49704	glutelin	109	EEC72834	2.19E-16	GO:0016023	1.00E+02	77.0258	35
THC_THT_c49734	hypothetical protein PHAVU_010G056300g	118	XP_007134548	0.49509	-	63	32.7278	38
THC_THT_c49735	tpa: 40s ribosomal protein s6	116	NP_001050273	1.62E-15	GO:0003735	100	72.4034	35
THC_THT_c49745	ac079633_7 leucine-rich repeat transmembrane protein kinase 1	110	NP_001049184	0.00394019	-	66	39.2762	36
THC_THT_c49771	hypothetical protein	138	BAC16390	0.0148349	-	58	35.4242	48
THC_THT_c49781	retrotransposon ty3-gypsy subclass	113	AAM74390	9.73E-13	GO:0004523	9.70E+01	67.3958	34
THC_THT_c49885	predicted protein	106	XP_001753797	0.489233	-	57	32.7278	35
THC_THT_c49887	senescence-associated protein	151	XP_004492032	0.515244	-	53	32.7278	49
THC_THT_c49895	outer membrane protein a	144	XP_002540262	3.04E-05	-	69	4.39E+01	43
THC_THT_c49911	dihydroxy-acid dehydratase	114	KDD76640	5.73E-05	GO:0044249	7.30E+01	44.669	38
THC_THT_c49947	retrotransposon ty3-gypsy subclass	110	CAE05312	2.85E-08	GO:0004523	9.70E+01	54.299	36

THC_THT_c49963	type vi secretion system vgr family protein	137	KEH15984	4.14E-05	-	6.40E+01	45.8246	45
THC_THT_c50024	hypothetical protein (mitochondrion)	116	AEZ03709	9.07E-19	GO:0005739	100	8.05E+01	38
THC_THT_c50073	kinesin heavy chain-like	157	AAR89035	1.52E-09	GO:0005739	7.00E+01	57.7658	50
THC_THT_c50105	conserved hypothetical protein	110	XP_002539786	0.0377495	-	61	35.4242	34
THC_THT_c50158	hypothetical protein VITISV_015221	109	CAN60595	0.224802	-	64	33.8834	37
THC_THT_c50174	s-type anion channel slah1-like	113	AAD38288	2.59E-16	GO:0055085	1.00E+02	75.485	37
THC_THT_c50234	conserved hypothetical protein	107	XP_002537435	8.73E-09	GO:0043565	82	5.47E+01	35
THC_THT_c50267	dual-specificity rna methyltransferase 1-like	113	XP_008365113	2.26E-10	GO:0030488	8.00E+01	59.3066	36
THC_THT_c50363	retrotransposon ty3-gypsy subclass	135	CAE76024	1.11E-15	GO:0004523	8.90E+01	76.2554	46
THC_THT_c50395	f-box protein interaction domain containing expressed	120	BAD37944	2.27E-19	-	97	8.43E+01	40
THC_THT_c50401	atp synthase cf1 beta subunit	108	NP_050811	5.00E-05	GO:0046933	85	4.47E+01	34
THC_THT_c50441	hypothetical protein B456_011G261100	141	KJB73921	0.891371	-	55	31.5722	34
THC_THT_c50452	hypothetical protein SORBIDRAFT_01g009050	115	XP_002466511	0.00198838	-	52	37.7354	44
THC_THT_c50682	hypothetical protein	104	BAD68440	1.45E-13	-	9.10E+01	65.0846	34
THC_THT_c50773	ycf68 protein	151	ADK60808	5.58E-09	-	6.50E+01	53.1434	41
THC_THT_c50812	retrotransposon ty1-copia subclass	118	AAL78103	2.68E-19	GO:0004252	100	8.59E+01	39
THC_THT_c50865	zinc finger protein 7-like	101	XP_003562779	0.00494361	-	64	37.7354	34
THC_THT_c50895	hypothetical protein	110	BAD32093	0.01454	-	58	35.039	34
THC_THT_c50920	luminidependens-like protein	172	XP_001690070	0.0648292	-	56	36.1946	44

THC_THT_c50967	hypothetical protein NitaMp073	231	YP_173415	1.35E-05	-	68	4.54E+01	35
THC_THT_c51003	hypothetical protein OsI_12735	108	EEC75799	0.530137	-	59	32.3426	37
THC_THT_c51008	protein glutamine dumper 4-like	104	EEE66061	8.60E-09	GO:0016023	1.00E+02	52.373	34
THC_THT_c51042	hypothetical protein	118	BAD25823	1.41E-12	-	94	63.929	3.60E+01
THC_THT_c51057	programmed cell death 6-interacting partial	121	AEJ07955	1.69E-19	GO:0003676	1.00E+02	86.6557	40
THC_THT_c51100	retrotransposon unclassified	151	ABA99480	1.71E-19	GO:0016023	9.10E+01	87.4261	49
THC_THT_c51126	l-carnitine dehydratase bile acid-inducible protein f	112	XP_011016736	8.45E-09	-	7.50E+01	55.4546	37
THC_THT_c51192	hypothetical protein MIMGU_mgv11b017415 mg	181	EYU24217	0.0122918	-	70	38.1206	37
THC_THT_c51197	pentatricopeptide repeat-containing protein mitochondrial-like	104	CBI18917	2.12E-07	-	7.30E+01	51.2174	34
THC_THT_c51352	transposon mutator sub-class	122	CAD41543	4.87E-18	GO:0008270	9.20E+01	82.8037	40
THC_THT_c51405	retrotransposon ty3-gypsy subclass	129	CAE01888	2.67E-17	GO:0004523	9.70E+01	80.8777	42
THC_THT_c51463	plasma membrane atpase 4-like	109	XP_009381563	3.59E-10	GO:0098655	8.80E+01	59.6918	34
THC_THT_c51540	retrotransposon unclassified	114	AAZ06210	1.52E-11	GO:0005739	91	6.01E+01	34
THC_THT_c51689	carbamoyl-phosphate synthase small chloroplastic-like isoform x2	110	XP_006580325	1.06E-04	GO:0071704	7.60E+01	43.5134	34
THC_THT_c51690	strictosidine synthase precursor	125	EAZ28597	6.40E-22	GO:0005739	1.00E+02	90.8929	41
THC_THT_c51729	hypothetical protein OsI_15236	105	EEC76953	0.0125275	-	71	37.3502	35
THC_THT_c51804	OJ1116_C07.7	166	BAC00690	0.0133621	-	53	37.7354	41
THC_THT_c51828	f-box domain containing protein	111	EEC74783	0.00279067	-	66	39.6614	36

THC_THT_c51864	nadh dehydrogenase subunit j	120	YP_008757442	1.11E-20	GO:0005886	100	85.8853	39
THC_THT_c51962	retrotransposon ty1-copia subclass	129	BAD81143	2.11E-20	GO:0003676	100	83.1889	42
THC_THT_c52180	transposon en spm sub-class	150	AAX95063	1.88E-17	GO:0016023	1.00E+02	78.9518	36
THC_THT_c52239	rna polymerase beta subunit	107	AGW31114	2.18E-08	GO:0003899	8.20E+01	54.6842	34
THC_THT_c52261	polyribonucleotide nucleotidyltransferase	117	XP_001782634	2.48E-07	GO:0003723	78	5.16E+01	37
THC_THT_c52290	retrotransposon ty1-copia subclass	121	AAP44605	4.29E-18	GO:0003677	1.00E+02	82.8037	39
THC_THT_c52342	retrotransposon unclassified	109	CAE02251	2.51E-13	GO:0004523	9.40E+01	68.9366	36
THC_THT_c52476	retrotransposon unclassified	113	ABA94955	1.27E-11	GO:0009536	8.60E+01	63.1586	36
THC_THT_c52487	pura1_ricco ame: full=adenylosuccinate synthetase chloroplastic short=ampsase 1	114	XP_002535199	0.568453	-	47	32.7278	38
THC_THT_c52525	chaperone protein chloroplastic-like	112	XP_009351046	5.53E-05	-	6.90E+01	44.669	36
THC_THT_c52542	gdsl esterase lipase at4g26790-like	125	NP_001058145	1.14E-16	GO:0016788	100	77.0258	3.70E+01
THC_THT_c52553	retrotransposon unclassified	133	AAK50597	2.30E-20	GO:0004523	9.70E+01	88.9669	41
THC_THT_c52610	nicotianamine aminotransferase a	118	EEC72975	9.82E-14	GO:0030170	9.10E+01	69.3218	35
THC_THT_c52635	conserved hypothetical protein	112	XP_002535457	7.18E-19	GO:0003676	100	8.32E+01	37
THC_THT_c52656	hypothetical protein CICLE_v100111082mg, partial	115	XP_006428914	0.0875586	-	54	33.4982	35
THC_THT_c52674	retrotransposon ty3-gypsy subclass	104	AAX94789	0.00786768	-	64	36.965	34
THC_THT_c52737	pentatricopeptide repeat-containing protein at5g15300-like	112	AAL84319	1.17E-17	-	1.00E+02	80.8777	37

THC_THT_c52750	hypothetical protein SORBIDRAFT_08g000660	120	XP_002441678	0.273808	-	57	33.8834	35
THC_THT_c52843	chaperonin cpn60-mitochondrial	105	EMT21164	4.80E-07	GO:0005507	8.50E+01	50.447	34
THC_THT_c52849	amidohydrolase -like	133	EEE52322	0.00490615	-	69	39.2762	39
THC_THT_c52922	conserved hypothetical protein	116	XP_002534887	0.0142957	-	67	37.3502	34
THC_THT_c52995	probable wrky transcription factor 70-like	106	EAY99043	4.43E-14	GO:0043565	9.70E+01	68.9366	34
THC_THT_c53005	formate dehydrogenase, putative	134	XP_002536031	1.53E-10	GO:0044710	78	6.08E+01	38
THC_THT_c53059	e3 ubiquitin-protein ligase os03g0188200-like	166	EAZ36533	3.42E-32	GO:0016023	1.00E+02	115.931	54
THC_THT_c53077	tyrosyl-trna synthetase	127	XP_001699082	5.67E-05	GO:0006412	68	4.47E+01	38
THC_THT_c53099	auxin-responsive family protein	107	EAY88886	1.46E-14	GO:0016021	1.00E+02	71.2478	35
THC_THT_c53100	hth-type transcriptional regulator	148	EDQ49166	3.06E-05	-	7.10E+01	45.4394	38
THC_THT_c53307	1211235ad orf 77	105	A05190	0.00764658	-	71	35.8094	35
THC_THT_c53309	two component histidine	123	XP_002535010	0.493775	-	69	33.113	36
THC_THT_c53373	predicted protein	126	XP_002876409	0.904971	-	55	30.8018	36
THC_THT_c53397	small heat shock chloroplastic-like	123	XP_009372878	0.00422396	-	63	38.5058	38
THC_THT_c53402	low quality protein: f-box protein pp2-b15-like	138	XP_007208595	0.237958	-	59	33.8834	47
THC_THT_c53470	glutamate synthase	120	Q03460	3.73E-10	GO:0055114	77	6.01E+01	35
THC_THT_c53513	zinc finger	120	EEC73943	1.31E-12	GO:0008270	9.40E+01	65.4698	34
THC_THT_c53521	retrotransposon unclassified	117	ABA95676	2.79E-14	GO:0036459	9.20E+01	68.9366	38
THC_THT_c53533	adenylyltransferase and sulfurtransferase mocs3-like	117	XP_004491427	0.727898	-	56	32.3426	37

THC_THT_c53535	nad -binding rossmann-fold superfamily protein	114	ABS17662	2.28E-05	-	6.70E+01	45.0542	37
THC_THT_c53549	leucine--trna mitochondrial	125	XP_009350805	1.15E-08	GO:0048731	7.00E+01	55.8398	40
THC_THT_c53578	low quality protein: proline-rich receptor-like protein kinase perk14	111	BAD01722	2.18E-09	-	77	5.58E+01	36
THC_THT_c53591	Os07g0191900	142	NP_001059097	5.70E-06	-	63	4.54E+01	47
THC_THT_c53602	copia-type pol polyprotein	124	CAD39877	3.80E-18	GO:0003964	8.90E+01	83.1889	38
THC_THT_c53705	probable lrr receptor-like serine threonine-protein kinase at1g56130-like	109	EEC78861	8.99E-18	GO:0006468	1.00E+02	80.8777	36
THC_THT_c53724	fumarate hydratase mitochondrial	164	XP_002523133	7.16E-13	GO:0045239	80	5.82E+01	36
THC_THT_c53737	abc transporter c family member 4-like	168	CAD59594	2.28E-19	GO:0055085	100	8.43E+01	40
THC_THT_c53755	hypothetical protein OsI_14937	176	EAY93131	0.00195071	-	70	40.817	34
THC_THT_c53772	glucosamine-fructose-6-phosphate aminotransferase, putative	108	XP_002535681	1.25E-13	GO:0004360	97	6.93E+01	36
THC_THT_c53810	aminotransferase-like protein	103	AAM01066	1.18E-13	GO:0008483	94	69.707	3.50E+01
THC_THT_c53823	hypothetical protein OsI_29235	155	EAZ06990	3.26E-19	-	9.10E+01	85.5001	46
THC_THT_c53859	low quality protein: beta-galactosidase 9-like	128	XP_006656180	1.37E-20	GO:0005975	1.00E+02	89.7373	41
THC_THT_c53935	PREDICTED: uncharacterized protein LOC105037323	128	XP_010911304	0.748392	-	87	31.9574	39
THC_THT_c54003	ac091238_3 gag-pol precursor	144	AAK70625	0.958433	-	55	32.7278	38
THC_THT_c54048	ribosomal protein s7	106	XP_011016126	7.73E-09	GO:0003735	8.50E+01	55.8398	35

THC_THT_c54089	acetolactate synthase small subunit chloroplastic-like	123	CAN67345	3.93E-07	GO:0009082	8.00E+01	49.2914	35
THC_THT_c54112	cinnamyl alcohol dehydrogenase 5	190	EMS45840	2.88E-04	GO:0046872	7.30E+01	43.1282	34
THC_THT_c54116	nbs-lrr partial	119	AFE62433	1.18E-17	GO:0043531	100	78.9518	39
THC_THT_c54121	formiminotransferase-like isoform x3	119	AFW65332	0.0162554	-	61	36.965	42
THC_THT_c54238	unknown protein	131	ABK34476	1.08E-16	GO:0009536	9.70E+01	78.9518	38
THC_THT_c54257	cysteine-rich receptor-like protein kinase 24	162	XP_010439188	0.635362	-	50	33.113	38
THC_THT_c54275	nadh oxidase	108	XP_005846937	5.53E-04	GO:0044699	74	4.16E+01	35
THC_THT_c54277	cystathionine gamma synthase	145	NP_001064540	0.973104	-	47	32.3426	48
THC_THT_c54309	transposon mutator sub-class	141	AAV32210	3.68E-13	GO:0016020	8.90E+01	68.9366	39
THC_THT_c54370	conserved hypothetical protein	148	XP_002535050	0.00475734	-	67	36.965	37
THC_THT_c54402	glycosyl hydrolase family protein	104	XP_006404419	0.00295827	-	63	39.6614	38
THC_THT_c54418	hypothetical protein	117	BAI39791	0.284714	-	60	31.9574	38
THC_THT_c54433	glyceraldehyde-3-phosphate dehydrogenase	103	ABR25332	4.49E-16	GO:0005737	1.00E+02	73.559	34
THC_THT_c54437	callose synthase 5-like	105	XP_008780822	5.40E-16	GO:0016021	1.00E+02	73.1738	35
THC_THT_c54440	aquaporin 2	116	AAV80229	6.74E-12	GO:0016021	8.60E+01	63.1586	38
THC_THT_c54450	conserved hypothetical protein	160	XP_002540586	6.73E-06	-	59	4.51E+01	57
THC_THT_c54466	hypothetical protein SORBIDRAFT_1211s002020	199	XP_002488956	1.88E-08	-	72	52.373	43
THC_THT_c54485	hypothetical protein VITISV_006524	137	CAN79826	0.176527	-	51	34.6538	43
THC_THT_c54497	twitching mobility	127	XP_002534939	2.88E-07	GO:0006810	81	5.12E+01	37
THC_THT_c54501	BnaCnng67370D	109	CDY70240	0.267264	-	61	33.113	36
THC_THT_c54552	hypothetical protein SORBIDRAFT_1211s002020	180	XP_002488956	4.72E-11	-	75	58.9214	44

THC_THT_c54570	retrotransposon ty3-gypsy subclass	111	ABG22099	1.18E-06	-	7.60E+01	49.2914	34
THC_THT_c54609	retrotransposon unclassified	112	AAM93461	1.20E-16	GO:0008270	100	7.47E+01	36
THC_THT_c54663	putative polyprotein	104	AAQ56486	1.29E-05	GO:0046872	7.30E+01	46.595	34
THC_THT_c54717	cleavage and polyadenylation specificity factor subunit 3-ii isoform x1	123	NP_001063107	1.80E-20	GO:0090305	100	88.9669	41
THC_THT_c54809	dna polymerase delta catalytic subunit-like	105	XP_006339225	1.51E-10	GO:0003887	7.90E+01	60.8474	34
THC_THT_c54817	hutu_trirp ame: full=urocanate hydratase short=urocanase ame: full=imidazolonepropionate hydrolase	106	P53385	3.41E-09	GO:0019557	8.80E+01	56.6102	35
THC_THT_c54844	heat shock protein 70	117	CAA10980	1.53E-20	GO:0005524	1.00E+02	83.9593	38
THC_THT_c55035	histidine biosynthesis bifunctional protein chloroplastic	180	CDP00032	0.00279773	-	61	39.6614	39
THC_THT_c55166	predicted protein	116	BAK02440	0.00303877	-	67	39.6614	37
THC_THT_c55174	rrna intron-encoded homing endonuclease	178	EXC10888	1.89E-22	GO:0004519	6.40E+01	91.6633	79
THC_THT_c55293	probable 1-deoxy-d-xylulose-5-phosphate synthase chloroplastic	157	ERN07247	6.97E-09	GO:0044710	7.20E+01	55.4546	37
THC_THT_c55319	predicted protein	103	XP_001786556	1.11E-07	GO:0004129	82	5.20E+01	34
THC_THT_c55390	rna 2 - tpt1 family protein	177	KEH29088	0.541437	-	60	33.113	50
THC_THT_c55429	calpain-type cysteine protease dek1-like	133	AAL38190	8.36E-11	GO:2000014	8.80E+01	60.8474	34
THC_THT_c55447	3-oxoacyl-	118	XP_002535079	5.46E-09	GO:0008667	79	53.5286	39
THC_THT_c55572	retrotransposon unclassified	205	CAD39933	2.37E-16	GO:0004523	9.40E+01	67.781	37
THC_THT_c55576	retrotransposon ty3-gypsy sub-class	131	AAQ56463	6.30E-21	GO:0016023	1.00E+02	90.1225	42
THC_THT_c55582	cell wall	175	BAD29155	2.98E-08	GO:0009536	68	5.47E+01	57

THC_THT_c55592	web family protein chloroplastic-like	184	XP_011012023	0.638917	-	56	33.4982	41
THC_THT_c55596	retrotransposon ty3-gypsy subclass	126	CAE03652	8.36E-22	GO:0003964	9.50E+01	93.9745	42
THC_THT_c55598	OSJNBa0079A21.2	131	CAE02013	1.42E-04	-	6.00E+01	43.8986	41
THC_THT_c55600	transposon mutator sub-class	154	ABF95899	1.68E-18	GO:0016020	8.40E+01	84.7297	51
THC_THT_c55607	60s ribosomal protein l22-2	195	NP_001050078	2.03E-24	GO:0005840	98	95.5153	64
THC_THT_c55616	cell wall-associated partial	234	CDM84611	1.35E-09	-	8.50E+01	56.9954	35
THC_THT_c55617	retrotransposon ty3-gypsy subclass	117	AAV31300	3.44E-17	GO:0004523	97	8.05E+01	38
THC_THT_c55624	retrotransposon unclassified	184	ABA91114	0.00388369	-	90	34.6538	43
THC_THT_c55626	unknow protein	182	EPS74505	1.00E-07	-	7.00E+01	52.373	40
THC_THT_c55640	formaldehyde dehydrogenase s-formylglutathione hydrolase	144	KIZ06980	3.59E-07	GO:0071704	7.20E+01	51.2174	37
THC_THT_c55649	retrotransposon ty3-gypsy subclass	167	AAV31373	3.49E-21	GO:0003964	1.00E+02	92.8189	43
THC_THT_c55650	PREDICTED: uncharacterized protein LOC104214891	157	XP_009762916	0.744882	-	52	33.113	46
THC_THT_c55652	xs domain containing	196	BAC84465	1.37E-22	GO:0005739	83	8.97E+01	60
THC_THT_c55655	solo-ltr retrotransposon protein	170	ABC18340	0.0576017	-	83	35.8094	37
THC_THT_c55691	retrotransposon unclassified	184	CAE05318	2.06E-07	-	6.30E+01	50.447	47
THC_THT_c55693	retrotransposon ty1-copia subclass	147	BAC21536	8.74E-10	GO:0003676	8.80E+01	55.8398	34
THC_THT_c55712	AC078839_20Hypothetical protein	156	AAK13104	1.48E-07	-	6.90E+01	53.1434	43
THC_THT_c55722	hypothetical protein JCGZ_06007	139	KDP20462	6.74E-04	-	5.70E+01	39.2762	35
THC_THT_c55754	retrotransposon ty3-gypsy subclass	211	ABA98792	6.34E-07	-	6.60E+01	41.5874	36

THC_THT_c55760	retrotransposon unclassified	106	ABA94516	8.50E-13	-	9.40E+01	67.3958	34
THC_THT_c55796	26s proteasome non-atpase regulatory subunit 6-like	110	EEC73534	2.49E-14	GO:0006470	9.70E+01	72.0182	36
THC_THT_c55838	retrotransposon ty3-gypsy subclass	104	AAQ56341	3.34E-07	GO:0004523	7.60E+01	51.2174	34
THC_THT_c55845	conserved hypothetical protein	149	XP_002536962	0.119794	-	62	35.039	37
THC_THT_c55847	senescence-associated protein	207	BAB33421	2.22E-16	-	8.30E+01	76.6406	43
THC_THT_c55852	hgwp repeat containing protein	108	AAQ56366	0.188504	-	64	33.8834	34
THC_THT_c55860	low affinity potassium transport system protein	103	XP_002534890	0.115045	-	67	34.6538	34
THC_THT_c55873	ac078944_27 retroelement	149	BAD62523	2.20E-06	-	6.50E+01	46.595	43
THC_THT_c55876	retrotransposon unclassified	125	BAD53734	3.71E-08	-	7.60E+01	50.447	38
THC_THT_c55889	retrotransposon unclassified	181	AAM00989	5.03E-14	GO:0003676	8.00E+01	70.8626	46
THC_THT_c55902	hypothetical protein	110	BAD46544	0.446437	-	58	31.9574	34
THC_THT_c55915	hgwp repeat containing	263	ABA98576	2.39E-12	GO:0005739	5.90E+01	68.1662	79

Supplemental Table S4. List of *apriori* genes

Sl.No.	Locus_ID	Chr #	Homolog (description)
1	LOC_Os01g178300	1	OsCDT3
2	LOC_Os01g46350	1	proteins of unknown function
3	LOC_Os01g53090	1	pathogen-related protein, putative
4	LOC_Os01g56080	1	expressed protein
5	LOC_Os01g64120	1	2Fe-2S iron-sulfur cluster binding
6	LOC_Os01g64890	1	CorA-like magnesium transporter
7	LOC_Os01g69010	1	(SbMATE) MATE efflux protein
8	LOC_Os01g69020	1	retrotransposon protein, putative
9	LOC_Os01g0716400	1	SAM-dependen methyltransferase
10	LOC_Os02g03900	2	(Nrat1) metal transporter Nramp6
11	LOC_Os02g09390	2	cytochrome P450, putative
12	LOC_Os02g38200	2	dehydrogenase, putative, expressed
13	LOC_Os02g51930	2	cytokinin-O-glucosyltransferase 2
14	LOC_Os02g53130	2	nitrate reductase, putative, expressed
15	LOC_Os03g11734	3	MATE efflux protein
16	LOC_Os03g19170	3	GCRP7 - Glycine and cysteine rich
17	LOC_Os03g21950	3	fumarate hydratase
18	LOC_Os03g54790	3	(ALS1) ABC transporter, ATP-binding protein
19	LOC_Os03g55290	3	GASR3 - Gibberellin-regulated
20	Os03g0760800	3	GA-regulated protein family
21	Os03g0126900	3	hypothetical protein
22	LOC_Os04g34010	4	(ALMT1) aluminum-activated malate transporter
23	LOC_Os04g41750	4	expressed protein
24	LOC_Os04g49410	4	expansin precursor
25	LOC_Os05g02750	5	(ALS3 and STAR2) ABC transporter
26	LOC_Os05g02780	5	glycine-rich protein A3, putative
27	LOC_Os05g08810	5	phosphatidylinositol 3-kinase

28	LOC_Os05g09440	5	malic enzyme
29	LOC_Os06g36450	6	ferroportin1 protein
30	LOC_Os06g48060	6	(STAR1) ABC transporter, ATP-binding
31	LOC_Os07g23710	7	cytochrome P450, putative
32	LOC_Os07g34520	7	isocitrate lyase
33	LOC_Os07g39860	7	expressed protein
34	LOC_Os09g25850	9	WAX2, oxidoreductase;
35	LOC_Os09g30250	9	OsSub58 - Putative Subtilisin
36	LOC_Os10g12080	10	cytochrome P450, putative
37	LOC_Os10g13940	10	MATE efflux protein
38	LOC_Os10g26680	10	pectinesterase, putative, expressed
39	LOC_Os10g38080	10	OsSub61 - Putative Subtilisin homologue
40	LOC_Os10g42780	10	lrgB-like family protein, expressed
41	LOC_Os11g26850	11	erythronate-4-phosphate dehydrogenase
42	LOC_Os11g29680	11	expressed protein
43	LOC_Os11g29780	11	plant-specific domain TIGR01627
44	LOC_Os12g03890	12	major facilitator superfamily
45	LOC_Os12g05860	12	Cupin domain containing protein
46	LOC_Os12g12590	12	NADP-dependent oxidoreducta
47	LOC_Os03g07480	3	sugar transporter
48	LOC_Os12g44380	12	sugar transporter
49	LOC_Os10g26470	10	sugar transporter
50	LOC_Os02g58080	2	sugar transporter
51	LOC_Os02g36700	2	sugar transporter
52	LOC_Os11g43860	11	OsMHX
53	LOC_Os06g44150	6	OsMGT
54	LOC_Os04g32920	4	high affinity K transporter gene *OsHAK1*

Supplemental Table S5. Details of mapped QTLs

Sample	Transcript ID	Up/Down Regulated	Chromosome_japonica	Star_Japonica	End_Japonica	QTL_Info	log_2 Fold Change [Treated/Control]
ARC_ART	ARC_ART_c1640	Down_regulated	2	28716516	28716752	White-backed_planthopper_resistance_(Biotic_stress)_AQBG004	-5.369
ARC_ART	ARC_ART_c686	Down_regulated	1	33586869	33586682	ultraviolet-b_resistance_(Abiotic_stress)_AQCS001	-4.393
ARC_ART	ARC_ART_c89973	Down_regulated	2	28716573	28716659	planthopper_resistance_(Biotic_stress)_AQBG004	-5.212
IRC_IRT	IRC_IRT_c108013	Up_regulated	1	33586893	33586811	ultraviolet-b_resistance_(Abiotic_stress)_AQCS001	2.634
IRC_IRT	IRC_IRT_c24238	Down_regulated	4	32928321	32928208	ultraviolet-b_resistance_(Abiotic_stress)_AQCS001	-8.229
IRC_IRT	IRC_IRT_c10008	Down_regulated	2	28715924	28716006	spikelet_number_(Yield)_CQN53	-6.830
IRC_IRT	IRC_IRT_c105	Down_regulated	2	28721019	28721060	planthopper_resistance_(Biotic_stress)_AQBG004	-9.729
IRC_IRT	IRC_IRT_c107997	Down_regulated	2	28721009	28721061	white-backed_planthopper_resistance_(Biotic_stress)_AQBG004	-7.937

IRC_IRT	IRC_IRT_c108023	Down_regulated	9	16308	16234	spikelet_number_(Yield)_CQN53	-9.194
THC_THT	THC_THT_good_c10484	Up_regulated	3	26104000	26103930	leaf_length_(Anatomy)_AQHF031	2.415
THC_THT	THC_THT_c23349	Up_regulated	4	32927314	32927257	white-backed_planthopper_resistance_(Biotic_stress)_AQBG004	3.117
THC_THT	THC_THT_c240	Up_regulated	2	28715750	28715824	planthopper_resistance_(Biotic_stress)_AQBG004	2.196
THC_THT	THC_THT_c2412	Up_regulated	2	14246103	14246165	spikelet_number_(Yield)_CQN53	2.012
THC_THT	THC_THT_c41	Up_regulated	2	28715955	28716002	ultraviolet-b_resistance_(Abiotic_stress)_AQCS001	2.297
THC_THT	THC_THT_c41683	Up_regulated	4	32927512	32927444	planthopper_resistance_(Biotic_stress)_AQBG004	2.063
THC_THT	THC_THT_c641	Up_regulated	2	28721026	28721066	spikelet_number_(Yield)_CQN53	2.446
THC_THT	THC_THT_c8246	Up_regulated	2	28716078	28716143	planthopper_resistance_(Biotic_stress)_AQBG004	2.727
THC_THT	THC_THT_c8521	Up_regulated	2	28716635	28716717	planthopper_resistance_(Biotic_stress)_AQBG004	2.325
THC_THT	THC_THT_c8698	Up_regulated	3	13644920	13644977	spikelet_density_(Anatomy)_CQAS150	3.195
THC_THT	THC_THT_c8890	Up_regulated	9	13207	13265	planthopper_resistance_(Biotic_stress)_AQBG004	2.189

THC_THT	THC_THT_good_c1150	Down_regulated	3	19768510	19768706	tiller_number_(Vigor)_EQC0002	-2.415
THC_THT	THC_THT_good_c1166	Down_regulated	4	10611580	10611778	tiller_number_(Vigor)_EQC0002	-2.347
THC_THT	THC_THT_good_c1168	Down_regulated	8	22588381	22588592	seed_dormancy_(Vigor)_EQB0001	-2.807
THC_THT	THC_THT_good_c1222	Down_regulated	2	23369944	23369470	seed_dormancy_(Vigor)_EQB0001	-2.137
THC_THT	THC_THT_good_c1247	Down_regulated	4	11350837	11350731	plant_height_(Vigor)_AQED055	-2.152
THC_THT	THC_THT_good_c1257	Down_regulated	10	3344075	3344566	leaf_length_(Anatomy)_AQHF031	-2.285
THC_THT	THC_THT_good_c1277	Down_regulated	5	8505287	8505090	spikelet_sterility_(Sterility_or_fertility)_AQJ008	-2.184
THC_THT	THC_THT_good_c1374	Down_regulated	1	26858008	26858459	seed_dormancy_(Vigor)_EQB0001	-2.519
THC_THT	THC_THT_good_c2581	Down_regulated	5	14062128	14061772	root_number_(Vigor)_AQA022	-2.618
THC_THT	THC_THT_good_c3099	Down_regulated	3	25441034	25441173	root_number_(Vigor)_AQA022	-2.125
THC_THT	THC_THT_good_c3370	Down_regulated	5	17410415	17410128	leaf_length_(Anatomy)_AQHF031	-2.125
THC_THT	THC_THT_good_c416	Down_regulated	1	4843467	4843424	plant_height_(Vigor)_AQED055	-2.862
THC_THT	THC_THT_good_c514	Down_regulated	3	30652481	30653064	seed_dormancy_(Vigor)_EQB0001	-3.205

Supplemental Table S6. List of reported Al toxicity QTLs

Segment_No	Chromosome	Position	Start	End
1	1	35.2	35100000	35300000
2	1	38	37900000	38100000
3	**2**	27.61	27510000	27710000
4	6	5.81	5710000	5910000
5	9	18.81	18710000	18910000
6	12	3.19	3090000	3290000
7	12	23.36	23260000	23460000

Supplementary Table S7 Nucleotide diversity (π) estimates for genes Nrat1 (*LOC_Os02g03900*) and glycine-like protein A3 (*LOC_Os05g02780)* in four rice genotypes showing contrasting response to aluminium toxicity

Sl. No.	Genotype Code	Name	Response to Al toxicity tolerance	*Nrat1*		*Glycine-rich A3*		
				π (intronic region)	π (coding region)	π 5´ UTR	π (intronic region)	π (coding region)
1	AR	ARR 09	T	0.0005	*	0.01	0.001	0.0017
2	UR 29	Azucena	T	0.001	*	*	*	*
3	TH	Theruvii	S	*	*	0.066	0.006	0.005
4	IR	IR 1552	S	0.001	0.0085	0.012	0.006	0.005

Nipponbare sequence was taken as reference for diversity calculations. ‘*‘denotes no variation with respect to the reference genome. Terms tolerant (T) and susceptible (S) are based on hydroponics study conducted and acidic soil field performance
